# Comparative Proteomics Analysis of the Root Apoplasts of Rice Seedlings in Response to Hydrogen Peroxide

**DOI:** 10.1371/journal.pone.0016723

**Published:** 2011-02-10

**Authors:** Lu Zhou, Saleem A. Bokhari, Chun-Juan Dong, Jin-Yuan Liu

**Affiliations:** Laboratory of Molecular Biology and MOE Laboratory of Protein Science, School of Life Sciences, Tsinghua University, Beijing, People's Republic of China; Instituto de Biología Moleculary Celular de Plantas, Spain

## Abstract

**Background:**

Plant apoplast is the prime site for signal perception and defense response, and of great importance in responding to environmental stresses. Hydrogen peroxide (H_2_O_2_) plays a pivotal role in determining the responsiveness of cells to stress. However, how the apoplast proteome changes under oxidative condition is largely unknown. In this study, we initiated a comparative proteomic analysis to explore H_2_O_2_-responsive proteins in the apoplast of rice seedling roots.

**Methodology/Principal Findings:**

14-day-old rice seedlings were treated with low concentrations (300 and 600 µM) of H_2_O_2_ for 6 h and the levels of relative electrolyte leakage, malondialdehyde and H_2_O_2_ were assayed in roots. The modified vacuum infiltration method was used to extract apoplast proteins of rice seedling roots, and then two-dimensional electrophoresis gel analysis revealed 58 differentially expressed protein spots under low H_2_O_2_ conditions. Of these, 54 were successfully identified by PMF or MS/MS as matches to 35 different proteins including known and novel H_2_O_2_-responsive proteins. Almost all of these identities (98%) were indeed apoplast proteins confirmed either by previous experiments or through publicly available prediction programs. These proteins identified are involved in a variety of processes, including redox homeostasis, cell wall modification, signal transduction, cell defense and carbohydrate metabolism, indicating a complex regulative network in the apoplast of seedling roots under H_2_O_2_ stress.

**Conclusions/Significance:**

The present study is the first apoplast proteome investigation of plant seedlings in response to H_2_O_2_ and may be of paramount importance for the understanding of the plant network to environmental stresses. Based on the abundant changes in these proteins, together with their putative functions, we proposed a possible protein network that provides new insights into oxidative stress response in the rice root apoplast and clues for the further functional research of target proteins associated with H_2_O_2_ response.

## Introduction

Reactive oxygen species (ROS), including singlet oxygen (^1^O_2_), superoxide anions (O_2_^−^), hydrogen peroxide (H_2_O_2_) and hydroxyl radicals (HO·) are highly reactive and toxic, and they can lead to the oxidative destruction of cells. However, ROS have also been discovered to function as important regulators of many biological processes, such as cell growth and development, hormone signaling and stress responses [1]. ROS imbalance is closely linked to a wide range of oxidative destruction, so that the cellular redox condition should be tightly regulated. Unlike other ROS, H_2_O_2_ is non-radical, carrying no net charge, and has a comparatively longer half-life, which makes it a more likely long-distance signaling molecule [2]. As a physiological indicator of stress intensity, when plants are challenged with biotic and/or abiotic stresses, H_2_O_2_ can accumulate and be used to activate stress-responsive genes [3]. Therefore, an omics analysis for H_2_O_2_-response may be of paramount importance for the understanding of the plant network to environmental stresses.

Until now, most studies on H_2_O_2_ in plants have focused on changes in transcriptional levels [4]–[6]. Desikan *et al.* reported that more than 170 non-redundant ESTs were regulated by H_2_O_2_ in Arabidopsis [4]. Another study revealed that 349 transcripts were up-regulated and 88 were down-regulated by high levels of light-induced H_2_O_2_ in catalase-deficient Arabidopsis plants [5]. Similarly, 713 ESTs were found to be regulated by high levels of light-induced H_2_O_2_ in catalase-deficient tobacco plants [6]. Although large-scale transcriptome studies have revealed the transcriptional dynamics of a large number of antioxidative genes, the molecular mechanisms involved in the response to H_2_O_2_ can not be thoroughly characterized without information about their functional products of these genes. In our previous work, a comparative proteomic study on rice seedling leaves under H_2_O_2_ stress revealed how the leaves adapt to oxidative challenge [3], which provides new insights into the oxidative stress responses of rice leaves; however, little is known about how the proteome changes under oxidative stress in other organs of rice plants, such as the root, which is a main exchange interface between plants and their environments.

The plant cell is enclosed by the apoplast, which consists of the cell wall and the intercellular spaces [7]. As the first compartment of the plant cell, the apoplast, especially the root apoplast, is important for all of the plant's interactions with its environment. It can sense environmental changes and stress signals and then transfer them into the cell interior to trigger a whole cell response [7]. In addition to signal perception and transduction, apoplast proteins can also be used for cell wall modification and reconstruction, as well as defense responses, although they comprise only 5%–10% of the wall dry weight [7]. Using a bioinformatics approach, it is estimated that there are more than 1000 different apoplastic proteins in Arabidopsis [8]. However, to date, the biolgoical functions of only a few apoplast proteins have been reported [9]. Recently, there have been several reports about leaf apoplast proteomes under biotic or abiotic stresses, such as salt in tobacco [10], wounding in Medicago [11], dehydration in chickpea and rice [12], [13], virus infection in oilseed rape and tobacco [14], [15], manganese toxicity in cowpea [16], and boron deficiency in *Lupinus albus* [17]. These studies broadened our understanding of the complicated regulation of leaf apoplast proteins. Nevertheless, few studies have addressed the dynamic changes in the proteome of the plant root apoplast in response to oxidative stress, especially to H_2_O_2_, which serves as a physiological indicator of biotic and abiotic stress intensity.

In this study, we initiated a proteomic investigation into functional H_2_O_2_-responsive proteins in the root apoplast of rice seedlings using two-dimensional electrophoresis (2-DE). Under H_2_O_2_ stress, 58 protein spots were found to be differentially expressed, of which 54 were successfully identified by mass spectrometry and represented 35 different proteins, of which more than one-third are newly identified at the protein level. The abundant changes in these identified proteins, as well as their putative functions, are consistent with the proposed role of the apoplast in H_2_O_2_ signal perception, transduction and defense response. The correlation between these H_2_O_2_-responsive proteins and apoplast response was explored. This is the first proteomic study of the apoplast in response to H_2_O_2_, and the results greatly expand our knowledge about the complexity of apoplast proteins in rice and provide a framework for further functional studies of each identified protein.

## Results and Discussion

### Preparation of apoplast proteins from rice seeding roots

**Figure 1:**
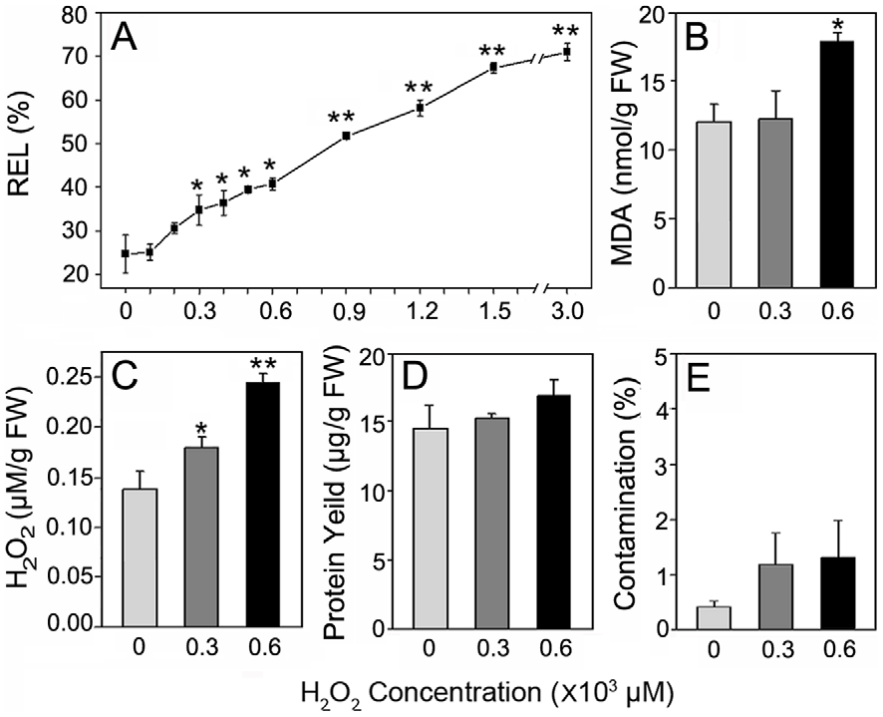
Effects of H_2_O_2_ treatments in rice seedlings, protein yields and intracellular contamination ratio. (A) Effects of H_2_O_2_ treatments on REL in rice seedlings. (B) Effects of H_2_O_2_ treatments on MDA concentrations in rice seedling roots. (C) Accumulation of apoplastic H_2_O_2_ in rice seedling roots. (D) Protein yields from vacuum infiltrates. (E) Intracellular contamination of vacuum infiltrates. Two-week-old seedlings were treated with H_2_O_2_ at different concentrations for 6 h. Values are means of independent replicates±SE, n = 3. Levels of significance of *T*-test are shown by * and ** for *p*<0.05 and 0.01, compared to the control.

To minimize the contamination of apoplast proteins with intracellular proteins, it is absolutely critical to adopt appropriate H_2_O_2_ concentrations for the treatments of rice seedling roots. Electrolyte leakage is an important indicator of the cell membrane damage under adverse conditions including oxidative stress [18]. To evaluate the effect of H_2_O_2_ stress on the cell membrane, changes in relative electrolyte leakage (REL) were measured in rice seedling roots treated with different concentrations of H_2_O_2_. As shown in Figure 1A, REL increased slightly from 24.76% (control) to 34.82% (300 µM) or 40.83% (600 µM) as the concentration of H_2_O_2_ increased from 0 to 600 µM, indicating that these treatments with H_2_O_2_ indeed triggered some root responses. When the concentrations of H_2_O_2_ were increased to 900 µM or higher, REL jumped to more than 50%, suggesting some oxidative damage to the cell membrane. It has been reported that, if the seedling REL of the chilling-sensitive rice cultivar TN.1 reaches 50% or higher under chilling (5°C) treatment, the survival ratio is less than 70%, demonstrating severe oxidative damage to the cell membrane [19]. These data suggest that treatments with higher than 600 µM H_2_O_2_ may be inappropriate for this study. The effect of H_2_O_2_ concentrations on rice seedling roots was further evaluated by an assay for malondialdehyde (MDA), which is a breakdown product of membrane lipid peroxidation and can also be used as a marker to indicate the degree of damage of the cell membrane under oxidative stress [20]. As presented in Figure 1B, there were almost no changes in the MDA concentration between the control (12.1±1.22 nmol/g FW) and samples treated with 300 µM H_2_O_2_ (12.3±2.03 nmol/g FW). Even under the 600 µM H_2_O_2_ treatment, the MDA concentration increased only slightly to 18.2±0.6 nmol/g FW (0.01<p<0.05, compared to the control) (Figure 1B). Based on the results of the REL and MDA assays, it is clear that 300 µM H_2_O_2_ led to some responses in the cell membrane without obvious lipid peroxidation change. However, 600 µM H_2_O_2_ caused slight oxidative damage to the cell membrane with increased lipid peroxidation and electrolyte leakage. Additionally, the levels of apoplastic H_2_O_2_ in roots treated with exogenous H_2_O_2_ of 300 and 600 µM H_2_O_2_ concentrations were increased by 30.8% and 76.8% over control, respectively, showing a dose-dependent pattern of accumulation (Figure 1C). Furthermore, 600 µM H_2_O_2_ treatment for 6 h caused the seedling leaves to roll inward, and the net photosynthetic rate was declined by about 20% over control [3]. Taken together, these results suggest that these two concentrations represent different oxidative conditions and rice seedlings could tolerate these concentrations without serious destruction to the cell membrane, therefore, 300 µM and 600 µM H_2_O_2_ were adopted for the treatments in this study.

A modified vacuum infiltration method was employed to extract apoplast proteins from the H_2_O_2_-treated or control rice seedling roots. The protein yields were about 15 µg/g FW (Figure 1D) although the protein yield of samples treated with 600 µM H_2_O_2_ slightly increased compared to the other conditions. However, the difference among the three sample sets was not stastically significant (p>0.05), indicating H_2_O_2_ had no obvious effect on the total apoplast protein amount. The levels of intracellular contamination in the vacuum infiltrates (VI) were quantitatively evaluated from the enzyme activity of glucose-6-phosphate dehydrogenase (G6PDH), which is a cytoplasmic enzyme that can be used as a specific marker for any plasma membrane damage that may occur during apoplast extraction by the vacuum infiltration procedure [21]. The average contamination percentage was 0.43%, 1.2% and 1.32% for the control, 300 µM and 600 µM H_2_O_2_ treated samples, respectively (Figure 1E). According to the previous studies, less than 3% is considered to be negligible contamination [17]. Hence, the apoplast extracts prepared in this study were relatively pure and were suitable for the subsequent proteomic analysis.

### Apoplast proteome of rice seedling roots under H_2_O_2_ stress

**Figure 2:**
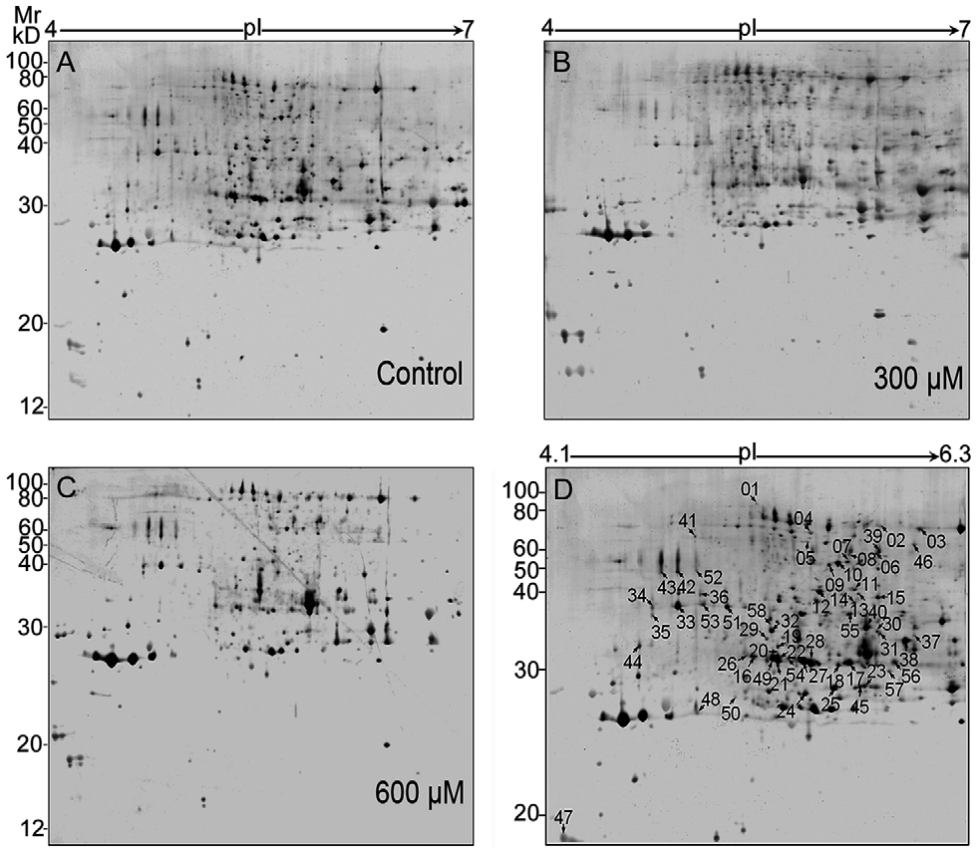
2-DE image analysis of rice root apoplast proteome under H_2_O_2_ treatments. (A–C) Set of three gels corresponding to apoplast protein samples treated with 0 (A), 300 (B), and 600 µM (C)of H_2_O_2_. (D) A representative gel showing the identified differentially expressed protein spots. 200 µg of the protein sample was loaded onto 24 cm IPG strips, pH 4–7 and electrophoresed as described in the text. The gel (12.5%) was stained using a mass spectrometry compatible silver staining method. A total of 58 protein spots were expressed differentially in response to the oxidative treatments, with *p*<0.05. Among these, 54 spots corresponding to 35 proteins are listed in Table S1.

Using the modified vacuum infiltration procedure in combination with MS compatible silver staining, the average number of reproducible spots in this study reached around 400 spots on each 2-DE gel (Figure 2), of which the spot number is greatly increased compared to the previous report of rice root apoplast (around 100 spots) [22]. The three reproducible gel maps for the control and two different treatments are shown in Figure 2A–C. From the profile analysis combined with statistical tests, a total of 58 stained spots were found to show significant changes (*p*<0.05) and are marked in Figure 2D. Most of these spots (51 spots) had a greater than 2-fold change in abundance under at least one of the H_2_O_2_ treatments (Table S1).

**Figure 3:**
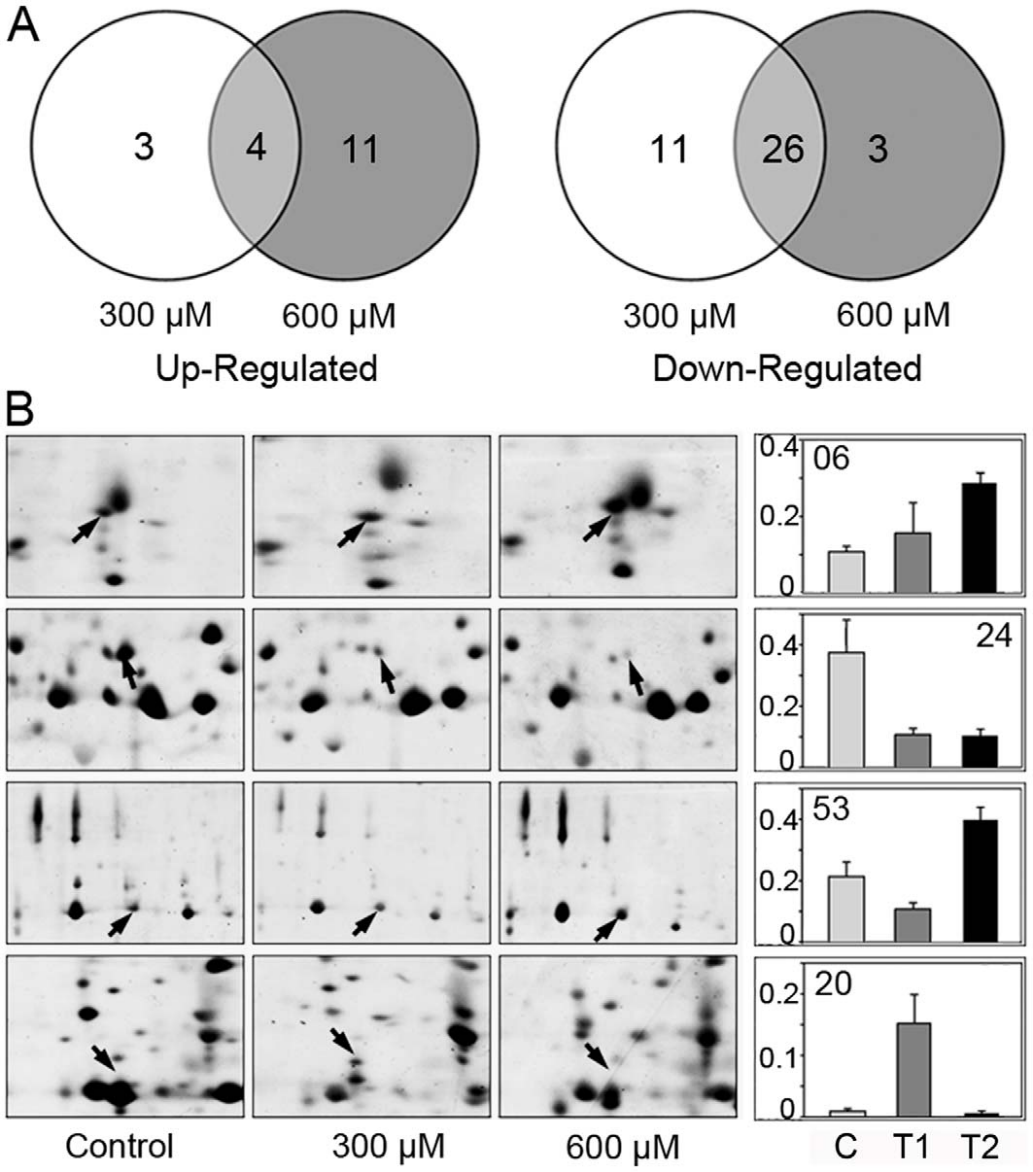
Change profile of the differentially expressed protein spots. (A) Venn diagram analysis of the differentially expressed protein spots in the apoplast of rice seedling roots treated with 300 µM and 600 µM H_2_O_2_. The number of differentially expressed protein spots with up- (left panel) or down-regulation (right panel) under a given concentration of H_2_O_2_ are also shown. (B) Typical examples of spSots showing different profiles.

Figure 3A shows the number of differentially displayed spots under different H_2_O_2_ treatments, and a venn diagram illustrates the overlap of these spots. Among the 18 up-regulated spots, 4 spots co-increased in abundance, and among the 40 down-regulated spots, 26 spots co-decreased in abundance under the two H_2_O_2_ treatments (Figure 3A). Obviously, more than 50% of the differentially expressed spots (30 spots) exhibited a similar regulatory pattern under 300 and 600 µM H_2_O_2_ (Table S1). The expression levels of the other 28 spots increased or decreased under only one treatment (Figure 3A). Of these, 14 spots were found to be differentially expressed in response to 300 µM H_2_O_2_, while the other 14 spots showed significant changes under 600 µM H_2_O_2_ (Table S1), indicating that these spots were specifically responsive to a treatment concentration. Figure 3B shows examples (spot 06, 20, 24 and 53) representing the dynamic changes of differentially expressed proteins in response to H_2_O_2_ treatments. Together, these data suggest that plant cells are able to monitor different levels of stress intensity by modulating corresponding protein expression.

### Identification of the differentially expressed proteins

A total of 58 differentially expressed protein spots were subjected to in-gel digestion and analyzed by MALDI-TOF/TOF-MS, and 54 spots were successfully identified (Table S1). Of these, 33 spots were identified by PMF, and 21 spots were identified by MS/MS analysis (Table S2). The PMF images and annotated spectra by MS/MS analysis for all spots are shown in File S1 and File S2 in Supporting Information, respectively. Among the 54 identities, 53 are deposited in the current database as putative functional proteins, whereas spot 54 is a hypothetical rice protein with unknown function. To annotate its identity, its sequence was used as a query to search for homologs using BLASTP (www.ncbi.nlm.nih.gov/BLAST/). The corresponding homolog with the highest score was pyridoxamine 5'-phosphate oxidase in *Desulfotomaculum* with a 31% identity at the amino acid level, suggesting that it might have similar functions in rice. Additionally, for those peptides that matched several members of a protein family, the one with the highest score was selected (Table S3). Taken together, the 54 identities represent 35 different proteins (Table S1), and more than one-third of these identities are newly identified at the protein level.

**Figure 4:**
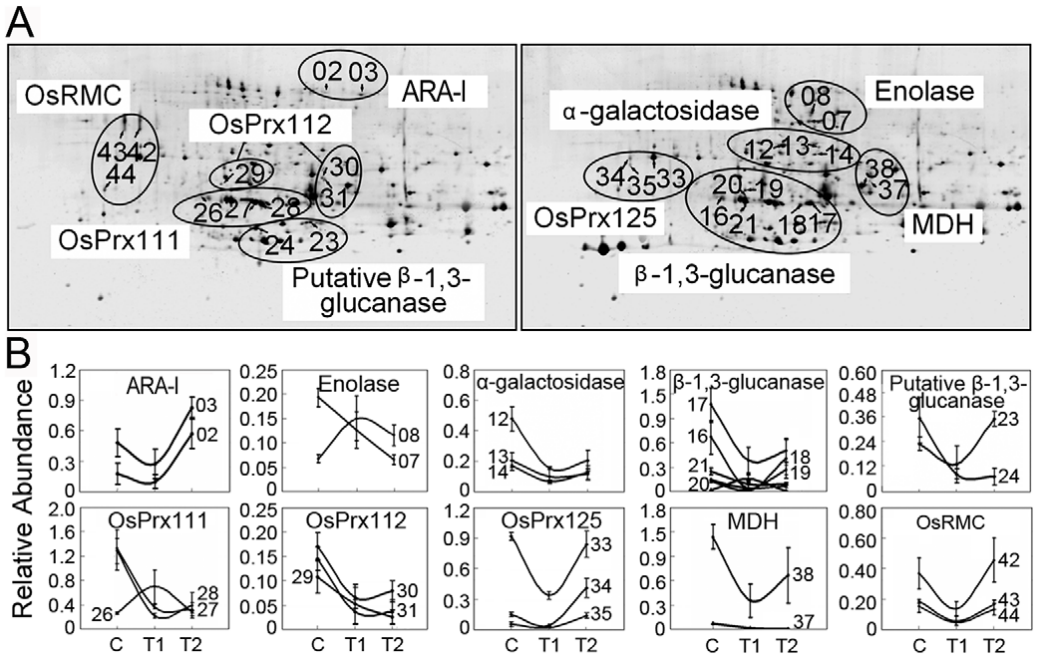
Close-up of possible isoforms detected by 2-DE (A) and their expression profile patterns (B). All 29 differentially expressed protein spots, matching 10 different proteins, are shown. T1 and T2 represent H_2_O_2_ treatments of 300 µM and 600 µM, respectively. ARA-I, α-arabinofuranosidse/β-D-xylosidase isoenzyme; Prx, peroxidase; MDH, malate dehydrogenase; RMC, root meader curling.

Moreover, among the 35 differentially expressed proteins, 10 proteins were present as multiple spots on the 2-DE gels, with one spot representing an isoform (Figure 4A). Of these, the isoforms for 8 proteins [α-L-arabinofuranosidase/β-D-xylosidase isoenzyme, enolase, putative α-galactosidases, a putative β-1,3-glucanase, two peroxidases (OsPrx112 and OsPrx125), a malate dehydrogenase and the DUF26 motif-containing protein, OsRMC], representing 20 identities showed similar up- or down-regulated changes in abundance in response to H_2_O_2_ treatment. The isoforms for the other two proteins (OsPrx111 and β-1,3-glucanase), representing 9 identities, exhibited opposite expression patterns (Figure 4B). Likewise, similar phenomena have been observed in previous proteomics studies [3], [23], [24], which are probably due to the posttranslational modifications. These results suggest that isoforms of a certain protein may play either the same or different roles in modulating cell responses to H_2_O_2_ treatments in rice seedlings.

The apparent *Mr* value predicted by SDS-PAGE has an error deviation of about ±10% compared to the theoretical value (Table S1). Among the 58 identified protein spots, 3 spots (spots 23, 24 and 45) representing 2 proteins (putative β-1,3-glucanase and Arm repeat protein) were found with observed *Mr* values that were much smaller than the theoretical values (less than 55%) (Table S1), suggesting that these proteins might be the partially degraded products of their intact proteins. This speculation is supported by the previous finding that protein degradation is enhanced in H_2_O_2_-treated rice seedlings [3]. Also, this apoplast protein degradation phenomenon was reported in a previous proteomic study of the chickpea extracellular matrix during dehydration stress [12].

### Subcellular localization prediction and functional classification of H_2_O_2_-responsive proteins

To confirm that all the proteins we identified were indeed apoplast proteins, their subcellular location were predicted by the TargetP program (www.cbs.dtu.dk/services/TargetP) [25]. As listed in Table S1, 36 of the 54 identities were predicted to be typical secretory proteins with signal peptide sequences. In plants, there have been many reports of the existence of non-classical proteins in the apoplast [8], [9], [26]–[29]. Moreover, several cytosolic proteins called moonlighting proteins have been experimentally shown to perform another function in the cell wall or outside the cell [30]–[32]. Therefore, we also used the SecretomeP set-up (http://www.cbs.dtu.dk/services/SecretomeP-1.0) [33] to inspect the non-classical secretory proteins, and 6 of the 18 spots were predicted to be putative leaderless secreted proteins (Table S4). For the remaining 12 spots, we determined their potential subcellular localization from reports in the literature. As listed in Table S4, 11 of the 12 spots have been reported to exist in the apoplast (except for spot 40), and most of the 11 spots have been detected in the apoplast by Western blot or immunocytochemistry in addition to proteomics [12], [27], [28], [34]–[40], with only two spots (spots 45 and 51) detected just by proteomic approaches [27], [41]. Taken together, 53 out of the 54 spots (98%) were confirmed to be localized in the apoplast either through publicly available prediction programs or according to previous references. Therefore, consistent with the results of the G6PDH enzyme assay (Figure 1E), our apoplast protein samples are of high quality, with no obvious cytoplasmic contamination, and can be used to reflect the actual apoplast proteome profile.

**Figure 5:**
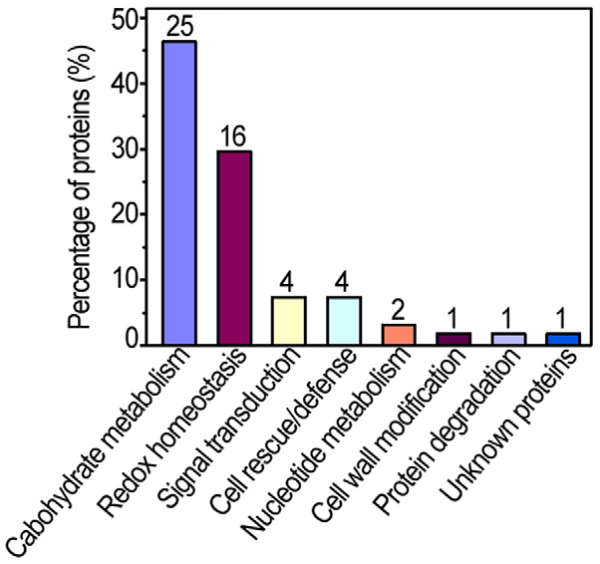
Functional classification and distribution of the 54 identified protein spots. The number represents the number of protein spots identified in each functional catalog.

To characterize the apoplast proteome response to H_2_O_2_, all 54 identified protein sequences were functionally classified by GoFigure (http//www.geneontology.org). All H_2_O_2_ responsive proteins were grouped into 8 major categories as shown in Figure 5 and Table S1. An impressive 45% of these identities belonged to functional categories including redox homeostasis, cell rescue/defense and signal transduction, suggesting the functional importance of these processes in the apoplastic response to H_2_O_2_ treatments.

### Carbohydrate metabolism in the root apoplast of rice seedlings under H_2_O_2_ Stress

One of the noteworthy aspects of this research is that 26 of the 54 H_2_O_2_-responsive protein spots were involved in carbohydrate metabolism (Figure 5). Of which 17 were glycosylhydrolases (GHs) that catalyze the hydrolysis of the glycosidic linkage. The result is remarkably similar to a previous report in which nearly 30% of apoplastic proteins are predicted to act on polysaccharides of the cell wall in *Arabidopsis* [8]. Polysaccharides, which make up around 90% of the apoplast, form a rigid structure to strengthen the plant cell wall and participate in cell-cell interactions and the defense responses [42]. In this work, most of the GHs such as α-galactosidase (spots 12–14), β-1,3-glucanases (spots 16–24), and β-1,3;1-4-glucanase precursor (spot 25) were found to be down-regulated in the H_2_O_2_-treated seedlings (, Figure S1A and Figure S2A). Suppression of these polysaccharide hydrolases under H_2_O_2_ stress might reduce the hydrolysis of glucan and other polysaccharides to alter the dynamic remodeling of the polysaccharides to withstand the deleterious effects of oxidative stress [43]. For example, the down regulation of α-galactosidase (EC 3.2.1.22) might lead to raffinose accumulation (Figure S2A), which plays effective roles in stabilizing the membrane [44] and scavenging ROS [45], and subsequently confers tolerance to various abiotic stresses [44], [46]. In comparison, the activities of α-L-arabinofuranosidases (spots 01-04) were up-regulated under at least one of the H_2_O_2_ treatments (Table S1, Figure S1A and Figure S2A). The α-L-arabinofuranosidase can catalyze the degradation of the carbohydrate moieties of arabinogalactan-proteins (AGPs), a family of highly glycosylated hydroxyproline-rich glycoproteins involved in intercellular signal transduction and stress responses [47], [48]. Therefore, the up-regulated α-L-arabinofuranosidase might make the AGP core protein accessible for providing signaling information to reinforce the cellular defense responses under oxidative stress.

Besides, proteins related to the synthesis of cell wall polysaccharides is also influenced by H_2_O_2_ treatments in rice seedlings. UDP-glucose pyrophosphorylase (UGPase, EC 2.7.7.9) (spot 06), a key enzyme in producing UDP-glucose from glucose-1-phosphate and UTP, was found to be up-regulated under H_2_O_2_ stress (Table S1, Figure 1SA and Figure S2A). As UDP-glucose is the substrate for the synthesis of cell wall polysaccharides, such as cellulose [49], the up-regulation of UGPase probably benefit for maintaining normal wall polysaccharide synthesis by providing UDP-glucoses to compensate for the reduced cytosolic nucleoside diphosphate sugars [50]. In agreement, a UGPase was indeed induced upon exposure to low doses of H_2_O_2_ in *Saccharomyces cerevisiae* [51]. Additionally, a cell wall modifying enzyme of pectinesterase (PME, spot 46) was found to be up-regulated upon H_2_O_2_ treatment (Table S1, Figure S1A and Figure S2A). PME (EC 3.1.1.11) could catalyze the specific demethylesterification of homogalacturonans to strengthen the cell wall. The methanol released during this process may diffuse into the cytoplasm and be converted to formate, which is used for the biosynthesis of sugars, amino acids, purines and organic acids during plant defense metabolism [52], [53].

Interestingly, some glycolytic enzymes, such as phosphoglycerate mutase (PGM, EC 5.4.2.1) (spot 05) and enolases (EC 4.2.1.11) (spots 07-10, representing 3 proteins), were also identified in this study. These enzymes catalyze the initial steps of the glycolytic pathway and are known to be in the cytoplasm. However, accumulating experimental data illustrate that many glycolytic enzymes function as cell wall components [28]–[30], [37], [38], although their exact function in the apoplast is still unclear. The differential expression profiles of these glycolytic enzymes under H_2_O_2_ treatments (Table S1 and Figure S2A) indicate their potential roles in the root apoplast under oxidative stress.

### Redox state regulation in the root apoplast of rice seedlings under H_2_O_2_ stress

Another noteworthy aspect in this study is that nearly half (24/54) of the identities are related to redox state regulation. These proteins can be classified into three functional subgroups: redox homeostasis (spots 26-41), signal transduction (spots 42-45) and cell rescue/defense (spots 47-50) (Table S1 and Figure S2B). In the first subgroup of redox homeostasis, 11 out of 16 identities belong to Class III peroxidases: OsPrx111 (spots 26, 27 and 28), OsPrx112 (spots 29, 30 and 31), OsPrx22 (spot 32), OsPrx125 (spots 33, 34 and 35) and OsPrx71 precursor (spot 36). Class III peroxidases (EC 1.11.1.7), secreted glycoproteins encoded by a large number of paralogous genes, are key enzymes for regulating the H_2_O_2_ level in the apoplast [54]. In this study, all of the peroxidases identified were acidic, suggesting they might participate in the consumption of H_2_O_2_ [55], [56]. The comparative proteomic analysis and enzymatic assays showed that both the abundance and activity of almost all these isoenzymes were down-regulated by H_2_O_2_ especially at the lower H_2_O_2_ concentration (300 µM) except spots 26 and 35 which were more than two-fold up-regulated in abundance (Table S1, Figure S1B and Figure S1B), indicating the different functional assignments for each individual peroxidase in rice seedlings under stress conditions, although little is known concerning the function of individual peroxidase in plants. Besides, it should be noted that malate dehydrogenase (MDH, spots 37 and 38), a protein related to H_2_O_2_-producing was down-regulated, which might decrease H_2_O_2_ production by supplying less NADH to avoid unnecessary H_2_O_2_ production [13], [57] (Figure S1B) under H_2_O_2_ treatments. Moreover, proteins participating in the thiol redox were regulated in the root apoplast of rice seedlings treated with low-dose H_2_O_2_, of which the flavin-containing monooxygenase (FMO, spot 40) was markedly down-regulated, while protein disulfide-isomerase (PDI, spot 41) was up-regulated at 600 µM H_2_O_2_ concentration (Table S1 and Figure S2B). FMO (EC 1.14.13.8) is involved in the formation of protein disulfide bonds, and its suppressed expression might result in an accumulation of reduced proteins and sulfide compounds to maintain the reducing potential [58]. PDI could function to remove the abnormal disulfide bonds induced under oxidative conditions and forms part of the antioxidative defense system [59].

In this study, we also found four differentially expressed protein spots related to signal transduction including three isoforms of OsRMC protein with the DUF26 motif (C-X8-C-X2-C, a cysteine-rich-repeat motif) and an Arm repeat protein in response to H_2_O_2_ treatments. DUF26 motif-containing proteins can be classified into two main groups: receptor-like kinases (RLK) and receptor-like proteins (RLP). The former consist of three distinct domains including an extracellular domain containing two DUF26 motifs, a transmembrane domain and an intracellular kinase domain, while the latter only have an extracellular domain [60]. All three isoforms (spots 42, 43 and 44) of the OsRMC protein identified in the present study only contain an extracellular domain with two DUF26 motifs, and lack the transmembrane domain and intracellular kinase domain (Figure S3). Expression of these isoforms was observed to be repressed at 300 µM H_2_O_2_ treatment (Table S1 and Figure S2B). Therefore, they might function as RLP and play a negative role in the RLK signaling pathway as an antagonist similar to other reported plant RLPs to RLKs [61]. Furthermore, another line of evidence has indicated that knocking down the expression of OsRMC in transgenic rice led to improved tolerance to NaCl stress [22], although further work is required to elucidate its regulation mechanism. On the other hand, a partially degraded form of an arm repeat protein (spot 45) was found to be down-regulated under H_2_O_2_ stress (Table S1 and Figure S2B). This protein is proposed to be plasmodesmatal components, interacting with plant receptor kinases and participating in the signal transduction [62]. This is the first time that an Arm repeat protein was identified as an H_2_O_2_-responsive protein in the root apoplast of rice seedlings, although definitive function of this protein would require further investigation.

It is widely recognized that chitinases and pathogenesis-related protein PR-1a play important roles in self-defense against pathogens and can be induced by both biotic and abiotic stresses [63], [64]. Not surprisingly, one up-regulated PR-1a (spot 47) and one up-regulated chitinases (spot 48) were identified in the root apoplast of rice seedlings under oxidative stress. Interestingly, two down-regulated chitinases (spots 49 and 50) were found in this study. The different expression profiles of the three chitinase proteins indicate the complex function of chitinases in response to H_2_O_2_.

### A possible H_2_O_2_-responsive protein network in the root apoplast of rice seedlings

**Figure 6:**
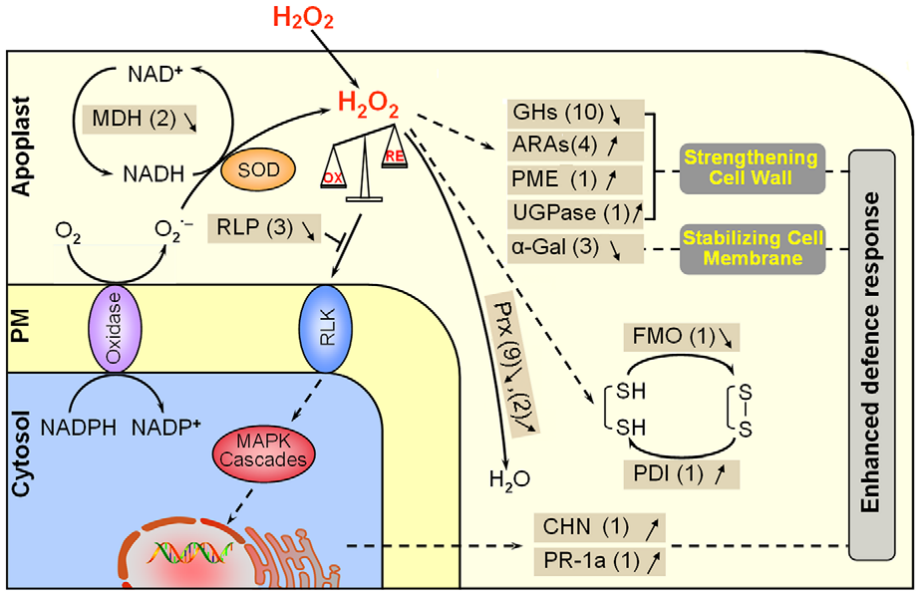
A putative network of H_2_O_2_-responsive apoplast proteins in rice seedling roots. The H_2_O_2_-responsive proteins identified in this study are listed in brown boxes. The up-regulated proteins are marked by↑, and the down-regulated proteins are marked by↓. The number represents the number of protein spots identified. SOD, superoxidase dismutase; FMO, flavin-containing monooxygenases; PDI, protein disulfide-isomerase; Prx, peroxidase; MDH, malate dehydrogenase; GH, glycosylhydrolase; ARAs, proteins with α-L-arabinofuranosidase activity; PME, pectinesterase; UGPase, UDP-glucose pyrophosphorylase; α-GAL, α-galactosidases; RLP, receptor like protein; RLK, receptor like protein kinase; CHN, chitinase; PR-1a, PR-1 type pathogenesis-related protein; S-S, disulfide bond; SH, the reduced formation of S-S.

The proteins present in apoplast are essential constituents of plant cells and have critical roles in modifications of cell wall components, plant defense and signal transduction that allow cells to respond effectively to various extracellular signals, possibly through regulation of H_2_O_2_ levels [8]. However, no systemic investigation into how apoplast proteomes function under oxidative stress through adjustment of the metabolic process and antioxidative system has been conducted to date [8]. In the present work, we initiated a carefully performed proteomic investigation of proteins that are responsive to low-dose H_2_O_2_ in the root apoplast of rice seedlings. Based on our proteomic data, H_2_O_2_-responsive proteins could be divided into two main categories: one group is related to redox regulation including adjusting redox homeostasis, sensing stress cue and thus triggering corresponding reactions; while the other group participates in adjusting metabolism to overcome the deleterious effects of oxidative stress (Table S1, Figure 5 and Figure S2). The results presented in this work are demonstrated in a putative H_2_O_2_-responsive protein network (Figure 6) to address the events that occur in the root apoplast of rice seedlings under oxidative stress condition.

When rice seedlings are exposed to low dose of H_2_O_2_, a large amount of exogenous H_2_O_2_ can easily diffuse into apoplast and thus result in an overall increase of apoplastic H_2_O_2_ level in rice roots (Figure 1C). It is noteworthy that a number of redox-associated enzymes such as MDH and Prx are suppressed at protein levels, which might avoid unnecessary H_2_O_2_ production and modulate the H_2_O_2_ concentration to an appropriate level [13], [57]. Additionally, thiol redox-associated proteins, such as FMO and PDI, are regulated to influence the disulfide bond formation and breakage within proteins, thus activating proteins involved in the antioxidative defense (Figure 6) [58], [59]. Furthermore, down-regulation of the receptor-like protein (RLP) might facilitate transduction of oxidative signals via the receptor-like kinase (RLK). Through a series of signal transduction pathways, finally, defense-related genes such as chitinase and PR-1a could be activated and their products are produced, and then function as a defense factor against oxidative stress in the apoplast of rice roots (Figure 6).

The overall enhanced level of H_2_O_2_ in the root apoplast not only strongly affects the redox homeostasis-related proteins but also acts intensively on carbohydrate metabolism-associated proteins. Down-regulation of almost all GHs, together with up-regulation of UGPase, ARA and PME might strengthen the cell wall through influencing polysaccharide degradation and synthesis, and increasing the pectin demethylesterification (Figure 6). Furthermore, the down-regulation of α-GAL may contribute to stabilize the cell membrane [44], which could reduce the damage to the membrane caused by oxidative stress (Figure 6).

The present work proposed a H_2_O_2_-responsive protein network to elucidate the molecular basis of antioxidative system in the root apoplast of rice seedlings. As H_2_O_2_ could function as a signal molecule in stress perception, we compared our results with other rice apoplast proteome studies under stress conditions [13], [22] to analyze the similarities and differences (Table S5), and found proteins involved in the redox regulation, such as Class III peroxidases and OsRMC, were common in the rice seedling roots under H_2_O_2_ and NaCl treatments [22], and proteins related with antioxidative reactions were both regulated under H_2_O_2_ and dehydration [13], though the specific proteins were different, which might be due to the different tissues studied. However, many proteins were unique to each of the treatments, indicating although the cellular responses to abiotic challenges are similar in regard to production of ROS, the detailed stress response mechanisms might be different. Therefore, a big effort should be made to answer the specificity and cross-talking under different stress conditions.

Overall, by comparative proteomic study of the root apoplast of rice seedlings under low-dose H_2_O_2_ condition, we have identified 54 H_2_O_2_-responsive proteins with obvious functional tendencies towards redox regulation and carbohydrate metabolism. These proteins might work cooperatively to establish a complex network of apoplast response to exogenous H_2_O_2_ in the rice seedling root, and depict the strategies of the root apoplast to oxidative challenge. These findings, in conjunction with previously reported results, significantly advanced our understanding of the molecular basis associated with the oxidative responses occurring in plant apoplast and are also expected to provide a basis for further functional and mechanical research of each identified protein.

## Materials and Methods

### Chemicals

CHAPS, IPG DryStrips, IPG buffers, glucose-6-phosphate (G6P), NADP disodium salts and iodoacetamide were purchased from GE Healthcare (Buckinghamshire, UK); modified trypsin, urea, acrylamide and bis-acrylamide were from Promega (Madison, USA); TCA was from Merck (Darm-stadt, Germany) while thiourea and guaiacol were from Sigma (St. Louis, MO, USA). Deionized water (Millipore, Bedford, MA, USA) with resistance of greater than 18 MΩ cm was used throughout.

### Plant growth and treatment

Rice seeds (*Oryza sativa L. ssp Indica* cv. 93-11) were soaked in distilled water for 24 h and germinated in the dark for 45 h at 37°C. Then the rice seedlings were grown in the biological incubator I-36LL (Percival, IA, USA) at 28/21°C (16-h day/8-h night) with a relative humidity of 70%. To provide whole nutrition to the rice seedlings, Hogland solution was supplied every 2 days. Two-week-old rice seedlings were treated with H_2_O_2_ at different concentrations for 6 h in different plastic containers. Seedlings immersed in double-distilled H_2_O were used as control. Roots were harvested immediately for apoplast protein extraction.

### Measurements of relative electrolyte leakage, malondialdehyde and apoplastic H_2_O_2_ content

The relative electrolyte leakage (REL) assay was performed as previously described by Huang *et al* [65]. with some modifications. Two-week-old rice seedling roots were subjected to test tubes containing 5 ml of an aqueous solution with different H_2_O_2_ concentrations (0–600 µM) at room temperature. The tubes were incubated for 6 h at room temperature with gentle shaking, after which the conductivities of the solutions were determined by a conductivity meter (Model DDS-IIA, Leici Instrument Inc., Shanghai, China). The tubes with roots were then placed in boiling water for 20 min. After cooling down to room temperature, the conductivities of the solutions were measured again. The relative electrolyte leakage was calculated as the percentage of the conductivity before boiling over that after boiling. Three biological replicates for each treatment were made.

MDA content was measured using the thiobarbituric acid (TBA) assay [66]. Two-week-old rice seedlings were treated with or without H_2_O_2_ (300 or 600 µM), then the roots (∼2 g per sample) were harvested and ground on ice with 0.4 g quartz sand, then extracted with 5 ml phosphate buffer (10 mM, pH 7.0). After centrifugation (12 000 *g* for 10 min at 4°C), the supernatant was used to determine the MDA concentration using an MDA detection kit (Jiancheng Bio., Nanjing, China). Three biological replicates for each treatment were made.

H_2_O_2_ accumulated in the apoplast of treated or control roots was measured according to Chang *et al.* [67] with some modifications. The H_2_O_2_ in the apoplast was extracted by the vacuum infiltration-centrifugation method [10] with phosphate buffer (50 mM, pH 6.5). To quantify the H_2_O_2_ content, 750 µL of the extracted solution was mixed with 250 µL of 0.1% titanium sulphate in 20% (v/v) H_2_SO_4_ and then the mixture was centrifugated at 6 000 g for 15 min. Oxidation of titanium sulfate was recorded by reading *A*410. Readings were converted to corresponding concentrations using a standard calibration plot. Three biological replicates for each treatment were made.

### Apoplast protein extraction

To extract the apoplast proteins, the vacuum infiltration-centrifugation method [10] was adopted with some modifications. Roots were cut into approximately 5 cm segments, placed inside tubes, washed with chilled deionized water as rapidly as possible and then submerged into the chilled extraction buffer (100 mM Tris-HCl pH 7.5, 0.2 M KCl, 1 mM PMSF,) in Beckmann centrifuge bottles. The bottles were placed over ice and were subjected to vacuum infiltration at a reduced pressure of 70 kPa for 15 min. The vacuum was then gradually released for 5 min until normal pressure was reached. After the excess extraction buffer was dried under gravity, the infiltrate was collected by centrifugation at 1 000 *g* for 10 min at 4°C. The procedure was repeated and the combined extract was filtered through 0.22 µm membrane filters and concentrated to 500–700 µl using the Microcon YM-5 (Millipore). The concentrate was then subjected to the glucose-6-phosphate dehydrogenase (G6PDH) enzyme assay or TCA precipitation for 2-D electrophoresis. Three biological replicates for each treatment, corresponding to independent protein extracts were performed.

### G6PDH enzyme assay and cytoplasmic contamination calculation

The G6PDH enzyme assay was performed according to the method described by Weimar *et al*. [68] to assess the contamination of the apoplast extracts by cytoplasmic proteins. The following reaction mixtures were prepared: 850 µl reaction buffer (0.1 M Tris-HCl pH 7.6, containing 12.5 mM MgCl_2_), 50 µl glucose-6-phosphate (60 mM), and 50 µl nicotinamide adenine dinucleotide phosphate (NADP) (20 mM). The reaction was started by the addition of 50 µl of the concentrated apoplast protein extract or total root soluble extract, and the change in absorbance at 340 nm was monitored over 5 min at 25°C using a UV/Visible Spectrophotometer (Ultrospec 3300 pro, Amersham Biosciences, Sweden).

To calculate the cytoplasmic contamination, the total soluble proteins were also extracted. Root tissues were frozen in liquid N_2_ and ground to a fine powder with quartz sand. The powder was suspended in extraction buffer (100 mM Tris-HCl pH 7.5, 0.2 M KCl, 5 mM DTT, 1 mM PMSF) and then sonicated 10 times for 10 seconds each time. After centrifugation (16 000 *g* for 30 min at room temperature), the supernatant was used for the G6PDH enzyme assay. Finally, the cytoplasmic contamination was calculated as the percentage of G6PDH activity in the apoplast extracts compared with the activity in the total root soluble protein extracts on a fresh weight basis. Three biological replicates for each treatment were made.

### 2-DE and gel staining

For 2-DE analysis, apoplast proteins were precipitated from the extract by adding equal volume of 20% TCA in acetone containing 0.5% DTT as described by Haslam *et al.* [69] and incubated overnight at −20°C. The precipitates were collected and resolubilized as described by Wan and Liu [3]. First, the precipitates were collected by centrifugation at 23 000 *g* for 30 min at 4°C and subsequent washed three times with acetone and centrifugated at 20 000 *g* for 15 min at 4°C with 30 min incubation at −20°C each time. The pellet was dried with N_2_ to remove any remaining acetone. The dried powder was resuspended completely in 300 µl lysis buffer (9 M urea, 4% [w/v] CHAPS, and 2% ampholytes, pH 3–10). After incubation at 25°C for 0.5 h, the extract was reduced by adding 5 mM Tris-(β-carboxyethyl)-phosphine hydrochloride. The reduction continued for 1 h at room temperature. Samples were then alkylated by treatment with 16 mM iodoacetamide for 1.5 h at room temperature. This reaction was quenched by the addition of 50 mM DTT. The samples were centrifugated at 16 000 *g* for 30 min at 25°C and stored at −80°C for further 2-DE gel analysis. The protein concentration was estimated by Bradford method [70]. Prior to IEF, the samples were diluted with the rehydration buffer (6 M urea, 2 M thiourea, 2% CHAPS, and 40 mM DTT) so as to load 200 µg proteins per 24 cm linear pH 4–7 IPG strip. The strips were subjected to active rehydration at 30 V/h for 18 h on the Ettan IPGphor system (GE Healthcare) with the following IEF program: 200 V for 40 min, 500 V for 40 min, 1000 V for 1 h, 4000 V for 2 h, 8000 V gradient for 1 h, and 8000 V for around 7.5 h until a total voltage hour of 75000 was achieved. Before the SDS-PAGE, the strips were equilibrated for 15 min in 10 ml of reducing equilibration buffer (6 M urea, 50 mM Tris-HCl with pH 8.8, 30% [v/v] glycerol, 2% [w/v] SDS, a trace of bromophenol blue and 1% [w/v] DTT) and for another 15 min in alkylating equilibration buffer that contained 2.5% (w/v) iodoacetamide instead of 1% DTT. The strips were placed on the top of vertical 12.5% SDS-PAGE selfcast gels. The electrophoresis was carried out at 25°C and 2.5 W/gel for 30 min, and then at 17 W/gel until the dye front reached about 1 mm from the bottom of the gel using an EttanTM DALT System (GE Healthcare). For each treatment, three gel replicates were run with three biological replicates. Protein spots were visualized by the mass spectrometry compatible silver staining method [11].

### Gel scanning and image analysis

The 2-DE gels were scanned with a UMAX PowerLook 2100XL scanner (Willich, Germany) using LabScan 5.0 software. As triplicates were applied to each treatment, a total of 9 silver-stained 2-DE gels were analyzed using the ImageMaster^TM^ 2-D platinum software version 5.0 (GE Healthcare). Only the spots present in all the three replicate gels and qualitatively consistent in size and shape in the replicate gels were considered. Matching spots were rechecked manually. The spot volume was taken as a percentage relative to the total volume of all spots in the gel. A criterion of *p*<0.05 was used to define the significant difference when analyzing the parallel spots between groups. One-way ANOVA and the Student-Newman-Keuls test was carried out using the SAS software package version 8.2 (SAS Institute).

### Protein identification

Protein spots showing significant changes in abundance during the treatments were selected and excised manually for protein identification. In- gel digestion of protein spots was performed according to Wan and Liu [3], except that gel pieces were first destained with a solution of 15 mM potassium ferricyanide and 50 mM sodium thiosulfate (1∶1) instead of 25 mM ammonium bicarbonate with 50% ACN. After that, the particles were washed twice in 100% ACN for 10 min and then dried under vacuum for 15 min. Proteins in the gel pieces were digested in 25 mM NH_4_HCO_3_, 10 ng/mL trypsin over night at 37°C. To recover the peptides, four volumes of 5 mM n-OGP in 0.25% TFA were added, and incubation was performed for 1 h at 37°C. The extraction solutions were used in the subsequent mass analysis. All MALDI-TOF/TOF information was obtained from an ABI 4800 MALDI TOF/TOF^TM^ Analyzer (Applied Biosystems, CA, USA). The TOF spectra were recorded in the positive ion reflector mode with a mass range from 700 to 3500 Da, and 5 of the strongest peaks of the TOF spectra per sample were chosen for MS/MS analysis. The spectra were corrected by an external standard method using trypsin-treated myoglobin peptides. The MS/MS results were searched using GPS (Applied Biosystems, USA) - MASCOT (Matrix Science, London, UK) with the following criteria: NCBInr database; species restriction to *Oryza sativa* (rice); MS tolerance was set at ±100 ppm and MS/MS at ±0.6 Da; at most one missed cleavage site; fixed modification was carbamidomethyl (Cys) and variable modification was oxidation (Met); and cleavage by trypsin was the C-terminal side of Lys and Arg unless the next residue was Pro. If peptides were matched to multiple members of a protein family, or if a protein appeared under different names and accession numbers, the entry with the highest score was selected. In addition, the theoretical molecular weights and p*I* of the identified proteins were calculated using the PeptideMass program (http://au.expasy.org/tools/peptide-mass.html).

### Location prediction

The subcellular location of the identified proteins were predicted by the TargetP program (www.cbs.dtu.dk/services/TargetP) [25]. For those proteins without typical signal peptide sequences, SecretomeP software program (http://www.cbs.dtu.dk/services/SecretomeP-1.0) [33] was performed to inspect the non-classical secretory proteins.

### Peroxidase enzyme assay

The peroxidase enzyme assay was performed according to Fecht-Christoffers *et al.* [56]. To measure the H_2_O_2_-consuming peroxidase (acidic) activity in the VI (vacuum infiltrates), the apoplast protein extract was mixed with 10 mM Na_2_HPO_4_ buffer (pH 6.0), 20 mM guaiacol and 0.03% (w/w) H_2_O_2_. Then the activity was measured at λ = 470 nm. For the enzyme activity calculation, the molar extinction coefficient of 26.6 mM^−1^cm^−1^ for tetraguaiacol was used. Three biological replicates for each treatment were made.

## Supporting Information

Figure S1 Proteins identified related with carbohydrate metabolism and redox homeostasis in response to H_2_O_2_.
(DOCX)

Figure S2 Hierarchical clustering of H_2_O_2_-responsive proteins associated with carbohydrate metabolism (A) and redox regulation (B).
(DOCX)

Figure S3 Schematic structure of the At-RLK3 protein and the RLP protein we identified (OsRMC).
(DOCX)

Table S1 Differentially expressed protein spots identified by PMF or MS/MS.
(DOCX)

Table S2 Peptide tags of proteins identified by MS/MS.
(DOCX)

Table S3 Redundancy of differentially displayed protein spots identified by PMF or MS/MS.
(XLS)

Table S4 Localization prediction of proteins without signal sequences by the SecretomeP software.
(DOCX)

Table S5 List of common proteins in the rice apoplast proteome under different treatments.
(DOCX)

File S1 **Supplemental spectra PMF.** Annotated spectra for 33 differentially expressed protein spots identified by PMF.
(PPT)

File S2 **Supplemental spectra MS/MS.** Annotated spectra of 21 differentially expressed protein spots identified by MS/MS.
(PPT)

## References

[1] Mittler R, Vanderauwera S, Gollery M, Van Breusegem F (2004). Reactive oxygen gene network of plants.. Trends Plant Sci.

[2] Slesak I, Libik M, Karpinska B, Karpinski S, Miszalski Z (2007). The role of hydrogen peroxide in regulation of plant metabolism and cellular signalling in response to environmental stresses.. Acta Biochim Pol.

[3] Wan XY, Liu JY (2008). Comparative proteomics analysis reveals an intimate protein network provoked by hydrogen peroxide stress in rice seedling leaves.. Mol Cell Proteomics.

[4] Desikan R, Mackerness SAH, Hancock JT, Neill SJ (2001). Regulation of the Arabidopsis transcriptome by oxidative stress.. Plant Physiol.

[5] Vanderauwera S, Zimmermann P, Rombauts S, Vandenabeele S, Langebartels C (2005). Genome-wide analysis of hydrogen peroxide-regulated gene expression in Arabidopsis reveals a high light-induced transcriptional cluster involved in anthocyanin biosynthesis.. Plant Physiol.

[6] Vandenabeele S, Van Der Kelen K, Dat J, Gadjev I, Boonefaes T (2003). A comprehensive analysis of hydrogen peroxide-induced gene expression in tobacco.. Proc Natl Acad Sci U S A.

[7] Lee SJ, Saravanan RS, Damasceno CMB, Yamane H, Kim BD (2004). Digging deeper into the plant cell wall proteome.. Plant Physiol Biochem.

[8] Jamet E, Canut H, Boudart G, Pont-Lezica RF (2006). Cell wall proteins: a new insight through proteomics.. Trends Plant Sci.

[9] Jamet E, Albenne C, Boudart G, Irshad M, Canut H (2008). Recent advances in plant cell wall proteomics.. Proteomics.

[10] Dani V, Simon WJ, Duranti M, Croy RRD (2005). Changes in the tobacco leaf apoplast proteome in response to salt stress.. Proteomics.

[11] Soares NC, Francisco R, Vielba JM, Ricardo CP, Jackson PA (2009). Associating wound-related changes in the apoplast proteome of Medicago with early steps in the ROS signal transduction pathway.. J Proteome Res.

[12] Bhushan D, Pandey A, Choudhary MK, Datta A, Chakraborty S (2007). Comparative proteomics analysis of differentially expressed proteins in chickpea extracellular matrix during dehydration stress.. Mol Cell Proteomics.

[13] Pandey A, Rajamani U, Verma J, Subba P, Chakraborty N (2010). Identification ofextracellular matrix proteins of rice (Oryza sativa L.) involved in dehydration-responsive network: a proteomic approach.. J Proteome Res.

[14] Floerl S, Druebert C, Majcherczyk A, Karlovsky P, Kues U (2008). Defence reactions in the apoplastic proteome of oilseed rape (Brassica napus var. napus) attenuate Verticillium longisporum growth but not disease symptoms.. BMC Plant Biol.

[15] Goulet C, Goulet MC, Michaud D (2010). 2-DE proteome maps for the leaf apoplast of Nicotiana benthamiana.. Proteomics.

[16] Fecht-Christoffers MM, Braun HP, Lemaitre-Guillier C, VanDorsselaer A, Horst WJ (2003). Effect of Manganese toxicity on the proteome of the leaf apoplast in cowpea.. Plant Physiol.

[17] Alves M, Francisco R, Martins I, Ricardo CPP (2006). Analysis of Lupinus albus leaf apoplastic proteins in response to boron deficiency.. Plant Soil.

[18] Wang FZ, Wang QB, Kwon SY, Kwak SS, Su WA (2005). Enhanced drought tolerance of transgenic rice plants expressing a pea manganese superoxide dismutase.. J Plant Physiol.

[19] Lee TM, Lur HS, Chu C (1997). Role of abscisic acid in chilling tolerance of rice (Oryza sativa L) seedlings.II. Modulation of free polyamine levels.. Plant Sci.

[20] Zhang JX, Kirkham MB (1996). Antioxidant responses to drought in sunflower and sorghum seedlings.. New Phytol.

[21] Vanacker H, Carver TLW, Foyer CH (1998). Pathogen-induced changes in the antioxidant status of the apoplast in barley leaves.. Plant Physiol.

[22] Zhang L, Tian LH, Zhao JF, Song Y, Zhang CJ (2009). Identification of an apoplastic protein involved in the initial phase of salt stress response in rice root by two-dimensional electrophoresis.. Plant Physiol.

[23] Yan SP, Zhang QY, Tang ZC, Su WA, Sun WN (2006). Comparative proteomic analysis provides new insights into chilling stress responses in rice.. Mol Cell Proteomics.

[24] Weeks ME, Sinclair J, Butt A, Chung YL, Worthington JL (2006). A parallel proteomic and metabolomic analysis of the hydrogen peroxide- and Sty1p-dependent stress response in Schizosaccharomyces pombe.. Proteomics.

[25] Emanuelsson O, Nielsen H, Brunak S, von Heijne G (2000). Predicting subcellular localization of proteins based on their N-terminal amino acid sequence.. J Mol Biol.

[26] Slabas AR, Ndimba B, Simon WJ, Chivasa S (2004). Proteomic analysis of the Arabidopsis cell wall reveals unexpected proteins with new cellular locations.. Biochem Soc Trans.

[27] Chivasa S, Ndimba BK, Simon WJ, Robertson D, Yu XL (2002). Proteomic analysis of the Arabidopsis thaliana cell wall.. Electrophoresis.

[28] Watson BS, Lei ZT, Dixon RA, Sumner LW (2004). Proteomics of Medicago sativa cell walls.. Phytochemistry.

[29] Natera SHA, Ford KL, Cassin AM, Patterson JH, Newbigin EJ (2008). Analysis of the Oryza sativa plasma membrane proteome using combined protein and peptide fractionation approaches in conjunction with mass spectrometry.. J Proteome Res.

[30] Pitarch A, Sanchez M, Nombela C, Gil C (2002). Sequential fractionation and two-dimensional gel analysis unravels the complexity of the dimorphic fungus Candida albicans cell wall proteome.. Mol Cell Proteomics.

[31] Copley SD (2003). Enzymes with extra talents: moonlighting functions and catalytic promiscuity.. Curr Opin Chem Biol.

[32] Jeffery CJ (2005). Mass spectrometry and the search for moonlighting proteins.. Mass Spectrom Rev.

[33] Bendtsen JD, Jensen LJ, Blom N, von Heijne G, Brunak S (2004). Feature-based prediction of non-classical and leaderless protein secretion.. Protein Eng Des Sel.

[34] Gross GG (1977). Cell wall bound malate-dehydrogenase from horseradish.. Phytochemistry.

[35] Mader M, Schloss P (1979). Isolation of malate dehydrogenase from cell walls of nicotiana tabacum.. Plant Sci Lett.

[36] Edwards SR, Braley R, Chaffin WL (1999). Enolase is present in the cell wall of Saccharomyces cerevisiae.. FEMS.

[37] Motshwene P, Brandt W, Lindsey G (2003). Significant quantities of the glycolytic enzyme, phosphoglycerate mutase are present in the cell wall of yeast Saccharomyces cerevisiae.. Biochem J.

[38] Negri AS, Prinsi B, Scienza A, Morgutti S, Cocucci M (2008). Analysis of grape berry cell wall proteome: A comparative evaluation of extraction methods.. J Plant Physiol.

[39] Kleczkowski LA, Geisler M, Ciereszko I, Johansson H (2004). UDP-Glucose pyrophosphorylase. An old protein with new tricks.. Plant Physiol.

[40] Sagi G, Katz A, Guenoune-Gelbart D, Epel BL (2005). Class 1 reversibly glycosylated polypeptides are plasmodesmal-associated proteins delivered to plasmodesmata via the Golgi apparatus.. Plant Cell.

[41] Zhu JM, Alvarez S, Marsh EL, LeNoble ME, Cho IJ (2007). Cell wall Proteome in the maize primary root elongation zone. II. Region-specific changes in water soluble and lightly ionically bound proteins under water deficit.. Plant Physiol.

[42] Minic Z (2008). Physiological roles of plant glycoside hydrolases.. Planta.

[43] Hoson T (1998). Apoplast as the site of response to environmental signals.. J Plant Res.

[44] Taji T, Ohsumi C, Iuchi S, Seki M, Kasuga M (2002). Important roles of drought- and cold-inducible genes for galactinol synthase in stress tolerance in Arabidopsis thaliana.. Plant J.

[45] Nishizawa A, Yabuta Y, Shigeoka S (2008). Galactinol and raffinose constitute a novel function to protect plants from oxidative damage.. Plant Physiol.

[46] Pennycooke JC, Jones ML, Stushnoff C (2003). Down-regulating alpha-galactosidase enhances freezing tolerance in transgenic petunia.. Plant Physiol.

[47] Showalter AM (2001). Arabinogalactan-proteins: structure, expression and function.. Cell Mol Life Sci.

[48] Kotake T, Tsuchiya K, Aohara T, Konishi T, Kaneko S (2006). An alpha-L-arabinofuranosidase/beta-D-xylosidase from immature seeds of radish (Raphanus sativus L.).. J Exp Bot.

[49] Chen RZ, Zhao X, Shao Z, Wei Z, Wang YY (2007). Rice UDP-glucose pyrophosphorylase 1 is essential for pollen callose deposition and its cosuppression results in a new type of thermosensitive genic male sterility.. Plant Cell.

[50] Pacoda D, Montefusco A, Piro G, Dalessandro G (2004). Reactive oxygen species and nitric oxide affect cell wall metabolism in tobacco BY-2 cells.. J Plant Physiol.

[51] Godon C, Lagniel G, Lee J, Buhler JM, Kieffer S (1998). The H_2_O_2_ stimulon in Saccharomyces cerevisiae.. J Biol Chem.

[52] Pelloux J, Rusterucci C, Mellerowicz EJ (2007). New insights into pectin methylesterase structure and function.. Trends Plant Sci.

[53] Micheli F (2001). Pectin methylesterases: cell wall enzymes with important roles in plant physiology.. Trends Plant Sci.

[54] Passardi F, Cosio C, Penel C, Dunand C (2005). Peroxidases have more functions than a Swissarmy knife.. Plant Cell Rep.

[55] Passardi F, Longet D, Penel C, Dunand C (2004). The class III peroxidase multigenic in land plants family in rice and its evolution.. Phytochemistry.

[56] Fecht-Christoffers MM, Fuhrs H, Braun HP, Horst WJ (2006). The role of hydrogen peroxide-producing and hydrogen peroxide-consuming peroxidases in the leaf apoplast of cowpea in manganese tolerance.. Plant Physiol.

[57] Gross GG, Janse C, Elstner EF (1977). Involvement of malate, monophenols, and superoxide radical in hydrogen-peroxide formation by isolated cell-walls from Horseradish (Armoracia-Lapathifolia-Gilib).. Planta.

[58] Schlaich NL (2007). Flavin-containing monooxygenases in plants: looking beyond detox.. Trends Plant Sci.

[59] Sweetlove LJ, Heazlewood JL, Herald V, Holtzapffel R, Day DA (2002). The impact of oxidative stress on Arabidopsis mitochondria.. Plant J.

[60] Chen ZX (2001). A superfamily of proteins with novel cysteine-rich repeats.. Plant Physiol.

[61] Shiu SH, Bleecker AB (2001). Plant receptor-like kinase gene family: diversity, function, and signaling.. Sci STKE.

[62] Samuel MA, Salt JN, Shiu SH, Goring DR (2006). Multifunctional arm repeat domains in plants.. Int Rev Cytol.

[63] Green R, Fluhr R (1995). Uv-B-Induced Pr-1 accumulation is mediated by active oxygen species.. Plant Cell.

[64] Sasaki C, Varum KM, Itoh Y, Tamoi M, Fukamizo T (2006). Rice chitinases: sugar recognition specificities of the individual subsites.. Glycobiology.

[65] Huang J, Sun SJ, Xu DQ, Yang X, Bao YM (2009). Increased tolerance of rice to cold, drought and oxidative stresses mediated by the overexpression of a gene that encodes the zinc finger protein ZFP245.. Biochem Biophys Res Commun.

[66] Li T, Liu GL, Duan MX, Liu JY (2009). Radish phospholipid hydroperoxide glutathione peroxidase provides protection against hydroperoxide-mediated injury in mouse 3T3 fibroblasts.. BMB Rep.

[67] Chang CJ, Kao CH (1998). H_2_O_2_ metabolism during senescence of rice leaves: changes in enzyme activities in light and darkness.. Plant Growth Regul.

[68] Weimar M, Rothe GM (1987). Preparation of extracts from mature spruce needles for enzymatic analyses.. Physiol Plant.

[69] Haslam RP, Downie AL, Raveton M, Gallardo K, Job D (2003). The assessment of enriched apoplastic extracts using proteomic approaches.. Ann Appl Biol.

[70] Yao Y, Yang YW, Liu JY (2006). An efficient protein preparation for proteomic analysis of developing cotton fibers by 2-DE.. Electrophoresis.

