# Supplementary material for: Comparative Proteomics Analysis of the Root Apoplasts of Rice Seedlings in Response to Hydrogen Peroxide
Source: PLoS One. 2011 Feb 10;6(2):e16723. doi: 10.1371/journal.pone.0016723 (PMC3037377; doi:10.1371/journal.pone.0016723)
Supplement: Figure S1 — Proteins identified related with carbohydrate metabolism and redox homeostasis in response to H2O2. (DOCX) [file pone.0016723.s001.docx]

**
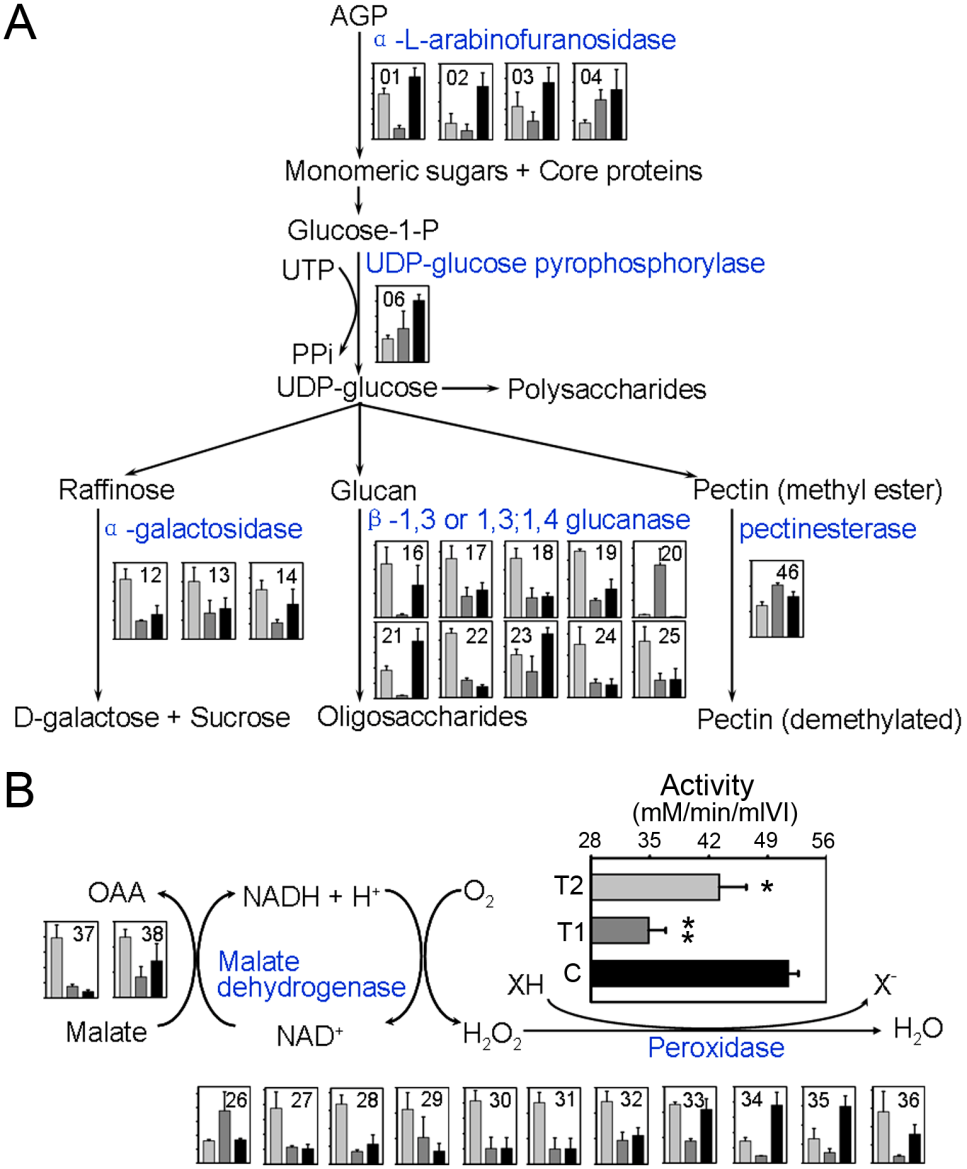
**

**Figure S1. Proteins identified related with carbohydrate metabolism and redox homeostasis in response to H_2_O_2._** (A) Pathways involved in carbohydrate metabolism that respond to the H_2_O_2_ treatments. (B) Expression profile of malate dehydrogenase (MDH) and peroxidase and peroxidase activity. XH represents substrates that can be oxidized to X**^·^** form. OAA, oxaloacetate; VI, vacuum infiltrates. T1 and T2 represent H_2_O_2_ treatments of 300 µM and 600 µM, respectively. Values are means of independent replicates±SE, n=3. Levels of significance of *T*-test are shown by * and ** for *p*<0.05 and 0.01, compared to the control.
