# Supplementary material for: Comparative Proteomics Analysis of the Root Apoplasts of Rice Seedlings in Response to Hydrogen Peroxide
Source: PLoS One. 2011 Feb 10;6(2):e16723. doi: 10.1371/journal.pone.0016723 (PMC3037377; doi:10.1371/journal.pone.0016723)
Supplement: Figure S2 — Hierarchical clustering of H2O2-responsive proteins associated with carbohydrate metabolism (A) and redox regulation (B). (DOCX) [file pone.0016723.s002.docx]

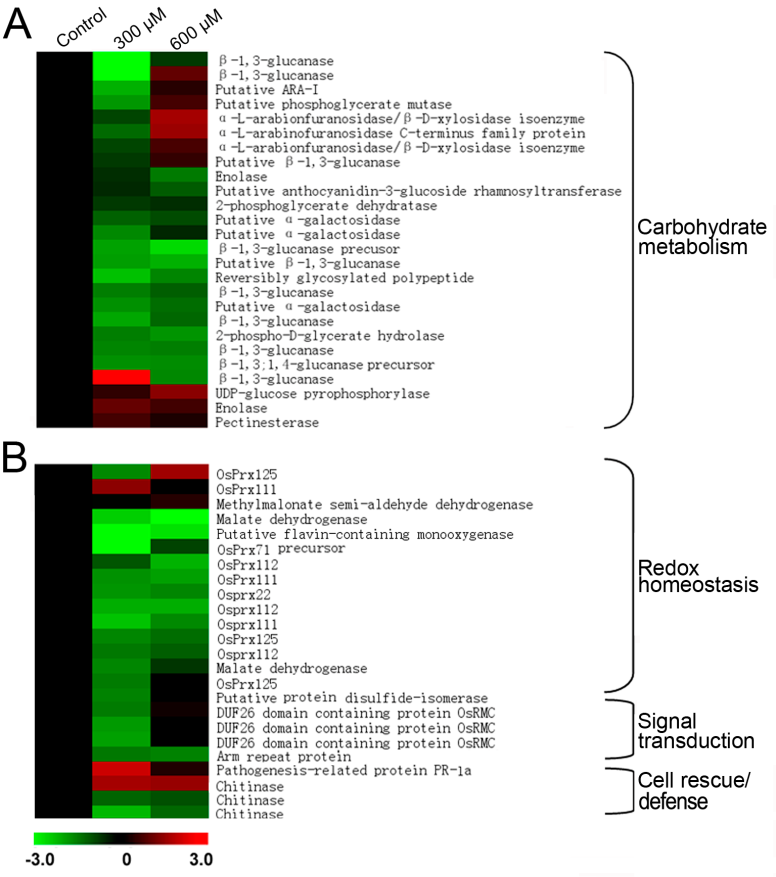


**Figure S2. Hierarchical clustering of H_2_O_2_-responsive proteins associated with carbohydrate metabolism (A) and redox regulation (B).** The hierarchical cluster analysis was conducted using the MultiExperiment Viewer (MEV) software.
