# Supplementary material for: Comparative Proteomics Analysis of the Root Apoplasts of Rice Seedlings in Response to Hydrogen Peroxide
Source: PLoS One. 2011 Feb 10;6(2):e16723. doi: 10.1371/journal.pone.0016723 (PMC3037377; doi:10.1371/journal.pone.0016723)
Supplement: Figure S3 — Schematic structure of the At-RLK3 protein and the RLP protein we identified (OsRMC). (DOCX) [file pone.0016723.s003.docx]

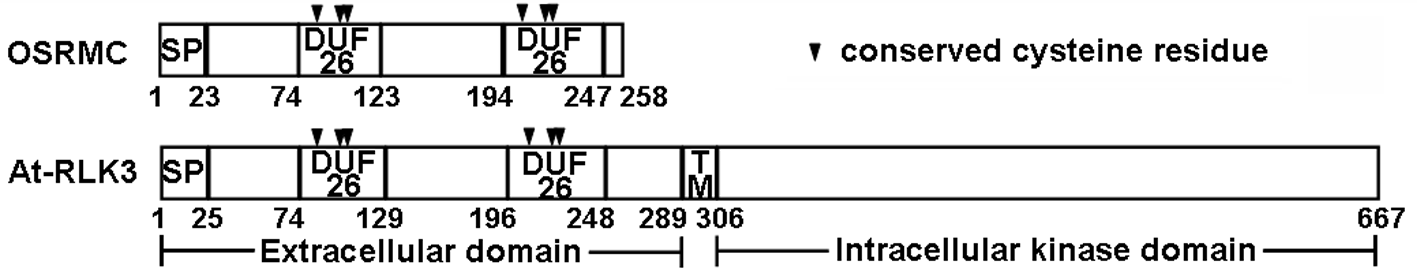


**Figure S3. Schematic structure of the At-RLK3 protein and the RLP protein we identified (OsRMC).** The signal peptide (SP), DUF26 motif, extracellular domain, transmembrane domain (TM) and the intracellular kinase domain are indicated.
