# Supplementary material for: Comparative Proteomics Analysis of the Root Apoplasts of Rice Seedlings in Response to Hydrogen Peroxide
Source: PLoS One. 2011 Feb 10;6(2):e16723. doi: 10.1371/journal.pone.0016723 (PMC3037377; doi:10.1371/journal.pone.0016723)
Supplement: Table S1 — Differentially expressed protein spots identified by PMF or MS/MS. (DOCX) [file pone.0016723.s004.docx]

**Table S1.** Differentially expressed protein spots identified by PMF or MS/MS

| **Spot No.** | **NCBI accession no.** | **Protein name** | **Theoretical** | | |  | **Observed** | |  | **Average fold change*^a^*** | | **Score** | **M*^b^*** | **C(%)*^c^*** | **Loc*^d^*** |
| --- | --- | --- | --- | --- | --- | --- | --- | --- | --- | --- | --- | --- | --- | --- | --- |
|  |  |  | ***Mr*** | | **p*I*** |  | ***Mr*** | **p*I*** |  | **T1** | **T2** |  |  |  |  |
| **Carbohydrate metabolism** | | | | | | | | | | | | | | | |
| 01 | BAB55751 | Putative α-L-arabinofuranosidase/β-D-xylosidase isoenzyme ARA-I | 87129 | | 5.21 | 95060 | | 5.17 | -4.16 | | 1.38 | 68 | 11 | 26 | SP |
| 02 | NP_001054038 | α-L-arabinofuranosidase/β-D-xylosidase isoenzyme ARA-I | 80921 | | 6.42 | 76460 | | 5.81 | ns*^e^* | | 3.21 | 171 | 22 | 40 | SP |
| 03 | NP_001054038 | α-L-arabinofuranosidase/β-D-xylosidase isoenzyme ARA-I | 80921 | | 6.42 | 74992 | | 6.01 | ns | | 1.71 | 140 | 18 | 37 | SP |
| 04 | NP_001066062 | α-L-arabinofuranosidase C-terminus family protein | 72878 | | 5.72 | 72959 | | 5.45 | 2.39 | | 3.01 | 108 | 15 | 42 | SP |
| 05 | NP_001044625 | Putative phosphoglycerate mutase | 60790 | | 5.42 | 68669 | | 5.43 | -3.43 | | ns | 105 | 16 | 42 | R |
| 06 | NP_001063879 | UDP-glucose pyrophosphorylase | 51682 | | 5.43 | 63083 | | 5.79 | ns | | 2.65 | 201 | 22 | 62 | R |
| 07 | Q42971 | Enolase | 47972 | | 5.41 | 59522 | | 5.64 | ns | | -2.72 | 250 | 24 | 73 | R |
| 08 | Q42971 | Enolase | 47972 | | 5.41 | 62474 | | 5.65 | 2.08 | | 1.63 | 107 | 2* | 10 | R |
| 09 | AAC49173 | 2-phospho-D-glycerate hydrolase | 47986 | | 5.42 | 58297 | | 5.54 | -2.78 | | -3.35 | 141 | 2* | 22 | R |
| 10 | NP_001064223 | 2-phosphoglycerate dehydratase | 47914 | | 5.50 | 58954 | | 5.59 | -1.63 | | ns | 224 | 23 | 60 | R |
| 11 | NP_001064066 | Putative anthocyanidin-3-  glucoside rhamnosyltransferase | 53426 | | 6.32 | 48872 | | 5.68 | ns | | -2.15 | 61 | 13 | 21 | SP |
| 12 | NP_001060639 | Putative α-galactosidase | 47049 | | 5.93 | 47913 | | 5.49 | -3.27 | | -2.41 | 158 | 18 | 62 | SP |
| 13 | NP_001060639 | Putative α-galactosidase | 47049 | | 5.93 | 47112 | | 5.70 | -2.21 | | ns | 136 | 14 | 54 | SP |
| 14 | NP_001060639 | Putative α-galactosidase | 47049 | | 5.93 | 43648 | | 5.67 | -3.02 | | ns | 136 | 3* | 17 | SP |
| 15 | CAA77235 | Reversibly glycosylated polypeptide | 41375 | | 5.83 | 45730 | | 5.80 | -4.86 | | -2.91 | 104 | 13 | 55 | R |
| 16 | BAA77785 | β-1,3-glucanase | 35607 | | 7.02 | 33882 | | 5.15 | -21.55 | | -1.65 | 66 | 9 | 44 | SP |
| 17 | BAA77785 | β-1,3-glucanase | 35607 | | 7.02 | 33110 | | 5.64 | -2.78 | | -2.16 | 108 | 12 | 53 | SP |
| 18 | BAA77785 | β-1,3-glucanase | 35607 | | 7.02 | 32824 | | 5.59 | -2.98 | | -2.79 | 79 | 10 | 51 | SP |
| 19 | BAA77785 | β-1,3-glucanase | 35607 | | 7.02 | 35187 | | 5.28 | -3.89 | | -2.32 | 98 | 2* | 27 | SP |
| 20 | BAA77785 | β-1,3-glucanase | 35607 | | 7.02 | 34371 | | 5.28 | 10.17 | | -3.01 | 192 | 3* | 25 | SP |
| 21 | BAA77785 | β-1,3-glucanase | 35607 | | 7.02 | 32696 | | 5.28 | -9.21 | | 2.03 | 95 | 2* | 23 | SP |
| 22 | NP_001044052 | β-1,3-glucanase precursor | 35810 | | 6.83 | 33545 | | 5.28 | -3.7 | | -5.96 | 133 | 2* | 16 | SP |
| 23 | NP_001059883 | Putative β-1,3-glucanase | 58545 | | 4.98 | 30314 | | 5.73 | ns | | 1.48 | 97 | 2* | 4 | SP |
| 24 | NP_001059883 | Putative β-1,3-glucanase | 58545 | | 4.98 | 29542 | | 5.42 | -3.61 | | -4.19 | 62 | 1* | 6 | SP |
| 25 | NP_001055377 | β-1,3;1,4-glucanase precursor | 34706 | | 5.92 | 30078 | | 5.57 | -3.23 | | -3.12 | 145 | 2* | 23 | SP |
| **Redox homeostasis** | | | | | | | | | | | | | | | |
| 26 | CAA46916 | Peroxidase OsPrx111 | | 32876 | 5.77 | 34192 | | 5.11 | 2.35 | | ns | 76 | 9 | 47 | SP |
| 27 | CAA46916 | Peroxidase OsPrx111 | | 32876 | 5.77 | 33298 | | 5.42 | -3.29 | | -3.62 | 67 | 8 | 44 | SP |
| 28 | CAA46916 | Peroxidase OsPrx111 | | 32876 | 5.77 | 33038 | | 5.45 | -4.8 | | -3.02 | 112 | 2* | 31 | SP |
| 29 | NP_001060629 | Peroxidase OsPrx112 | | 32606 | 5.51 | 36242 | | 5.22 | ns | | -4.2 | 95 | 10 | 60 | SP |
| 30 | NP_001060629 | Peroxidase OsPrx112 | | 32606 | 5.51 | 37297 | | 5.79 | -4.04 | | -4.02 | 250 | 3* | 25 | SP |
| 31 | NP_001060629 | Peroxidase OsPrx112 | | 32606 | 5.51 | 36942 | | 5.80 | -2.62 | | -2.18 | 244 | 3* | 25 | SP |
| 32 | NP_001045483 | Peroxidase OsPrx22 | | 38047 | 4.99 | 37394 | | 5.25 | -3.34 | | -2.96 | 105 | 2* | 18 | SP |
| 33 | NP_001064030 | Peroxidase OsPrx125 | | 35700 | 4.72 | 42368 | | 4.77 | -2.67 | | ns | 66 | 9 | 39 | SP |
| 34 | NP_001064030 | Peroxidase OsPrx125 | | 35700 | 4.72 | 42033 | | 4.64 | -3.05 | | ns | 102 | 10 | 58 | SP |
| 35 | NP_001064030 | Peroxidase OsPrx125 | | 35700 | 4.72 | 39602 | | 4.64 | ns | | 2.31 | 115 | 13 | 59 | SP |
| 36 | CAH69313 | Peroxidase OsPrx71 precursor | | 35342 | 4.83 | 46878 | | 4.88 | -7.58 | | ns | 66 | 2* | 25 | SP |
| 37 | NP_001064860 | Malate dehydrogenase | | 35569 | 5.75 | 36718 | | 5.98 | -5.23 | | -9.55 | 126 | 13 | 61 | R |
| 38 | NP_001064860 | Malate dehydrogenase | | 35569 | 5.75 | 35960 | | 5.94 | -2.94 | | -1.65 | 145 | 2* | 39 | R |
| 39 | NP_001059082 | Methylmalonate semi-aldehyde dehydrogenase | | 57247 | 5.99 | 64162 | | 5.80 | ns | | 1.35 | 160 | 19 | 53 | S |
| 40 | NP_001058740 | Putative flavin-containing monooxygenase | | 53047 | 5.52 | 38067 | | 5.74 | -7.58 | | -6.25 | 66 | 10 | 15 | — |
| 41 | NP_001067436 | Putative protein disulfide isomerase | | 56855 | 5.01 | 71212 | | 4.86 | -2.89 | | 1.11 | 146 | 4* | 15 | SP |
| **Signal transduction** | | | | | | | | | | | | | | | |
| 42 | NP_001054135 | DUF26 motif containing protein OsRMC | 27306 | | 5.01 | 56057 | | 4.77 | -2.69 | | ns | 121 | 13 | 61 | SP |
| 43 | NP_001054135 | DUF26 motif containing protein OsRMC | 27306 | | 5.01 | 55433 | | 4.68 | -3.49 | | ns | 77 | 10 | 36 | SP |
| 44 | NP_001054135 | DUF26 motif containing protein OsRMC | 27306 | | 5.01 | 35248 | | 4.57 | -3.54 | | ns | 92 | 10 | 63 | SP |
| 45 | ABB47908 | Arm repeat protein | 58748 | | 8.78 | 30236 | | 5.70 | -2.6 | | -2.89 | 62 | 15 | 24 | R |
| **Cell wall modification** | | | | | | | | | | | | | | | |
| 46 | EAY76707 | Pectinesterase | 57903 | | 6.14 | 67024 | | 5.98 | 1.65 | | 1.29 | 168 | 17 | 52 | S |
| **Cell rescue/defense** | | | | | | | | | | | | | | | |
| 47 | NP_001058815 | Pathogenesis-related protein PR-1a | 17534 | | 4.55 | 19004 | | 4.18 | 3.44 | | ns | 78 | 5 | 68 | SP |
| 48 | EAY79390 | Chitinase | 27536 | | 6.09 | 27826 | | 4.87 | ns | | 2.48 | 87 | 9 | 60 | SP |
| 49 | AAO15366 | Chitinase | 31199 | | 4.94 | 33720 | | 5.25 | -2.26 | | -1.95 | 63 | 1* | 9 | S |
| 50 | AAC37516 | Chitinase | 18968 | | 5.01 | 29305 | | 5.06 | -4.44 | | -2.3 | 85 | 1* | 10 | S |
| **Nucleotide metabolism** | | | | | | | | | | | | | | | |
| 51 | NP_001047479 | Putative adenosine kinase | 36993 | | 5.07 | 42033 | | 5.02 | -1.6 | | -1.93 | 70 | 8 | 39 | R |
| 52 | NP_001042295 | Putative nucleotide diphosphatase | 52198 | | 5.11 | 56417 | | 4.86 | -1.67 | | ns | 150 | 3* | 17 | S |
| **Protein degredation** | | | | | | | | | | | | | | | |
| 53 | NP_001047954 | Peptidase A1 | 47852 | | 5.55 | 43003 | | 4.90 | -1.96 | | 1.85 | 136 | 2* | 9 | SP |
| **Unknown Proteins** | | | | | | | | | | | | | | | |
| 54 | BAD29182 | Putative uncharacterized protein P0453B09.39 | 19363 | | 8.87 | 33588 | | 5.33 | -1.81 | | -2.7 | 71 | 8 | 59 | S |

*^a^* Spot abundance is calculated as the intensity of the up-regulated (plus value) or down-regulated (minus value) protein over the intensity of the control. -Fold changes had *p* values<0.05. T1 and T2 represent 300 µM or 600 µM H_2_O_2_ treatment concentrations, respectively.

*^b^* Number of matched mass values.

*^c^* Sequence coverage.

*^d^*Subcellular localization was predicted by the TargetP program (www.cbs.dtu.dk/services/TargetP) (Emanuelsson, O., *et al.*, 2000) or SecretomeP program (for non-classical secreted protein predication) (<http://www.cbs.dtu.dk/services/SecretomeP-1.0>) (Bendtsen, J. D, *et a.,* 2004). loc, location; SP, secretory pathway; S, predicted location in the apoplast by SecretomeP program. R, reported to be found in the apoplast by references.

*^e^* ns indicates no significant change of spot abundance between treatment and control.

The number marked by * indicates the peptide fragment(s) sequenced by MS/MS. The detailed amino acid sequence of each fragment is listed in Table S1 in Supporting Information.
