# Supplementary material for: Comparative Proteomics Analysis of the Root Apoplasts of Rice Seedlings in Response to Hydrogen Peroxide
Source: PLoS One. 2011 Feb 10;6(2):e16723. doi: 10.1371/journal.pone.0016723 (PMC3037377; doi:10.1371/journal.pone.0016723)
Supplement: Table S2 — Peptide tags of proteins identified by MS/MS. (DOCX) [file pone.0016723.s005.docx]

**Table S2.** Differentially expressed protein spots identified by MS/MS

| **Spot No.** | **NCBI accession no.** | **Protein name** | **Average fold change*^a^*** | | **Score** | **C(%)*^b^*** | **Loc*^c^*** | **Sequence** | **m/z** | **Z*^d^*** |
| --- | --- | --- | --- | --- | --- | --- | --- | --- | --- | --- |
|  |  |  | **T1** | **T2** |  |  |  |  |  |  |
| **Carbohydrate metabolism** | | | | | | | | | | |
| 08 | Q42971 | Enolase | 2.08 | 1.63 | 107 | 10 | R | FRAPVEPY  AAVPSGASTGVYEALELR | 978.57  1791.05 | +1,+1 |
| 09 | AAC49173 | 2-phospho-D-glycerate hydrolas | -2.78 | -3.35 | 141 | 22 | R | AAVPSGASTGVYEALELR  FRAPVEPY | 1791.07  978.57 | +1, +1 |
| 14 | NP_001060639 | Putative α-galactosidase | -3.02 | ns*^e^* | 136 | 17 | SP | EVIQVNQDPLGVQGR  LAVVLWNR  LPSVGLDGSSPYSVR | 1651.99  970.65  1533.89 | +1,+1,  +1 |
| 19 | BAA77785 | β-1,3-glucanase | -3.89 | -2.32 | 98 | 27 | SP | DNIQAYPGVSFR  FDAFADTFPPSSGR | 1366.79  1514.81 | +1,+1 |
| 20 | BAA77785 | β-1,3-glucanase | 10.17 | -3.01 | 192 | 25 | SP | DNIQAYPGVSFR  FDAFADTFPPSSGR  VGASVNNAQTYNQGLINHVR | 1366.81  1514.84  2155.29 | +1,+1,  +1 |
| 21 | BAA77785 | β-1,3-glucanase | -9.21 | 2.03 | 95 | 25 | SP | DNIQAYPGVSFR  FDAFADTFPPSSGR | 1366.79  1514.82 | +1,+1 |
| 22 | NP_001044052 | β-1,3-glucanase precursor | -3.7 | -5.96 | 133 | 16 | SP | DNIQAYPGVSFR  FDAFADTFPPSSGR | 1366.73  1514.76 | +1,+1 |
| 23 | NP_001059883 | Putative β-1,3-glucanase | ns | 1.48 | 97 | 4 | SP | AYNNGLIR  VAHATYAFNDYYQTAGR | 920.55  1948.01 | +1.+1 |
| 24 | NP_001059883 | Putative β-1,3-glucanase | -3.61 | -4.19 | 62 | 6 | SP | VAHATYAFNDYYQTAGR | 1947.98 | +1 |
| 25 | NP_001055377 | β-1,3;1,4-glucanase precursor | -3.23 | -3.12 | 145 | 23 | SP | NNIQAYPSVSFR  IYNQNLINHVGR | 1395.71  1440.78 | +1,+1 |
| **Redox homeostasis** | | | | | | | | | | |
| 28 | CAA46916 | Peroxidase OsPrx 111 | -4.8 | -3.02 | 112 | 31 | SP | GLLHSDQVLFNGGSADNTVR  MGNISPLTGTQGQIR | 2100.10  1572.85 | +1,+1 |
| 30 | NP_001060629 | Peroxidase OsPrx 112 | -4.04 | -4.02 | 250 | 25 | SP | DSVVALGGPSWTVLLGR  GLLHSDQVLFNGGSTDNTVR  MGNISPLTGTQGQIR | 1727.08  2130.23  1572.93 | +1,+1,  +1 |
| 31 | NP_001060629 | Peroxidase OsPrx 112 | -2.62 | -2.18 | 244 | 25 | SP | DSVVALGGPSWTVLLGR  GLLHSDQVLFNGGSTDNTVR  MGNISPLTGTQGQIR | 1727.11  2130.27  1572.96 | +1,+1,  +1 |
| 32 | NP_001045483 | Peroxidase OsPrx 22 | -3.34 | -2.96 | 105 | 18 | SP | DVGLAAALIR  YYVDLQNR | 998.71  1070.63 | +1.+1 |
| 36 | CAH69313 | Peroxidase OsPrx71 precursor | -7.58 | ns | 66 | 25 | SP | MDNQYYR  LYAAGDPAAWLAR | 989.50  1374.84 | +1,+1 |
| 38 | NP_001064860 | Malate dehydrogenase | -2.94 | -1.65 | 145 | 39 | R | MELVDAAFPLLK  NAIIWGNHSSTQYPDVNHATVK | 1346.73  2452.22 | +1,+1 |
| 41 | NP_001067436 | Putative protein disulfide isomerase | -2.89 | 1.11 | 146 | 15 | SP | MVPYESGR  GDAAVERPLVR  VVVADNVHDFVFK  SDYDFGHTLHANHLPR | 938.51  1182.73  1488.88  1880.02 | +1,+1,  +1,+1 |
| **Cell rescue/defense** | | | | | | | | | | |
| 49 | AAO15366 | Chitinase | -2.26 | -1.95 | 63 | 9 | S | FASIAPFGNAEVQR | 1506.85 | +1 |
| 50 | AAC37516 | Chitinase | -4.44 | -2.3 | 85 | 10 | S | ELAAFFGQTSHETTGGT | 1910.08 | +1 |
| **Nucleotide metabolism** | | | | | | | | | | |
| 52 | NP_001042295 | Putative nucleoside diphosphatase | -1.67 | ns | 150 | 17 | S | LEKPVVILISSDGFR  LHYADSYR  TIFIAHGPR | 1673.09  1024.57  1011.66 | +1,+1,  +1 |
| **Protein degradation** | | | | | | | | | | |
| 53 | NP_001047954 | Peptidase A1 | -1.96 | 1.85 | 136 | 9 | SP | QIPTFYGAR  LPPTAYSALSSAFK | 1052.66  1452.91 | +1,+1 |

*^a^* Spot abundance is calculated as the intensity of the up-regulated (plus value) or down-regulated (minus value) protein over the intensity of the control. -Fold changes had *p* values<0.05. T1 and T2 represent 300 µM or 600 µM H_2_O_2_ treatment concentrations, respectively.

*^b^* Sequence coverage.

*^d^*Subcellular localization was predicted by the TargetP program (www.cbs.dtu.dk/services/TargetP) (Emanuelsson, O., et al., 2000) or SecretomeP program (for non-classical secreted protein predication) (<http://www.cbs.dtu.dk/services/SecretomeP-1.0>) (Bendtsen, J. D, *et al*., 2004). loc, location; SP, secretory pathway; S, predicted located in the apoplast by SecretomeP software. R, reported to be found in the apoplast by references.

*^d^* Precursor charge.

*^e^* ns represents no significant change of spot abundance between treatment and control.
