# Supplementary material for: Comparative Proteomics Analysis of the Root Apoplasts of Rice Seedlings in Response to Hydrogen Peroxide
Source: PLoS One. 2011 Feb 10;6(2):e16723. doi: 10.1371/journal.pone.0016723 (PMC3037377; doi:10.1371/journal.pone.0016723)
Supplement: Table S4 — Localization prediction of proteins without signal sequences by the SecretomeP software. (DOCX) [file pone.0016723.s007.docx]

**Table S4.** Localization prediction of proteins without signal sequences by the SecretomeP software

| **Spot No.** | **NCBI accession no.** | **Protein Name** | **Neural Network- score** | **Verified Method** | **Reference^a^** |
| --- | --- | --- | --- | --- | --- |
| 54 | BAD29182 | Putative uncharacterized  protein P0453B09.39 | 0.876 |  |  |
| 52 | NP_001042295 | Putative nucleotide diphosphatase | 0.867 |  | 71 |
| 46 | EAY76707 | Pectinesterase | 0.741 |  |  |
| 49 | AAO15366 | Chitinase | 0.621 |  | 12, 72,73 |
| 50 | AAC37516 | Chitinase | 0.619 |  | 12, 72,73 |
| 39 | NP_001059082 | Methylmalonate  semi-aldehyde dehydrogenase | 0.615 |  |  |
| 51 | NP_001047479 | Putative adenosine kinase |  | Proteomics | 41 |
| 37 | NP_001064860 | Malate dehydrogenase |  | Proteomics, WB | 12, 34, 35 |
| 38 | NP_001064860 | Malate dehydrogenase |  | Proteomics, WB | 12, 34, 35 |
| 09 | AAC49173 | 2-phospho-D-glycerate hydrolase |  | Proteomics, WB, ICC | 27, 28, 36 |
| 07 | Q42971 | Enolase |  | Proteomics, WB, ICC | 27, 28, 36 |
| 08 | Q42971 | Enolase |  | Proteomics, WB, ICC | 27, 28, 36 |
| 10 | NP_001064223 | 2-phosphoglycerate dehydratase |  | Proteomics, WB, ICC | 27, 28, 36 |
| 05 | NP_001044625 | Putative phosphoglycerate mutase |  | Proteomics, ICC | 37, 38 |
| 06 | NP_001063879 | UDP-glucose pyrophosphorylase |  | Proteomics, enzyme activity | 27, 39 |
| 15 | CAA77235 | Reversibly glycosylated polypeptide |  | Proteomics, WB, ICC | 12,40 |
| 40 | NP_001058740 | Putative flavin-containing monooxygenase |  |  |  |
| 45 | ABB47908 | Arm repeat protein |  | Proteomics | 27 |

Proteins without signal peptides were analyzed using SecretomeP (http://www.cbs.dtu.dk/services/SecretomeP-1.0). Scores higher than 0.600 were considered significant, as reported by Bendtsen *et al.* Among the 18 proteins analyzed, 6 were predicted to be potential candidates that may be exported via non-classical secretion. WB, western blot; ICC, immunocytochemistry.

^a^Numbers correspond to the papers listed in the references.

(71) Bischoff E, Tranthi TA, Decker KFA (1975) Nucleotide pyrophosphatase of rat-liver - comparative study on enzymes solubilized and purified from plasma-membrane and endoplasmic-reticulum. Eur J Biochem 51 (2): 353-361.

(72) Collinge DB, Kragh KM, Mikkelsen JD, Nielsen KK, Rasmussen U, et al. (1993) Plant chitinases. Plant J 3 (1): 31-40.

(73) Fink W, Liefland M, Mendgen K (1988) Chitinases and beta-1,3-glucanases in the apoplastic compartment of Oat Leaves (Avena-Sativa L). Plant Physiol 88 (2): 270-275.
