# Supplementary material for: Comparative Proteomics Analysis of the Root Apoplasts of Rice Seedlings in Response to Hydrogen Peroxide
Source: PLoS One. 2011 Feb 10;6(2):e16723. doi: 10.1371/journal.pone.0016723 (PMC3037377; doi:10.1371/journal.pone.0016723)
Supplement: Table S5 — List of common proteins in the rice apoplast proteome under different treatments. (DOCX) [file pone.0016723.s008.docx]

**Table S5.** List of common proteins in the rice apoplast proteome under different treatments

| **Functional Classification** | **Identified protein** | **Spot ID** | | |
| --- | --- | --- | --- | --- |
|  |  | **H_2_O_2_** | **NaCl** [22] | **Dehydration** [13] |
| Carbohydrate metabolism | β-1,3-glucanase | 16-24 | 5,9 |  |
|  | Enolase | 07-10 |  | OsE-136  OsE-138  OsE-194 |
| Redox homeostasis | Class Ⅲ peroxidase | 26-36 | 2,4 |  |
| Signal transduction | OsRMC | 42-44 | 1,6 |  |
| Nucleotide metabolism | Adenosine kinase | 51 |  | OsE-70 |
