# Supplementary material for: Comparative Proteomics Analysis of the Root Apoplasts of Rice Seedlings in Response to Hydrogen Peroxide
Source: PLoS One. 2011 Feb 10;6(2):e16723. doi: 10.1371/journal.pone.0016723 (PMC3037377; doi:10.1371/journal.pone.0016723)
Supplement: File S1 — Supplemental spectra PMF. Annotated spectra for 33 differentially expressed protein spots identified by PMF. (PPT) [file pone.0016723.s009.ppt]

## Slide 1
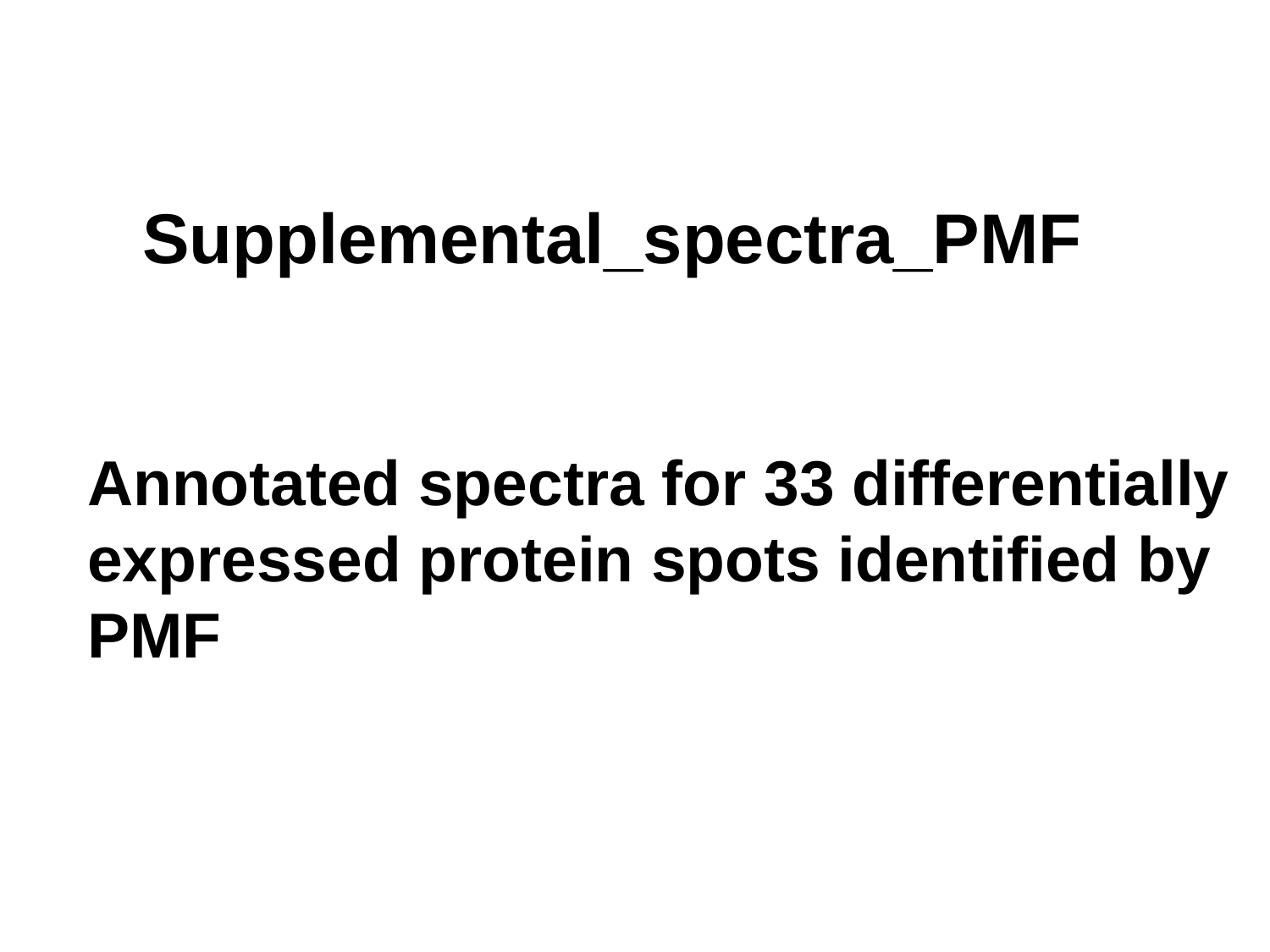

Supplemental_spectra_PMF
# Annotated spectra for 33 differentially expressed protein spots identified by PMF

## Slide 2
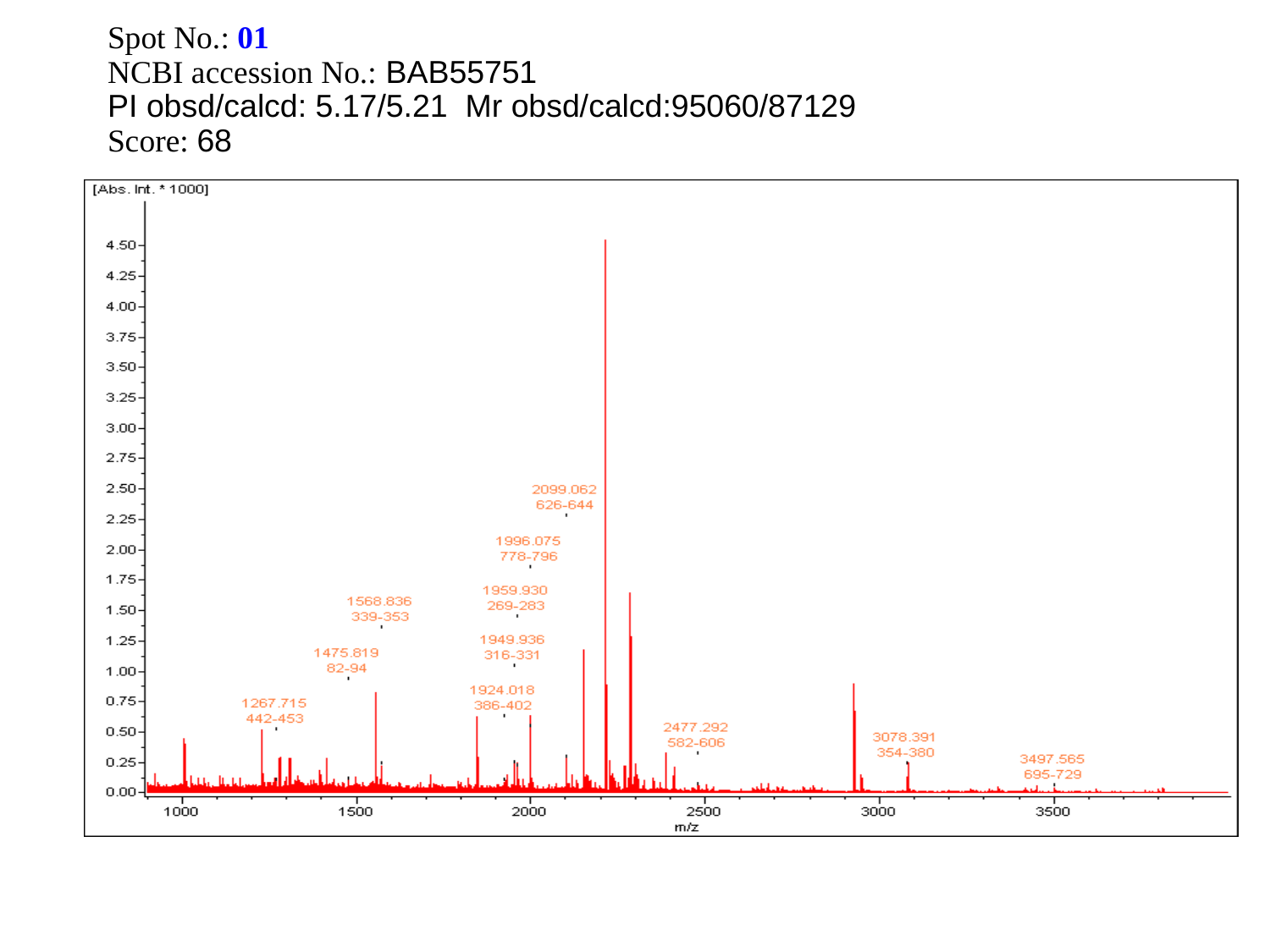

Spot No.: 01
NCBI accession No.: BAB55751
PI obsd/calcd: 5.17/5.21 Mr obsd/calcd:95060/87129
Score: 68

## Slide 3
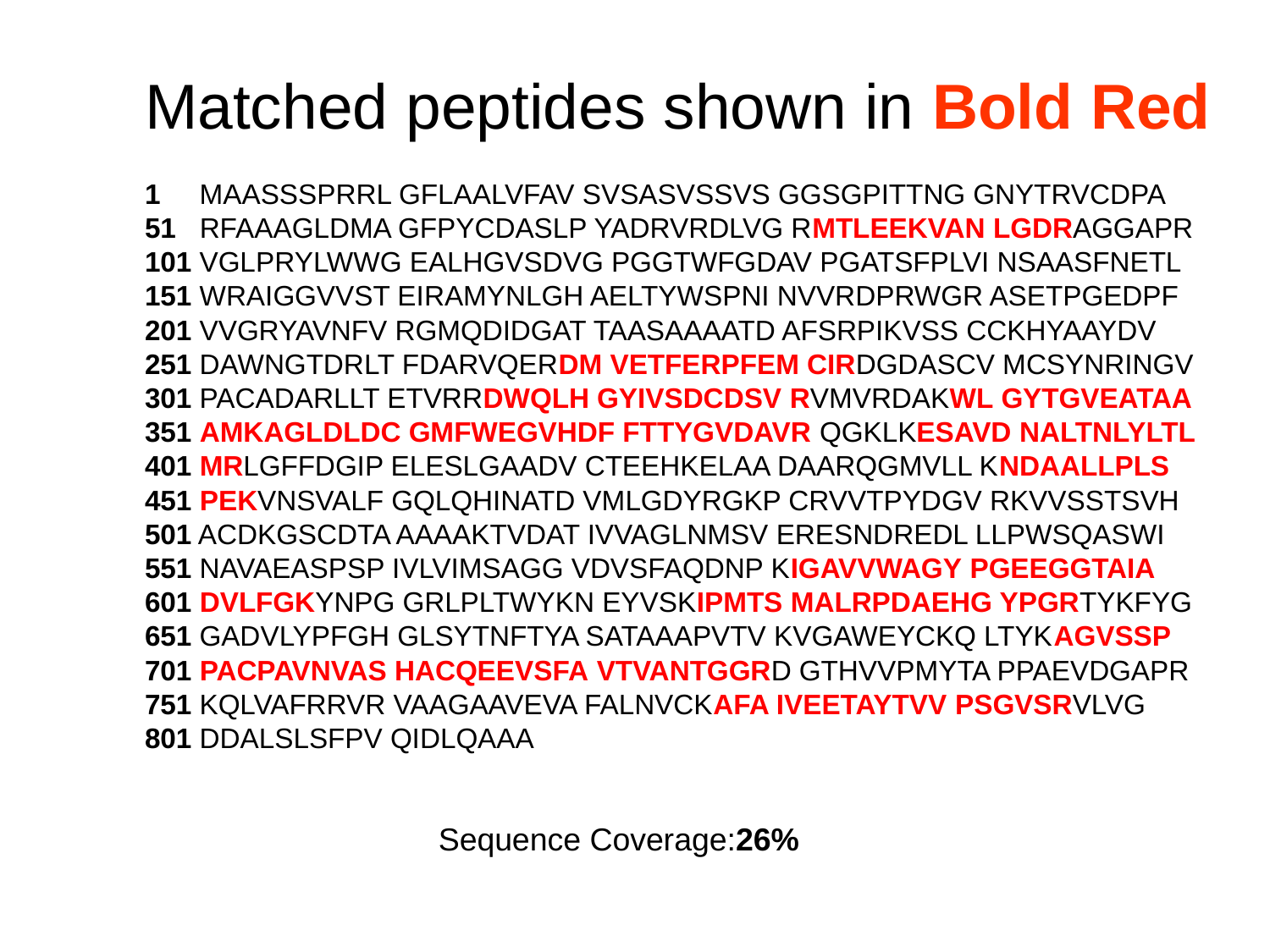

Matched peptides shown in Bold Red
1 MAASSSPRRL GFLAALVFAV SVSASVSSVS GGSGPITTNG GNYTRVCDPA
51 RFAAAGLDMA GFPYCDASLP YADRVRDLVG RMTLEEKVAN LGDRAGGAPR
101 VGLPRYLWWG EALHGVSDVG PGGTWFGDAV PGATSFPLVI NSAASFNETL
151 WRAIGGVVST EIRAMYNLGH AELTYWSPNI NVVRDPRWGR ASETPGEDPF
201 VVGRYAVNFV RGMQDIDGAT TAASAAAATD AFSRPIKVSS CCKHYAAYDV
251 DAWNGTDRLT FDARVQERDM VETFERPFEM CIRDGDASCV MCSYNRINGV
301 PACADARLLT ETVRRDWQLH GYIVSDCDSV RVMVRDAKWL GYTGVEATAA
351 AMKAGLDLDC GMFWEGVHDF FTTYGVDAVR QGKLKESAVD NALTNLYLTL
401 MRLGFFDGIP ELESLGAADV CTEEHKELAA DAARQGMVLL KNDAALLPLS
451 PEKVNSVALF GQLQHINATD VMLGDYRGKP CRVVTPYDGV RKVVSSTSVH
501 ACDKGSCDTA AAAAKTVDAT IVVAGLNMSV ERESNDREDL LLPWSQASWI
551 NAVAEASPSP IVLVIMSAGG VDVSFAQDNP KIGAVVWAGY PGEEGGTAIA
601 DVLFGKYNPG GRLPLTWYKN EYVSKIPMTS MALRPDAEHG YPGRTYKFYG
651 GADVLYPFGH GLSYTNFTYA SATAAAPVTV KVGAWEYCKQ LTYKAGVSSP
701 PACPAVNVAS HACQEEVSFA VTVANTGGRD GTHVVPMYTA PPAEVDGAPR
751 KQLVAFRRVR VAAGAAVEVA FALNVCKAFA IVEETAYTVV PSGVSRVLVG
801 DDALSLSFPV QIDLQAAA
# Sequence Coverage:26%

## Slide 4
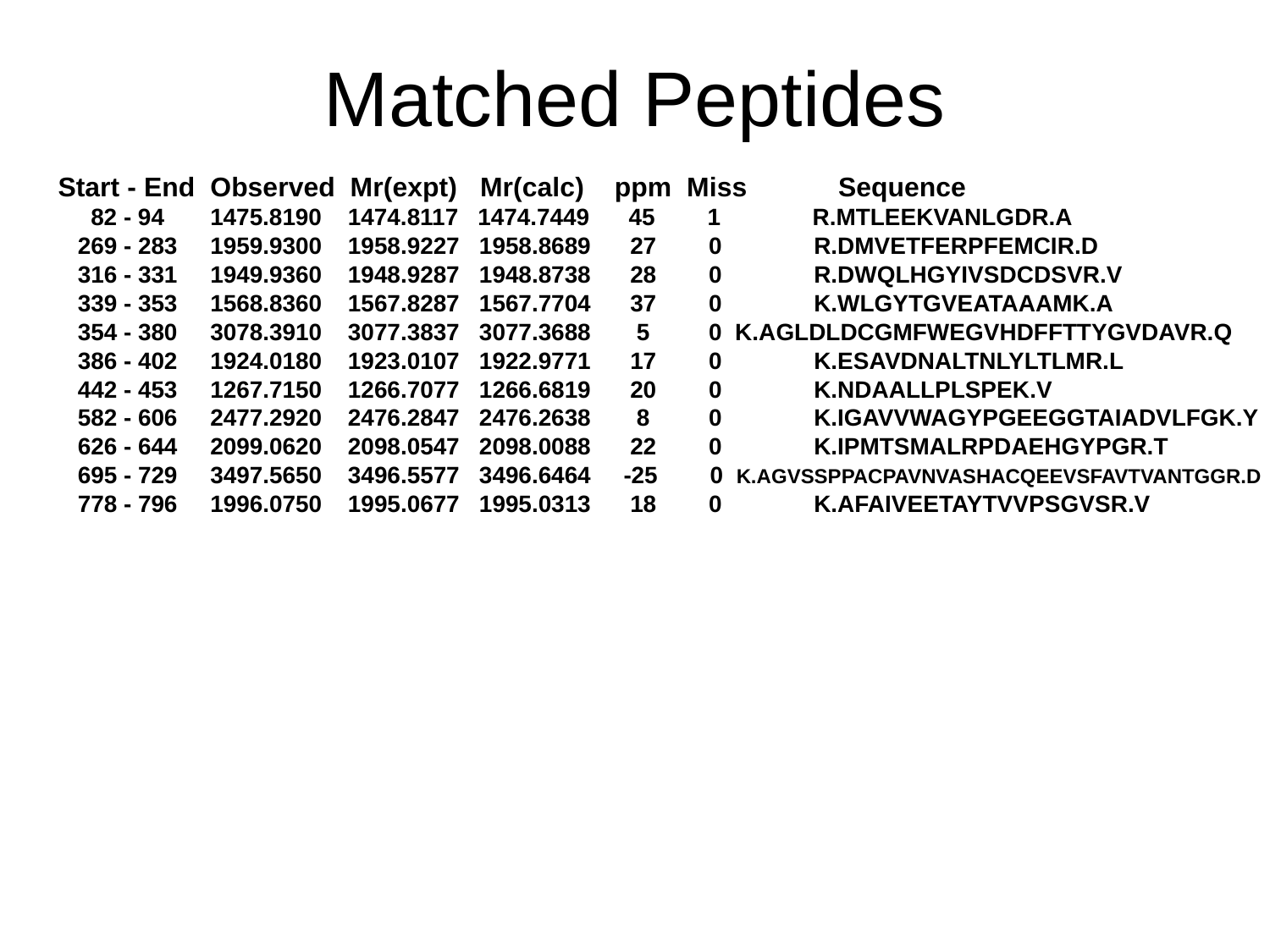

# Matched Peptides
Start - End Observed Mr(expt) Mr(calc) ppm Miss Sequence
 82 - 94 1475.8190 1474.8117 1474.7449 45 1 R.MTLEEKVANLGDR.A
 269 - 283 1959.9300 1958.9227 1958.8689 27 0 R.DMVETFERPFEMCIR.D
 316 - 331 1949.9360 1948.9287 1948.8738 28 0 R.DWQLHGYIVSDCDSVR.V
 339 - 353 1568.8360 1567.8287 1567.7704 37 0 K.WLGYTGVEATAAAMK.A
 354 - 380 3078.3910 3077.3837 3077.3688 5 0 K.AGLDLDCGMFWEGVHDFFTTYGVDAVR.Q
 386 - 402 1924.0180 1923.0107 1922.9771 17 0 K.ESAVDNALTNLYLTLMR.L
 442 - 453 1267.7150 1266.7077 1266.6819 20 0 K.NDAALLPLSPEK.V
 582 - 606 2477.2920 2476.2847 2476.2638 8 0 K.IGAVVWAGYPGEEGGTAIADVLFGK.Y
 626 - 644 2099.0620 2098.0547 2098.0088 22 0 K.IPMTSMALRPDAEHGYPGR.T
 695 - 729 3497.5650 3496.5577 3496.6464 -25 0 K.AGVSSPPACPAVNVASHACQEEVSFAVTVANTGGR.D
 778 - 796 1996.0750 1995.0677 1995.0313 18 0 K.AFAIVEETAYTVVPSGVSR.V

## Slide 5
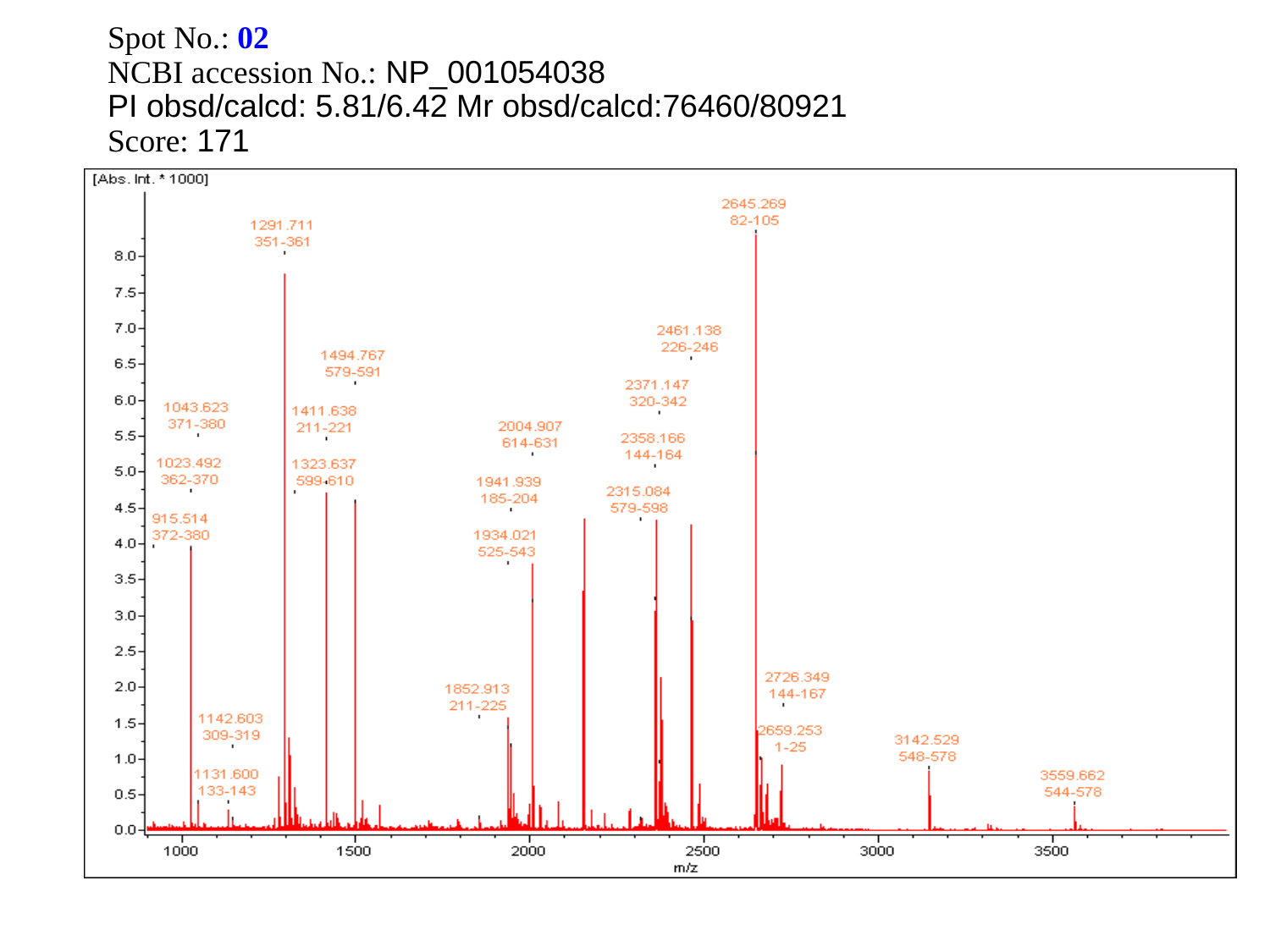

Spot No.: 02
NCBI accession No.: NP_001054038
PI obsd/calcd: 5.81/6.42 Mr obsd/calcd:76460/80921
Score: 171

## Slide 6
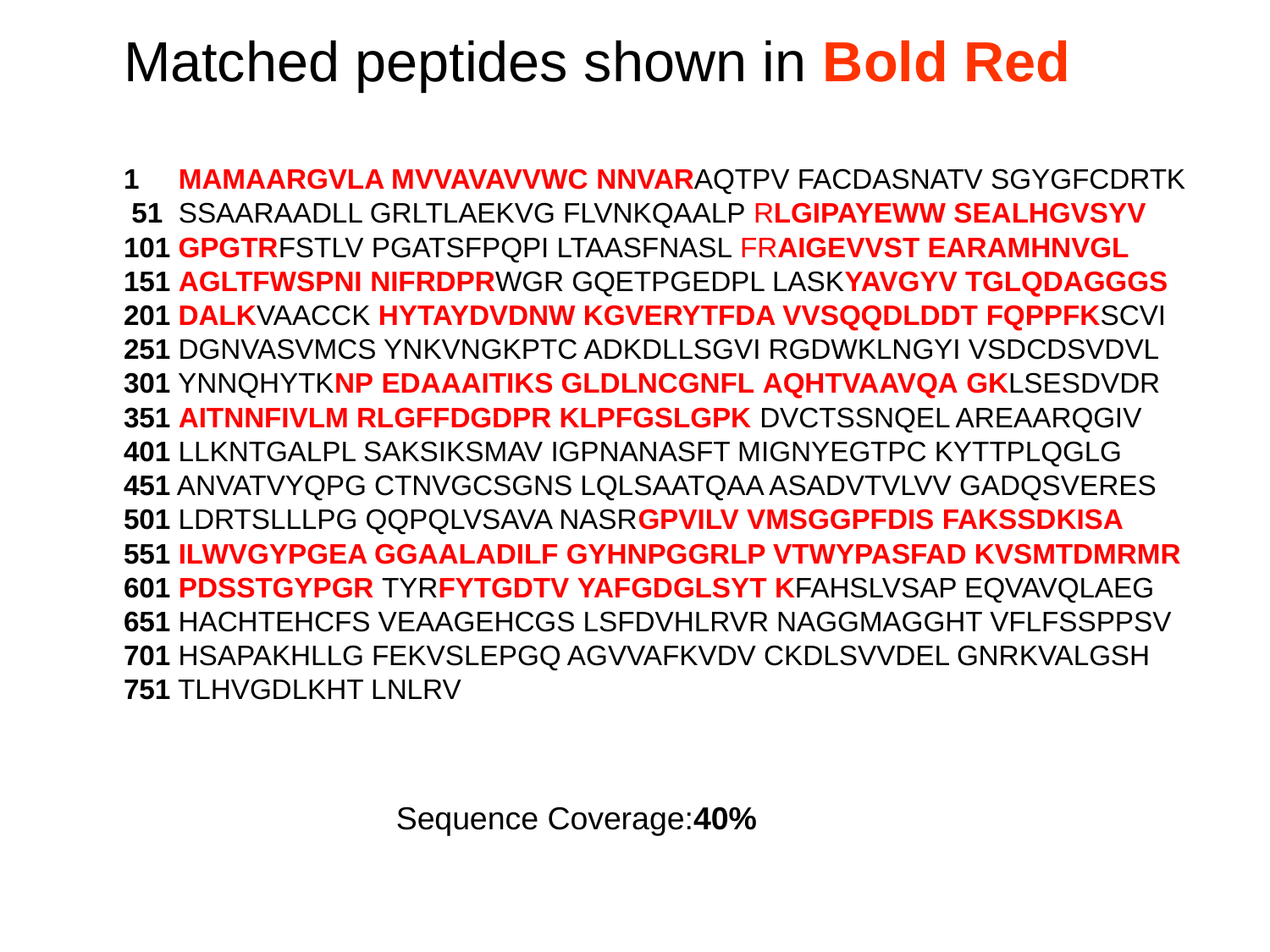

Matched peptides shown in Bold Red
1 MAMAARGVLA MVVAVAVVWC NNVARAQTPV FACDASNATV SGYGFCDRTK
 51 SSAARAADLL GRLTLAEKVG FLVNKQAALP RLGIPAYEWW SEALHGVSYV
101 GPGTRFSTLV PGATSFPQPI LTAASFNASL FRAIGEVVST EARAMHNVGL
151 AGLTFWSPNI NIFRDPRWGR GQETPGEDPL LASKYAVGYV TGLQDAGGGS
201 DALKVAACCK HYTAYDVDNW KGVERYTFDA VVSQQDLDDT FQPPFKSCVI
251 DGNVASVMCS YNKVNGKPTC ADKDLLSGVI RGDWKLNGYI VSDCDSVDVL
301 YNNQHYTKNP EDAAAITIKS GLDLNCGNFL AQHTVAAVQA GKLSESDVDR
351 AITNNFIVLM RLGFFDGDPR KLPFGSLGPK DVCTSSNQEL AREAARQGIV
401 LLKNTGALPL SAKSIKSMAV IGPNANASFT MIGNYEGTPC KYTTPLQGLG
451 ANVATVYQPG CTNVGCSGNS LQLSAATQAA ASADVTVLVV GADQSVERES
501 LDRTSLLLPG QQPQLVSAVA NASRGPVILV VMSGGPFDIS FAKSSDKISA
551 ILWVGYPGEA GGAALADILF GYHNPGGRLP VTWYPASFAD KVSMTDMRMR
601 PDSSTGYPGR TYRFYTGDTV YAFGDGLSYT KFAHSLVSAP EQVAVQLAEG
651 HACHTEHCFS VEAAGEHCGS LSFDVHLRVR NAGGMAGGHT VFLFSSPPSV
701 HSAPAKHLLG FEKVSLEPGQ AGVVAFKVDV CKDLSVVDEL GNRKVALGSH
751 TLHVGDLKHT LNLRV
# Sequence Coverage:40%

## Slide 7
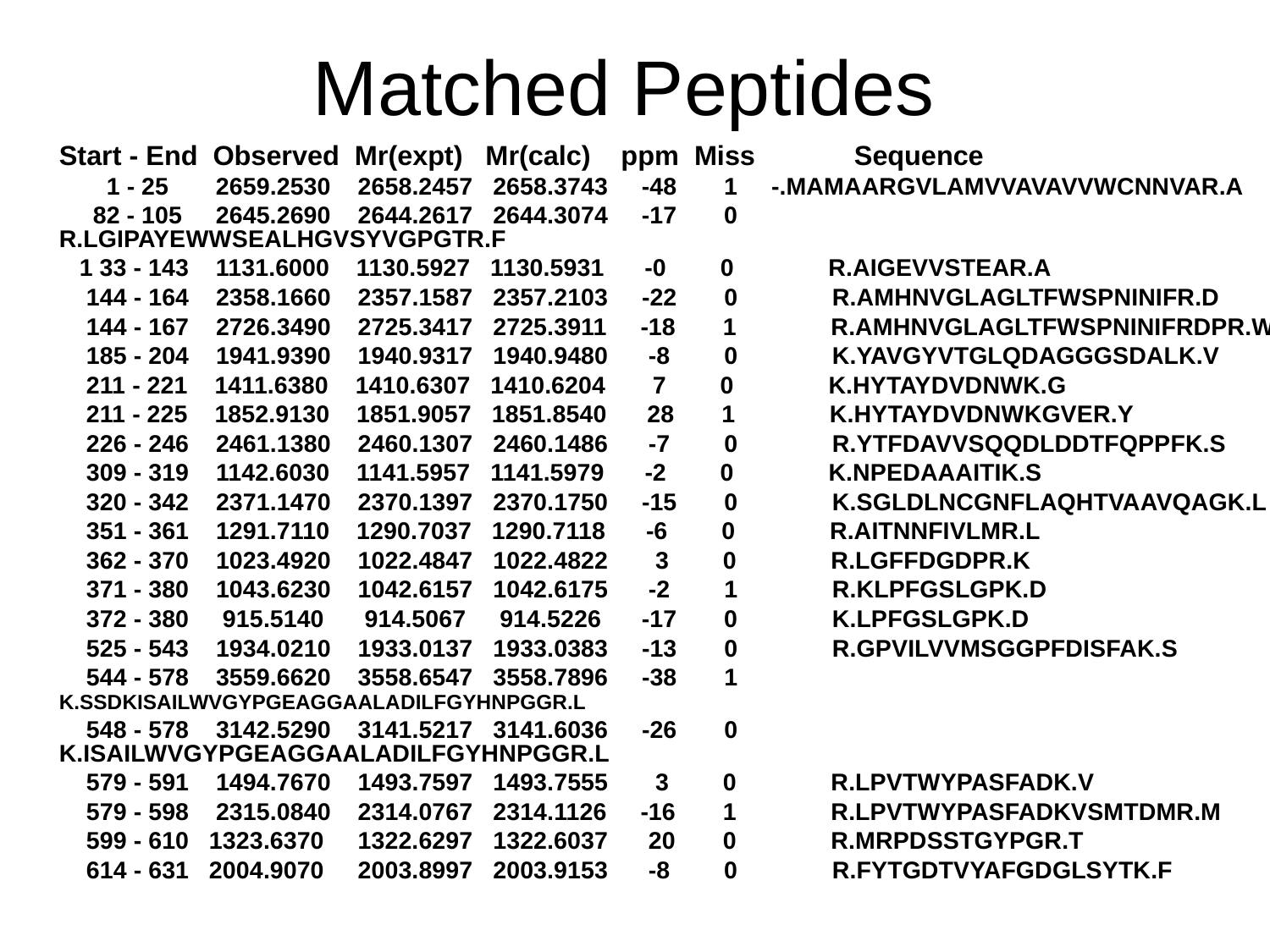

# Matched Peptides
Start - End Observed Mr(expt) Mr(calc) ppm Miss Sequence
 1 - 25 2659.2530 2658.2457 2658.3743 -48 1 -.MAMAARGVLAMVVAVAVVWCNNVAR.A
 82 - 105 2645.2690 2644.2617 2644.3074 -17 0 R.LGIPAYEWWSEALHGVSYVGPGTR.F
 1 33 - 143 1131.6000 1130.5927 1130.5931 -0 0 R.AIGEVVSTEAR.A
 144 - 164 2358.1660 2357.1587 2357.2103 -22 0 R.AMHNVGLAGLTFWSPNINIFR.D
 144 - 167 2726.3490 2725.3417 2725.3911 -18 1 R.AMHNVGLAGLTFWSPNINIFRDPR.W
 185 - 204 1941.9390 1940.9317 1940.9480 -8 0 K.YAVGYVTGLQDAGGGSDALK.V
 211 - 221 1411.6380 1410.6307 1410.6204 7 0 K.HYTAYDVDNWK.G
 211 - 225 1852.9130 1851.9057 1851.8540 28 1 K.HYTAYDVDNWKGVER.Y
 226 - 246 2461.1380 2460.1307 2460.1486 -7 0 R.YTFDAVVSQQDLDDTFQPPFK.S
 309 - 319 1142.6030 1141.5957 1141.5979 -2 0 K.NPEDAAAITIK.S
 320 - 342 2371.1470 2370.1397 2370.1750 -15 0 K.SGLDLNCGNFLAQHTVAAVQAGK.L
 351 - 361 1291.7110 1290.7037 1290.7118 -6 0 R.AITNNFIVLMR.L
 362 - 370 1023.4920 1022.4847 1022.4822 3 0 R.LGFFDGDPR.K
 371 - 380 1043.6230 1042.6157 1042.6175 -2 1 R.KLPFGSLGPK.D
 372 - 380 915.5140 914.5067 914.5226 -17 0 K.LPFGSLGPK.D
 525 - 543 1934.0210 1933.0137 1933.0383 -13 0 R.GPVILVVMSGGPFDISFAK.S
 544 - 578 3559.6620 3558.6547 3558.7896 -38 1 K.SSDKISAILWVGYPGEAGGAALADILFGYHNPGGR.L
 548 - 578 3142.5290 3141.5217 3141.6036 -26 0 K.ISAILWVGYPGEAGGAALADILFGYHNPGGR.L
 579 - 591 1494.7670 1493.7597 1493.7555 3 0 R.LPVTWYPASFADK.V
 579 - 598 2315.0840 2314.0767 2314.1126 -16 1 R.LPVTWYPASFADKVSMTDMR.M
 599 - 610 1323.6370 1322.6297 1322.6037 20 0 R.MRPDSSTGYPGR.T
 614 - 631 2004.9070 2003.8997 2003.9153 -8 0 R.FYTGDTVYAFGDGLSYTK.F

## Slide 8
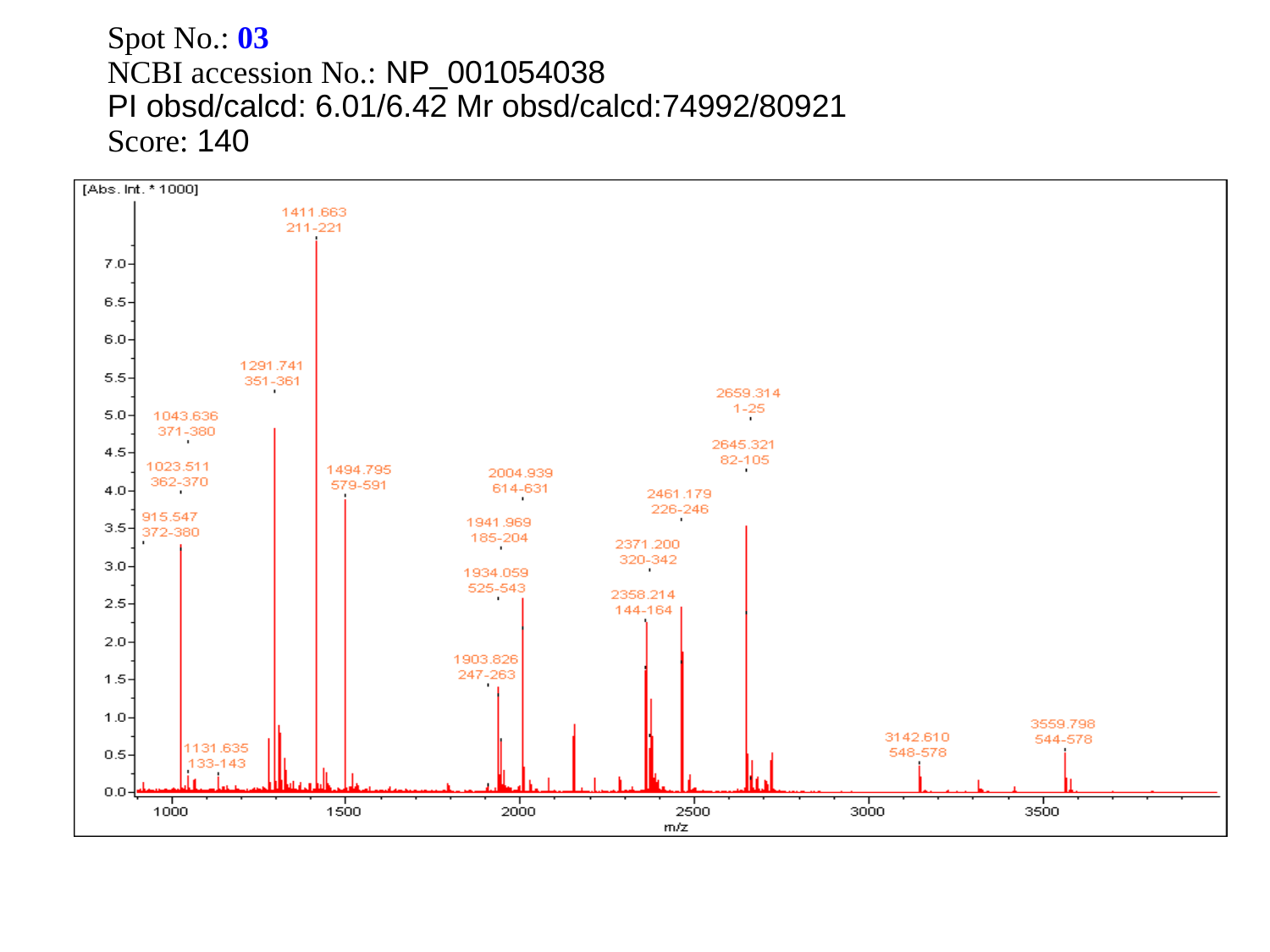

Spot No.: 03
NCBI accession No.: NP_001054038
PI obsd/calcd: 6.01/6.42 Mr obsd/calcd:74992/80921
Score: 140

## Slide 9
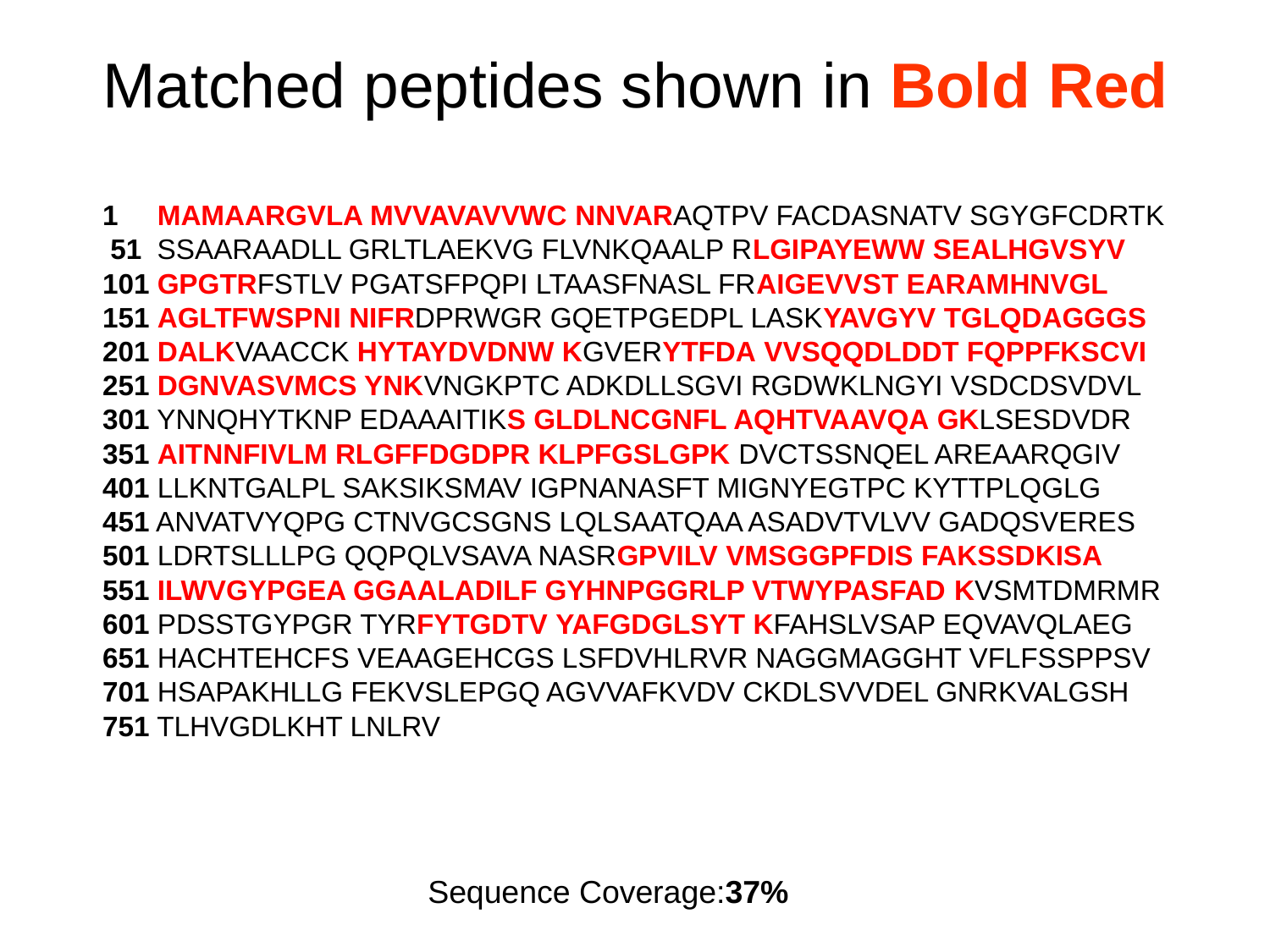

Matched peptides shown in Bold Red
1 MAMAARGVLA MVVAVAVVWC NNVARAQTPV FACDASNATV SGYGFCDRTK
 51 SSAARAADLL GRLTLAEKVG FLVNKQAALP RLGIPAYEWW SEALHGVSYV
101 GPGTRFSTLV PGATSFPQPI LTAASFNASL FRAIGEVVST EARAMHNVGL
151 AGLTFWSPNI NIFRDPRWGR GQETPGEDPL LASKYAVGYV TGLQDAGGGS
201 DALKVAACCK HYTAYDVDNW KGVERYTFDA VVSQQDLDDT FQPPFKSCVI
251 DGNVASVMCS YNKVNGKPTC ADKDLLSGVI RGDWKLNGYI VSDCDSVDVL
301 YNNQHYTKNP EDAAAITIKS GLDLNCGNFL AQHTVAAVQA GKLSESDVDR
351 AITNNFIVLM RLGFFDGDPR KLPFGSLGPK DVCTSSNQEL AREAARQGIV
401 LLKNTGALPL SAKSIKSMAV IGPNANASFT MIGNYEGTPC KYTTPLQGLG
451 ANVATVYQPG CTNVGCSGNS LQLSAATQAA ASADVTVLVV GADQSVERES
501 LDRTSLLLPG QQPQLVSAVA NASRGPVILV VMSGGPFDIS FAKSSDKISA
551 ILWVGYPGEA GGAALADILF GYHNPGGRLP VTWYPASFAD KVSMTDMRMR
601 PDSSTGYPGR TYRFYTGDTV YAFGDGLSYT KFAHSLVSAP EQVAVQLAEG
651 HACHTEHCFS VEAAGEHCGS LSFDVHLRVR NAGGMAGGHT VFLFSSPPSV
701 HSAPAKHLLG FEKVSLEPGQ AGVVAFKVDV CKDLSVVDEL GNRKVALGSH
751 TLHVGDLKHT LNLRV
# Sequence Coverage:37%

## Slide 10
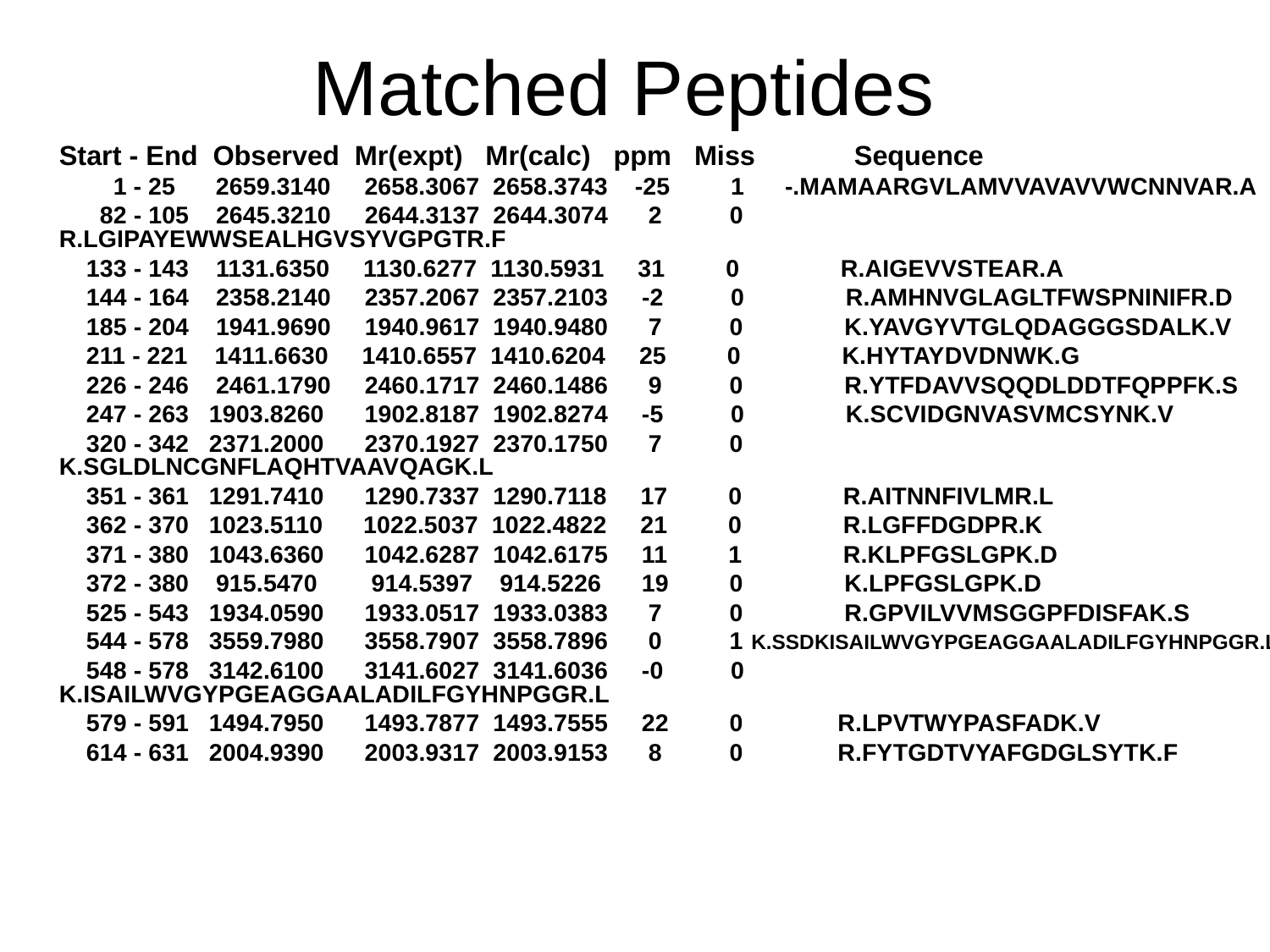

# Matched Peptides
Start - End Observed Mr(expt) Mr(calc) ppm Miss Sequence
 1 - 25 2659.3140 2658.3067 2658.3743 -25 1 -.MAMAARGVLAMVVAVAVVWCNNVAR.A
 82 - 105 2645.3210 2644.3137 2644.3074 2 0 R.LGIPAYEWWSEALHGVSYVGPGTR.F
 133 - 143 1131.6350 1130.6277 1130.5931 31 0 R.AIGEVVSTEAR.A
 144 - 164 2358.2140 2357.2067 2357.2103 -2 0 R.AMHNVGLAGLTFWSPNINIFR.D
 185 - 204 1941.9690 1940.9617 1940.9480 7 0 K.YAVGYVTGLQDAGGGSDALK.V
 211 - 221 1411.6630 1410.6557 1410.6204 25 0 K.HYTAYDVDNWK.G
 226 - 246 2461.1790 2460.1717 2460.1486 9 0 R.YTFDAVVSQQDLDDTFQPPFK.S
 247 - 263 1903.8260 1902.8187 1902.8274 -5 0 K.SCVIDGNVASVMCSYNK.V
 320 - 342 2371.2000 2370.1927 2370.1750 7 0 K.SGLDLNCGNFLAQHTVAAVQAGK.L
 351 - 361 1291.7410 1290.7337 1290.7118 17 0 R.AITNNFIVLMR.L
 362 - 370 1023.5110 1022.5037 1022.4822 21 0 R.LGFFDGDPR.K
 371 - 380 1043.6360 1042.6287 1042.6175 11 1 R.KLPFGSLGPK.D
 372 - 380 915.5470 914.5397 914.5226 19 0 K.LPFGSLGPK.D
 525 - 543 1934.0590 1933.0517 1933.0383 7 0 R.GPVILVVMSGGPFDISFAK.S
 544 - 578 3559.7980 3558.7907 3558.7896 0 1 K.SSDKISAILWVGYPGEAGGAALADILFGYHNPGGR.L
 548 - 578 3142.6100 3141.6027 3141.6036 -0 0 K.ISAILWVGYPGEAGGAALADILFGYHNPGGR.L
 579 - 591 1494.7950 1493.7877 1493.7555 22 0 R.LPVTWYPASFADK.V
 614 - 631 2004.9390 2003.9317 2003.9153 8 0 R.FYTGDTVYAFGDGLSYTK.F

## Slide 11
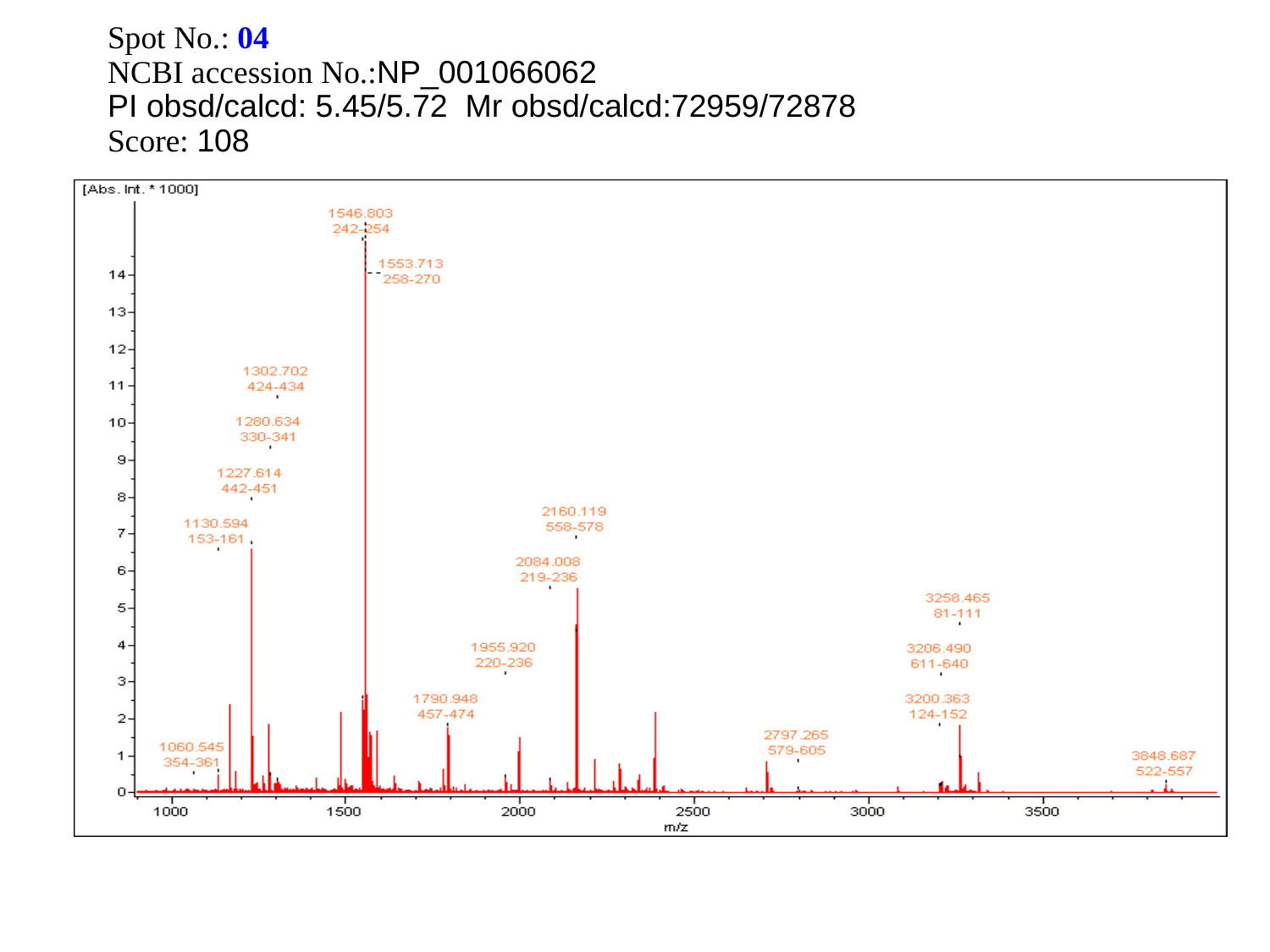

Spot No.: 04
NCBI accession No.:NP_001066062
PI obsd/calcd: 5.45/5.72 Mr obsd/calcd:72959/72878
Score: 108

## Slide 12
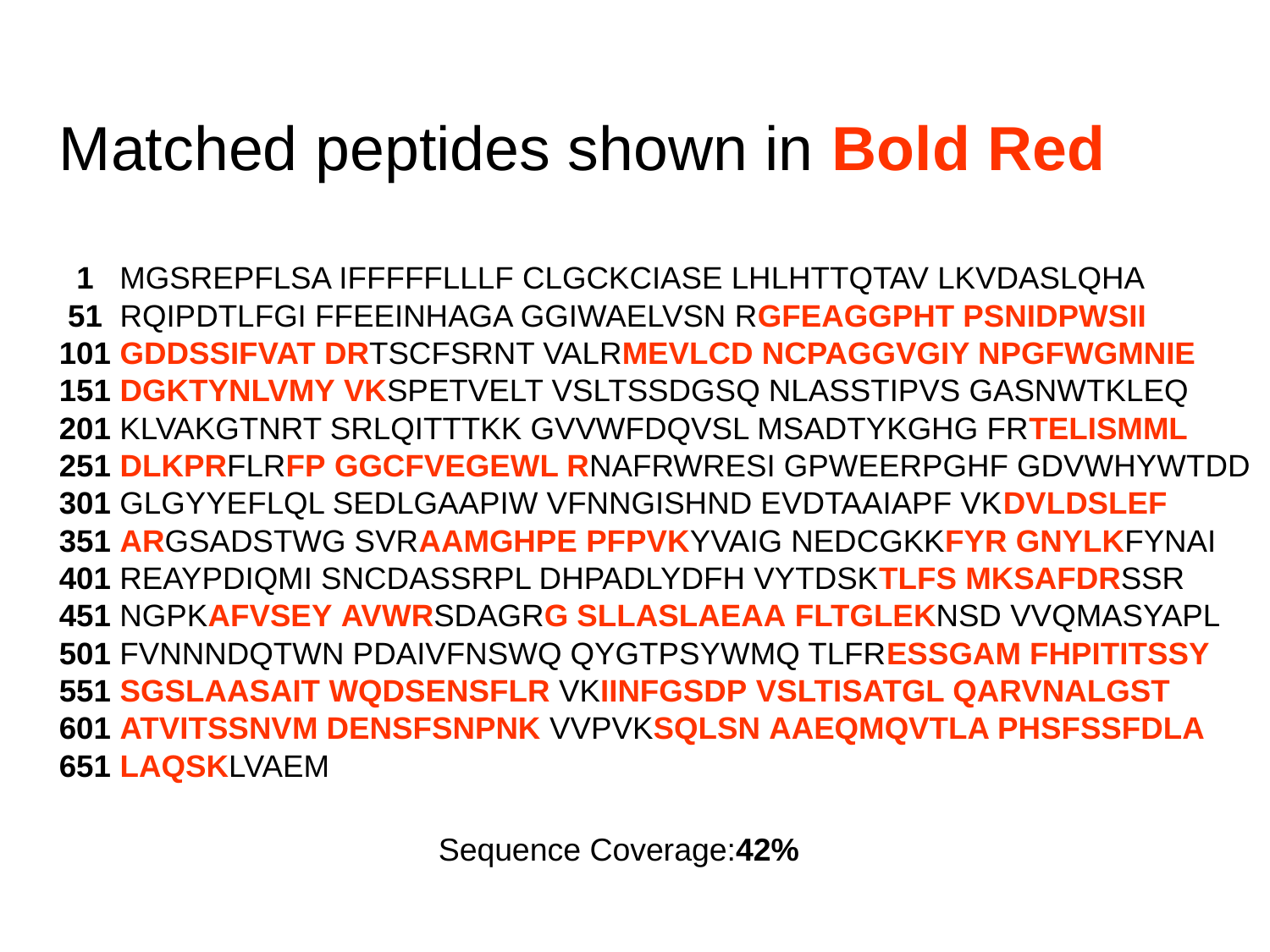

Matched peptides shown in Bold Red
 1 MGSREPFLSA IFFFFFLLLF CLGCKCIASE LHLHTTQTAV LKVDASLQHA
 51 RQIPDTLFGI FFEEINHAGA GGIWAELVSN RGFEAGGPHT PSNIDPWSII
101 GDDSSIFVAT DRTSCFSRNT VALRMEVLCD NCPAGGVGIY NPGFWGMNIE
151 DGKTYNLVMY VKSPETVELT VSLTSSDGSQ NLASSTIPVS GASNWTKLEQ
201 KLVAKGTNRT SRLQITTTKK GVVWFDQVSL MSADTYKGHG FRTELISMML
251 DLKPRFLRFP GGCFVEGEWL RNAFRWRESI GPWEERPGHF GDVWHYWTDD
301 GLGYYEFLQL SEDLGAAPIW VFNNGISHND EVDTAAIAPF VKDVLDSLEF
351 ARGSADSTWG SVRAAMGHPE PFPVKYVAIG NEDCGKKFYR GNYLKFYNAI
401 REAYPDIQMI SNCDASSRPL DHPADLYDFH VYTDSKTLFS MKSAFDRSSR
451 NGPKAFVSEY AVWRSDAGRG SLLASLAEAA FLTGLEKNSD VVQMASYAPL
501 FVNNNDQTWN PDAIVFNSWQ QYGTPSYWMQ TLFRESSGAM FHPITITSSY
551 SGSLAASAIT WQDSENSFLR VKIINFGSDP VSLTISATGL QARVNALGST
601 ATVITSSNVM DENSFSNPNK VVPVKSQLSN AAEQMQVTLA PHSFSSFDLA
651 LAQSKLVAEM
# Sequence Coverage:42%

## Slide 13
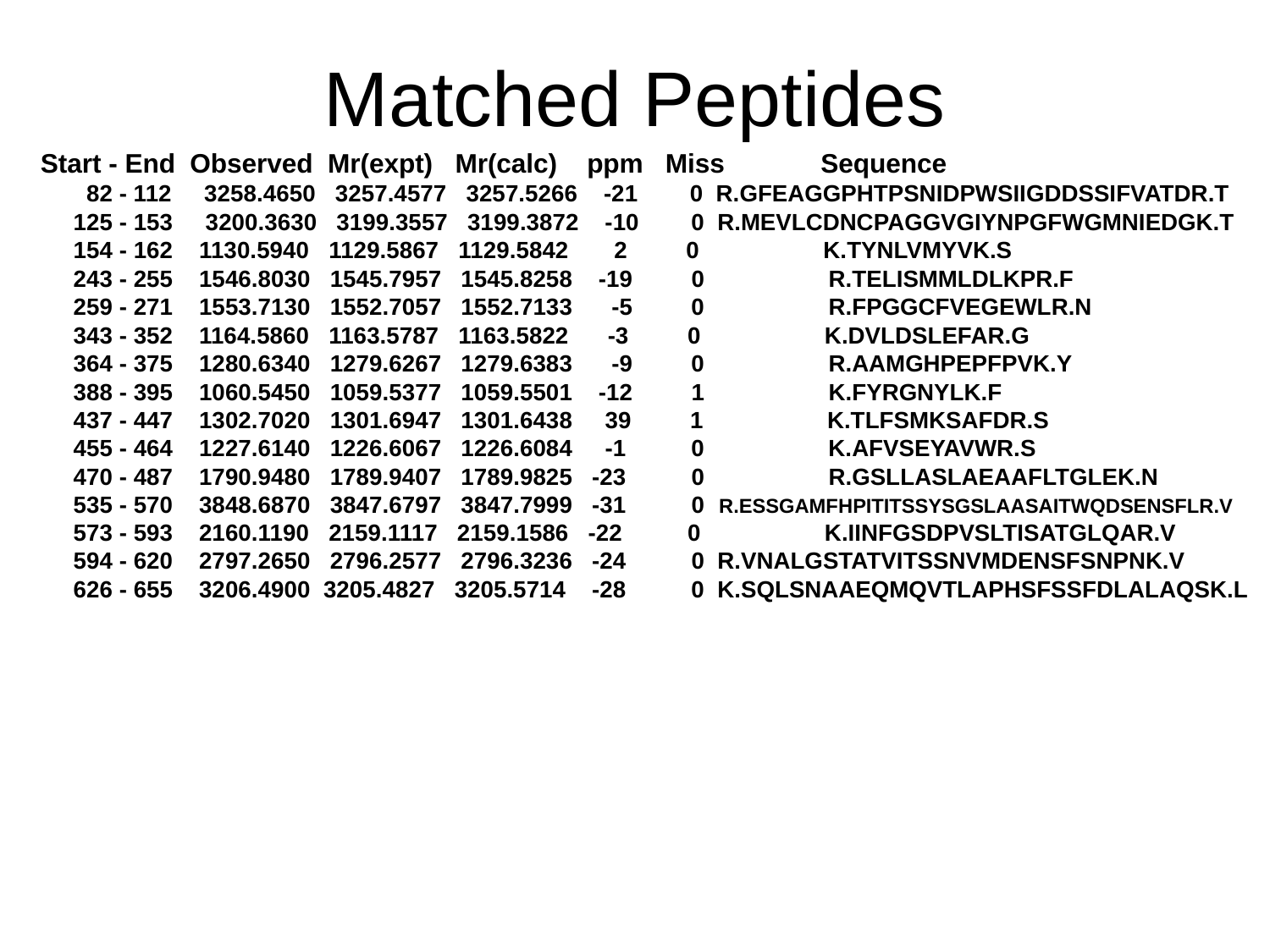

# Matched Peptides
Start - End Observed Mr(expt) Mr(calc) ppm Miss Sequence
 82 - 112 3258.4650 3257.4577 3257.5266 -21 0 R.GFEAGGPHTPSNIDPWSIIGDDSSIFVATDR.T
 125 - 153 3200.3630 3199.3557 3199.3872 -10 0 R.MEVLCDNCPAGGVGIYNPGFWGMNIEDGK.T
 154 - 162 1130.5940 1129.5867 1129.5842 2 0 K.TYNLVMYVK.S
 243 - 255 1546.8030 1545.7957 1545.8258 -19 0 R.TELISMMLDLKPR.F
 259 - 271 1553.7130 1552.7057 1552.7133 -5 0 R.FPGGCFVEGEWLR.N
 343 - 352 1164.5860 1163.5787 1163.5822 -3 0 K.DVLDSLEFAR.G
 364 - 375 1280.6340 1279.6267 1279.6383 -9 0 R.AAMGHPEPFPVK.Y
 388 - 395 1060.5450 1059.5377 1059.5501 -12 1 K.FYRGNYLK.F
 437 - 447 1302.7020 1301.6947 1301.6438 39 1 K.TLFSMKSAFDR.S
 455 - 464 1227.6140 1226.6067 1226.6084 -1 0 K.AFVSEYAVWR.S
 470 - 487 1790.9480 1789.9407 1789.9825 -23 0 R.GSLLASLAEAAFLTGLEK.N
 535 - 570 3848.6870 3847.6797 3847.7999 -31 0 R.ESSGAMFHPITITSSYSGSLAASAITWQDSENSFLR.V
 573 - 593 2160.1190 2159.1117 2159.1586 -22 0 K.IINFGSDPVSLTISATGLQAR.V
 594 - 620 2797.2650 2796.2577 2796.3236 -24 0 R.VNALGSTATVITSSNVMDENSFSNPNK.V
 626 - 655 3206.4900 3205.4827 3205.5714 -28 0 K.SQLSNAAEQMQVTLAPHSFSSFDLALAQSK.L

## Slide 14
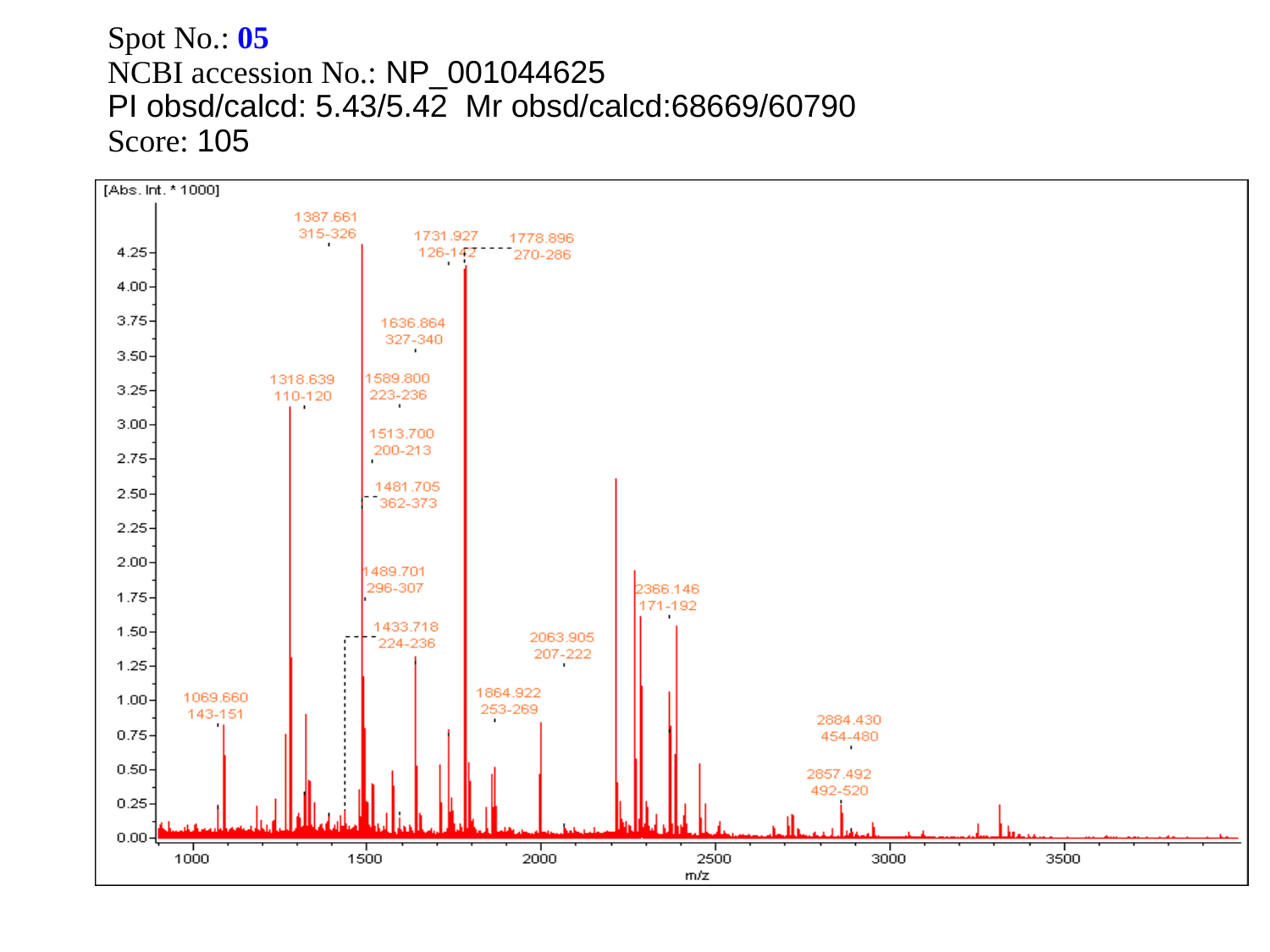

Spot No.: 05
NCBI accession No.: NP_001044625
PI obsd/calcd: 5.43/5.42 Mr obsd/calcd:68669/60790
Score: 105

## Slide 15
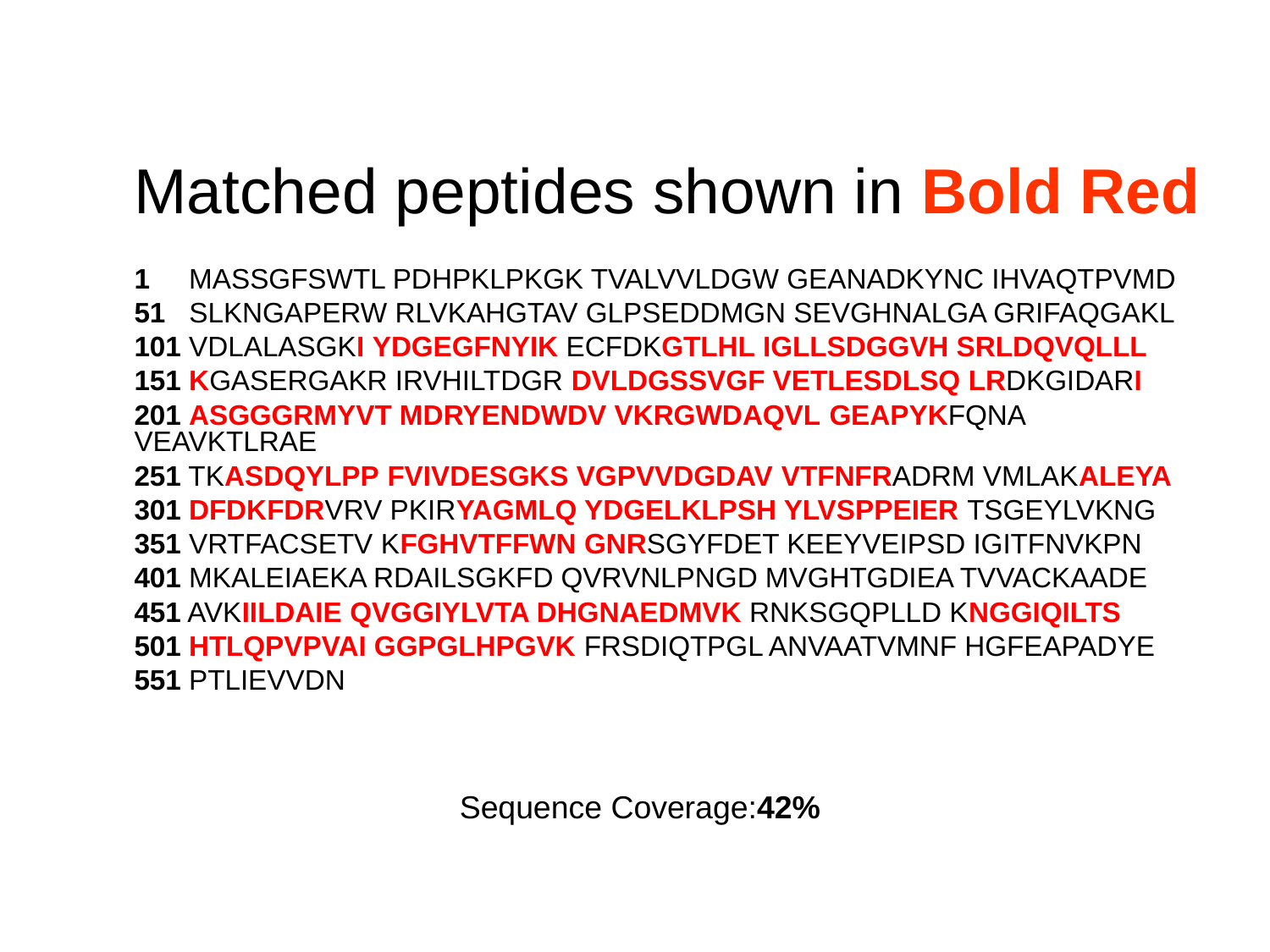

Matched peptides shown in Bold Red
1 MASSGFSWTL PDHPKLPKGK TVALVVLDGW GEANADKYNC IHVAQTPVMD
51 SLKNGAPERW RLVKAHGTAV GLPSEDDMGN SEVGHNALGA GRIFAQGAKL
101 VDLALASGKI YDGEGFNYIK ECFDKGTLHL IGLLSDGGVH SRLDQVQLLL
151 KGASERGAKR IRVHILTDGR DVLDGSSVGF VETLESDLSQ LRDKGIDARI
201 ASGGGRMYVT MDRYENDWDV VKRGWDAQVL GEAPYKFQNA VEAVKTLRAE
251 TKASDQYLPP FVIVDESGKS VGPVVDGDAV VTFNFRADRM VMLAKALEYA
301 DFDKFDRVRV PKIRYAGMLQ YDGELKLPSH YLVSPPEIER TSGEYLVKNG
351 VRTFACSETV KFGHVTFFWN GNRSGYFDET KEEYVEIPSD IGITFNVKPN
401 MKALEIAEKA RDAILSGKFD QVRVNLPNGD MVGHTGDIEA TVVACKAADE
451 AVKIILDAIE QVGGIYLVTA DHGNAEDMVK RNKSGQPLLD KNGGIQILTS
501 HTLQPVPVAI GGPGLHPGVK FRSDIQTPGL ANVAATVMNF HGFEAPADYE
551 PTLIEVVDN
# Sequence Coverage:42%

## Slide 16
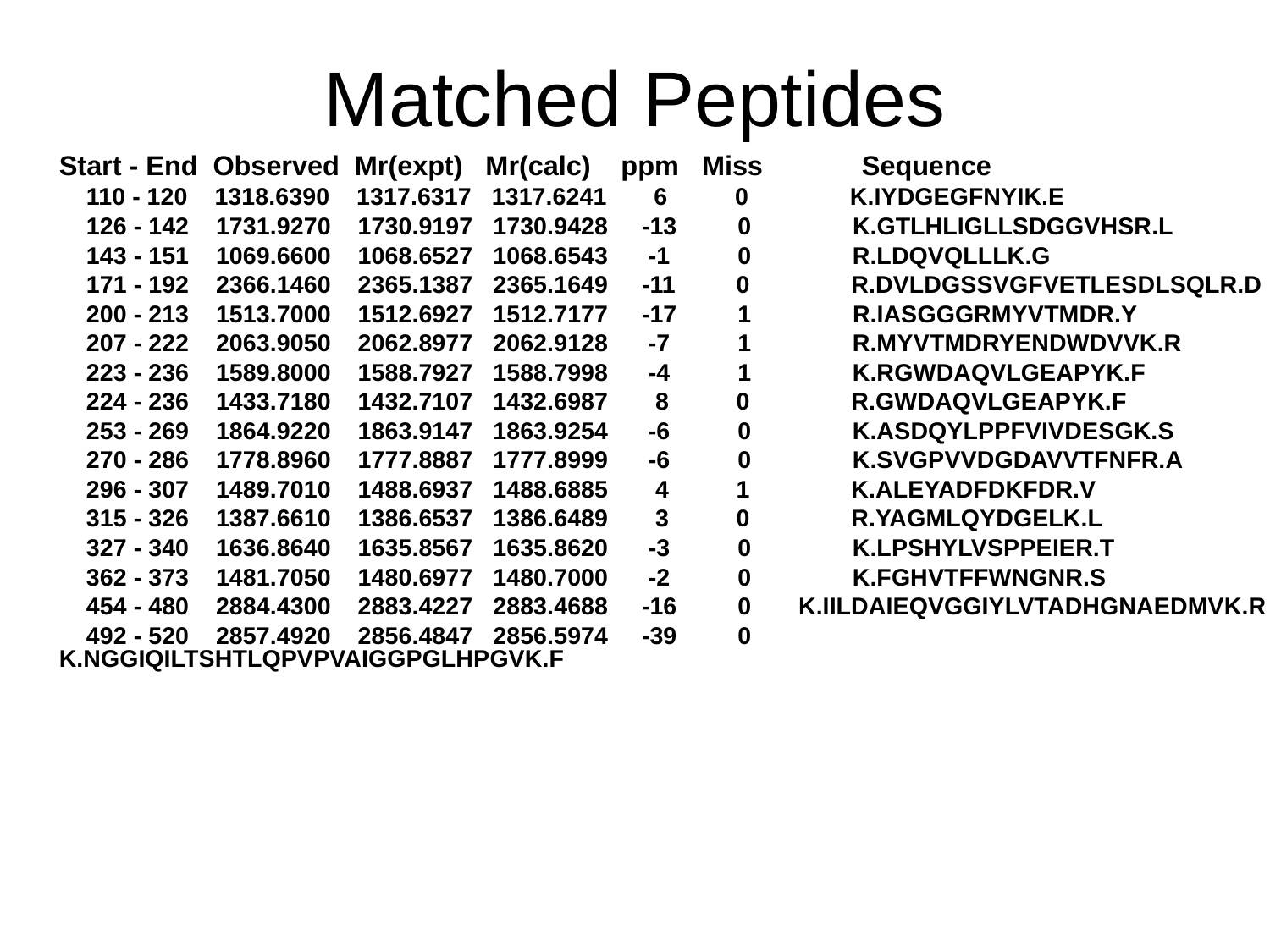

# Matched Peptides
Start - End Observed Mr(expt) Mr(calc) ppm Miss Sequence
 110 - 120 1318.6390 1317.6317 1317.6241 6 0 K.IYDGEGFNYIK.E
 126 - 142 1731.9270 1730.9197 1730.9428 -13 0 K.GTLHLIGLLSDGGVHSR.L
 143 - 151 1069.6600 1068.6527 1068.6543 -1 0 R.LDQVQLLLK.G
 171 - 192 2366.1460 2365.1387 2365.1649 -11 0 R.DVLDGSSVGFVETLESDLSQLR.D
 200 - 213 1513.7000 1512.6927 1512.7177 -17 1 R.IASGGGRMYVTMDR.Y
 207 - 222 2063.9050 2062.8977 2062.9128 -7 1 R.MYVTMDRYENDWDVVK.R
 223 - 236 1589.8000 1588.7927 1588.7998 -4 1 K.RGWDAQVLGEAPYK.F
 224 - 236 1433.7180 1432.7107 1432.6987 8 0 R.GWDAQVLGEAPYK.F
 253 - 269 1864.9220 1863.9147 1863.9254 -6 0 K.ASDQYLPPFVIVDESGK.S
 270 - 286 1778.8960 1777.8887 1777.8999 -6 0 K.SVGPVVDGDAVVTFNFR.A
 296 - 307 1489.7010 1488.6937 1488.6885 4 1 K.ALEYADFDKFDR.V
 315 - 326 1387.6610 1386.6537 1386.6489 3 0 R.YAGMLQYDGELK.L
 327 - 340 1636.8640 1635.8567 1635.8620 -3 0 K.LPSHYLVSPPEIER.T
 362 - 373 1481.7050 1480.6977 1480.7000 -2 0 K.FGHVTFFWNGNR.S
 454 - 480 2884.4300 2883.4227 2883.4688 -16 0 K.IILDAIEQVGGIYLVTADHGNAEDMVK.R
 492 - 520 2857.4920 2856.4847 2856.5974 -39 0 K.NGGIQILTSHTLQPVPVAIGGPGLHPGVK.F

## Slide 17
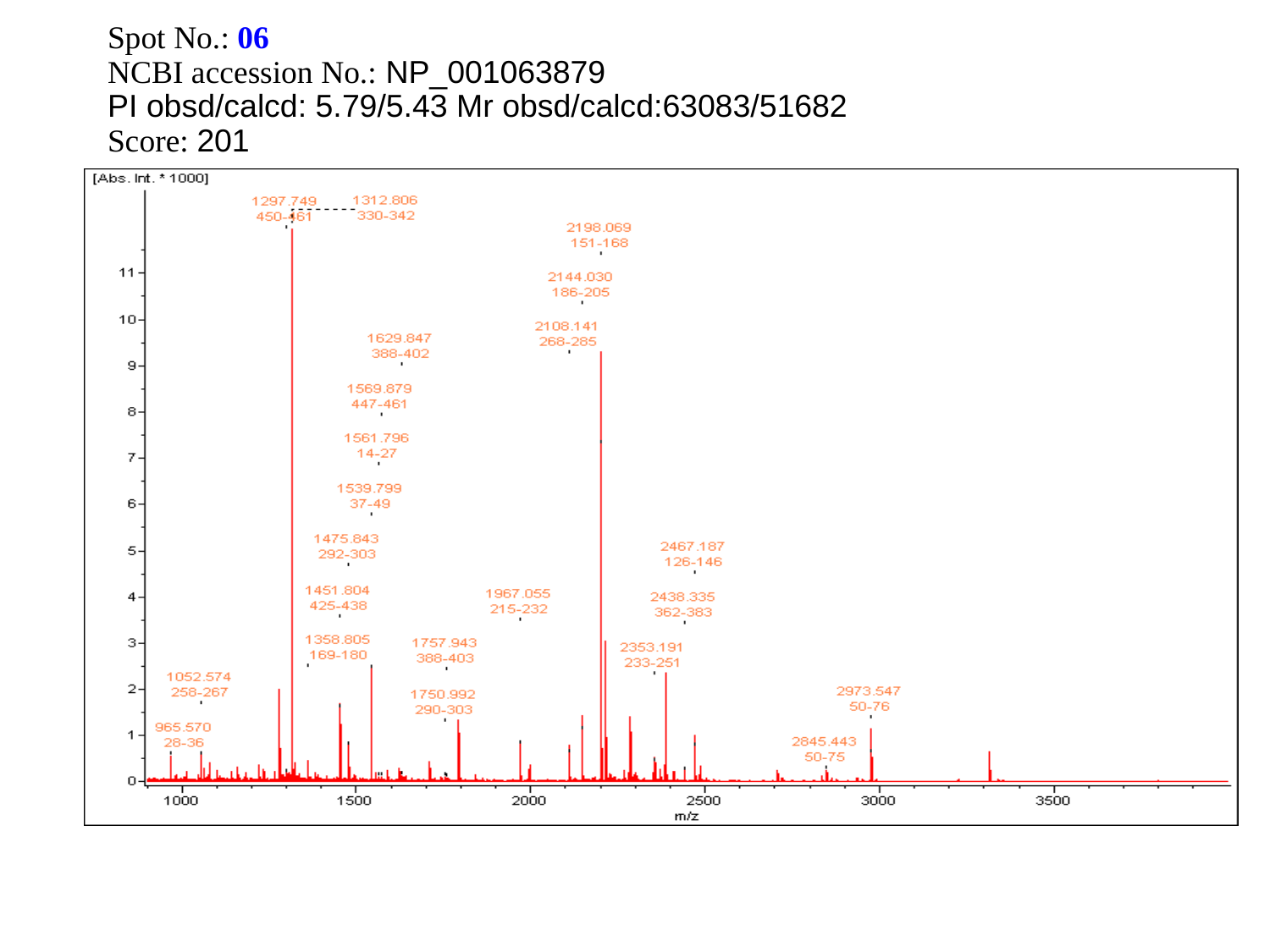

Spot No.: 06
NCBI accession No.: NP_001063879
PI obsd/calcd: 5.79/5.43 Mr obsd/calcd:63083/51682
Score: 201

## Slide 18
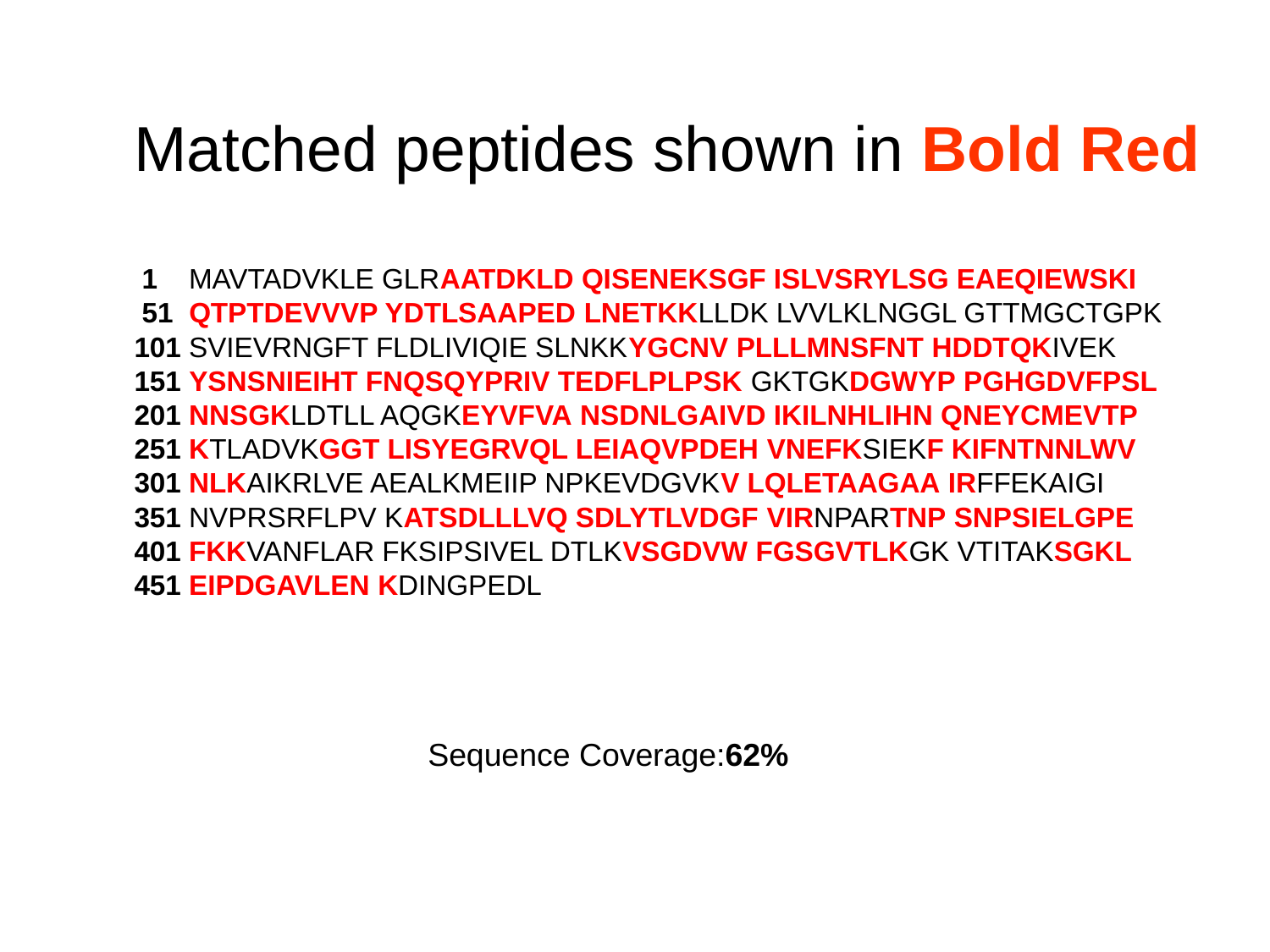

Matched peptides shown in Bold Red
 1 MAVTADVKLE GLRAATDKLD QISENEKSGF ISLVSRYLSG EAEQIEWSKI
 51 QTPTDEVVVP YDTLSAAPED LNETKKLLDK LVVLKLNGGL GTTMGCTGPK
101 SVIEVRNGFT FLDLIVIQIE SLNKKYGCNV PLLLMNSFNT HDDTQKIVEK
151 YSNSNIEIHT FNQSQYPRIV TEDFLPLPSK GKTGKDGWYP PGHGDVFPSL
201 NNSGKLDTLL AQGKEYVFVA NSDNLGAIVD IKILNHLIHN QNEYCMEVTP
251 KTLADVKGGT LISYEGRVQL LEIAQVPDEH VNEFKSIEKF KIFNTNNLWV
301 NLKAIKRLVE AEALKMEIIP NPKEVDGVKV LQLETAAGAA IRFFEKAIGI
351 NVPRSRFLPV KATSDLLLVQ SDLYTLVDGF VIRNPARTNP SNPSIELGPE
401 FKKVANFLAR FKSIPSIVEL DTLKVSGDVW FGSGVTLKGK VTITAKSGKL
451 EIPDGAVLEN KDINGPEDL
# Sequence Coverage:62%

## Slide 19
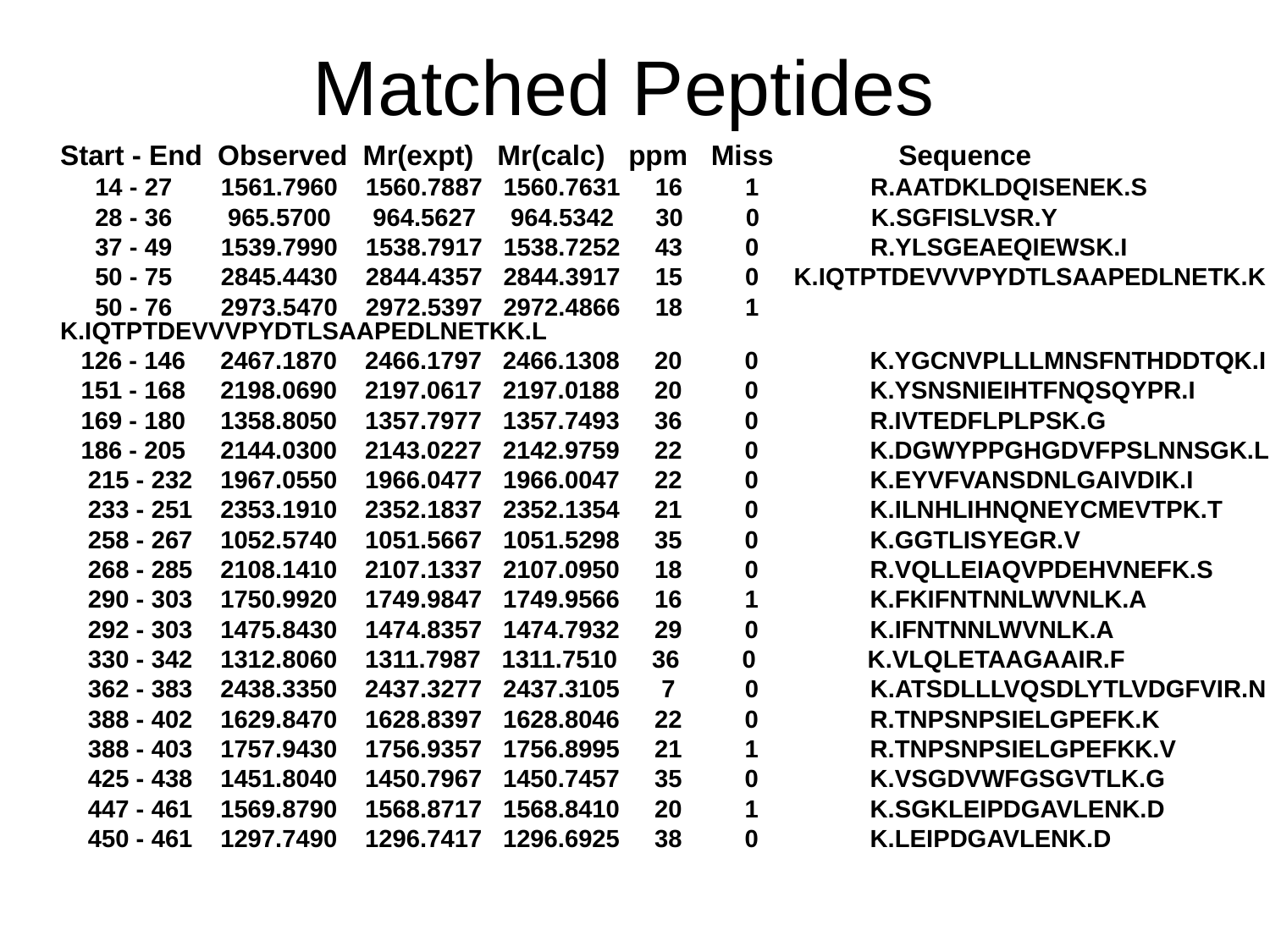

# Matched Peptides
Start - End Observed Mr(expt) Mr(calc) ppm Miss Sequence
 14 - 27 1561.7960 1560.7887 1560.7631 16 1 R.AATDKLDQISENEK.S
 28 - 36 965.5700 964.5627 964.5342 30 0 K.SGFISLVSR.Y
 37 - 49 1539.7990 1538.7917 1538.7252 43 0 R.YLSGEAEQIEWSK.I
 50 - 75 2845.4430 2844.4357 2844.3917 15 0 K.IQTPTDEVVVPYDTLSAAPEDLNETK.K
 50 - 76 2973.5470 2972.5397 2972.4866 18 1 K.IQTPTDEVVVPYDTLSAAPEDLNETKK.L
 126 - 146 2467.1870 2466.1797 2466.1308 20 0 K.YGCNVPLLLMNSFNTHDDTQK.I
 151 - 168 2198.0690 2197.0617 2197.0188 20 0 K.YSNSNIEIHTFNQSQYPR.I
 169 - 180 1358.8050 1357.7977 1357.7493 36 0 R.IVTEDFLPLPSK.G
 186 - 205 2144.0300 2143.0227 2142.9759 22 0 K.DGWYPPGHGDVFPSLNNSGK.L
 215 - 232 1967.0550 1966.0477 1966.0047 22 0 K.EYVFVANSDNLGAIVDIK.I
 233 - 251 2353.1910 2352.1837 2352.1354 21 0 K.ILNHLIHNQNEYCMEVTPK.T
 258 - 267 1052.5740 1051.5667 1051.5298 35 0 K.GGTLISYEGR.V
 268 - 285 2108.1410 2107.1337 2107.0950 18 0 R.VQLLEIAQVPDEHVNEFK.S
 290 - 303 1750.9920 1749.9847 1749.9566 16 1 K.FKIFNTNNLWVNLK.A
 292 - 303 1475.8430 1474.8357 1474.7932 29 0 K.IFNTNNLWVNLK.A
 330 - 342 1312.8060 1311.7987 1311.7510 36 0 K.VLQLETAAGAAIR.F
 362 - 383 2438.3350 2437.3277 2437.3105 7 0 K.ATSDLLLVQSDLYTLVDGFVIR.N
 388 - 402 1629.8470 1628.8397 1628.8046 22 0 R.TNPSNPSIELGPEFK.K
 388 - 403 1757.9430 1756.9357 1756.8995 21 1 R.TNPSNPSIELGPEFKK.V
 425 - 438 1451.8040 1450.7967 1450.7457 35 0 K.VSGDVWFGSGVTLK.G
 447 - 461 1569.8790 1568.8717 1568.8410 20 1 K.SGKLEIPDGAVLENK.D
 450 - 461 1297.7490 1296.7417 1296.6925 38 0 K.LEIPDGAVLENK.D

## Slide 20
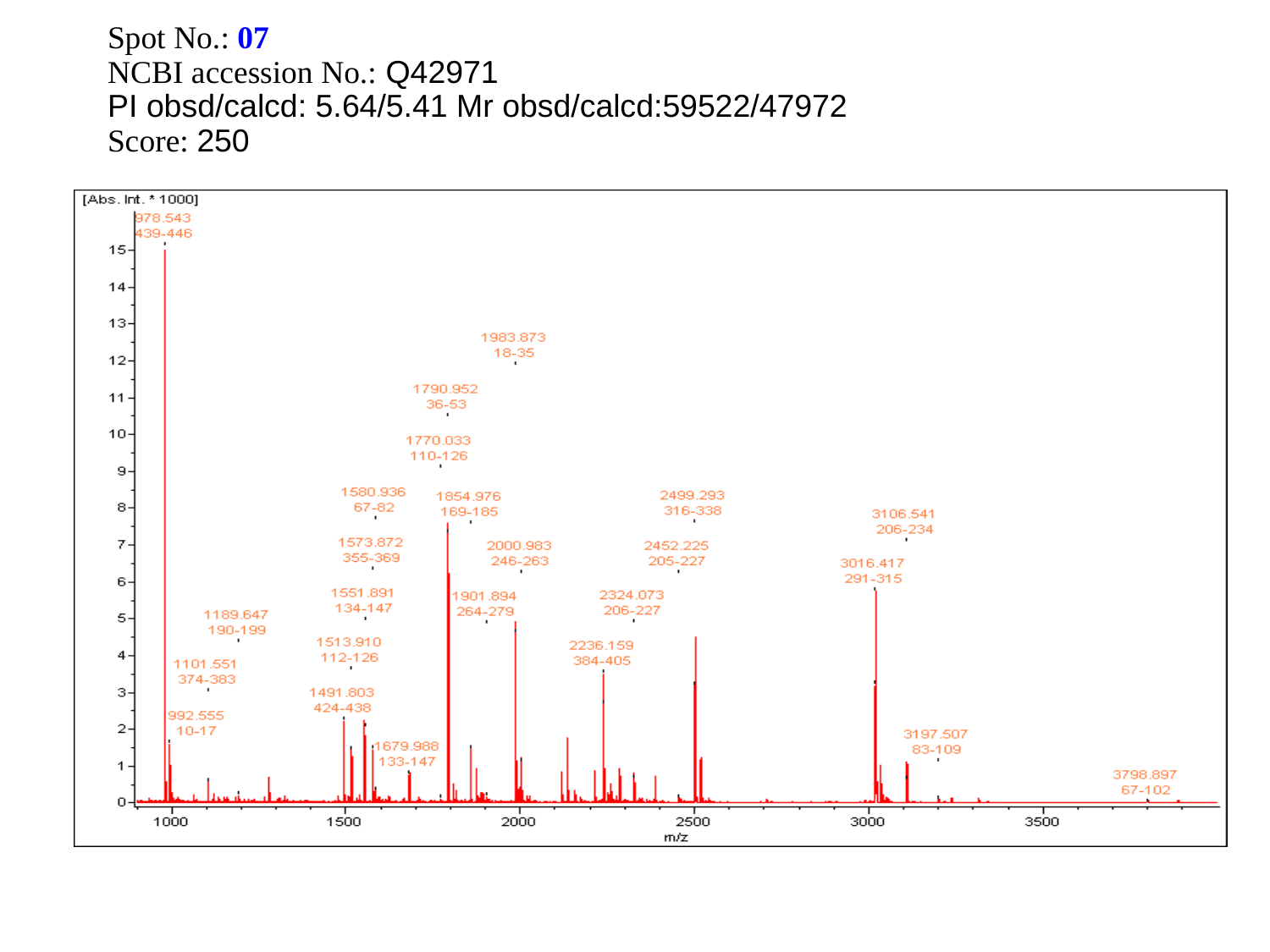

Spot No.: 07
NCBI accession No.: Q42971
PI obsd/calcd: 5.64/5.41 Mr obsd/calcd:59522/47972
Score: 250

## Slide 21
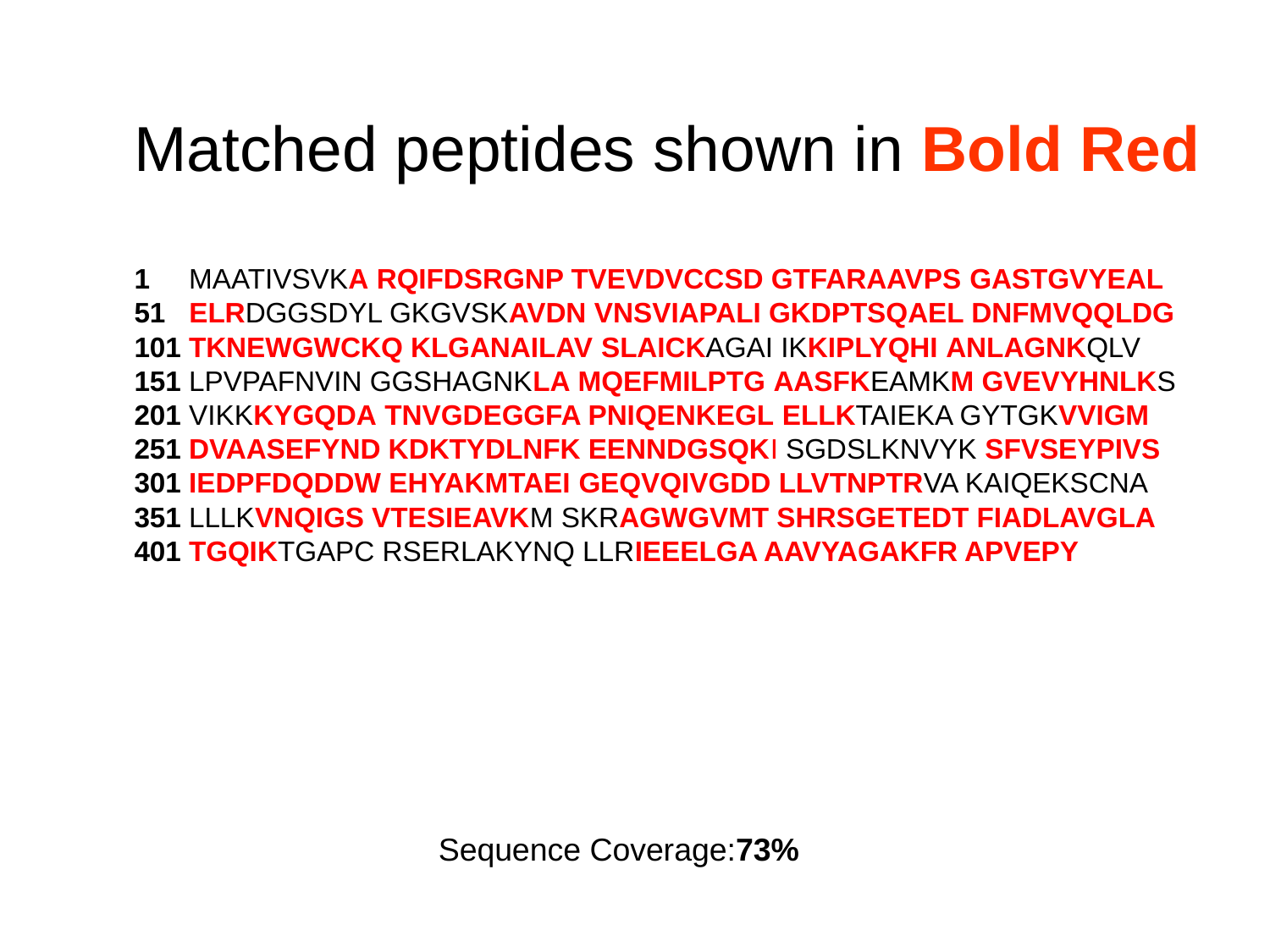

Matched peptides shown in Bold Red
1 MAATIVSVKA RQIFDSRGNP TVEVDVCCSD GTFARAAVPS GASTGVYEAL
51 ELRDGGSDYL GKGVSKAVDN VNSVIAPALI GKDPTSQAEL DNFMVQQLDG
101 TKNEWGWCKQ KLGANAILAV SLAICKAGAI IKKIPLYQHI ANLAGNKQLV
151 LPVPAFNVIN GGSHAGNKLA MQEFMILPTG AASFKEAMKM GVEVYHNLKS
201 VIKKKYGQDA TNVGDEGGFA PNIQENKEGL ELLKTAIEKA GYTGKVVIGM
251 DVAASEFYND KDKTYDLNFK EENNDGSQKI SGDSLKNVYK SFVSEYPIVS
301 IEDPFDQDDW EHYAKMTAEI GEQVQIVGDD LLVTNPTRVA KAIQEKSCNA
351 LLLKVNQIGS VTESIEAVKM SKRAGWGVMT SHRSGETEDT FIADLAVGLA
401 TGQIKTGAPC RSERLAKYNQ LLRIEEELGA AAVYAGAKFR APVEPY
# Sequence Coverage:73%

## Slide 22
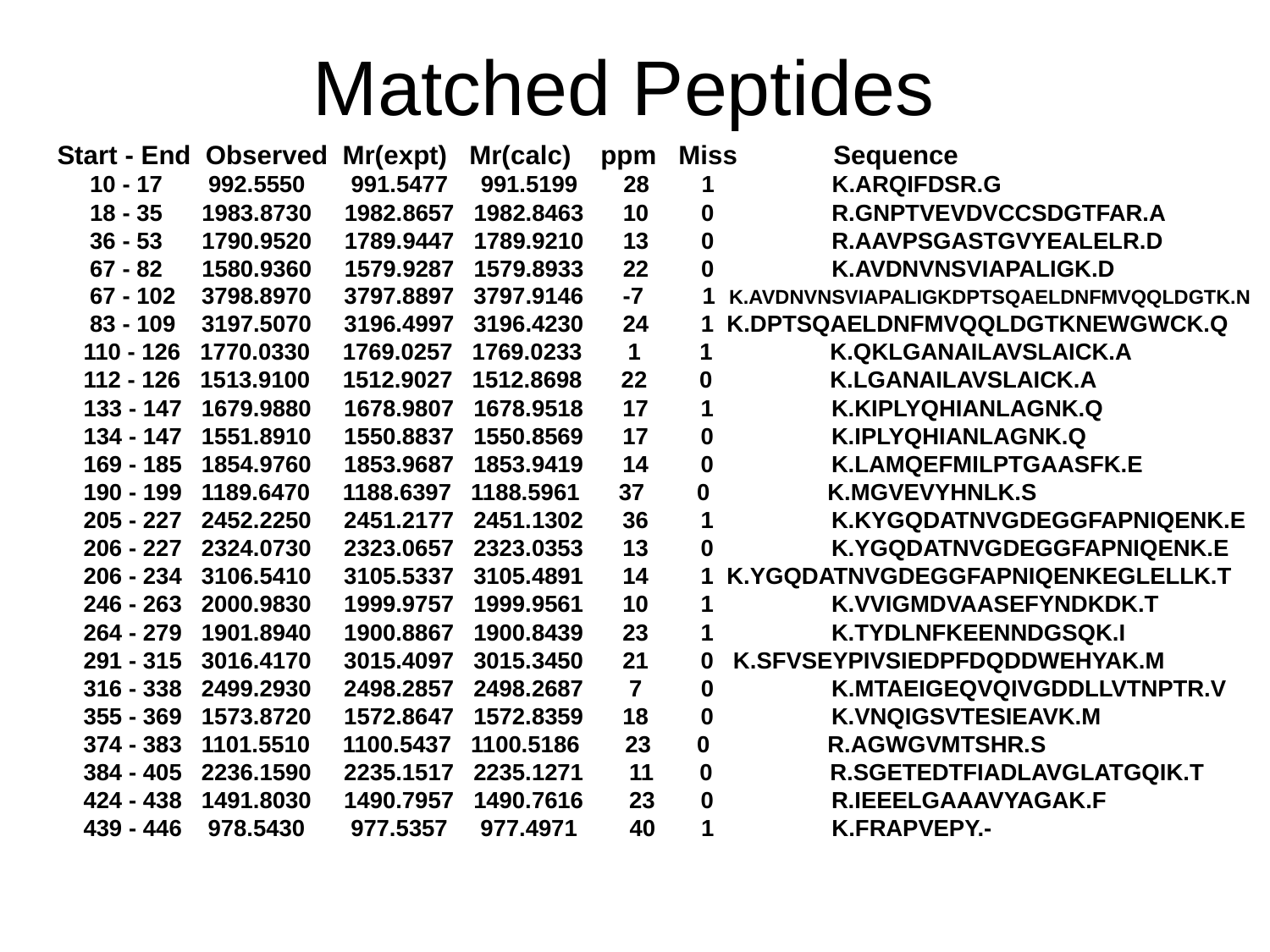

# Matched Peptides
Start - End Observed Mr(expt) Mr(calc) ppm Miss Sequence
 10 - 17 992.5550 991.5477 991.5199 28 1 K.ARQIFDSR.G
 18 - 35 1983.8730 1982.8657 1982.8463 10 0 R.GNPTVEVDVCCSDGTFAR.A
 36 - 53 1790.9520 1789.9447 1789.9210 13 0 R.AAVPSGASTGVYEALELR.D
 67 - 82 1580.9360 1579.9287 1579.8933 22 0 K.AVDNVNSVIAPALIGK.D
 67 - 102 3798.8970 3797.8897 3797.9146 -7 1 K.AVDNVNSVIAPALIGKDPTSQAELDNFMVQQLDGTK.N
 83 - 109 3197.5070 3196.4997 3196.4230 24 1 K.DPTSQAELDNFMVQQLDGTKNEWGWCK.Q
 110 - 126 1770.0330 1769.0257 1769.0233 1 1 K.QKLGANAILAVSLAICK.A
 112 - 126 1513.9100 1512.9027 1512.8698 22 0 K.LGANAILAVSLAICK.A
 133 - 147 1679.9880 1678.9807 1678.9518 17 1 K.KIPLYQHIANLAGNK.Q
 134 - 147 1551.8910 1550.8837 1550.8569 17 0 K.IPLYQHIANLAGNK.Q
 169 - 185 1854.9760 1853.9687 1853.9419 14 0 K.LAMQEFMILPTGAASFK.E
 190 - 199 1189.6470 1188.6397 1188.5961 37 0 K.MGVEVYHNLK.S
 205 - 227 2452.2250 2451.2177 2451.1302 36 1 K.KYGQDATNVGDEGGFAPNIQENK.E
 206 - 227 2324.0730 2323.0657 2323.0353 13 0 K.YGQDATNVGDEGGFAPNIQENK.E
 206 - 234 3106.5410 3105.5337 3105.4891 14 1 K.YGQDATNVGDEGGFAPNIQENKEGLELLK.T
 246 - 263 2000.9830 1999.9757 1999.9561 10 1 K.VVIGMDVAASEFYNDKDK.T
 264 - 279 1901.8940 1900.8867 1900.8439 23 1 K.TYDLNFKEENNDGSQK.I
 291 - 315 3016.4170 3015.4097 3015.3450 21 0 K.SFVSEYPIVSIEDPFDQDDWEHYAK.M
 316 - 338 2499.2930 2498.2857 2498.2687 7 0 K.MTAEIGEQVQIVGDDLLVTNPTR.V
 355 - 369 1573.8720 1572.8647 1572.8359 18 0 K.VNQIGSVTESIEAVK.M
 374 - 383 1101.5510 1100.5437 1100.5186 23 0 R.AGWGVMTSHR.S
 384 - 405 2236.1590 2235.1517 2235.1271 11 0 R.SGETEDTFIADLAVGLATGQIK.T
 424 - 438 1491.8030 1490.7957 1490.7616 23 0 R.IEEELGAAAVYAGAK.F
 439 - 446 978.5430 977.5357 977.4971 40 1 K.FRAPVEPY.-

## Slide 23
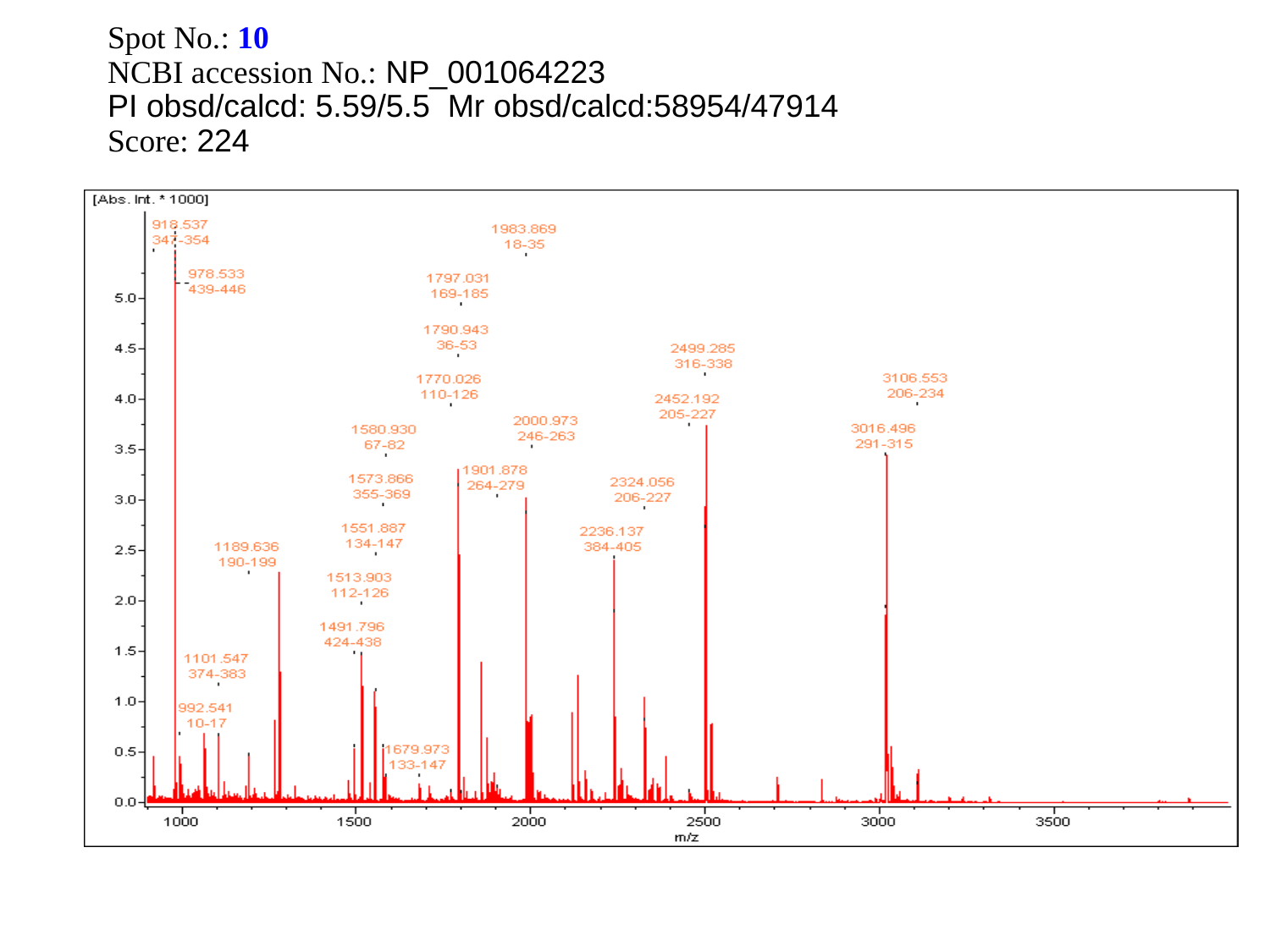

Spot No.: 10
NCBI accession No.: NP_001064223
PI obsd/calcd: 5.59/5.5 Mr obsd/calcd:58954/47914
Score: 224

## Slide 24
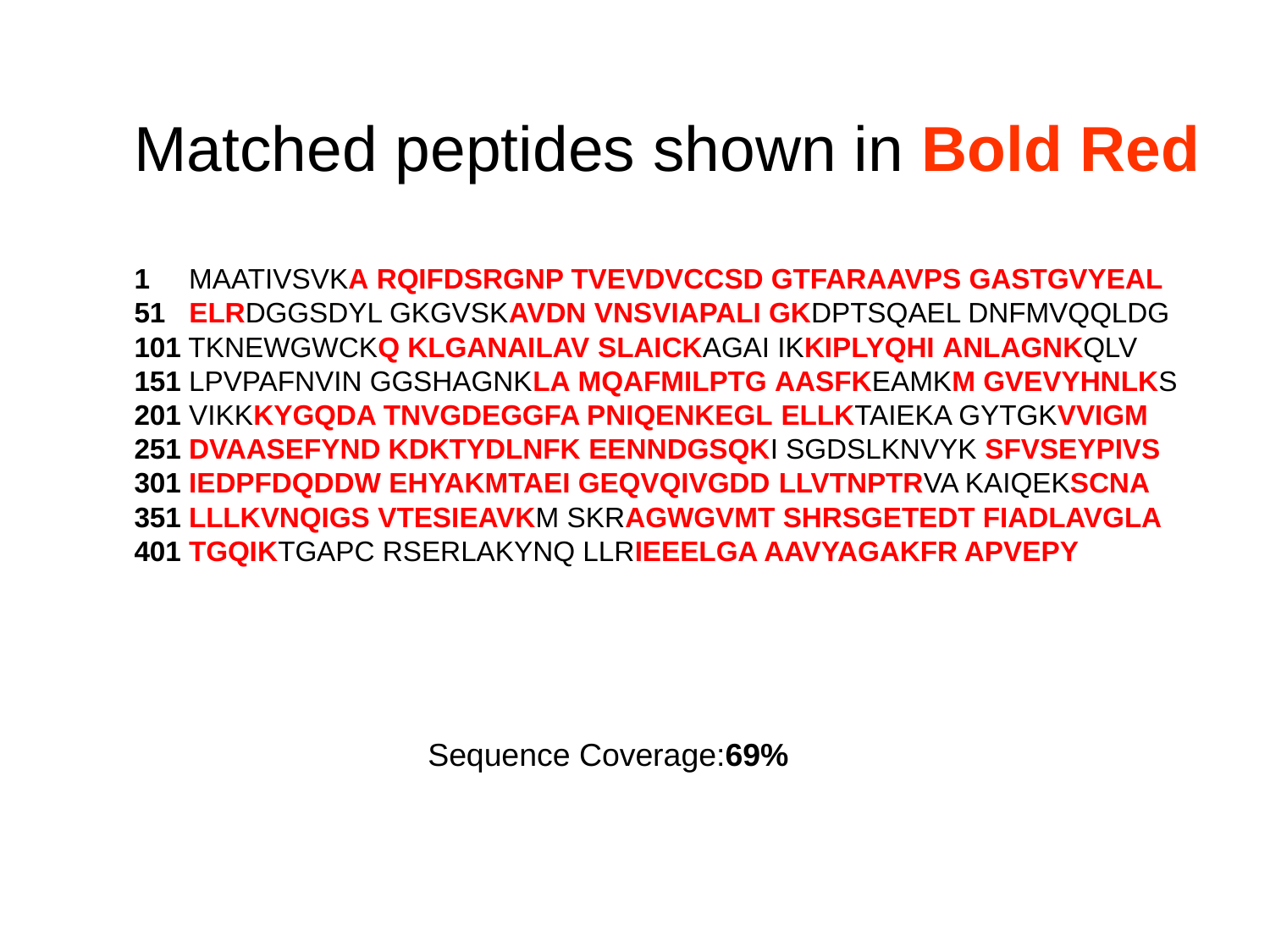

Matched peptides shown in Bold Red
1 MAATIVSVKA RQIFDSRGNP TVEVDVCCSD GTFARAAVPS GASTGVYEAL
51 ELRDGGSDYL GKGVSKAVDN VNSVIAPALI GKDPTSQAEL DNFMVQQLDG
101 TKNEWGWCKQ KLGANAILAV SLAICKAGAI IKKIPLYQHI ANLAGNKQLV
151 LPVPAFNVIN GGSHAGNKLA MQAFMILPTG AASFKEAMKM GVEVYHNLKS
201 VIKKKYGQDA TNVGDEGGFA PNIQENKEGL ELLKTAIEKA GYTGKVVIGM
251 DVAASEFYND KDKTYDLNFK EENNDGSQKI SGDSLKNVYK SFVSEYPIVS
301 IEDPFDQDDW EHYAKMTAEI GEQVQIVGDD LLVTNPTRVA KAIQEKSCNA
351 LLLKVNQIGS VTESIEAVKM SKRAGWGVMT SHRSGETEDT FIADLAVGLA
401 TGQIKTGAPC RSERLAKYNQ LLRIEEELGA AAVYAGAKFR APVEPY
# Sequence Coverage:69%

## Slide 25
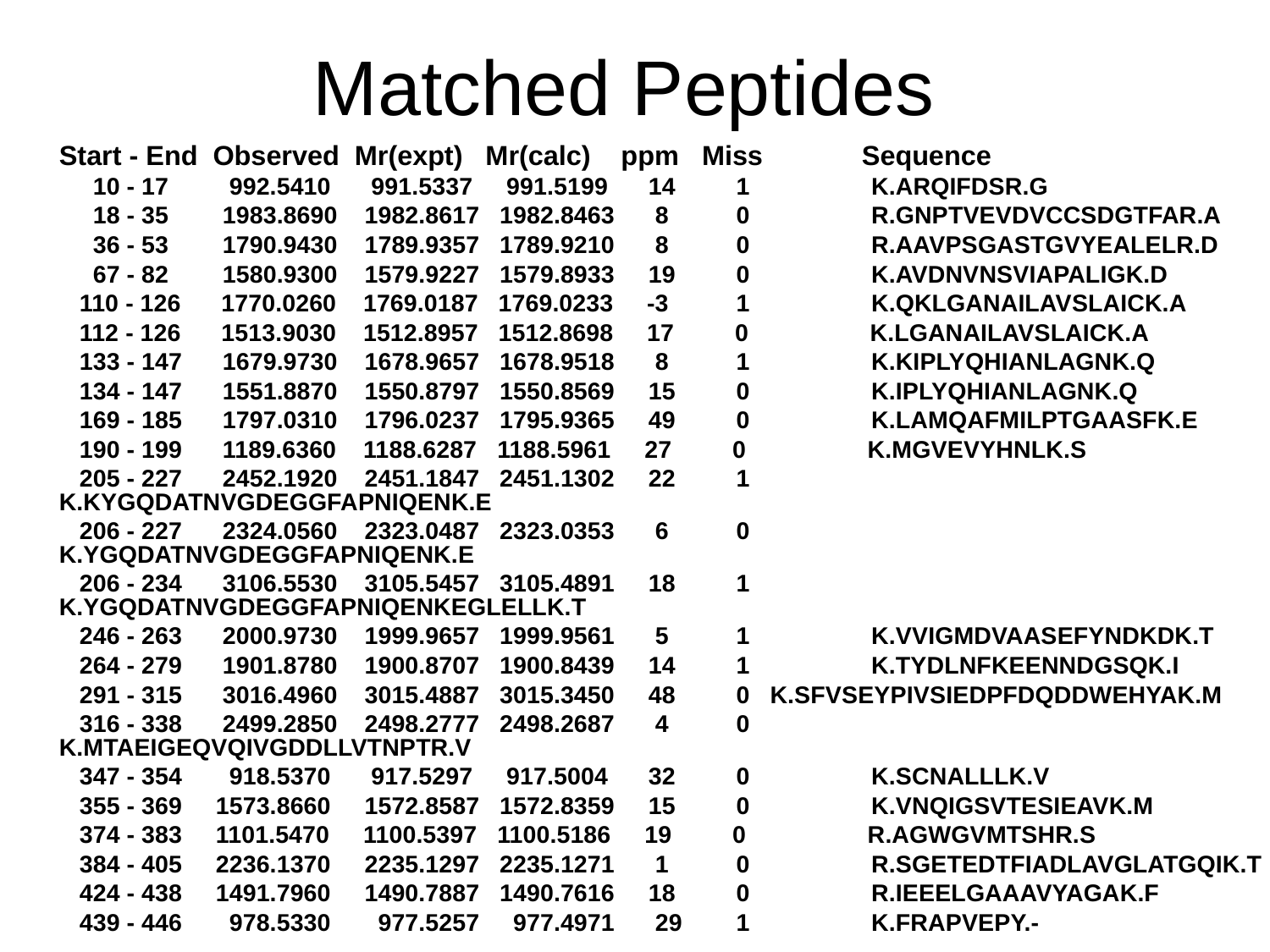

# Matched Peptides
Start - End Observed Mr(expt) Mr(calc) ppm Miss Sequence
 10 - 17 992.5410 991.5337 991.5199 14 1 K.ARQIFDSR.G
 18 - 35 1983.8690 1982.8617 1982.8463 8 0 R.GNPTVEVDVCCSDGTFAR.A
 36 - 53 1790.9430 1789.9357 1789.9210 8 0 R.AAVPSGASTGVYEALELR.D
 67 - 82 1580.9300 1579.9227 1579.8933 19 0 K.AVDNVNSVIAPALIGK.D
 110 - 126 1770.0260 1769.0187 1769.0233 -3 1 K.QKLGANAILAVSLAICK.A
 112 - 126 1513.9030 1512.8957 1512.8698 17 0 K.LGANAILAVSLAICK.A
 133 - 147 1679.9730 1678.9657 1678.9518 8 1 K.KIPLYQHIANLAGNK.Q
 134 - 147 1551.8870 1550.8797 1550.8569 15 0 K.IPLYQHIANLAGNK.Q
 169 - 185 1797.0310 1796.0237 1795.9365 49 0 K.LAMQAFMILPTGAASFK.E
 190 - 199 1189.6360 1188.6287 1188.5961 27 0 K.MGVEVYHNLK.S
 205 - 227 2452.1920 2451.1847 2451.1302 22 1 K.KYGQDATNVGDEGGFAPNIQENK.E
 206 - 227 2324.0560 2323.0487 2323.0353 6 0 K.YGQDATNVGDEGGFAPNIQENK.E
 206 - 234 3106.5530 3105.5457 3105.4891 18 1 K.YGQDATNVGDEGGFAPNIQENKEGLELLK.T
 246 - 263 2000.9730 1999.9657 1999.9561 5 1 K.VVIGMDVAASEFYNDKDK.T
 264 - 279 1901.8780 1900.8707 1900.8439 14 1 K.TYDLNFKEENNDGSQK.I
 291 - 315 3016.4960 3015.4887 3015.3450 48 0 K.SFVSEYPIVSIEDPFDQDDWEHYAK.M
 316 - 338 2499.2850 2498.2777 2498.2687 4 0 K.MTAEIGEQVQIVGDDLLVTNPTR.V
 347 - 354 918.5370 917.5297 917.5004 32 0 K.SCNALLLK.V
 355 - 369 1573.8660 1572.8587 1572.8359 15 0 K.VNQIGSVTESIEAVK.M
 374 - 383 1101.5470 1100.5397 1100.5186 19 0 R.AGWGVMTSHR.S
 384 - 405 2236.1370 2235.1297 2235.1271 1 0 R.SGETEDTFIADLAVGLATGQIK.T
 424 - 438 1491.7960 1490.7887 1490.7616 18 0 R.IEEELGAAAVYAGAK.F
 439 - 446 978.5330 977.5257 977.4971 29 1 K.FRAPVEPY.-

## Slide 26
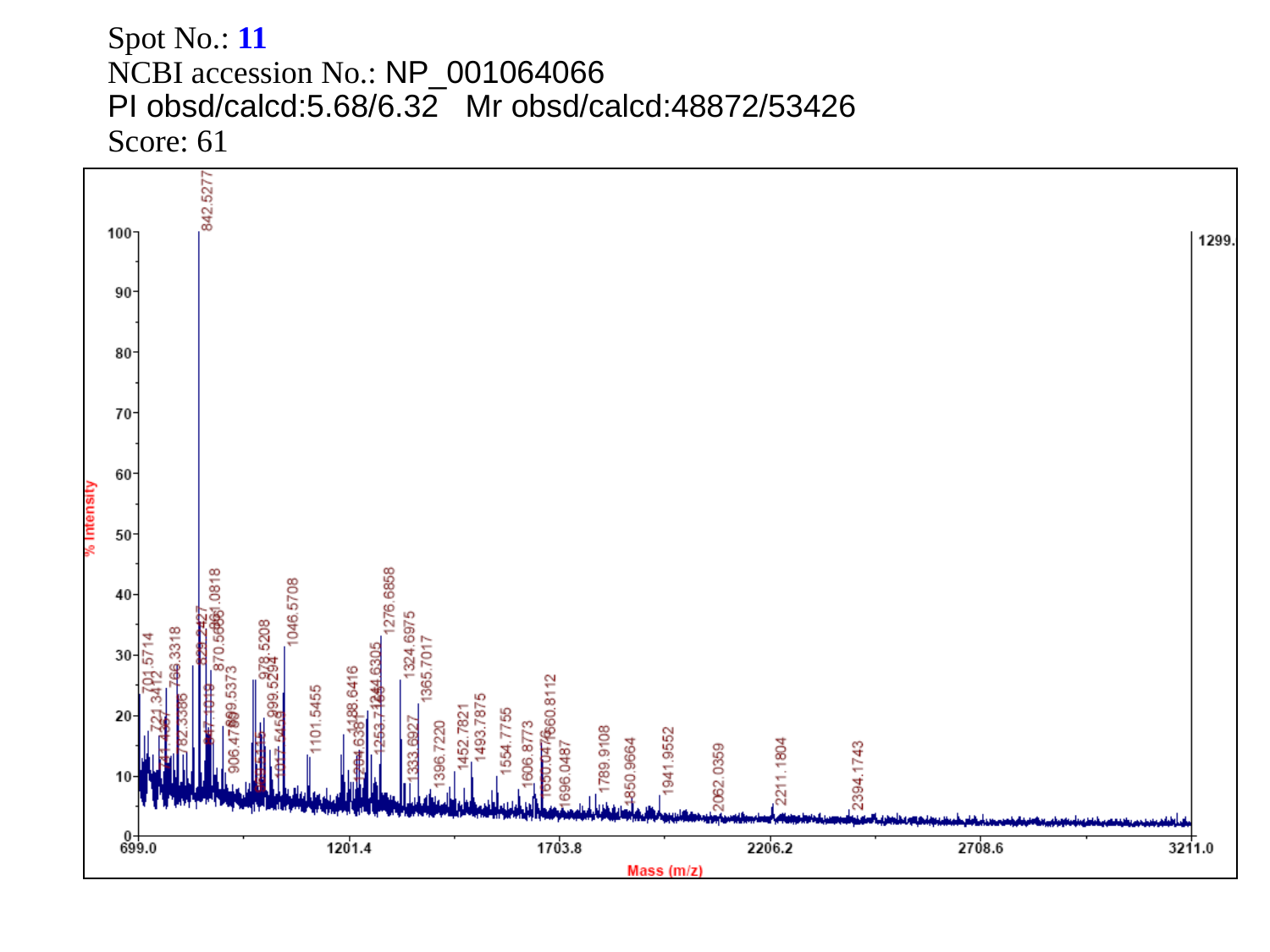

Spot No.: 11
NCBI accession No.: NP_001064066
PI obsd/calcd:5.68/6.32 Mr obsd/calcd:48872/53426
Score: 61

## Slide 27
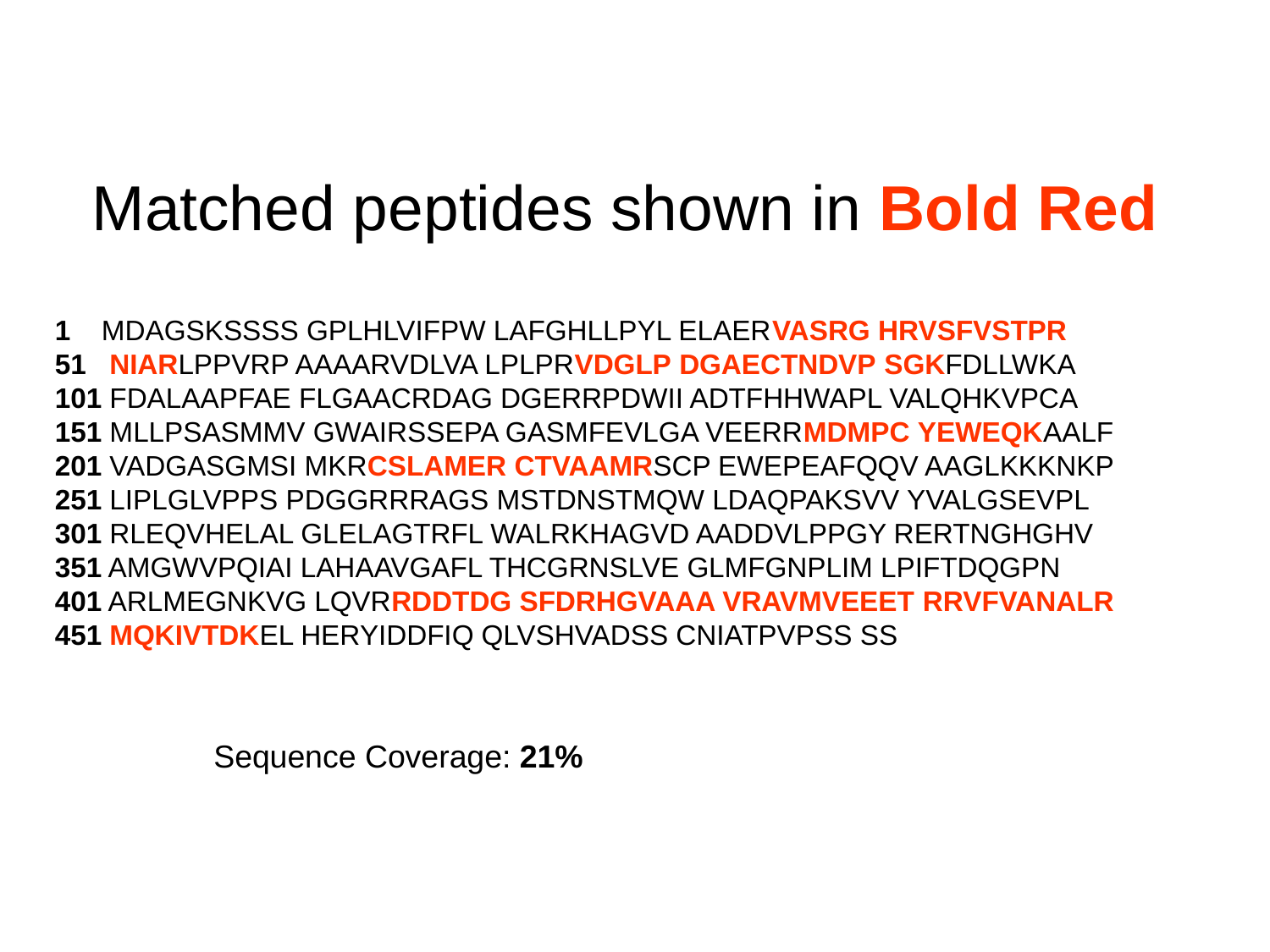

#
Matched peptides shown in Bold Red
1 MDAGSKSSSS GPLHLVIFPW LAFGHLLPYL ELAERVASRG HRVSFVSTPR
51 NIARLPPVRP AAAARVDLVA LPLPRVDGLP DGAECTNDVP SGKFDLLWKA
101 FDALAAPFAE FLGAACRDAG DGERRPDWII ADTFHHWAPL VALQHKVPCA
151 MLLPSASMMV GWAIRSSEPA GASMFEVLGA VEERRMDMPC YEWEQKAALF
201 VADGASGMSI MKRCSLAMER CTVAAMRSCP EWEPEAFQQV AAGLKKKNKP
251 LIPLGLVPPS PDGGRRRAGS MSTDNSTMQW LDAQPAKSVV YVALGSEVPL
301 RLEQVHELAL GLELAGTRFL WALRKHAGVD AADDVLPPGY RERTNGHGHV
351 AMGWVPQIAI LAHAAVGAFL THCGRNSLVE GLMFGNPLIM LPIFTDQGPN
401 ARLMEGNKVG LQVRRDDTDG SFDRHGVAAA VRAVMVEEET RRVFVANALR
451 MQKIVTDKEL HERYIDDFIQ QLVSHVADSS CNIATPVPSS SS
Sequence Coverage: 21%

## Slide 28
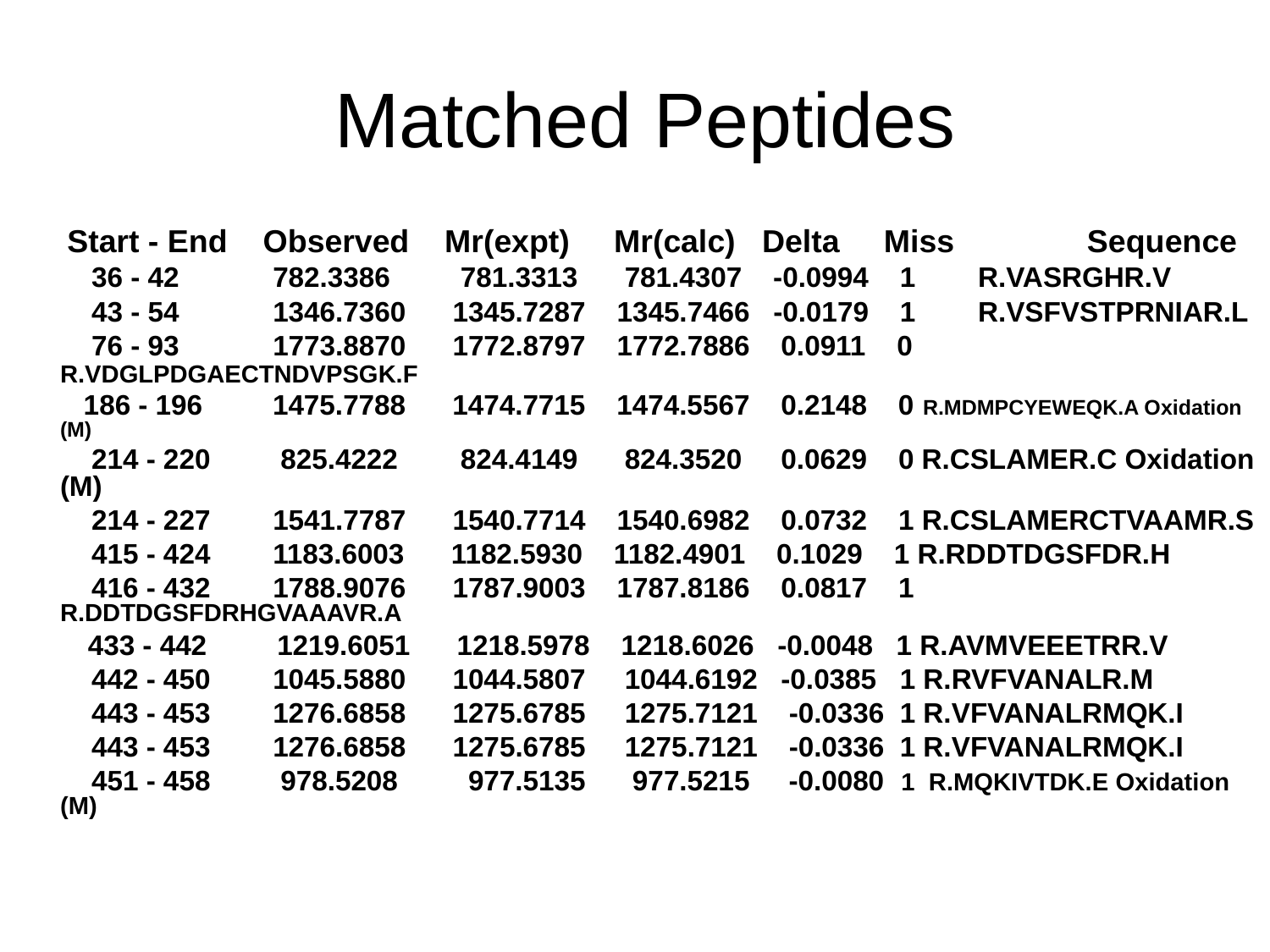

# Matched Peptides
 Start - End Observed Mr(expt) Mr(calc) Delta Miss Sequence
 36 - 42 782.3386 781.3313 781.4307 -0.0994 1 R.VASRGHR.V
 43 - 54 1346.7360 1345.7287 1345.7466 -0.0179 1 R.VSFVSTPRNIAR.L
 76 - 93 1773.8870 1772.8797 1772.7886 0.0911 0 R.VDGLPDGAECTNDVPSGK.F
 186 - 196 1475.7788 1474.7715 1474.5567 0.2148 0 R.MDMPCYEWEQK.A Oxidation (M)
 214 - 220 825.4222 824.4149 824.3520 0.0629 0 R.CSLAMER.C Oxidation (M)
 214 - 227 1541.7787 1540.7714 1540.6982 0.0732 1 R.CSLAMERCTVAAMR.S
 415 - 424 1183.6003 1182.5930 1182.4901 0.1029 1 R.RDDTDGSFDR.H
 416 - 432 1788.9076 1787.9003 1787.8186 0.0817 1 R.DDTDGSFDRHGVAAAVR.A
 433 - 442 1219.6051 1218.5978 1218.6026 -0.0048 1 R.AVMVEEETRR.V
 442 - 450 1045.5880 1044.5807 1044.6192 -0.0385 1 R.RVFVANALR.M
 443 - 453 1276.6858 1275.6785 1275.7121 -0.0336 1 R.VFVANALRMQK.I
 443 - 453 1276.6858 1275.6785 1275.7121 -0.0336 1 R.VFVANALRMQK.I
 451 - 458 978.5208 977.5135 977.5215 -0.0080 1 R.MQKIVTDK.E Oxidation (M)

## Slide 29
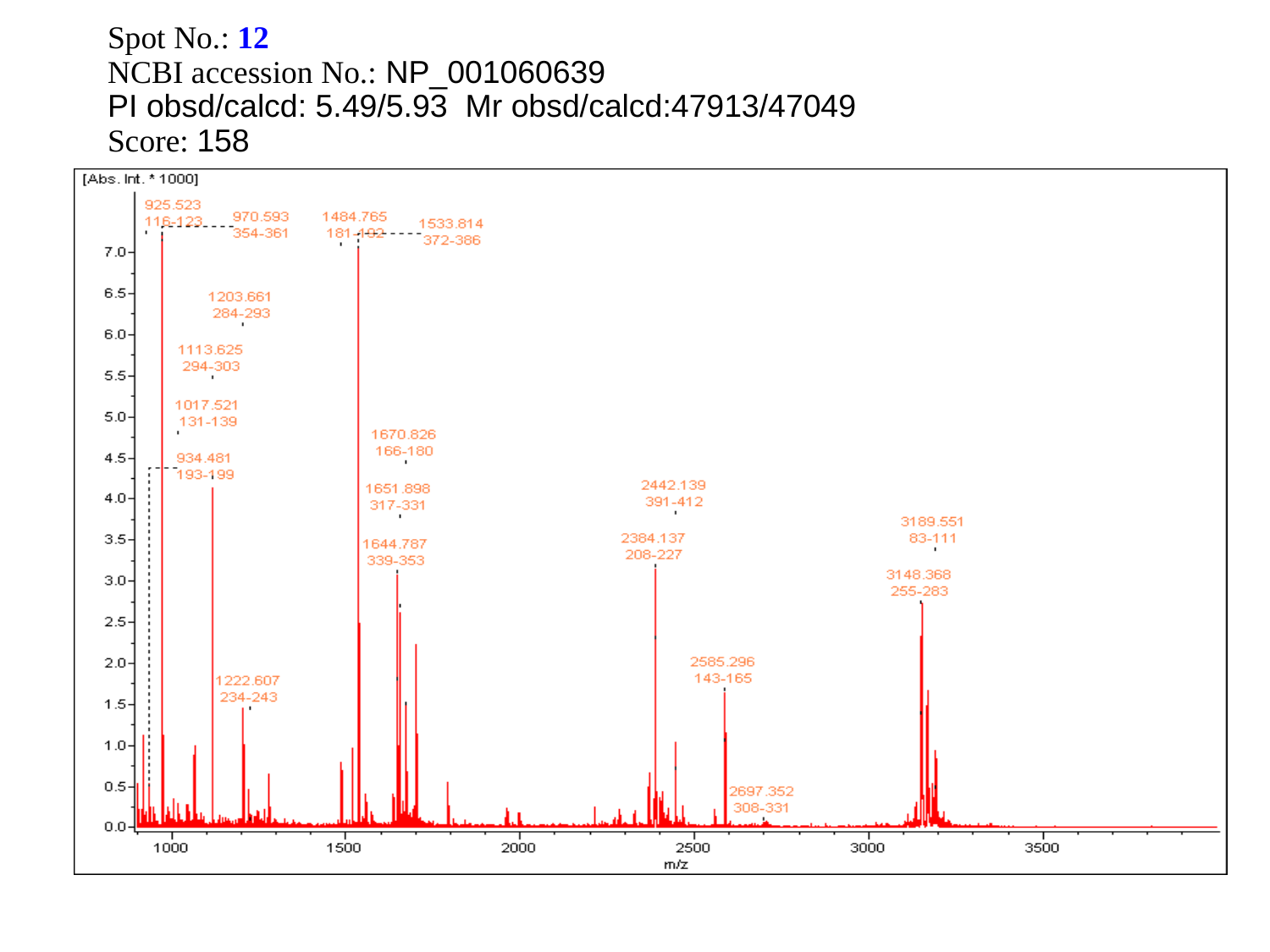

Spot No.: 12
NCBI accession No.: NP_001060639
PI obsd/calcd: 5.49/5.93 Mr obsd/calcd:47913/47049
Score: 158

## Slide 30
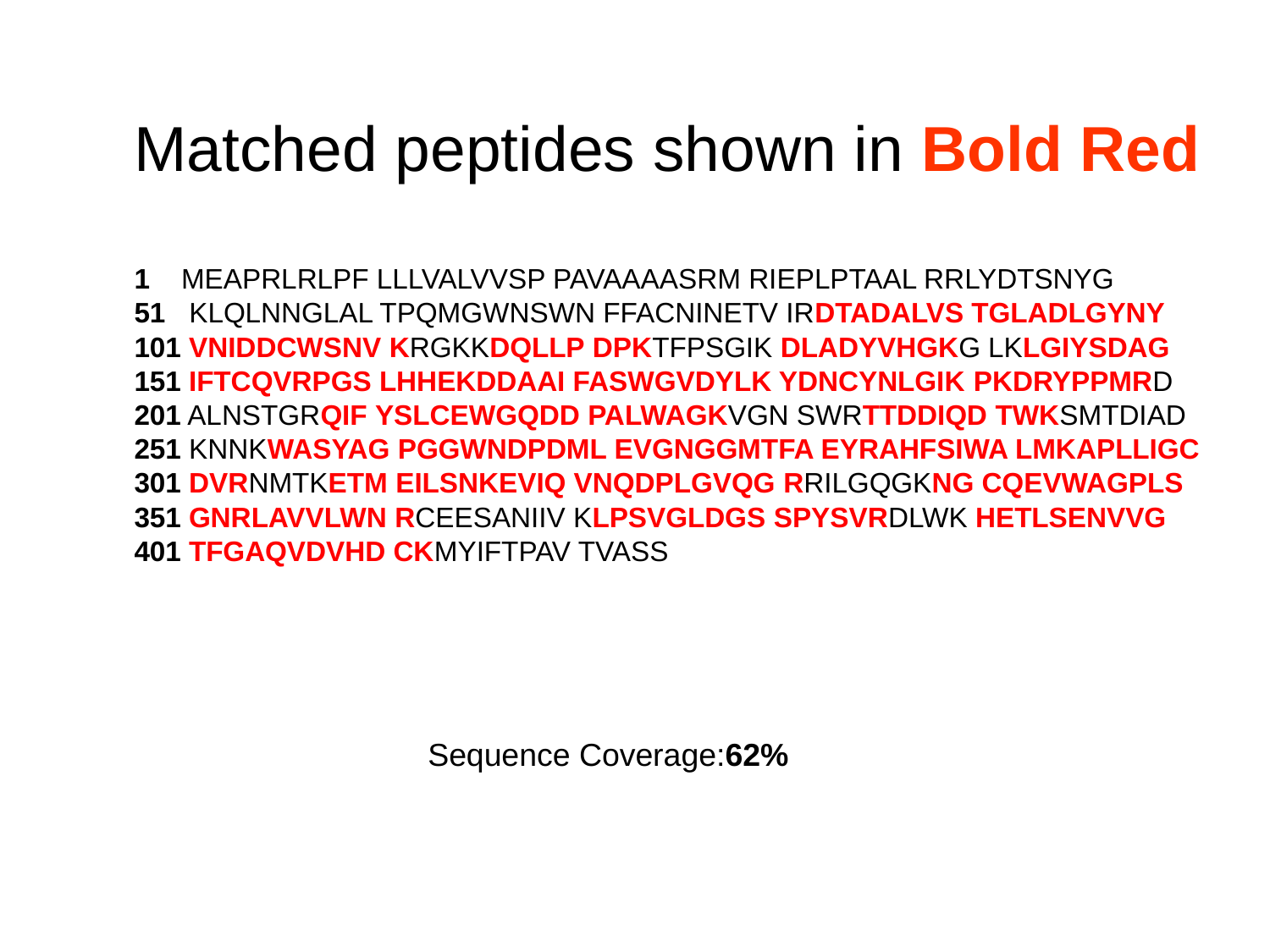

Matched peptides shown in Bold Red
1 MEAPRLRLPF LLLVALVVSP PAVAAAASRM RIEPLPTAAL RRLYDTSNYG
51 KLQLNNGLAL TPQMGWNSWN FFACNINETV IRDTADALVS TGLADLGYNY
101 VNIDDCWSNV KRGKKDQLLP DPKTFPSGIK DLADYVHGKG LKLGIYSDAG
151 IFTCQVRPGS LHHEKDDAAI FASWGVDYLK YDNCYNLGIK PKDRYPPMRD
201 ALNSTGRQIF YSLCEWGQDD PALWAGKVGN SWRTTDDIQD TWKSMTDIAD
251 KNNKWASYAG PGGWNDPDML EVGNGGMTFA EYRAHFSIWA LMKAPLLIGC
301 DVRNMTKETM EILSNKEVIQ VNQDPLGVQG RRILGQGKNG CQEVWAGPLS
351 GNRLAVVLWN RCEESANIIV KLPSVGLDGS SPYSVRDLWK HETLSENVVG
401 TFGAQVDVHD CKMYIFTPAV TVASS
# Sequence Coverage:62%

## Slide 31
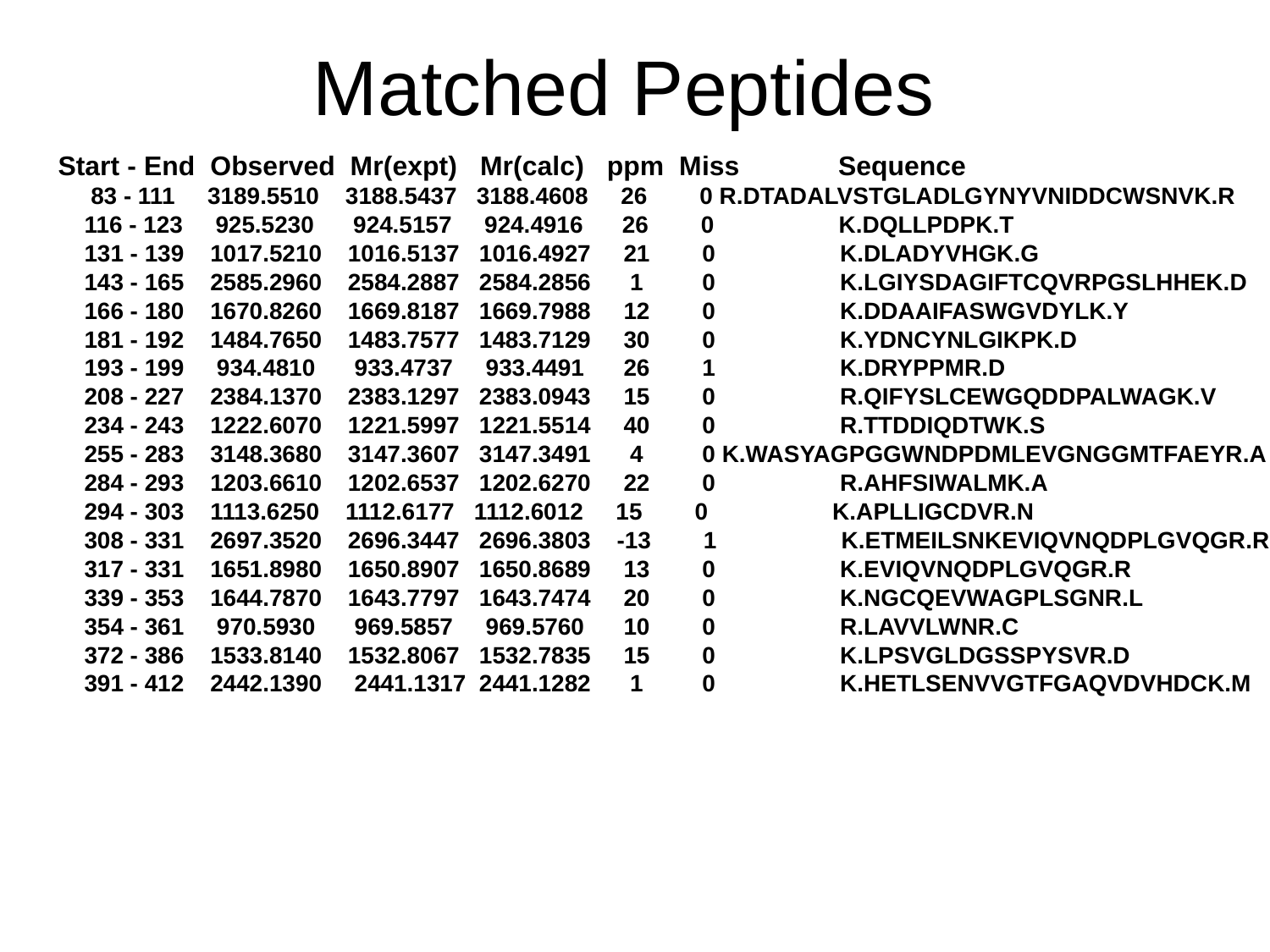

# Matched Peptides
Start - End Observed Mr(expt) Mr(calc) ppm Miss Sequence
 83 - 111 3189.5510 3188.5437 3188.4608 26 0 R.DTADALVSTGLADLGYNYVNIDDCWSNVK.R
 116 - 123 925.5230 924.5157 924.4916 26 0 K.DQLLPDPK.T
 131 - 139 1017.5210 1016.5137 1016.4927 21 0 K.DLADYVHGK.G
 143 - 165 2585.2960 2584.2887 2584.2856 1 0 K.LGIYSDAGIFTCQVRPGSLHHEK.D
 166 - 180 1670.8260 1669.8187 1669.7988 12 0 K.DDAAIFASWGVDYLK.Y
 181 - 192 1484.7650 1483.7577 1483.7129 30 0 K.YDNCYNLGIKPK.D
 193 - 199 934.4810 933.4737 933.4491 26 1 K.DRYPPMR.D
 208 - 227 2384.1370 2383.1297 2383.0943 15 0 R.QIFYSLCEWGQDDPALWAGK.V
 234 - 243 1222.6070 1221.5997 1221.5514 40 0 R.TTDDIQDTWK.S
 255 - 283 3148.3680 3147.3607 3147.3491 4 0 K.WASYAGPGGWNDPDMLEVGNGGMTFAEYR.A
 284 - 293 1203.6610 1202.6537 1202.6270 22 0 R.AHFSIWALMK.A
 294 - 303 1113.6250 1112.6177 1112.6012 15 0 K.APLLIGCDVR.N
 308 - 331 2697.3520 2696.3447 2696.3803 -13 1 K.ETMEILSNKEVIQVNQDPLGVQGR.R
 317 - 331 1651.8980 1650.8907 1650.8689 13 0 K.EVIQVNQDPLGVQGR.R
 339 - 353 1644.7870 1643.7797 1643.7474 20 0 K.NGCQEVWAGPLSGNR.L
 354 - 361 970.5930 969.5857 969.5760 10 0 R.LAVVLWNR.C
 372 - 386 1533.8140 1532.8067 1532.7835 15 0 K.LPSVGLDGSSPYSVR.D
 391 - 412 2442.1390 2441.1317 2441.1282 1 0 K.HETLSENVVGTFGAQVDVHDCK.M

## Slide 32
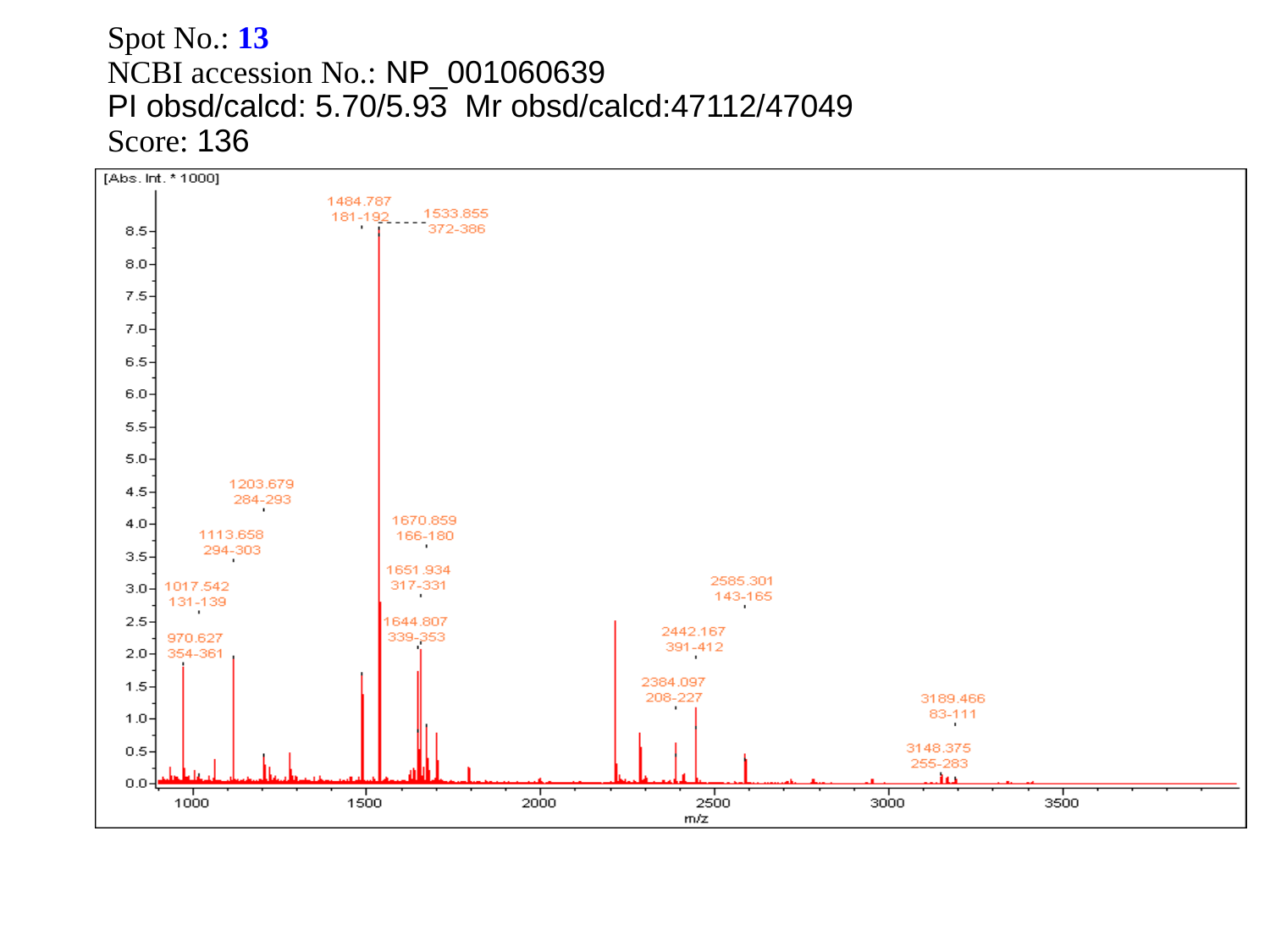

Spot No.: 13
NCBI accession No.: NP_001060639
PI obsd/calcd: 5.70/5.93 Mr obsd/calcd:47112/47049
Score: 136

## Slide 33
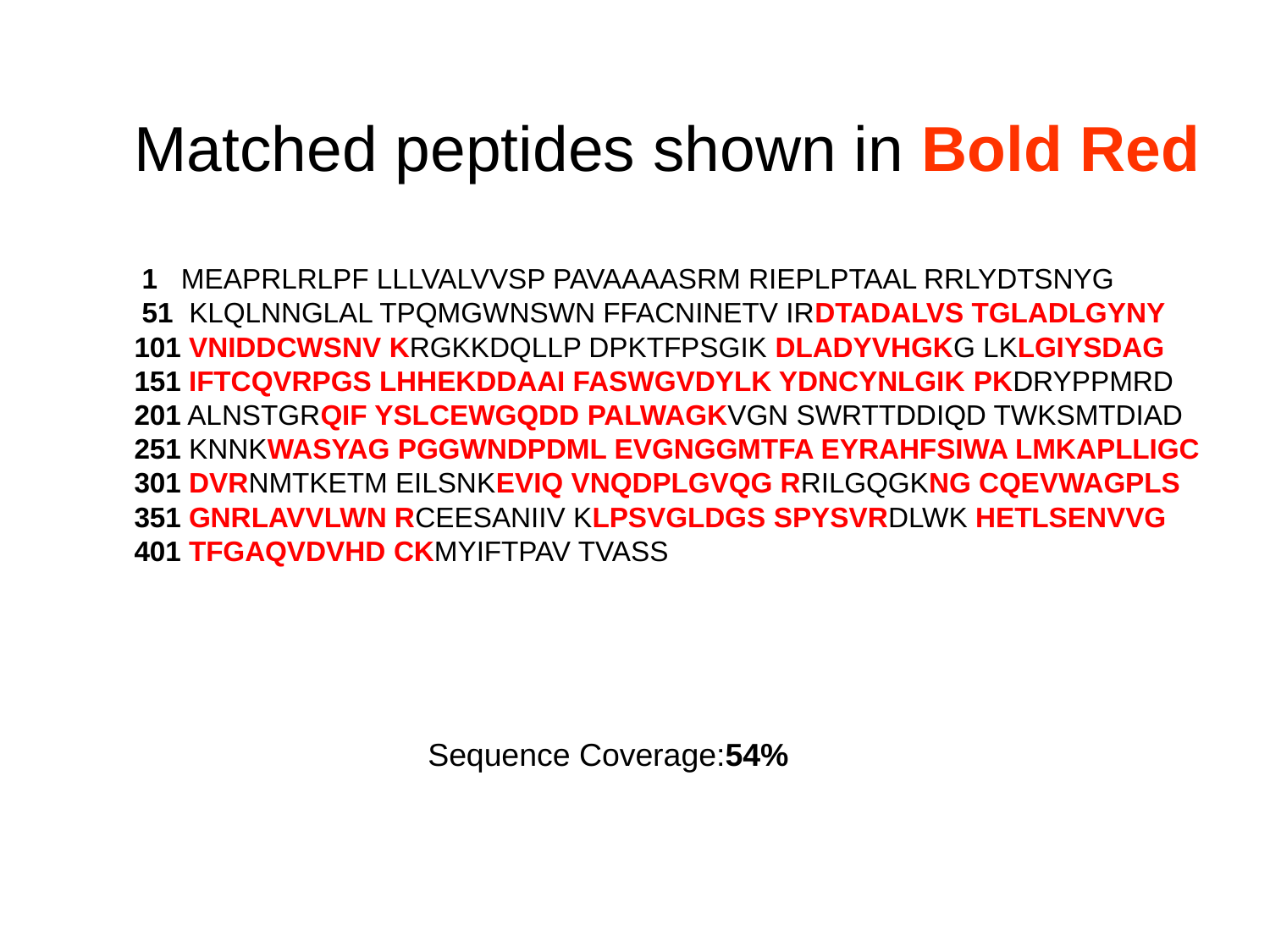

Matched peptides shown in Bold Red
 1 MEAPRLRLPF LLLVALVVSP PAVAAAASRM RIEPLPTAAL RRLYDTSNYG
 51 KLQLNNGLAL TPQMGWNSWN FFACNINETV IRDTADALVS TGLADLGYNY
101 VNIDDCWSNV KRGKKDQLLP DPKTFPSGIK DLADYVHGKG LKLGIYSDAG
151 IFTCQVRPGS LHHEKDDAAI FASWGVDYLK YDNCYNLGIK PKDRYPPMRD
201 ALNSTGRQIF YSLCEWGQDD PALWAGKVGN SWRTTDDIQD TWKSMTDIAD
251 KNNKWASYAG PGGWNDPDML EVGNGGMTFA EYRAHFSIWA LMKAPLLIGC
301 DVRNMTKETM EILSNKEVIQ VNQDPLGVQG RRILGQGKNG CQEVWAGPLS
351 GNRLAVVLWN RCEESANIIV KLPSVGLDGS SPYSVRDLWK HETLSENVVG
401 TFGAQVDVHD CKMYIFTPAV TVASS
# Sequence Coverage:54%

## Slide 34
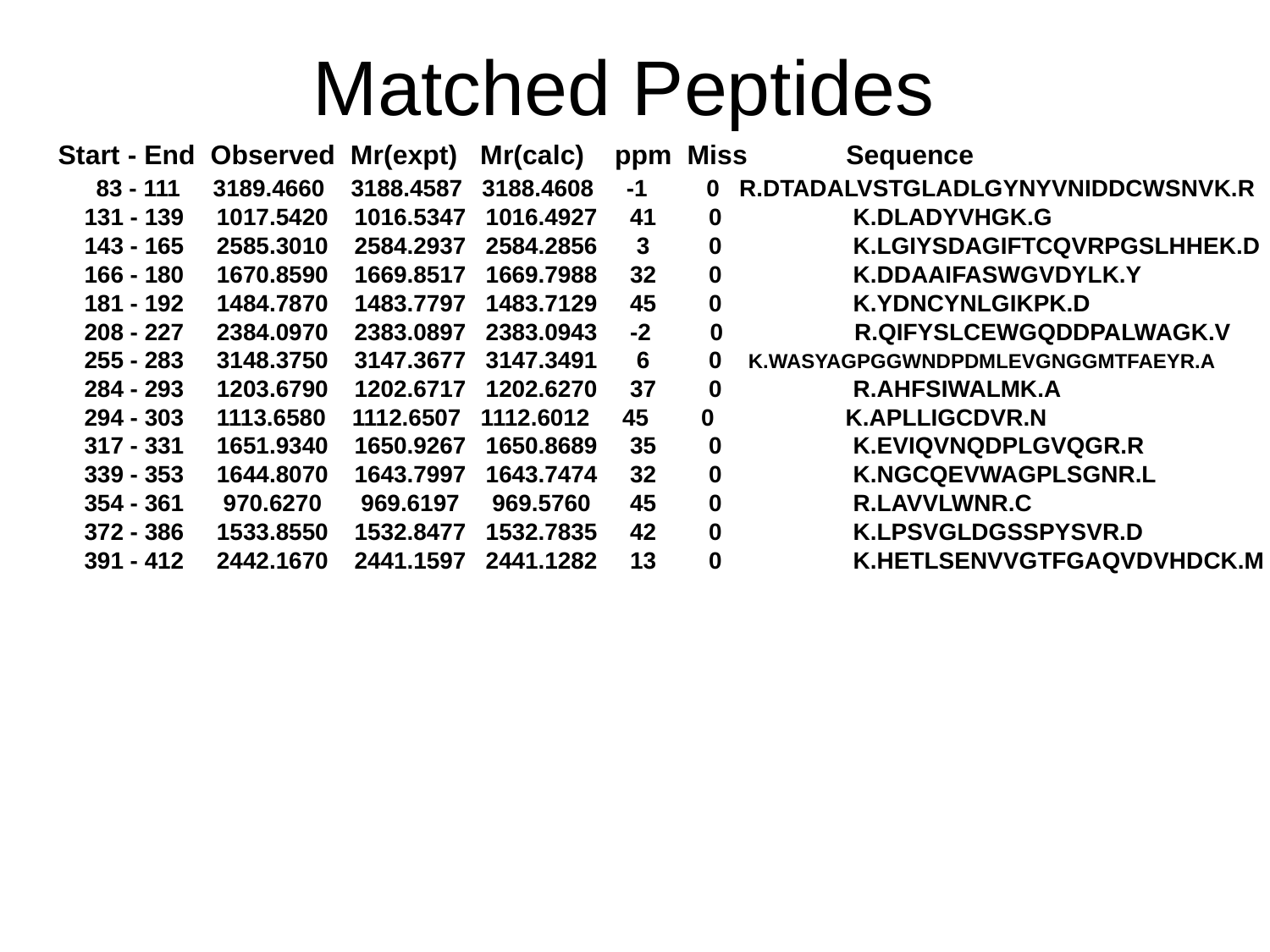

# Matched Peptides
Start - End Observed Mr(expt) Mr(calc) ppm Miss Sequence
 83 - 111 3189.4660 3188.4587 3188.4608 -1 0 R.DTADALVSTGLADLGYNYVNIDDCWSNVK.R
 131 - 139 1017.5420 1016.5347 1016.4927 41 0 K.DLADYVHGK.G
 143 - 165 2585.3010 2584.2937 2584.2856 3 0 K.LGIYSDAGIFTCQVRPGSLHHEK.D
 166 - 180 1670.8590 1669.8517 1669.7988 32 0 K.DDAAIFASWGVDYLK.Y
 181 - 192 1484.7870 1483.7797 1483.7129 45 0 K.YDNCYNLGIKPK.D
 208 - 227 2384.0970 2383.0897 2383.0943 -2 0 R.QIFYSLCEWGQDDPALWAGK.V
 255 - 283 3148.3750 3147.3677 3147.3491 6 0 K.WASYAGPGGWNDPDMLEVGNGGMTFAEYR.A
 284 - 293 1203.6790 1202.6717 1202.6270 37 0 R.AHFSIWALMK.A
 294 - 303 1113.6580 1112.6507 1112.6012 45 0 K.APLLIGCDVR.N
 317 - 331 1651.9340 1650.9267 1650.8689 35 0 K.EVIQVNQDPLGVQGR.R
 339 - 353 1644.8070 1643.7997 1643.7474 32 0 K.NGCQEVWAGPLSGNR.L
 354 - 361 970.6270 969.6197 969.5760 45 0 R.LAVVLWNR.C
 372 - 386 1533.8550 1532.8477 1532.7835 42 0 K.LPSVGLDGSSPYSVR.D
 391 - 412 2442.1670 2441.1597 2441.1282 13 0 K.HETLSENVVGTFGAQVDVHDCK.M

## Slide 35
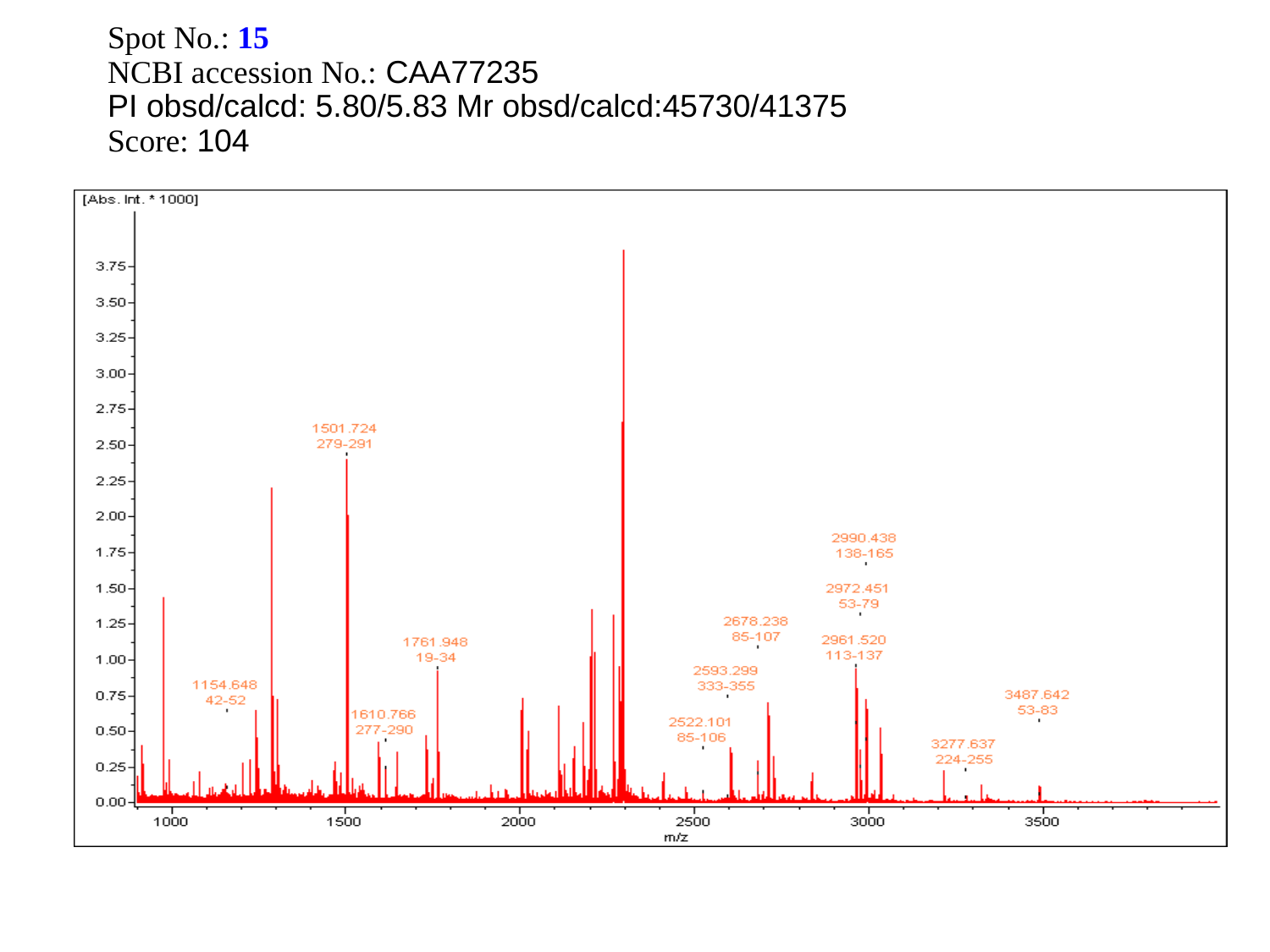

Spot No.: 15
NCBI accession No.: CAA77235
PI obsd/calcd: 5.80/5.83 Mr obsd/calcd:45730/41375
Score: 104

## Slide 36
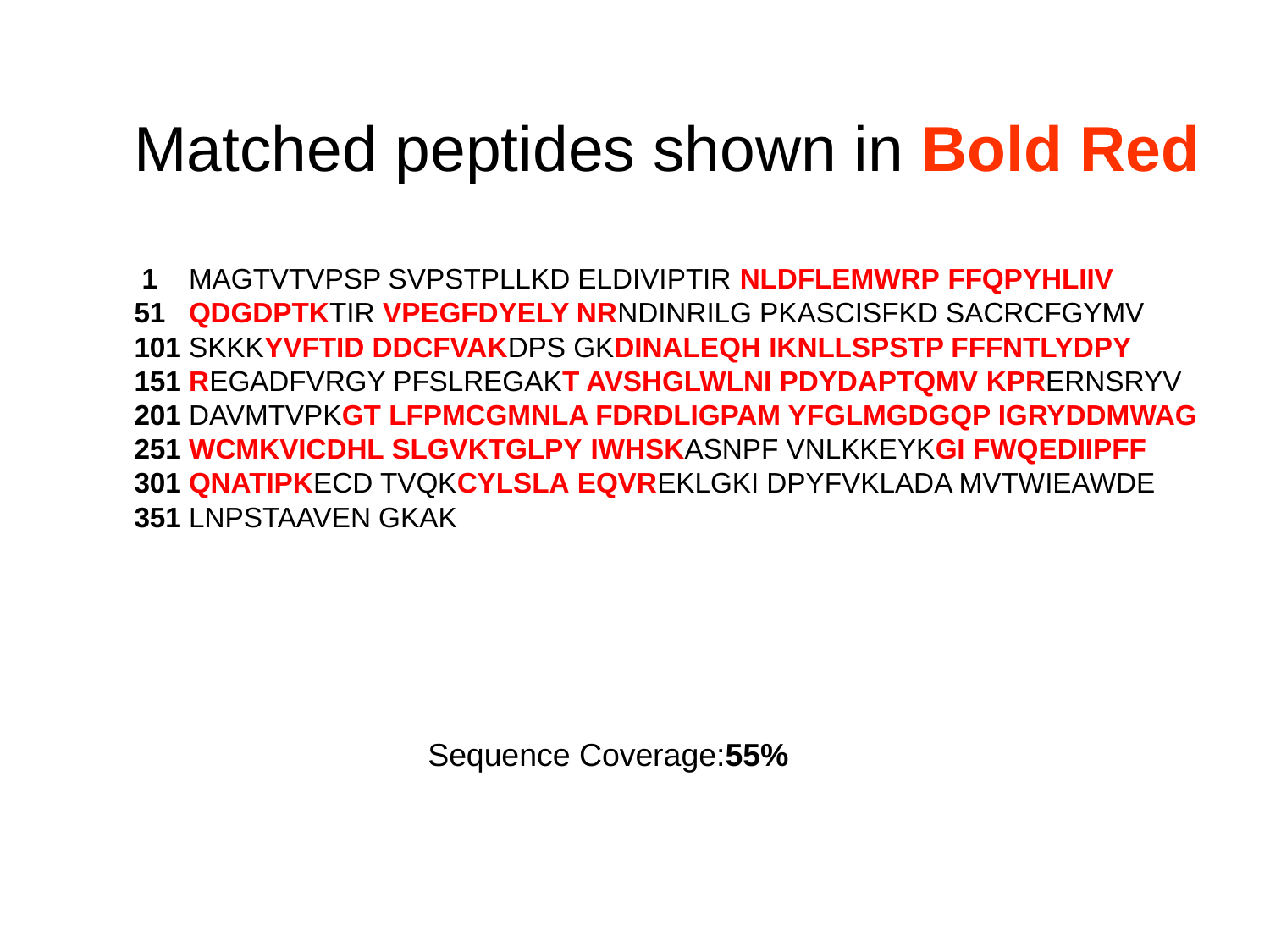

Matched peptides shown in Bold Red
 1 MAGTVTVPSP SVPSTPLLKD ELDIVIPTIR NLDFLEMWRP FFQPYHLIIV
51 QDGDPTKTIR VPEGFDYELY NRNDINRILG PKASCISFKD SACRCFGYMV
101 SKKKYVFTID DDCFVAKDPS GKDINALEQH IKNLLSPSTP FFFNTLYDPY
151 REGADFVRGY PFSLREGAKT AVSHGLWLNI PDYDAPTQMV KPRERNSRYV
201 DAVMTVPKGT LFPMCGMNLA FDRDLIGPAM YFGLMGDGQP IGRYDDMWAG
251 WCMKVICDHL SLGVKTGLPY IWHSKASNPF VNLKKEYKGI FWQEDIIPFF
301 QNATIPKECD TVQKCYLSLA EQVREKLGKI DPYFVKLADA MVTWIEAWDE
351 LNPSTAAVEN GKAK
# Sequence Coverage:55%

## Slide 37
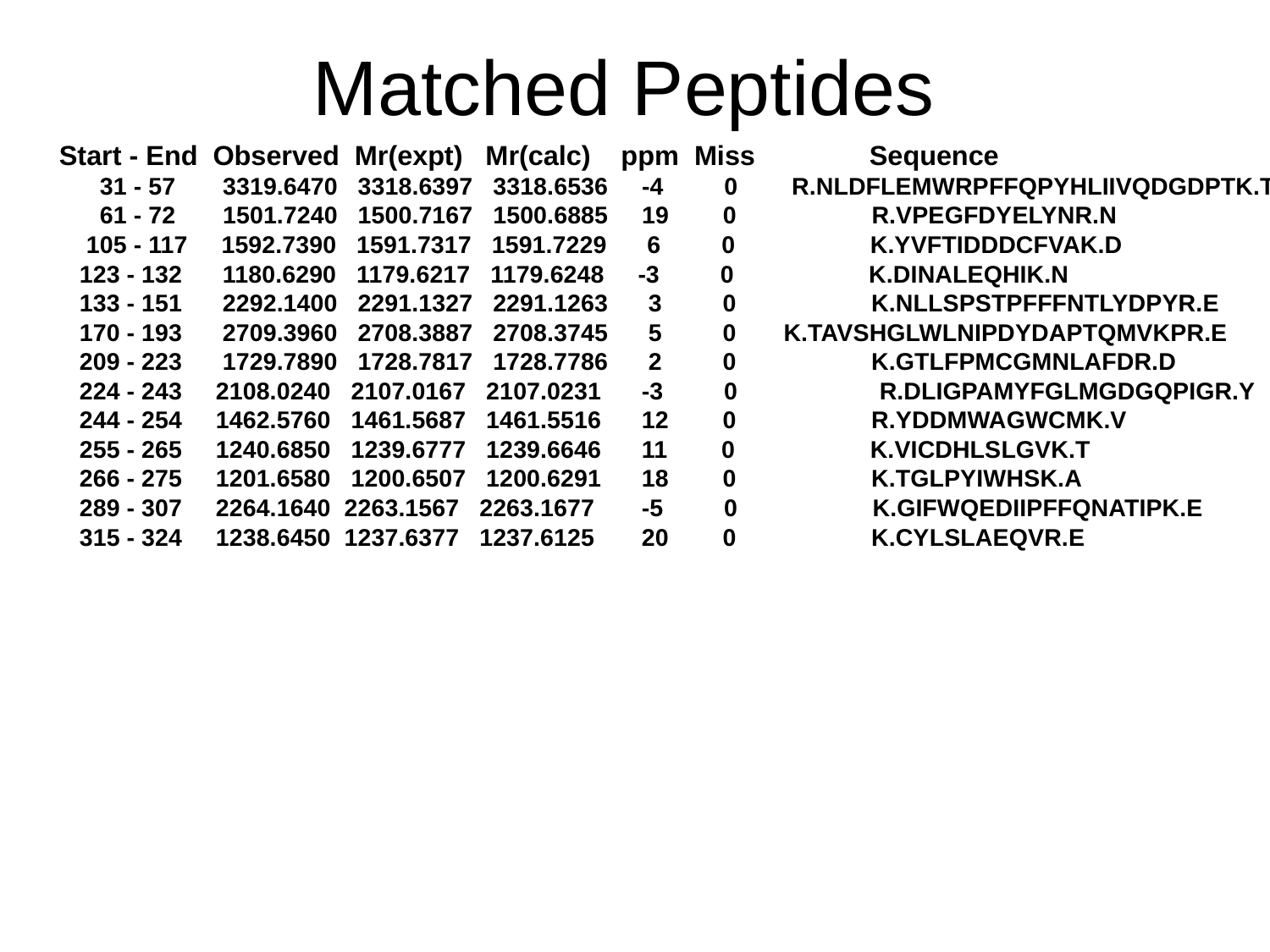

# Matched Peptides
Start - End Observed Mr(expt) Mr(calc) ppm Miss Sequence
 31 - 57 3319.6470 3318.6397 3318.6536 -4 0 R.NLDFLEMWRPFFQPYHLIIVQDGDPTK.T
 61 - 72 1501.7240 1500.7167 1500.6885 19 0 R.VPEGFDYELYNR.N
 105 - 117 1592.7390 1591.7317 1591.7229 6 0 K.YVFTIDDDCFVAK.D
 123 - 132 1180.6290 1179.6217 1179.6248 -3 0 K.DINALEQHIK.N
 133 - 151 2292.1400 2291.1327 2291.1263 3 0 K.NLLSPSTPFFFNTLYDPYR.E
 170 - 193 2709.3960 2708.3887 2708.3745 5 0 K.TAVSHGLWLNIPDYDAPTQMVKPR.E
 209 - 223 1729.7890 1728.7817 1728.7786 2 0 K.GTLFPMCGMNLAFDR.D
 224 - 243 2108.0240 2107.0167 2107.0231 -3 0 R.DLIGPAMYFGLMGDGQPIGR.Y
 244 - 254 1462.5760 1461.5687 1461.5516 12 0 R.YDDMWAGWCMK.V
 255 - 265 1240.6850 1239.6777 1239.6646 11 0 K.VICDHLSLGVK.T
 266 - 275 1201.6580 1200.6507 1200.6291 18 0 K.TGLPYIWHSK.A
 289 - 307 2264.1640 2263.1567 2263.1677 -5 0 K.GIFWQEDIIPFFQNATIPK.E
 315 - 324 1238.6450 1237.6377 1237.6125 20 0 K.CYLSLAEQVR.E

## Slide 38
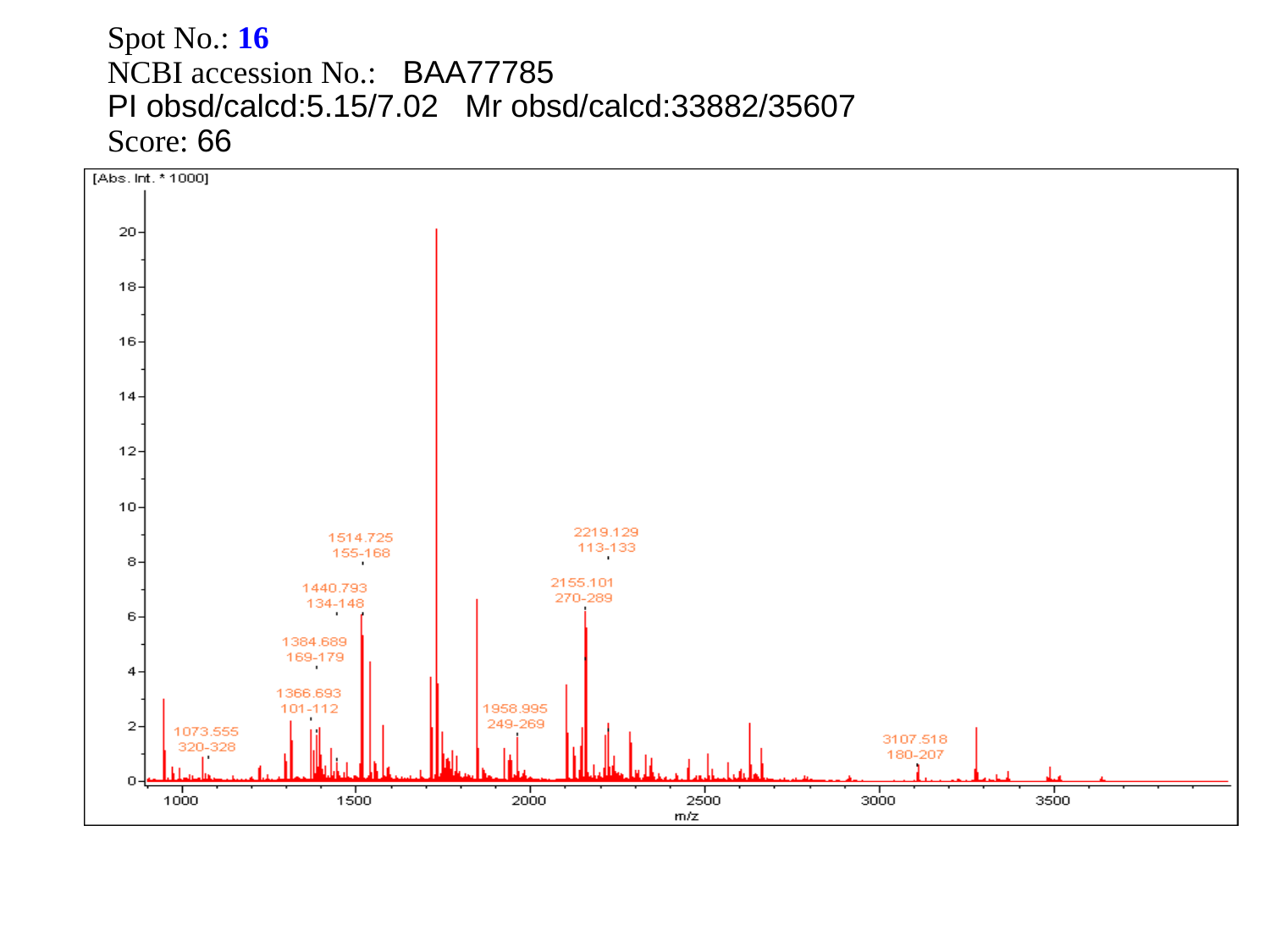

Spot No.: 16
NCBI accession No.: BAA77785
PI obsd/calcd:5.15/7.02 Mr obsd/calcd:33882/35607
Score: 66

## Slide 39
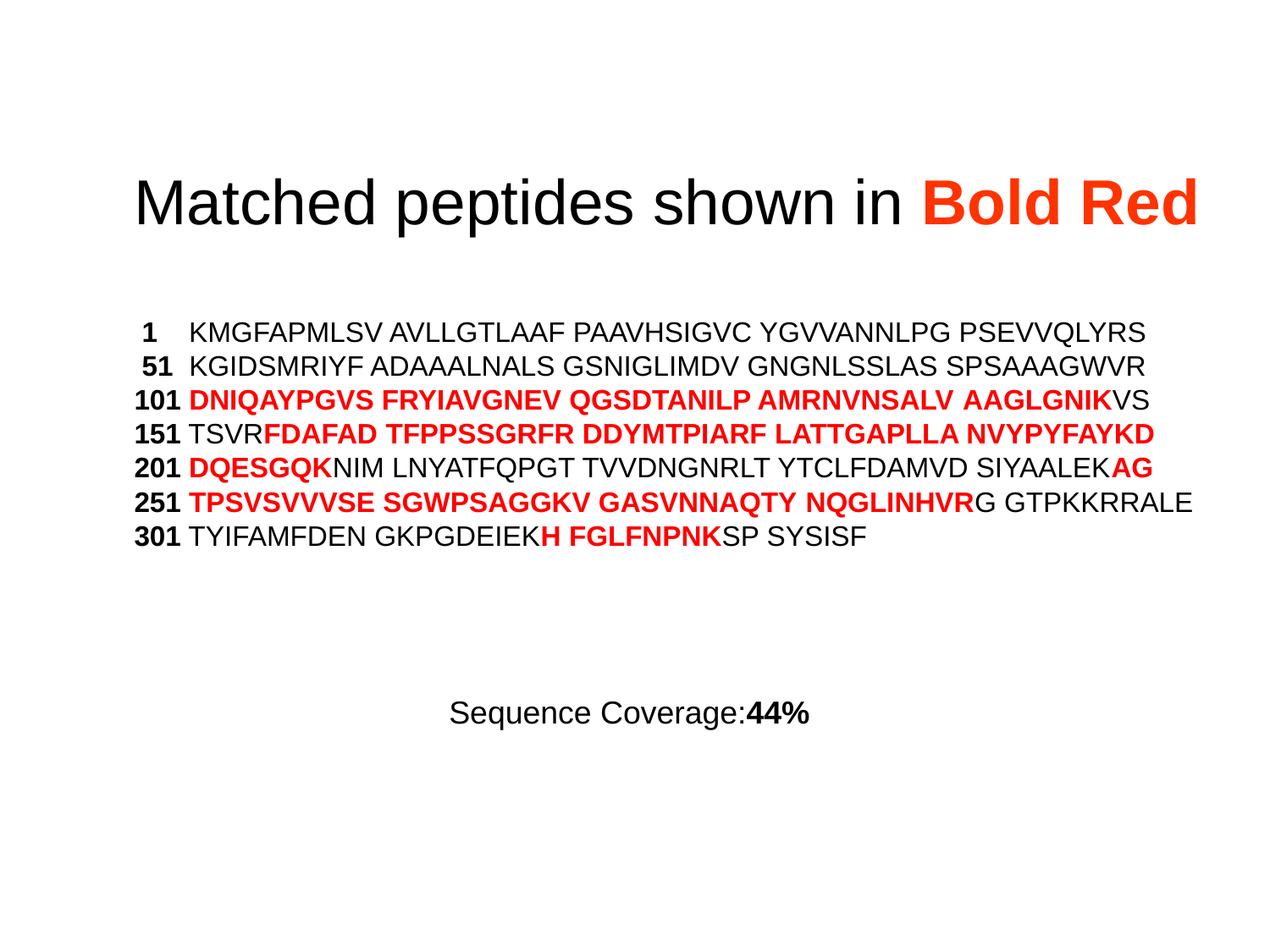

Matched peptides shown in Bold Red
 1 KMGFAPMLSV AVLLGTLAAF PAAVHSIGVC YGVVANNLPG PSEVVQLYRS
 51 KGIDSMRIYF ADAAALNALS GSNIGLIMDV GNGNLSSLAS SPSAAAGWVR
101 DNIQAYPGVS FRYIAVGNEV QGSDTANILP AMRNVNSALV AAGLGNIKVS
151 TSVRFDAFAD TFPPSSGRFR DDYMTPIARF LATTGAPLLA NVYPYFAYKD
201 DQESGQKNIM LNYATFQPGT TVVDNGNRLT YTCLFDAMVD SIYAALEKAG
251 TPSVSVVVSE SGWPSAGGKV GASVNNAQTY NQGLINHVRG GTPKKRRALE
301 TYIFAMFDEN GKPGDEIEKH FGLFNPNKSP SYSISF
# Sequence Coverage:44%

## Slide 40
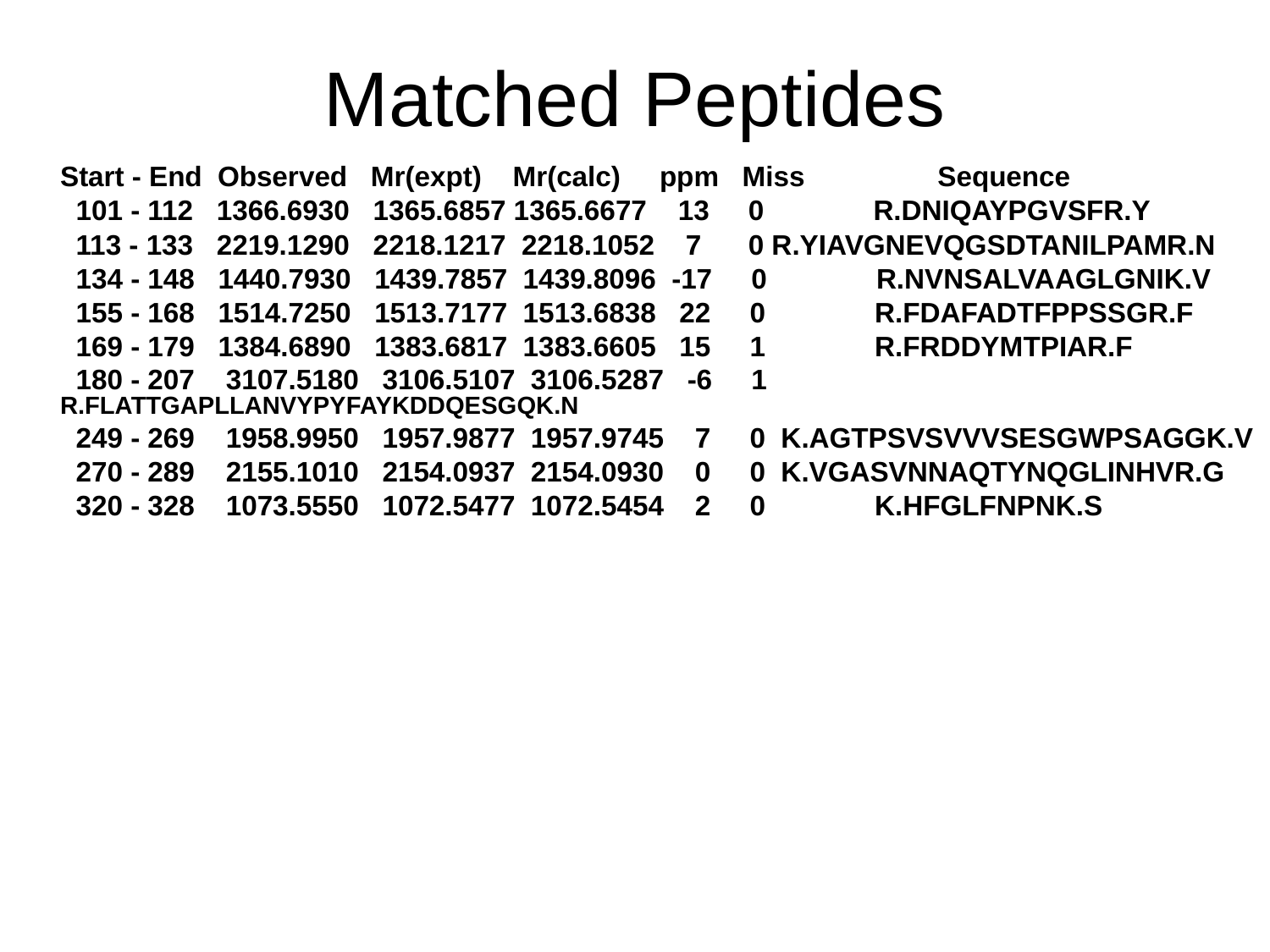

# Matched Peptides
Start - End Observed Mr(expt) Mr(calc) ppm Miss Sequence
 101 - 112 1366.6930 1365.6857 1365.6677 13 0 R.DNIQAYPGVSFR.Y
 113 - 133 2219.1290 2218.1217 2218.1052 7 0 R.YIAVGNEVQGSDTANILPAMR.N
 134 - 148 1440.7930 1439.7857 1439.8096 -17 0 R.NVNSALVAAGLGNIK.V
 155 - 168 1514.7250 1513.7177 1513.6838 22 0 R.FDAFADTFPPSSGR.F
 169 - 179 1384.6890 1383.6817 1383.6605 15 1 R.FRDDYMTPIAR.F
 180 - 207 3107.5180 3106.5107 3106.5287 -6 1 R.FLATTGAPLLANVYPYFAYKDDQESGQK.N
 249 - 269 1958.9950 1957.9877 1957.9745 7 0 K.AGTPSVSVVVSESGWPSAGGK.V
 270 - 289 2155.1010 2154.0937 2154.0930 0 0 K.VGASVNNAQTYNQGLINHVR.G
 320 - 328 1073.5550 1072.5477 1072.5454 2 0 K.HFGLFNPNK.S

## Slide 41
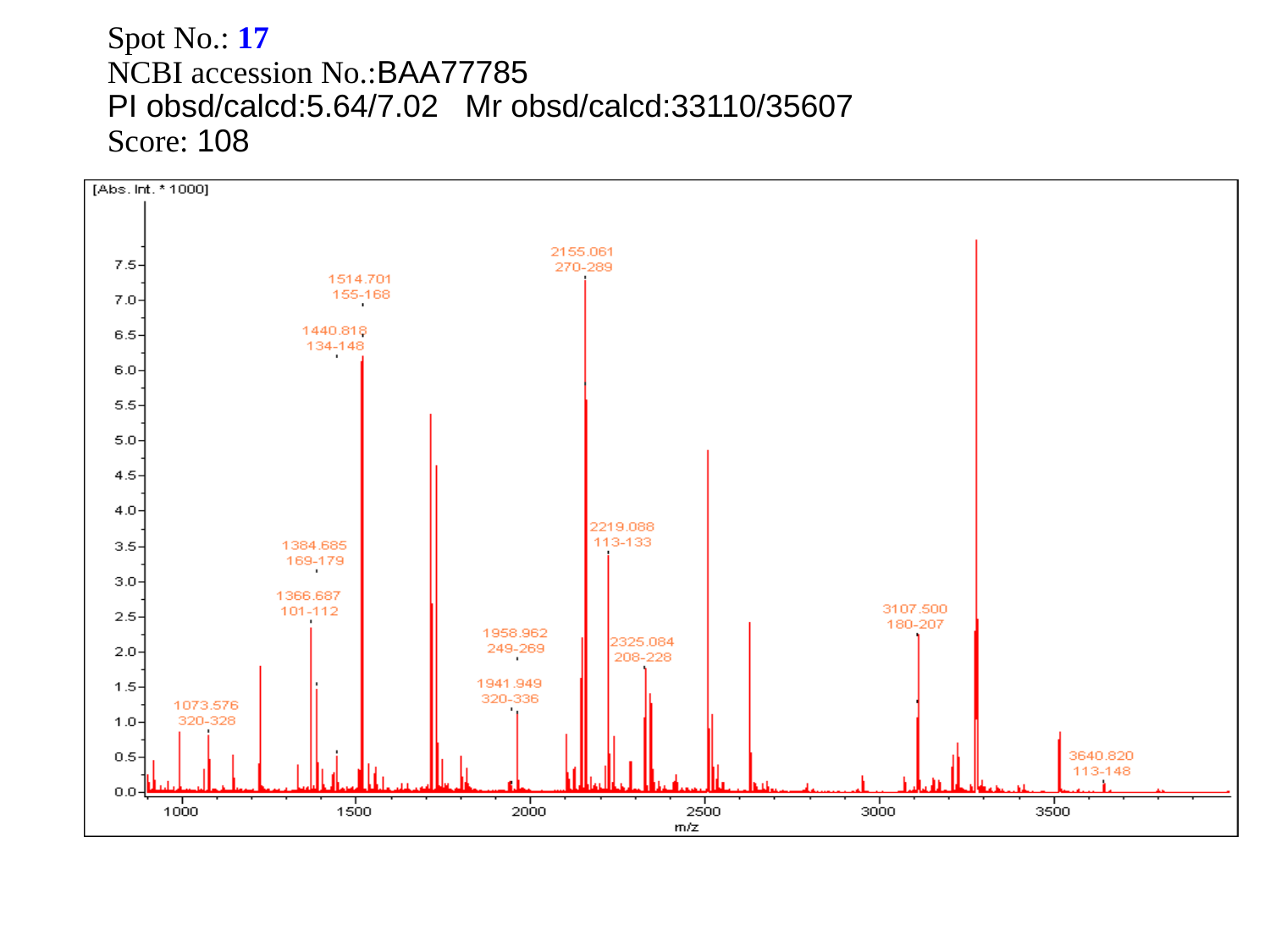

Spot No.: 17
NCBI accession No.:BAA77785
PI obsd/calcd:5.64/7.02 Mr obsd/calcd:33110/35607
Score: 108

## Slide 42
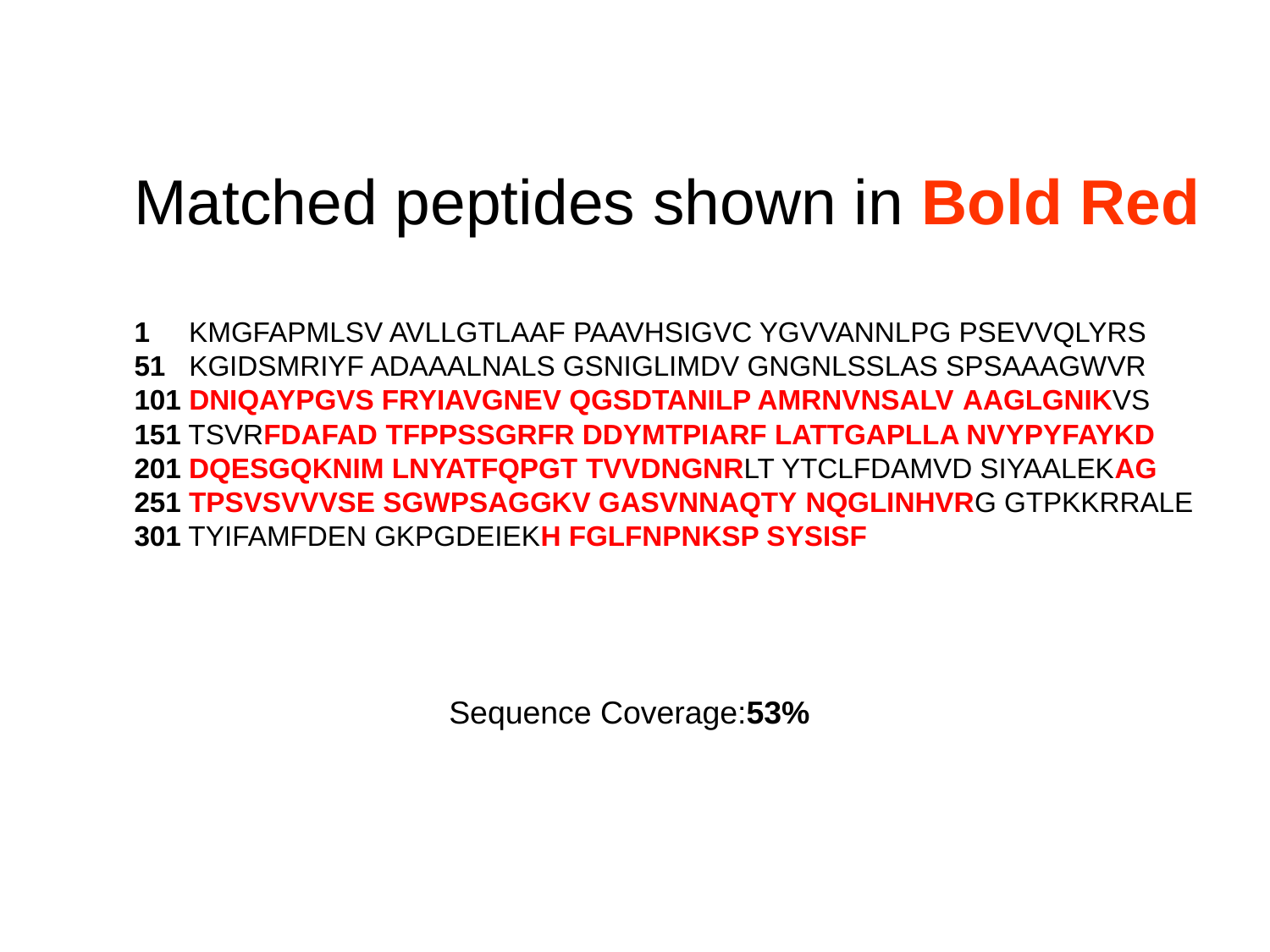

Matched peptides shown in Bold Red
1 KMGFAPMLSV AVLLGTLAAF PAAVHSIGVC YGVVANNLPG PSEVVQLYRS
51 KGIDSMRIYF ADAAALNALS GSNIGLIMDV GNGNLSSLAS SPSAAAGWVR
101 DNIQAYPGVS FRYIAVGNEV QGSDTANILP AMRNVNSALV AAGLGNIKVS
151 TSVRFDAFAD TFPPSSGRFR DDYMTPIARF LATTGAPLLA NVYPYFAYKD
201 DQESGQKNIM LNYATFQPGT TVVDNGNRLT YTCLFDAMVD SIYAALEKAG
251 TPSVSVVVSE SGWPSAGGKV GASVNNAQTY NQGLINHVRG GTPKKRRALE
301 TYIFAMFDEN GKPGDEIEKH FGLFNPNKSP SYSISF
# Sequence Coverage:53%

## Slide 43
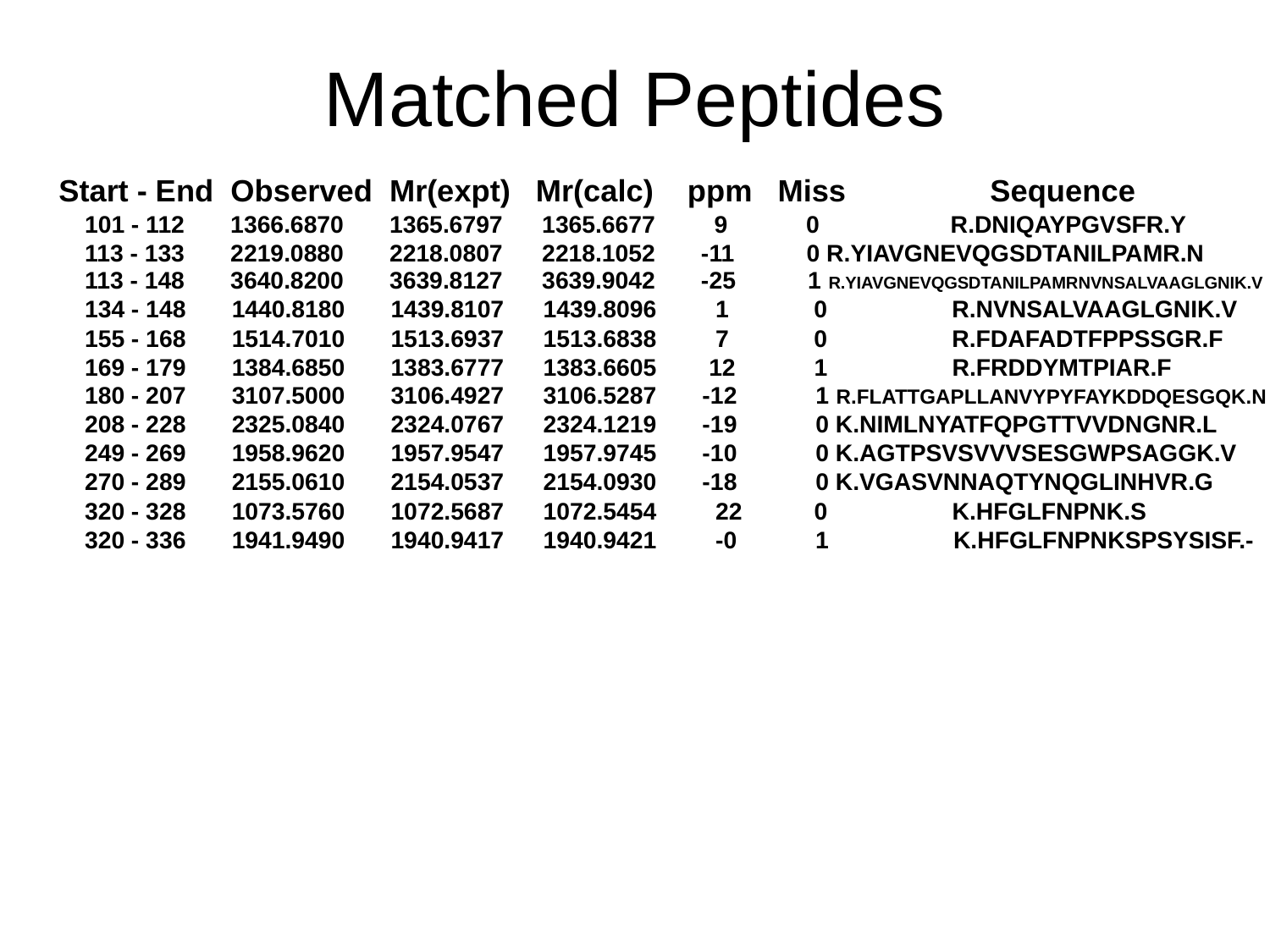

# Matched Peptides
Start - End Observed Mr(expt) Mr(calc) ppm Miss Sequence
 101 - 112 1366.6870 1365.6797 1365.6677 9 0 R.DNIQAYPGVSFR.Y
 113 - 133 2219.0880 2218.0807 2218.1052 -11 0 R.YIAVGNEVQGSDTANILPAMR.N
 113 - 148 3640.8200 3639.8127 3639.9042 -25 1 R.YIAVGNEVQGSDTANILPAMRNVNSALVAAGLGNIK.V
 134 - 148 1440.8180 1439.8107 1439.8096 1 0 R.NVNSALVAAGLGNIK.V
 155 - 168 1514.7010 1513.6937 1513.6838 7 0 R.FDAFADTFPPSSGR.F
 169 - 179 1384.6850 1383.6777 1383.6605 12 1 R.FRDDYMTPIAR.F
 180 - 207 3107.5000 3106.4927 3106.5287 -12 1 R.FLATTGAPLLANVYPYFAYKDDQESGQK.N
 208 - 228 2325.0840 2324.0767 2324.1219 -19 0 K.NIMLNYATFQPGTTVVDNGNR.L
 249 - 269 1958.9620 1957.9547 1957.9745 -10 0 K.AGTPSVSVVVSESGWPSAGGK.V
 270 - 289 2155.0610 2154.0537 2154.0930 -18 0 K.VGASVNNAQTYNQGLINHVR.G
 320 - 328 1073.5760 1072.5687 1072.5454 22 0 K.HFGLFNPNK.S
 320 - 336 1941.9490 1940.9417 1940.9421 -0 1 K.HFGLFNPNKSPSYSISF.-

## Slide 44
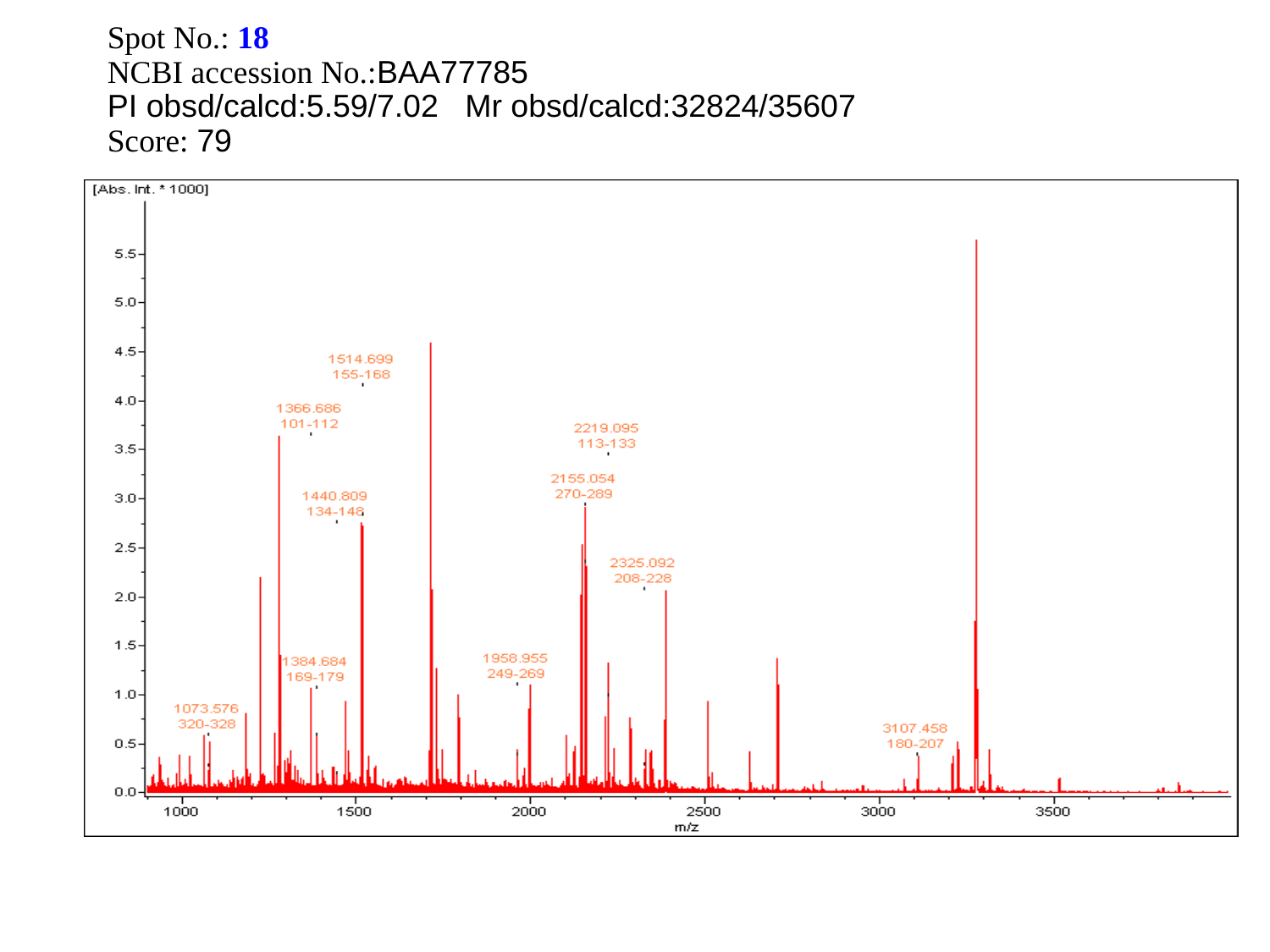

Spot No.: 18
NCBI accession No.:BAA77785
PI obsd/calcd:5.59/7.02 Mr obsd/calcd:32824/35607
Score: 79

## Slide 45
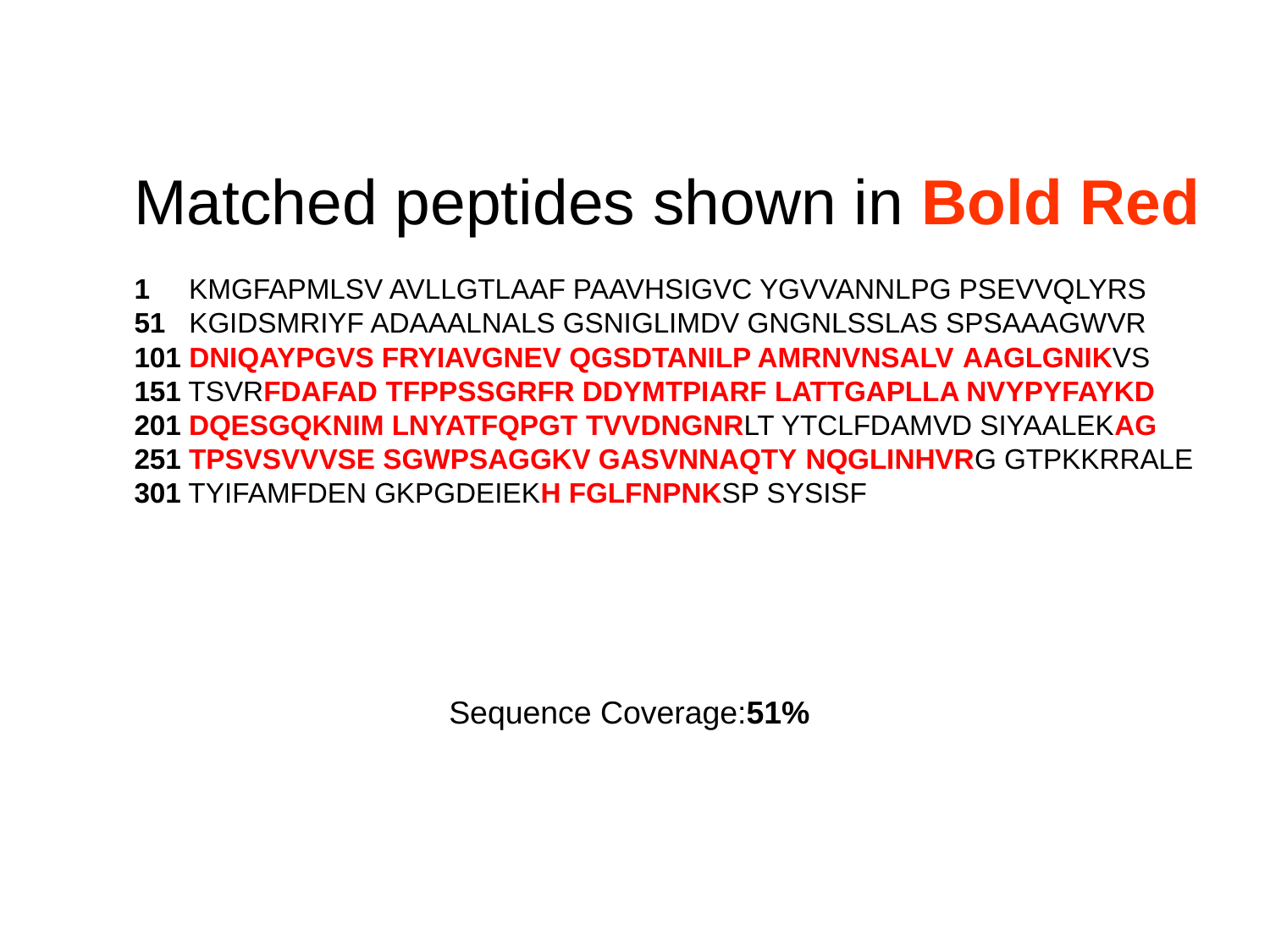

Matched peptides shown in Bold Red
1 KMGFAPMLSV AVLLGTLAAF PAAVHSIGVC YGVVANNLPG PSEVVQLYRS
51 KGIDSMRIYF ADAAALNALS GSNIGLIMDV GNGNLSSLAS SPSAAAGWVR
101 DNIQAYPGVS FRYIAVGNEV QGSDTANILP AMRNVNSALV AAGLGNIKVS
151 TSVRFDAFAD TFPPSSGRFR DDYMTPIARF LATTGAPLLA NVYPYFAYKD
201 DQESGQKNIM LNYATFQPGT TVVDNGNRLT YTCLFDAMVD SIYAALEKAG
251 TPSVSVVVSE SGWPSAGGKV GASVNNAQTY NQGLINHVRG GTPKKRRALE
301 TYIFAMFDEN GKPGDEIEKH FGLFNPNKSP SYSISF
# Sequence Coverage:51%

## Slide 46
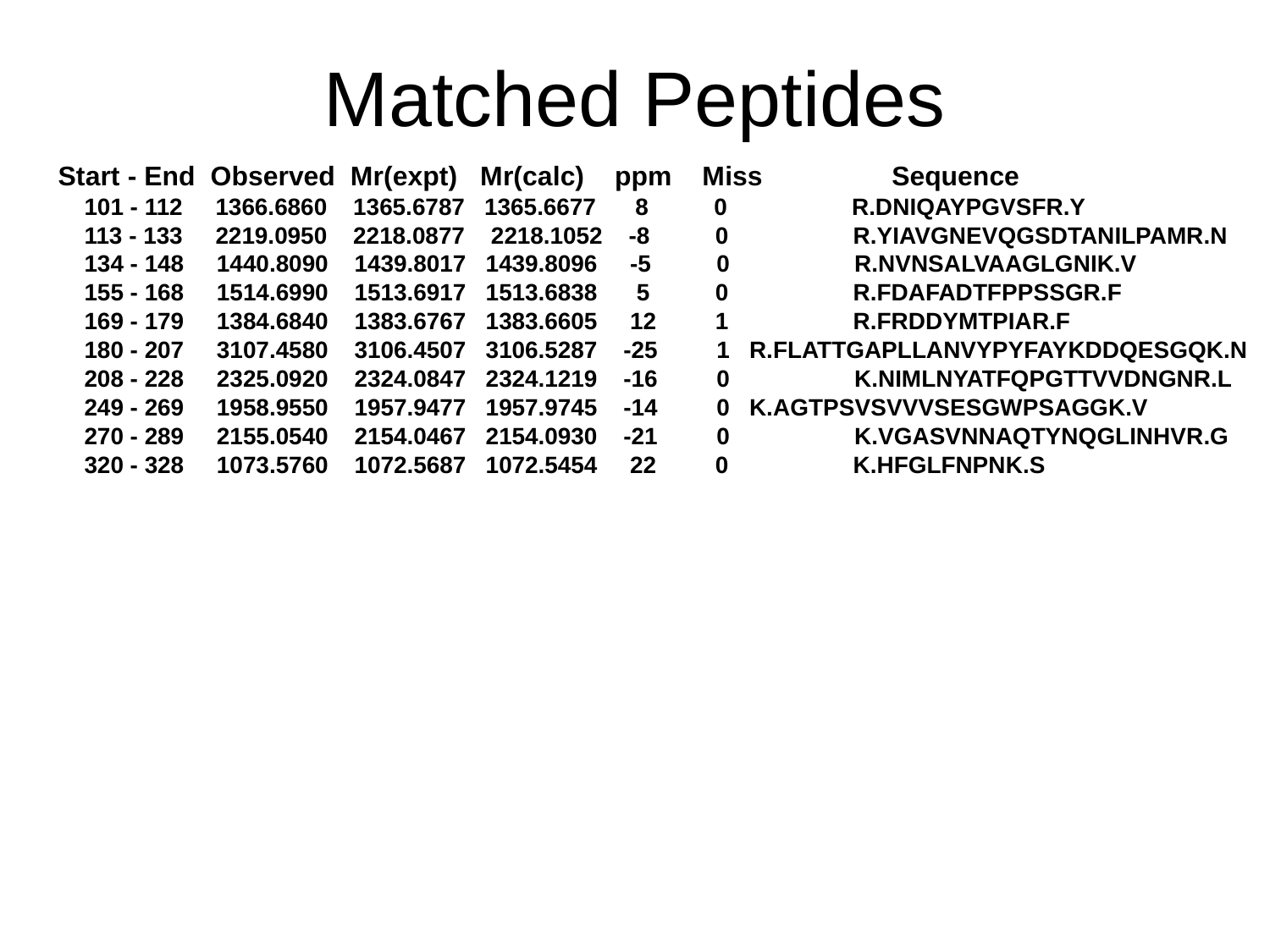

# Matched Peptides
Start - End Observed Mr(expt) Mr(calc) ppm Miss Sequence
 101 - 112 1366.6860 1365.6787 1365.6677 8 0 R.DNIQAYPGVSFR.Y
 113 - 133 2219.0950 2218.0877 2218.1052 -8 0 R.YIAVGNEVQGSDTANILPAMR.N
 134 - 148 1440.8090 1439.8017 1439.8096 -5 0 R.NVNSALVAAGLGNIK.V
 155 - 168 1514.6990 1513.6917 1513.6838 5 0 R.FDAFADTFPPSSGR.F
 169 - 179 1384.6840 1383.6767 1383.6605 12 1 R.FRDDYMTPIAR.F
 180 - 207 3107.4580 3106.4507 3106.5287 -25 1 R.FLATTGAPLLANVYPYFAYKDDQESGQK.N
 208 - 228 2325.0920 2324.0847 2324.1219 -16 0 K.NIMLNYATFQPGTTVVDNGNR.L
 249 - 269 1958.9550 1957.9477 1957.9745 -14 0 K.AGTPSVSVVVSESGWPSAGGK.V
 270 - 289 2155.0540 2154.0467 2154.0930 -21 0 K.VGASVNNAQTYNQGLINHVR.G
 320 - 328 1073.5760 1072.5687 1072.5454 22 0 K.HFGLFNPNK.S

## Slide 47
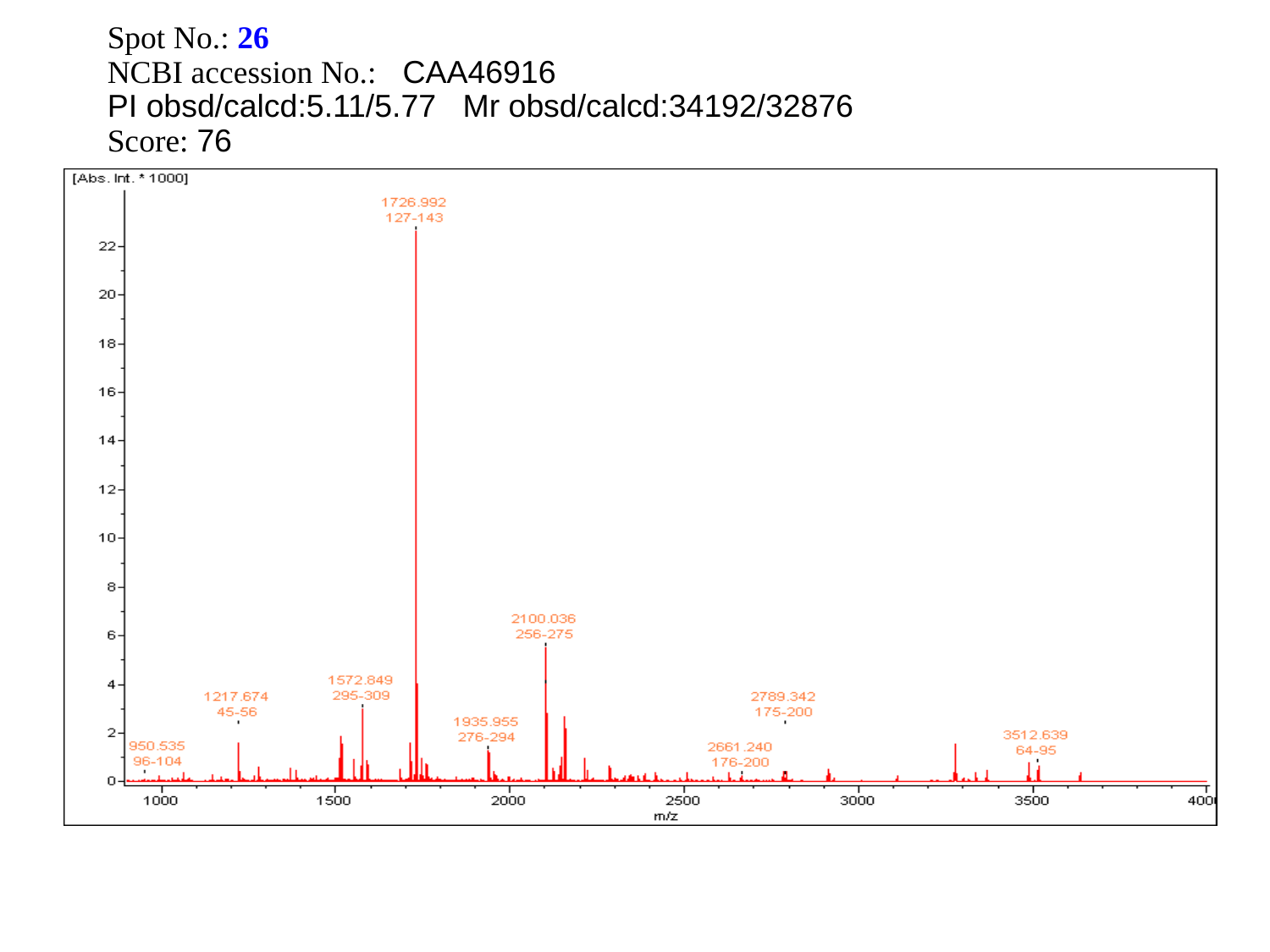

Spot No.: 26
NCBI accession No.: CAA46916
PI obsd/calcd:5.11/5.77 Mr obsd/calcd:34192/32876
Score: 76

## Slide 48
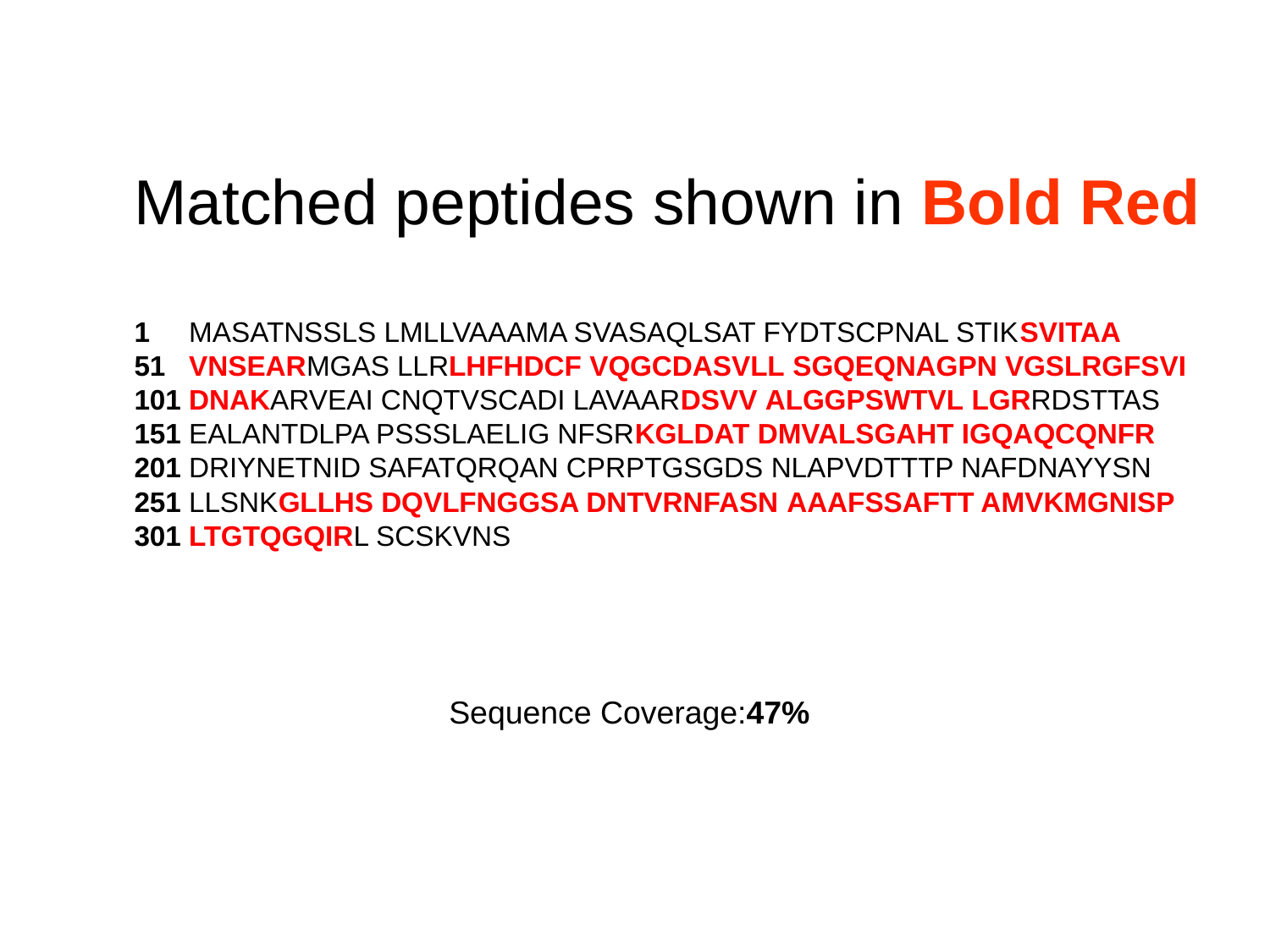

Matched peptides shown in Bold Red
1 MASATNSSLS LMLLVAAAMA SVASAQLSAT FYDTSCPNAL STIKSVITAA
51 VNSEARMGAS LLRLHFHDCF VQGCDASVLL SGQEQNAGPN VGSLRGFSVI
101 DNAKARVEAI CNQTVSCADI LAVAARDSVV ALGGPSWTVL LGRRDSTTAS
151 EALANTDLPA PSSSLAELIG NFSRKGLDAT DMVALSGAHT IGQAQCQNFR
201 DRIYNETNID SAFATQRQAN CPRPTGSGDS NLAPVDTTTP NAFDNAYYSN
251 LLSNKGLLHS DQVLFNGGSA DNTVRNFASN AAAFSSAFTT AMVKMGNISP
301 LTGTQGQIRL SCSKVNS
# Sequence Coverage:47%

## Slide 49
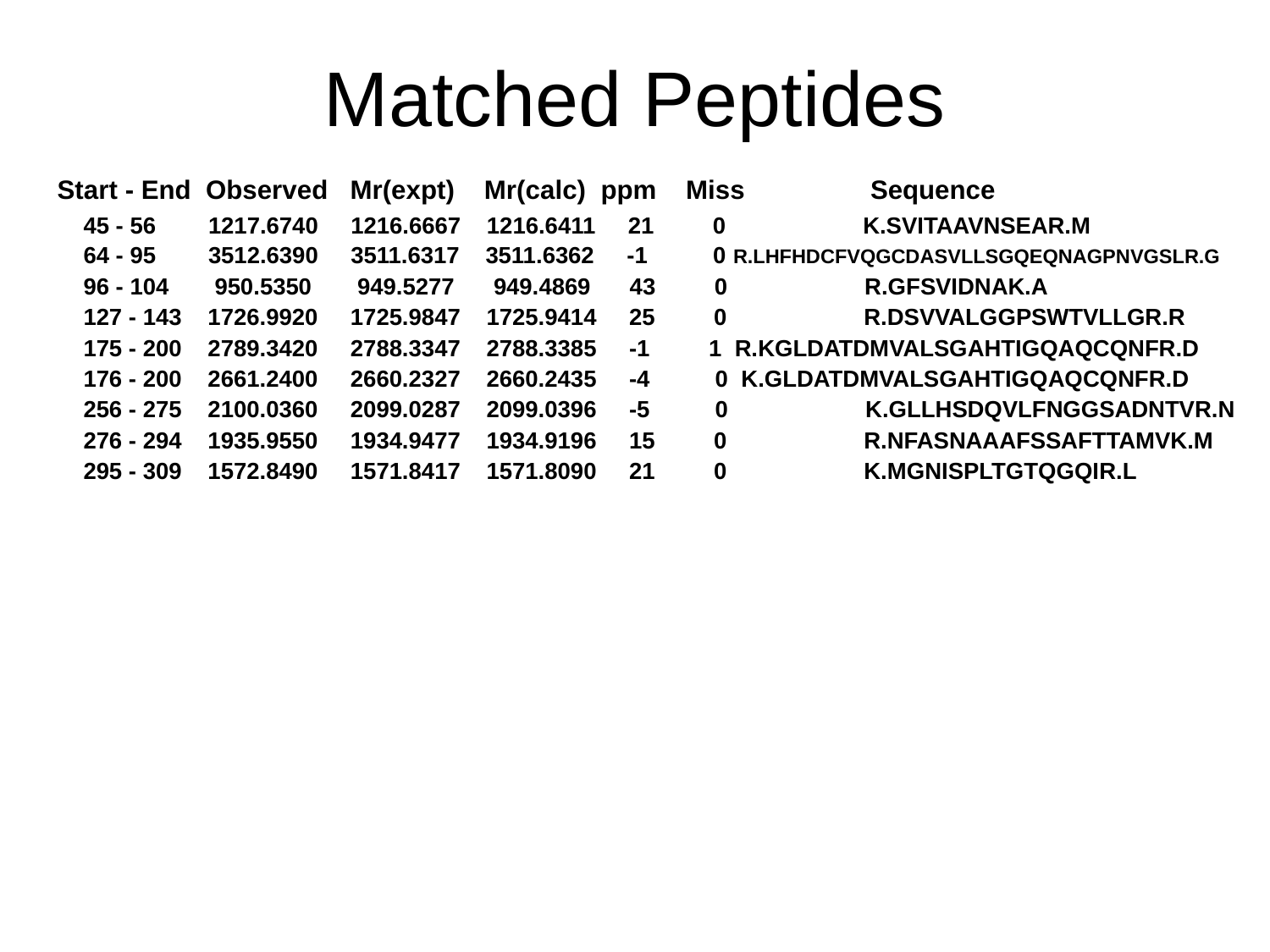

# Matched Peptides
Start - End Observed Mr(expt) Mr(calc) ppm Miss Sequence
 45 - 56 1217.6740 1216.6667 1216.6411 21 0 K.SVITAAVNSEAR.M
 64 - 95 3512.6390 3511.6317 3511.6362 -1 0 R.LHFHDCFVQGCDASVLLSGQEQNAGPNVGSLR.G
 96 - 104 950.5350 949.5277 949.4869 43 0 R.GFSVIDNAK.A
 127 - 143 1726.9920 1725.9847 1725.9414 25 0 R.DSVVALGGPSWTVLLGR.R
 175 - 200 2789.3420 2788.3347 2788.3385 -1 1 R.KGLDATDMVALSGAHTIGQAQCQNFR.D
 176 - 200 2661.2400 2660.2327 2660.2435 -4 0 K.GLDATDMVALSGAHTIGQAQCQNFR.D
 256 - 275 2100.0360 2099.0287 2099.0396 -5 0 K.GLLHSDQVLFNGGSADNTVR.N
 276 - 294 1935.9550 1934.9477 1934.9196 15 0 R.NFASNAAAFSSAFTTAMVK.M
 295 - 309 1572.8490 1571.8417 1571.8090 21 0 K.MGNISPLTGTQGQIR.L

## Slide 50
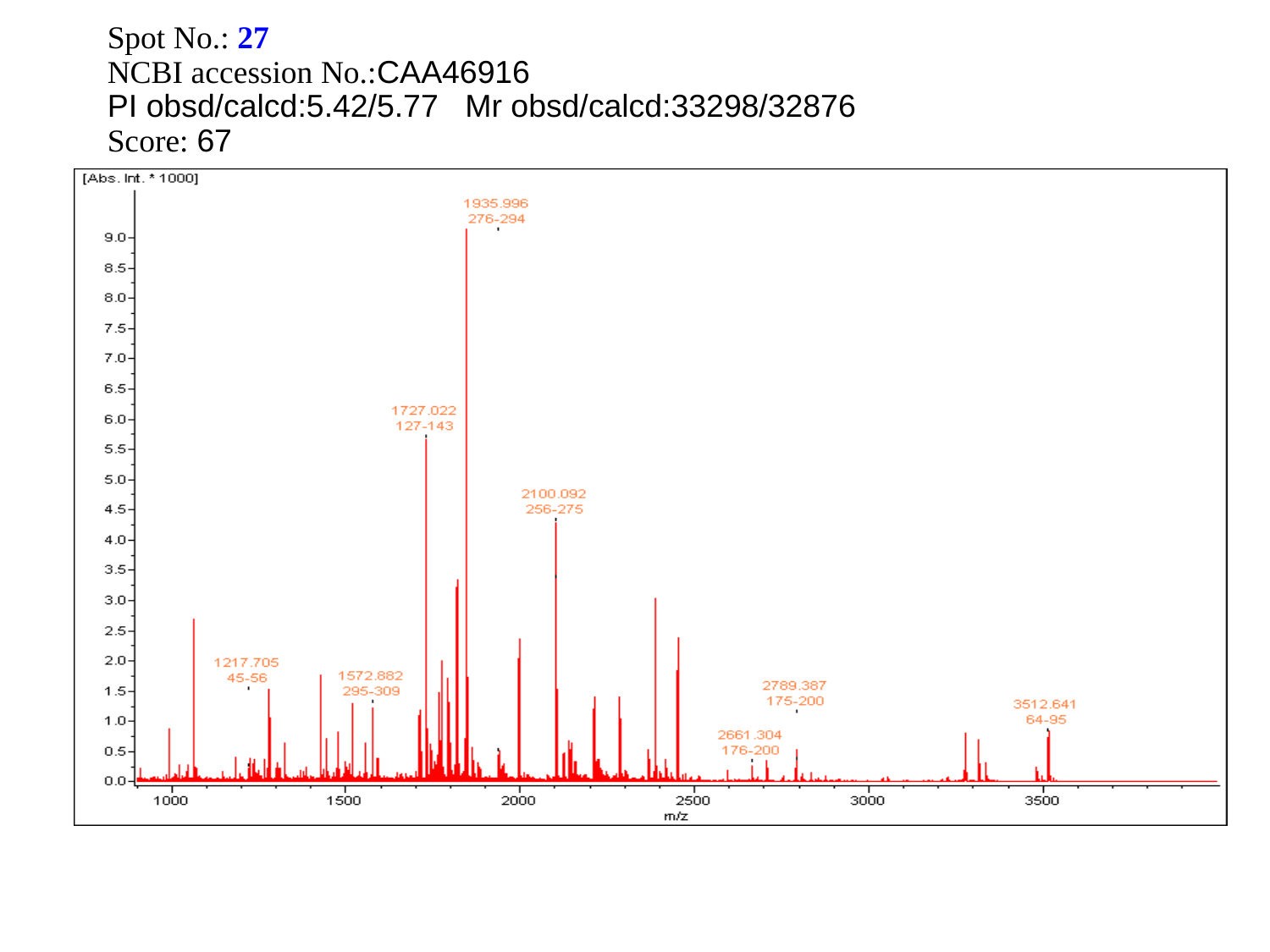

Spot No.: 27
NCBI accession No.:CAA46916
PI obsd/calcd:5.42/5.77 Mr obsd/calcd:33298/32876
Score: 67

## Slide 51
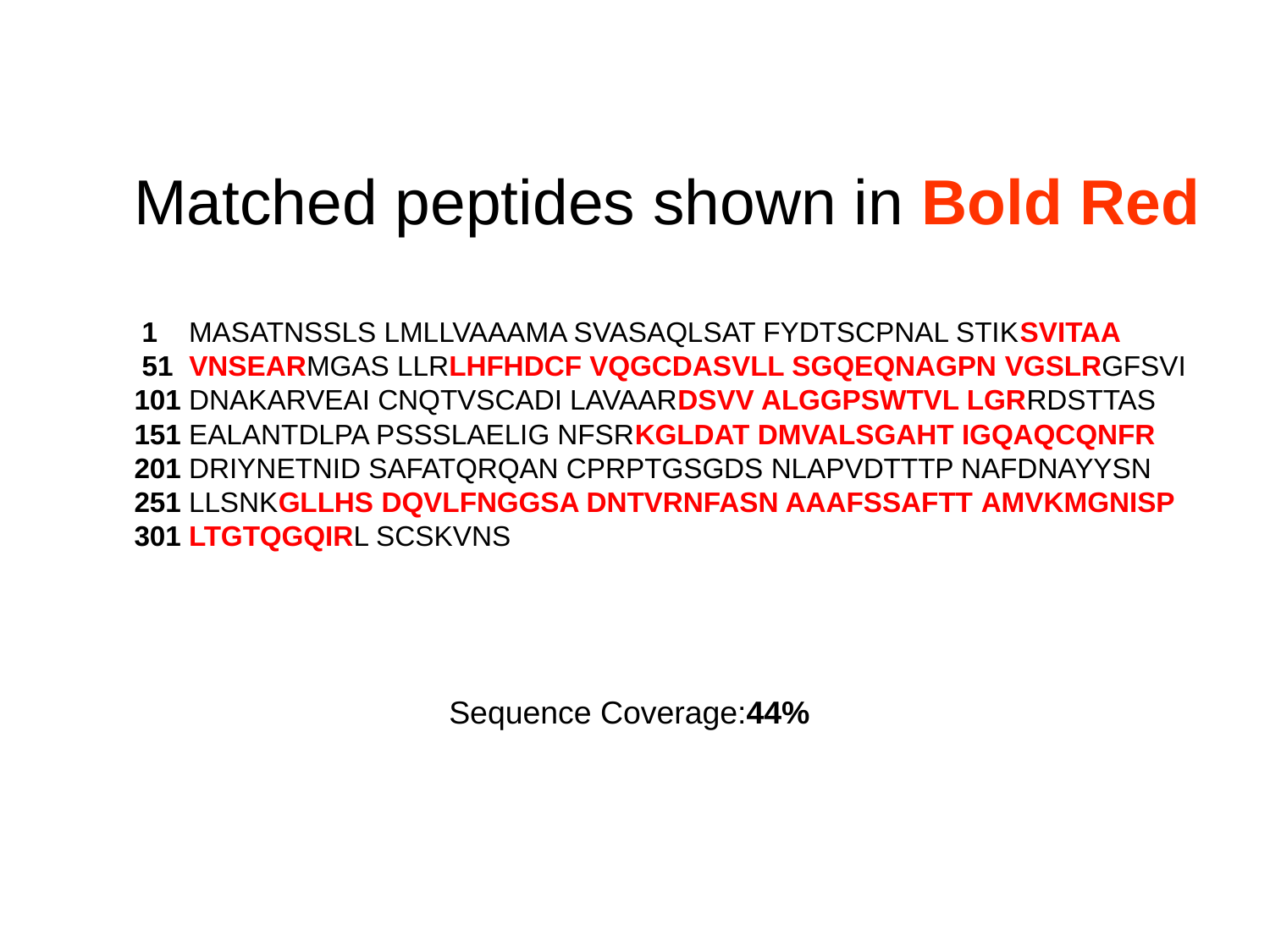

Matched peptides shown in Bold Red
 1 MASATNSSLS LMLLVAAAMA SVASAQLSAT FYDTSCPNAL STIKSVITAA
 51 VNSEARMGAS LLRLHFHDCF VQGCDASVLL SGQEQNAGPN VGSLRGFSVI
101 DNAKARVEAI CNQTVSCADI LAVAARDSVV ALGGPSWTVL LGRRDSTTAS
151 EALANTDLPA PSSSLAELIG NFSRKGLDAT DMVALSGAHT IGQAQCQNFR
201 DRIYNETNID SAFATQRQAN CPRPTGSGDS NLAPVDTTTP NAFDNAYYSN
251 LLSNKGLLHS DQVLFNGGSA DNTVRNFASN AAAFSSAFTT AMVKMGNISP
301 LTGTQGQIRL SCSKVNS
# Sequence Coverage:44%

## Slide 52
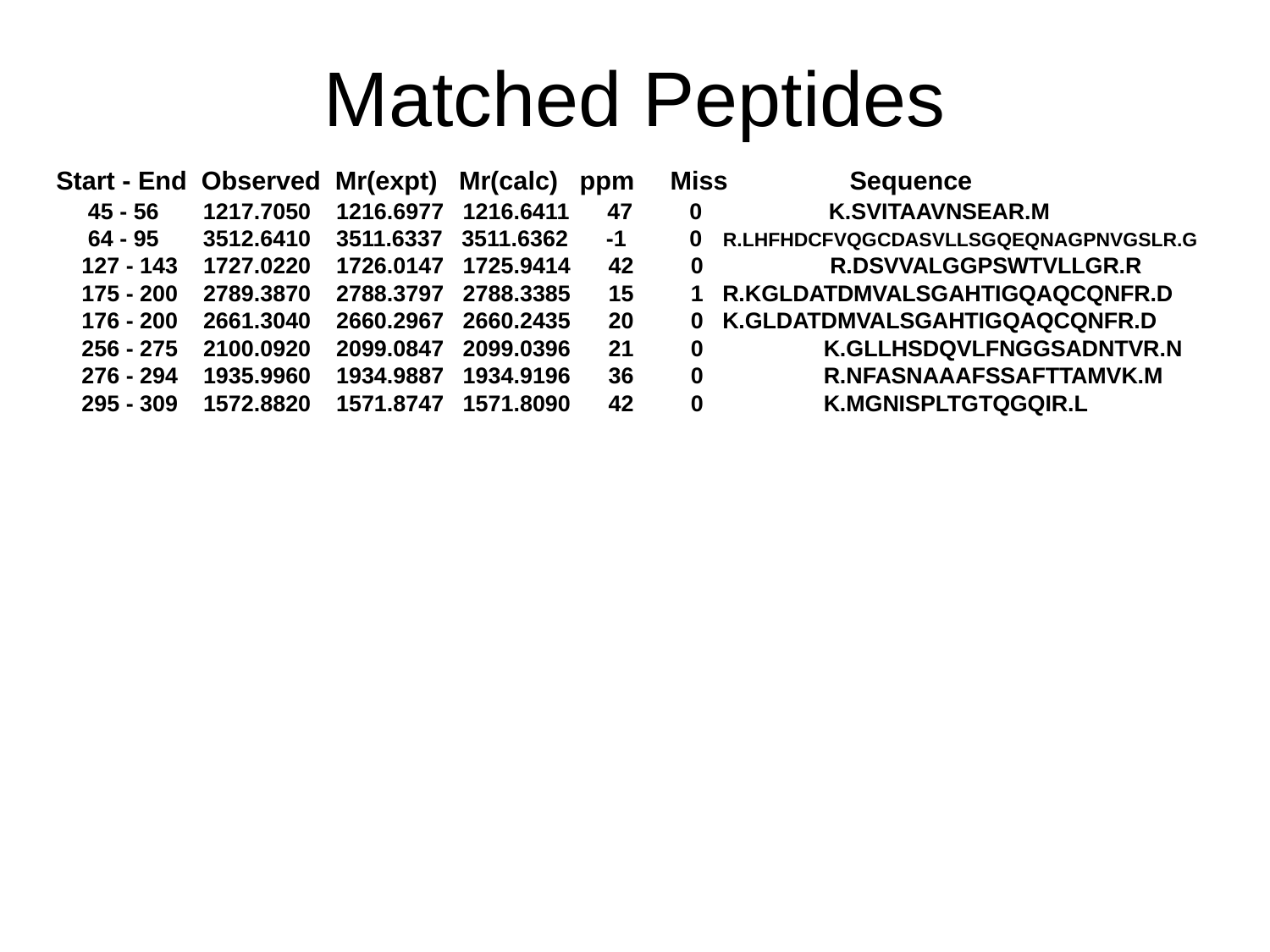

# Matched Peptides
Start - End Observed Mr(expt) Mr(calc) ppm Miss Sequence
 45 - 56 1217.7050 1216.6977 1216.6411 47 0 K.SVITAAVNSEAR.M
 64 - 95 3512.6410 3511.6337 3511.6362 -1 0 R.LHFHDCFVQGCDASVLLSGQEQNAGPNVGSLR.G
 127 - 143 1727.0220 1726.0147 1725.9414 42 0 R.DSVVALGGPSWTVLLGR.R
 175 - 200 2789.3870 2788.3797 2788.3385 15 1 R.KGLDATDMVALSGAHTIGQAQCQNFR.D
 176 - 200 2661.3040 2660.2967 2660.2435 20 0 K.GLDATDMVALSGAHTIGQAQCQNFR.D
 256 - 275 2100.0920 2099.0847 2099.0396 21 0 K.GLLHSDQVLFNGGSADNTVR.N
 276 - 294 1935.9960 1934.9887 1934.9196 36 0 R.NFASNAAAFSSAFTTAMVK.M
 295 - 309 1572.8820 1571.8747 1571.8090 42 0 K.MGNISPLTGTQGQIR.L

## Slide 53
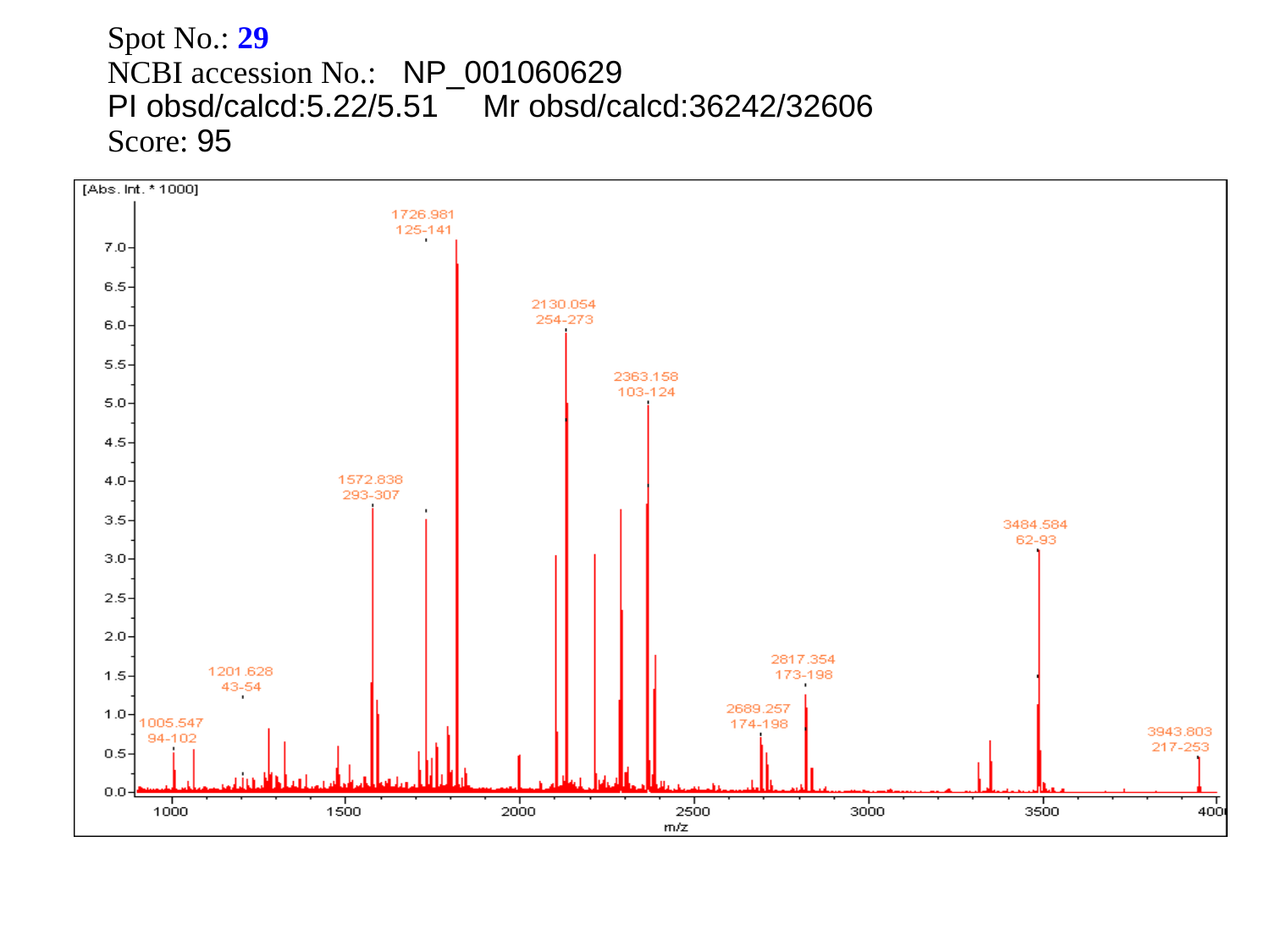

Spot No.: 29
NCBI accession No.: NP_001060629
PI obsd/calcd:5.22/5.51 Mr obsd/calcd:36242/32606
Score: 95

## Slide 54
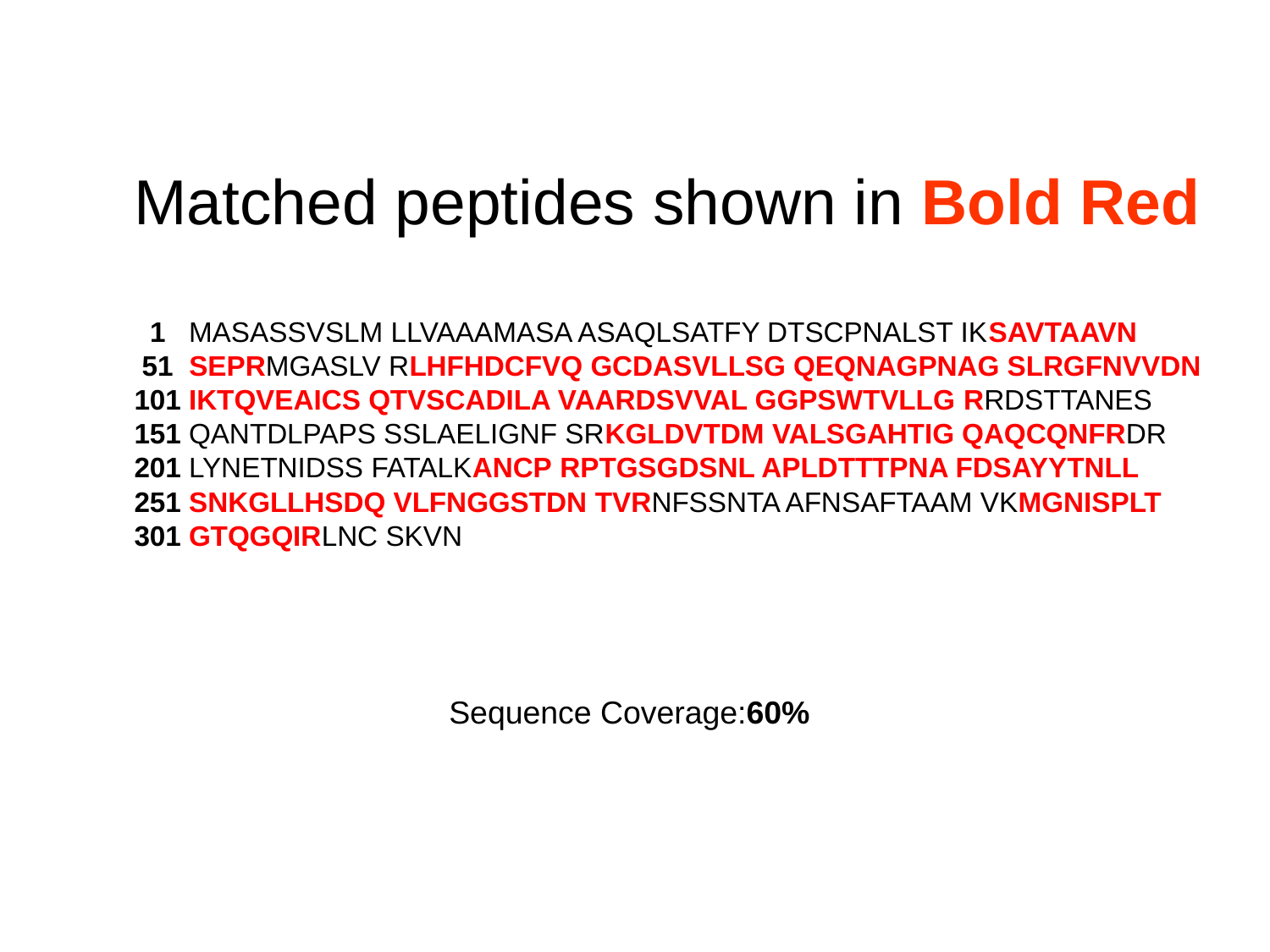

Matched peptides shown in Bold Red
 1 MASASSVSLM LLVAAAMASA ASAQLSATFY DTSCPNALST IKSAVTAAVN
 51 SEPRMGASLV RLHFHDCFVQ GCDASVLLSG QEQNAGPNAG SLRGFNVVDN
101 IKTQVEAICS QTVSCADILA VAARDSVVAL GGPSWTVLLG RRDSTTANES
151 QANTDLPAPS SSLAELIGNF SRKGLDVTDM VALSGAHTIG QAQCQNFRDR
201 LYNETNIDSS FATALKANCP RPTGSGDSNL APLDTTTPNA FDSAYYTNLL
251 SNKGLLHSDQ VLFNGGSTDN TVRNFSSNTA AFNSAFTAAM VKMGNISPLT
301 GTQGQIRLNC SKVN
# Sequence Coverage:60%

## Slide 55
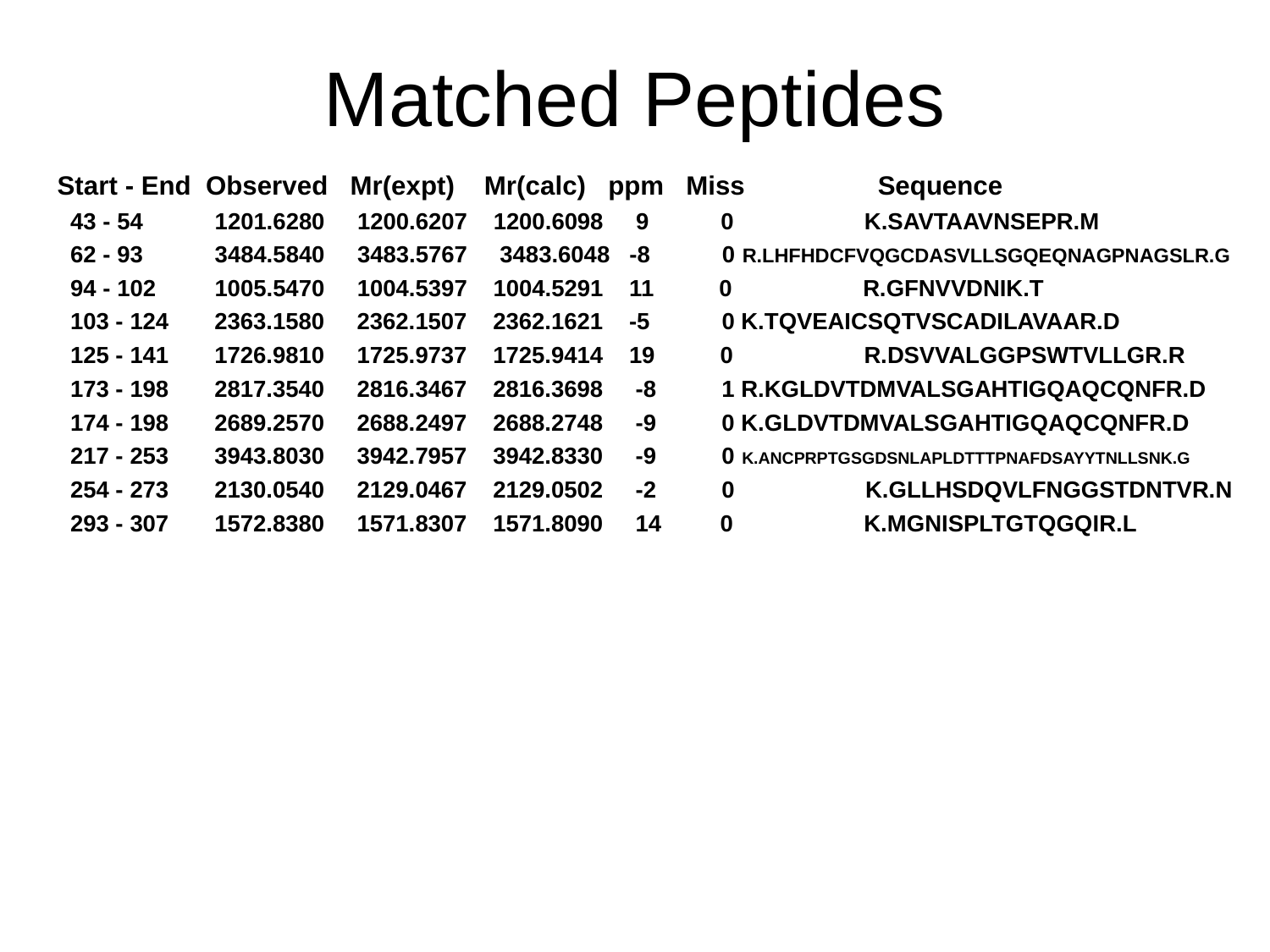

# Matched Peptides
Start - End Observed Mr(expt) Mr(calc) ppm Miss Sequence
 43 - 54 1201.6280 1200.6207 1200.6098 9 0 K.SAVTAAVNSEPR.M
 62 - 93 3484.5840 3483.5767 3483.6048 -8 0 R.LHFHDCFVQGCDASVLLSGQEQNAGPNAGSLR.G
 94 - 102 1005.5470 1004.5397 1004.5291 11 0 R.GFNVVDNIK.T
 103 - 124 2363.1580 2362.1507 2362.1621 -5 0 K.TQVEAICSQTVSCADILAVAAR.D
 125 - 141 1726.9810 1725.9737 1725.9414 19 0 R.DSVVALGGPSWTVLLGR.R
 173 - 198 2817.3540 2816.3467 2816.3698 -8 1 R.KGLDVTDMVALSGAHTIGQAQCQNFR.D
 174 - 198 2689.2570 2688.2497 2688.2748 -9 0 K.GLDVTDMVALSGAHTIGQAQCQNFR.D
 217 - 253 3943.8030 3942.7957 3942.8330 -9 0 K.ANCPRPTGSGDSNLAPLDTTTPNAFDSAYYTNLLSNK.G
 254 - 273 2130.0540 2129.0467 2129.0502 -2 0 K.GLLHSDQVLFNGGSTDNTVR.N
 293 - 307 1572.8380 1571.8307 1571.8090 14 0 K.MGNISPLTGTQGQIR.L

## Slide 56
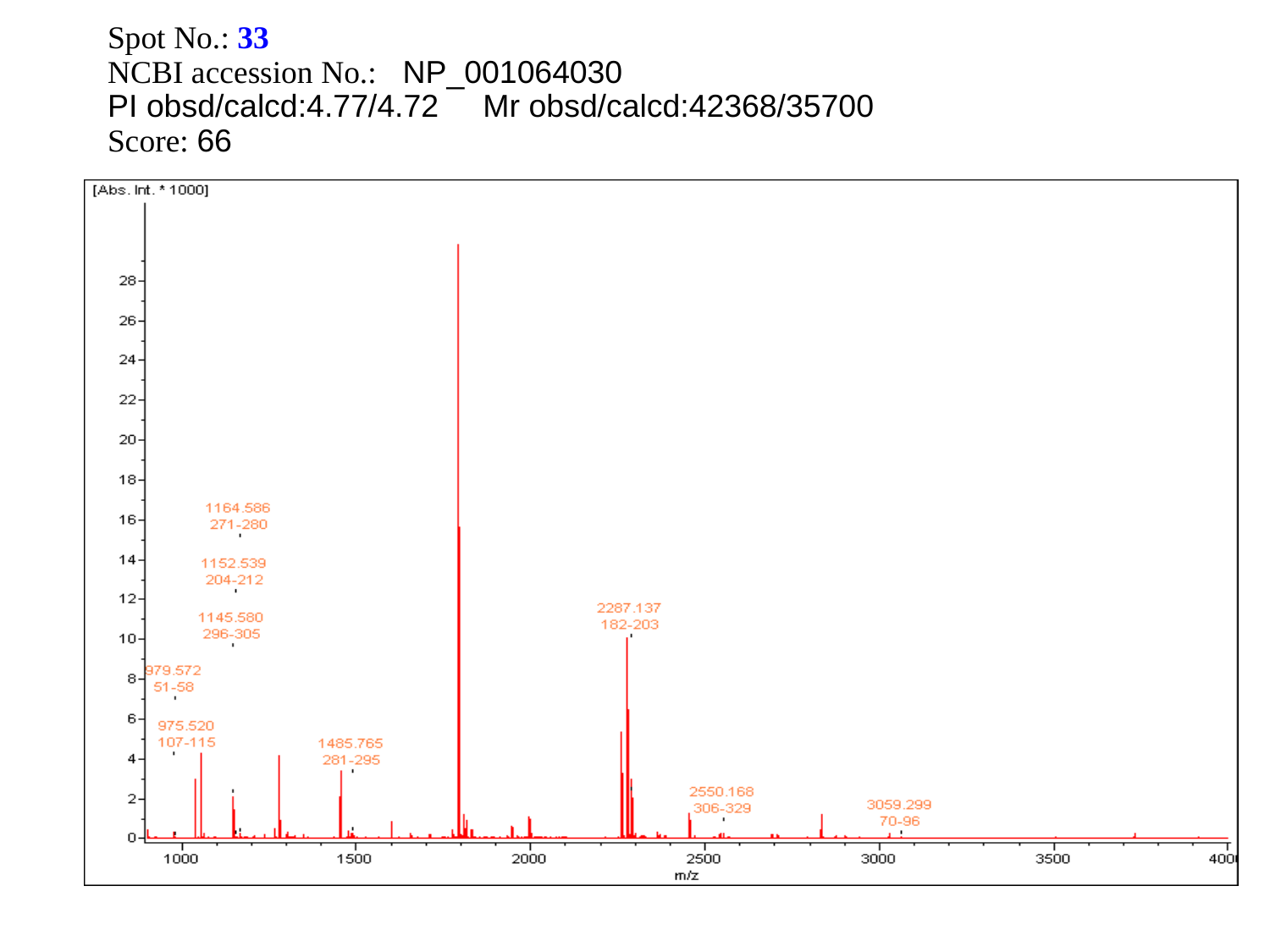

Spot No.: 33
NCBI accession No.: NP_001064030
PI obsd/calcd:4.77/4.72 Mr obsd/calcd:42368/35700
Score: 66

## Slide 57
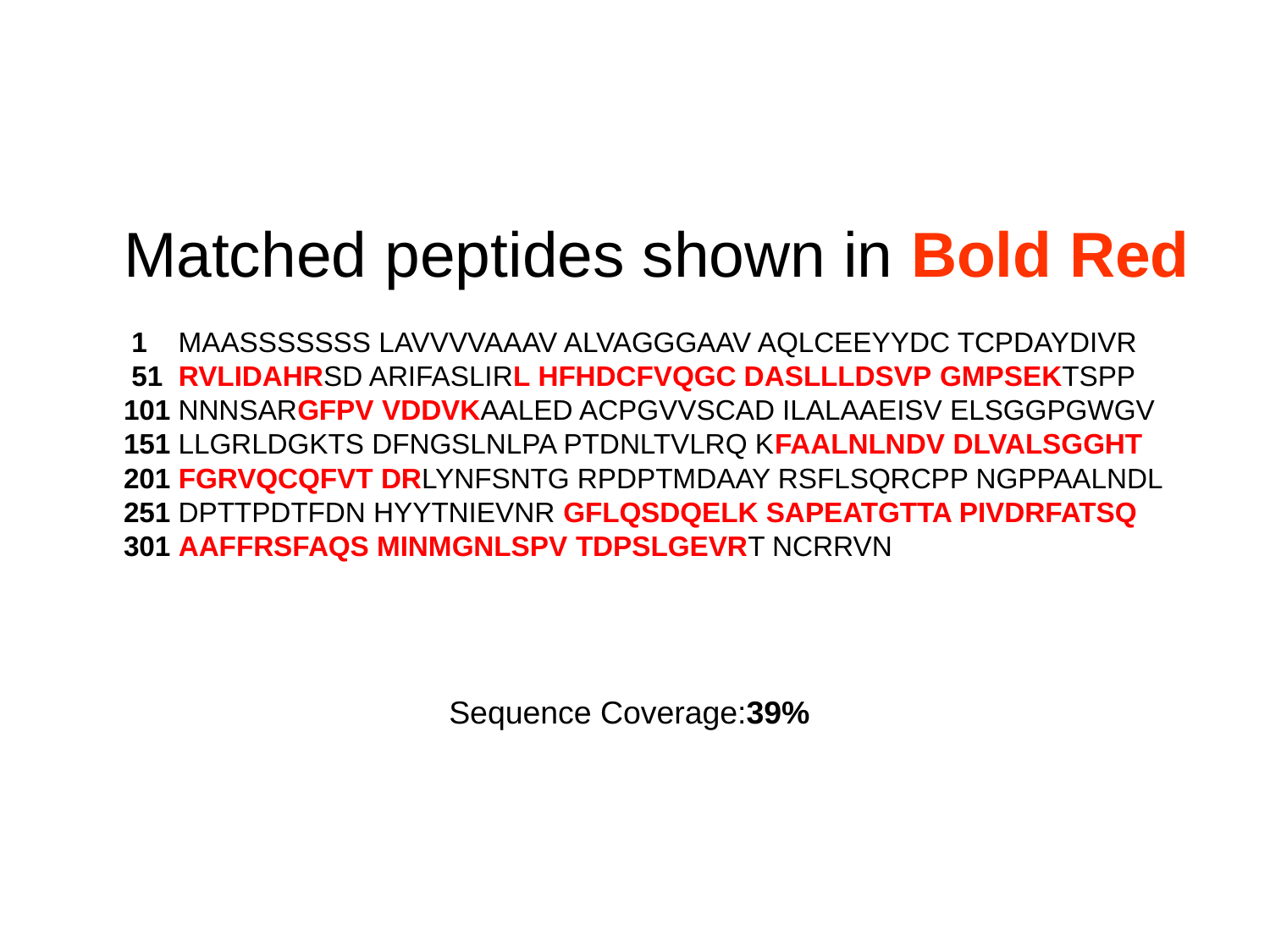

Matched peptides shown in Bold Red
 1 MAASSSSSSS LAVVVVAAAV ALVAGGGAAV AQLCEEYYDC TCPDAYDIVR
 51 RVLIDAHRSD ARIFASLIRL HFHDCFVQGC DASLLLDSVP GMPSEKTSPP
101 NNNSARGFPV VDDVKAALED ACPGVVSCAD ILALAAEISV ELSGGPGWGV
151 LLGRLDGKTS DFNGSLNLPA PTDNLTVLRQ KFAALNLNDV DLVALSGGHT
201 FGRVQCQFVT DRLYNFSNTG RPDPTMDAAY RSFLSQRCPP NGPPAALNDL
251 DPTTPDTFDN HYYTNIEVNR GFLQSDQELK SAPEATGTTA PIVDRFATSQ
301 AAFFRSFAQS MINMGNLSPV TDPSLGEVRT NCRRVN
# Sequence Coverage:39%

## Slide 58
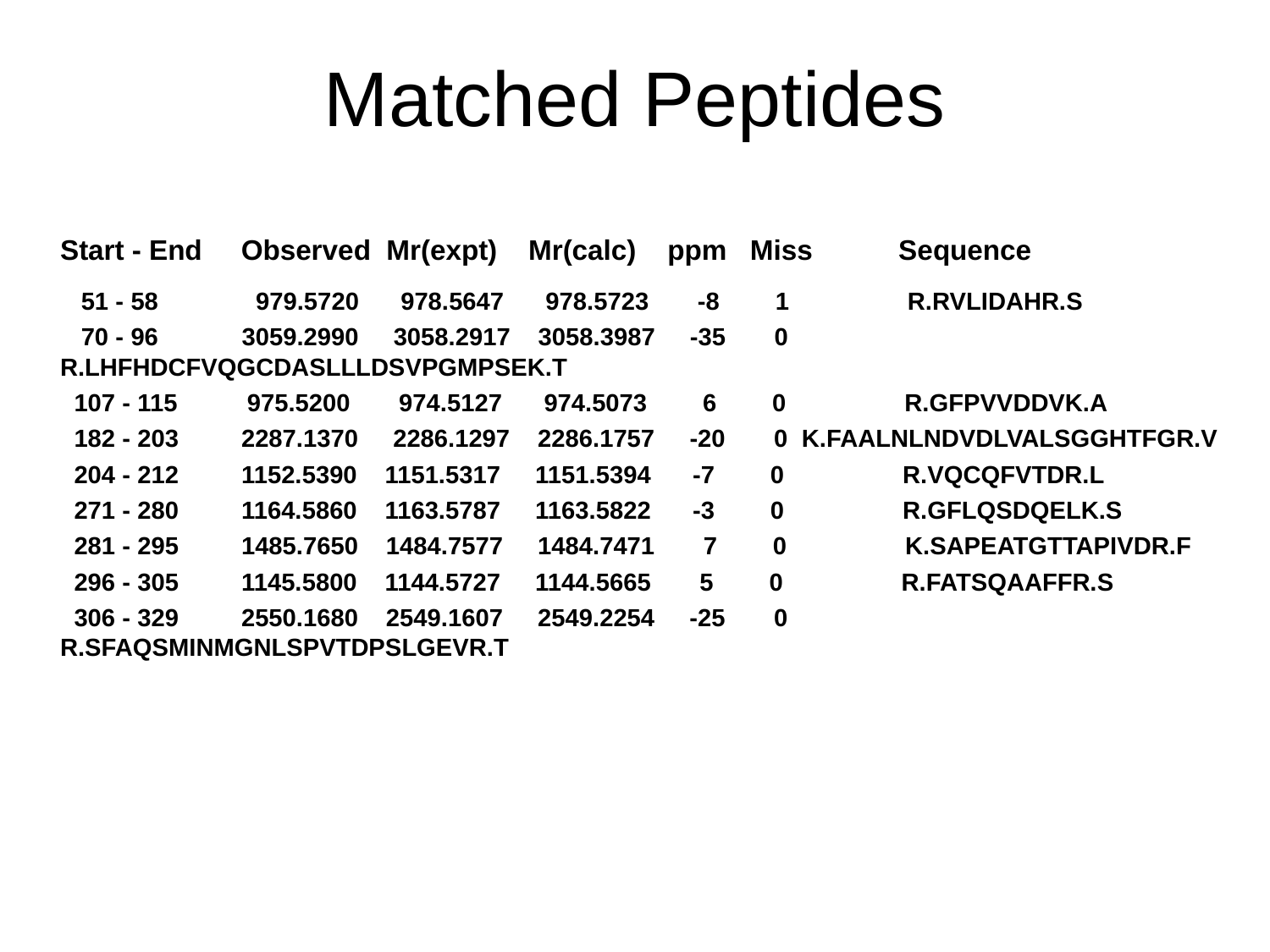

# Matched Peptides
Start - End Observed Mr(expt) Mr(calc) ppm Miss Sequence
 51 - 58 979.5720 978.5647 978.5723 -8 1 R.RVLIDAHR.S
 70 - 96 3059.2990 3058.2917 3058.3987 -35 0 R.LHFHDCFVQGCDASLLLDSVPGMPSEK.T
 107 - 115 975.5200 974.5127 974.5073 6 0 R.GFPVVDDVK.A
 182 - 203 2287.1370 2286.1297 2286.1757 -20 0 K.FAALNLNDVDLVALSGGHTFGR.V
 204 - 212 1152.5390 1151.5317 1151.5394 -7 0 R.VQCQFVTDR.L
 271 - 280 1164.5860 1163.5787 1163.5822 -3 0 R.GFLQSDQELK.S
 281 - 295 1485.7650 1484.7577 1484.7471 7 0 K.SAPEATGTTAPIVDR.F
 296 - 305 1145.5800 1144.5727 1144.5665 5 0 R.FATSQAAFFR.S
 306 - 329 2550.1680 2549.1607 2549.2254 -25 0 R.SFAQSMINMGNLSPVTDPSLGEVR.T

## Slide 59
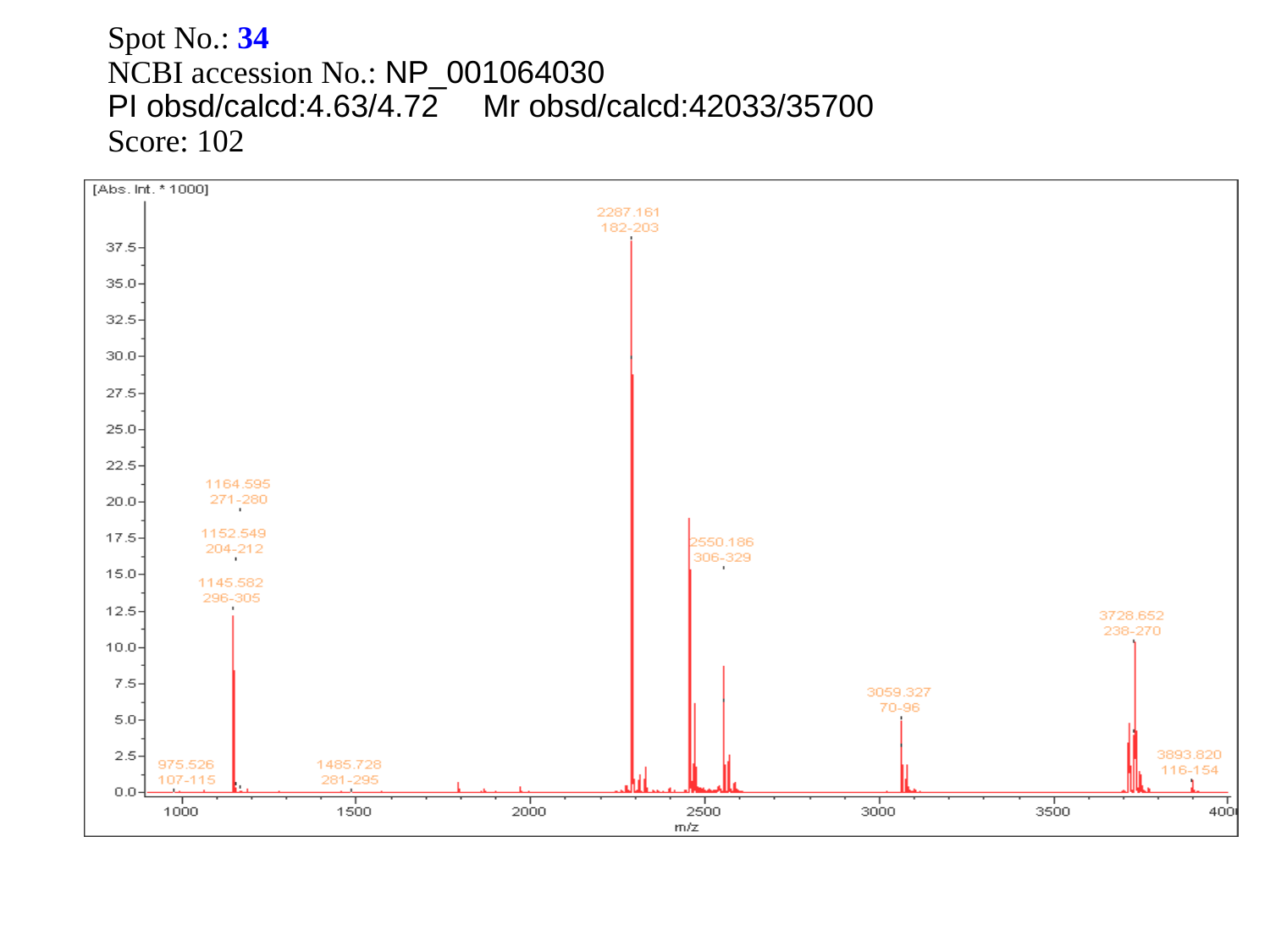

Spot No.: 34
NCBI accession No.: NP_001064030
PI obsd/calcd:4.63/4.72 Mr obsd/calcd:42033/35700
Score: 102

## Slide 60
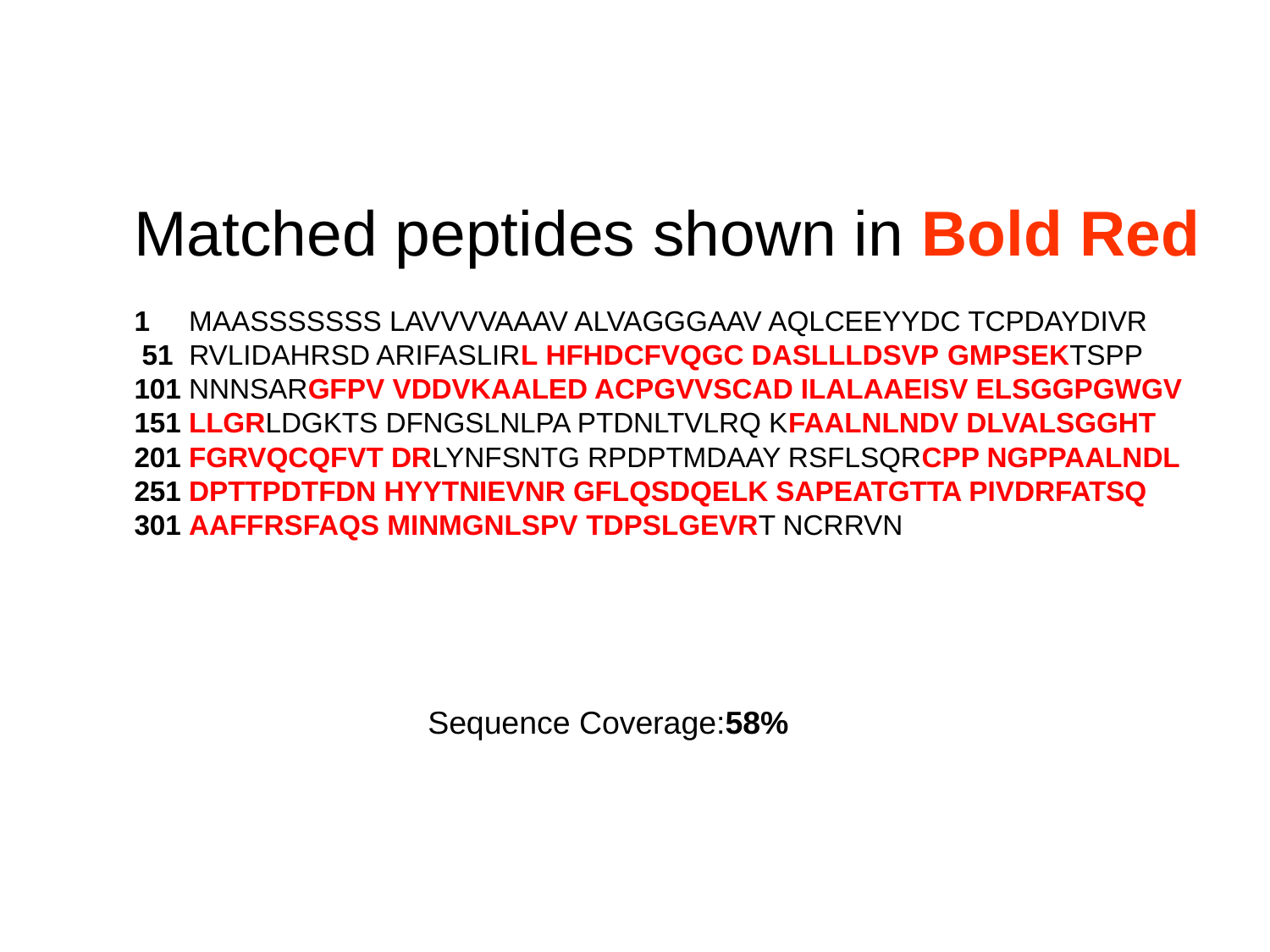

Matched peptides shown in Bold Red
1 MAASSSSSSS LAVVVVAAAV ALVAGGGAAV AQLCEEYYDC TCPDAYDIVR
 51 RVLIDAHRSD ARIFASLIRL HFHDCFVQGC DASLLLDSVP GMPSEKTSPP
101 NNNSARGFPV VDDVKAALED ACPGVVSCAD ILALAAEISV ELSGGPGWGV
151 LLGRLDGKTS DFNGSLNLPA PTDNLTVLRQ KFAALNLNDV DLVALSGGHT
201 FGRVQCQFVT DRLYNFSNTG RPDPTMDAAY RSFLSQRCPP NGPPAALNDL
251 DPTTPDTFDN HYYTNIEVNR GFLQSDQELK SAPEATGTTA PIVDRFATSQ
301 AAFFRSFAQS MINMGNLSPV TDPSLGEVRT NCRRVN
# Sequence Coverage:58%

## Slide 61
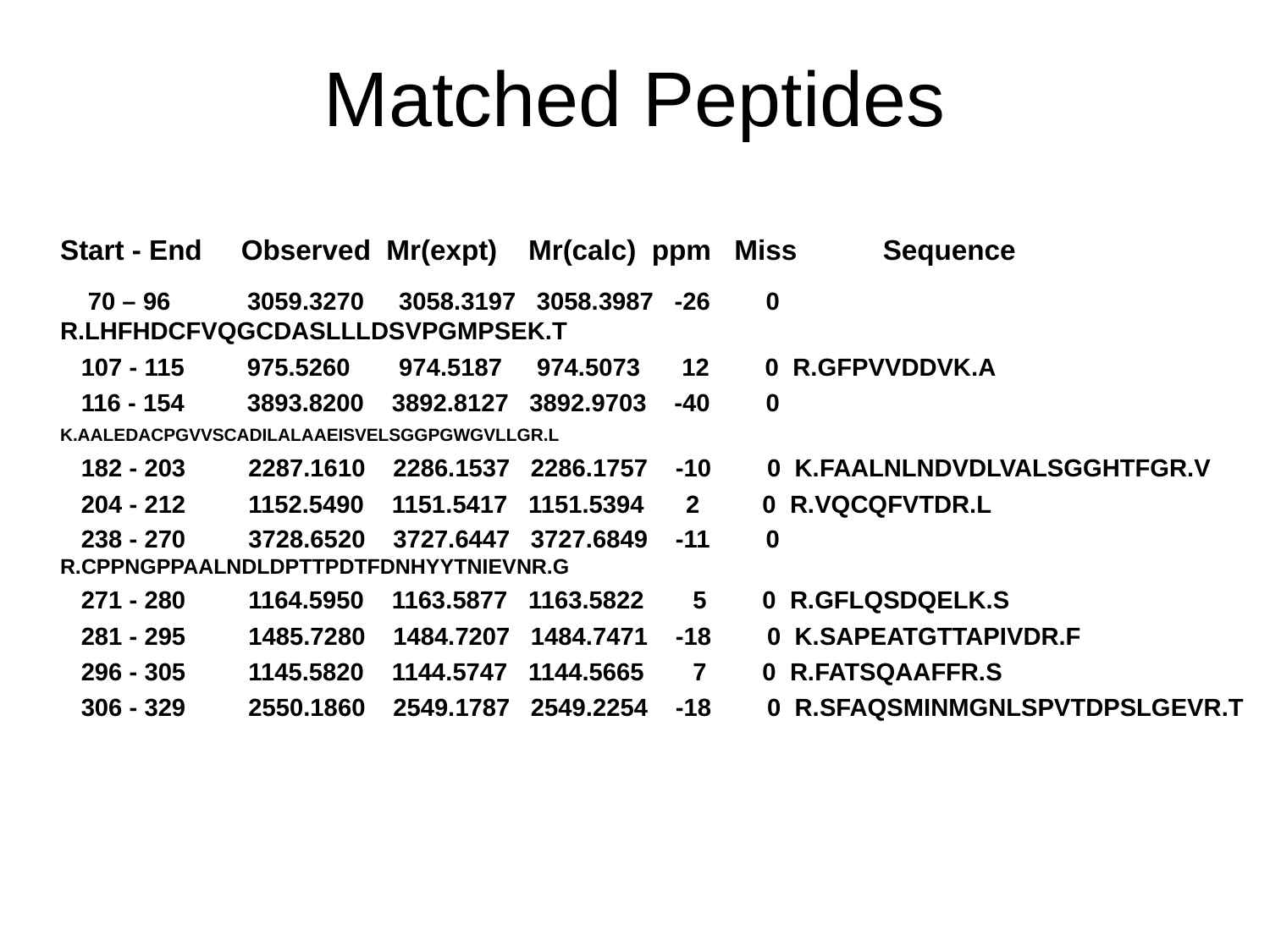

# Matched Peptides
Start - End Observed Mr(expt) Mr(calc) ppm Miss Sequence
 70 – 96 3059.3270 3058.3197 3058.3987 -26 0 R.LHFHDCFVQGCDASLLLDSVPGMPSEK.T
 107 - 115 975.5260 974.5187 974.5073 12 0 R.GFPVVDDVK.A
 116 - 154 3893.8200 3892.8127 3892.9703 -40 0 K.AALEDACPGVVSCADILALAAEISVELSGGPGWGVLLGR.L
 182 - 203 2287.1610 2286.1537 2286.1757 -10 0 K.FAALNLNDVDLVALSGGHTFGR.V
 204 - 212 1152.5490 1151.5417 1151.5394 2 0 R.VQCQFVTDR.L
 238 - 270 3728.6520 3727.6447 3727.6849 -11 0 R.CPPNGPPAALNDLDPTTPDTFDNHYYTNIEVNR.G
 271 - 280 1164.5950 1163.5877 1163.5822 5 0 R.GFLQSDQELK.S
 281 - 295 1485.7280 1484.7207 1484.7471 -18 0 K.SAPEATGTTAPIVDR.F
 296 - 305 1145.5820 1144.5747 1144.5665 7 0 R.FATSQAAFFR.S
 306 - 329 2550.1860 2549.1787 2549.2254 -18 0 R.SFAQSMINMGNLSPVTDPSLGEVR.T

## Slide 62
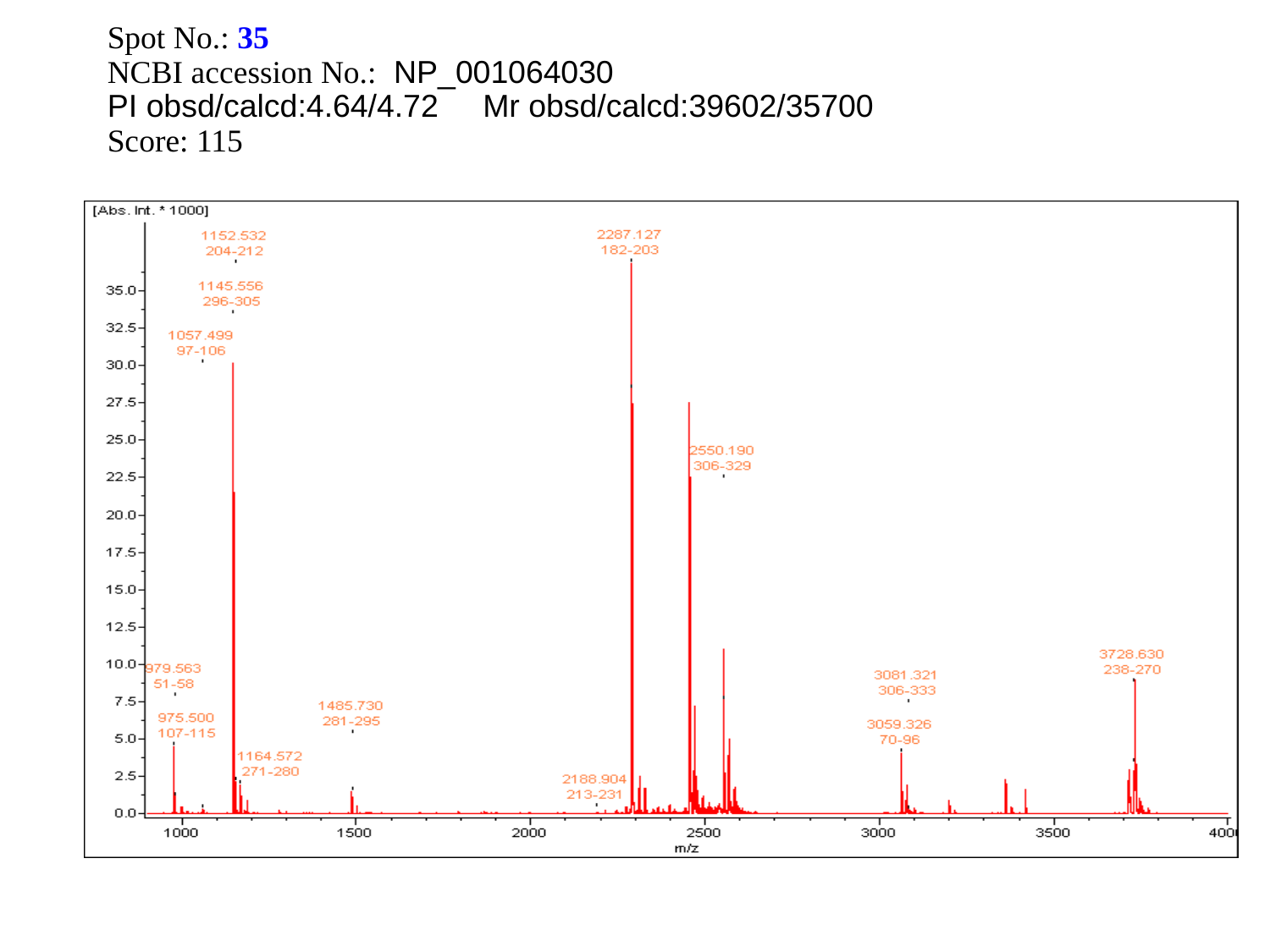

Spot No.: 35
NCBI accession No.: NP_001064030
PI obsd/calcd:4.64/4.72 Mr obsd/calcd:39602/35700
Score: 115

## Slide 63
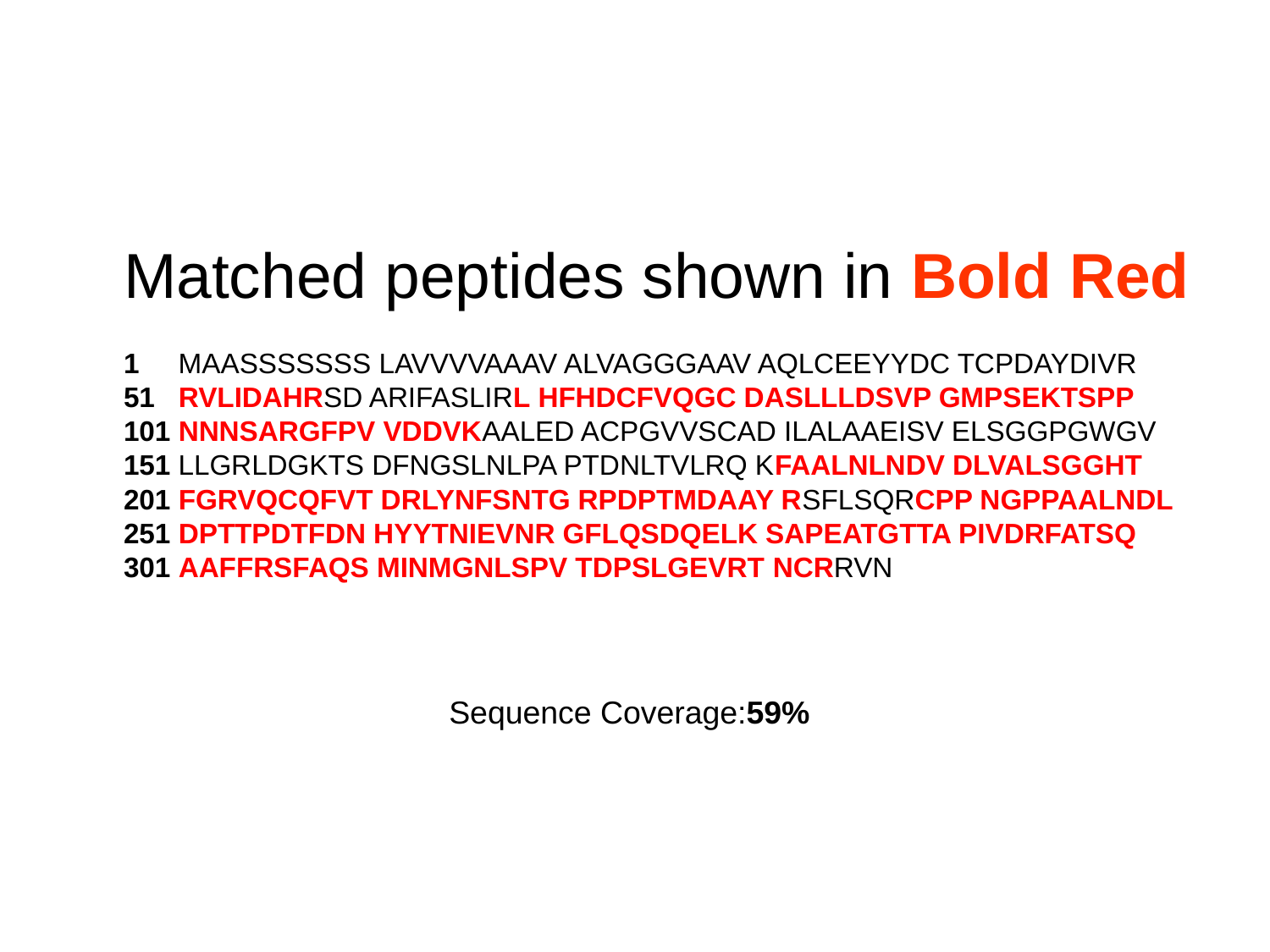

Matched peptides shown in Bold Red
1 MAASSSSSSS LAVVVVAAAV ALVAGGGAAV AQLCEEYYDC TCPDAYDIVR
51 RVLIDAHRSD ARIFASLIRL HFHDCFVQGC DASLLLDSVP GMPSEKTSPP
101 NNNSARGFPV VDDVKAALED ACPGVVSCAD ILALAAEISV ELSGGPGWGV
151 LLGRLDGKTS DFNGSLNLPA PTDNLTVLRQ KFAALNLNDV DLVALSGGHT
201 FGRVQCQFVT DRLYNFSNTG RPDPTMDAAY RSFLSQRCPP NGPPAALNDL
251 DPTTPDTFDN HYYTNIEVNR GFLQSDQELK SAPEATGTTA PIVDRFATSQ
301 AAFFRSFAQS MINMGNLSPV TDPSLGEVRT NCRRVN
# Sequence Coverage:59%

## Slide 64
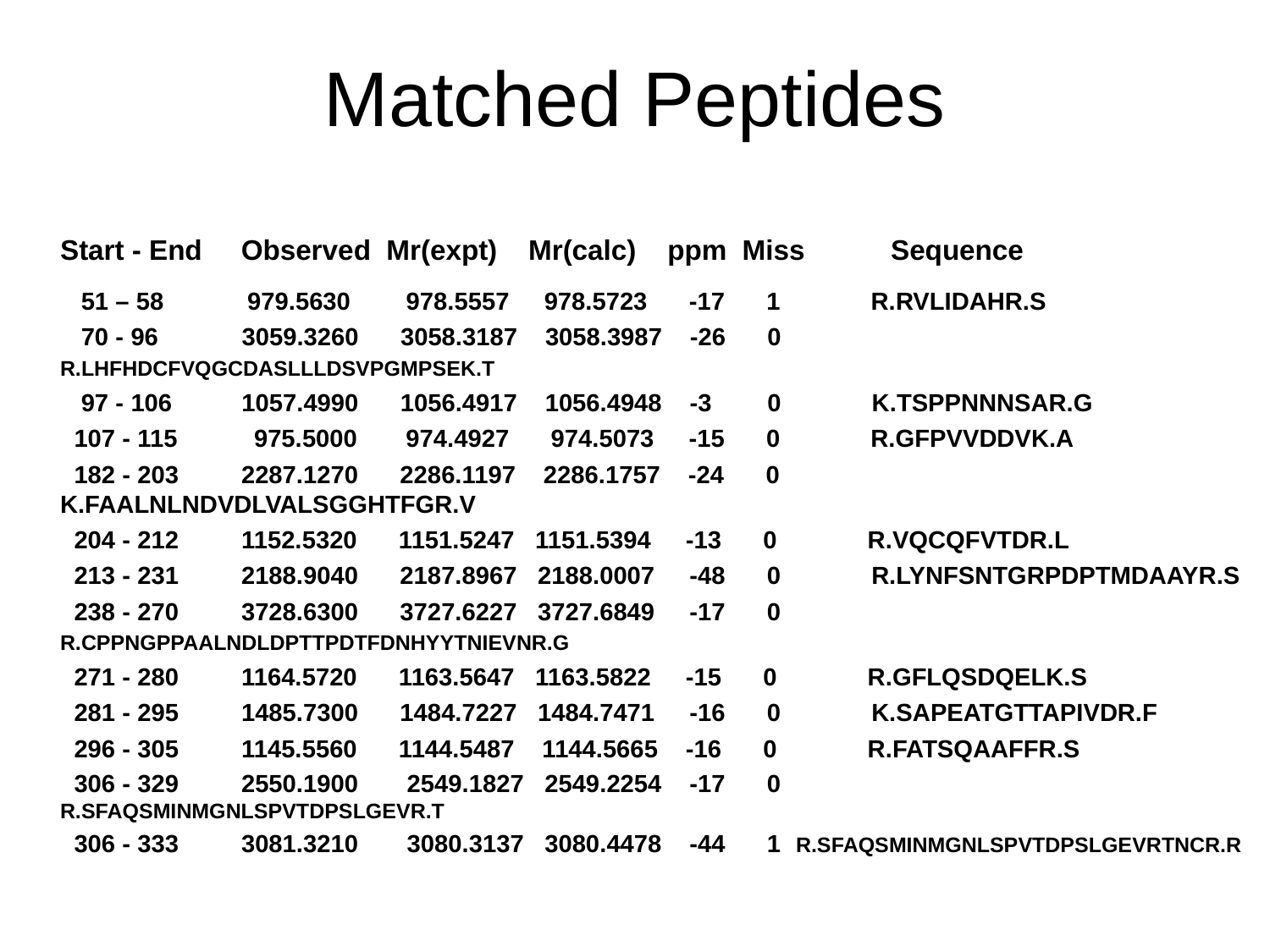

# Matched Peptides
Start - End Observed Mr(expt) Mr(calc) ppm Miss Sequence
 51 – 58 979.5630 978.5557 978.5723 -17 1 R.RVLIDAHR.S
 70 - 96 3059.3260 3058.3187 3058.3987 -26 0 R.LHFHDCFVQGCDASLLLDSVPGMPSEK.T
 97 - 106 1057.4990 1056.4917 1056.4948 -3 0 K.TSPPNNNSAR.G
 107 - 115 975.5000 974.4927 974.5073 -15 0 R.GFPVVDDVK.A
 182 - 203 2287.1270 2286.1197 2286.1757 -24 0 K.FAALNLNDVDLVALSGGHTFGR.V
 204 - 212 1152.5320 1151.5247 1151.5394 -13 0 R.VQCQFVTDR.L
 213 - 231 2188.9040 2187.8967 2188.0007 -48 0 R.LYNFSNTGRPDPTMDAAYR.S
 238 - 270 3728.6300 3727.6227 3727.6849 -17 0 R.CPPNGPPAALNDLDPTTPDTFDNHYYTNIEVNR.G
 271 - 280 1164.5720 1163.5647 1163.5822 -15 0 R.GFLQSDQELK.S
 281 - 295 1485.7300 1484.7227 1484.7471 -16 0 K.SAPEATGTTAPIVDR.F
 296 - 305 1145.5560 1144.5487 1144.5665 -16 0 R.FATSQAAFFR.S
 306 - 329 2550.1900 2549.1827 2549.2254 -17 0 R.SFAQSMINMGNLSPVTDPSLGEVR.T
 306 - 333 3081.3210 3080.3137 3080.4478 -44 1 R.SFAQSMINMGNLSPVTDPSLGEVRTNCR.R

## Slide 65
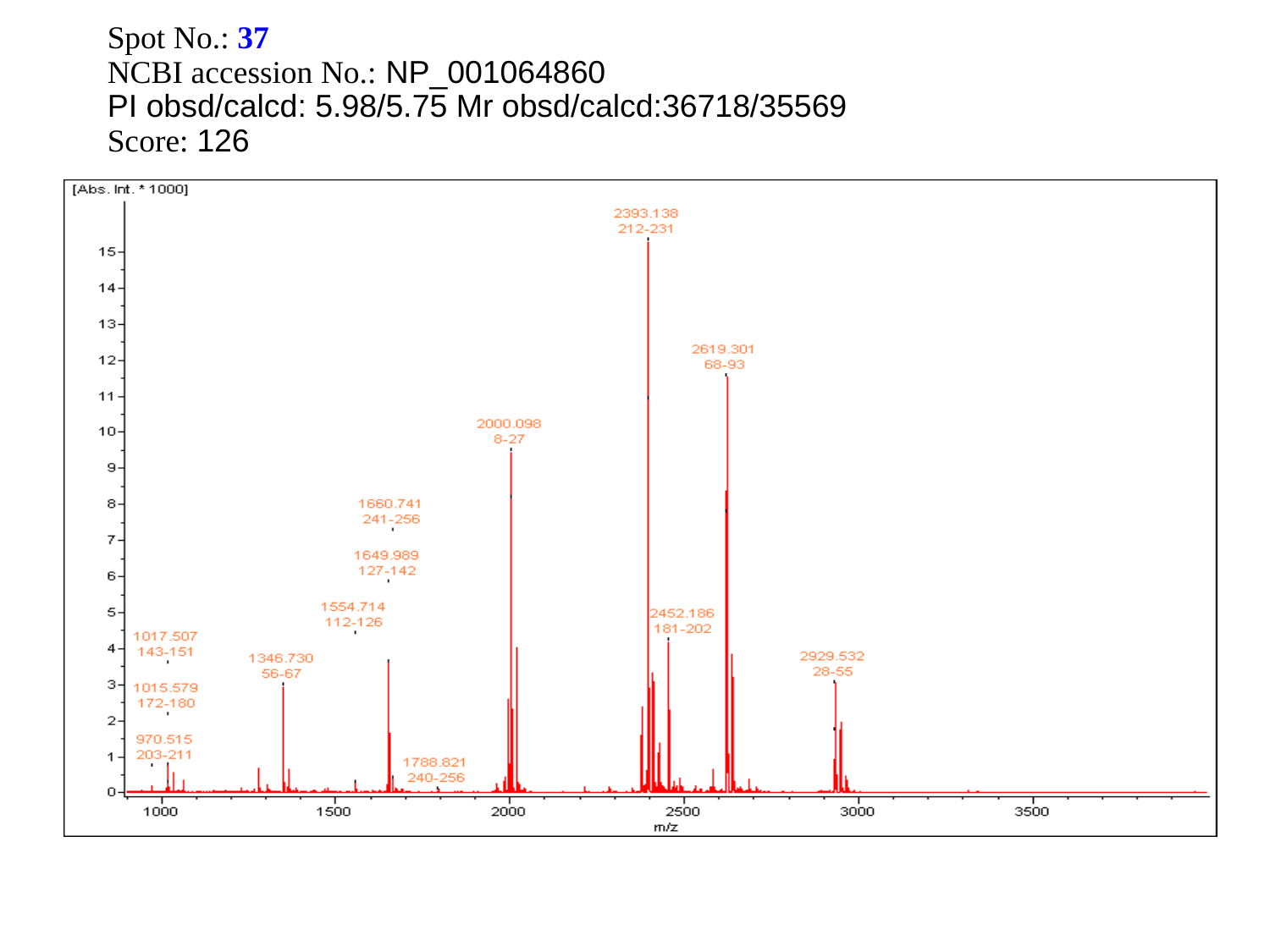

Spot No.: 37
NCBI accession No.: NP_001064860
PI obsd/calcd: 5.98/5.75 Mr obsd/calcd:36718/35569
Score: 126

## Slide 66
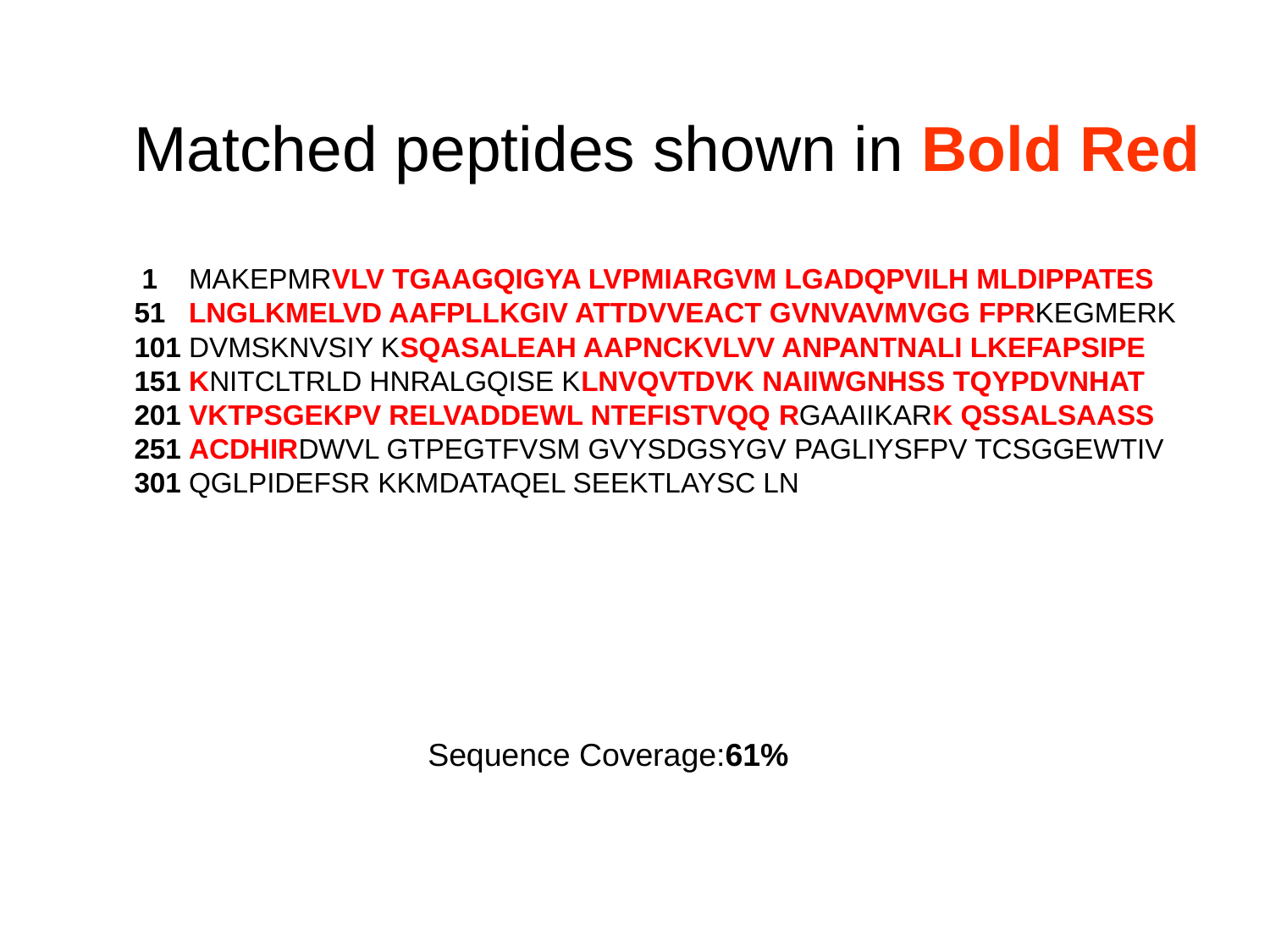

Matched peptides shown in Bold Red
 1 MAKEPMRVLV TGAAGQIGYA LVPMIARGVM LGADQPVILH MLDIPPATES
51 LNGLKMELVD AAFPLLKGIV ATTDVVEACT GVNVAVMVGG FPRKEGMERK
101 DVMSKNVSIY KSQASALEAH AAPNCKVLVV ANPANTNALI LKEFAPSIPE
151 KNITCLTRLD HNRALGQISE KLNVQVTDVK NAIIWGNHSS TQYPDVNHAT
201 VKTPSGEKPV RELVADDEWL NTEFISTVQQ RGAAIIKARK QSSALSAASS
251 ACDHIRDWVL GTPEGTFVSM GVYSDGSYGV PAGLIYSFPV TCSGGEWTIV
301 QGLPIDEFSR KKMDATAQEL SEEKTLAYSC LN
# Sequence Coverage:61%

## Slide 67
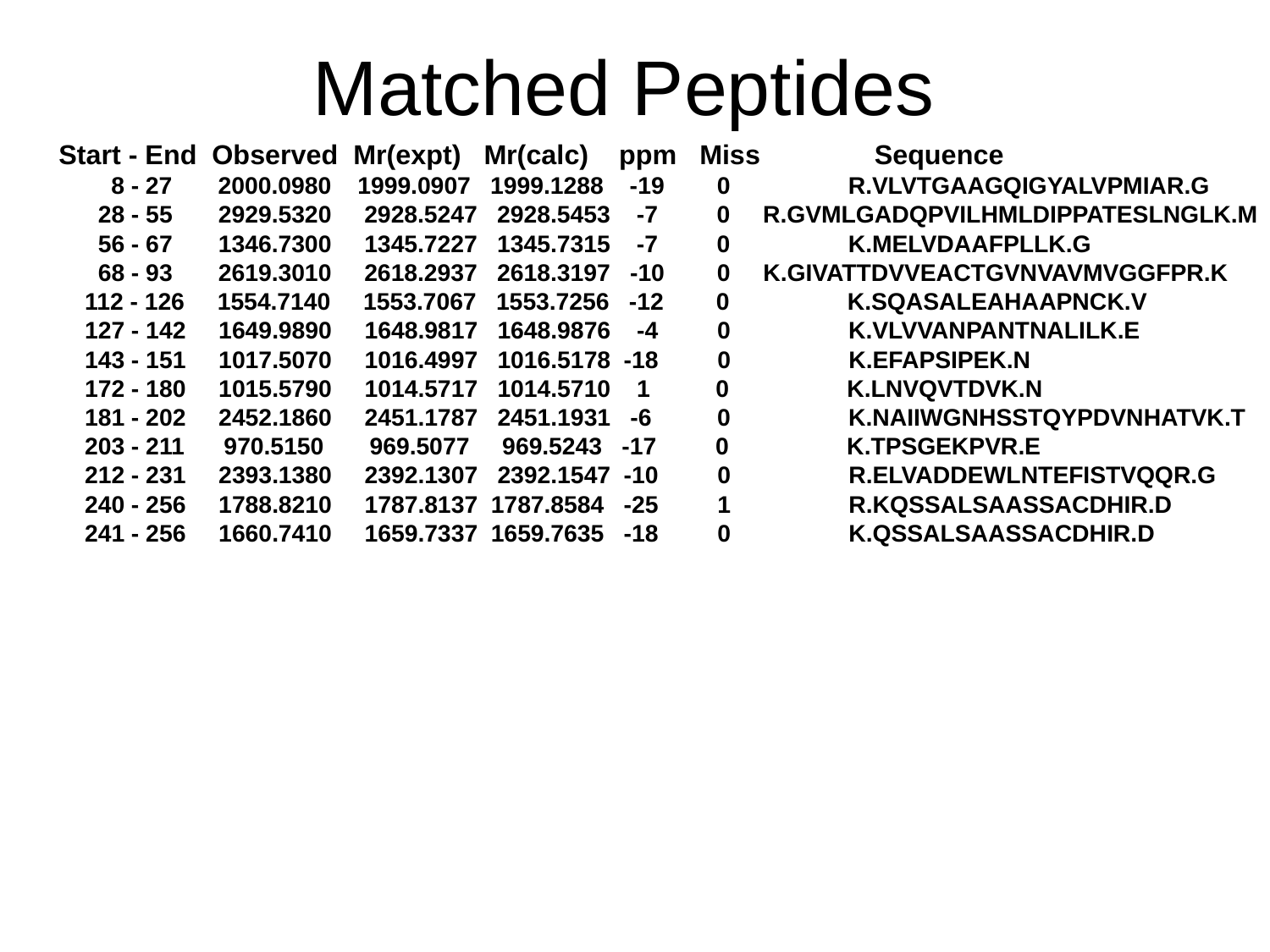

# Matched Peptides
Start - End Observed Mr(expt) Mr(calc) ppm Miss Sequence
 8 - 27 2000.0980 1999.0907 1999.1288 -19 0 R.VLVTGAAGQIGYALVPMIAR.G
 28 - 55 2929.5320 2928.5247 2928.5453 -7 0 R.GVMLGADQPVILHMLDIPPATESLNGLK.M
 56 - 67 1346.7300 1345.7227 1345.7315 -7 0 K.MELVDAAFPLLK.G
 68 - 93 2619.3010 2618.2937 2618.3197 -10 0 K.GIVATTDVVEACTGVNVAVMVGGFPR.K
 112 - 126 1554.7140 1553.7067 1553.7256 -12 0 K.SQASALEAHAAPNCK.V
 127 - 142 1649.9890 1648.9817 1648.9876 -4 0 K.VLVVANPANTNALILK.E
 143 - 151 1017.5070 1016.4997 1016.5178 -18 0 K.EFAPSIPEK.N
 172 - 180 1015.5790 1014.5717 1014.5710 1 0 K.LNVQVTDVK.N
 181 - 202 2452.1860 2451.1787 2451.1931 -6 0 K.NAIIWGNHSSTQYPDVNHATVK.T
 203 - 211 970.5150 969.5077 969.5243 -17 0 K.TPSGEKPVR.E
 212 - 231 2393.1380 2392.1307 2392.1547 -10 0 R.ELVADDEWLNTEFISTVQQR.G
 240 - 256 1788.8210 1787.8137 1787.8584 -25 1 R.KQSSALSAASSACDHIR.D
 241 - 256 1660.7410 1659.7337 1659.7635 -18 0 K.QSSALSAASSACDHIR.D

## Slide 68
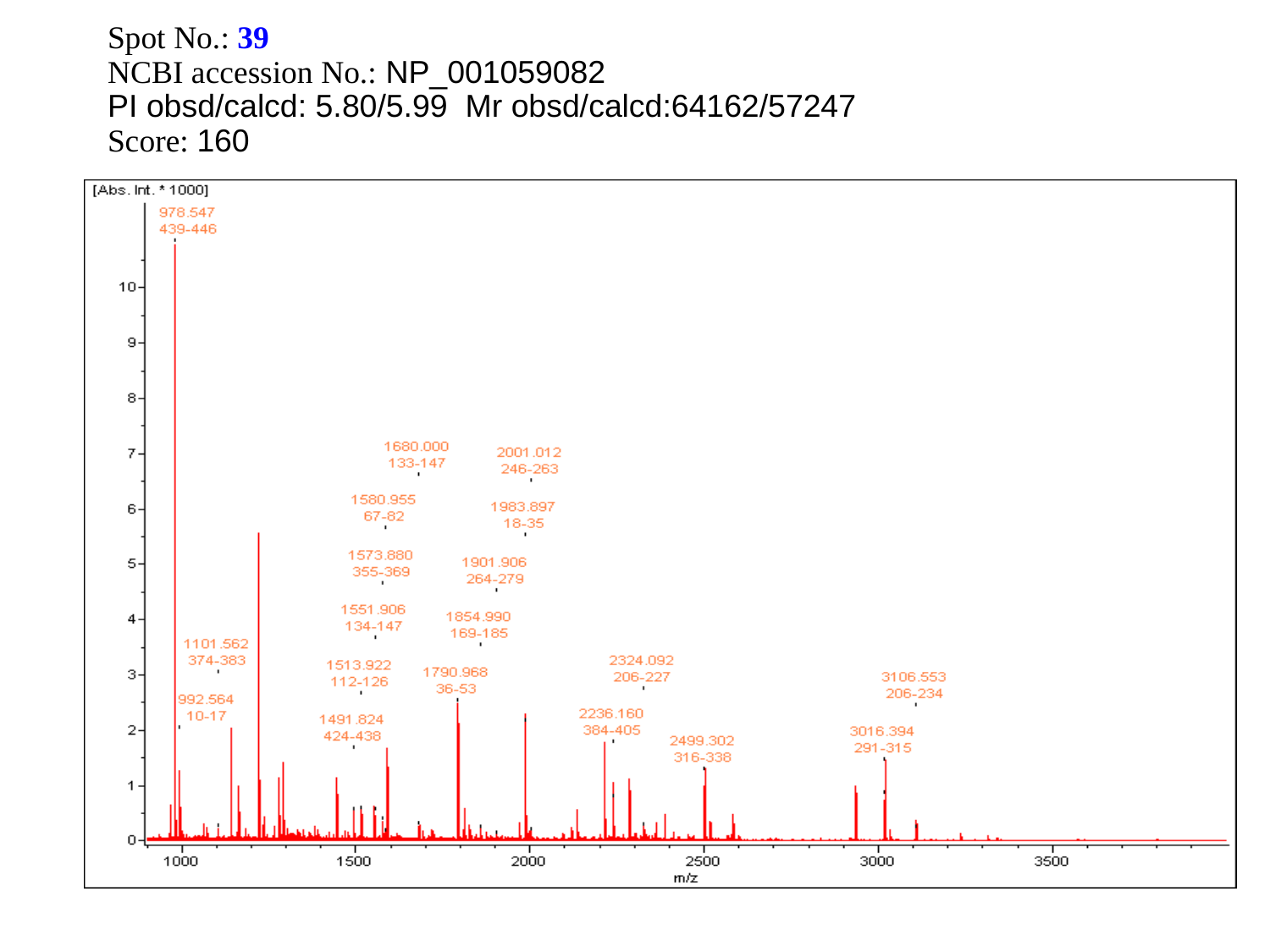

Spot No.: 39
NCBI accession No.: NP_001059082
PI obsd/calcd: 5.80/5.99 Mr obsd/calcd:64162/57247
Score: 160

## Slide 69
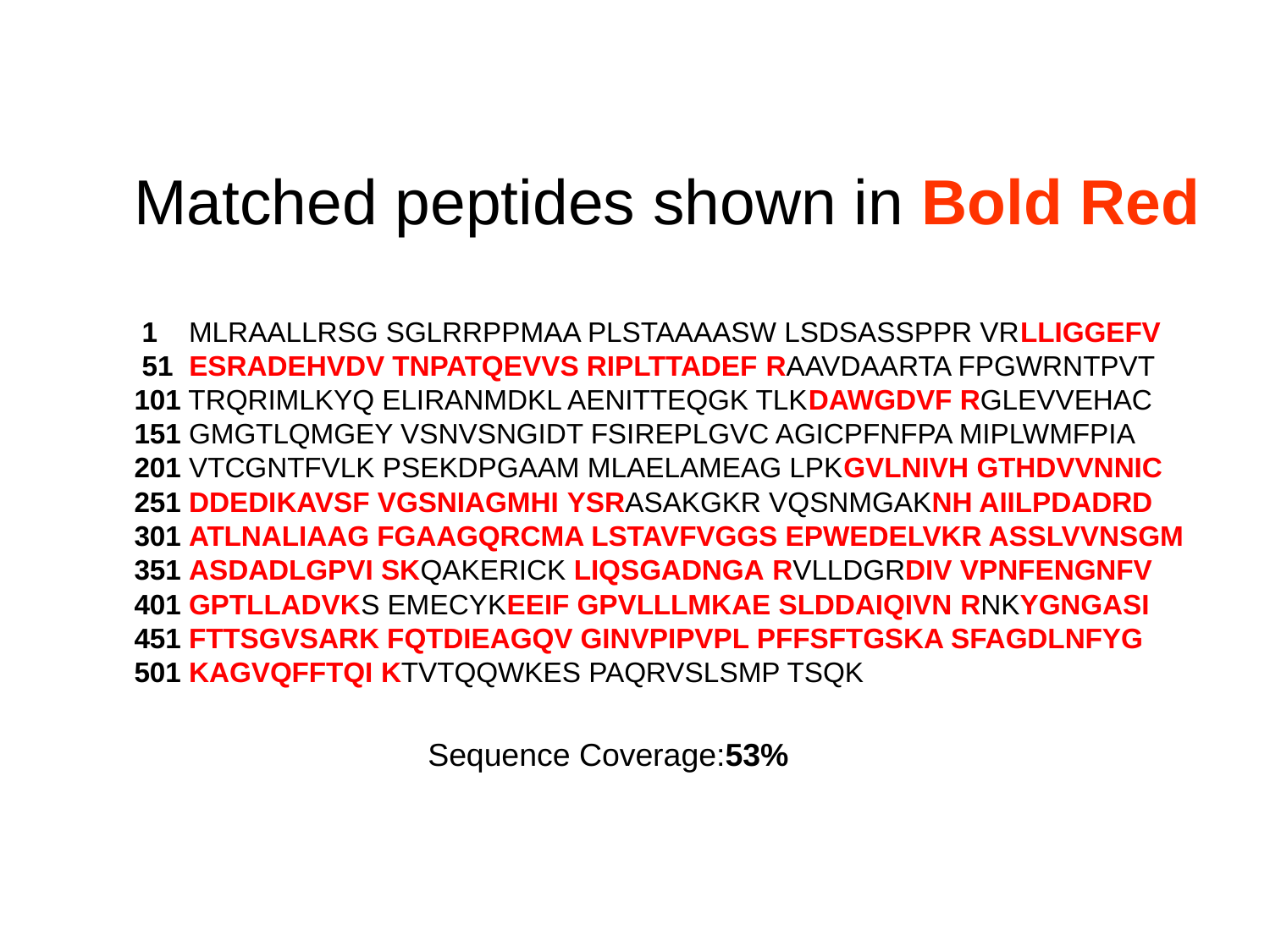

Matched peptides shown in Bold Red
 1 MLRAALLRSG SGLRRPPMAA PLSTAAAASW LSDSASSPPR VRLLIGGEFV
 51 ESRADEHVDV TNPATQEVVS RIPLTTADEF RAAVDAARTA FPGWRNTPVT
101 TRQRIMLKYQ ELIRANMDKL AENITTEQGK TLKDAWGDVF RGLEVVEHAC
151 GMGTLQMGEY VSNVSNGIDT FSIREPLGVC AGICPFNFPA MIPLWMFPIA
201 VTCGNTFVLK PSEKDPGAAM MLAELAMEAG LPKGVLNIVH GTHDVVNNIC
251 DDEDIKAVSF VGSNIAGMHI YSRASAKGKR VQSNMGAKNH AIILPDADRD
301 ATLNALIAAG FGAAGQRCMA LSTAVFVGGS EPWEDELVKR ASSLVVNSGM
351 ASDADLGPVI SKQAKERICK LIQSGADNGA RVLLDGRDIV VPNFENGNFV
401 GPTLLADVKS EMECYKEEIF GPVLLLMKAE SLDDAIQIVN RNKYGNGASI
451 FTTSGVSARK FQTDIEAGQV GINVPIPVPL PFFSFTGSKA SFAGDLNFYG
501 KAGVQFFTQI KTVTQQWKES PAQRVSLSMP TSQK
# Sequence Coverage:53%

## Slide 70
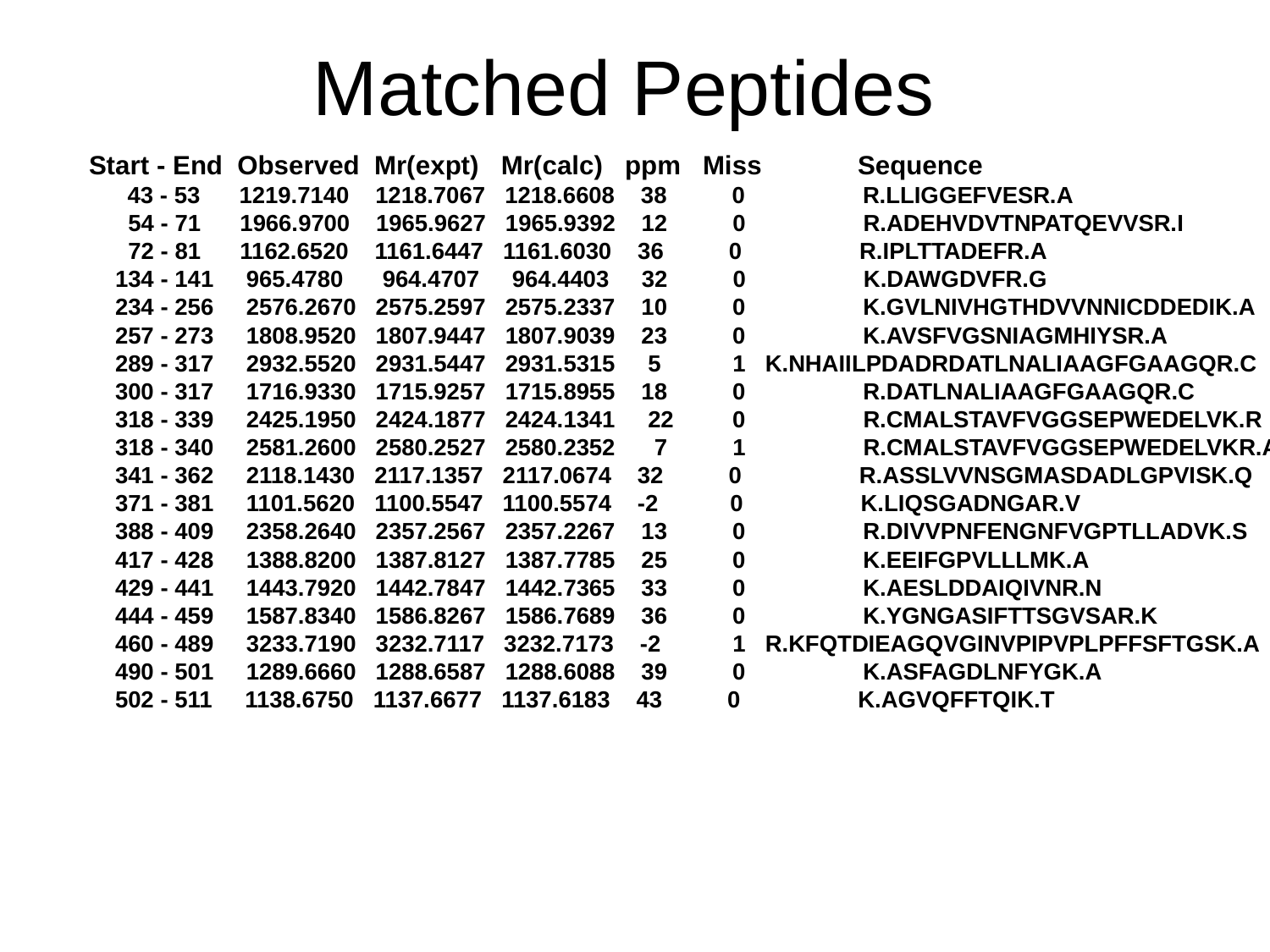

# Matched Peptides
Start - End Observed Mr(expt) Mr(calc) ppm Miss Sequence
 43 - 53 1219.7140 1218.7067 1218.6608 38 0 R.LLIGGEFVESR.A
 54 - 71 1966.9700 1965.9627 1965.9392 12 0 R.ADEHVDVTNPATQEVVSR.I
 72 - 81 1162.6520 1161.6447 1161.6030 36 0 R.IPLTTADEFR.A
 134 - 141 965.4780 964.4707 964.4403 32 0 K.DAWGDVFR.G
 234 - 256 2576.2670 2575.2597 2575.2337 10 0 K.GVLNIVHGTHDVVNNICDDEDIK.A
 257 - 273 1808.9520 1807.9447 1807.9039 23 0 K.AVSFVGSNIAGMHIYSR.A
 289 - 317 2932.5520 2931.5447 2931.5315 5 1 K.NHAIILPDADRDATLNALIAAGFGAAGQR.C
 300 - 317 1716.9330 1715.9257 1715.8955 18 0 R.DATLNALIAAGFGAAGQR.C
 318 - 339 2425.1950 2424.1877 2424.1341 22 0 R.CMALSTAVFVGGSEPWEDELVK.R
 318 - 340 2581.2600 2580.2527 2580.2352 7 1 R.CMALSTAVFVGGSEPWEDELVKR.A
 341 - 362 2118.1430 2117.1357 2117.0674 32 0 R.ASSLVVNSGMASDADLGPVISK.Q
 371 - 381 1101.5620 1100.5547 1100.5574 -2 0 K.LIQSGADNGAR.V
 388 - 409 2358.2640 2357.2567 2357.2267 13 0 R.DIVVPNFENGNFVGPTLLADVK.S
 417 - 428 1388.8200 1387.8127 1387.7785 25 0 K.EEIFGPVLLLMK.A
 429 - 441 1443.7920 1442.7847 1442.7365 33 0 K.AESLDDAIQIVNR.N
 444 - 459 1587.8340 1586.8267 1586.7689 36 0 K.YGNGASIFTTSGVSAR.K
 460 - 489 3233.7190 3232.7117 3232.7173 -2 1 R.KFQTDIEAGQVGINVPIPVPLPFFSFTGSK.A
 490 - 501 1289.6660 1288.6587 1288.6088 39 0 K.ASFAGDLNFYGK.A
 502 - 511 1138.6750 1137.6677 1137.6183 43 0 K.AGVQFFTQIK.T

## Slide 71
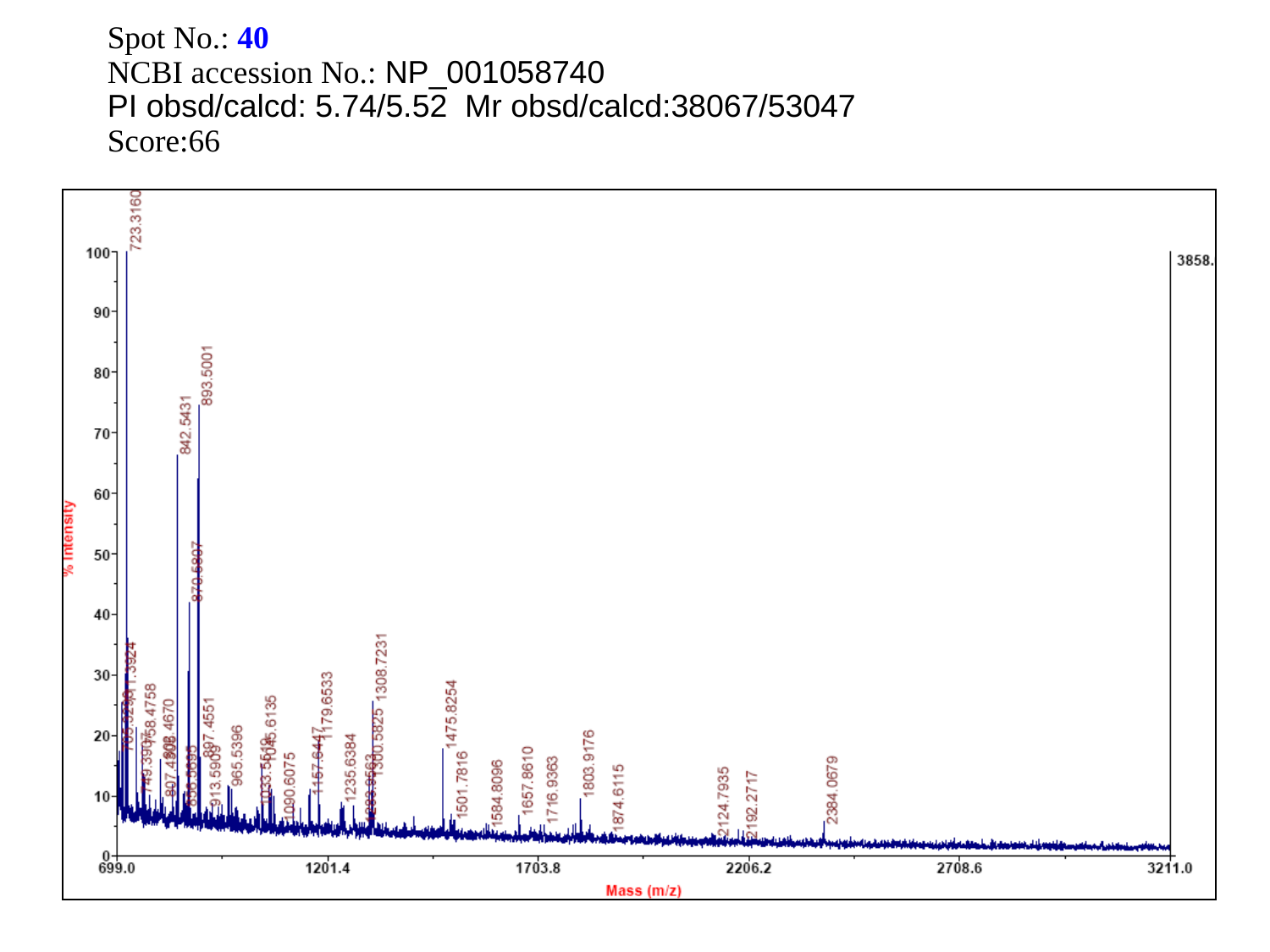

Spot No.: 40
NCBI accession No.: NP_001058740
PI obsd/calcd: 5.74/5.52 Mr obsd/calcd:38067/53047
Score:66

## Slide 72
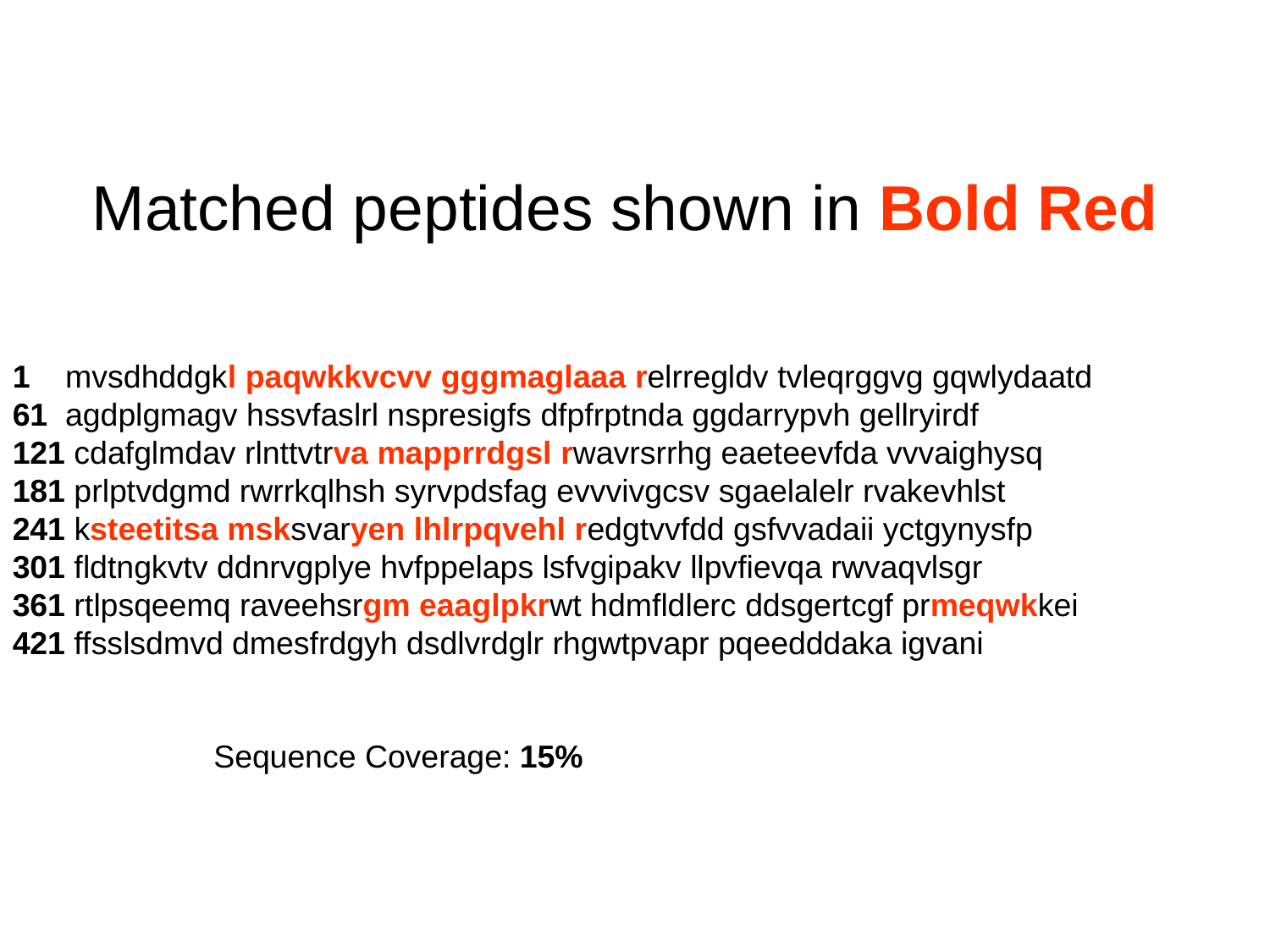

#
Matched peptides shown in Bold Red
1 mvsdhddgkl paqwkkvcvv gggmaglaaa relrregldv tvleqrggvg gqwlydaatd
61 agdplgmagv hssvfaslrl nspresigfs dfpfrptnda ggdarrypvh gellryirdf
121 cdafglmdav rlnttvtrva mapprrdgsl rwavrsrrhg eaeteevfda vvvaighysq
181 prlptvdgmd rwrrkqlhsh syrvpdsfag evvvivgcsv sgaelalelr rvakevhlst
241 ksteetitsa msksvaryen lhlrpqvehl redgtvvfdd gsfvvadaii yctgynysfp
301 fldtngkvtv ddnrvgplye hvfppelaps lsfvgipakv llpvfievqa rwvaqvlsgr
361 rtlpsqeemq raveehsrgm eaaglpkrwt hdmfldlerc ddsgertcgf prmeqwkkei
421 ffsslsdmvd dmesfrdgyh dsdlvrdglr rhgwtpvapr pqeedddaka igvani
Sequence Coverage: 15%

## Slide 73
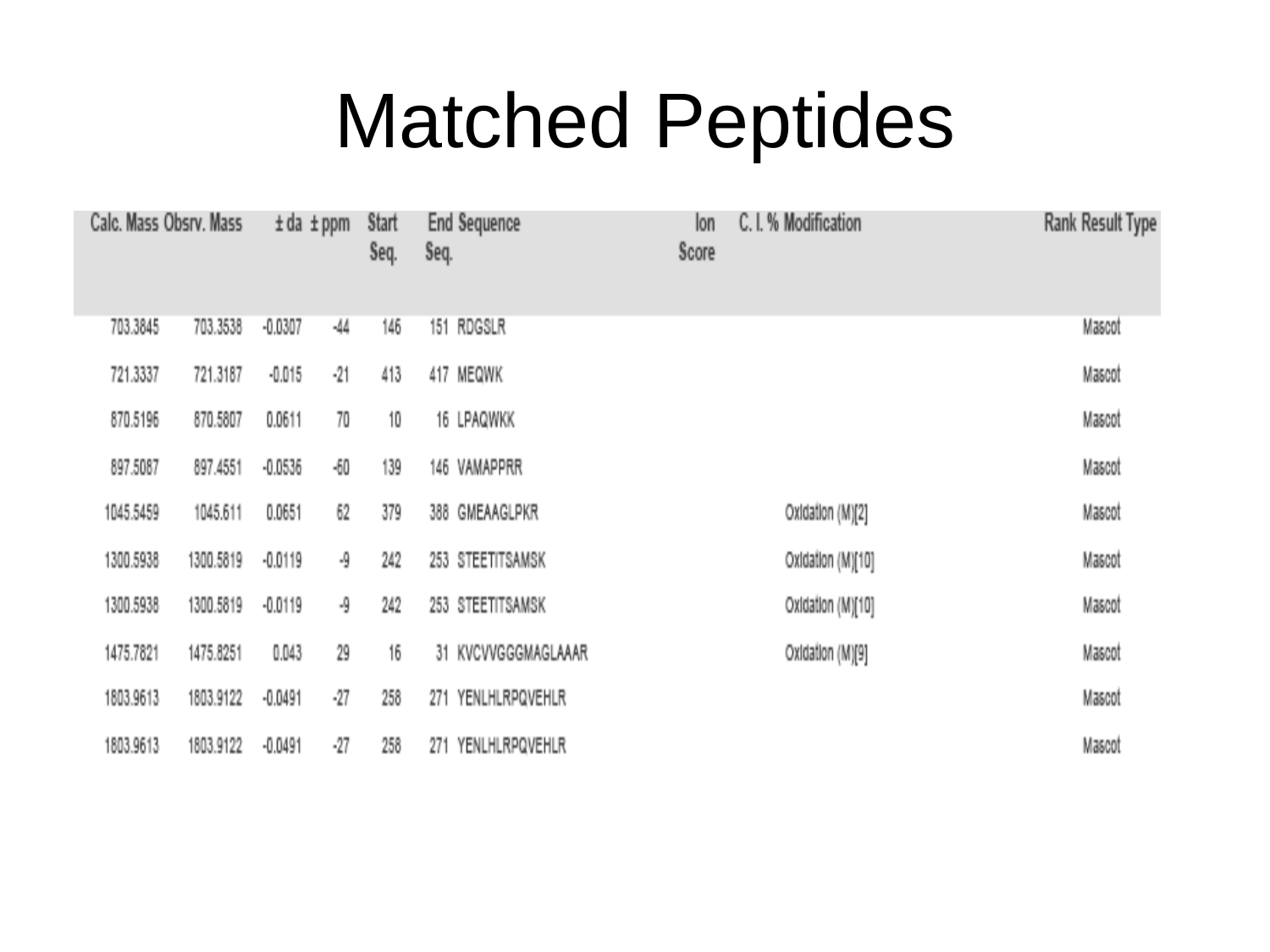

# Matched Peptides

## Slide 74
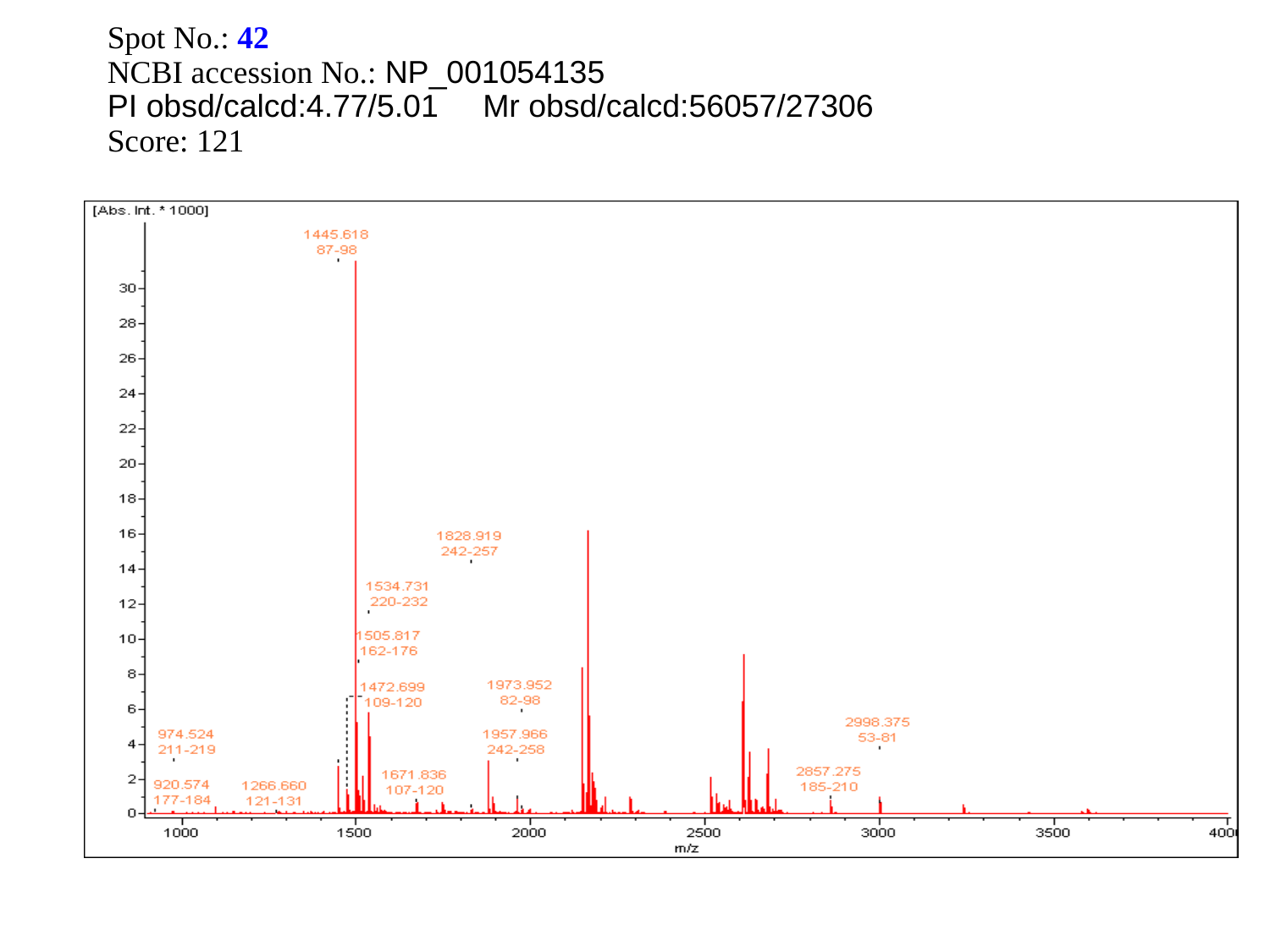

Spot No.: 42
NCBI accession No.: NP_001054135
PI obsd/calcd:4.77/5.01 Mr obsd/calcd:56057/27306
Score: 121

## Slide 75
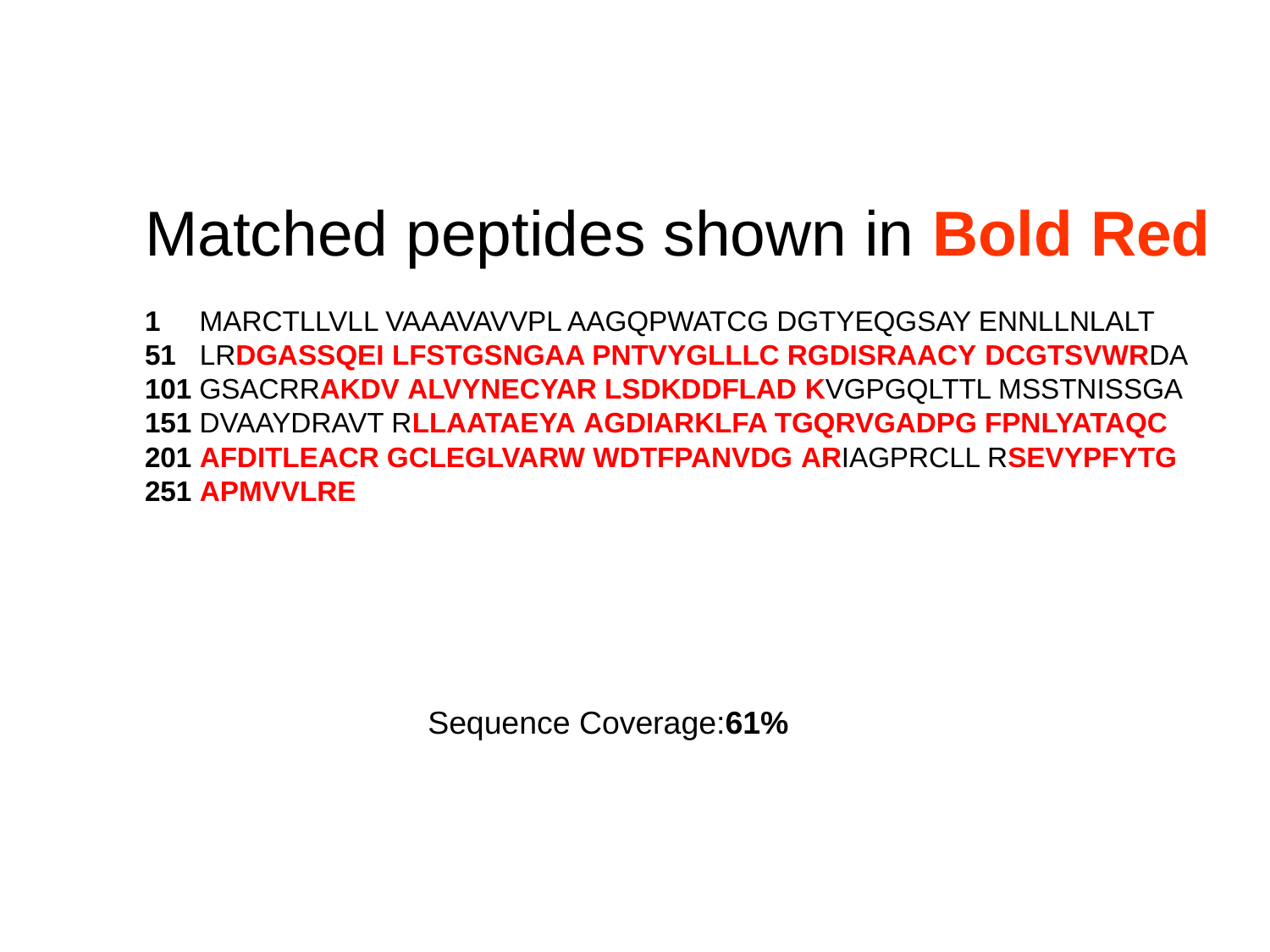

Matched peptides shown in Bold Red
1 MARCTLLVLL VAAAVAVVPL AAGQPWATCG DGTYEQGSAY ENNLLNLALT
51 LRDGASSQEI LFSTGSNGAA PNTVYGLLLC RGDISRAACY DCGTSVWRDA
101 GSACRRAKDV ALVYNECYAR LSDKDDFLAD KVGPGQLTTL MSSTNISSGA
151 DVAAYDRAVT RLLAATAEYA AGDIARKLFA TGQRVGADPG FPNLYATAQC
201 AFDITLEACR GCLEGLVARW WDTFPANVDG ARIAGPRCLL RSEVYPFYTG
251 APMVVLRE
# Sequence Coverage:61%

## Slide 76
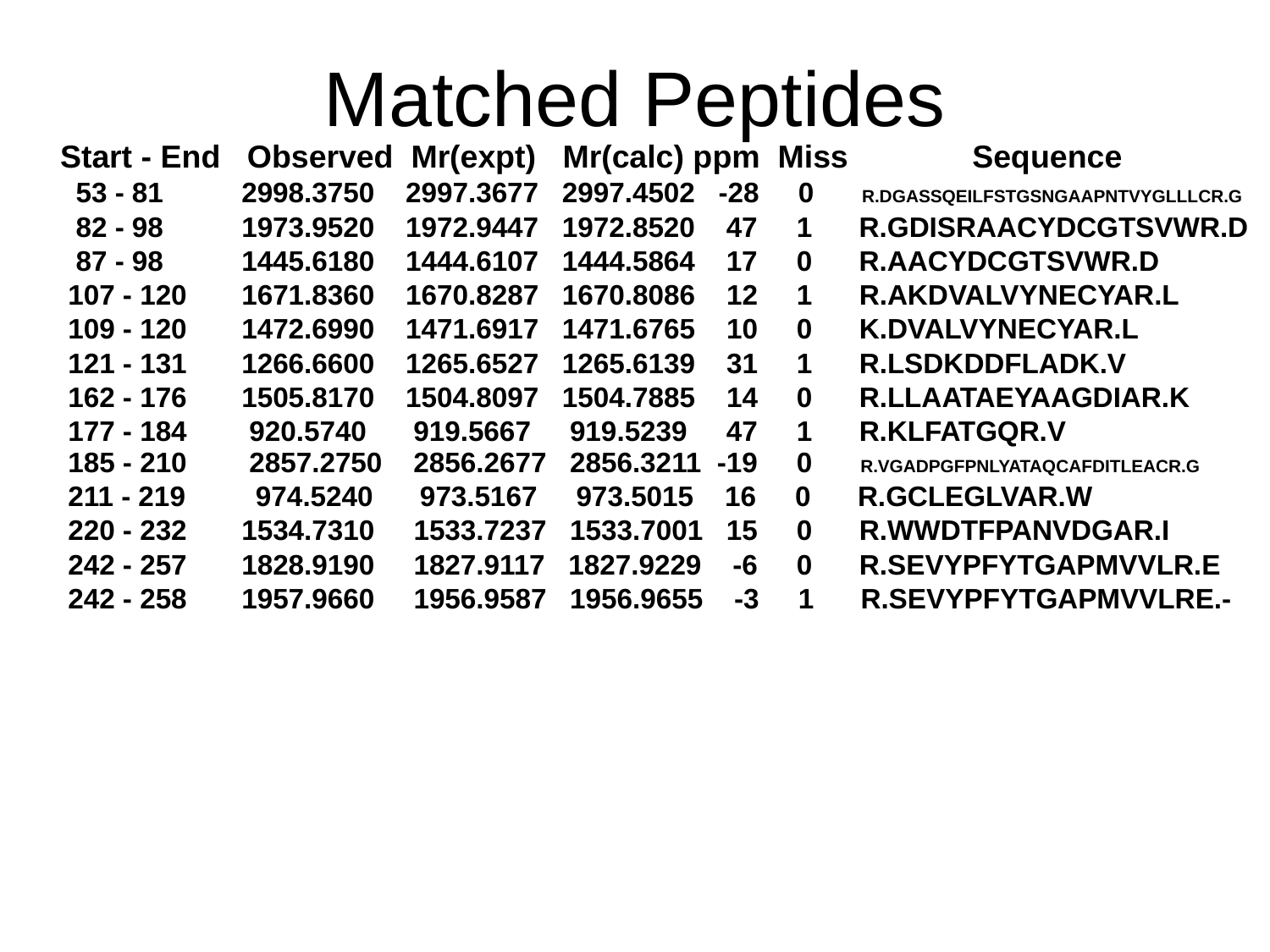

# Matched Peptides
Start - End Observed Mr(expt) Mr(calc) ppm Miss Sequence
 53 - 81 2998.3750 2997.3677 2997.4502 -28 0 R.DGASSQEILFSTGSNGAAPNTVYGLLLCR.G
 82 - 98 1973.9520 1972.9447 1972.8520 47 1 R.GDISRAACYDCGTSVWR.D
 87 - 98 1445.6180 1444.6107 1444.5864 17 0 R.AACYDCGTSVWR.D
 107 - 120 1671.8360 1670.8287 1670.8086 12 1 R.AKDVALVYNECYAR.L
 109 - 120 1472.6990 1471.6917 1471.6765 10 0 K.DVALVYNECYAR.L
 121 - 131 1266.6600 1265.6527 1265.6139 31 1 R.LSDKDDFLADK.V
 162 - 176 1505.8170 1504.8097 1504.7885 14 0 R.LLAATAEYAAGDIAR.K
 177 - 184 920.5740 919.5667 919.5239 47 1 R.KLFATGQR.V
 185 - 210 2857.2750 2856.2677 2856.3211 -19 0 R.VGADPGFPNLYATAQCAFDITLEACR.G
 211 - 219 974.5240 973.5167 973.5015 16 0 R.GCLEGLVAR.W
 220 - 232 1534.7310 1533.7237 1533.7001 15 0 R.WWDTFPANVDGAR.I
 242 - 257 1828.9190 1827.9117 1827.9229 -6 0 R.SEVYPFYTGAPMVVLR.E
 242 - 258 1957.9660 1956.9587 1956.9655 -3 1 R.SEVYPFYTGAPMVVLRE.-

## Slide 77
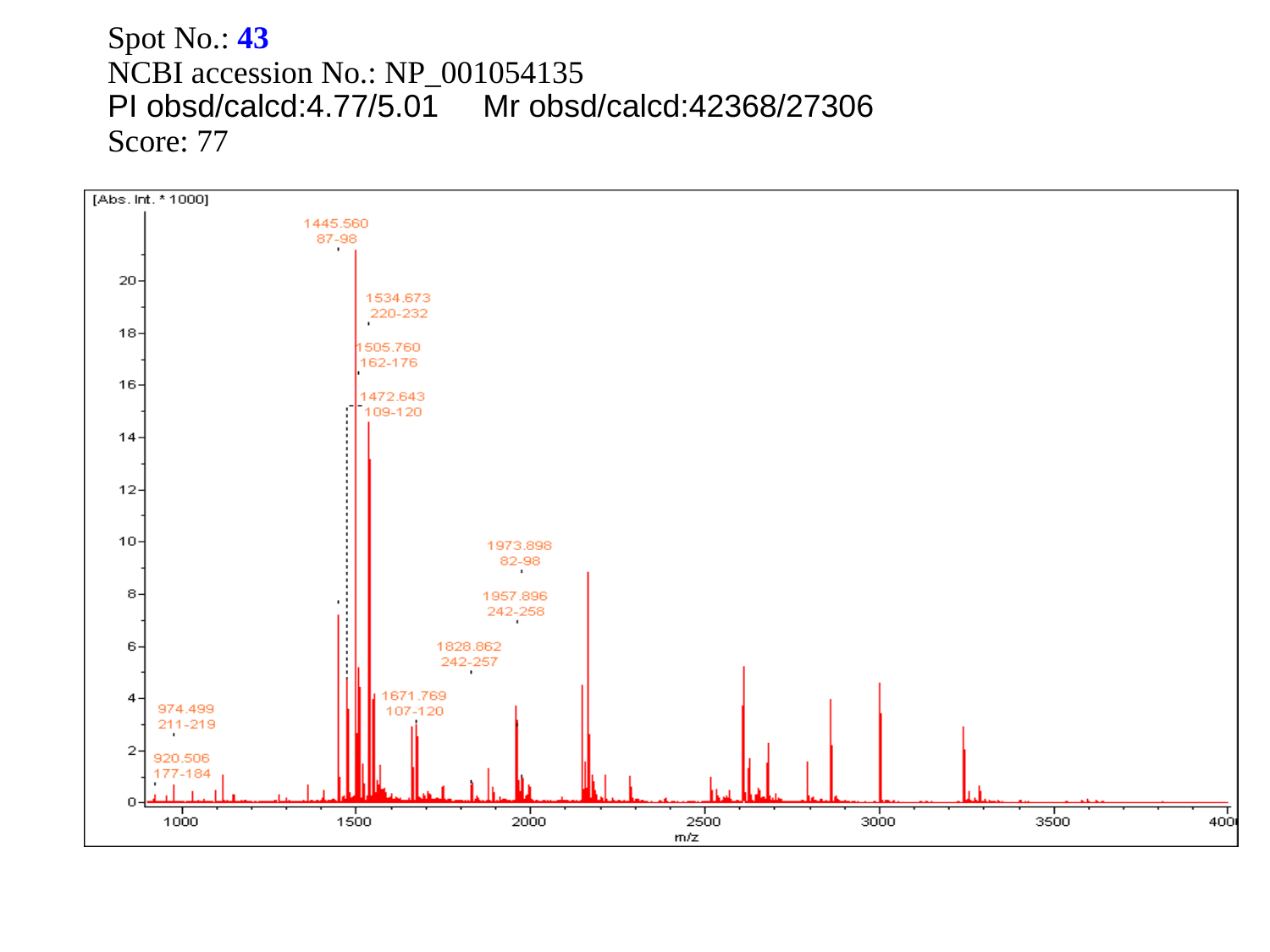

Spot No.: 43
NCBI accession No.: NP_001054135
PI obsd/calcd:4.77/5.01 Mr obsd/calcd:42368/27306
Score: 77

## Slide 78
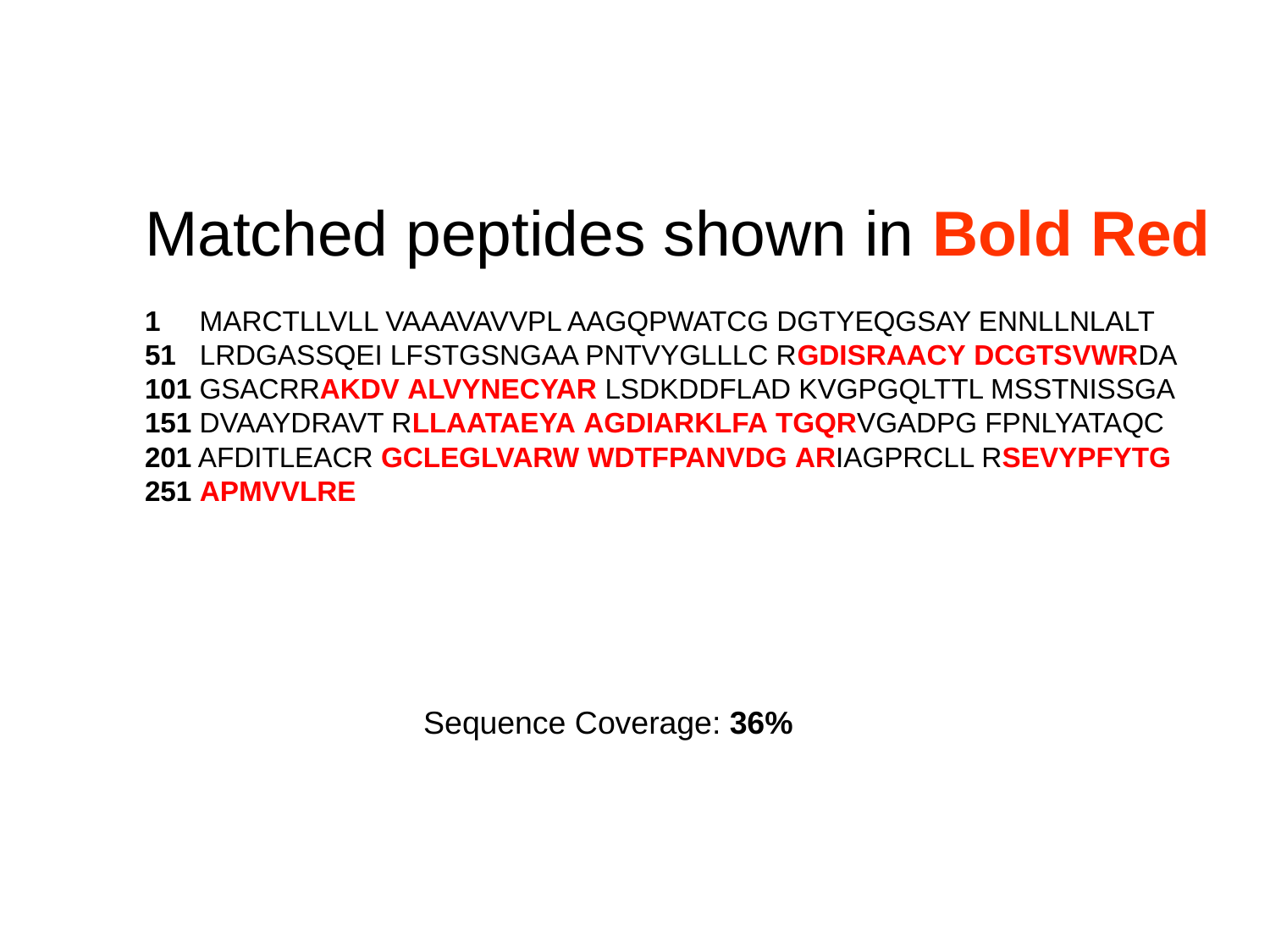

Matched peptides shown in Bold Red
1 MARCTLLVLL VAAAVAVVPL AAGQPWATCG DGTYEQGSAY ENNLLNLALT
51 LRDGASSQEI LFSTGSNGAA PNTVYGLLLC RGDISRAACY DCGTSVWRDA
101 GSACRRAKDV ALVYNECYAR LSDKDDFLAD KVGPGQLTTL MSSTNISSGA
151 DVAAYDRAVT RLLAATAEYA AGDIARKLFA TGQRVGADPG FPNLYATAQC
201 AFDITLEACR GCLEGLVARW WDTFPANVDG ARIAGPRCLL RSEVYPFYTG
251 APMVVLRE
# Sequence Coverage: 36%

## Slide 79
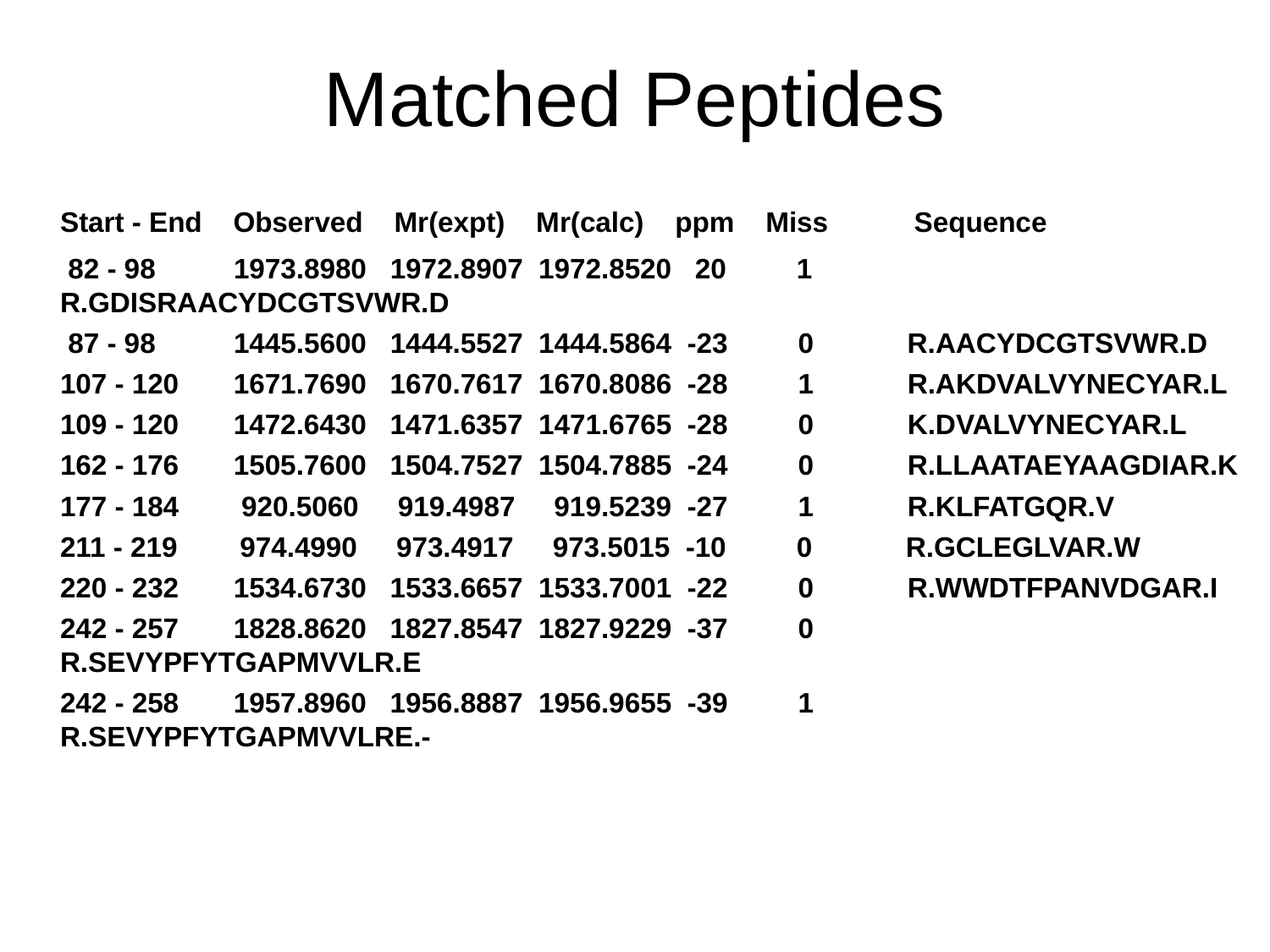

# Matched Peptides
Start - End Observed Mr(expt) Mr(calc) ppm Miss Sequence
 82 - 98 1973.8980 1972.8907 1972.8520 20 1 R.GDISRAACYDCGTSVWR.D
 87 - 98 1445.5600 1444.5527 1444.5864 -23 0 R.AACYDCGTSVWR.D
107 - 120 1671.7690 1670.7617 1670.8086 -28 1 R.AKDVALVYNECYAR.L
109 - 120 1472.6430 1471.6357 1471.6765 -28 0 K.DVALVYNECYAR.L
162 - 176 1505.7600 1504.7527 1504.7885 -24 0 R.LLAATAEYAAGDIAR.K
177 - 184 920.5060 919.4987 919.5239 -27 1 R.KLFATGQR.V
211 - 219 974.4990 973.4917 973.5015 -10 0 R.GCLEGLVAR.W
220 - 232 1534.6730 1533.6657 1533.7001 -22 0 R.WWDTFPANVDGAR.I
242 - 257 1828.8620 1827.8547 1827.9229 -37 0 R.SEVYPFYTGAPMVVLR.E
242 - 258 1957.8960 1956.8887 1956.9655 -39 1 R.SEVYPFYTGAPMVVLRE.-

## Slide 80
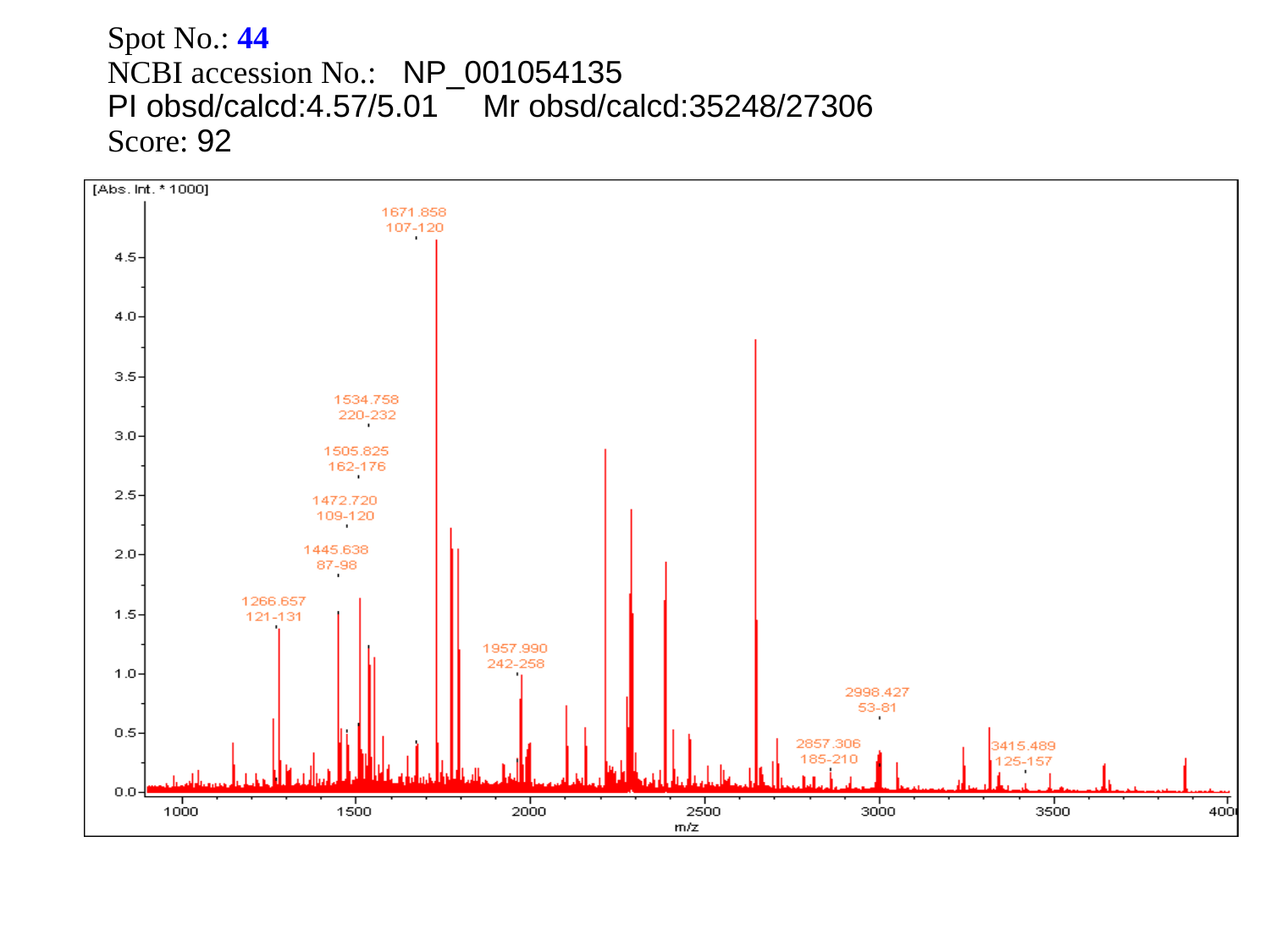

Spot No.: 44
NCBI accession No.: NP_001054135
PI obsd/calcd:4.57/5.01 Mr obsd/calcd:35248/27306
Score: 92

## Slide 81
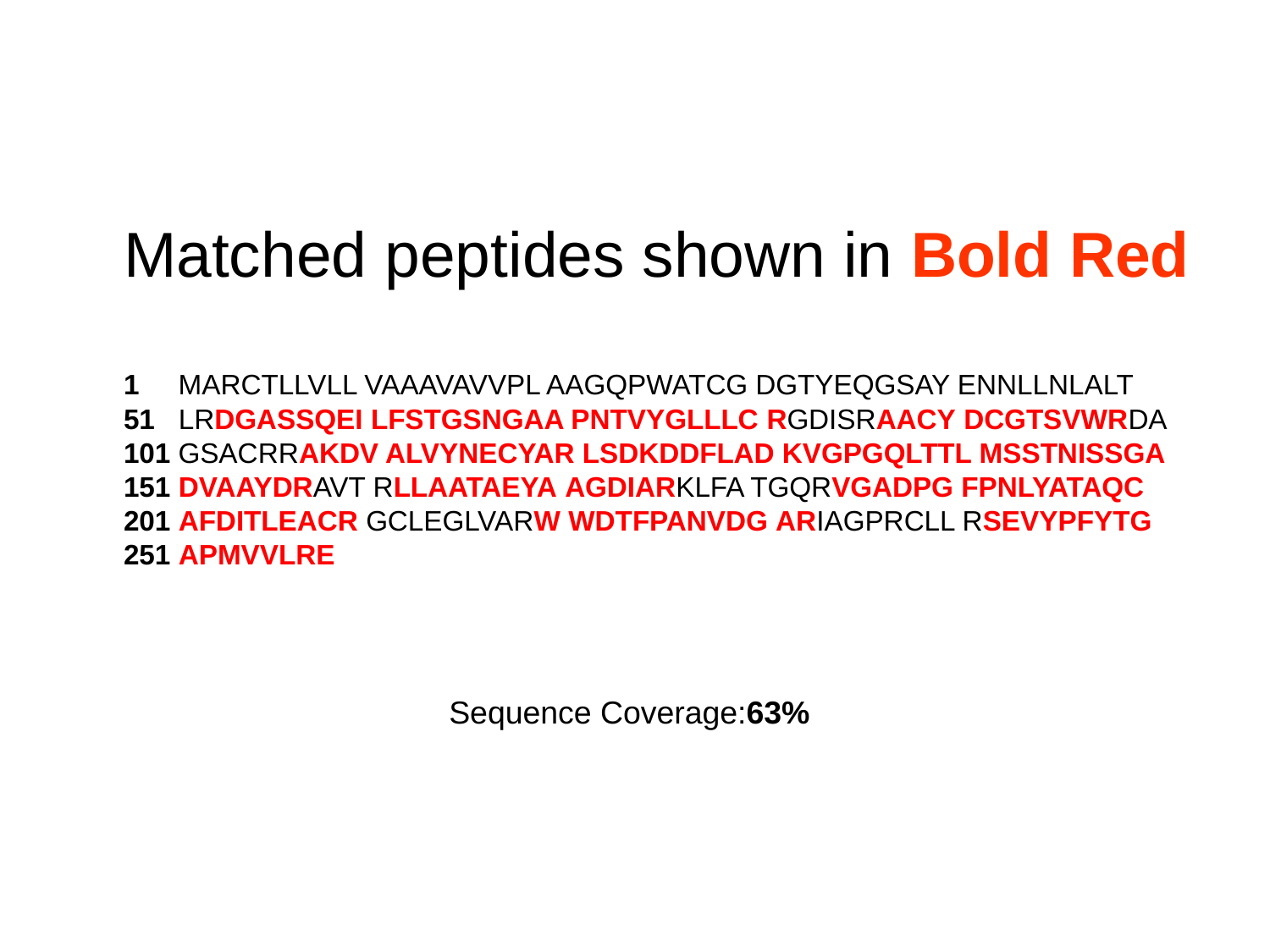

Matched peptides shown in Bold Red
1 MARCTLLVLL VAAAVAVVPL AAGQPWATCG DGTYEQGSAY ENNLLNLALT
51 LRDGASSQEI LFSTGSNGAA PNTVYGLLLC RGDISRAACY DCGTSVWRDA
101 GSACRRAKDV ALVYNECYAR LSDKDDFLAD KVGPGQLTTL MSSTNISSGA
151 DVAAYDRAVT RLLAATAEYA AGDIARKLFA TGQRVGADPG FPNLYATAQC
201 AFDITLEACR GCLEGLVARW WDTFPANVDG ARIAGPRCLL RSEVYPFYTG
251 APMVVLRE
# Sequence Coverage:63%

## Slide 82
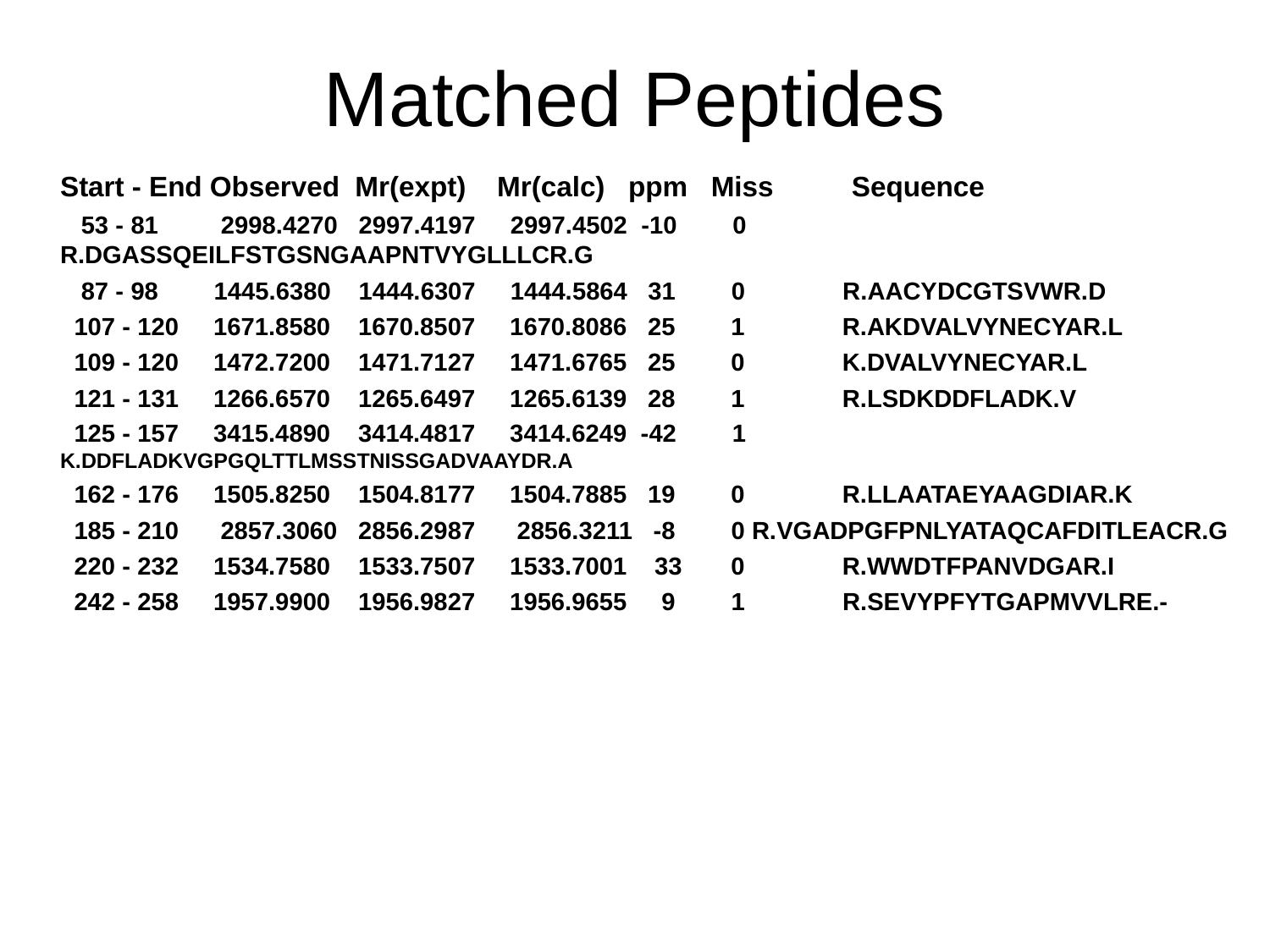

# Matched Peptides
Start - End Observed Mr(expt) Mr(calc) ppm Miss Sequence
 53 - 81 2998.4270 2997.4197 2997.4502 -10 0 R.DGASSQEILFSTGSNGAAPNTVYGLLLCR.G
 87 - 98 1445.6380 1444.6307 1444.5864 31 0 R.AACYDCGTSVWR.D
 107 - 120 1671.8580 1670.8507 1670.8086 25 1 R.AKDVALVYNECYAR.L
 109 - 120 1472.7200 1471.7127 1471.6765 25 0 K.DVALVYNECYAR.L
 121 - 131 1266.6570 1265.6497 1265.6139 28 1 R.LSDKDDFLADK.V
 125 - 157 3415.4890 3414.4817 3414.6249 -42 1 K.DDFLADKVGPGQLTTLMSSTNISSGADVAAYDR.A
 162 - 176 1505.8250 1504.8177 1504.7885 19 0 R.LLAATAEYAAGDIAR.K
 185 - 210 2857.3060 2856.2987 2856.3211 -8 0 R.VGADPGFPNLYATAQCAFDITLEACR.G
 220 - 232 1534.7580 1533.7507 1533.7001 33 0 R.WWDTFPANVDGAR.I
 242 - 258 1957.9900 1956.9827 1956.9655 9 1 R.SEVYPFYTGAPMVVLRE.-

## Slide 83
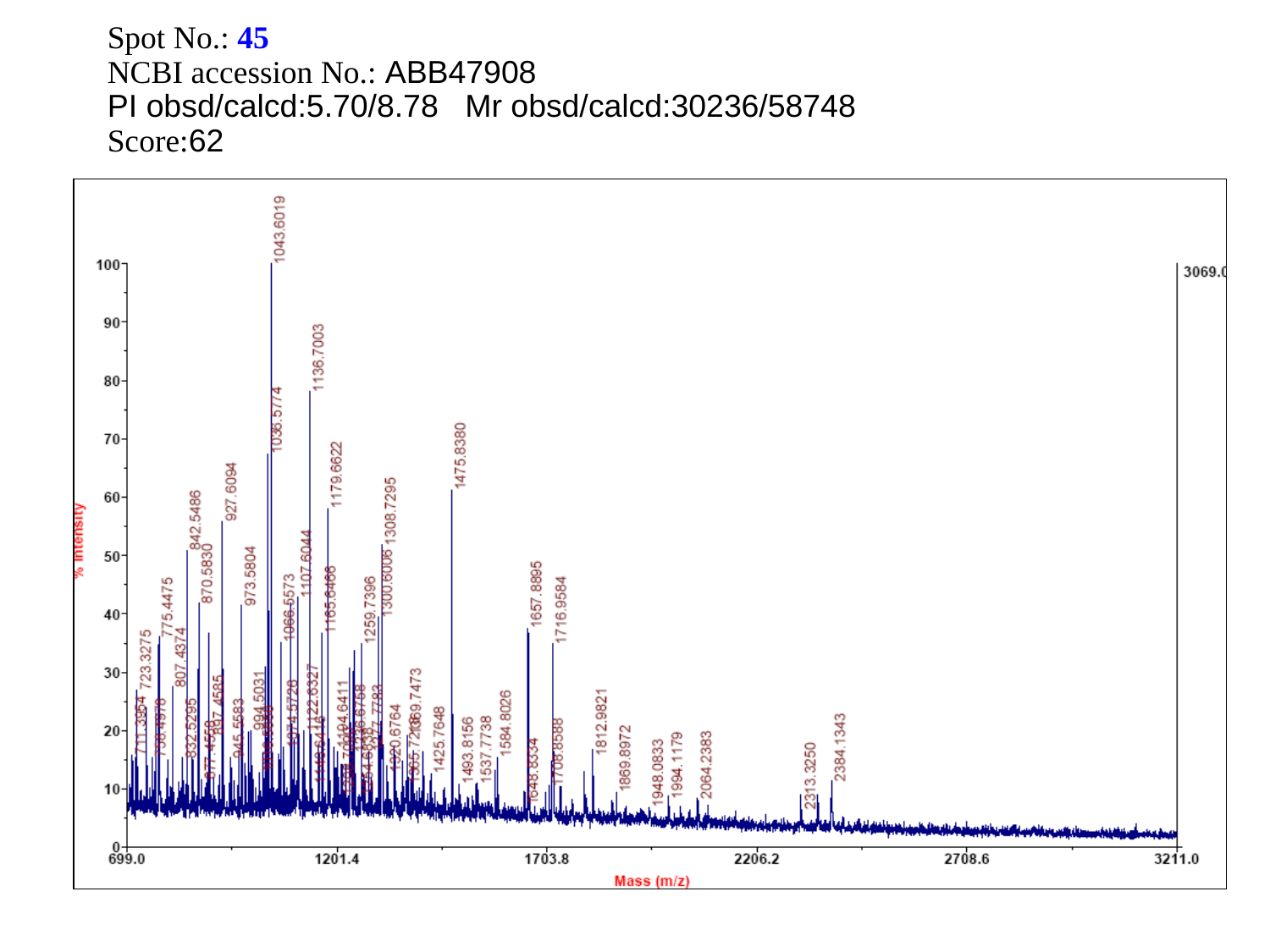

Spot No.: 45
NCBI accession No.: ABB47908
PI obsd/calcd:5.70/8.78 Mr obsd/calcd:30236/58748
Score:62

## Slide 84
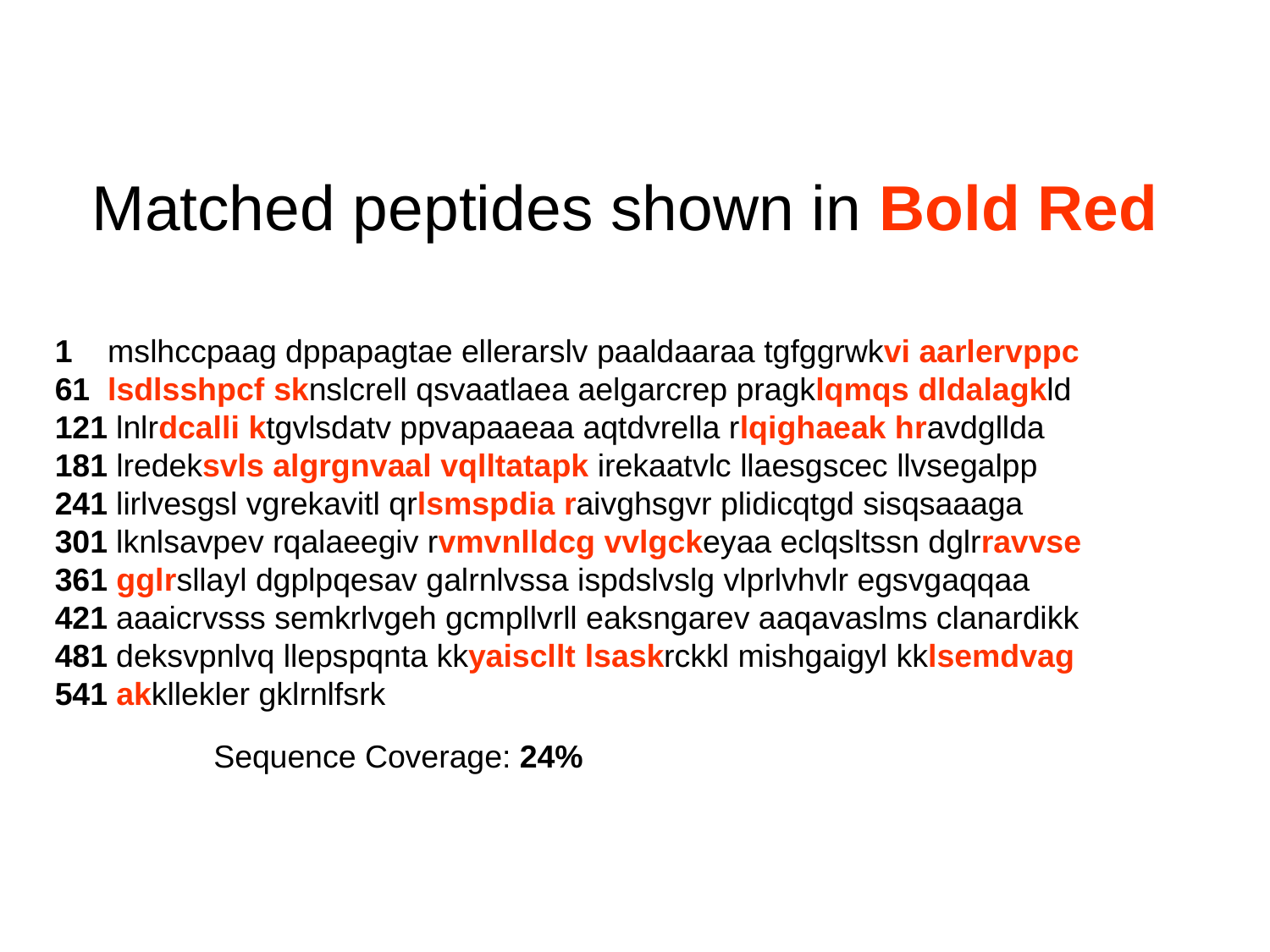

#
Matched peptides shown in Bold Red
1 mslhccpaag dppapagtae ellerarslv paaldaaraa tgfggrwkvi aarlervppc
61 lsdlsshpcf sknslcrell qsvaatlaea aelgarcrep pragklqmqs dldalagkld
121 lnlrdcalli ktgvlsdatv ppvapaaeaa aqtdvrella rlqighaeak hravdgllda
181 lredeksvls algrgnvaal vqlltatapk irekaatvlc llaesgscec llvsegalpp
241 lirlvesgsl vgrekavitl qrlsmspdia raivghsgvr plidicqtgd sisqsaaaga
301 lknlsavpev rqalaeegiv rvmvnlldcg vvlgckeyaa eclqsltssn dglrravvse
361 gglrsllayl dgplpqesav galrnlvssa ispdslvslg vlprlvhvlr egsvgaqqaa
421 aaaicrvsss semkrlvgeh gcmpllvrll eaksngarev aaqavaslms clanardikk
481 deksvpnlvq llepspqnta kkyaiscllt lsaskrckkl mishgaigyl kklsemdvag
541 akkllekler gklrnlfsrk
Sequence Coverage: 24%

## Slide 85
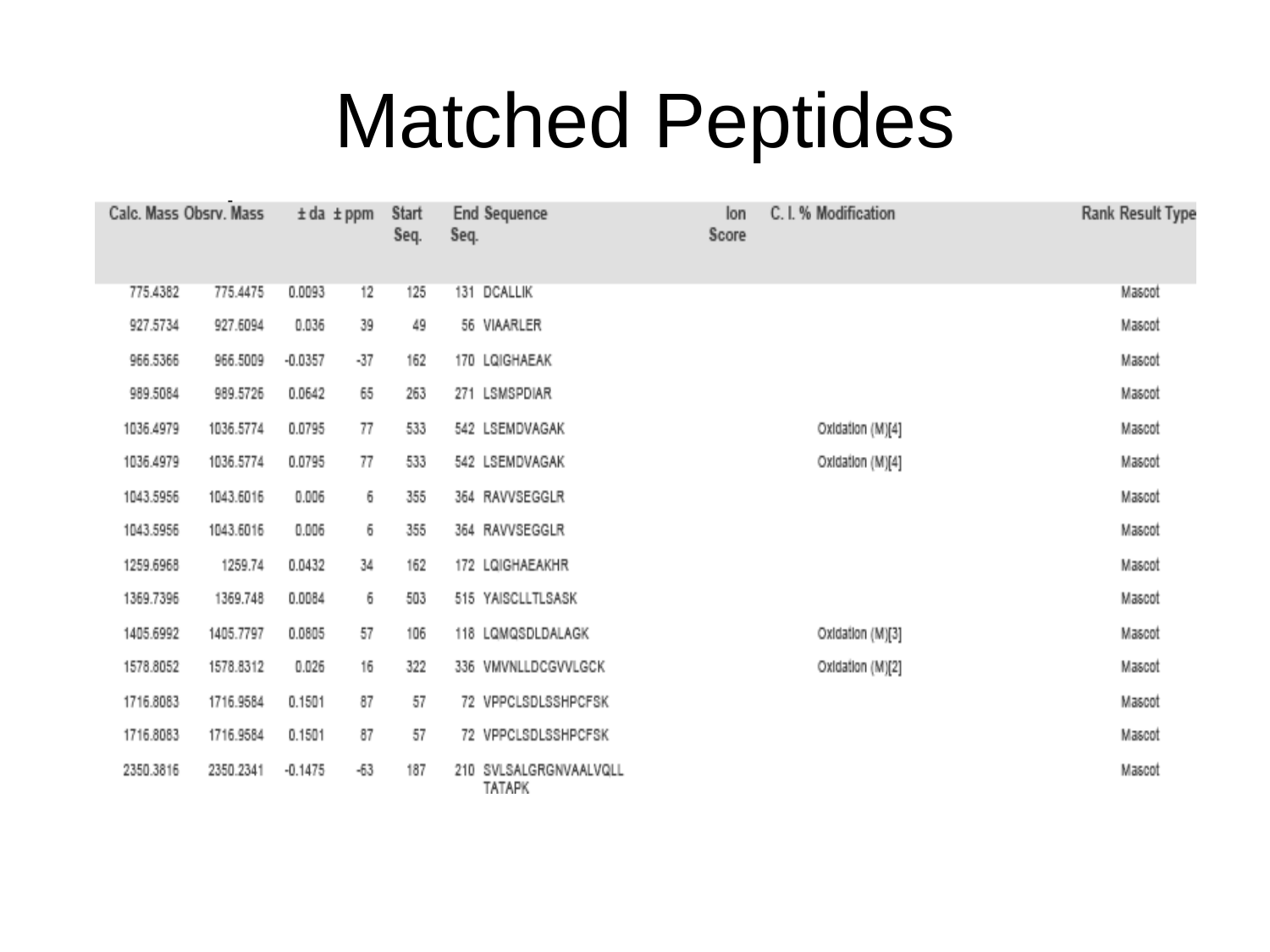

# Matched Peptides

## Slide 86
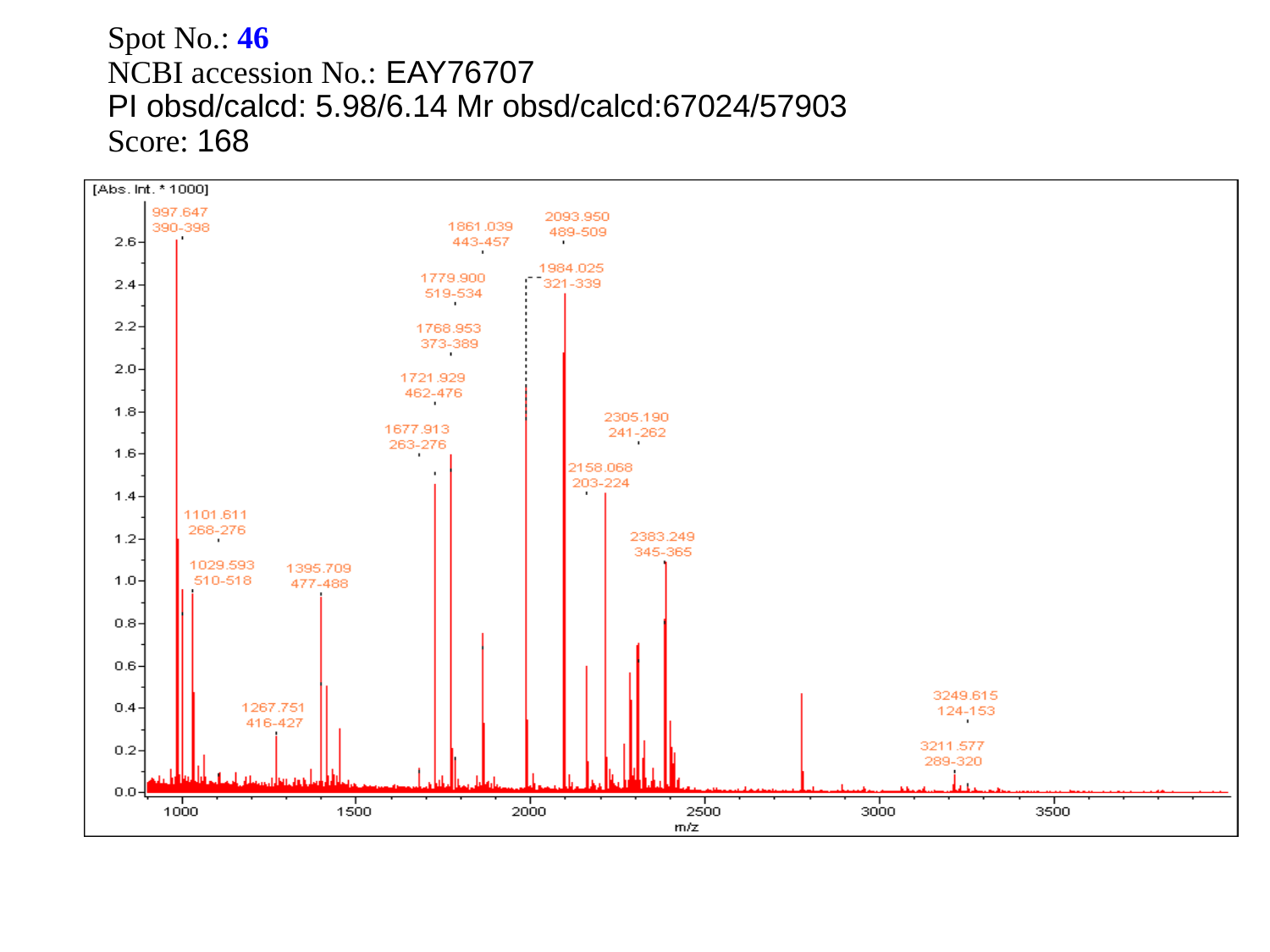

Spot No.: 46
NCBI accession No.: EAY76707
PI obsd/calcd: 5.98/6.14 Mr obsd/calcd:67024/57903
Score: 168

## Slide 87
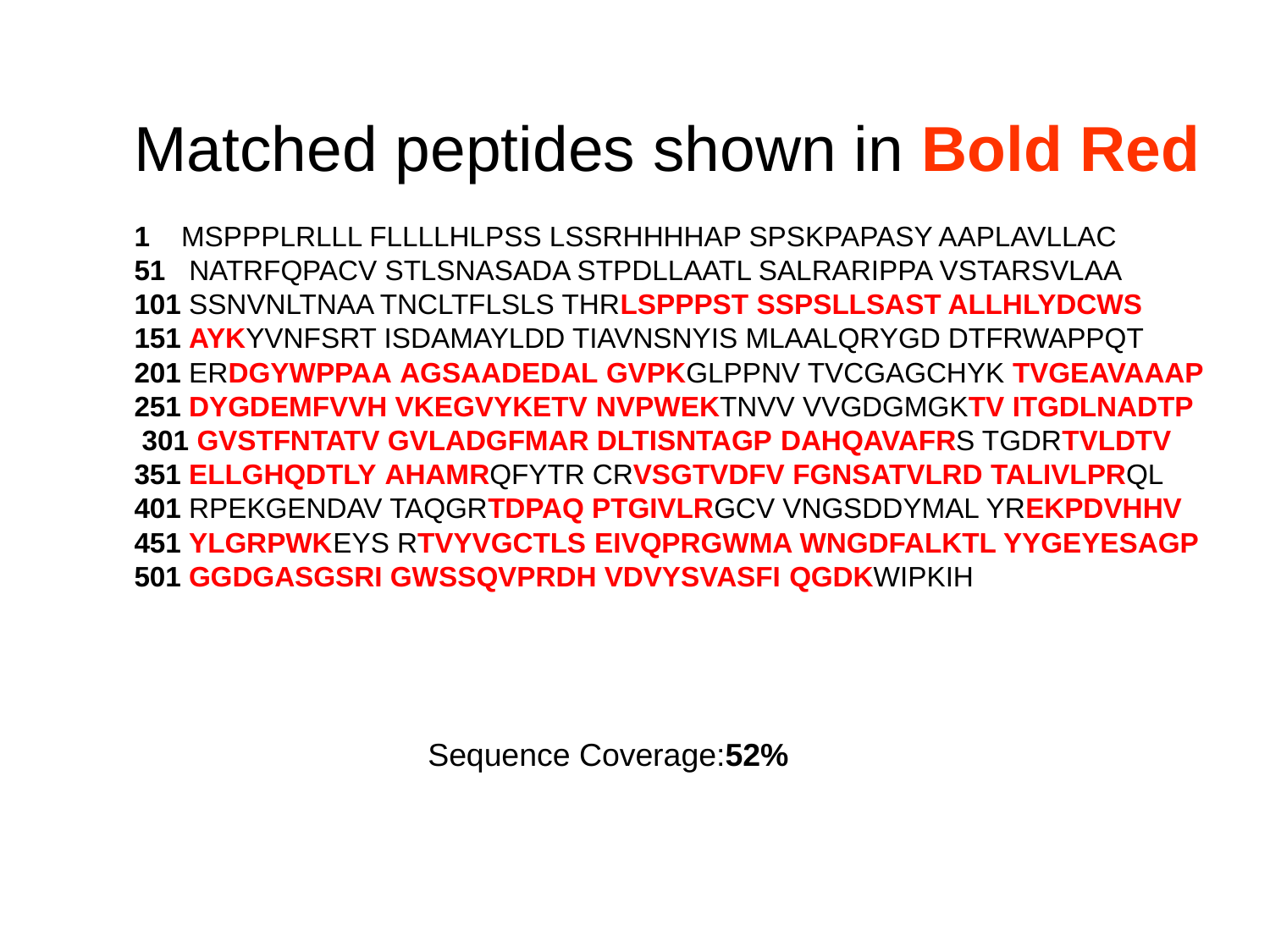

Matched peptides shown in Bold Red
1 MSPPPLRLLL FLLLLHLPSS LSSRHHHHAP SPSKPAPASY AAPLAVLLAC
51 NATRFQPACV STLSNASADA STPDLLAATL SALRARIPPA VSTARSVLAA
101 SSNVNLTNAA TNCLTFLSLS THRLSPPPST SSPSLLSAST ALLHLYDCWS
151 AYKYVNFSRT ISDAMAYLDD TIAVNSNYIS MLAALQRYGD DTFRWAPPQT
201 ERDGYWPPAA AGSAADEDAL GVPKGLPPNV TVCGAGCHYK TVGEAVAAAP
251 DYGDEMFVVH VKEGVYKETV NVPWEKTNVV VVGDGMGKTV ITGDLNADTP
 301 GVSTFNTATV GVLADGFMAR DLTISNTAGP DAHQAVAFRS TGDRTVLDTV
351 ELLGHQDTLY AHAMRQFYTR CRVSGTVDFV FGNSATVLRD TALIVLPRQL
401 RPEKGENDAV TAQGRTDPAQ PTGIVLRGCV VNGSDDYMAL YREKPDVHHV
451 YLGRPWKEYS RTVYVGCTLS EIVQPRGWMA WNGDFALKTL YYGEYESAGP
501 GGDGASGSRI GWSSQVPRDH VDVYSVASFI QGDKWIPKIH
# Sequence Coverage:52%

## Slide 88
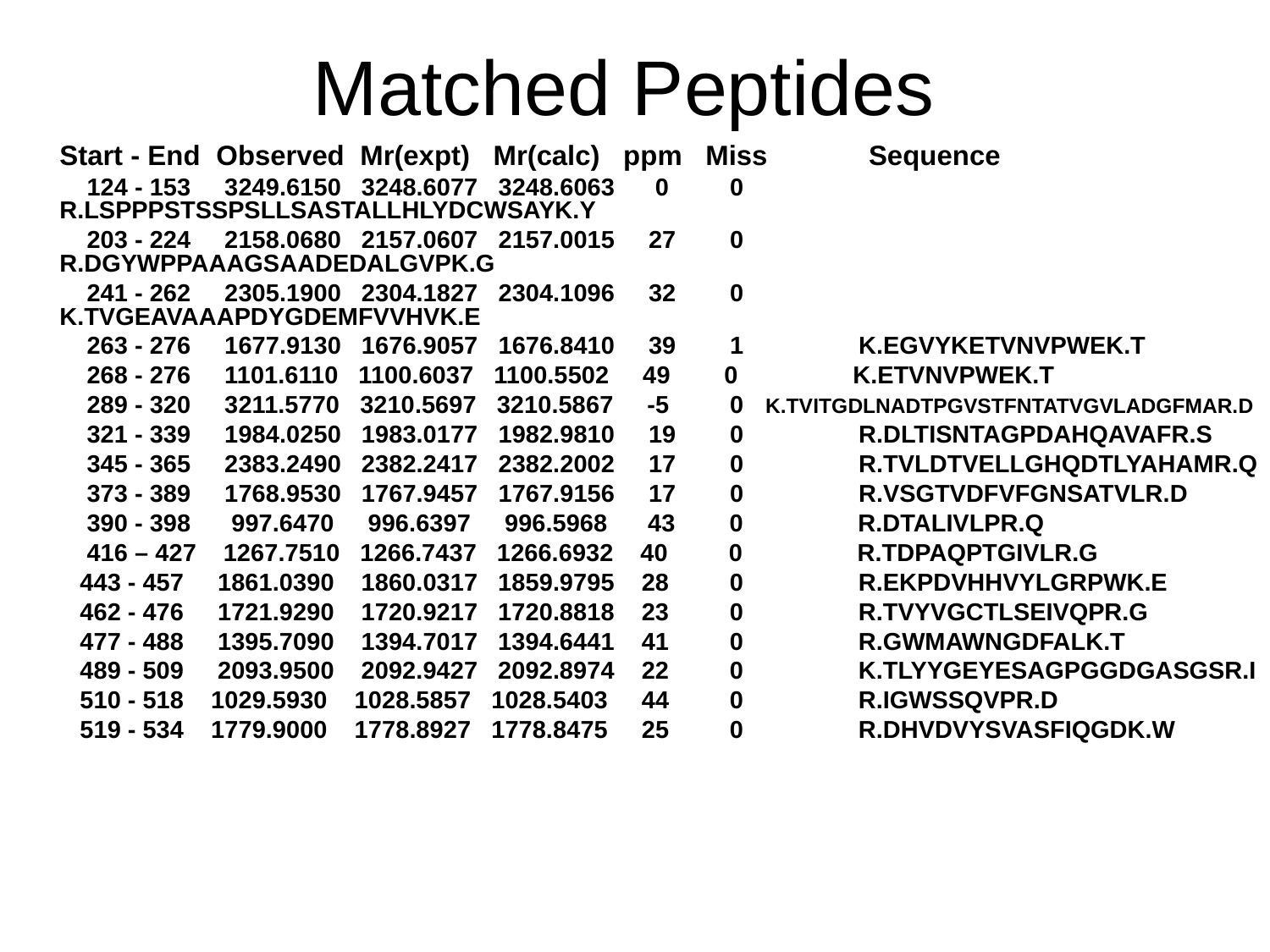

# Matched Peptides
Start - End Observed Mr(expt) Mr(calc) ppm Miss Sequence
 124 - 153 3249.6150 3248.6077 3248.6063 0 0 R.LSPPPSTSSPSLLSASTALLHLYDCWSAYK.Y
 203 - 224 2158.0680 2157.0607 2157.0015 27 0 R.DGYWPPAAAGSAADEDALGVPK.G
 241 - 262 2305.1900 2304.1827 2304.1096 32 0 K.TVGEAVAAAPDYGDEMFVVHVK.E
 263 - 276 1677.9130 1676.9057 1676.8410 39 1 K.EGVYKETVNVPWEK.T
 268 - 276 1101.6110 1100.6037 1100.5502 49 0 K.ETVNVPWEK.T
 289 - 320 3211.5770 3210.5697 3210.5867 -5 0 K.TVITGDLNADTPGVSTFNTATVGVLADGFMAR.D
 321 - 339 1984.0250 1983.0177 1982.9810 19 0 R.DLTISNTAGPDAHQAVAFR.S
 345 - 365 2383.2490 2382.2417 2382.2002 17 0 R.TVLDTVELLGHQDTLYAHAMR.Q
 373 - 389 1768.9530 1767.9457 1767.9156 17 0 R.VSGTVDFVFGNSATVLR.D
 390 - 398 997.6470 996.6397 996.5968 43 0 R.DTALIVLPR.Q
 416 – 427 1267.7510 1266.7437 1266.6932 40 0 R.TDPAQPTGIVLR.G
 443 - 457 1861.0390 1860.0317 1859.9795 28 0 R.EKPDVHHVYLGRPWK.E
 462 - 476 1721.9290 1720.9217 1720.8818 23 0 R.TVYVGCTLSEIVQPR.G
 477 - 488 1395.7090 1394.7017 1394.6441 41 0 R.GWMAWNGDFALK.T
 489 - 509 2093.9500 2092.9427 2092.8974 22 0 K.TLYYGEYESAGPGGDGASGSR.I
 510 - 518 1029.5930 1028.5857 1028.5403 44 0 R.IGWSSQVPR.D
 519 - 534 1779.9000 1778.8927 1778.8475 25 0 R.DHVDVYSVASFIQGDK.W

## Slide 89
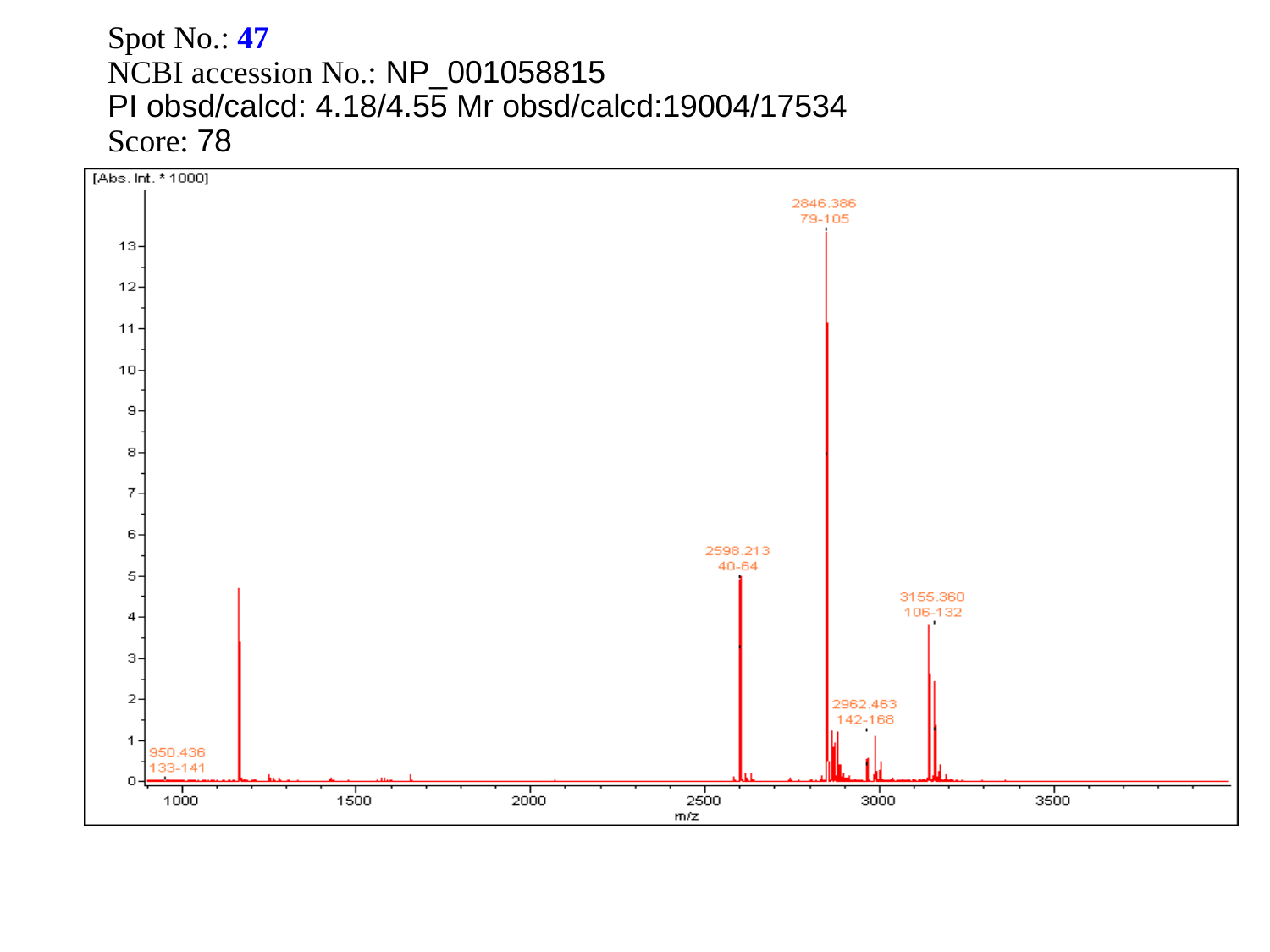

Spot No.: 47
NCBI accession No.: NP_001058815
PI obsd/calcd: 4.18/4.55 Mr obsd/calcd:19004/17534
Score: 78

## Slide 90
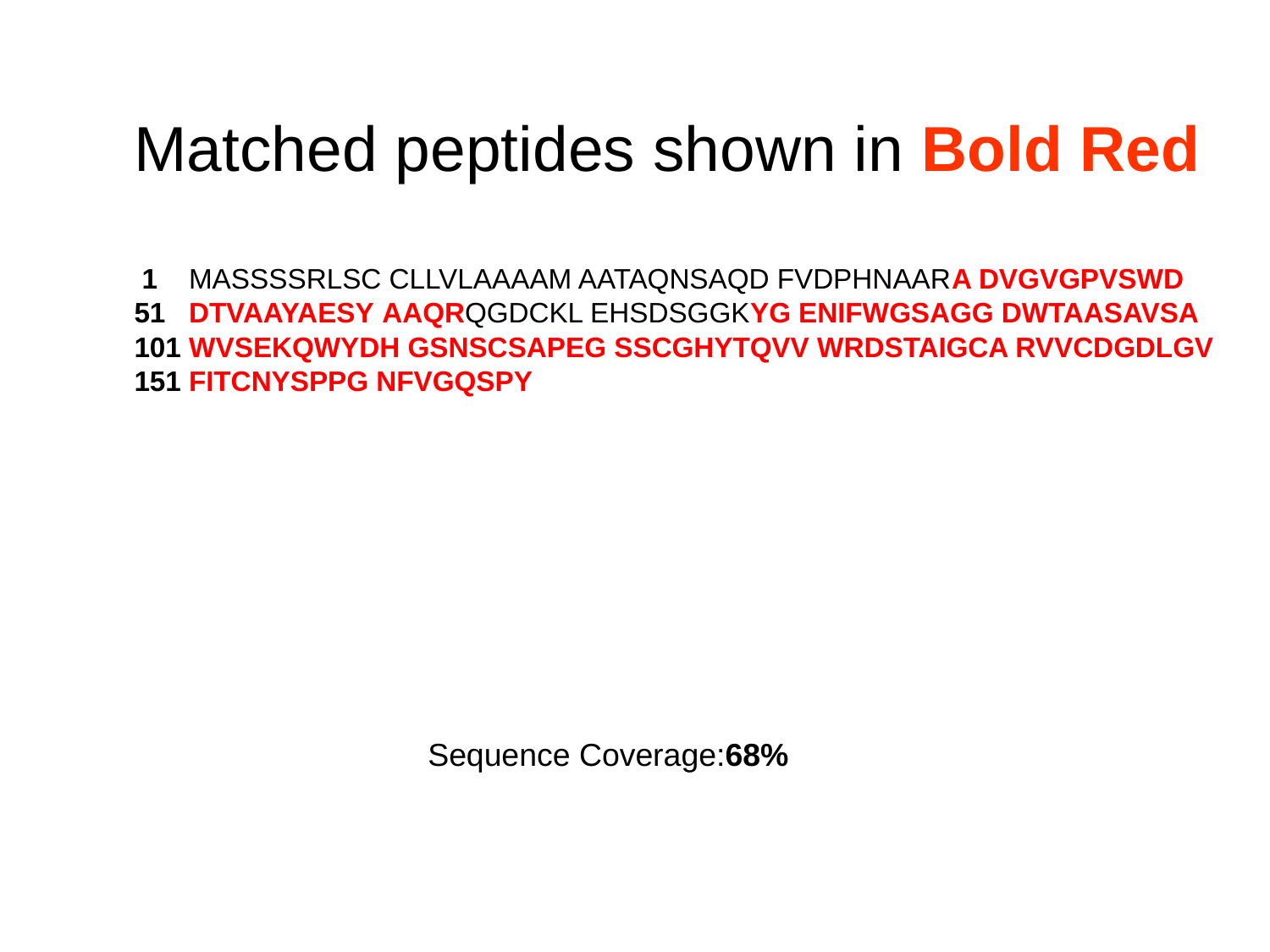

Matched peptides shown in Bold Red
 1 MASSSSRLSC CLLVLAAAAM AATAQNSAQD FVDPHNAARA DVGVGPVSWD
51 DTVAAYAESY AAQRQGDCKL EHSDSGGKYG ENIFWGSAGG DWTAASAVSA
101 WVSEKQWYDH GSNSCSAPEG SSCGHYTQVV WRDSTAIGCA RVVCDGDLGV
151 FITCNYSPPG NFVGQSPY
# Sequence Coverage:68%

## Slide 91
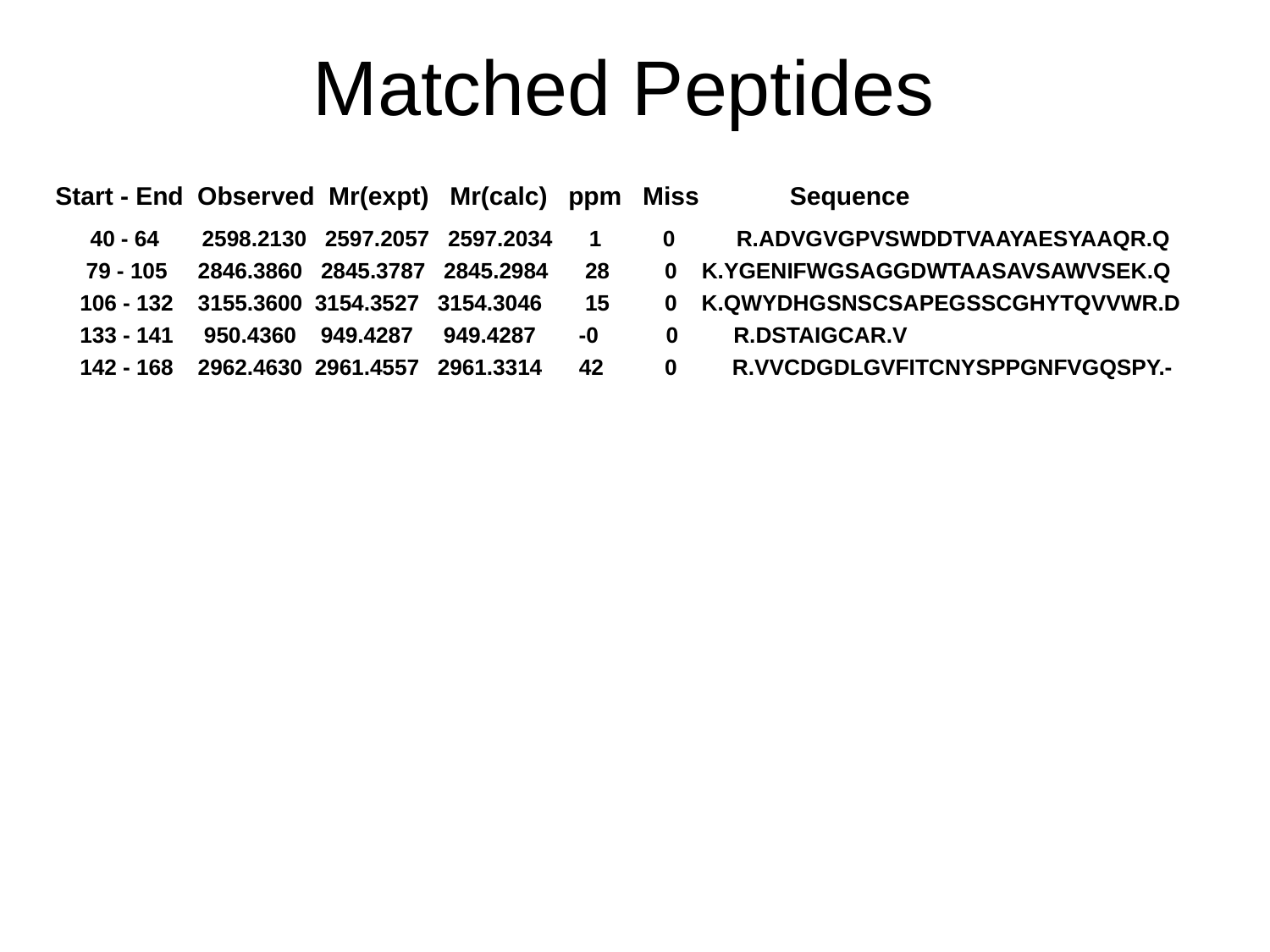

# Matched Peptides
Start - End Observed Mr(expt) Mr(calc) ppm Miss Sequence
 40 - 64 2598.2130 2597.2057 2597.2034 1 0 R.ADVGVGPVSWDDTVAAYAESYAAQR.Q
 79 - 105 2846.3860 2845.3787 2845.2984 28 0 K.YGENIFWGSAGGDWTAASAVSAWVSEK.Q
 106 - 132 3155.3600 3154.3527 3154.3046 15 0 K.QWYDHGSNSCSAPEGSSCGHYTQVVWR.D
 133 - 141 950.4360 949.4287 949.4287 -0 0 R.DSTAIGCAR.V
 142 - 168 2962.4630 2961.4557 2961.3314 42 0 R.VVCDGDLGVFITCNYSPPGNFVGQSPY.-

## Slide 92
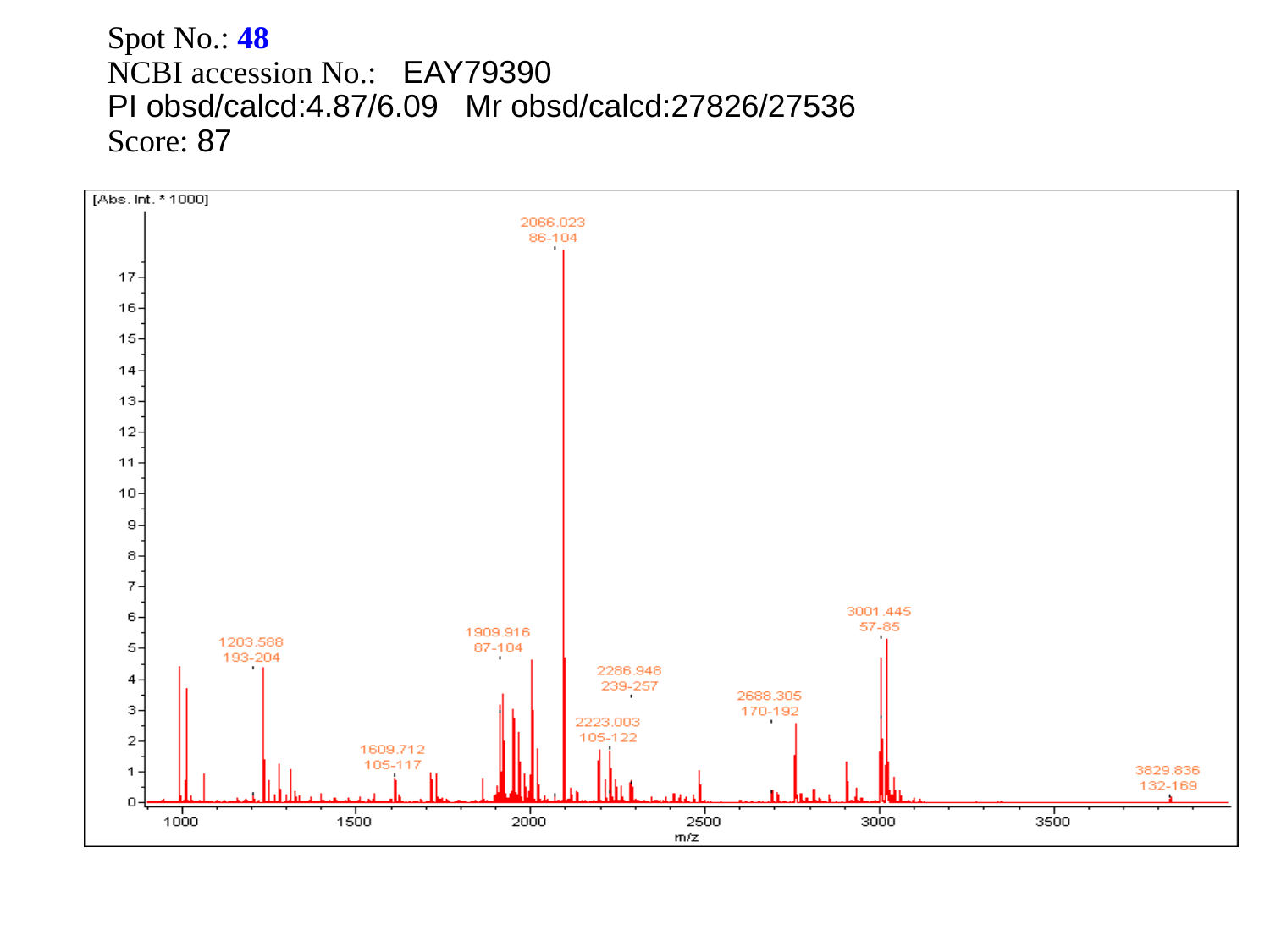

Spot No.: 48
NCBI accession No.: EAY79390
PI obsd/calcd:4.87/6.09 Mr obsd/calcd:27826/27536
Score: 87

## Slide 93
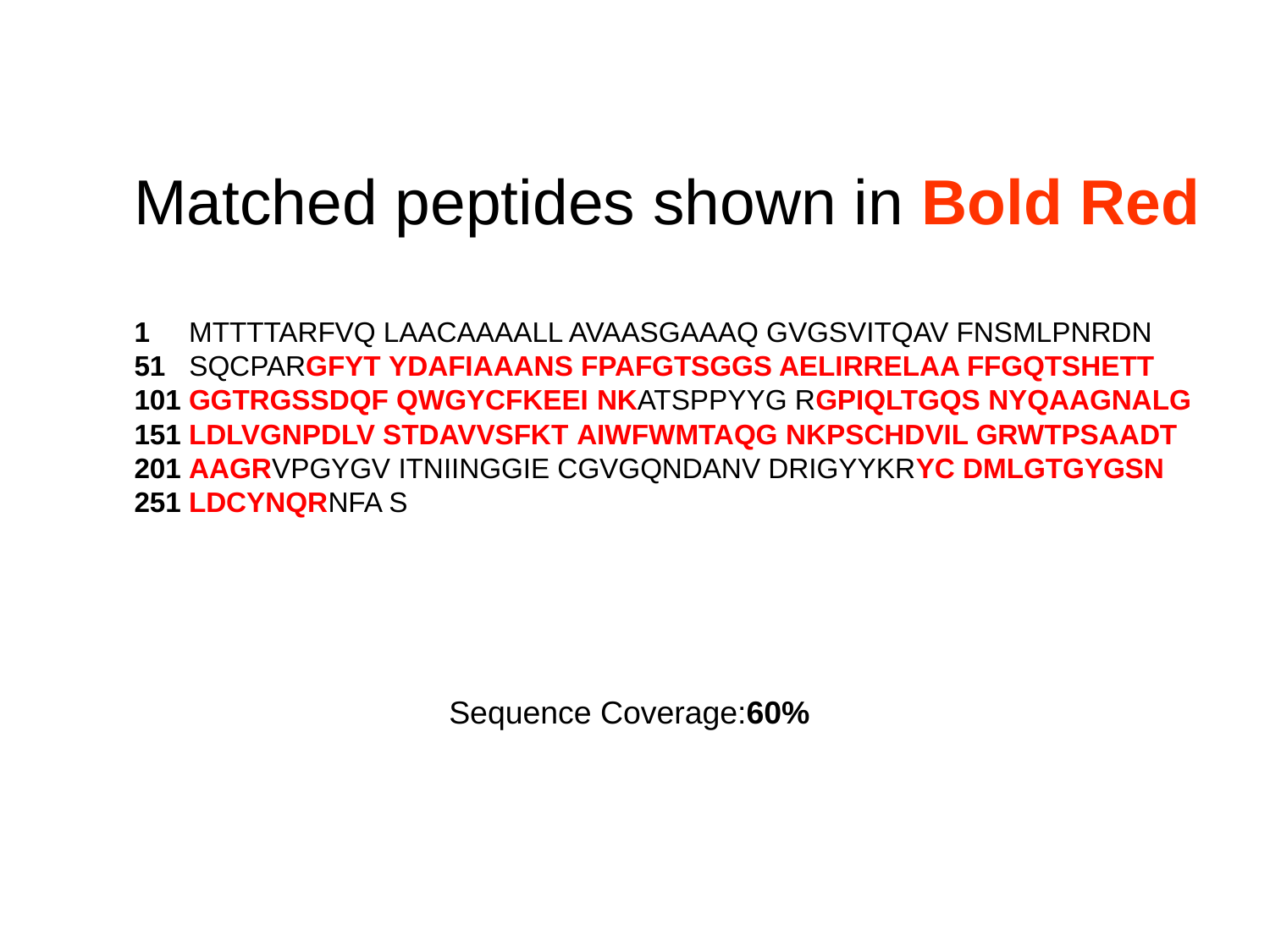

Matched peptides shown in Bold Red
1 MTTTTARFVQ LAACAAAALL AVAASGAAAQ GVGSVITQAV FNSMLPNRDN
51 SQCPARGFYT YDAFIAAANS FPAFGTSGGS AELIRRELAA FFGQTSHETT
101 GGTRGSSDQF QWGYCFKEEI NKATSPPYYG RGPIQLTGQS NYQAAGNALG
151 LDLVGNPDLV STDAVVSFKT AIWFWMTAQG NKPSCHDVIL GRWTPSAADT
201 AAGRVPGYGV ITNIINGGIE CGVGQNDANV DRIGYYKRYC DMLGTGYGSN
251 LDCYNQRNFA S
# Sequence Coverage:60%

## Slide 94
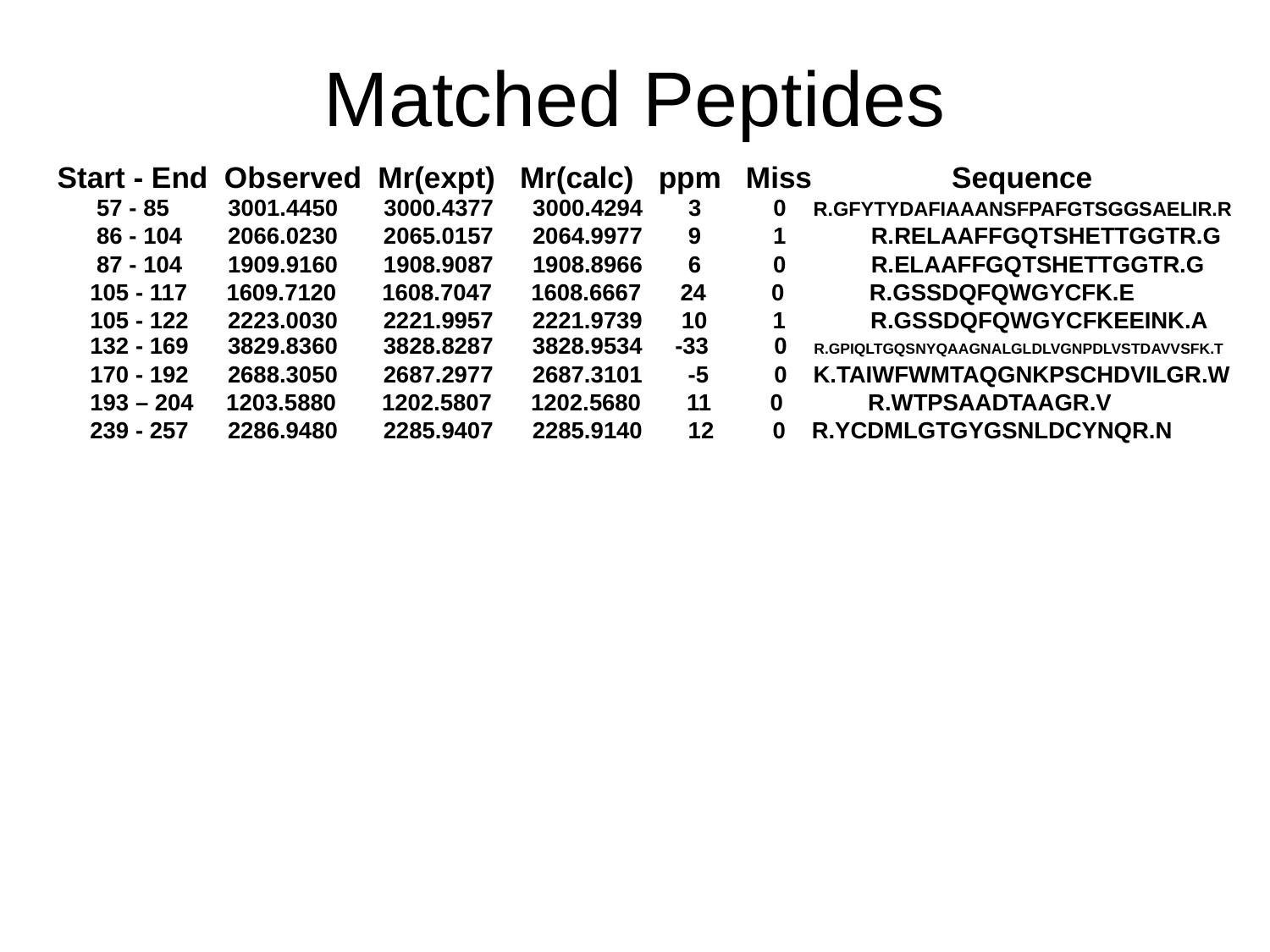

# Matched Peptides
Start - End Observed Mr(expt) Mr(calc) ppm Miss Sequence
 57 - 85 3001.4450 3000.4377 3000.4294 3 0 R.GFYTYDAFIAAANSFPAFGTSGGSAELIR.R
 86 - 104 2066.0230 2065.0157 2064.9977 9 1 R.RELAAFFGQTSHETTGGTR.G
 87 - 104 1909.9160 1908.9087 1908.8966 6 0 R.ELAAFFGQTSHETTGGTR.G
 105 - 117 1609.7120 1608.7047 1608.6667 24 0 R.GSSDQFQWGYCFK.E
 105 - 122 2223.0030 2221.9957 2221.9739 10 1 R.GSSDQFQWGYCFKEEINK.A
 132 - 169 3829.8360 3828.8287 3828.9534 -33 0 R.GPIQLTGQSNYQAAGNALGLDLVGNPDLVSTDAVVSFK.T
 170 - 192 2688.3050 2687.2977 2687.3101 -5 0 K.TAIWFWMTAQGNKPSCHDVILGR.W
 193 – 204 1203.5880 1202.5807 1202.5680 11 0 R.WTPSAADTAAGR.V
 239 - 257 2286.9480 2285.9407 2285.9140 12 0 R.YCDMLGTGYGSNLDCYNQR.N

## Slide 95
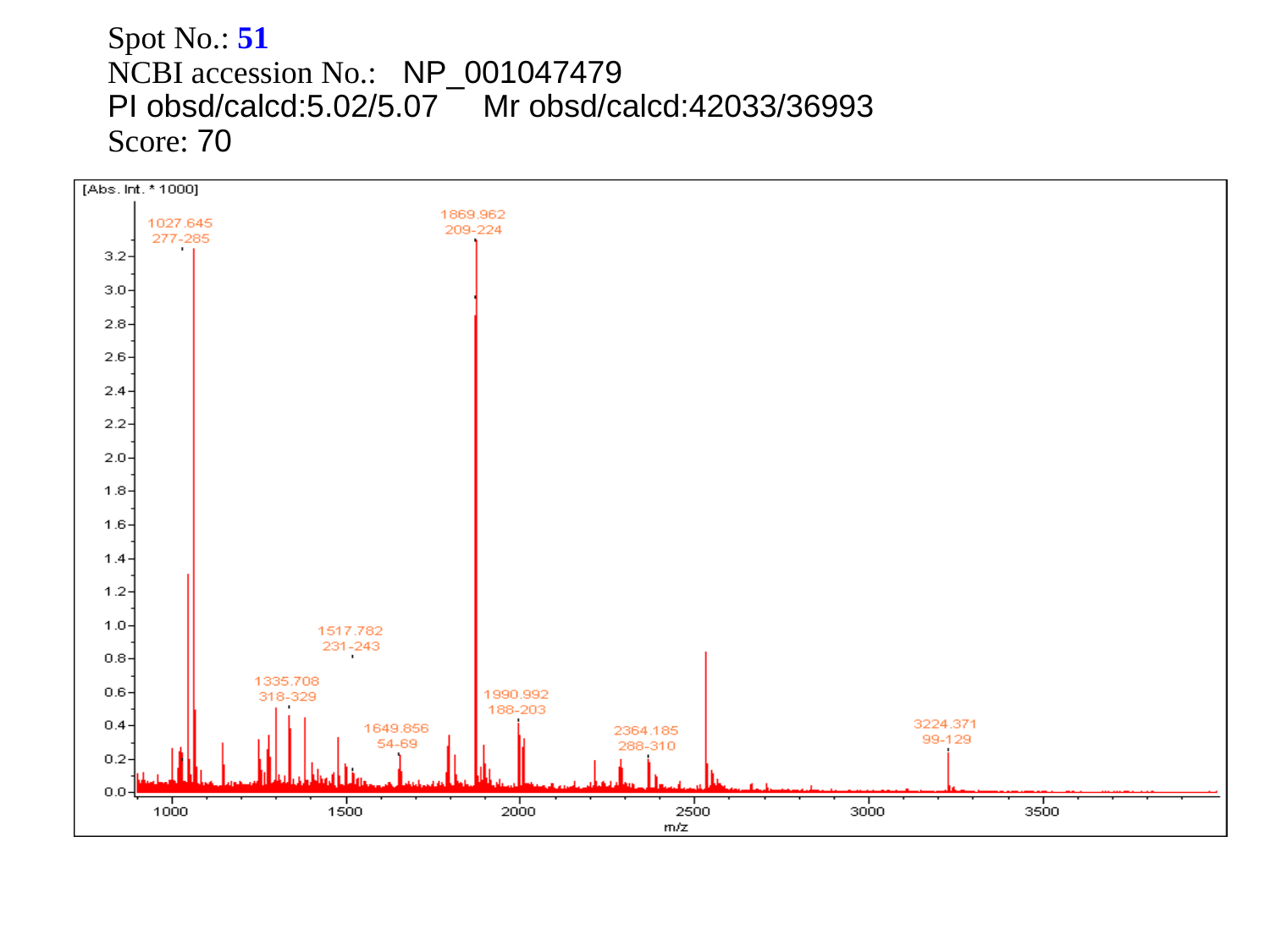

Spot No.: 51
NCBI accession No.: NP_001047479
PI obsd/calcd:5.02/5.07 Mr obsd/calcd:42033/36993
Score: 70

## Slide 96
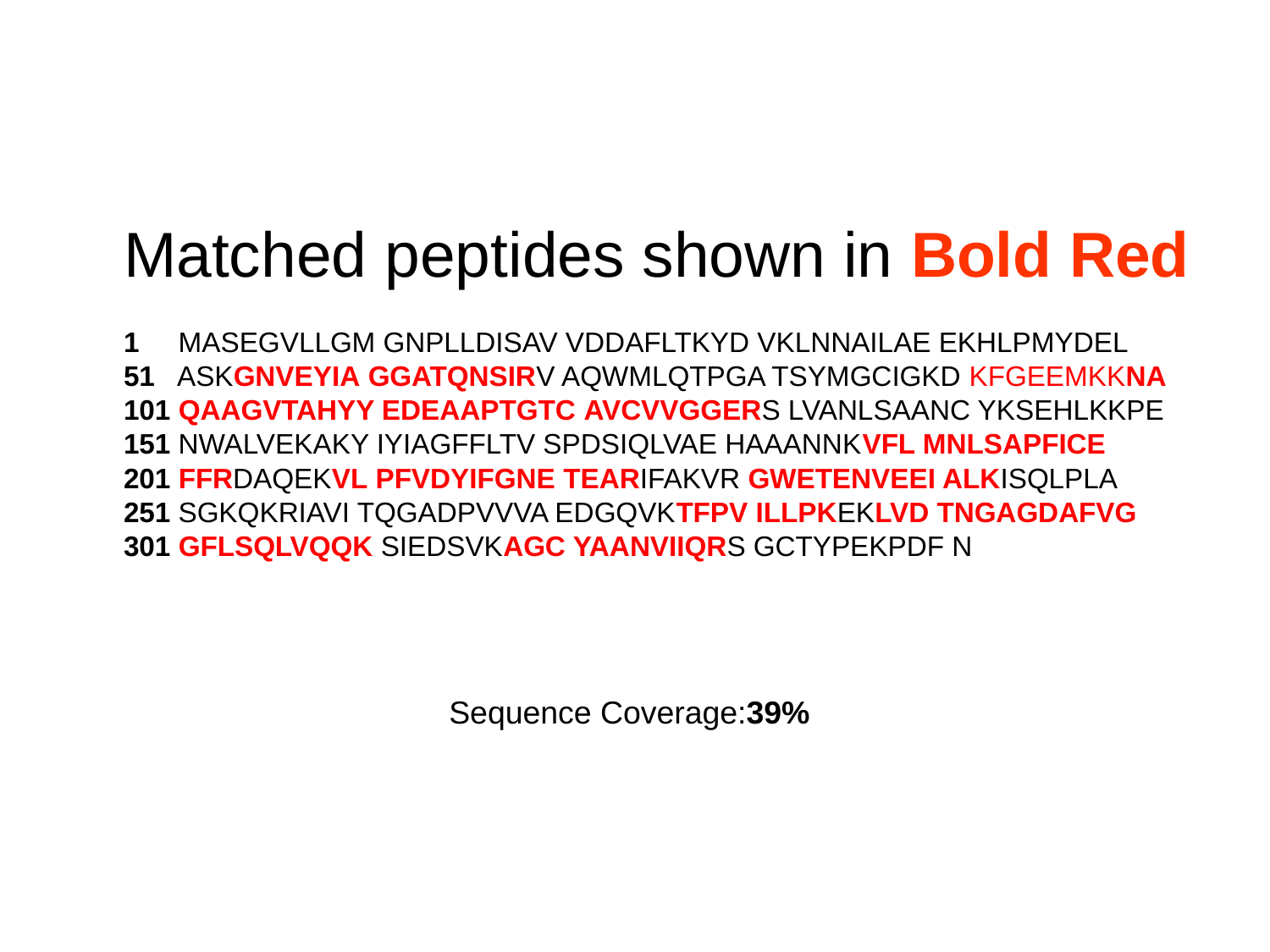

Matched peptides shown in Bold Red
1 MASEGVLLGM GNPLLDISAV VDDAFLTKYD VKLNNAILAE EKHLPMYDEL
51 ASKGNVEYIA GGATQNSIRV AQWMLQTPGA TSYMGCIGKD KFGEEMKKNA
101 QAAGVTAHYY EDEAAPTGTC AVCVVGGERS LVANLSAANC YKSEHLKKPE
151 NWALVEKAKY IYIAGFFLTV SPDSIQLVAE HAAANNKVFL MNLSAPFICE
201 FFRDAQEKVL PFVDYIFGNE TEARIFAKVR GWETENVEEI ALKISQLPLA
251 SGKQKRIAVI TQGADPVVVA EDGQVKTFPV ILLPKEKLVD TNGAGDAFVG
301 GFLSQLVQQK SIEDSVKAGC YAANVIIQRS GCTYPEKPDF N
# Sequence Coverage:39%

## Slide 97
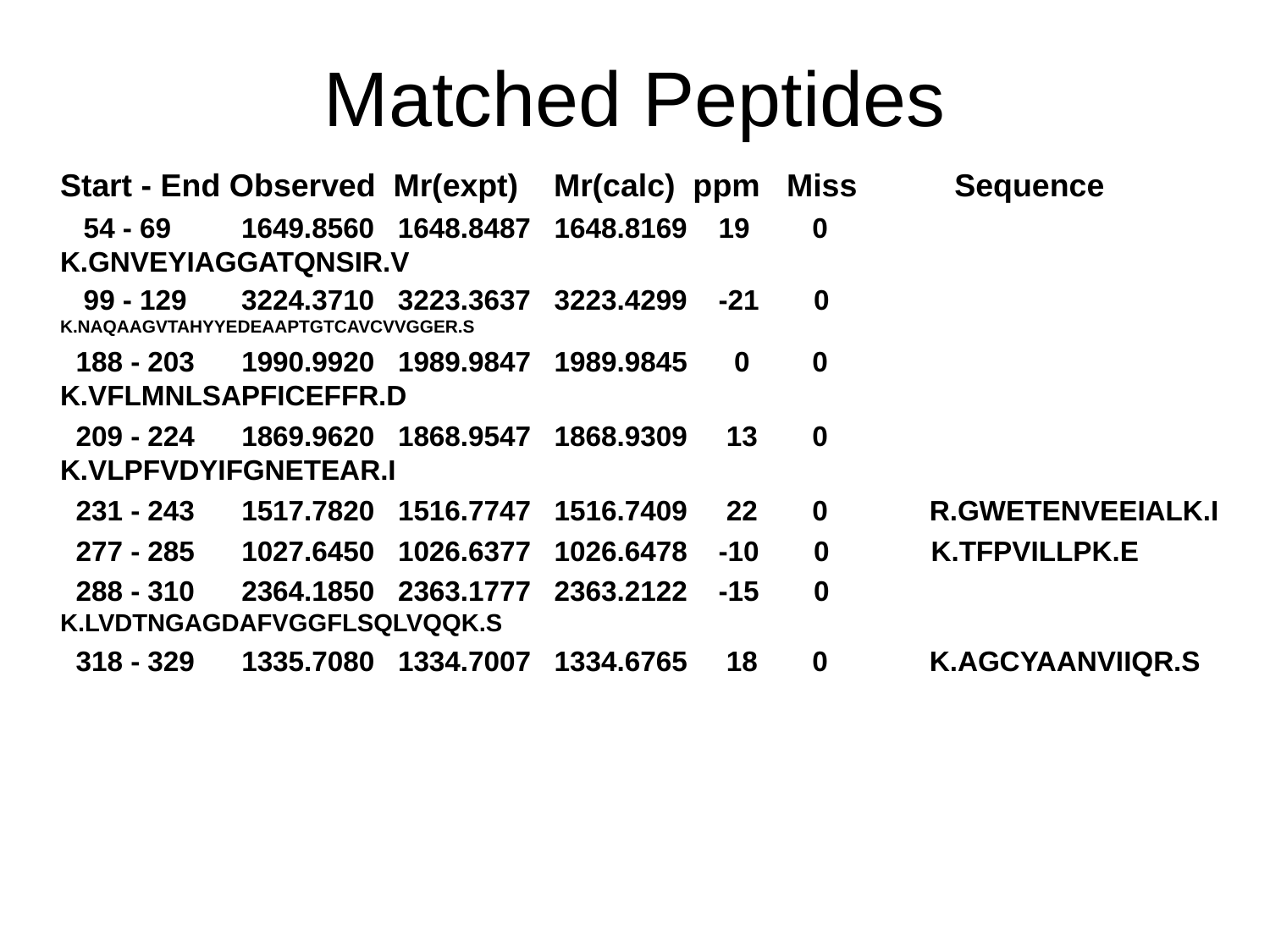

# Matched Peptides
Start - End Observed Mr(expt) Mr(calc) ppm Miss Sequence
 54 - 69 1649.8560 1648.8487 1648.8169 19 0 K.GNVEYIAGGATQNSIR.V
 99 - 129 3224.3710 3223.3637 3223.4299 -21 0 K.NAQAAGVTAHYYEDEAAPTGTCAVCVVGGER.S
 188 - 203 1990.9920 1989.9847 1989.9845 0 0 K.VFLMNLSAPFICEFFR.D
 209 - 224 1869.9620 1868.9547 1868.9309 13 0 K.VLPFVDYIFGNETEAR.I
 231 - 243 1517.7820 1516.7747 1516.7409 22 0 R.GWETENVEEIALK.I
 277 - 285 1027.6450 1026.6377 1026.6478 -10 0 K.TFPVILLPK.E
 288 - 310 2364.1850 2363.1777 2363.2122 -15 0 K.LVDTNGAGDAFVGGFLSQLVQQK.S
 318 - 329 1335.7080 1334.7007 1334.6765 18 0 K.AGCYAANVIIQR.S

## Slide 98
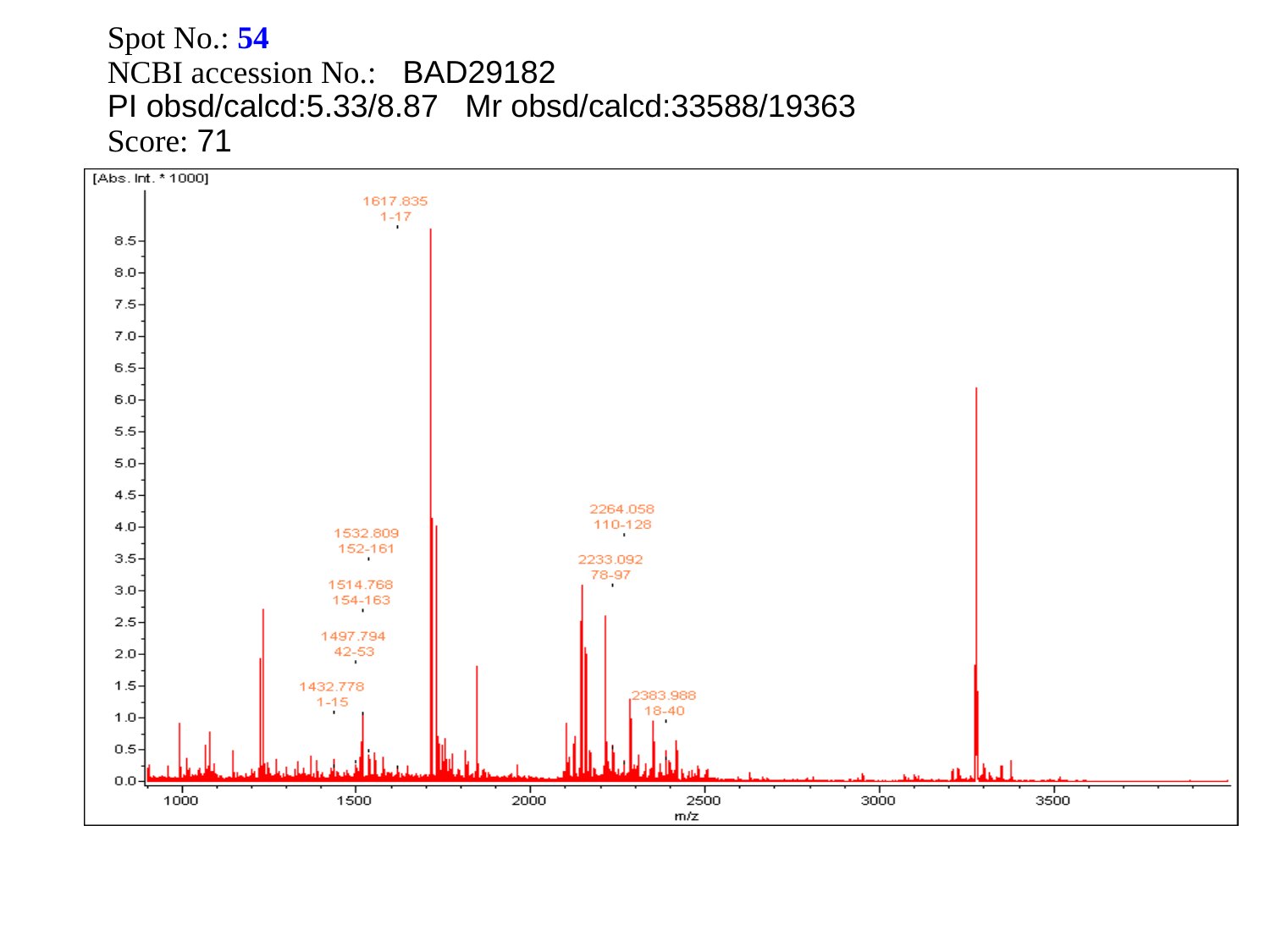

Spot No.: 54
NCBI accession No.: BAD29182
PI obsd/calcd:5.33/8.87 Mr obsd/calcd:33588/19363
Score: 71

## Slide 99
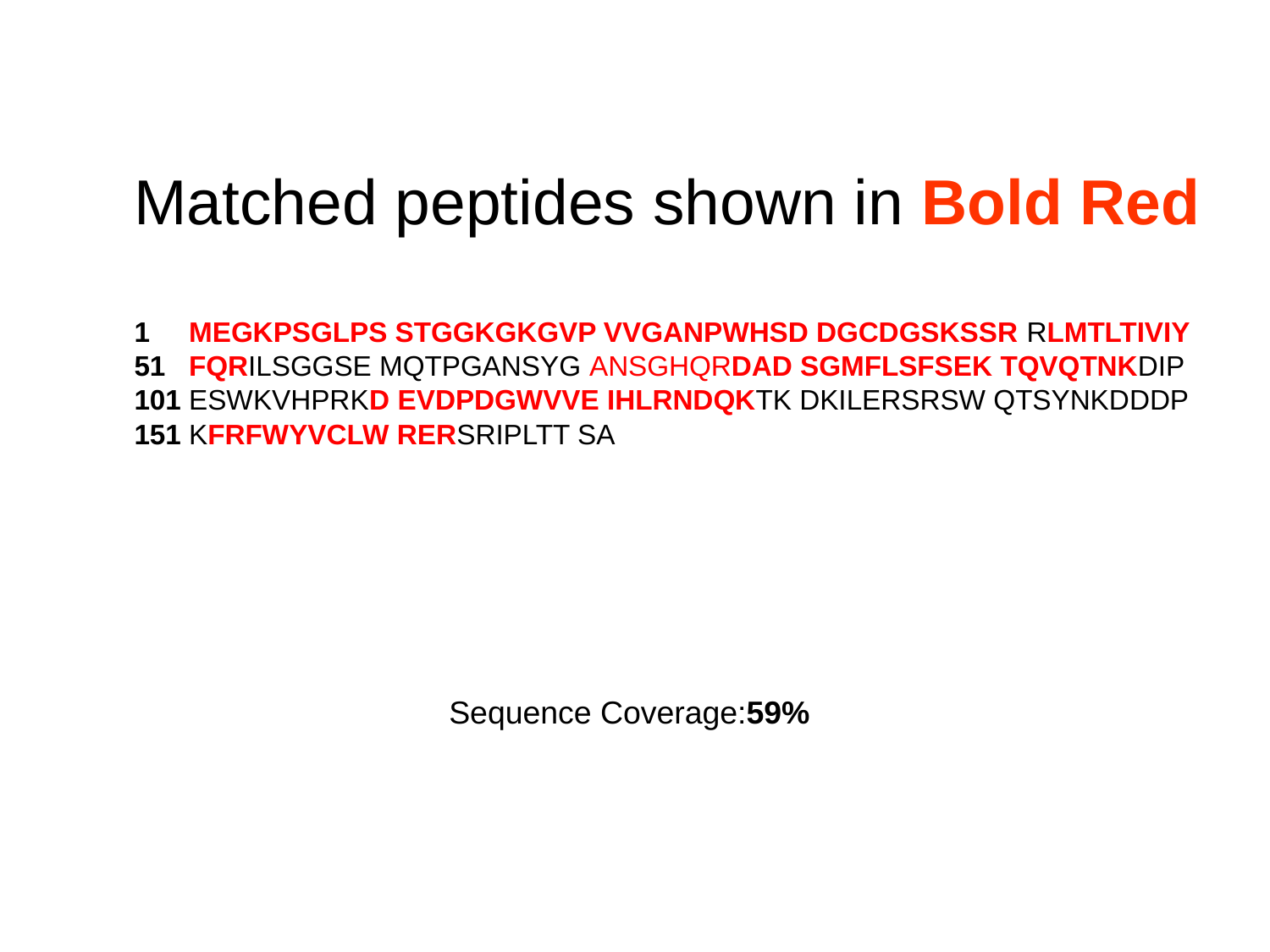

Matched peptides shown in Bold Red
1 MEGKPSGLPS STGGKGKGVP VVGANPWHSD DGCDGSKSSR RLMTLTIVIY
51 FQRILSGGSE MQTPGANSYG ANSGHQRDAD SGMFLSFSEK TQVQTNKDIP
101 ESWKVHPRKD EVDPDGWVVE IHLRNDQKTK DKILERSRSW QTSYNKDDDP
151 KFRFWYVCLW RERSRIPLTT SA
# Sequence Coverage:59%

## Slide 100
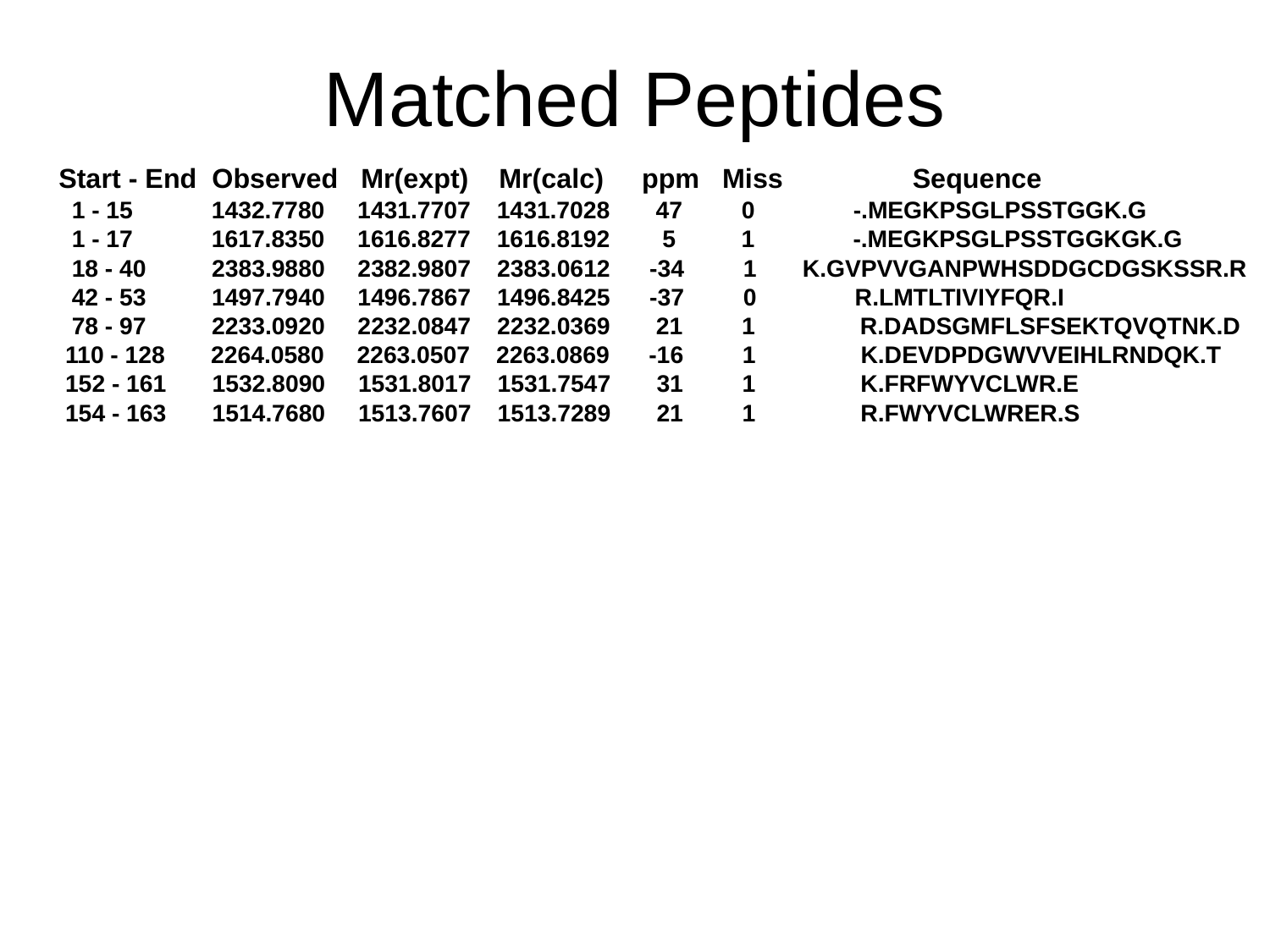

# Matched Peptides
Start - End Observed Mr(expt) Mr(calc) ppm Miss Sequence
 1 - 15 1432.7780 1431.7707 1431.7028 47 0 -.MEGKPSGLPSSTGGK.G
 1 - 17 1617.8350 1616.8277 1616.8192 5 1 -.MEGKPSGLPSSTGGKGK.G
 18 - 40 2383.9880 2382.9807 2383.0612 -34 1 K.GVPVVGANPWHSDDGCDGSKSSR.R
 42 - 53 1497.7940 1496.7867 1496.8425 -37 0 R.LMTLTIVIYFQR.I
 78 - 97 2233.0920 2232.0847 2232.0369 21 1 R.DADSGMFLSFSEKTQVQTNK.D
 110 - 128 2264.0580 2263.0507 2263.0869 -16 1 K.DEVDPDGWVVEIHLRNDQK.T
 152 - 161 1532.8090 1531.8017 1531.7547 31 1 K.FRFWYVCLWR.E
 154 - 163 1514.7680 1513.7607 1513.7289 21 1 R.FWYVCLWRER.S
