# Supplementary material for: Comparative Proteomics Analysis of the Root Apoplasts of Rice Seedlings in Response to Hydrogen Peroxide
Source: PLoS One. 2011 Feb 10;6(2):e16723. doi: 10.1371/journal.pone.0016723 (PMC3037377; doi:10.1371/journal.pone.0016723)
Supplement: File S2 — Supplemental spectra MS/MS. Annotated spectra of 21 differentially expressed protein spots identified by MS/MS. (PPT) [file pone.0016723.s010.ppt]

## Slide 1
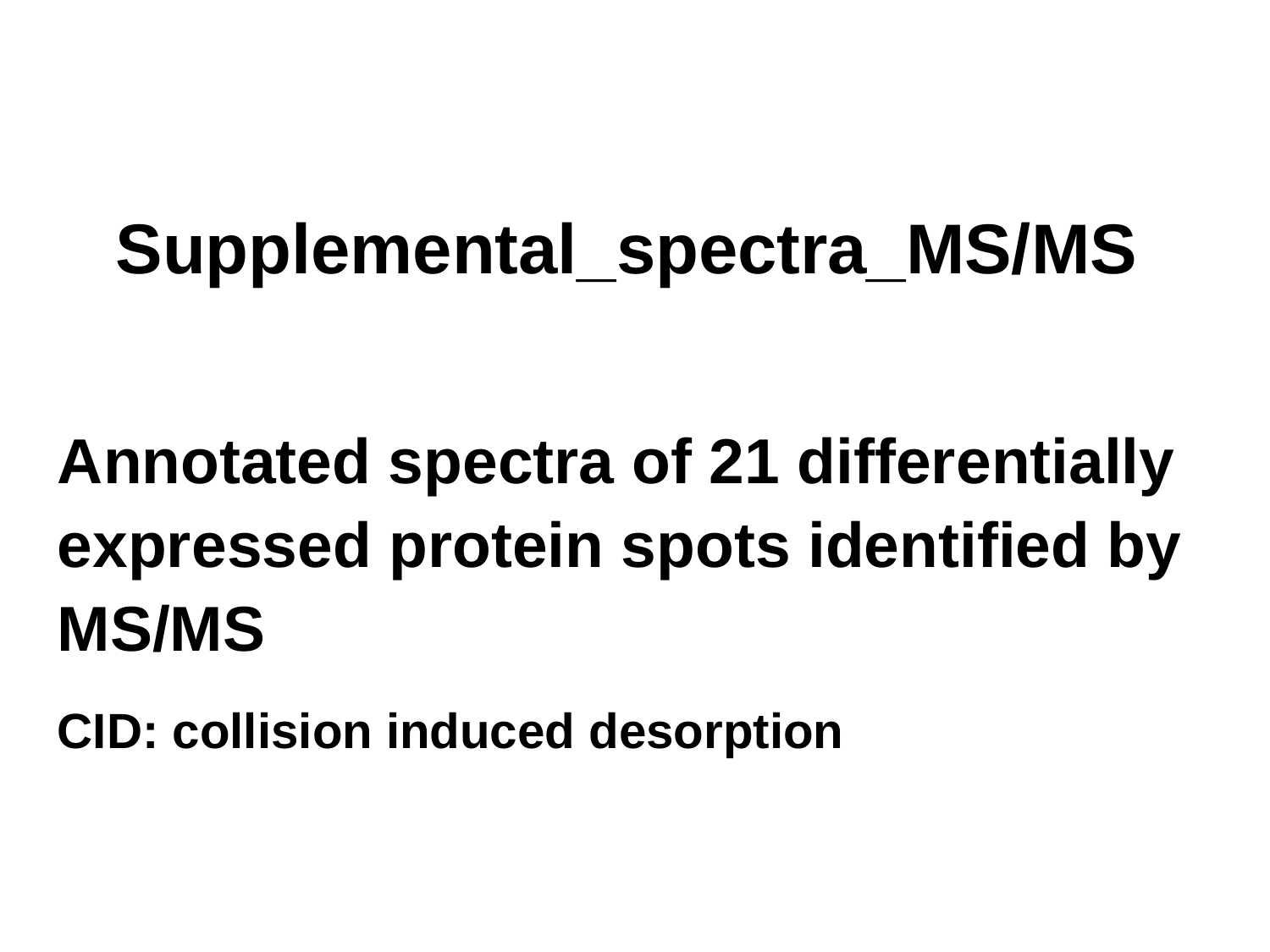

# Supplemental_spectra_MS/MS
Annotated spectra of 21 differentially expressed protein spots identified by MS/MS
CID: collision induced desorption

## Slide 2
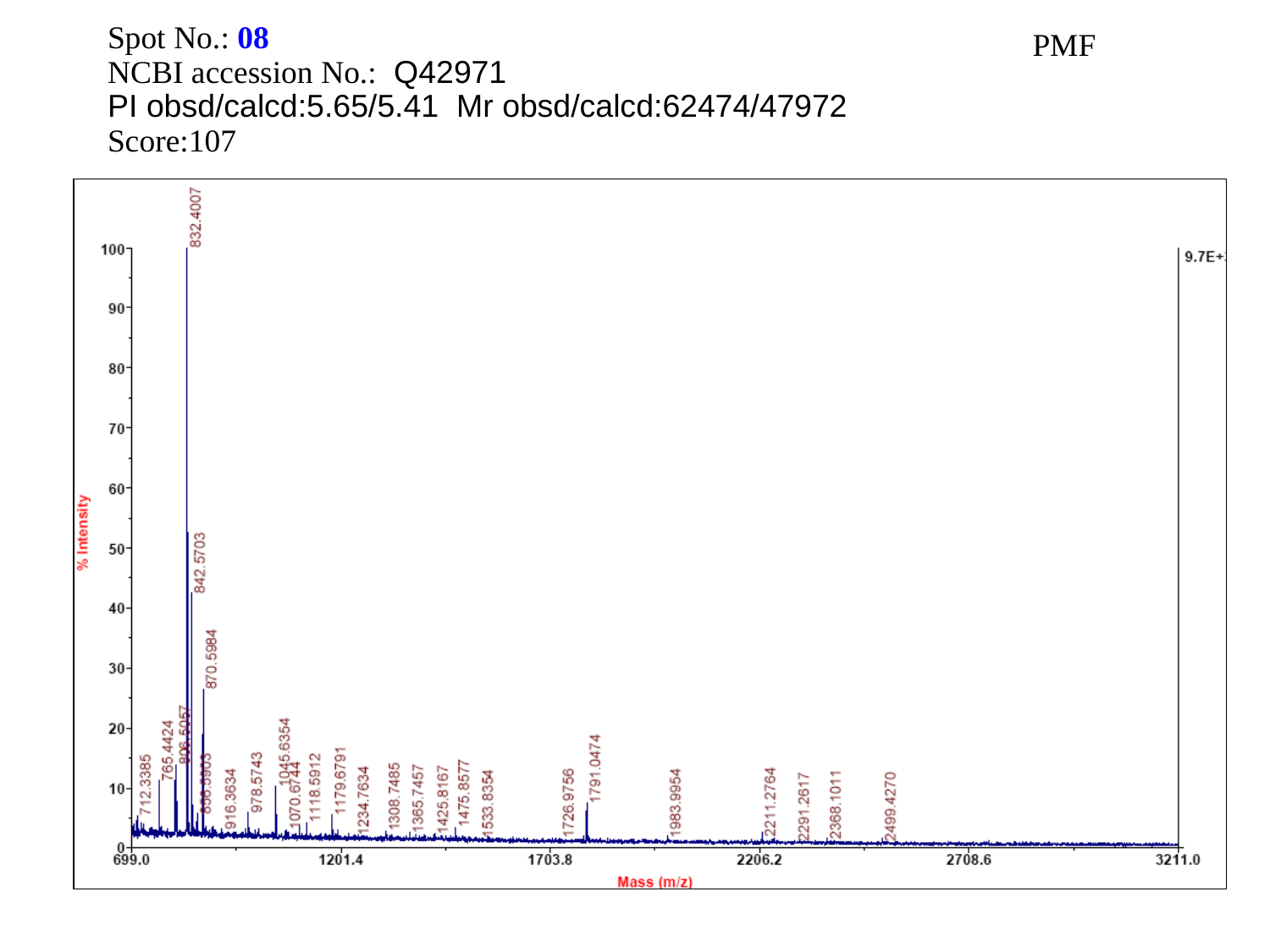

Spot No.: 08
NCBI accession No.: Q42971
PI obsd/calcd:5.65/5.41 Mr obsd/calcd:62474/47972
Score:107
PMF

## Slide 3
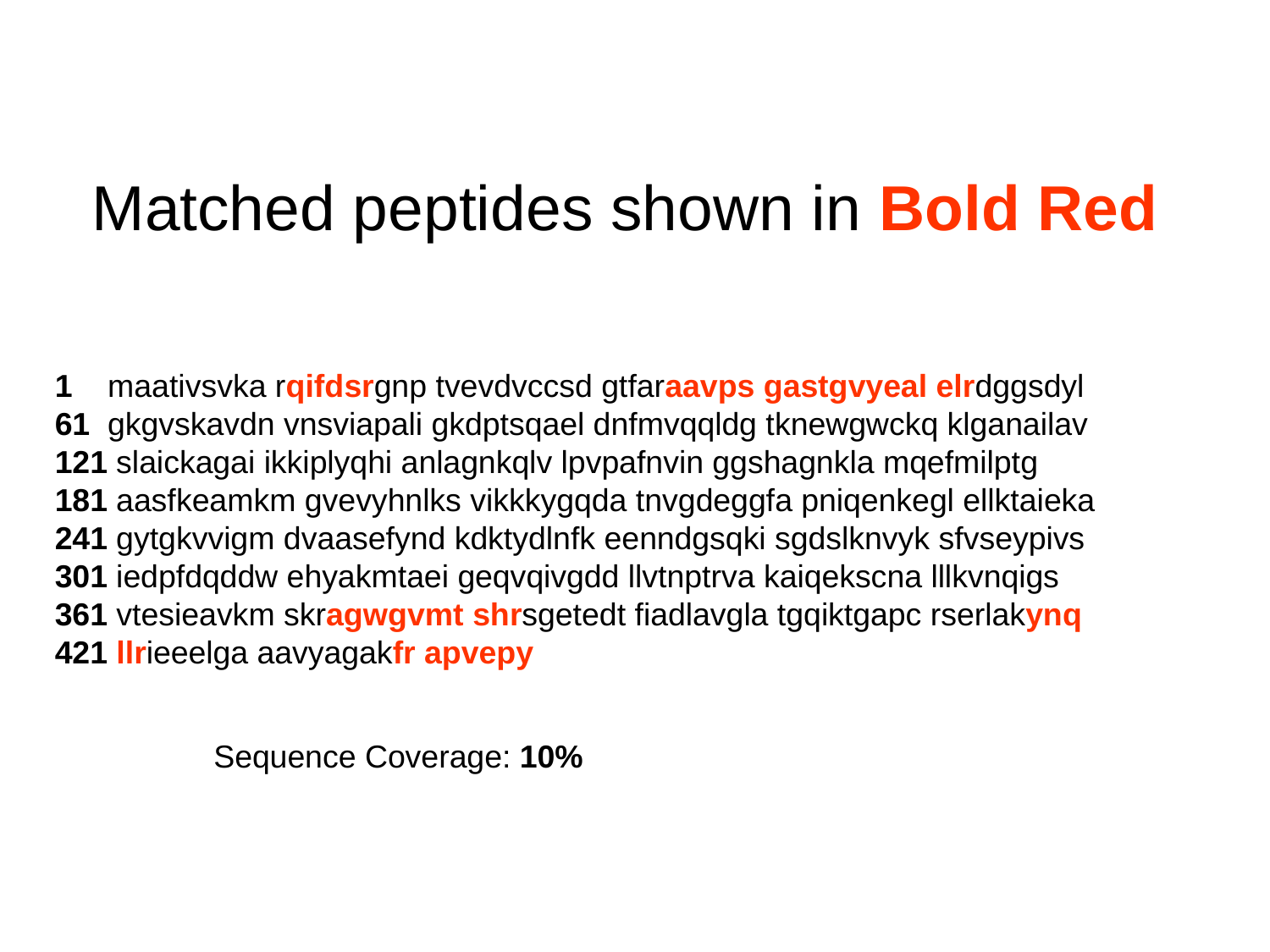

#
Matched peptides shown in Bold Red
1 maativsvka rqifdsrgnp tvevdvccsd gtfaraavps gastgvyeal elrdggsdyl
61 gkgvskavdn vnsviapali gkdptsqael dnfmvqqldg tknewgwckq klganailav
121 slaickagai ikkiplyqhi anlagnkqlv lpvpafnvin ggshagnkla mqefmilptg
181 aasfkeamkm gvevyhnlks vikkkygqda tnvgdeggfa pniqenkegl ellktaieka
241 gytgkvvigm dvaasefynd kdktydlnfk eenndgsqki sgdslknvyk sfvseypivs
301 iedpfdqddw ehyakmtaei geqvqivgdd llvtnptrva kaiqekscna lllkvnqigs
361 vtesieavkm skragwgvmt shrsgetedt fiadlavgla tgqiktgapc rserlakynq
421 llrieeelga aavyagakfr apvepy
Sequence Coverage: 10%

## Slide 4
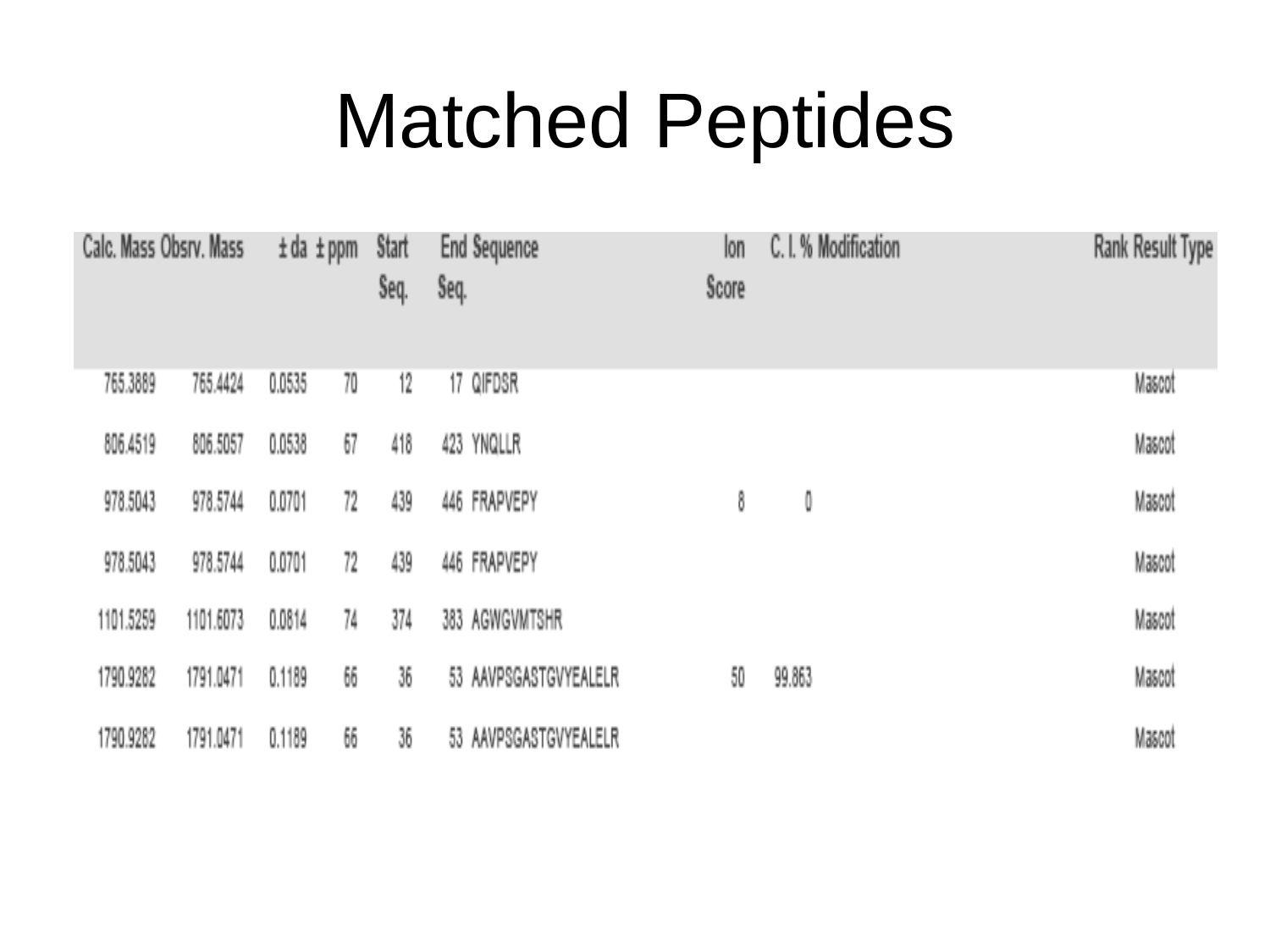

# Matched Peptides

## Slide 5
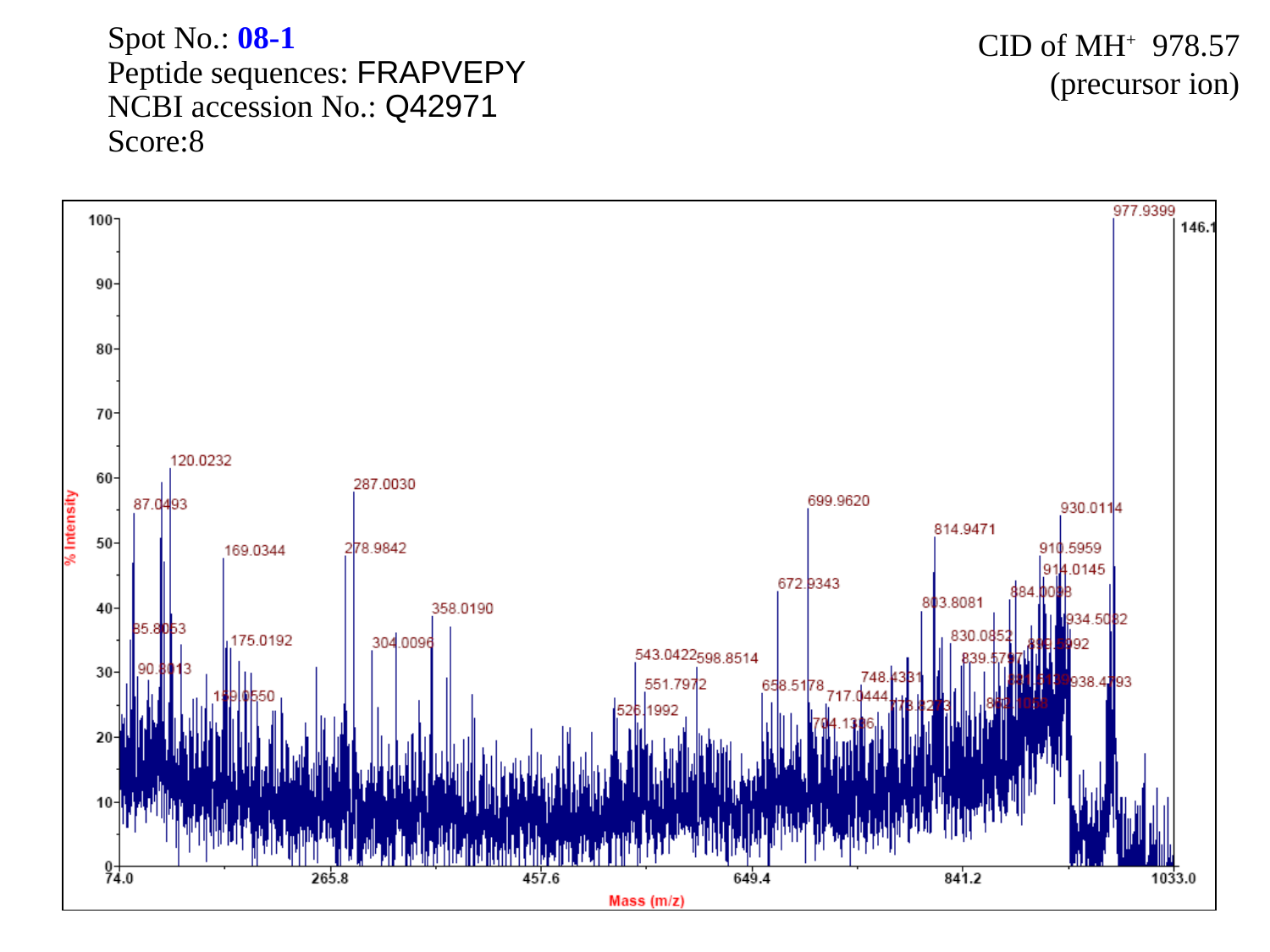

Spot No.: 08-1
Peptide sequences: FRAPVEPY
NCBI accession No.: Q42971
Score:8
CID of MH+ 978.57 (precursor ion)

## Slide 6
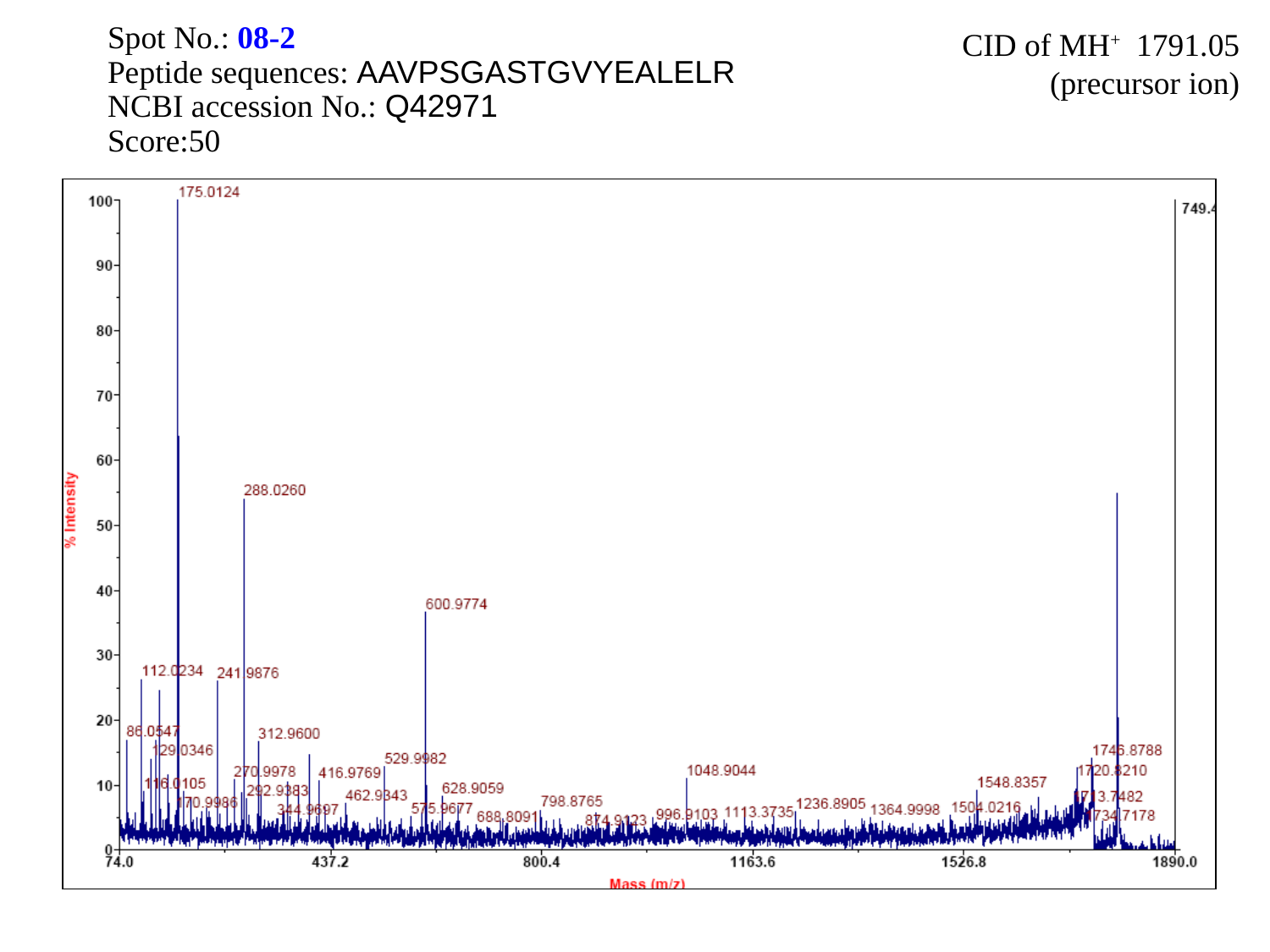

Spot No.: 08-2
Peptide sequences: AAVPSGASTGVYEALELR
NCBI accession No.: Q42971
Score:50
CID of MH+ 1791.05 (precursor ion)

## Slide 7
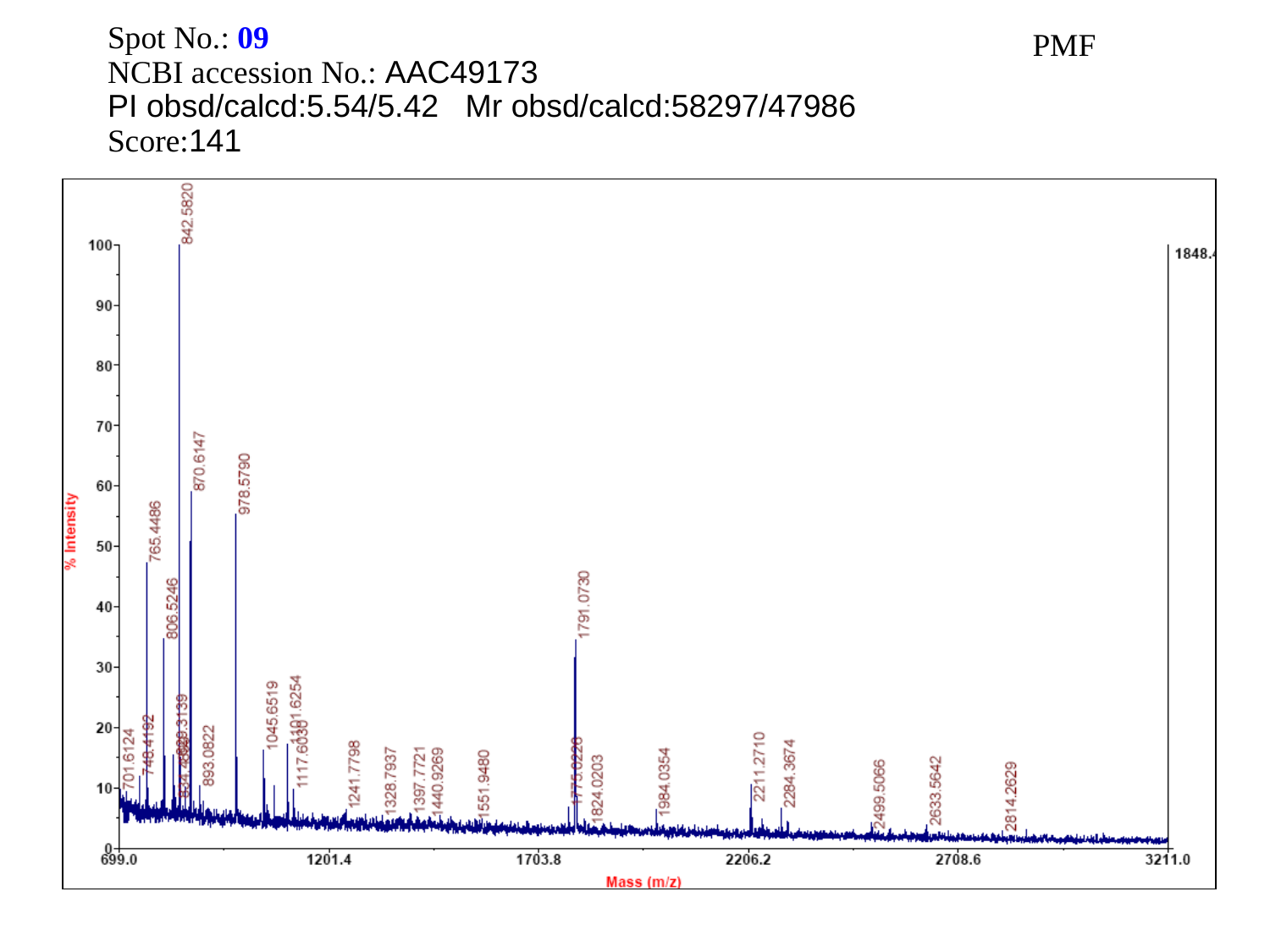

Spot No.: 09
NCBI accession No.: AAC49173
PI obsd/calcd:5.54/5.42 Mr obsd/calcd:58297/47986
Score:141
PMF

## Slide 8
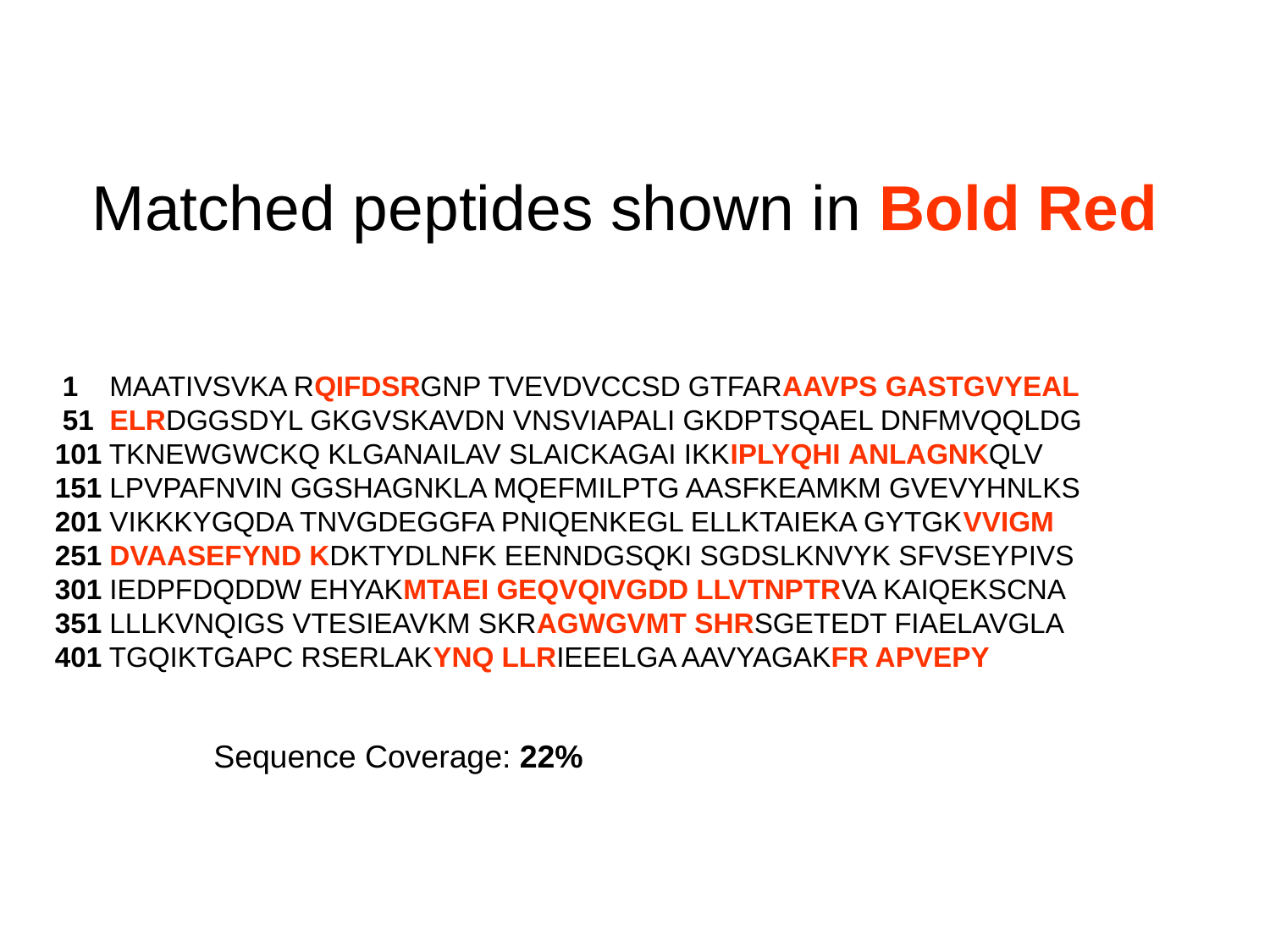

#
Matched peptides shown in Bold Red
 1 MAATIVSVKA RQIFDSRGNP TVEVDVCCSD GTFARAAVPS GASTGVYEAL
 51 ELRDGGSDYL GKGVSKAVDN VNSVIAPALI GKDPTSQAEL DNFMVQQLDG
101 TKNEWGWCKQ KLGANAILAV SLAICKAGAI IKKIPLYQHI ANLAGNKQLV
151 LPVPAFNVIN GGSHAGNKLA MQEFMILPTG AASFKEAMKM GVEVYHNLKS
201 VIKKKYGQDA TNVGDEGGFA PNIQENKEGL ELLKTAIEKA GYTGKVVIGM
251 DVAASEFYND KDKTYDLNFK EENNDGSQKI SGDSLKNVYK SFVSEYPIVS
301 IEDPFDQDDW EHYAKMTAEI GEQVQIVGDD LLVTNPTRVA KAIQEKSCNA
351 LLLKVNQIGS VTESIEAVKM SKRAGWGVMT SHRSGETEDT FIAELAVGLA
401 TGQIKTGAPC RSERLAKYNQ LLRIEEELGA AAVYAGAKFR APVEPY
Sequence Coverage: 22%

## Slide 9
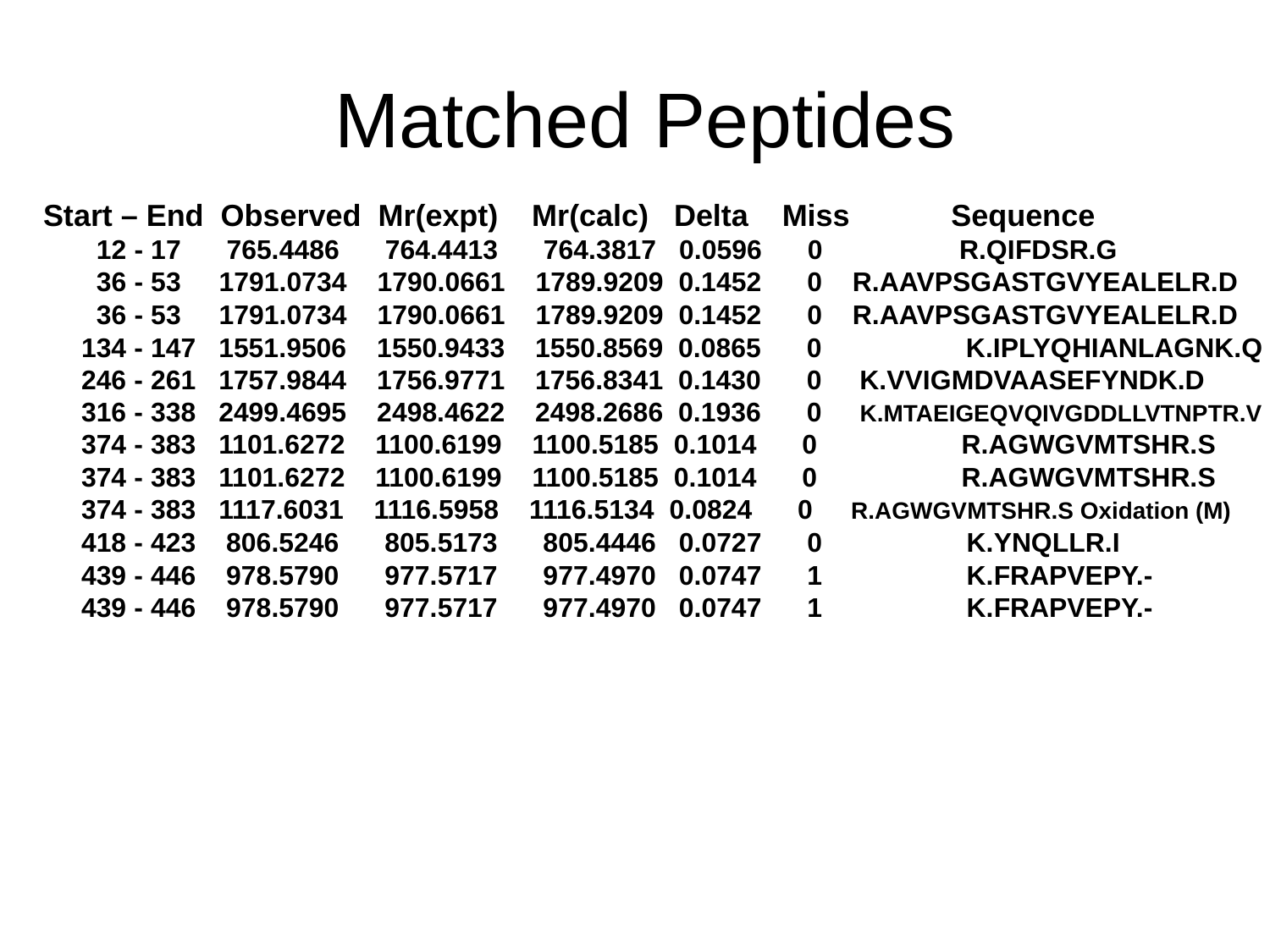

# Matched Peptides
Start – End Observed Mr(expt) Mr(calc) Delta Miss Sequence
 12 - 17 765.4486 764.4413 764.3817 0.0596 0 R.QIFDSR.G
 36 - 53 1791.0734 1790.0661 1789.9209 0.1452 0 R.AAVPSGASTGVYEALELR.D
 36 - 53 1791.0734 1790.0661 1789.9209 0.1452 0 R.AAVPSGASTGVYEALELR.D
 134 - 147 1551.9506 1550.9433 1550.8569 0.0865 0 K.IPLYQHIANLAGNK.Q
 246 - 261 1757.9844 1756.9771 1756.8341 0.1430 0 K.VVIGMDVAASEFYNDK.D
 316 - 338 2499.4695 2498.4622 2498.2686 0.1936 0 K.MTAEIGEQVQIVGDDLLVTNPTR.V
 374 - 383 1101.6272 1100.6199 1100.5185 0.1014 0 R.AGWGVMTSHR.S
 374 - 383 1101.6272 1100.6199 1100.5185 0.1014 0 R.AGWGVMTSHR.S
 374 - 383 1117.6031 1116.5958 1116.5134 0.0824 0 R.AGWGVMTSHR.S Oxidation (M)
 418 - 423 806.5246 805.5173 805.4446 0.0727 0 K.YNQLLR.I
 439 - 446 978.5790 977.5717 977.4970 0.0747 1 K.FRAPVEPY.-
 439 - 446 978.5790 977.5717 977.4970 0.0747 1 K.FRAPVEPY.-

## Slide 10
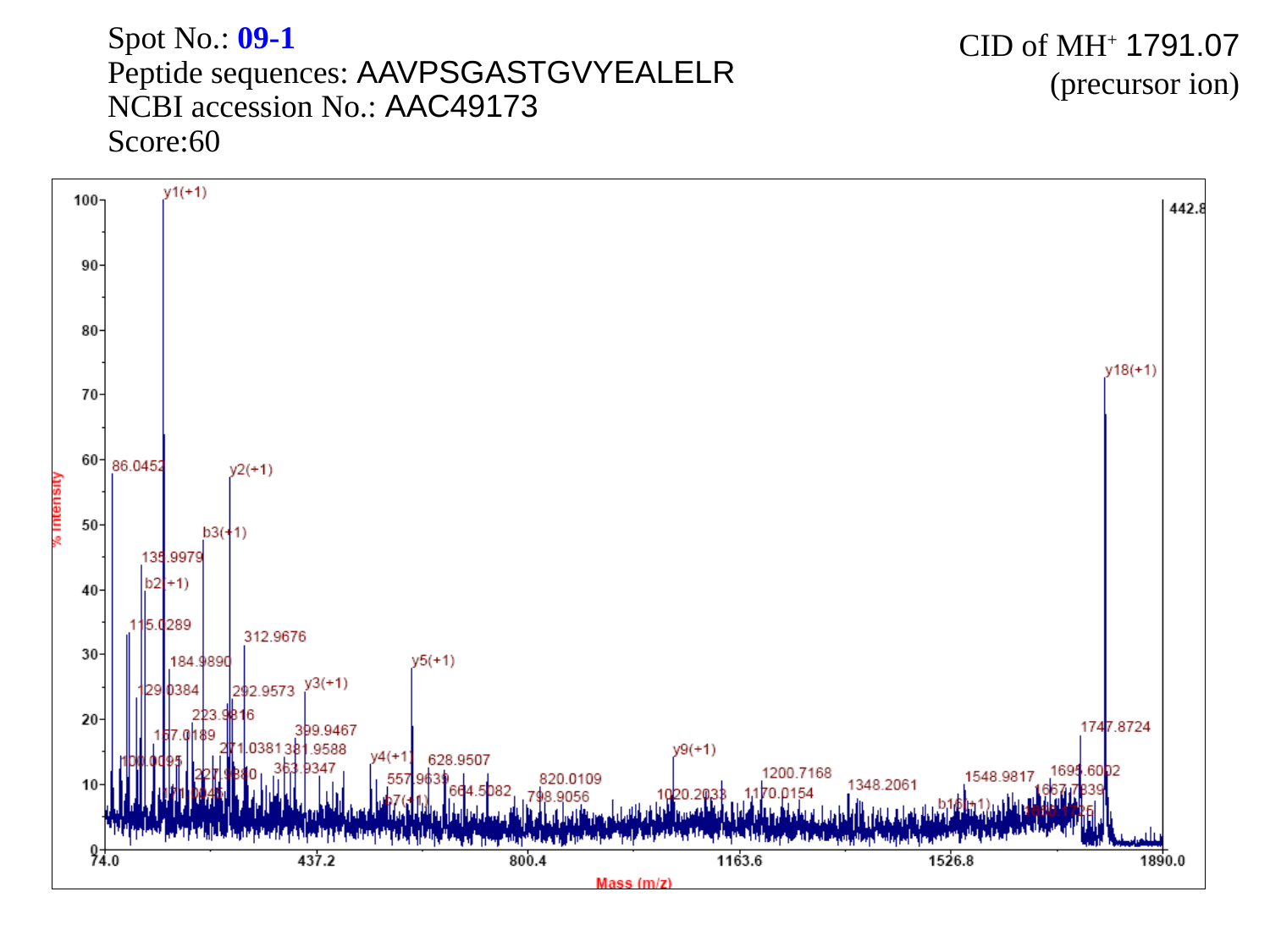

Spot No.: 09-1
Peptide sequences: AAVPSGASTGVYEALELR
NCBI accession No.: AAC49173
Score:60
CID of MH+ 1791.07 (precursor ion)

## Slide 11
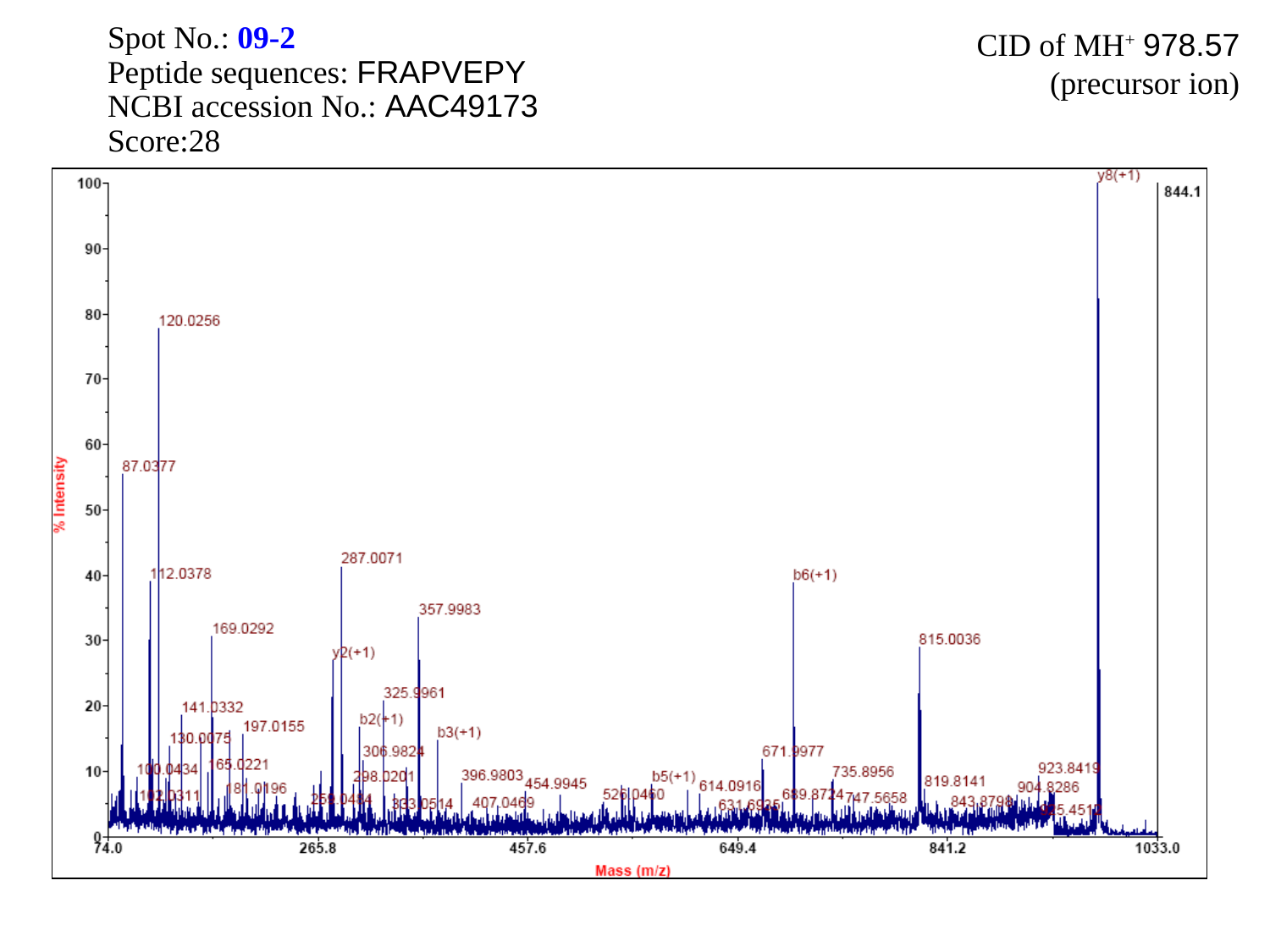

Spot No.: 09-2
Peptide sequences: FRAPVEPY
NCBI accession No.: AAC49173
Score:28
CID of MH+ 978.57 (precursor ion)

## Slide 12
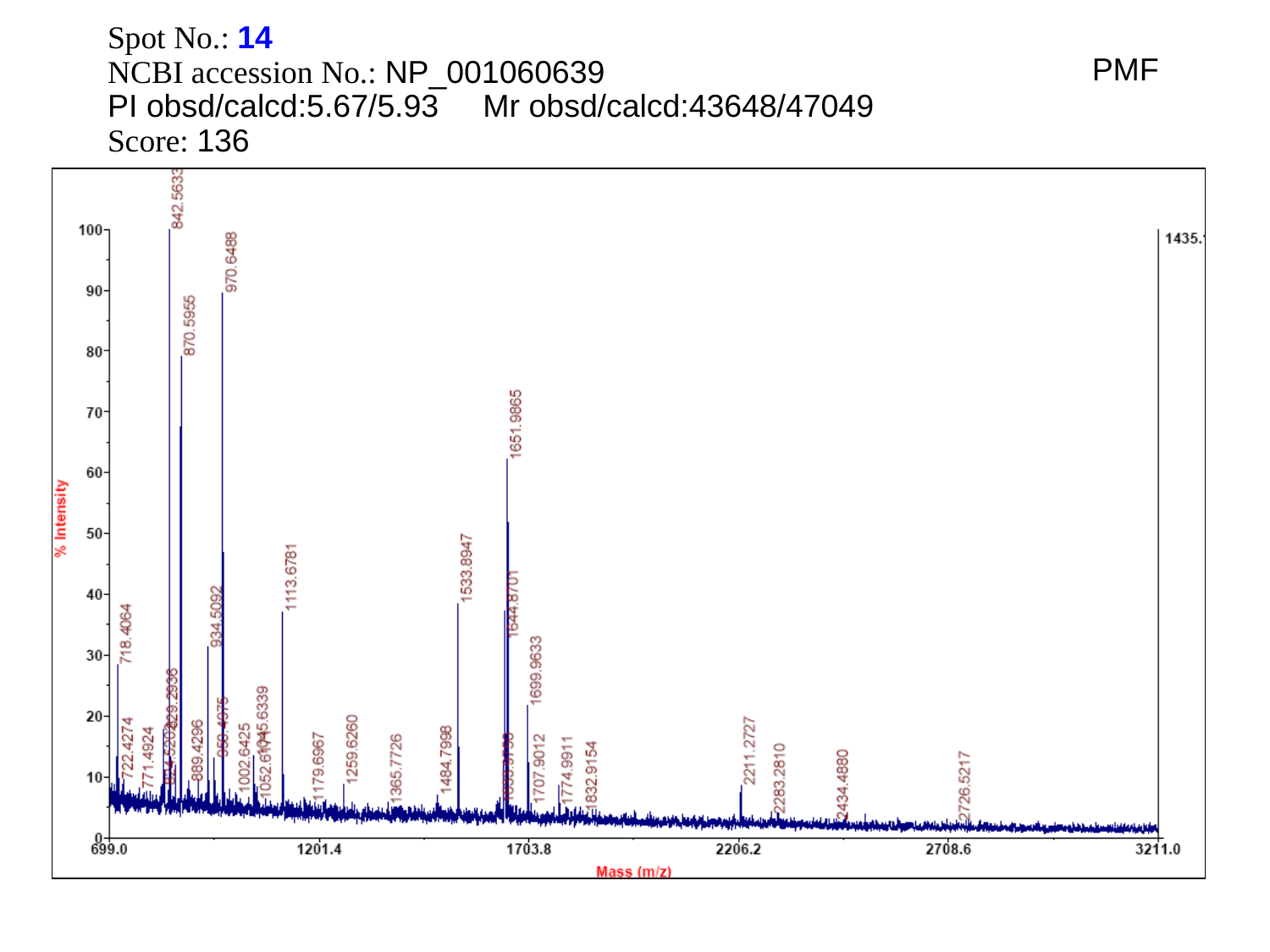

Spot No.: 14
NCBI accession No.: NP_001060639
PI obsd/calcd:5.67/5.93 Mr obsd/calcd:43648/47049
Score: 136
PMF

## Slide 13
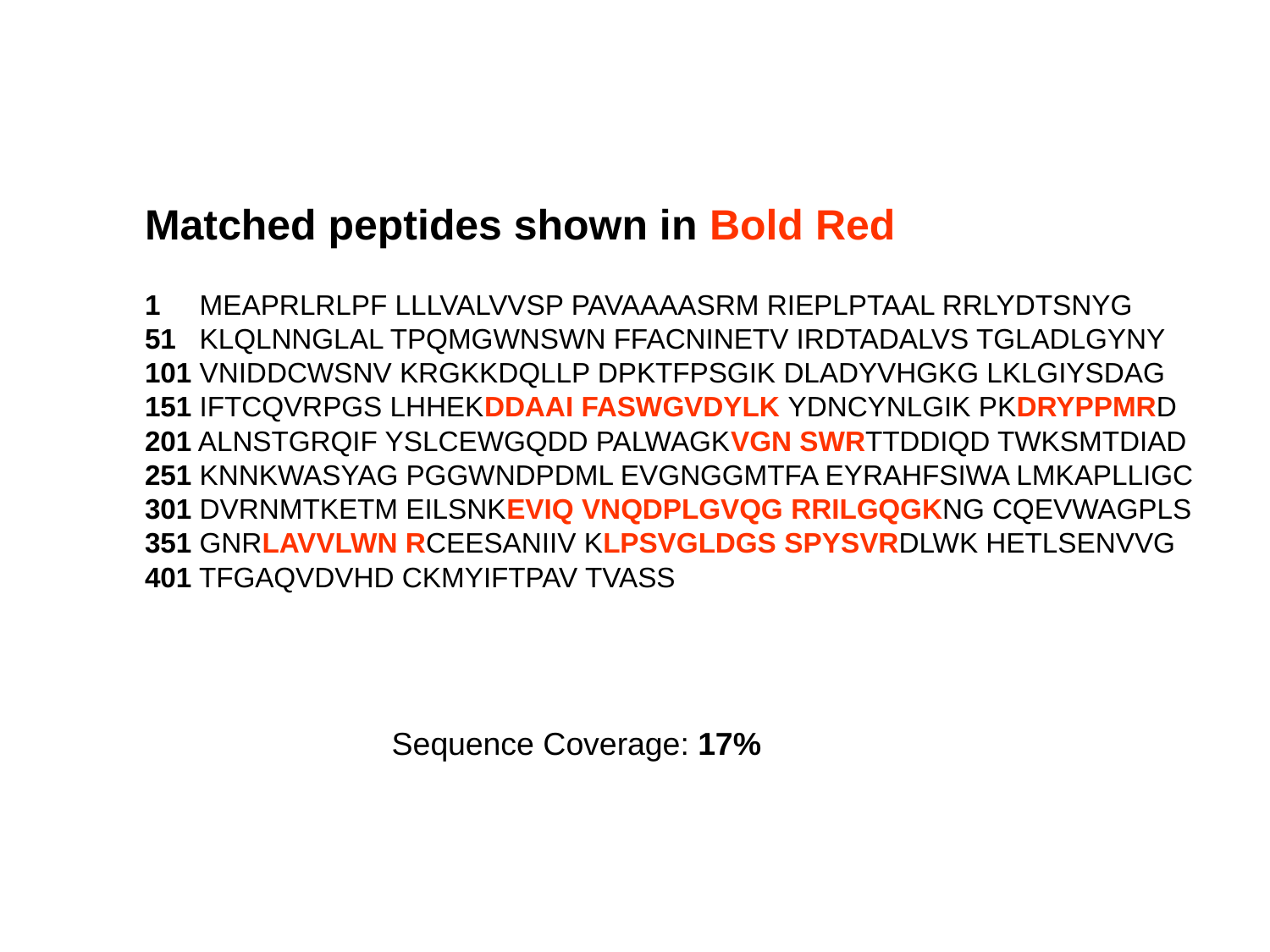

Matched peptides shown in Bold Red
1 MEAPRLRLPF LLLVALVVSP PAVAAAASRM RIEPLPTAAL RRLYDTSNYG
51 KLQLNNGLAL TPQMGWNSWN FFACNINETV IRDTADALVS TGLADLGYNY
101 VNIDDCWSNV KRGKKDQLLP DPKTFPSGIK DLADYVHGKG LKLGIYSDAG
151 IFTCQVRPGS LHHEKDDAAI FASWGVDYLK YDNCYNLGIK PKDRYPPMRD
201 ALNSTGRQIF YSLCEWGQDD PALWAGKVGN SWRTTDDIQD TWKSMTDIAD
251 KNNKWASYAG PGGWNDPDML EVGNGGMTFA EYRAHFSIWA LMKAPLLIGC
301 DVRNMTKETM EILSNKEVIQ VNQDPLGVQG RRILGQGKNG CQEVWAGPLS
351 GNRLAVVLWN RCEESANIIV KLPSVGLDGS SPYSVRDLWK HETLSENVVG
401 TFGAQVDVHD CKMYIFTPAV TVASS
# Sequence Coverage: 17%

## Slide 14
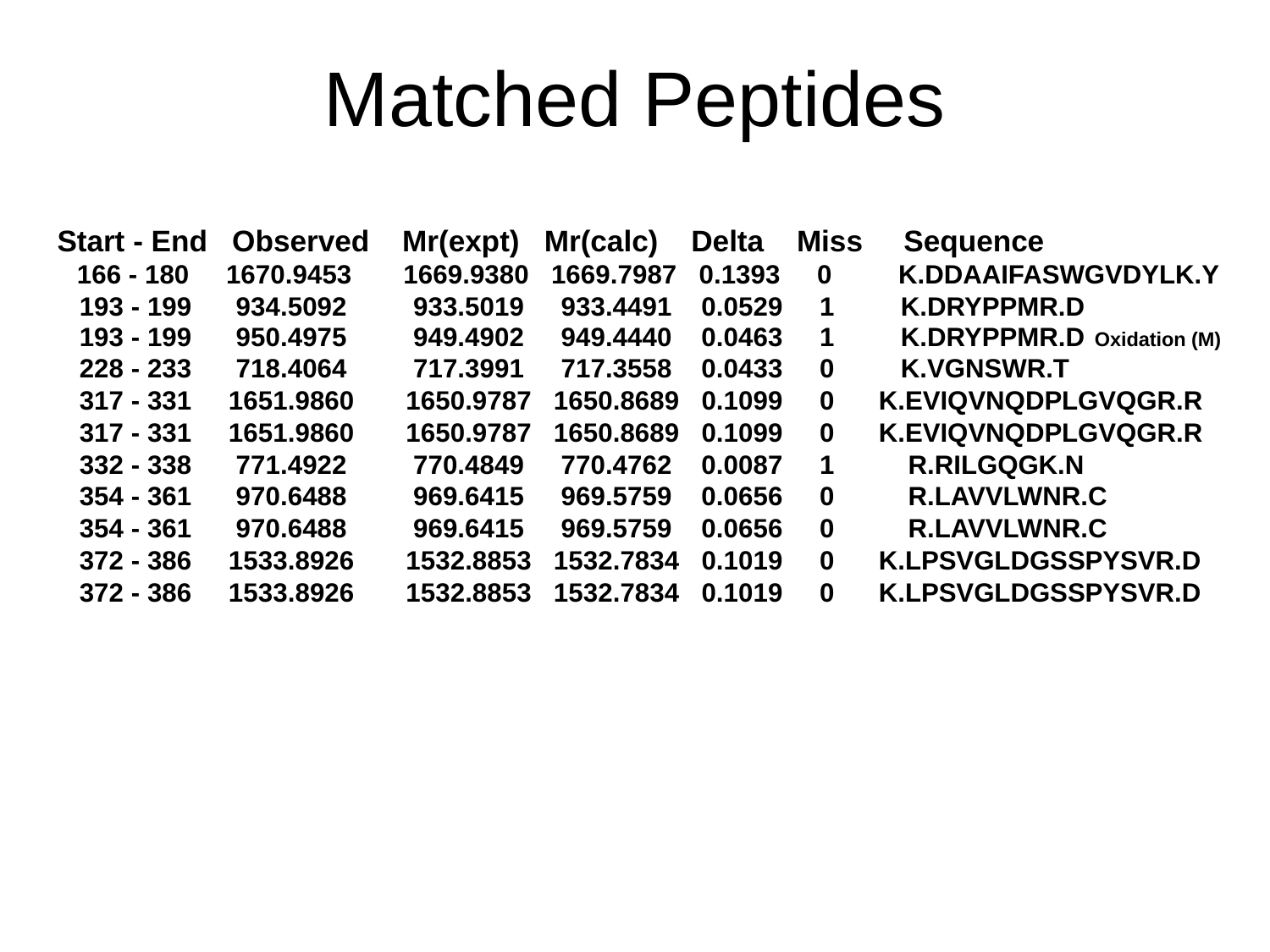

# Matched Peptides
Start - End Observed Mr(expt) Mr(calc) Delta Miss Sequence
 166 - 180 1670.9453 1669.9380 1669.7987 0.1393 0 K.DDAAIFASWGVDYLK.Y
 193 - 199 934.5092 933.5019 933.4491 0.0529 1 K.DRYPPMR.D
 193 - 199 950.4975 949.4902 949.4440 0.0463 1 K.DRYPPMR.D Oxidation (M)
 228 - 233 718.4064 717.3991 717.3558 0.0433 0 K.VGNSWR.T
 317 - 331 1651.9860 1650.9787 1650.8689 0.1099 0 K.EVIQVNQDPLGVQGR.R
 317 - 331 1651.9860 1650.9787 1650.8689 0.1099 0 K.EVIQVNQDPLGVQGR.R
 332 - 338 771.4922 770.4849 770.4762 0.0087 1 R.RILGQGK.N
 354 - 361 970.6488 969.6415 969.5759 0.0656 0 R.LAVVLWNR.C
 354 - 361 970.6488 969.6415 969.5759 0.0656 0 R.LAVVLWNR.C
 372 - 386 1533.8926 1532.8853 1532.7834 0.1019 0 K.LPSVGLDGSSPYSVR.D
 372 - 386 1533.8926 1532.8853 1532.7834 0.1019 0 K.LPSVGLDGSSPYSVR.D

## Slide 15
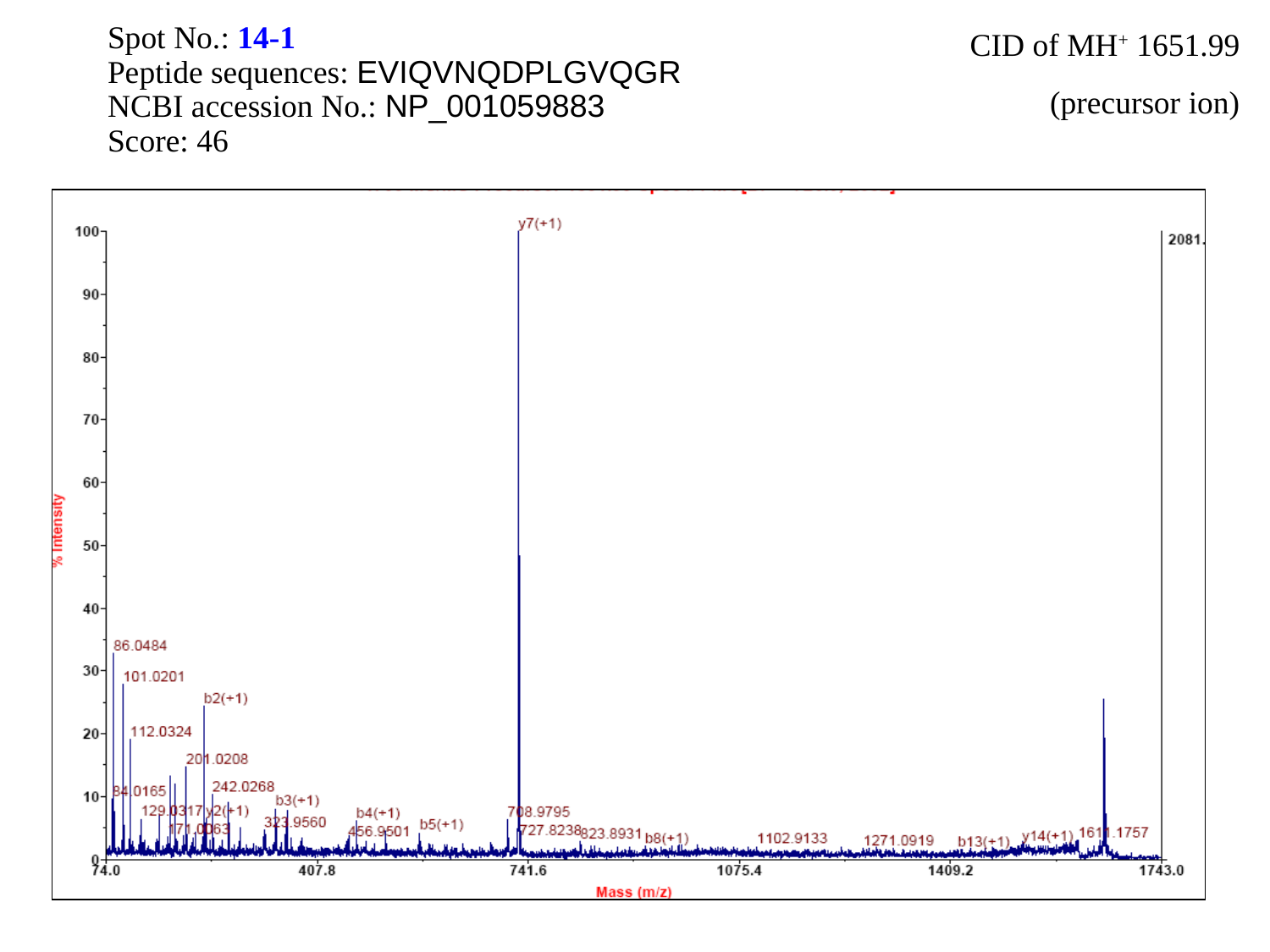

Spot No.: 14-1
Peptide sequences: EVIQVNQDPLGVQGR
NCBI accession No.: NP_001059883
Score: 46
CID of MH+ 1651.99
(precursor ion)

## Slide 16
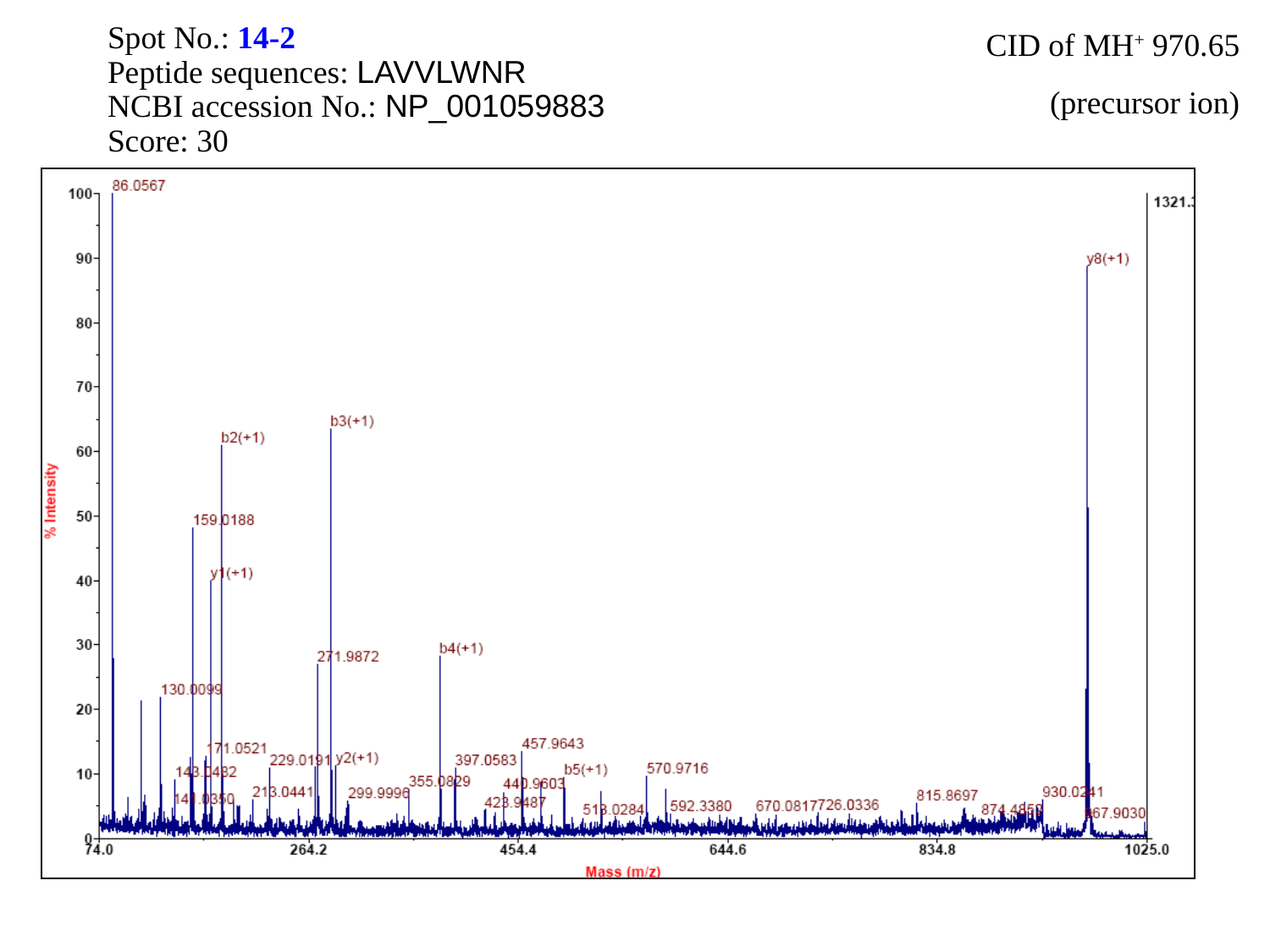

Spot No.: 14-2
Peptide sequences: LAVVLWNR
NCBI accession No.: NP_001059883
Score: 30
CID of MH+ 970.65
(precursor ion)

## Slide 17
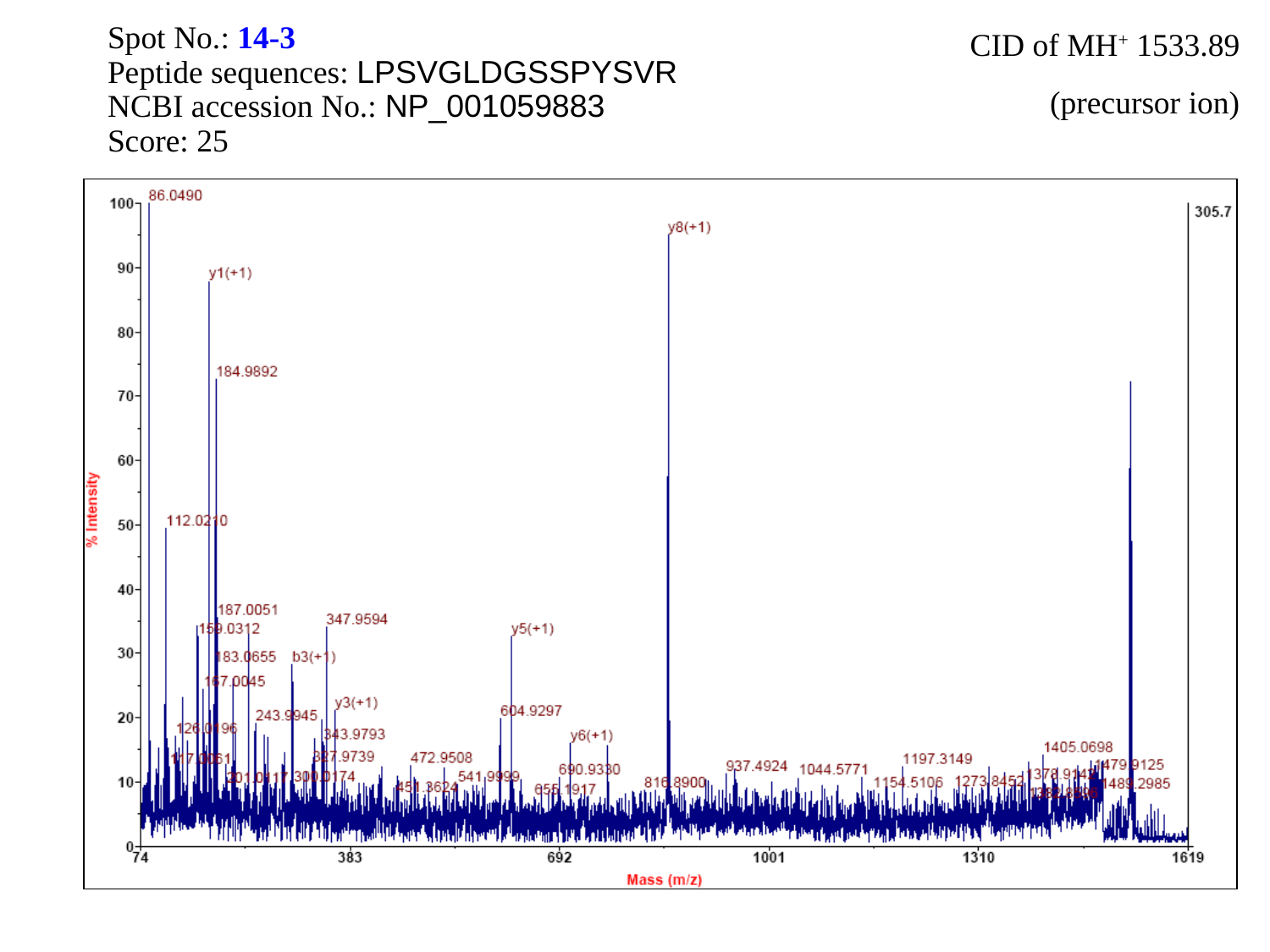

Spot No.: 14-3
Peptide sequences: LPSVGLDGSSPYSVR
NCBI accession No.: NP_001059883
Score: 25
CID of MH+ 1533.89
(precursor ion)

## Slide 18
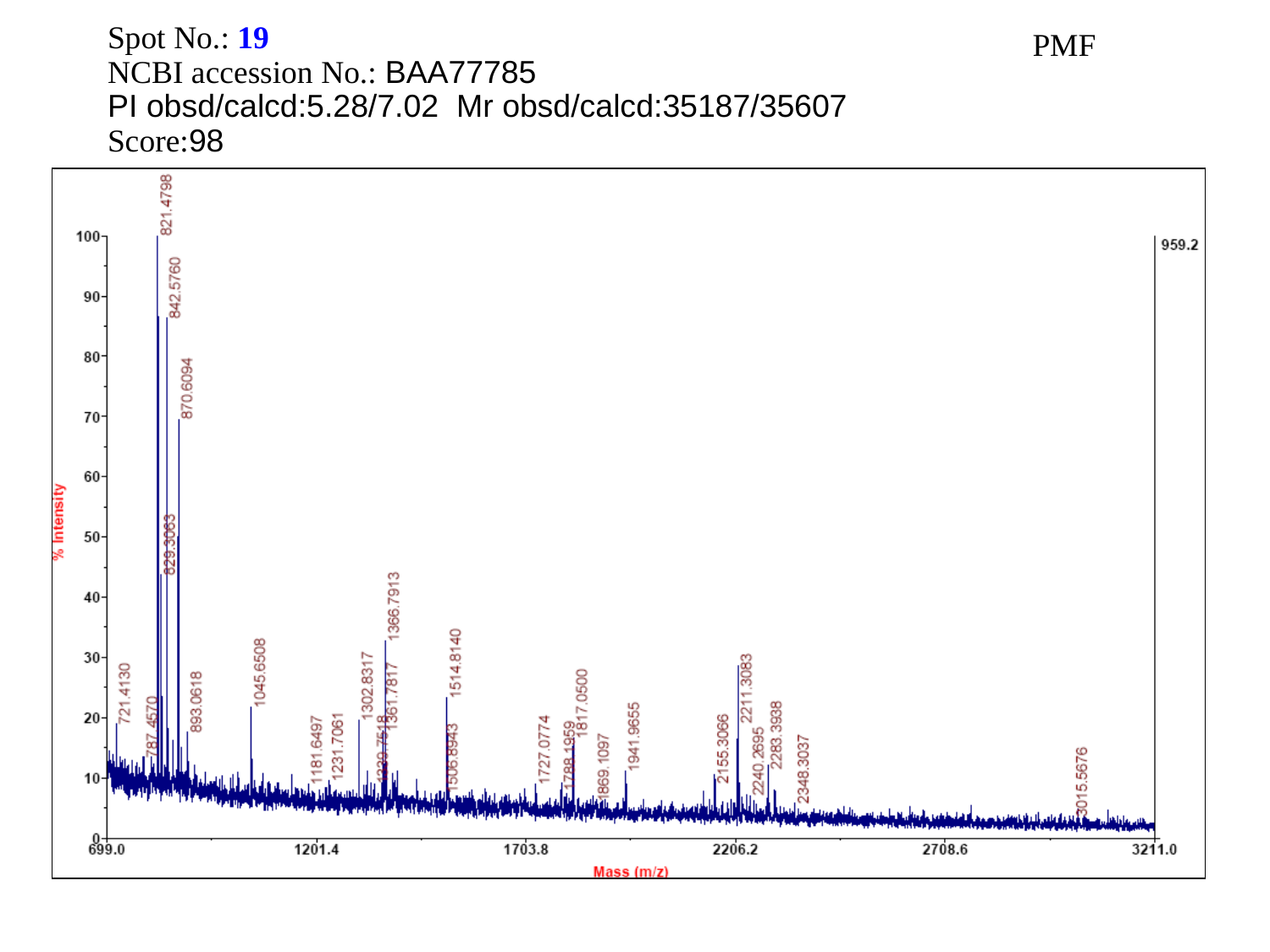

Spot No.: 19
NCBI accession No.: BAA77785
PI obsd/calcd:5.28/7.02 Mr obsd/calcd:35187/35607
Score:98
PMF

## Slide 19
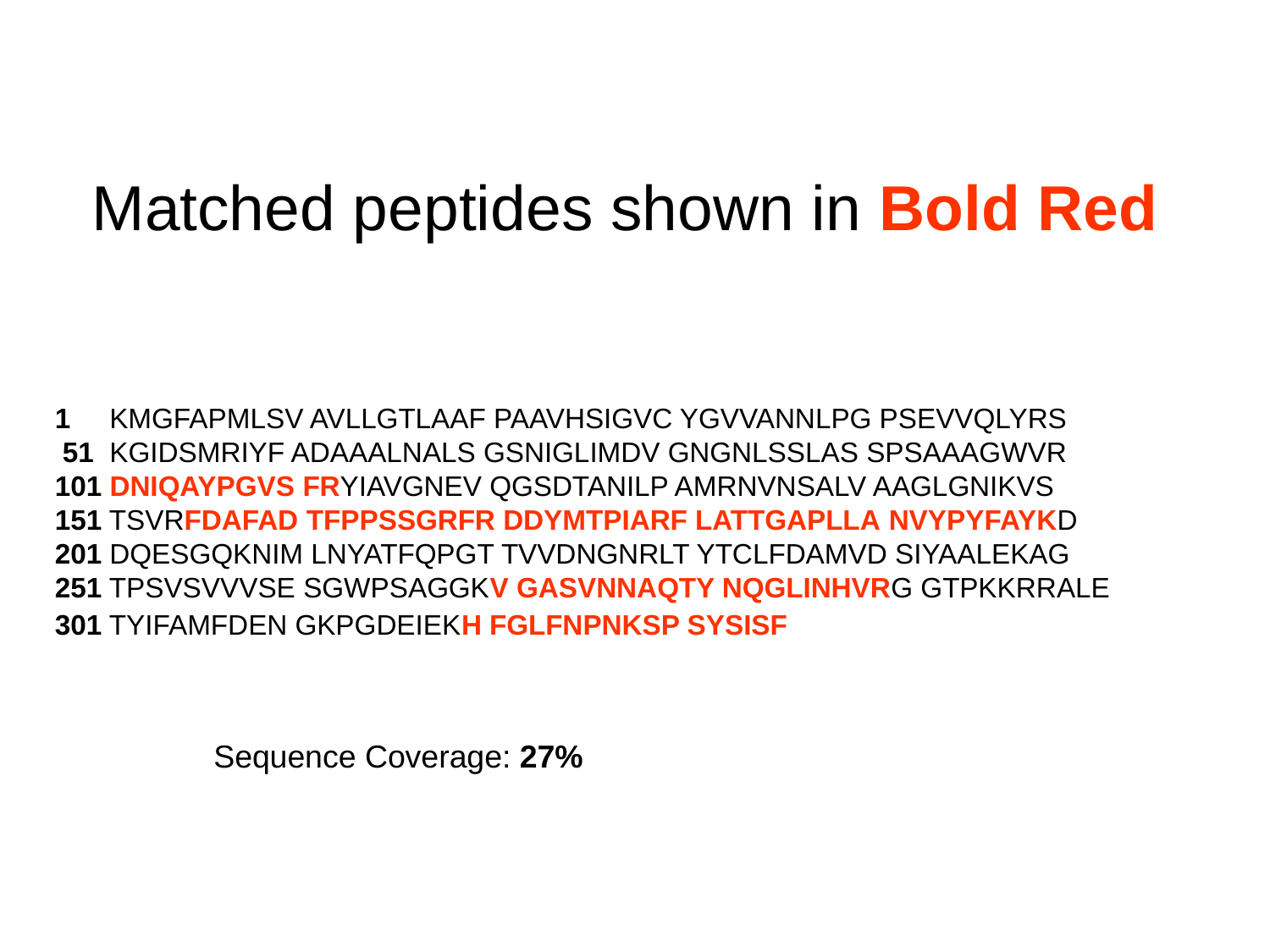

#
Matched peptides shown in Bold Red
1 KMGFAPMLSV AVLLGTLAAF PAAVHSIGVC YGVVANNLPG PSEVVQLYRS
 51 KGIDSMRIYF ADAAALNALS GSNIGLIMDV GNGNLSSLAS SPSAAAGWVR
101 DNIQAYPGVS FRYIAVGNEV QGSDTANILP AMRNVNSALV AAGLGNIKVS
151 TSVRFDAFAD TFPPSSGRFR DDYMTPIARF LATTGAPLLA NVYPYFAYKD
201 DQESGQKNIM LNYATFQPGT TVVDNGNRLT YTCLFDAMVD SIYAALEKAG
251 TPSVSVVVSE SGWPSAGGKV GASVNNAQTY NQGLINHVRG GTPKKRRALE
301 TYIFAMFDEN GKPGDEIEKH FGLFNPNKSP SYSISF
Sequence Coverage: 27%

## Slide 20
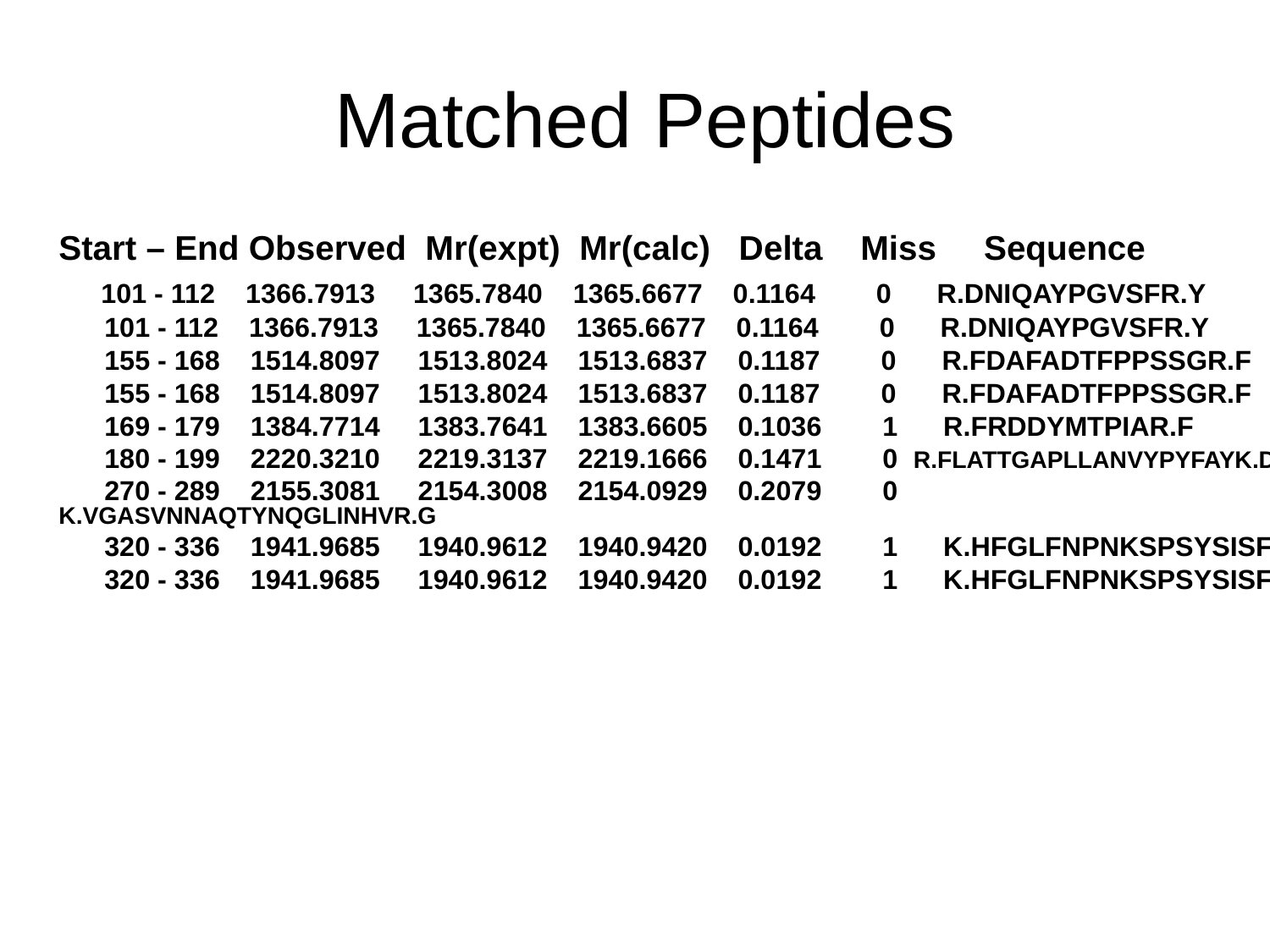

# Matched Peptides
Start – End Observed Mr(expt) Mr(calc) Delta Miss Sequence
 101 - 112 1366.7913 1365.7840 1365.6677 0.1164 0 R.DNIQAYPGVSFR.Y
 101 - 112 1366.7913 1365.7840 1365.6677 0.1164 0 R.DNIQAYPGVSFR.Y
 155 - 168 1514.8097 1513.8024 1513.6837 0.1187 0 R.FDAFADTFPPSSGR.F
 155 - 168 1514.8097 1513.8024 1513.6837 0.1187 0 R.FDAFADTFPPSSGR.F
 169 - 179 1384.7714 1383.7641 1383.6605 0.1036 1 R.FRDDYMTPIAR.F
 180 - 199 2220.3210 2219.3137 2219.1666 0.1471 0 R.FLATTGAPLLANVYPYFAYK.D
 270 - 289 2155.3081 2154.3008 2154.0929 0.2079 0 K.VGASVNNAQTYNQGLINHVR.G
 320 - 336 1941.9685 1940.9612 1940.9420 0.0192 1 K.HFGLFNPNKSPSYSISF.-
 320 - 336 1941.9685 1940.9612 1940.9420 0.0192 1 K.HFGLFNPNKSPSYSISF.-

## Slide 21
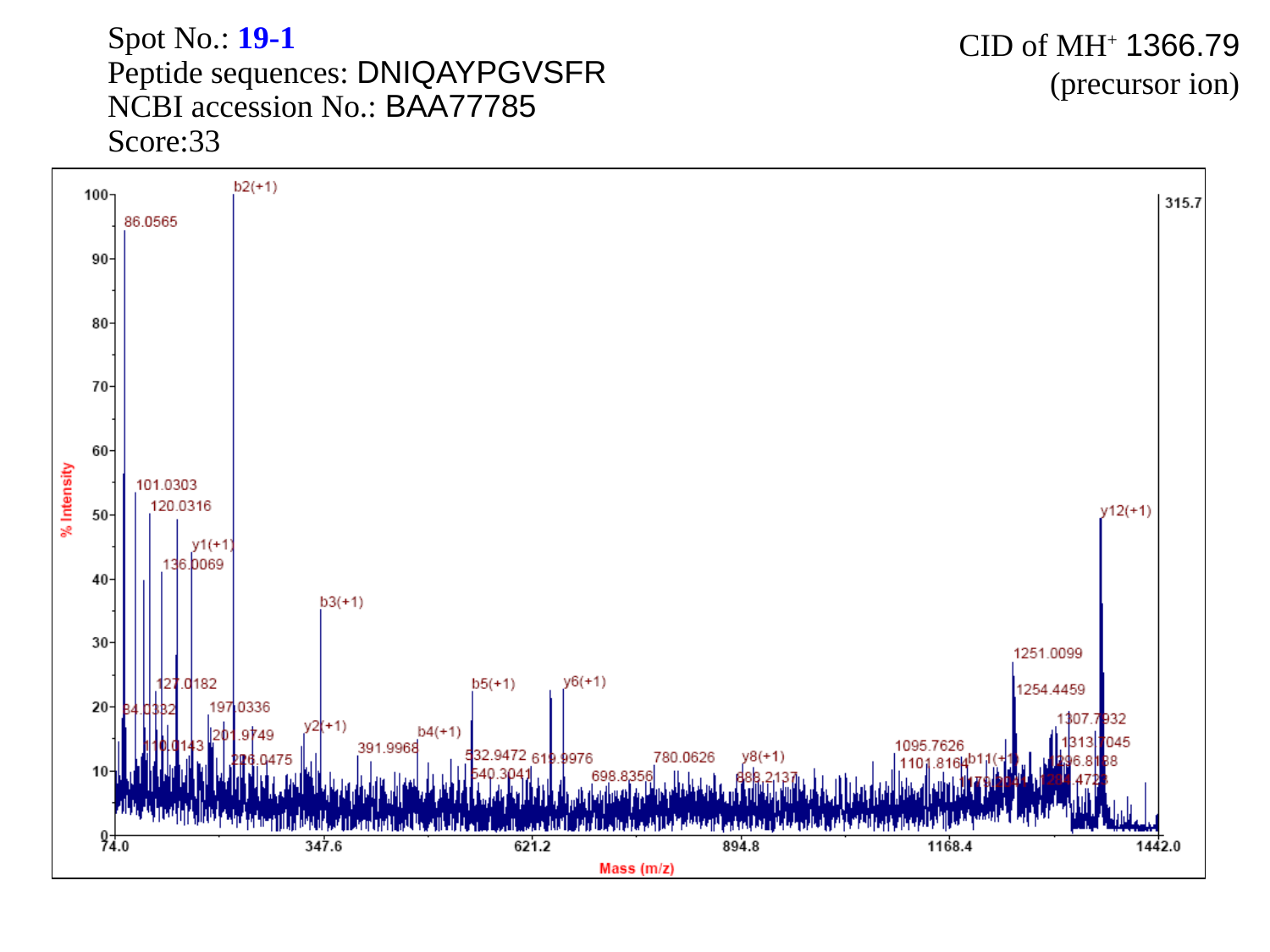

Spot No.: 19-1
Peptide sequences: DNIQAYPGVSFR
NCBI accession No.: BAA77785
Score:33
CID of MH+ 1366.79 (precursor ion)

## Slide 22
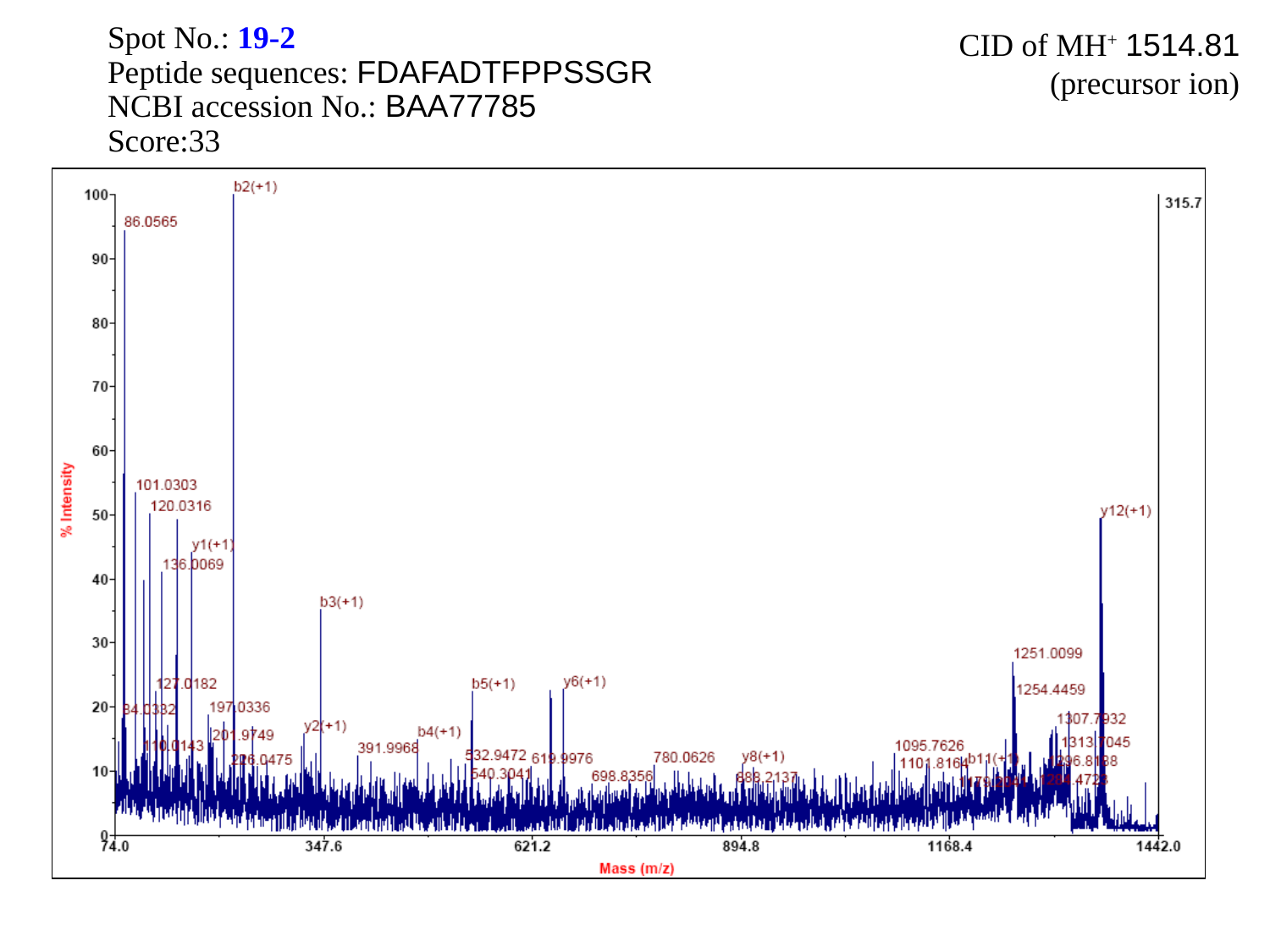

Spot No.: 19-2
Peptide sequences: FDAFADTFPPSSGR
NCBI accession No.: BAA77785
Score:33
CID of MH+ 1514.81 (precursor ion)

## Slide 23
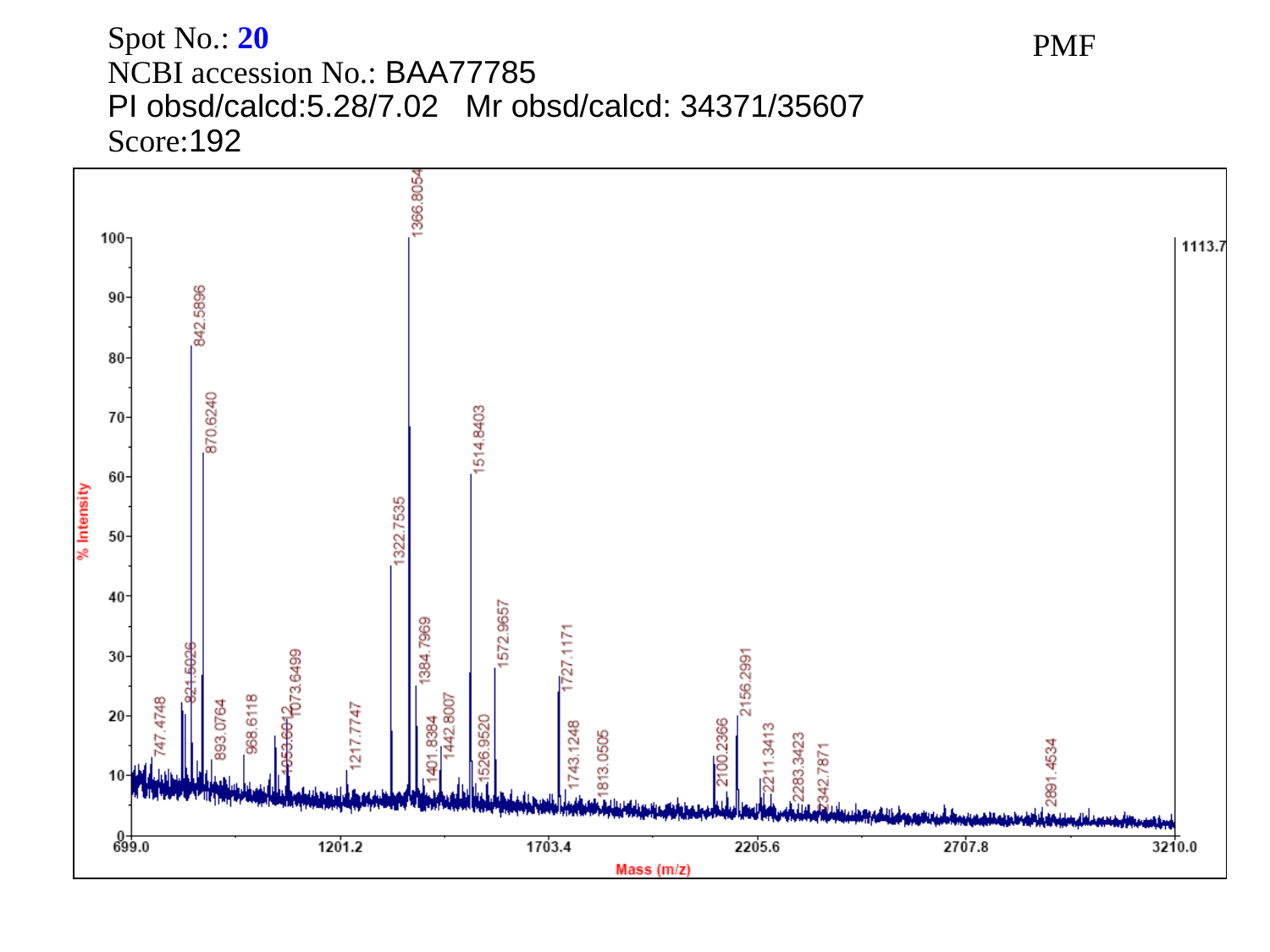

Spot No.: 20
NCBI accession No.: BAA77785
PI obsd/calcd:5.28/7.02 Mr obsd/calcd: 34371/35607
Score:192
PMF

## Slide 24
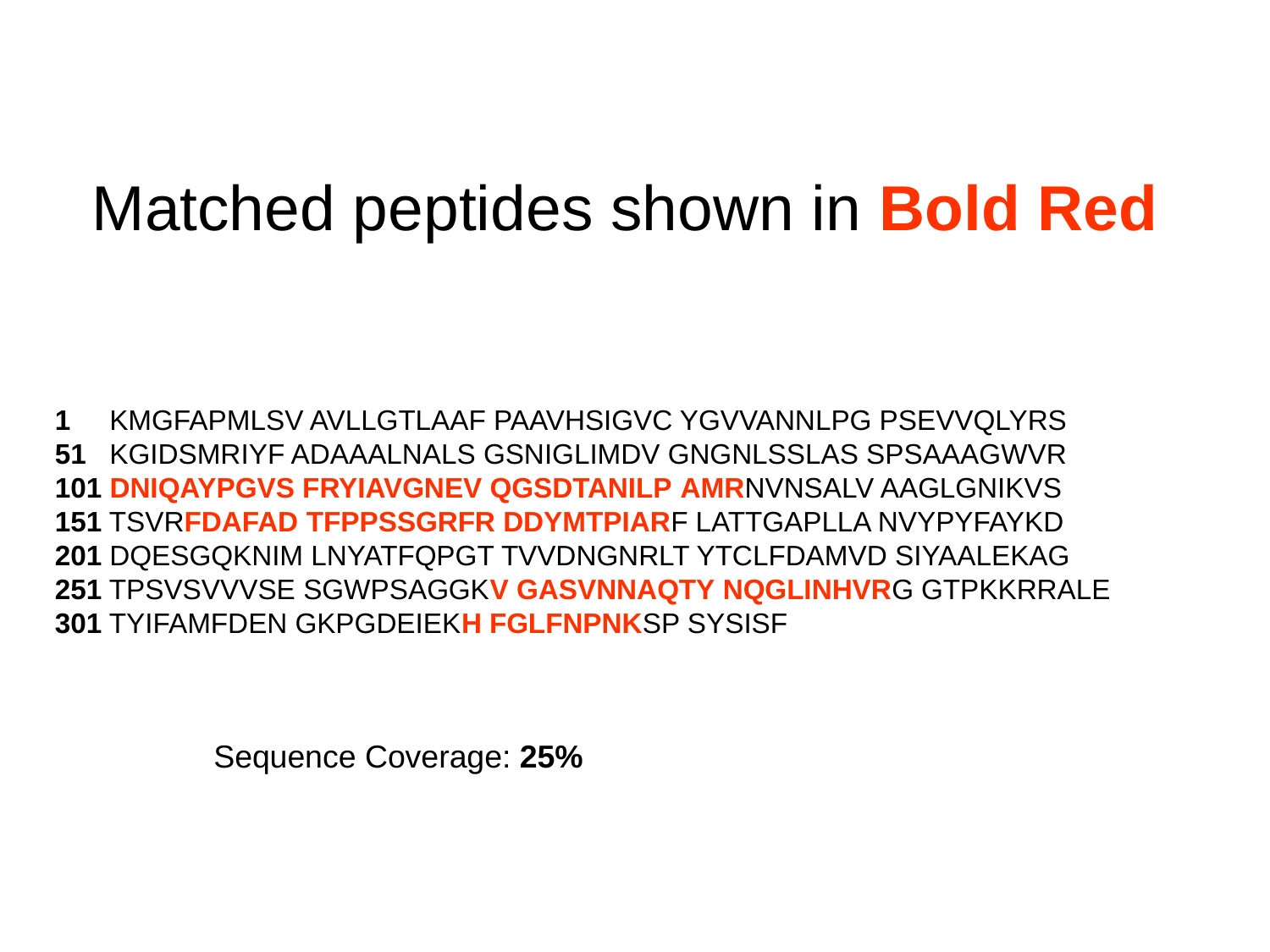

#
Matched peptides shown in Bold Red
1 KMGFAPMLSV AVLLGTLAAF PAAVHSIGVC YGVVANNLPG PSEVVQLYRS
51 KGIDSMRIYF ADAAALNALS GSNIGLIMDV GNGNLSSLAS SPSAAAGWVR
101 DNIQAYPGVS FRYIAVGNEV QGSDTANILP AMRNVNSALV AAGLGNIKVS
151 TSVRFDAFAD TFPPSSGRFR DDYMTPIARF LATTGAPLLA NVYPYFAYKD
201 DQESGQKNIM LNYATFQPGT TVVDNGNRLT YTCLFDAMVD SIYAALEKAG
251 TPSVSVVVSE SGWPSAGGKV GASVNNAQTY NQGLINHVRG GTPKKRRALE
301 TYIFAMFDEN GKPGDEIEKH FGLFNPNKSP SYSISF
Sequence Coverage: 25%

## Slide 25
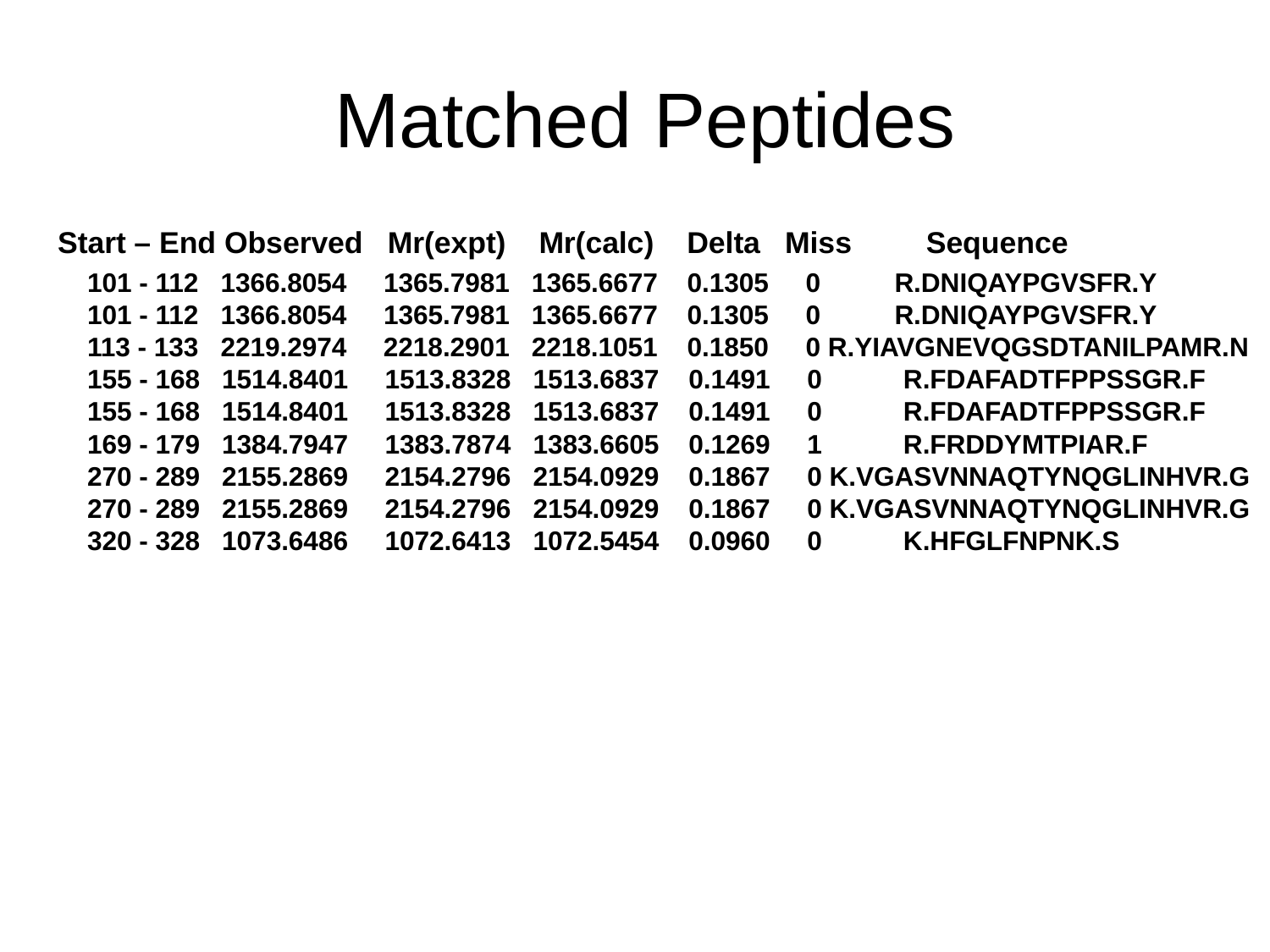

# Matched Peptides
Start – End Observed Mr(expt) Mr(calc) Delta Miss Sequence
 101 - 112 1366.8054 1365.7981 1365.6677 0.1305 0 R.DNIQAYPGVSFR.Y
 101 - 112 1366.8054 1365.7981 1365.6677 0.1305 0 R.DNIQAYPGVSFR.Y
 113 - 133 2219.2974 2218.2901 2218.1051 0.1850 0 R.YIAVGNEVQGSDTANILPAMR.N
 155 - 168 1514.8401 1513.8328 1513.6837 0.1491 0 R.FDAFADTFPPSSGR.F
 155 - 168 1514.8401 1513.8328 1513.6837 0.1491 0 R.FDAFADTFPPSSGR.F
 169 - 179 1384.7947 1383.7874 1383.6605 0.1269 1 R.FRDDYMTPIAR.F
 270 - 289 2155.2869 2154.2796 2154.0929 0.1867 0 K.VGASVNNAQTYNQGLINHVR.G
 270 - 289 2155.2869 2154.2796 2154.0929 0.1867 0 K.VGASVNNAQTYNQGLINHVR.G
 320 - 328 1073.6486 1072.6413 1072.5454 0.0960 0 K.HFGLFNPNK.S

## Slide 26
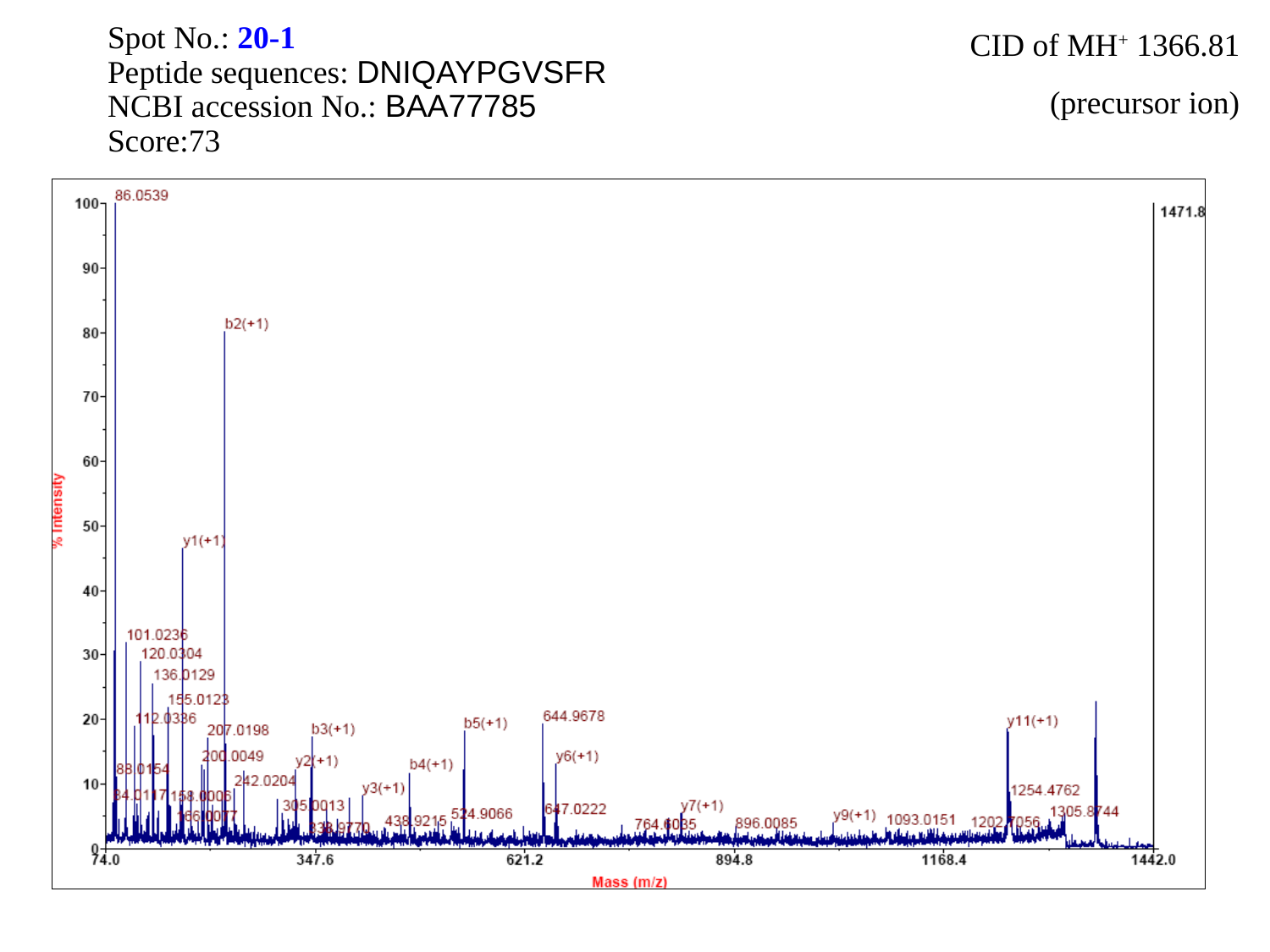

Spot No.: 20-1
Peptide sequences: DNIQAYPGVSFR
NCBI accession No.: BAA77785
Score:73
CID of MH+ 1366.81
(precursor ion)

## Slide 27
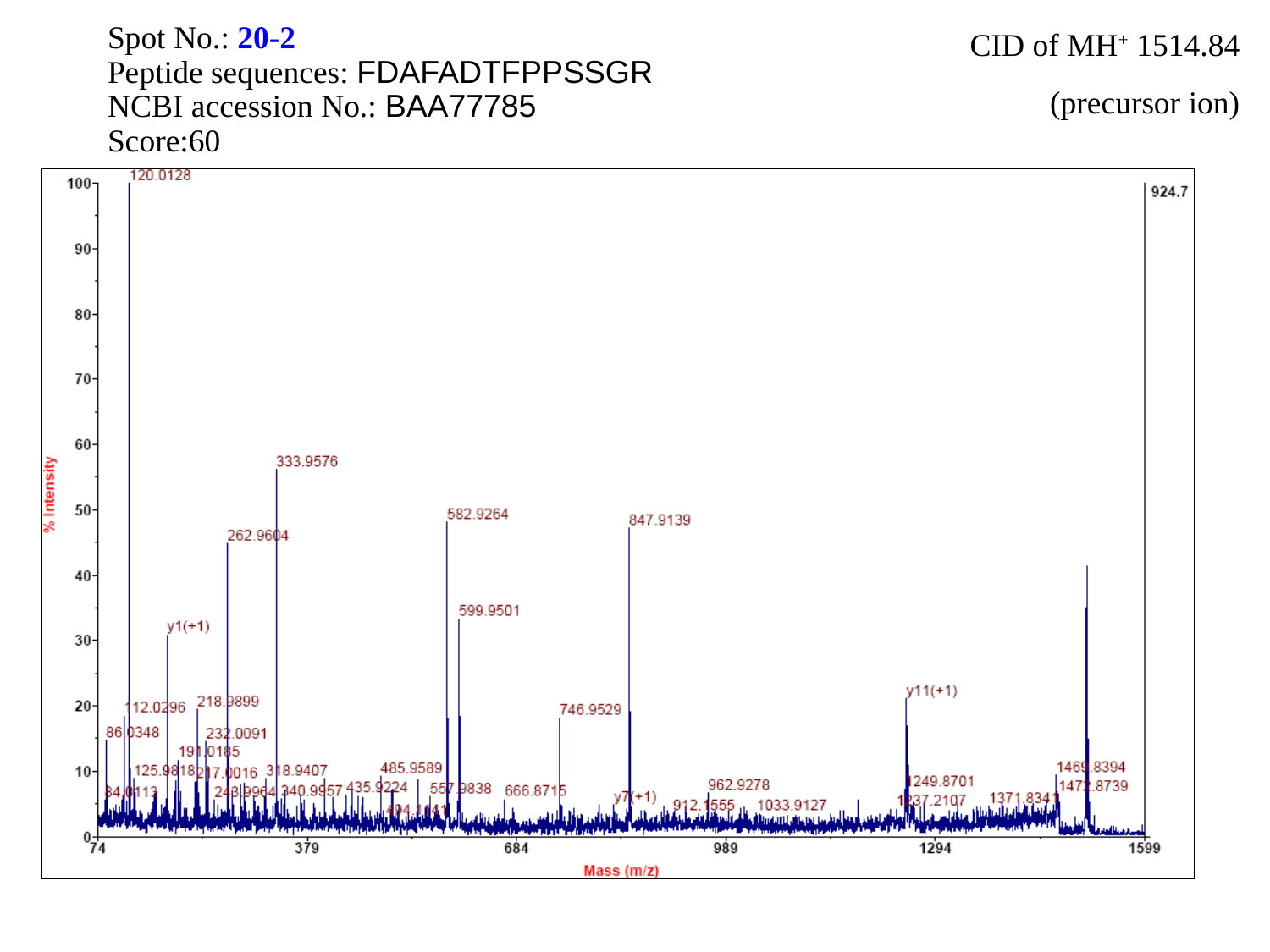

Spot No.: 20-2
Peptide sequences: FDAFADTFPPSSGR
NCBI accession No.: BAA77785
Score:60
CID of MH+ 1514.84
(precursor ion)

## Slide 28
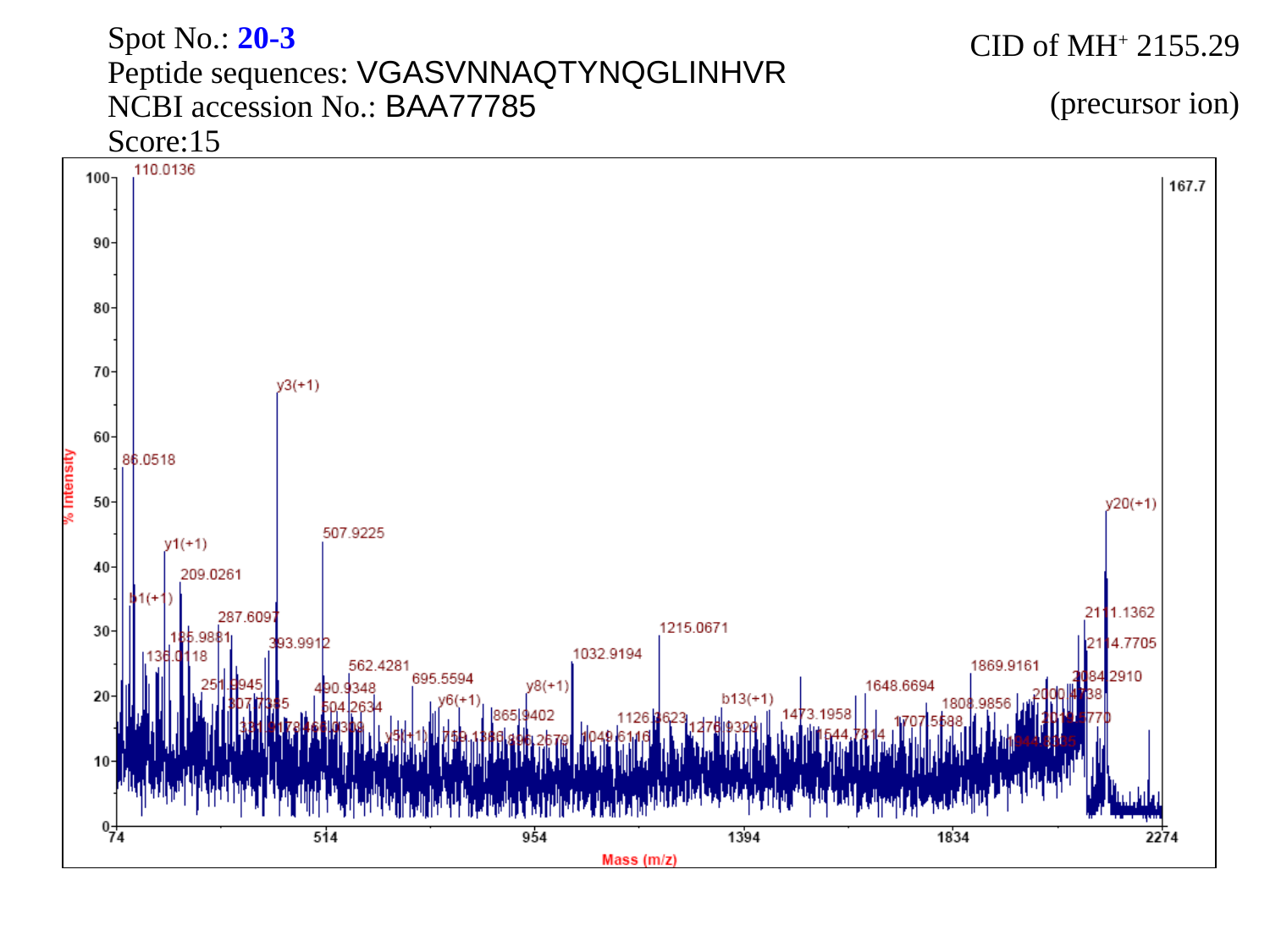

Spot No.: 20-3
Peptide sequences: VGASVNNAQTYNQGLINHVR
NCBI accession No.: BAA77785
Score:15
CID of MH+ 2155.29
(precursor ion)

## Slide 29
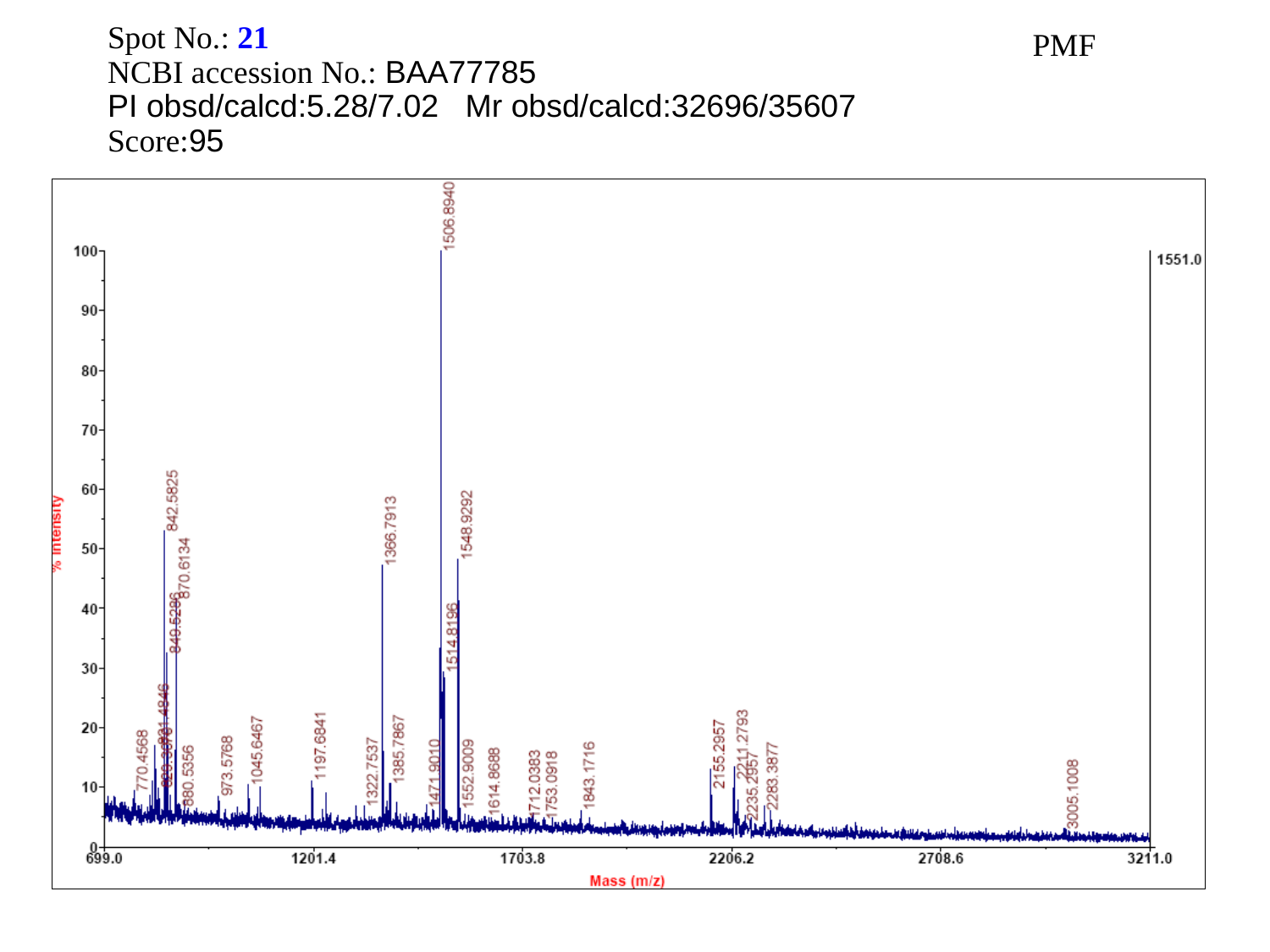

Spot No.: 21
NCBI accession No.: BAA77785
PI obsd/calcd:5.28/7.02 Mr obsd/calcd:32696/35607
Score:95
PMF

## Slide 30
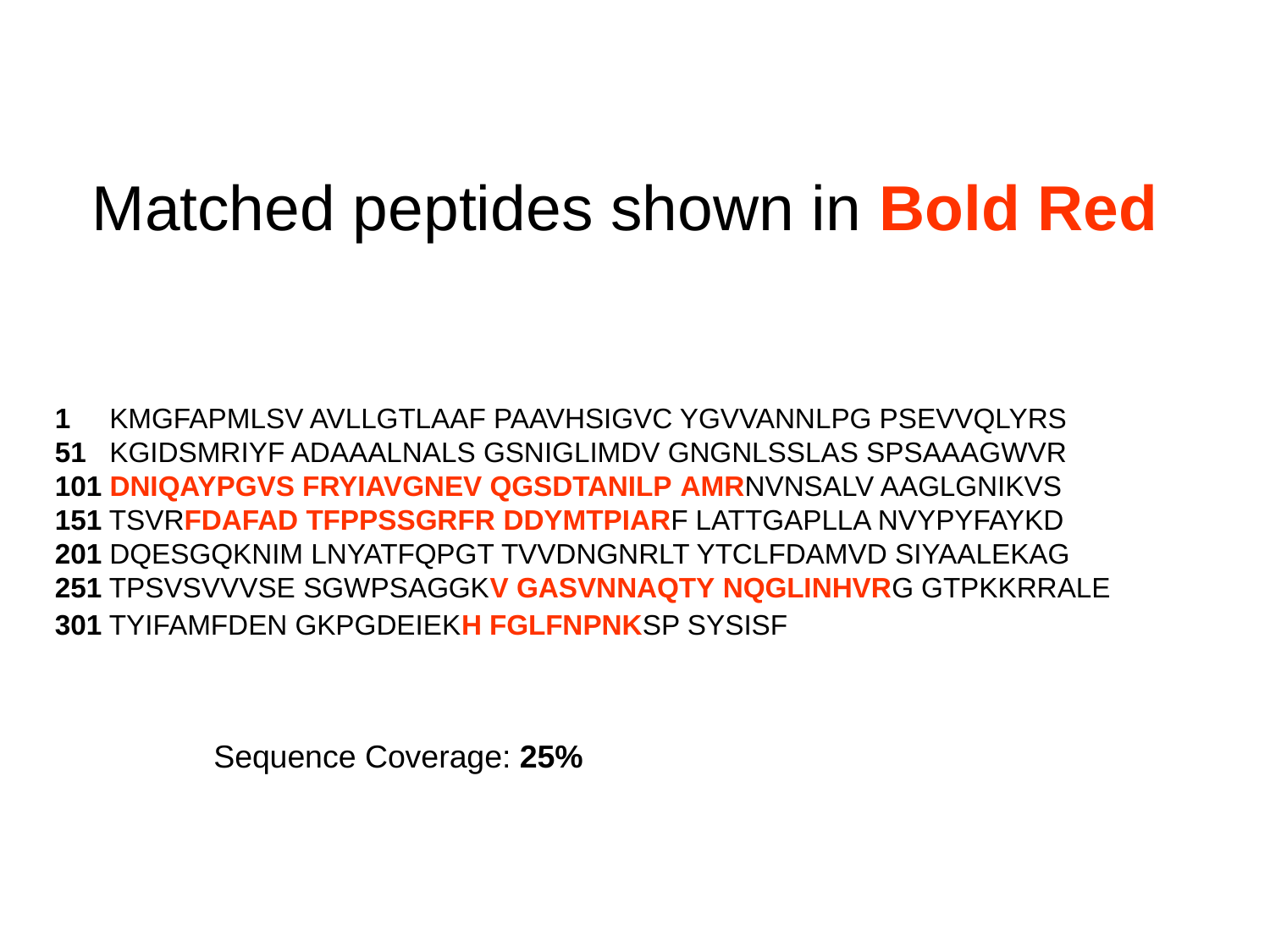

#
Matched peptides shown in Bold Red
1 KMGFAPMLSV AVLLGTLAAF PAAVHSIGVC YGVVANNLPG PSEVVQLYRS
51 KGIDSMRIYF ADAAALNALS GSNIGLIMDV GNGNLSSLAS SPSAAAGWVR
101 DNIQAYPGVS FRYIAVGNEV QGSDTANILP AMRNVNSALV AAGLGNIKVS
151 TSVRFDAFAD TFPPSSGRFR DDYMTPIARF LATTGAPLLA NVYPYFAYKD
201 DQESGQKNIM LNYATFQPGT TVVDNGNRLT YTCLFDAMVD SIYAALEKAG
251 TPSVSVVVSE SGWPSAGGKV GASVNNAQTY NQGLINHVRG GTPKKRRALE
301 TYIFAMFDEN GKPGDEIEKH FGLFNPNKSP SYSISF
Sequence Coverage: 25%

## Slide 31
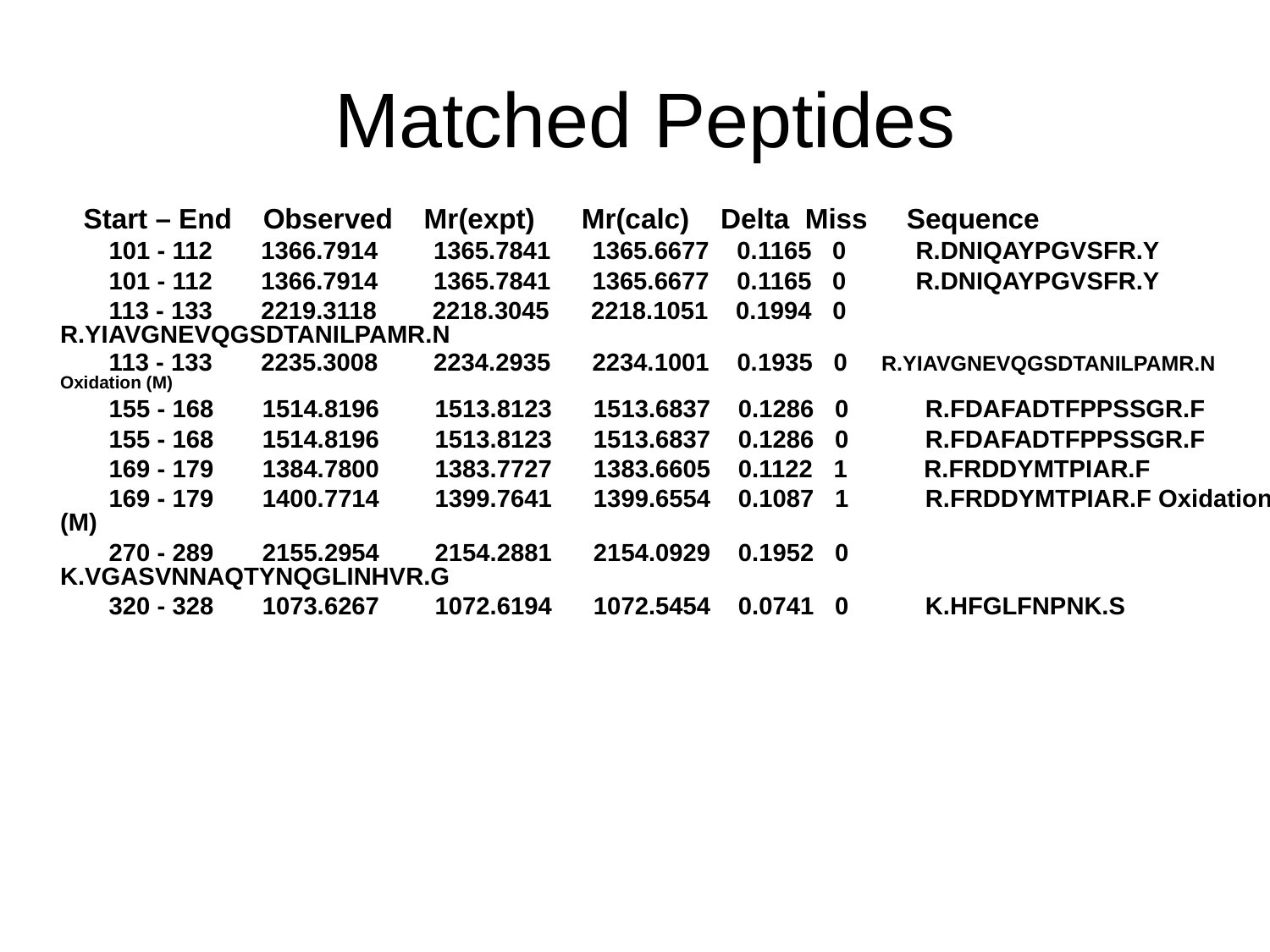

# Matched Peptides
 Start – End Observed Mr(expt) Mr(calc) Delta Miss Sequence
 101 - 112 1366.7914 1365.7841 1365.6677 0.1165 0 R.DNIQAYPGVSFR.Y
 101 - 112 1366.7914 1365.7841 1365.6677 0.1165 0 R.DNIQAYPGVSFR.Y
 113 - 133 2219.3118 2218.3045 2218.1051 0.1994 0 R.YIAVGNEVQGSDTANILPAMR.N
 113 - 133 2235.3008 2234.2935 2234.1001 0.1935 0 R.YIAVGNEVQGSDTANILPAMR.N Oxidation (M)
 155 - 168 1514.8196 1513.8123 1513.6837 0.1286 0 R.FDAFADTFPPSSGR.F
 155 - 168 1514.8196 1513.8123 1513.6837 0.1286 0 R.FDAFADTFPPSSGR.F
 169 - 179 1384.7800 1383.7727 1383.6605 0.1122 1 R.FRDDYMTPIAR.F
 169 - 179 1400.7714 1399.7641 1399.6554 0.1087 1 R.FRDDYMTPIAR.F Oxidation (M)
 270 - 289 2155.2954 2154.2881 2154.0929 0.1952 0 K.VGASVNNAQTYNQGLINHVR.G
 320 - 328 1073.6267 1072.6194 1072.5454 0.0741 0 K.HFGLFNPNK.S

## Slide 32
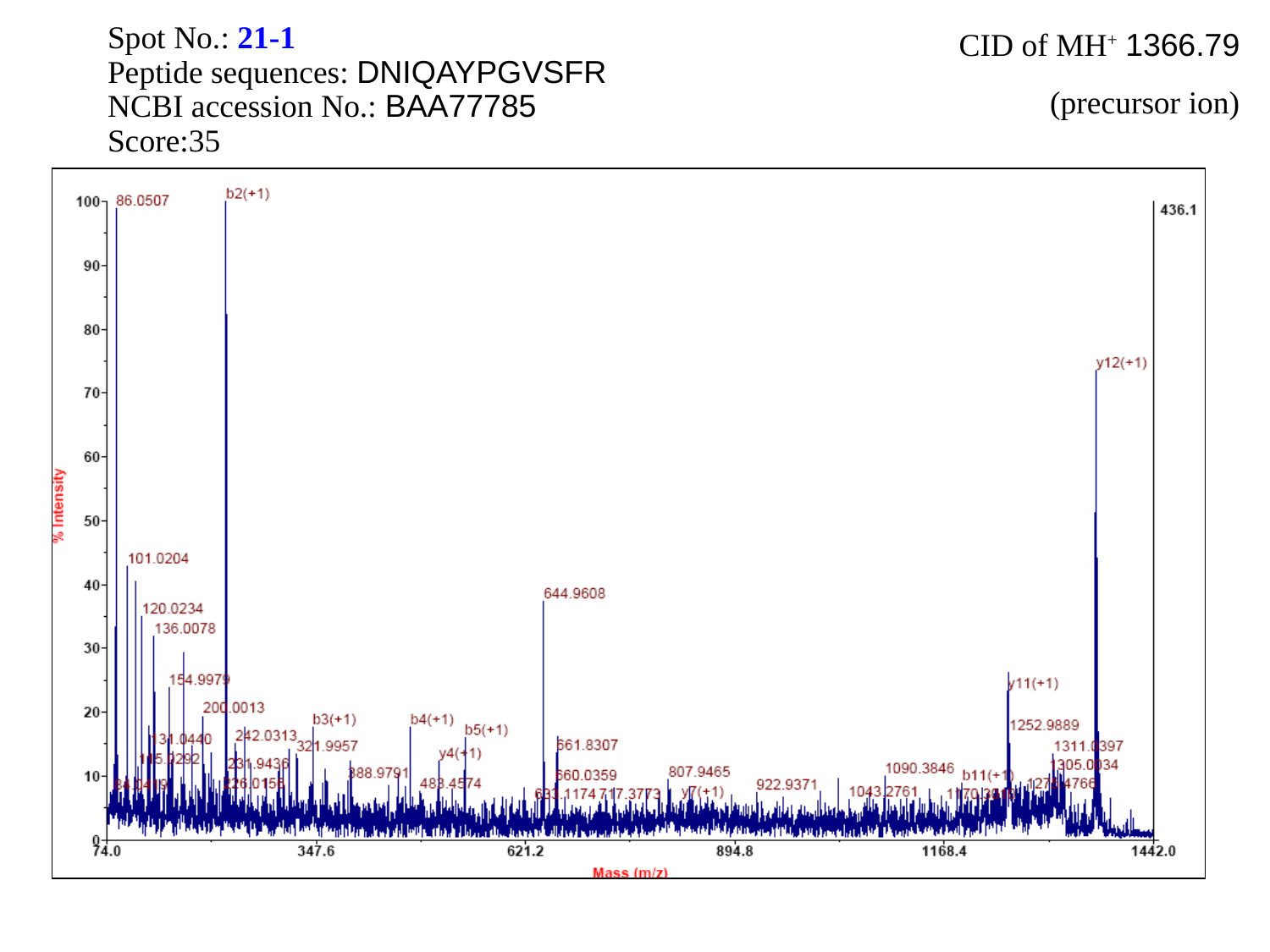

Spot No.: 21-1
Peptide sequences: DNIQAYPGVSFR
NCBI accession No.: BAA77785
Score:35
CID of MH+ 1366.79
(precursor ion)

## Slide 33
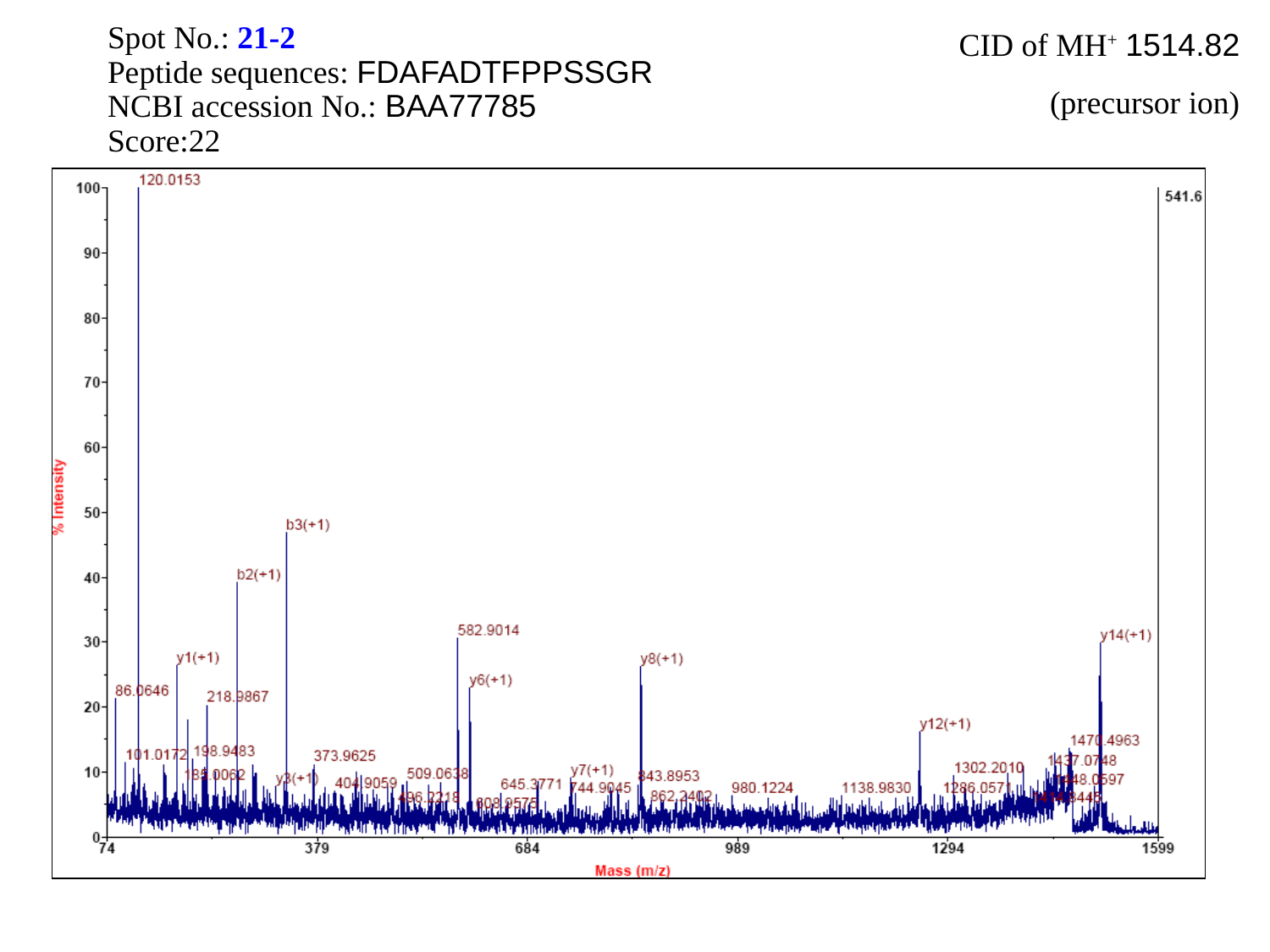

Spot No.: 21-2
Peptide sequences: FDAFADTFPPSSGR
NCBI accession No.: BAA77785
Score:22
CID of MH+ 1514.82
(precursor ion)

## Slide 34
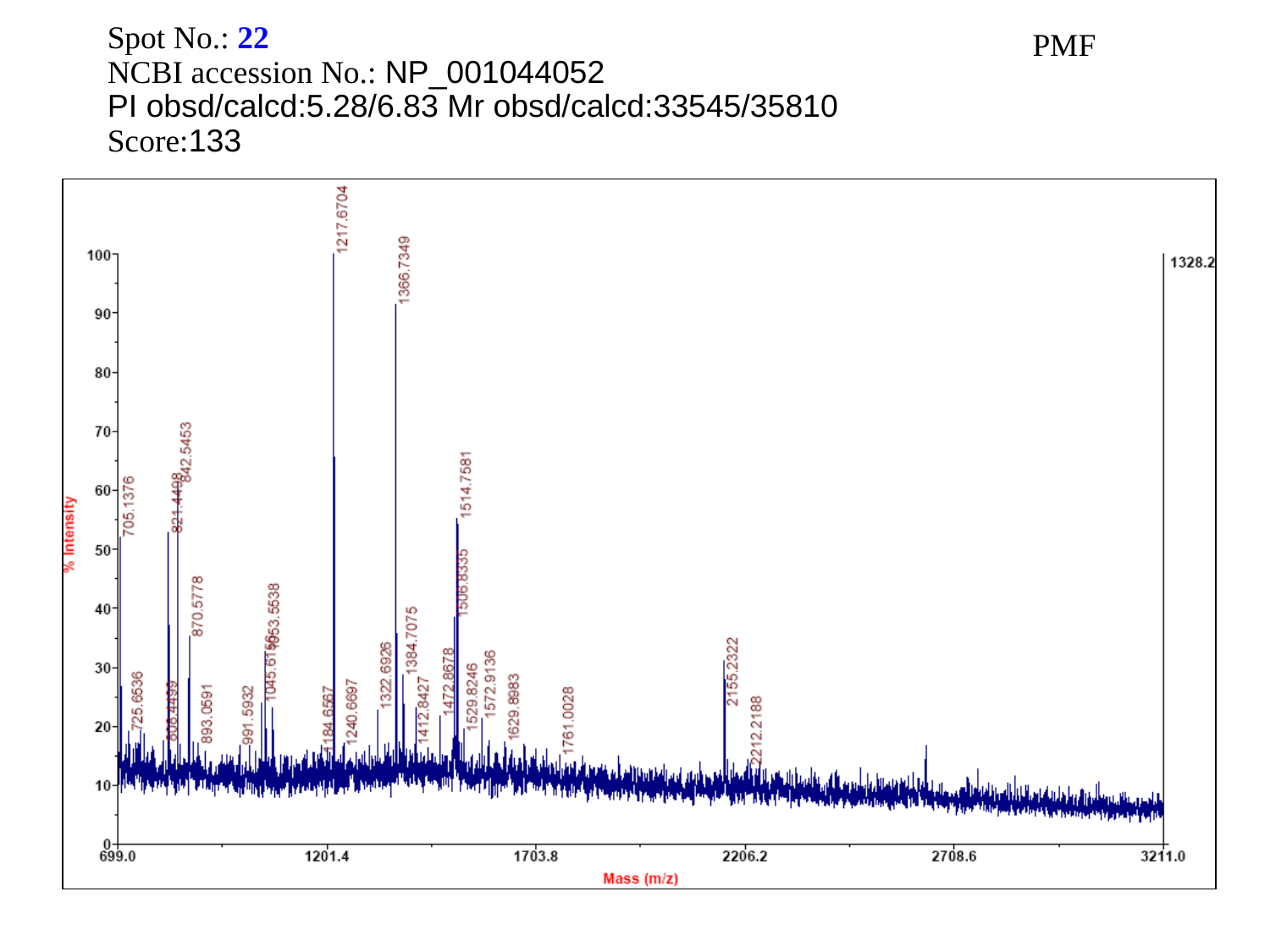

Spot No.: 22
NCBI accession No.: NP_001044052
PI obsd/calcd:5.28/6.83 Mr obsd/calcd:33545/35810
Score:133
PMF

## Slide 35
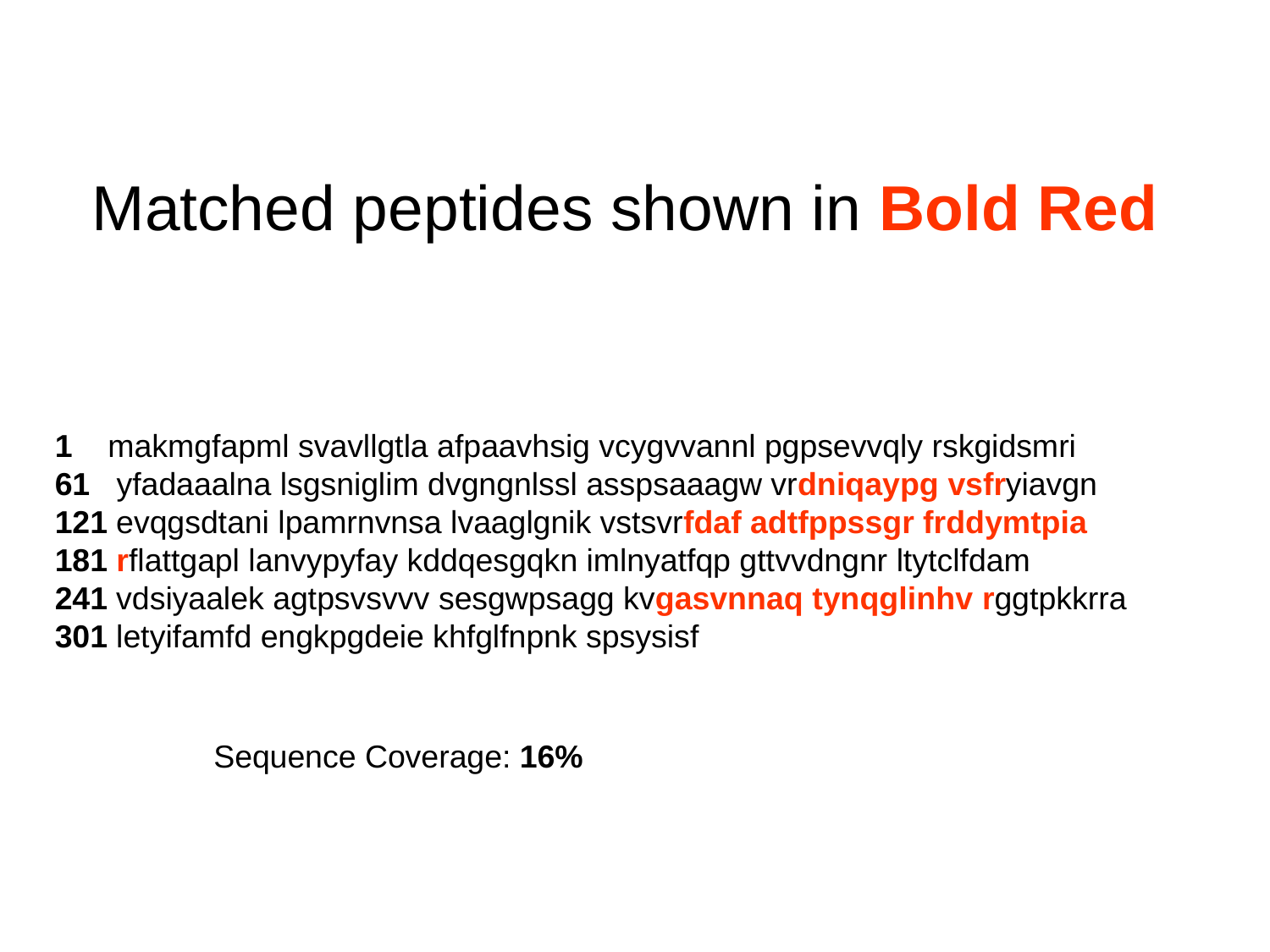

#
Matched peptides shown in Bold Red
1 makmgfapml svavllgtla afpaavhsig vcygvvannl pgpsevvqly rskgidsmri
61 yfadaaalna lsgsniglim dvgngnlssl asspsaaagw vrdniqaypg vsfryiavgn
121 evqgsdtani lpamrnvnsa lvaaglgnik vstsvrfdaf adtfppssgr frddymtpia
181 rflattgapl lanvypyfay kddqesgqkn imlnyatfqp gttvvdngnr ltytclfdam
241 vdsiyaalek agtpsvsvvv sesgwpsagg kvgasvnnaq tynqglinhv rggtpkkrra
301 letyifamfd engkpgdeie khfglfnpnk spsysisf
Sequence Coverage: 16%

## Slide 36
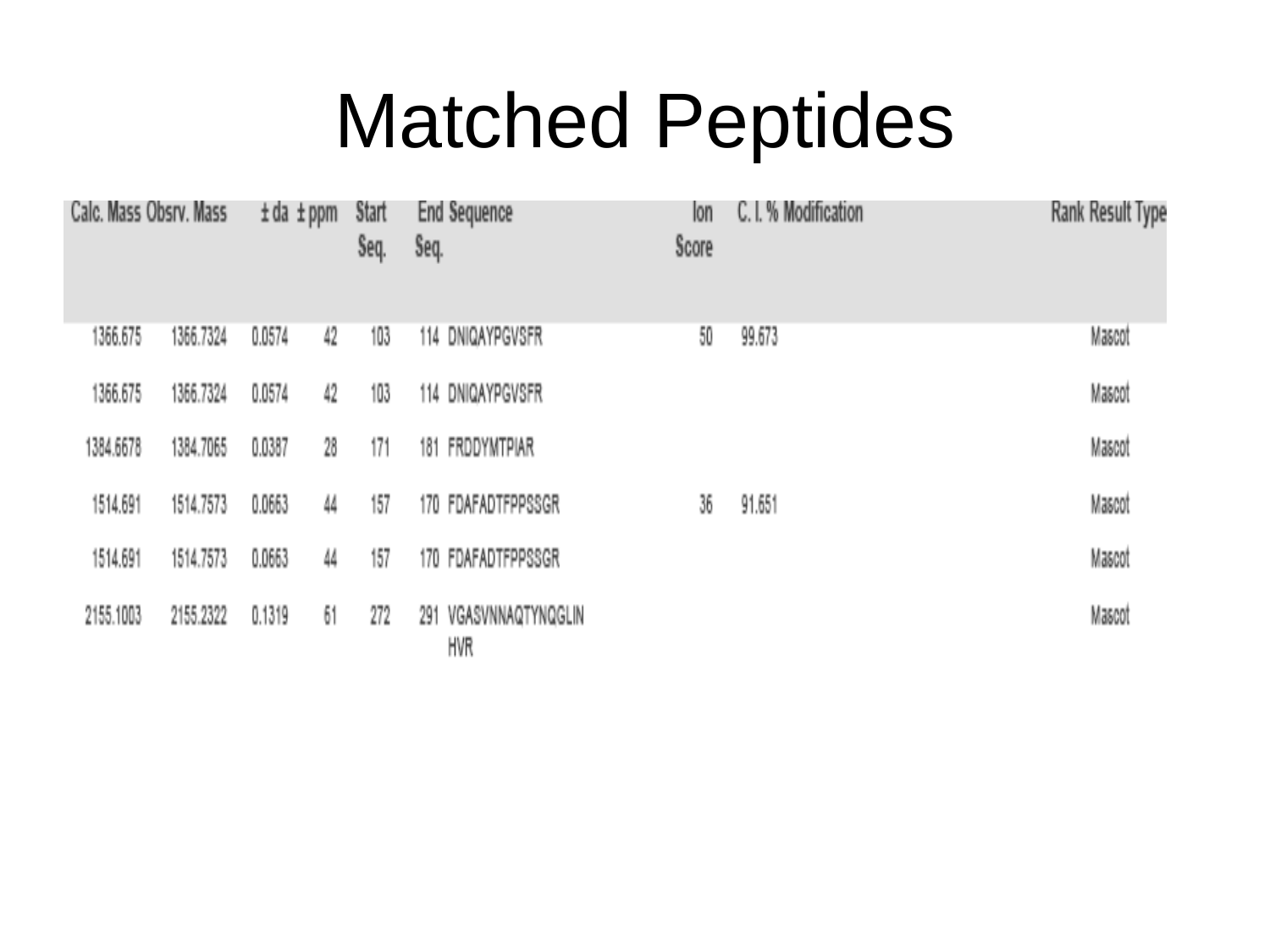

# Matched Peptides

## Slide 37
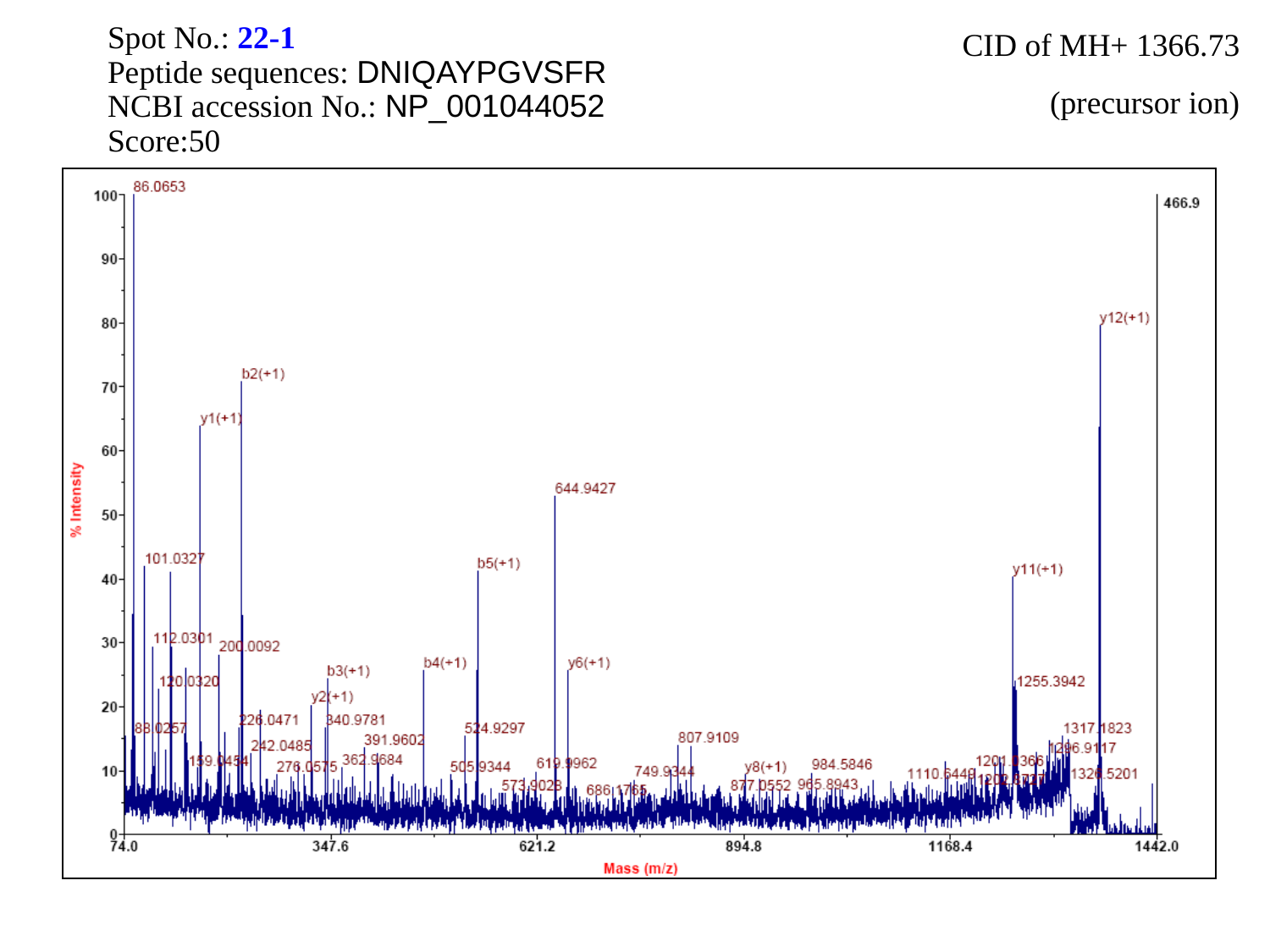

Spot No.: 22-1
Peptide sequences: DNIQAYPGVSFR
NCBI accession No.: NP_001044052
Score:50
CID of MH+ 1366.73
(precursor ion)

## Slide 38
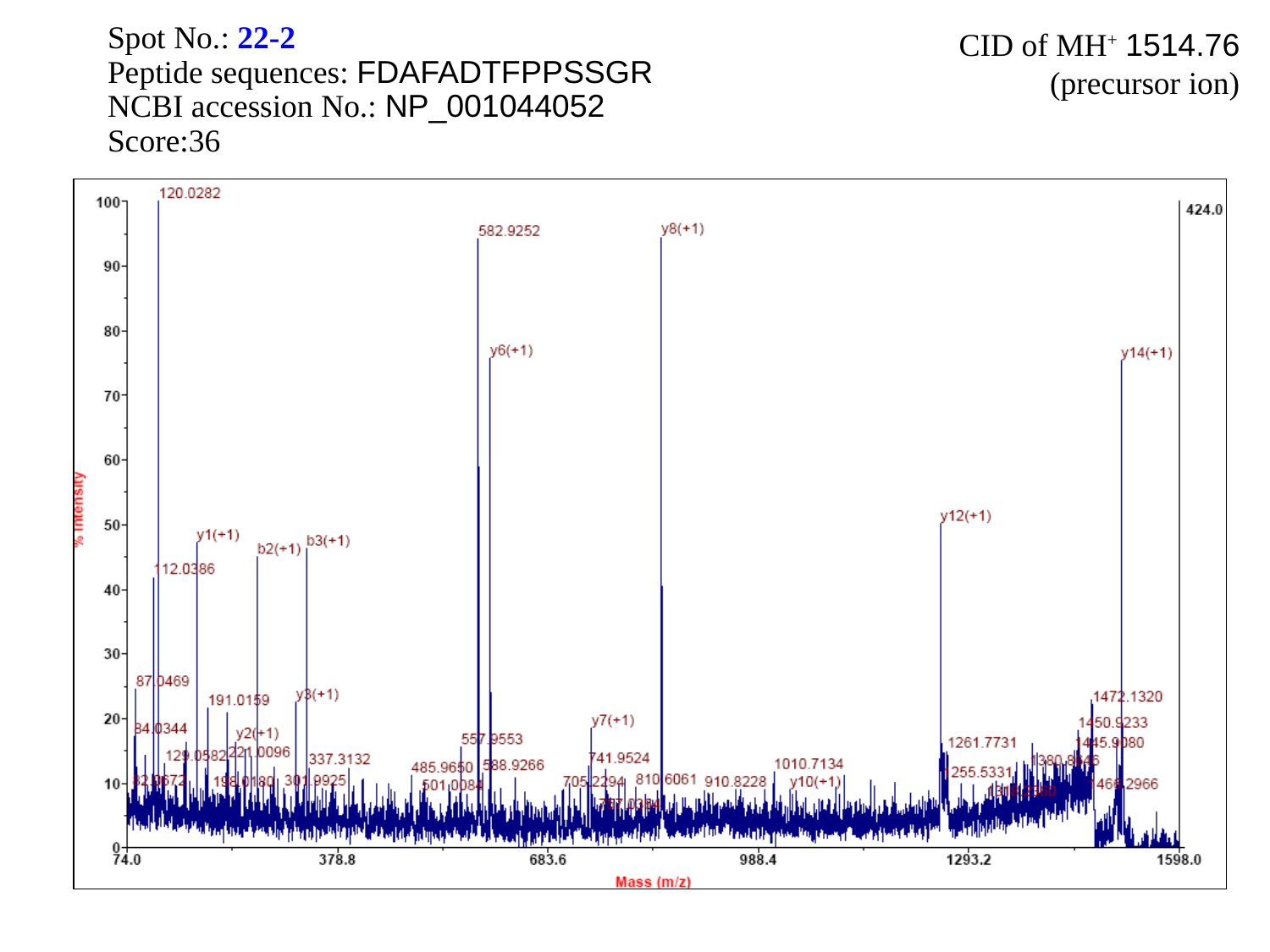

Spot No.: 22-2
Peptide sequences: FDAFADTFPPSSGR
NCBI accession No.: NP_001044052
Score:36
CID of MH+ 1514.76 (precursor ion)

## Slide 39
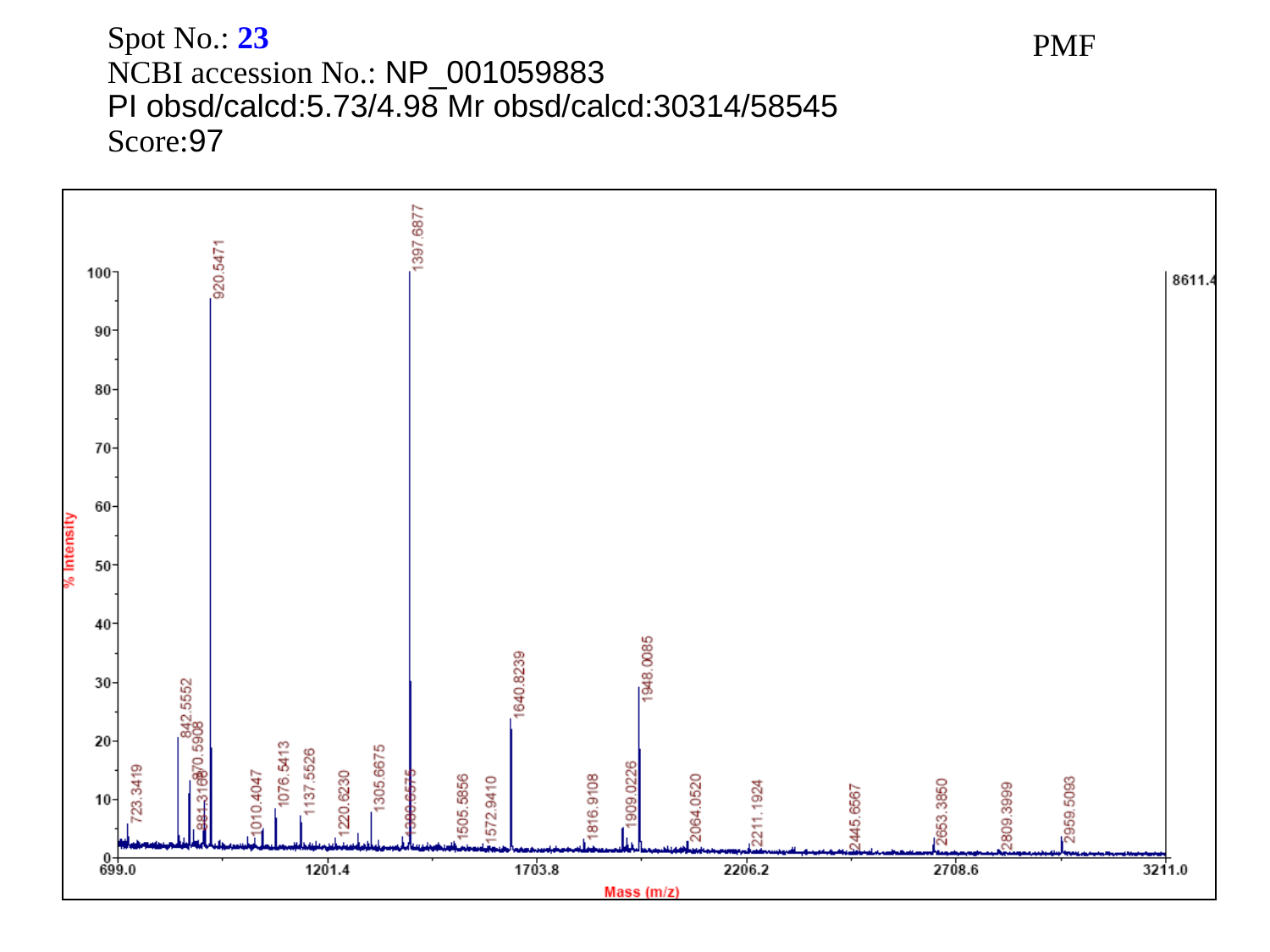

Spot No.: 23
NCBI accession No.: NP_001059883
PI obsd/calcd:5.73/4.98 Mr obsd/calcd:30314/58545
Score:97
PMF

## Slide 40
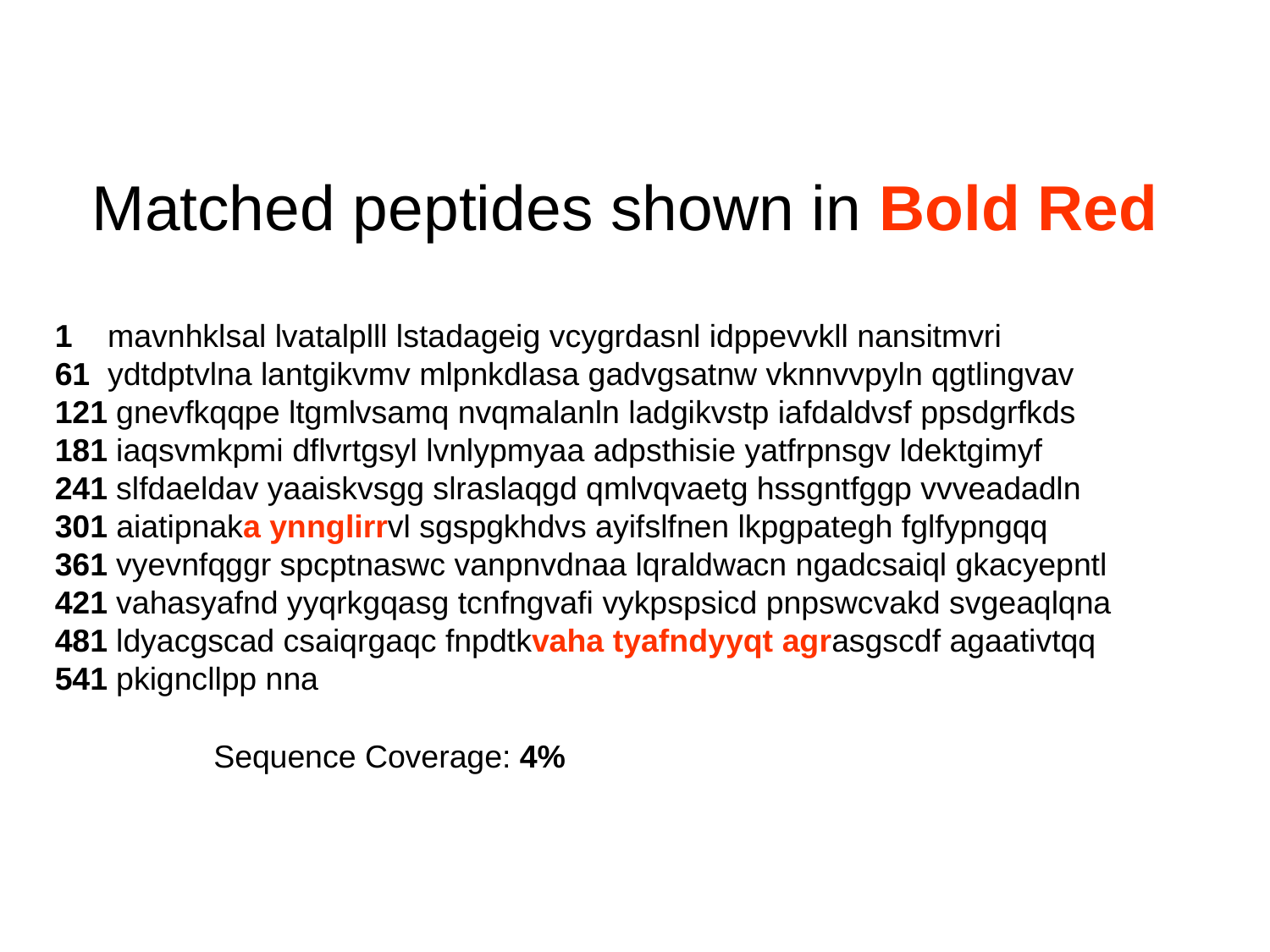

#
Matched peptides shown in Bold Red
1 mavnhklsal lvatalplll lstadageig vcygrdasnl idppevvkll nansitmvri
61 ydtdptvlna lantgikvmv mlpnkdlasa gadvgsatnw vknnvvpyln qgtlingvav
121 gnevfkqqpe ltgmlvsamq nvqmalanln ladgikvstp iafdaldvsf ppsdgrfkds
181 iaqsvmkpmi dflvrtgsyl lvnlypmyaa adpsthisie yatfrpnsgv ldektgimyf
241 slfdaeldav yaaiskvsgg slraslaqgd qmlvqvaetg hssgntfggp vvveadadln
301 aiatipnaka ynnglirrvl sgspgkhdvs ayifslfnen lkpgpategh fglfypngqq
361 vyevnfqggr spcptnaswc vanpnvdnaa lqraldwacn ngadcsaiql gkacyepntl
421 vahasyafnd yyqrkgqasg tcnfngvafi vykpspsicd pnpswcvakd svgeaqlqna
481 ldyacgscad csaiqrgaqc fnpdtkvaha tyafndyyqt agrasgscdf agaativtqq
541 pkigncllpp nna
Sequence Coverage: 4%

## Slide 41
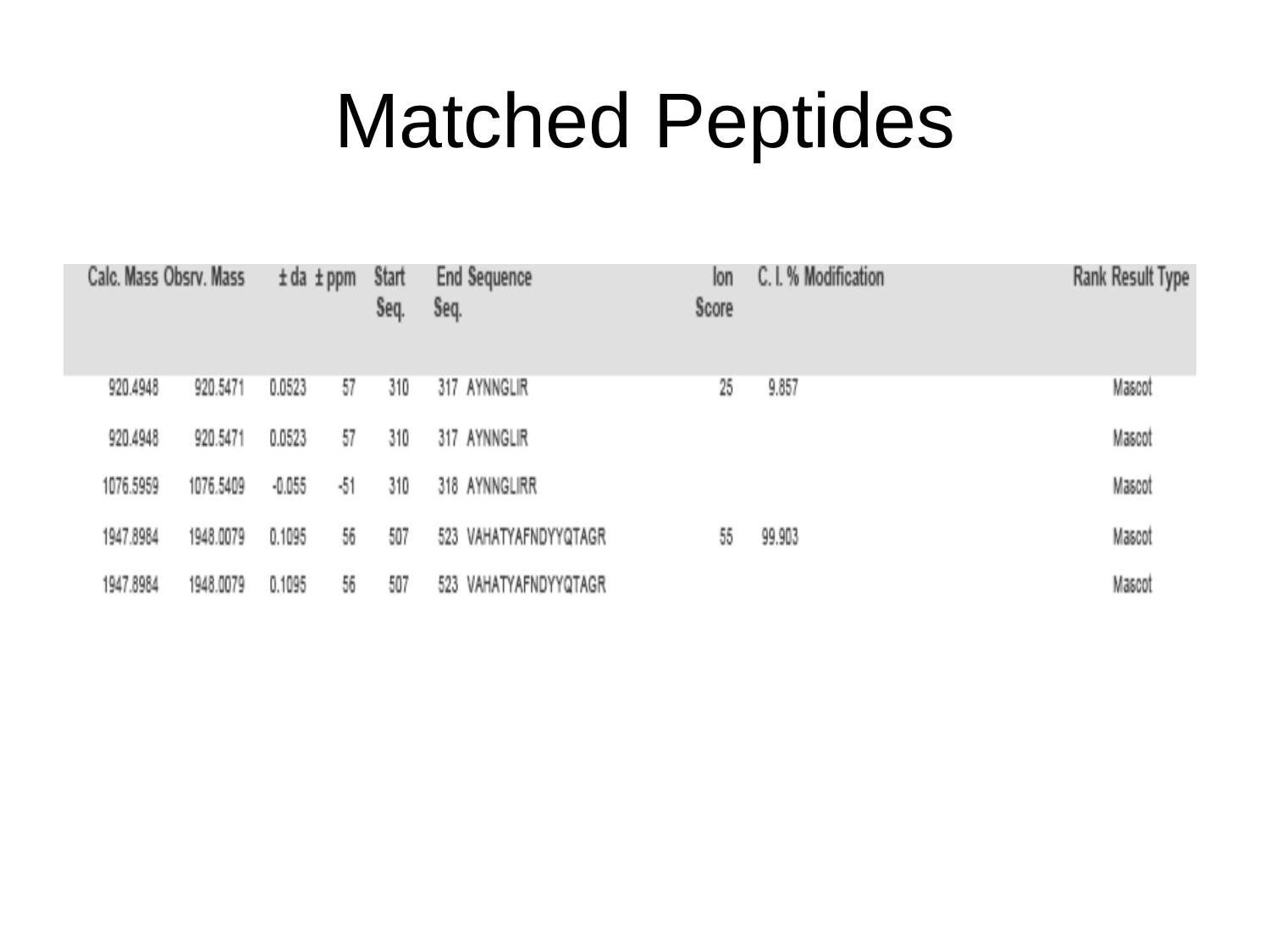

# Matched Peptides

## Slide 42
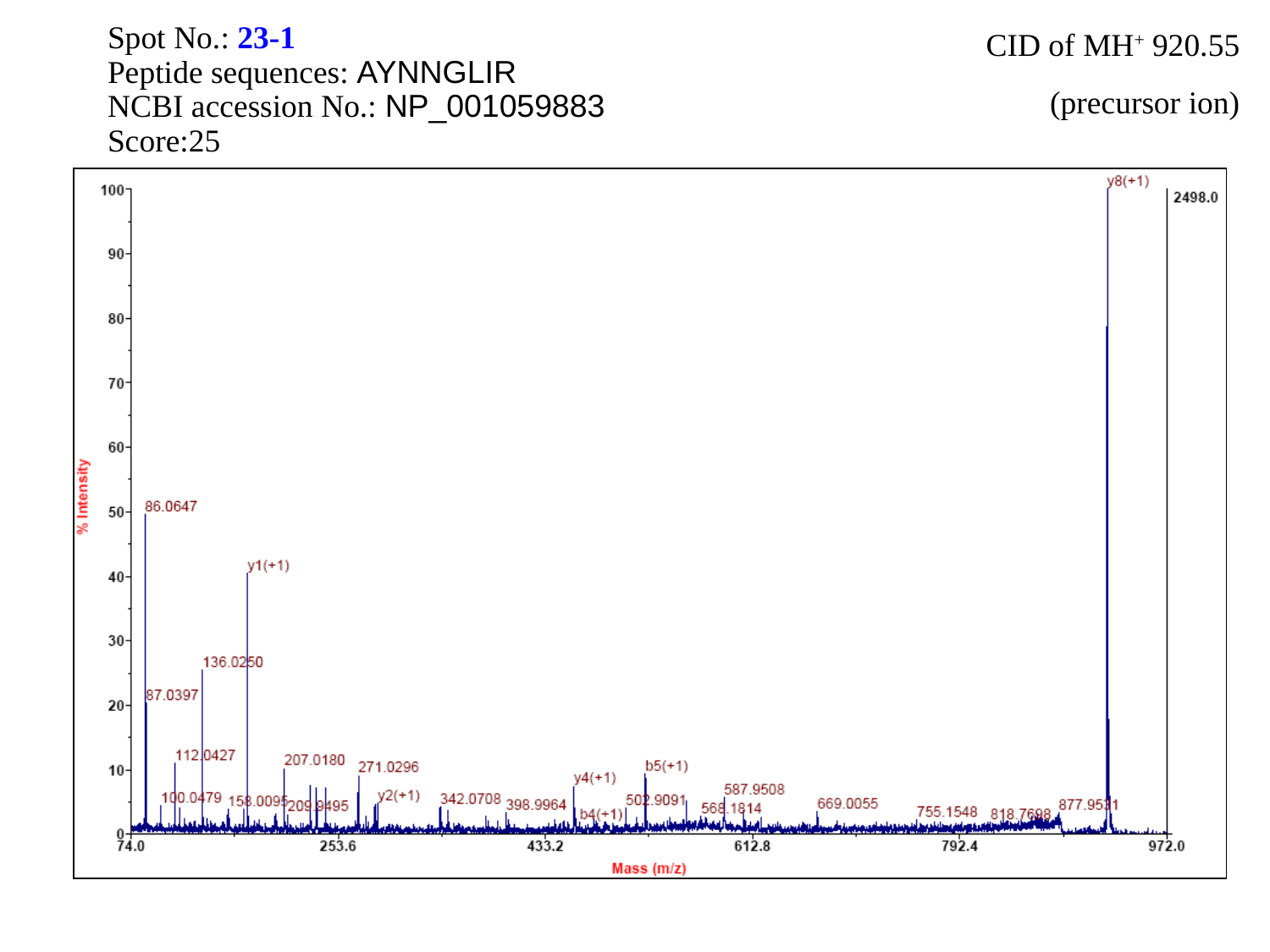

Spot No.: 23-1
Peptide sequences: AYNNGLIR
NCBI accession No.: NP_001059883
Score:25
CID of MH+ 920.55
(precursor ion)

## Slide 43
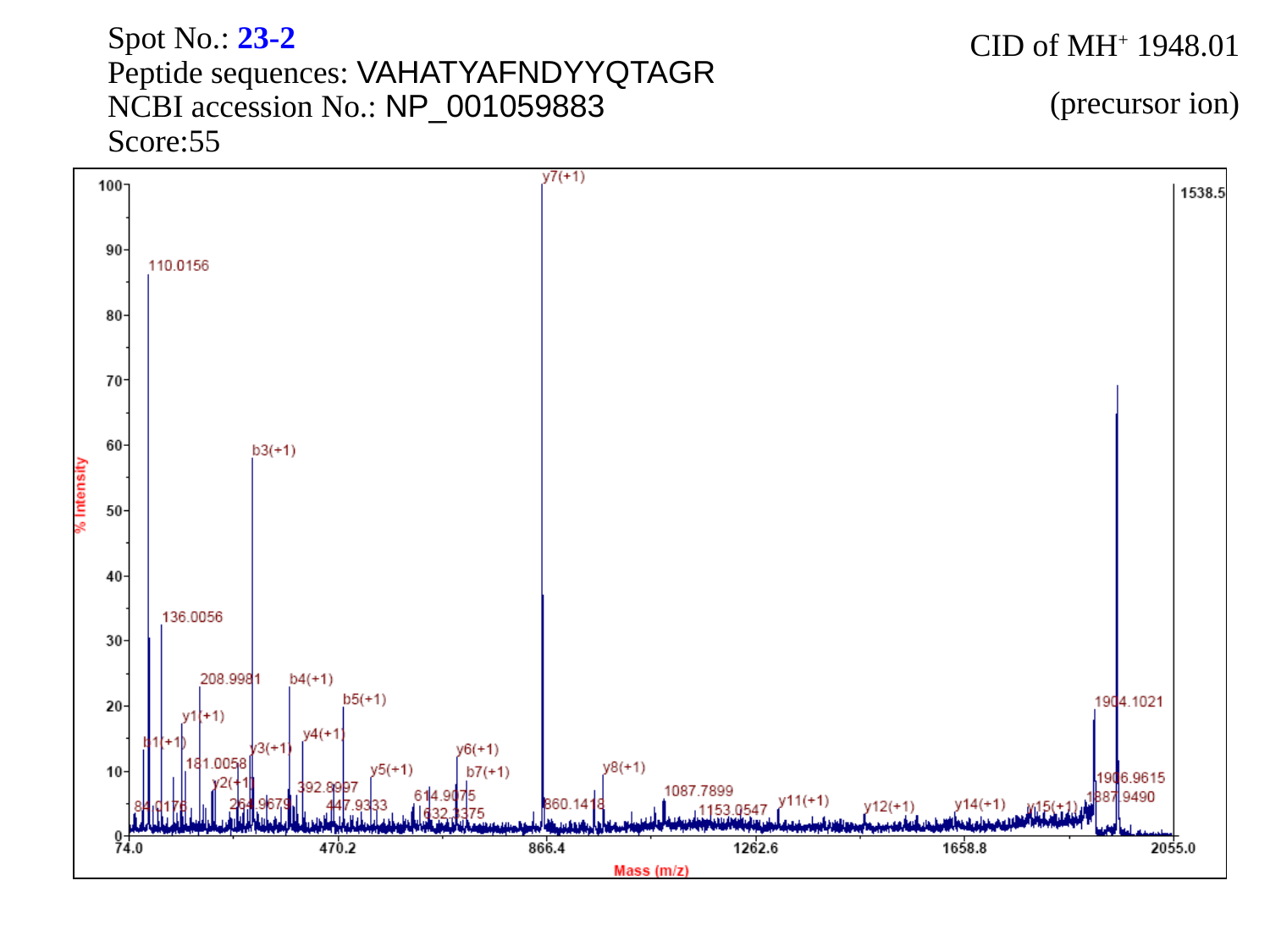

Spot No.: 23-2
Peptide sequences: VAHATYAFNDYYQTAGR
NCBI accession No.: NP_001059883
Score:55
CID of MH+ 1948.01
(precursor ion)

## Slide 44
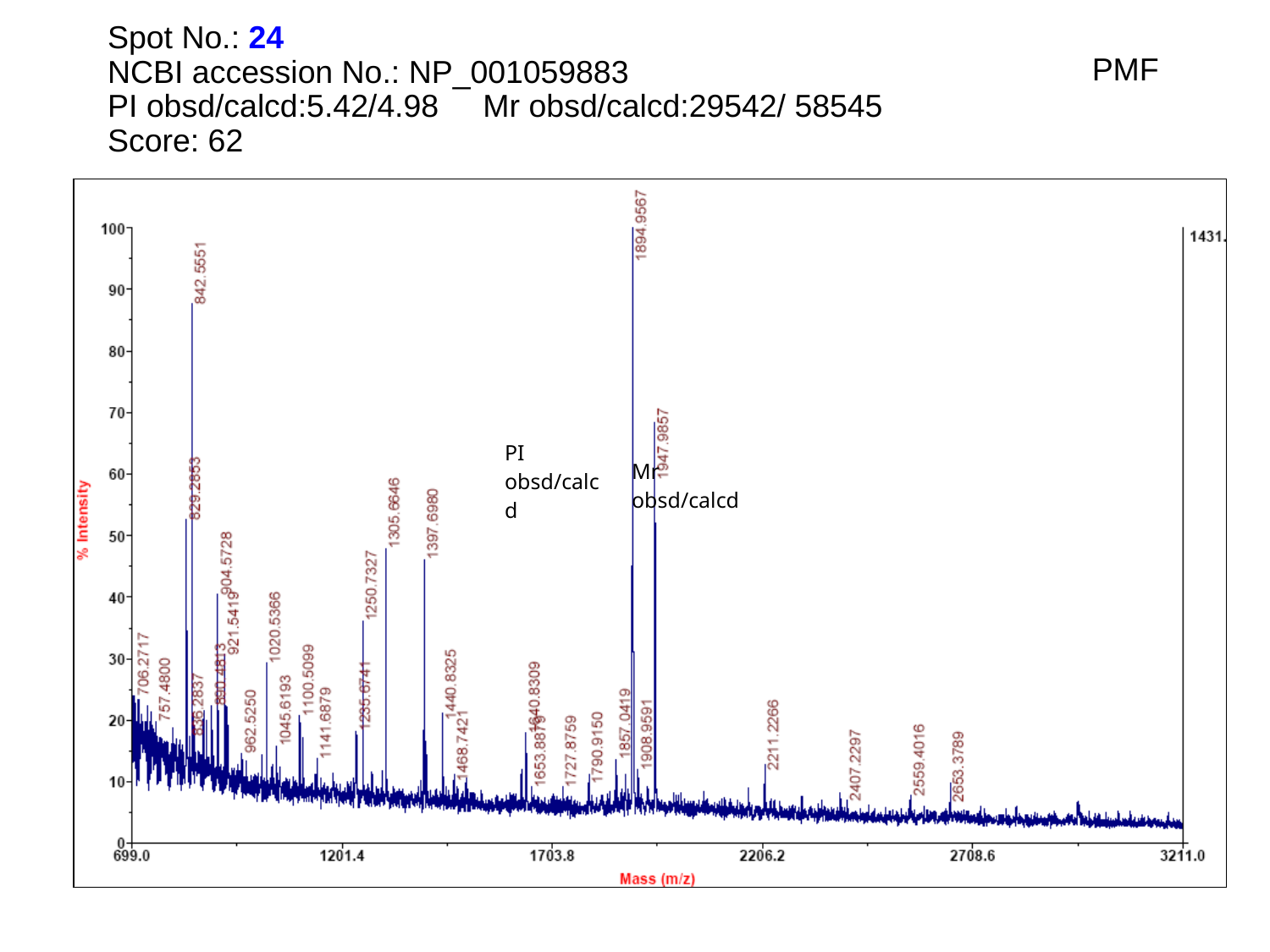

Spot No.: 24
NCBI accession No.: NP_001059883
PI obsd/calcd:5.42/4.98 Mr obsd/calcd:29542/ 58545
Score: 62
PMF
| PI obsd/calcd | Mr obsd/calcd |
| --- | --- |

## Slide 45
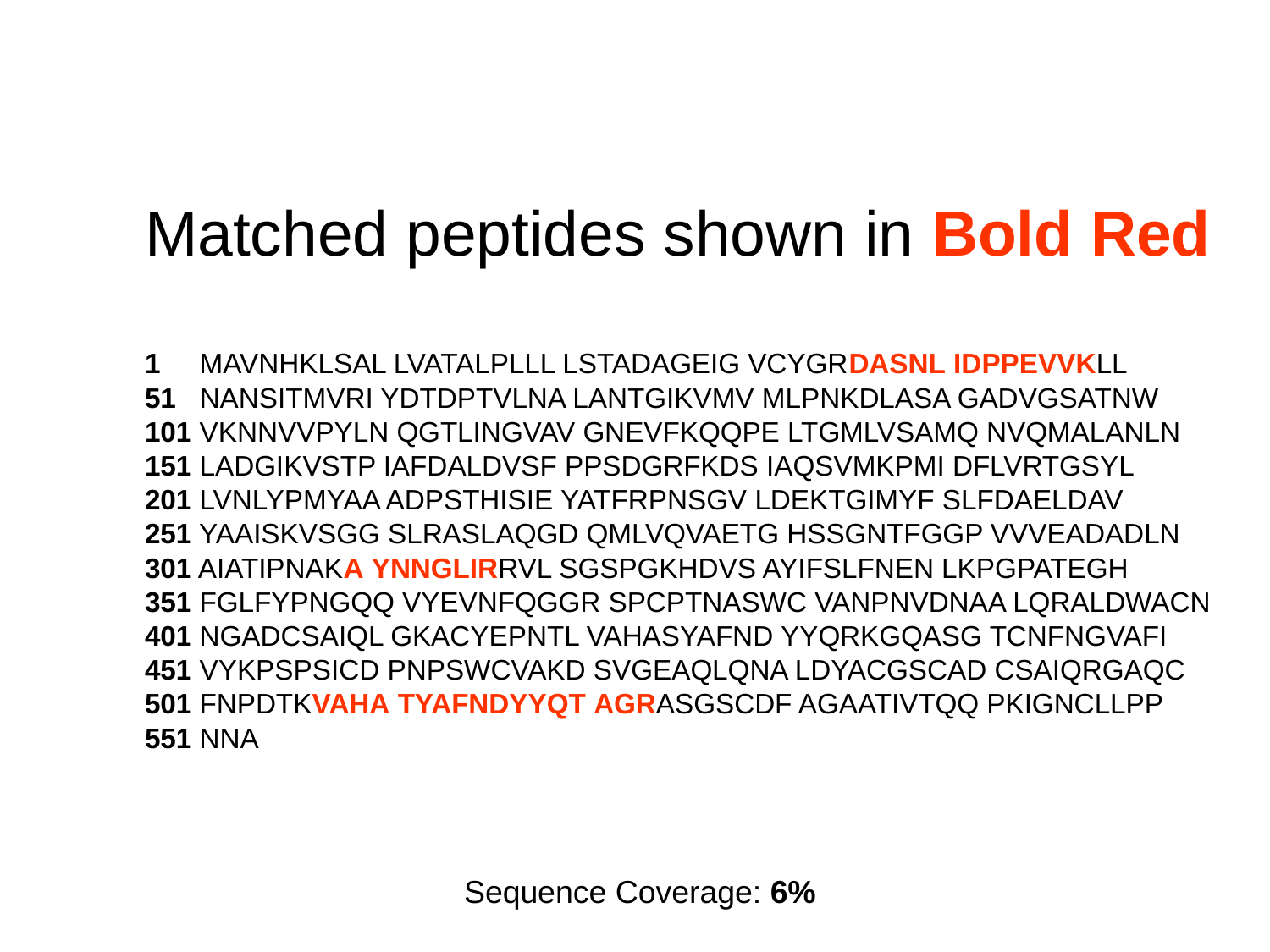

Matched peptides shown in Bold Red
1 MAVNHKLSAL LVATALPLLL LSTADAGEIG VCYGRDASNL IDPPEVVKLL
51 NANSITMVRI YDTDPTVLNA LANTGIKVMV MLPNKDLASA GADVGSATNW
101 VKNNVVPYLN QGTLINGVAV GNEVFKQQPE LTGMLVSAMQ NVQMALANLN
151 LADGIKVSTP IAFDALDVSF PPSDGRFKDS IAQSVMKPMI DFLVRTGSYL
201 LVNLYPMYAA ADPSTHISIE YATFRPNSGV LDEKTGIMYF SLFDAELDAV
251 YAAISKVSGG SLRASLAQGD QMLVQVAETG HSSGNTFGGP VVVEADADLN
301 AIATIPNAKA YNNGLIRRVL SGSPGKHDVS AYIFSLFNEN LKPGPATEGH
351 FGLFYPNGQQ VYEVNFQGGR SPCPTNASWC VANPNVDNAA LQRALDWACN
401 NGADCSAIQL GKACYEPNTL VAHASYAFND YYQRKGQASG TCNFNGVAFI
451 VYKPSPSICD PNPSWCVAKD SVGEAQLQNA LDYACGSCAD CSAIQRGAQC
501 FNPDTKVAHA TYAFNDYYQT AGRASGSCDF AGAATIVTQQ PKIGNCLLPP
551 NNA
# Sequence Coverage: 6%

## Slide 46
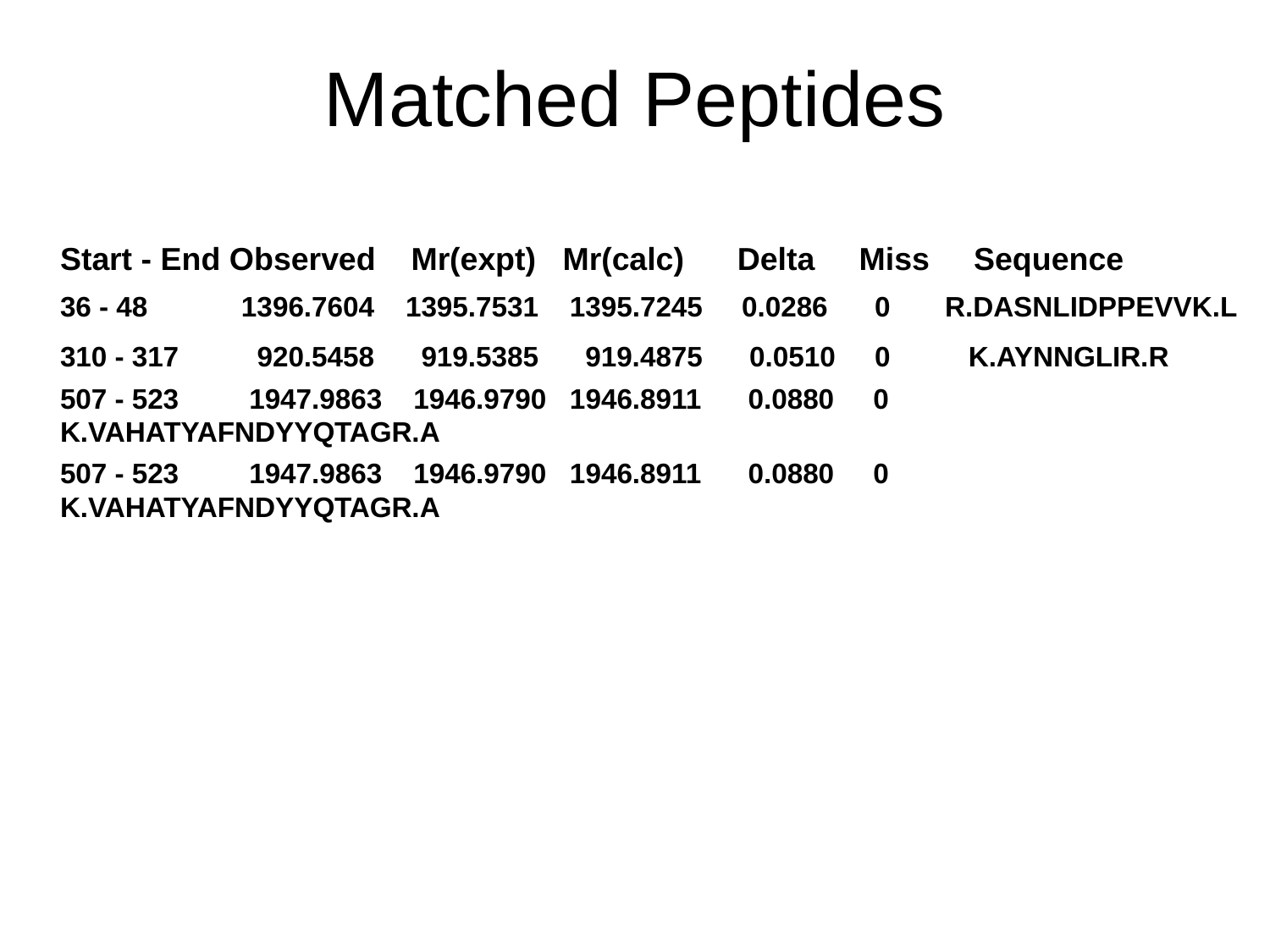

# Matched Peptides
Start - End Observed Mr(expt) Mr(calc) Delta Miss Sequence
36 - 48 1396.7604 1395.7531 1395.7245 0.0286 0 R.DASNLIDPPEVVK.L
310 - 317 920.5458 919.5385 919.4875 0.0510 0 K.AYNNGLIR.R
507 - 523 1947.9863 1946.9790 1946.8911 0.0880 0 K.VAHATYAFNDYYQTAGR.A
507 - 523 1947.9863 1946.9790 1946.8911 0.0880 0 K.VAHATYAFNDYYQTAGR.A

## Slide 47
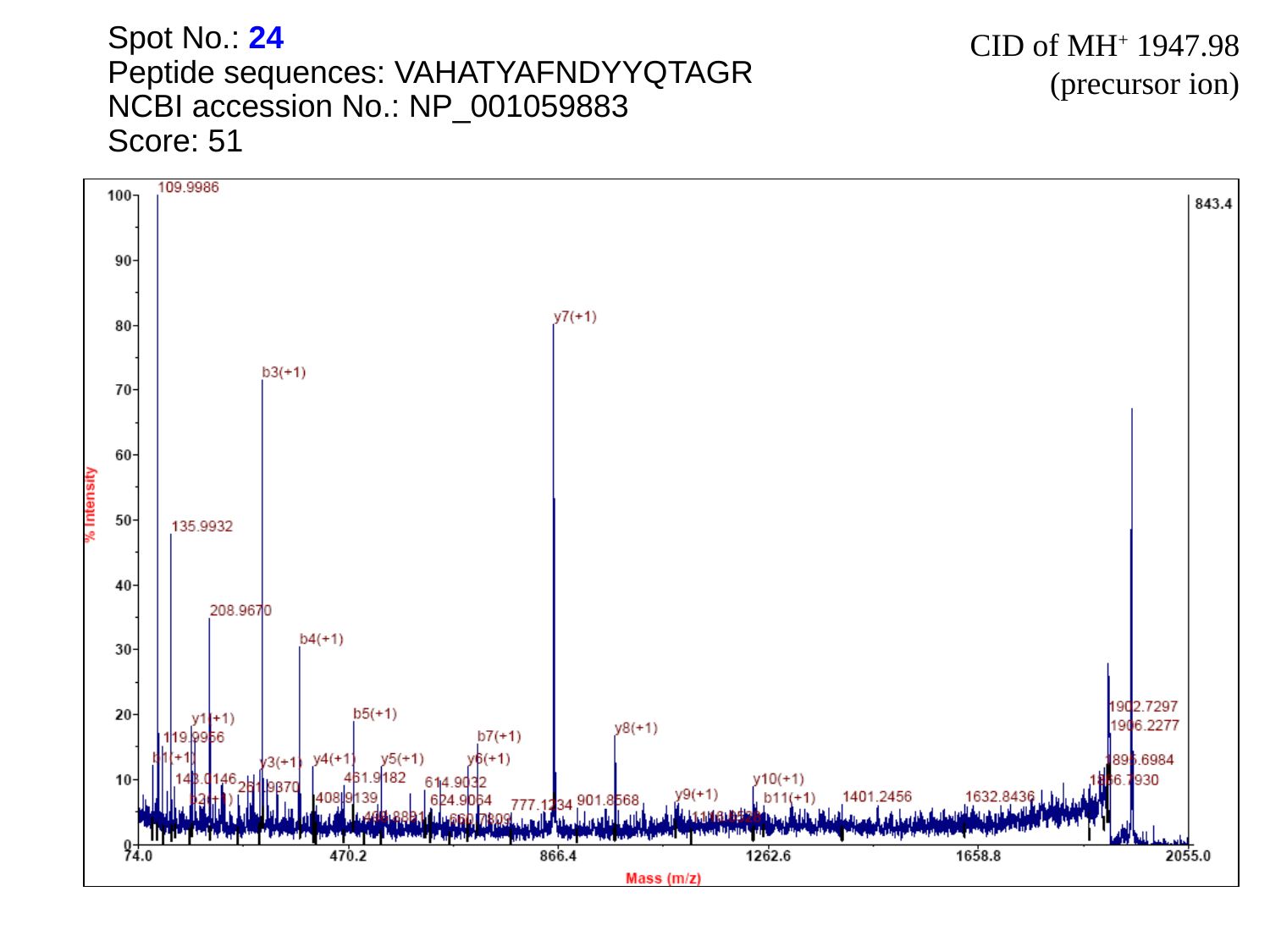

Spot No.: 24
Peptide sequences: VAHATYAFNDYYQTAGR
NCBI accession No.: NP_001059883
Score: 51
CID of MH+ 1947.98 (precursor ion)

## Slide 48
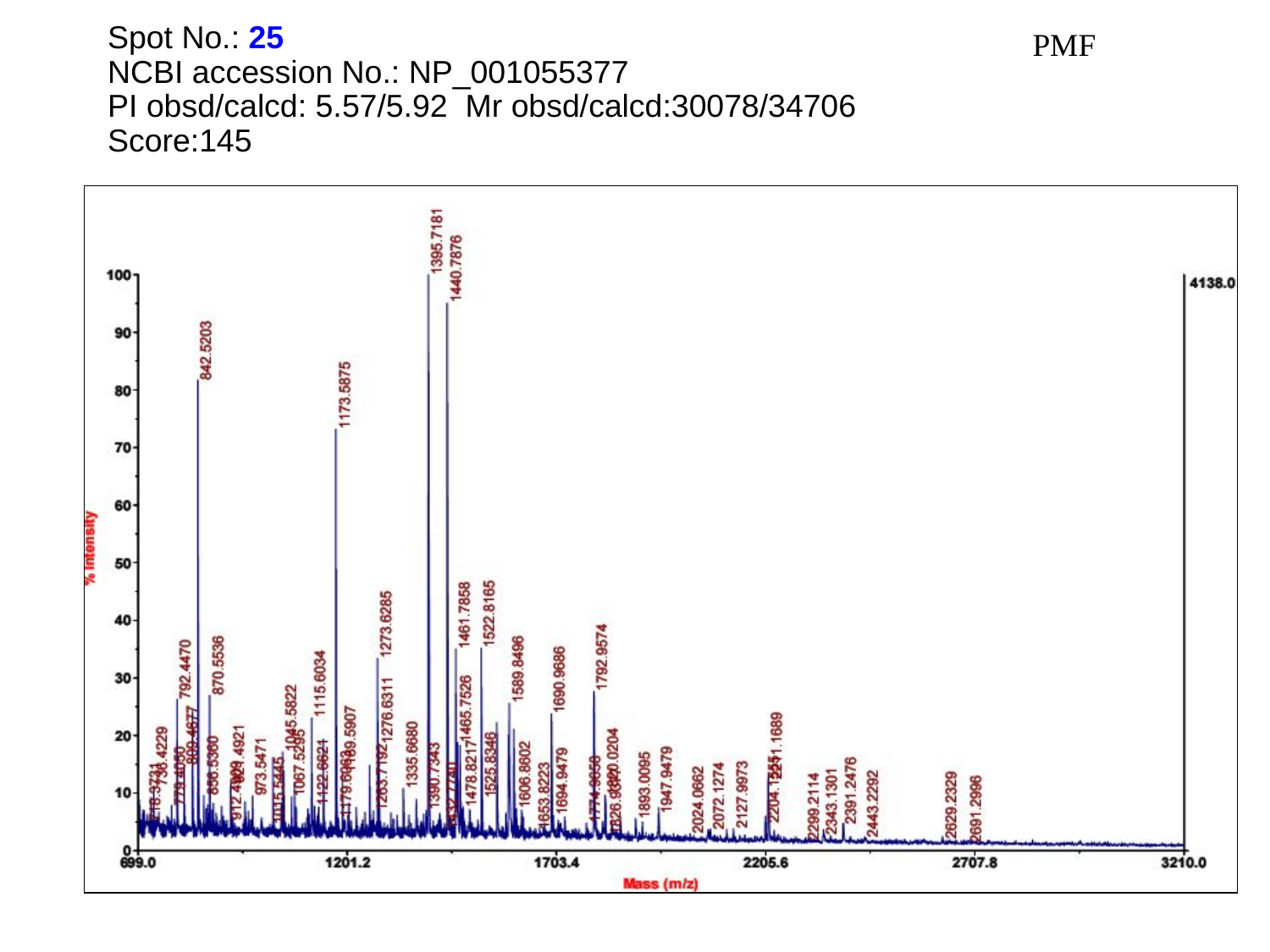

Spot No.: 25
NCBI accession No.: NP_001055377
PI obsd/calcd: 5.57/5.92 Mr obsd/calcd:30078/34706
Score:145
PMF

## Slide 49
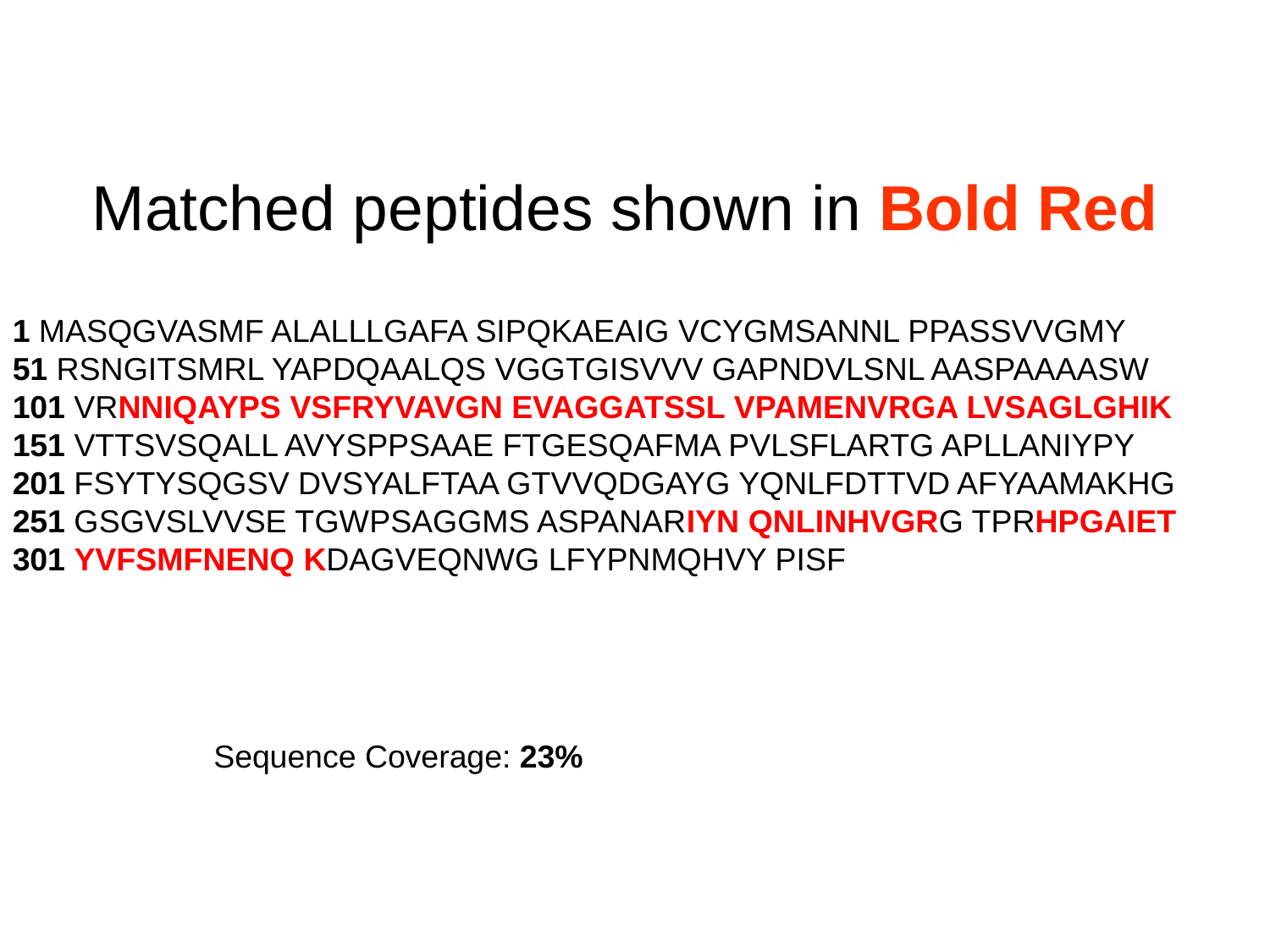

#
Matched peptides shown in Bold Red
1 MASQGVASMF ALALLLGAFA SIPQKAEAIG VCYGMSANNL PPASSVVGMY
51 RSNGITSMRL YAPDQAALQS VGGTGISVVV GAPNDVLSNL AASPAAAASW
101 VRNNIQAYPS VSFRYVAVGN EVAGGATSSL VPAMENVRGA LVSAGLGHIK
151 VTTSVSQALL AVYSPPSAAE FTGESQAFMA PVLSFLARTG APLLANIYPY
201 FSYTYSQGSV DVSYALFTAA GTVVQDGAYG YQNLFDTTVD AFYAAMAKHG
251 GSGVSLVVSE TGWPSAGGMS ASPANARIYN QNLINHVGRG TPRHPGAIET 301 YVFSMFNENQ KDAGVEQNWG LFYPNMQHVY PISF
Sequence Coverage: 23%

## Slide 50
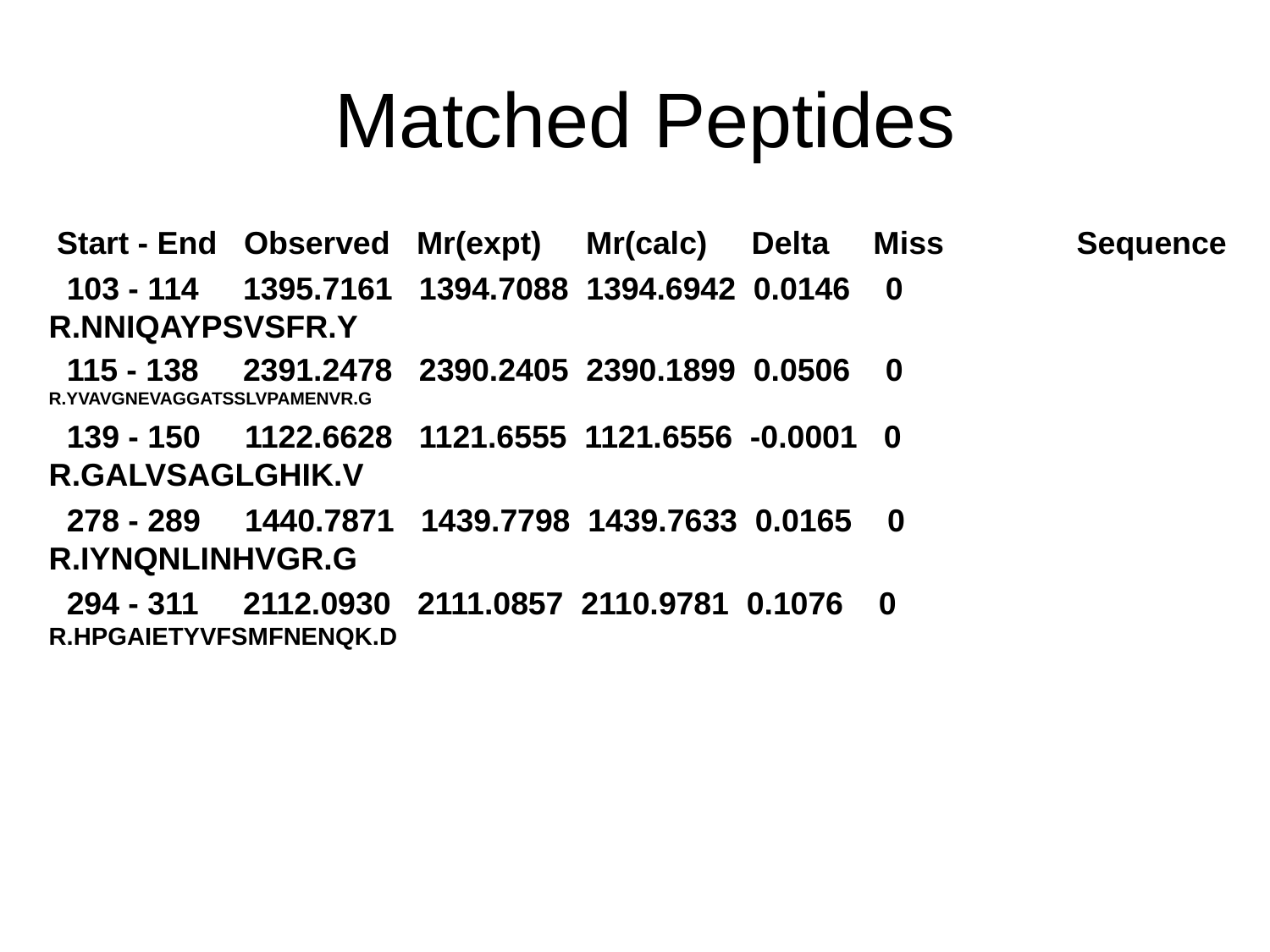

# Matched Peptides
 Start - End Observed Mr(expt) Mr(calc) Delta Miss Sequence
 103 - 114 1395.7161 1394.7088 1394.6942 0.0146 0 R.NNIQAYPSVSFR.Y
 115 - 138 2391.2478 2390.2405 2390.1899 0.0506 0 R.YVAVGNEVAGGATSSLVPAMENVR.G
 139 - 150 1122.6628 1121.6555 1121.6556 -0.0001 0 R.GALVSAGLGHIK.V
 278 - 289 1440.7871 1439.7798 1439.7633 0.0165 0 R.IYNQNLINHVGR.G
 294 - 311 2112.0930 2111.0857 2110.9781 0.1076 0 R.HPGAIETYVFSMFNENQK.D

## Slide 51
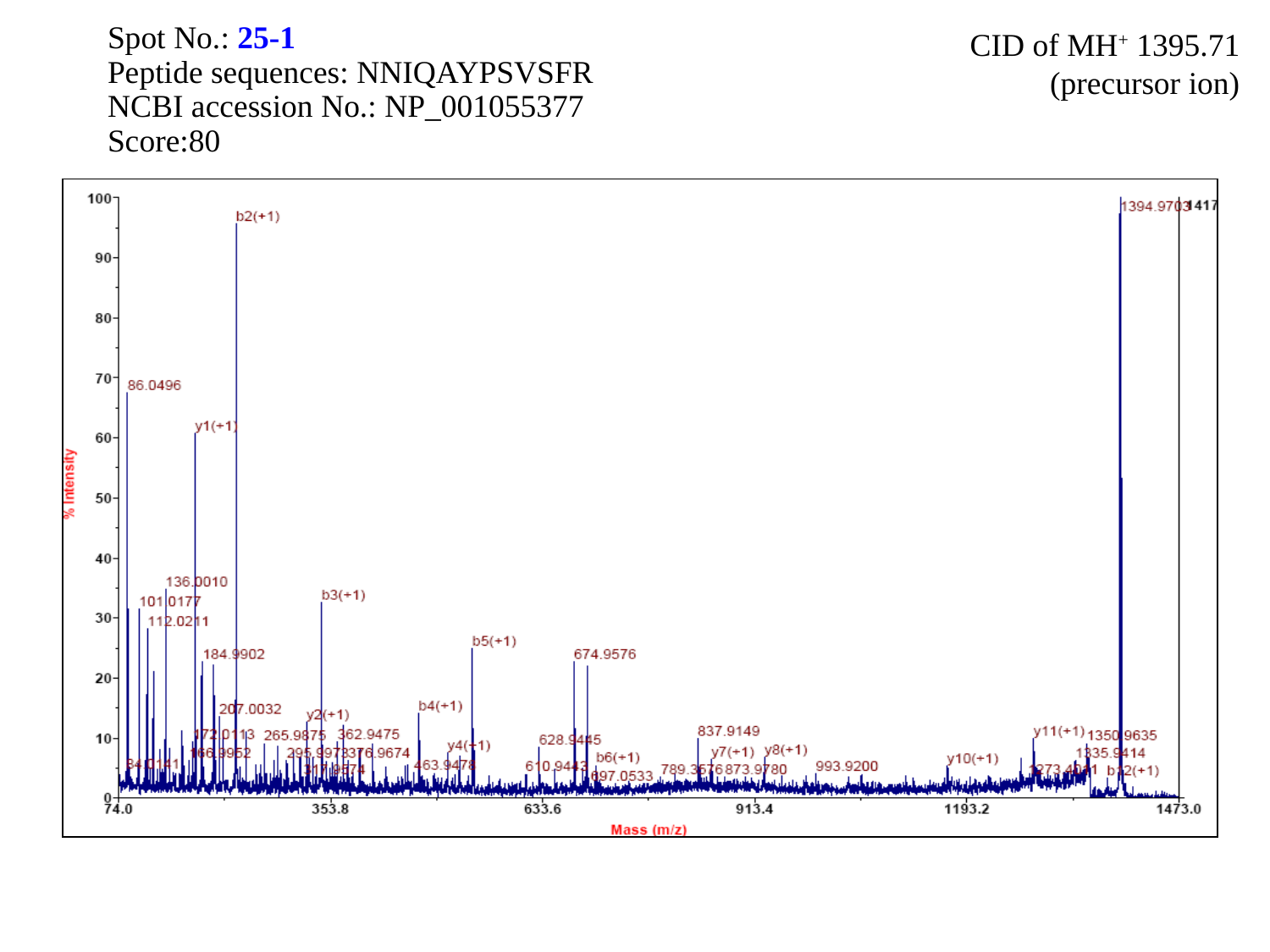

Spot No.: 25-1
Peptide sequences: NNIQAYPSVSFR
NCBI accession No.: NP_001055377
Score:80
CID of MH+ 1395.71 (precursor ion)

## Slide 52
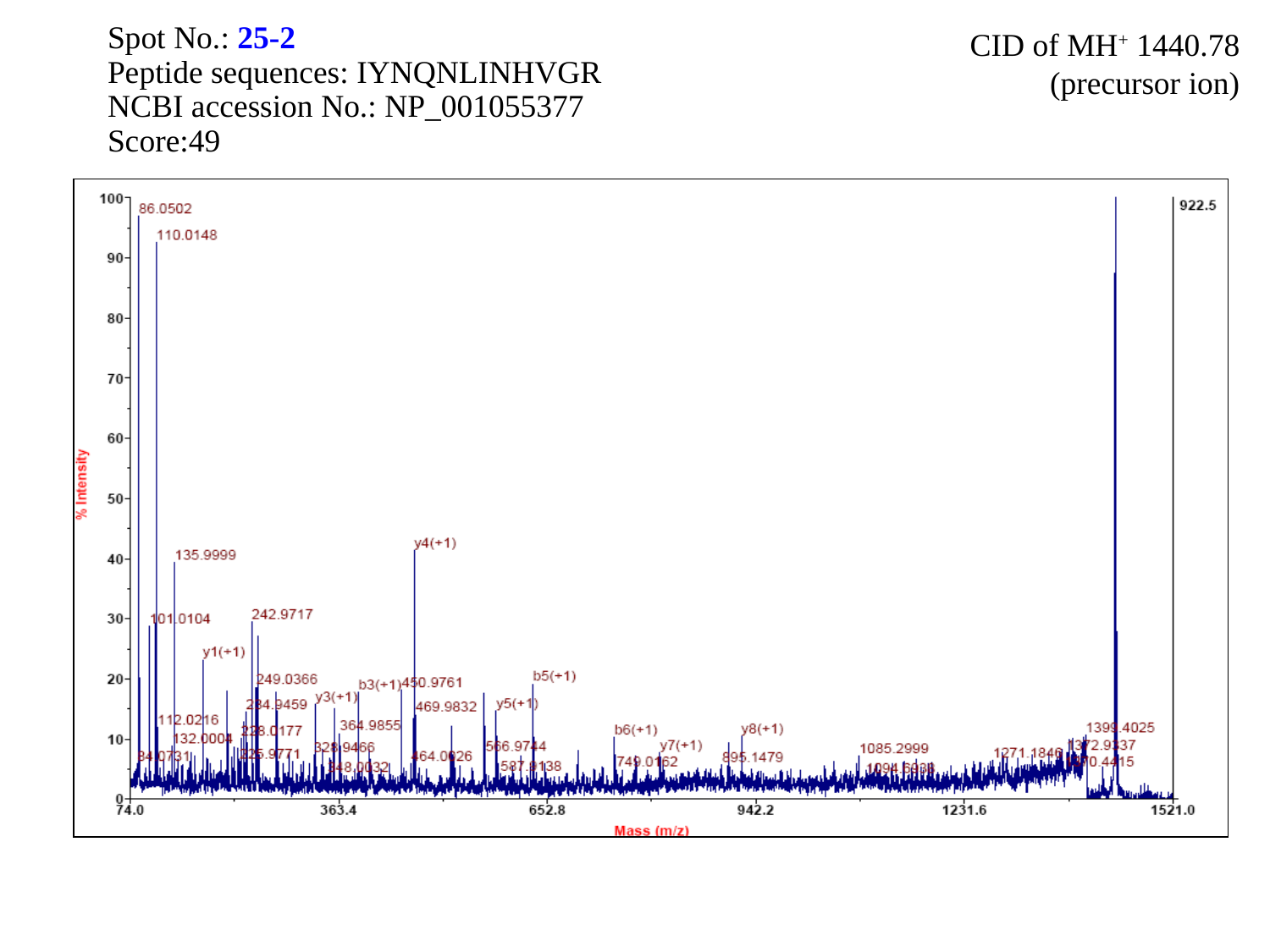

Spot No.: 25-2
Peptide sequences: IYNQNLINHVGR
NCBI accession No.: NP_001055377
Score:49
CID of MH+ 1440.78 (precursor ion)

## Slide 53
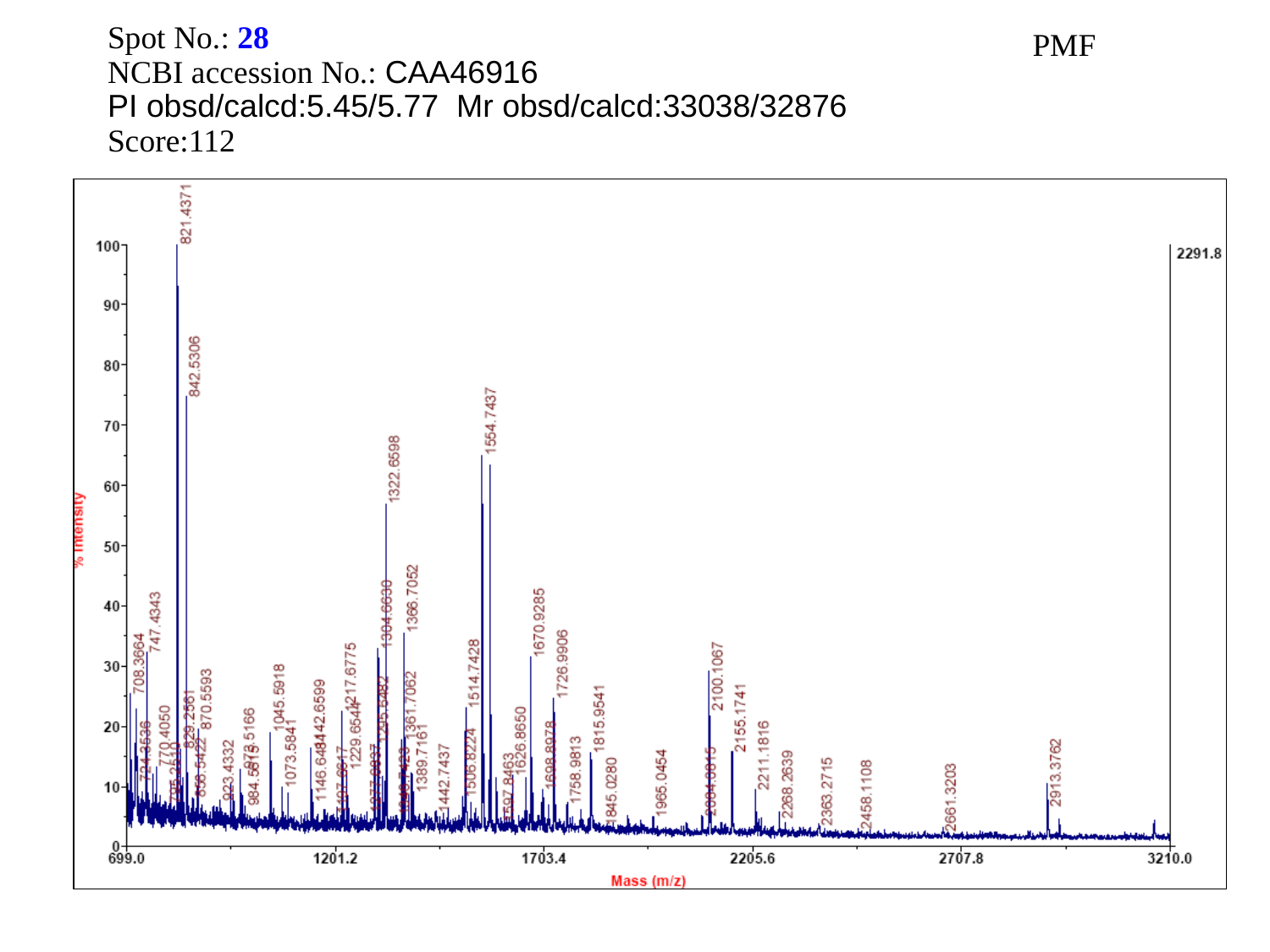

Spot No.: 28
NCBI accession No.: CAA46916
PI obsd/calcd:5.45/5.77 Mr obsd/calcd:33038/32876
Score:112
PMF

## Slide 54
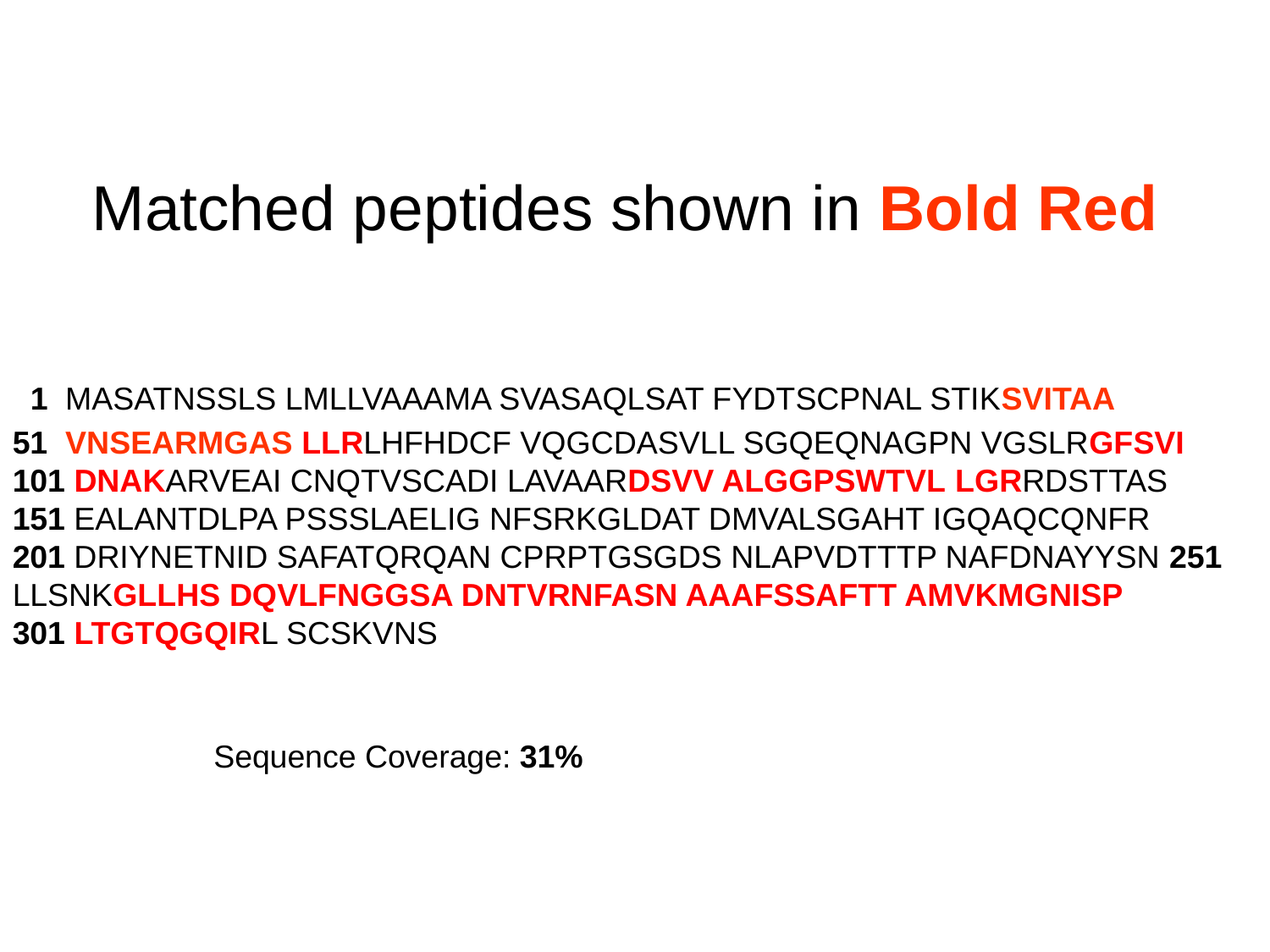

#
Matched peptides shown in Bold Red
 1 MASATNSSLS LMLLVAAAMA SVASAQLSAT FYDTSCPNAL STIKSVITAA
51 VNSEARMGAS LLRLHFHDCF VQGCDASVLL SGQEQNAGPN VGSLRGFSVI 101 DNAKARVEAI CNQTVSCADI LAVAARDSVV ALGGPSWTVL LGRRDSTTAS 151 EALANTDLPA PSSSLAELIG NFSRKGLDAT DMVALSGAHT IGQAQCQNFR
201 DRIYNETNID SAFATQRQAN CPRPTGSGDS NLAPVDTTTP NAFDNAYYSN 251 LLSNKGLLHS DQVLFNGGSA DNTVRNFASN AAAFSSAFTT AMVKMGNISP
301 LTGTQGQIRL SCSKVNS
Sequence Coverage: 31%

## Slide 55
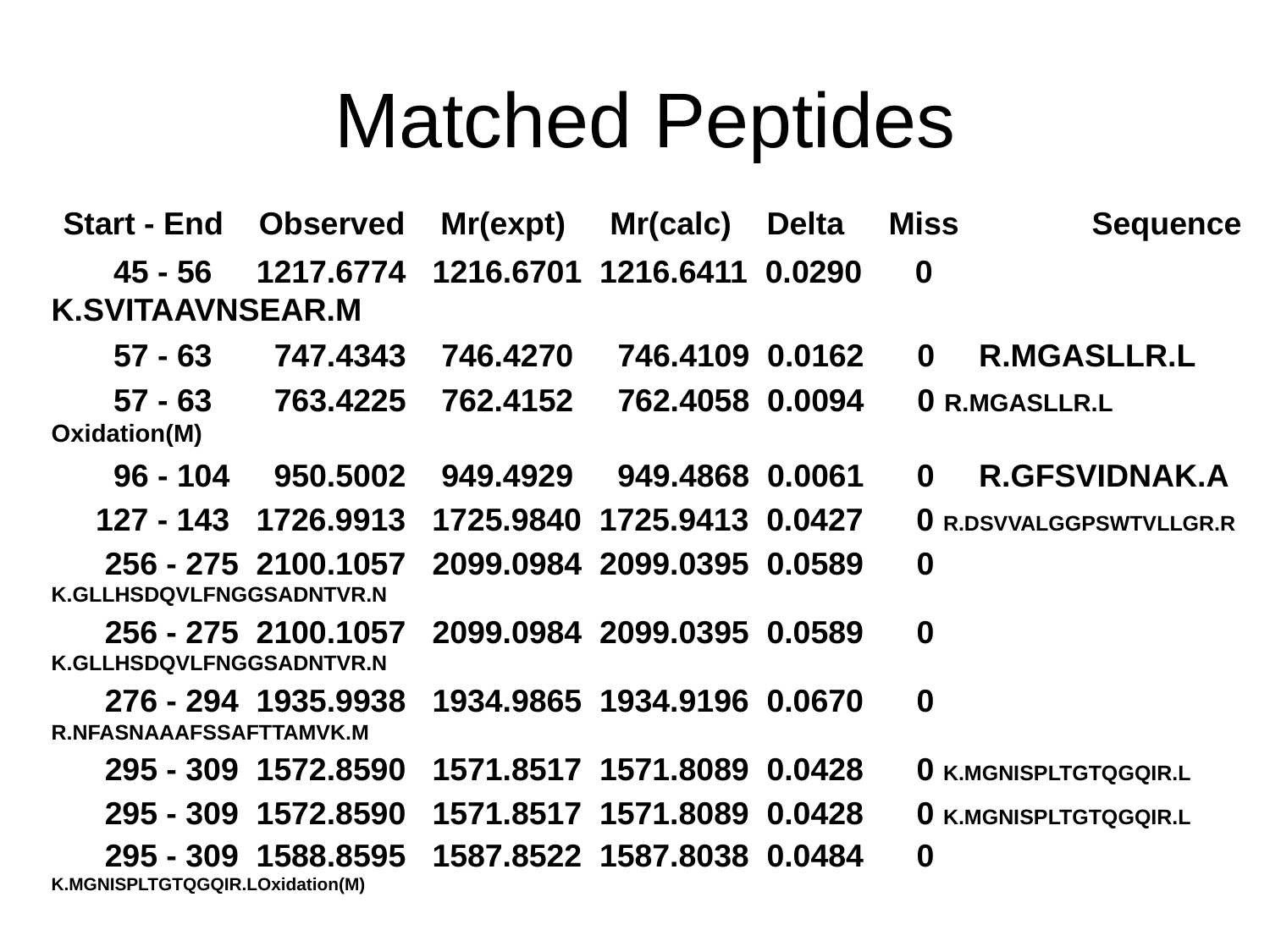

# Matched Peptides
 Start - End Observed Mr(expt) Mr(calc) Delta Miss Sequence
 45 - 56 1217.6774 1216.6701 1216.6411 0.0290 0 K.SVITAAVNSEAR.M
 57 - 63 747.4343 746.4270 746.4109 0.0162 0 R.MGASLLR.L
 57 - 63 763.4225 762.4152 762.4058 0.0094 0 R.MGASLLR.L Oxidation(M)
 96 - 104 950.5002 949.4929 949.4868 0.0061 0 R.GFSVIDNAK.A
 127 - 143 1726.9913 1725.9840 1725.9413 0.0427 0 R.DSVVALGGPSWTVLLGR.R
 256 - 275 2100.1057 2099.0984 2099.0395 0.0589 0 K.GLLHSDQVLFNGGSADNTVR.N
 256 - 275 2100.1057 2099.0984 2099.0395 0.0589 0 K.GLLHSDQVLFNGGSADNTVR.N
 276 - 294 1935.9938 1934.9865 1934.9196 0.0670 0 R.NFASNAAAFSSAFTTAMVK.M
 295 - 309 1572.8590 1571.8517 1571.8089 0.0428 0 K.MGNISPLTGTQGQIR.L
 295 - 309 1572.8590 1571.8517 1571.8089 0.0428 0 K.MGNISPLTGTQGQIR.L
 295 - 309 1588.8595 1587.8522 1587.8038 0.0484 0 K.MGNISPLTGTQGQIR.LOxidation(M)

## Slide 56
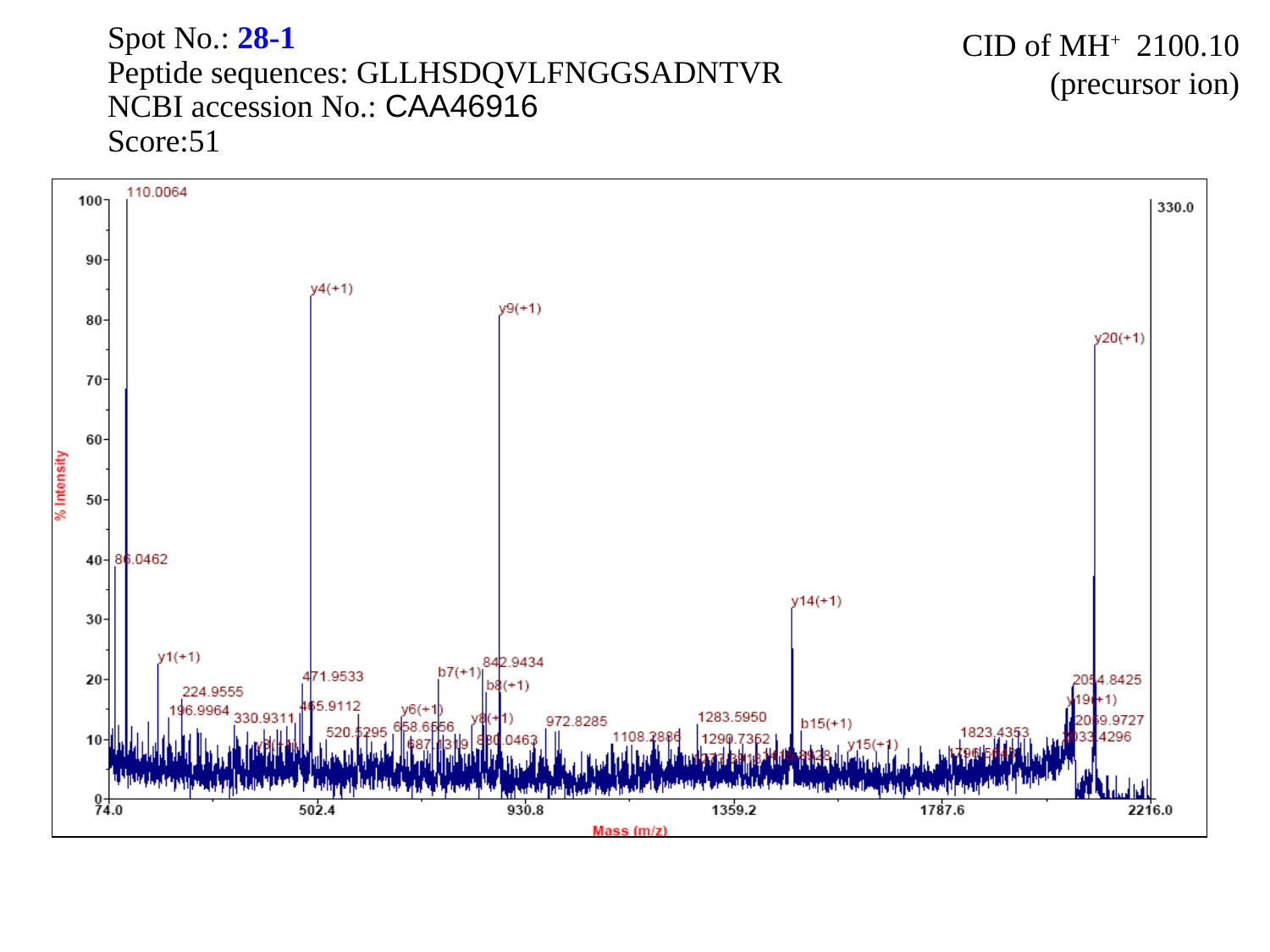

Spot No.: 28-1
Peptide sequences: GLLHSDQVLFNGGSADNTVR NCBI accession No.: CAA46916
Score:51
CID of MH+ 2100.10 (precursor ion)

## Slide 57
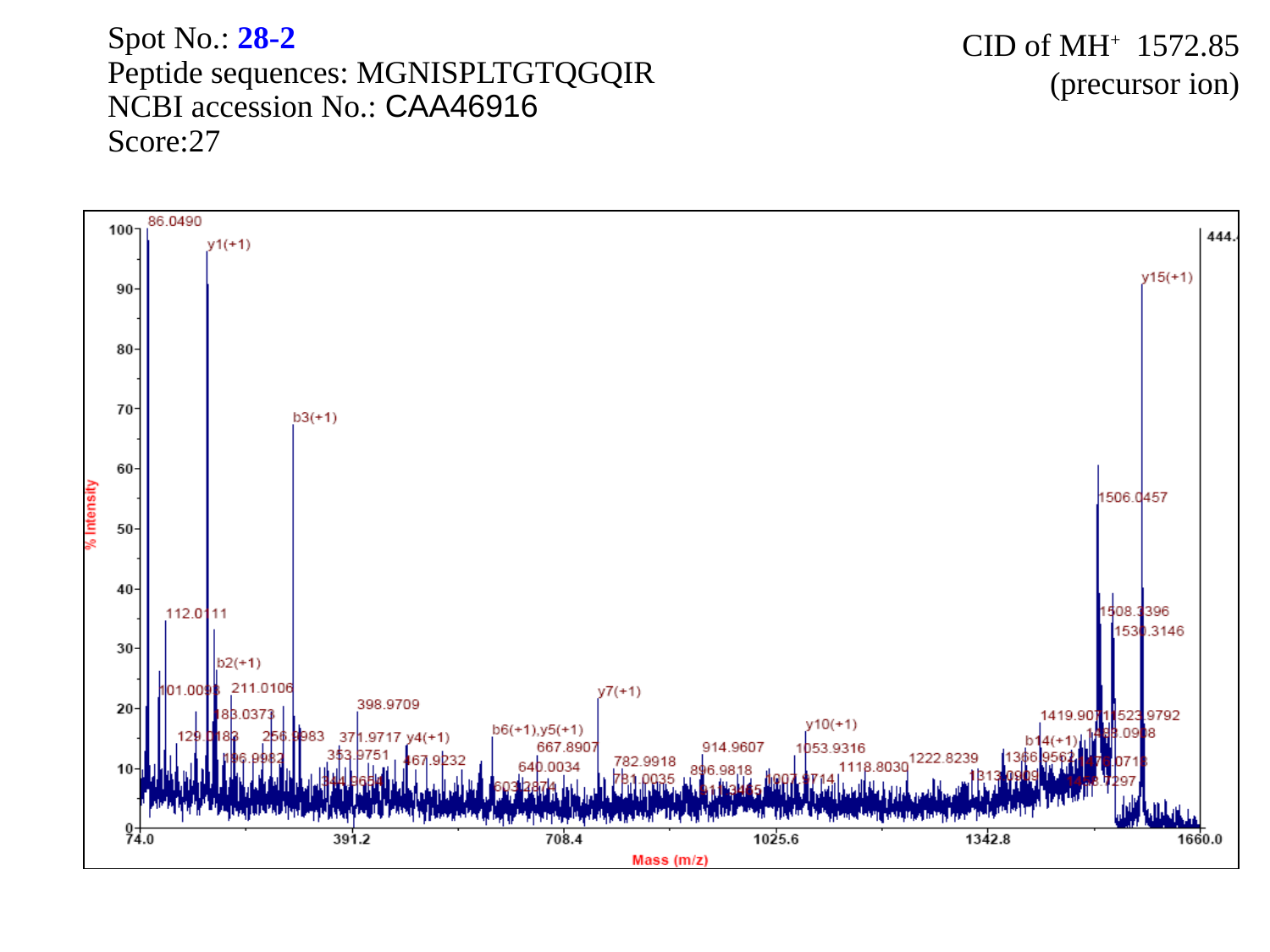

Spot No.: 28-2
Peptide sequences: MGNISPLTGTQGQIR
NCBI accession No.: CAA46916
Score:27
CID of MH+ 1572.85 (precursor ion)

## Slide 58
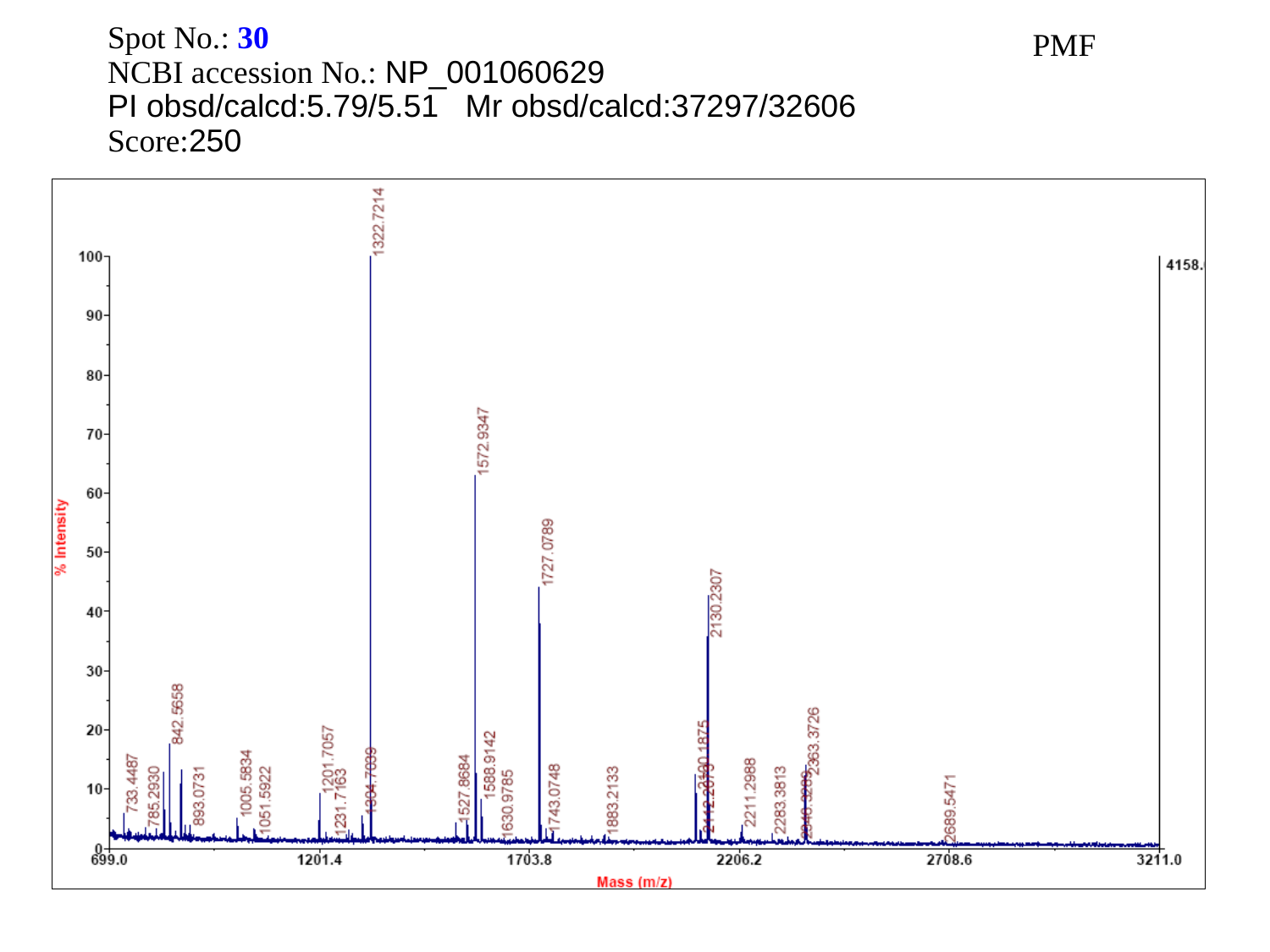

Spot No.: 30
NCBI accession No.: NP_001060629
PI obsd/calcd:5.79/5.51 Mr obsd/calcd:37297/32606
Score:250
PMF

## Slide 59
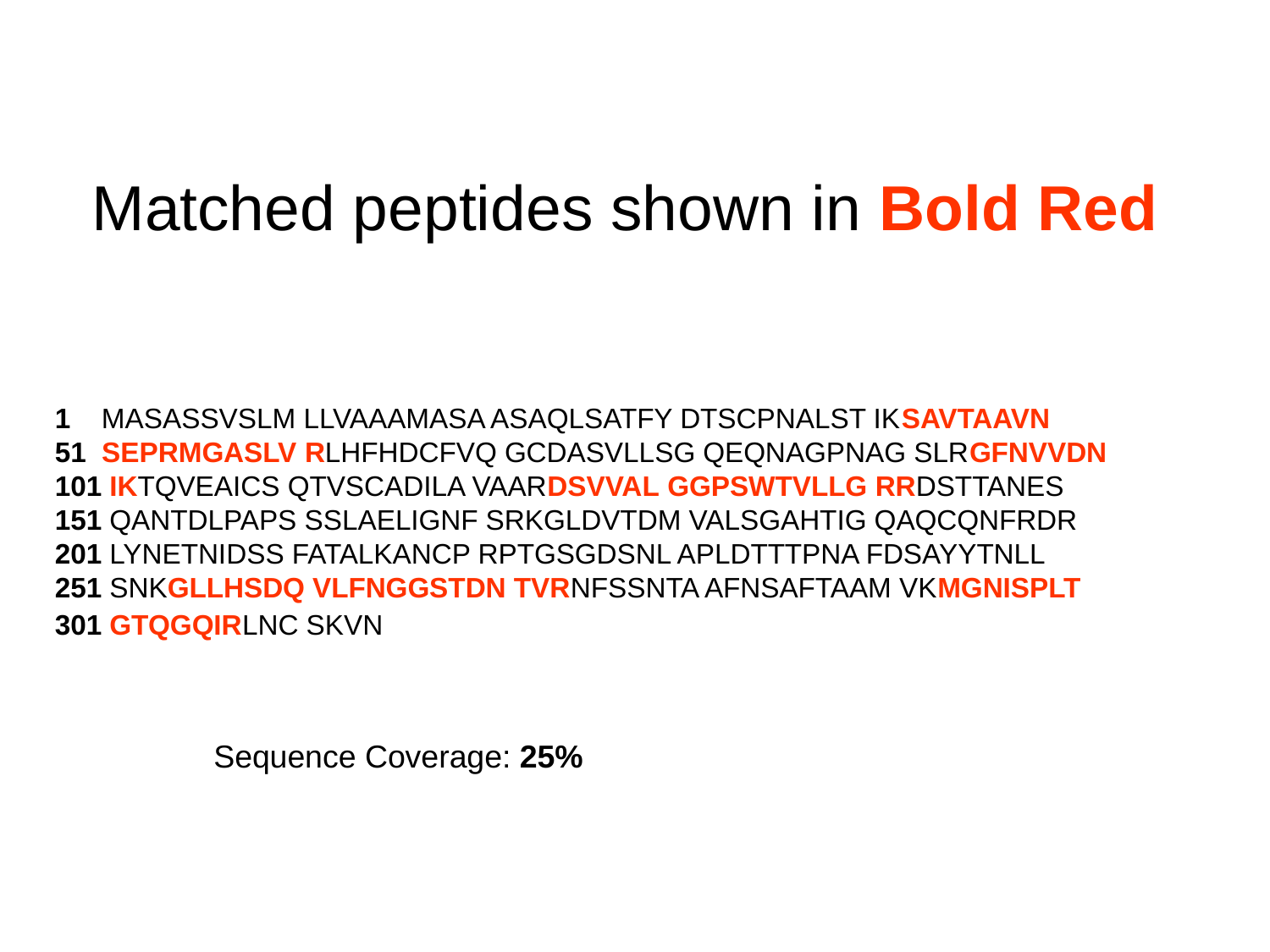

#
Matched peptides shown in Bold Red
1 MASASSVSLM LLVAAAMASA ASAQLSATFY DTSCPNALST IKSAVTAAVN
51 SEPRMGASLV RLHFHDCFVQ GCDASVLLSG QEQNAGPNAG SLRGFNVVDN
101 IKTQVEAICS QTVSCADILA VAARDSVVAL GGPSWTVLLG RRDSTTANES
151 QANTDLPAPS SSLAELIGNF SRKGLDVTDM VALSGAHTIG QAQCQNFRDR
201 LYNETNIDSS FATALKANCP RPTGSGDSNL APLDTTTPNA FDSAYYTNLL
251 SNKGLLHSDQ VLFNGGSTDN TVRNFSSNTA AFNSAFTAAM VKMGNISPLT
301 GTQGQIRLNC SKVN
Sequence Coverage: 25%

## Slide 60
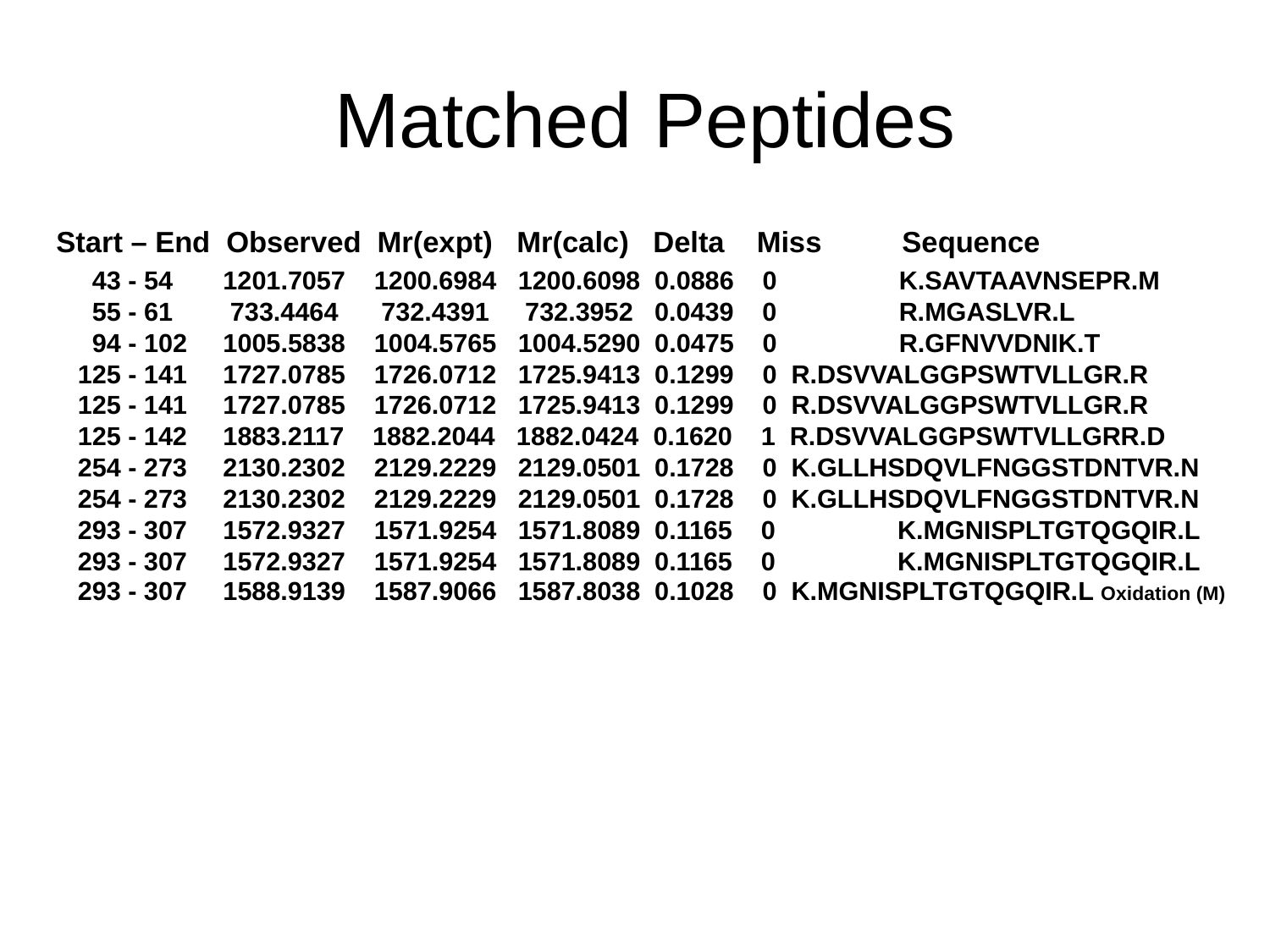

# Matched Peptides
Start – End Observed Mr(expt) Mr(calc) Delta Miss Sequence
 43 - 54 1201.7057 1200.6984 1200.6098 0.0886 0 K.SAVTAAVNSEPR.M
 55 - 61 733.4464 732.4391 732.3952 0.0439 0 R.MGASLVR.L
 94 - 102 1005.5838 1004.5765 1004.5290 0.0475 0 R.GFNVVDNIK.T
 125 - 141 1727.0785 1726.0712 1725.9413 0.1299 0 R.DSVVALGGPSWTVLLGR.R
 125 - 141 1727.0785 1726.0712 1725.9413 0.1299 0 R.DSVVALGGPSWTVLLGR.R
 125 - 142 1883.2117 1882.2044 1882.0424 0.1620 1 R.DSVVALGGPSWTVLLGRR.D
 254 - 273 2130.2302 2129.2229 2129.0501 0.1728 0 K.GLLHSDQVLFNGGSTDNTVR.N
 254 - 273 2130.2302 2129.2229 2129.0501 0.1728 0 K.GLLHSDQVLFNGGSTDNTVR.N
 293 - 307 1572.9327 1571.9254 1571.8089 0.1165 0 K.MGNISPLTGTQGQIR.L
 293 - 307 1572.9327 1571.9254 1571.8089 0.1165 0 K.MGNISPLTGTQGQIR.L
 293 - 307 1588.9139 1587.9066 1587.8038 0.1028 0 K.MGNISPLTGTQGQIR.L Oxidation (M)

## Slide 61
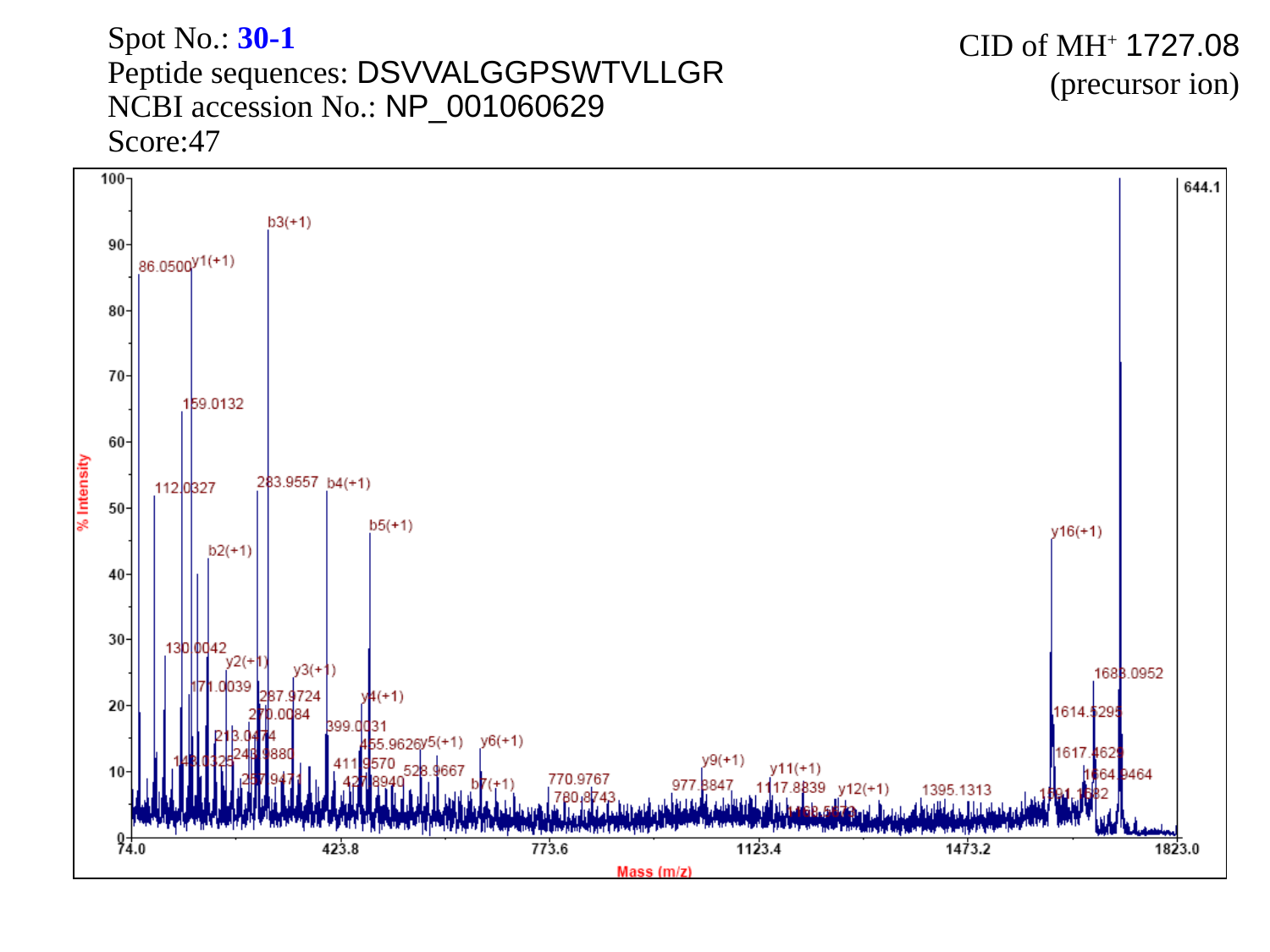

Spot No.: 30-1
Peptide sequences: DSVVALGGPSWTVLLGR
NCBI accession No.: NP_001060629
Score:47
CID of MH+ 1727.08 (precursor ion)

## Slide 62
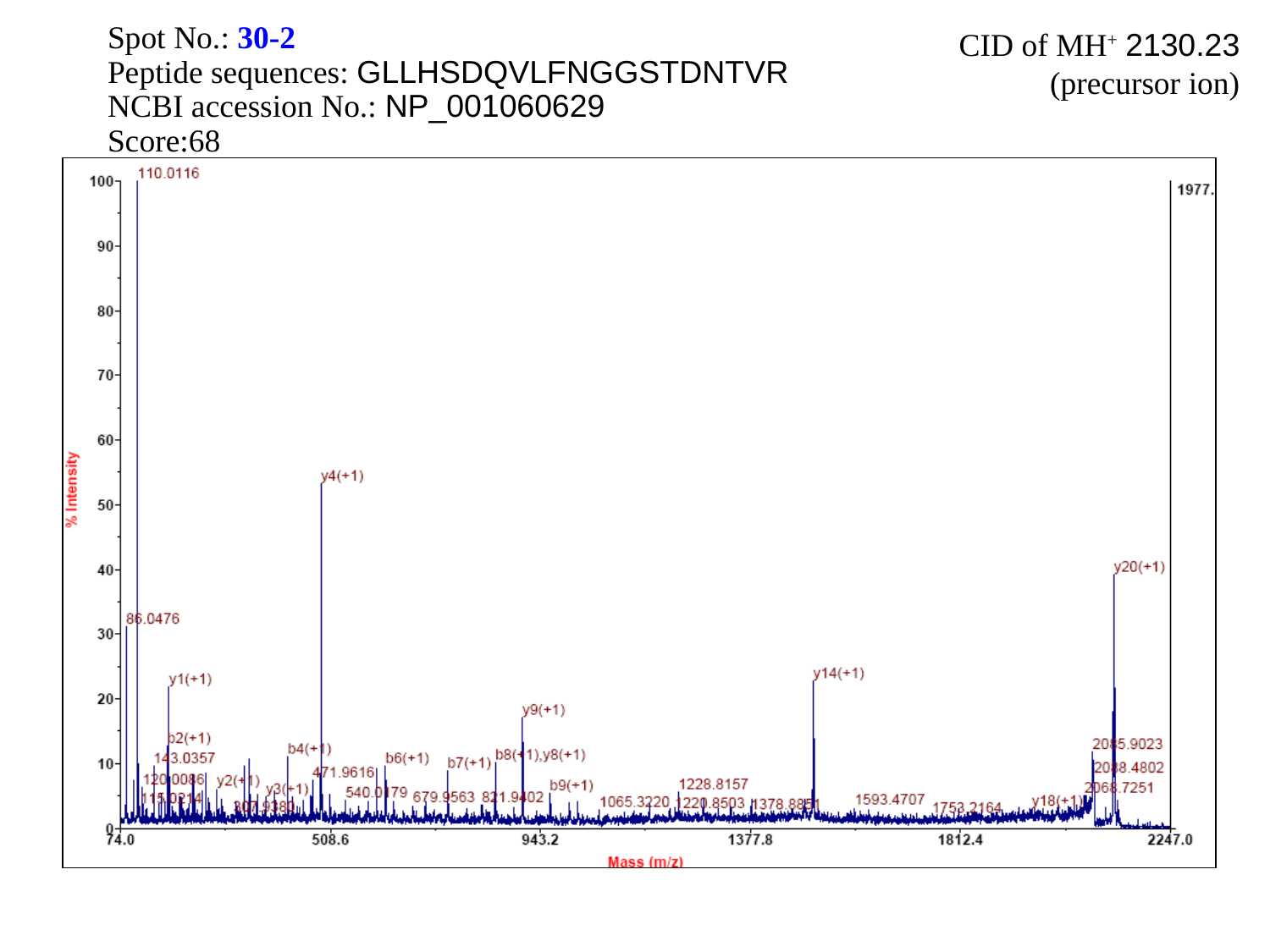

Spot No.: 30-2
Peptide sequences: GLLHSDQVLFNGGSTDNTVR
NCBI accession No.: NP_001060629
Score:68
CID of MH+ 2130.23 (precursor ion)

## Slide 63
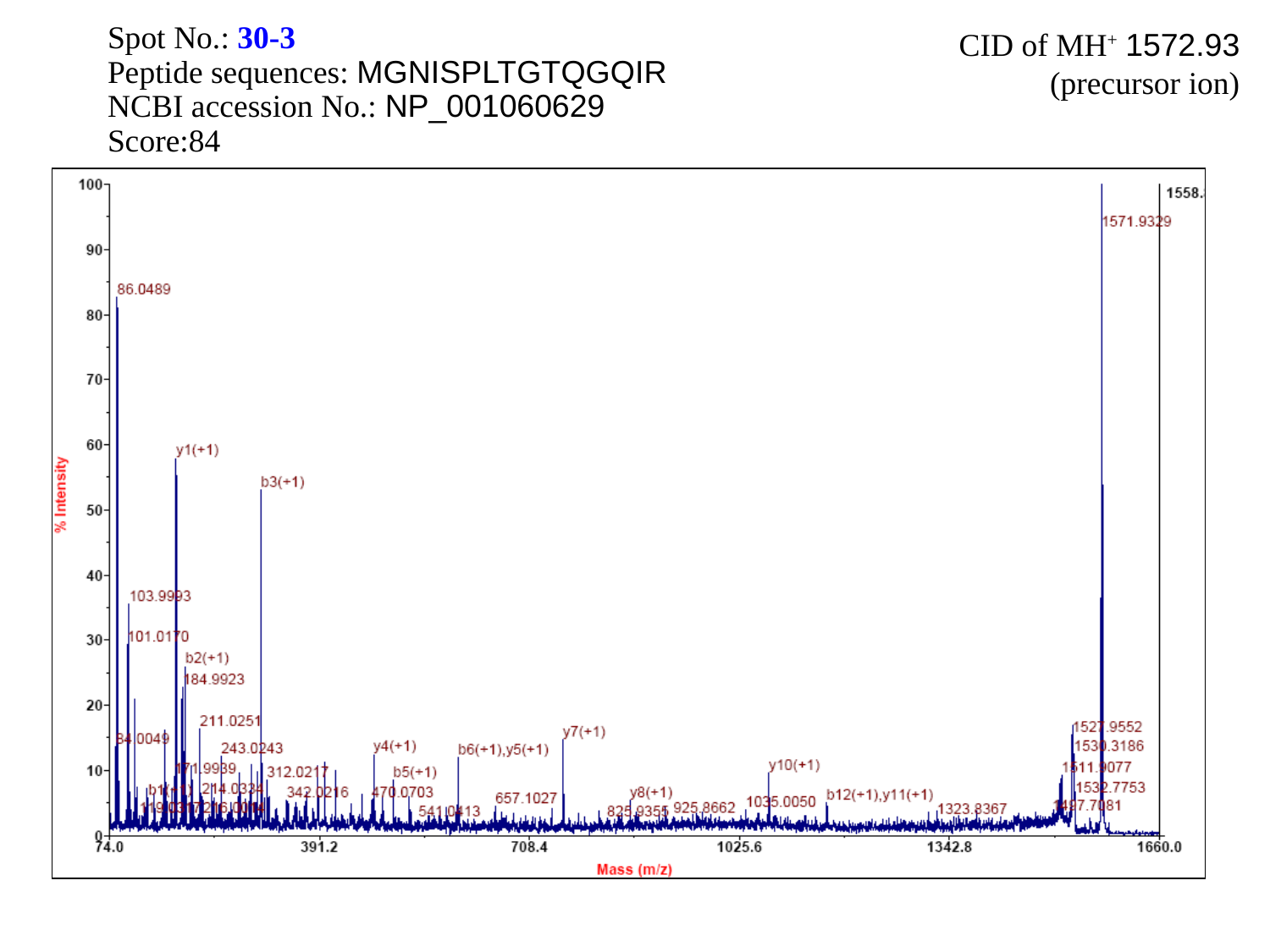

Spot No.: 30-3
Peptide sequences: MGNISPLTGTQGQIR
NCBI accession No.: NP_001060629
Score:84
CID of MH+ 1572.93 (precursor ion)

## Slide 64
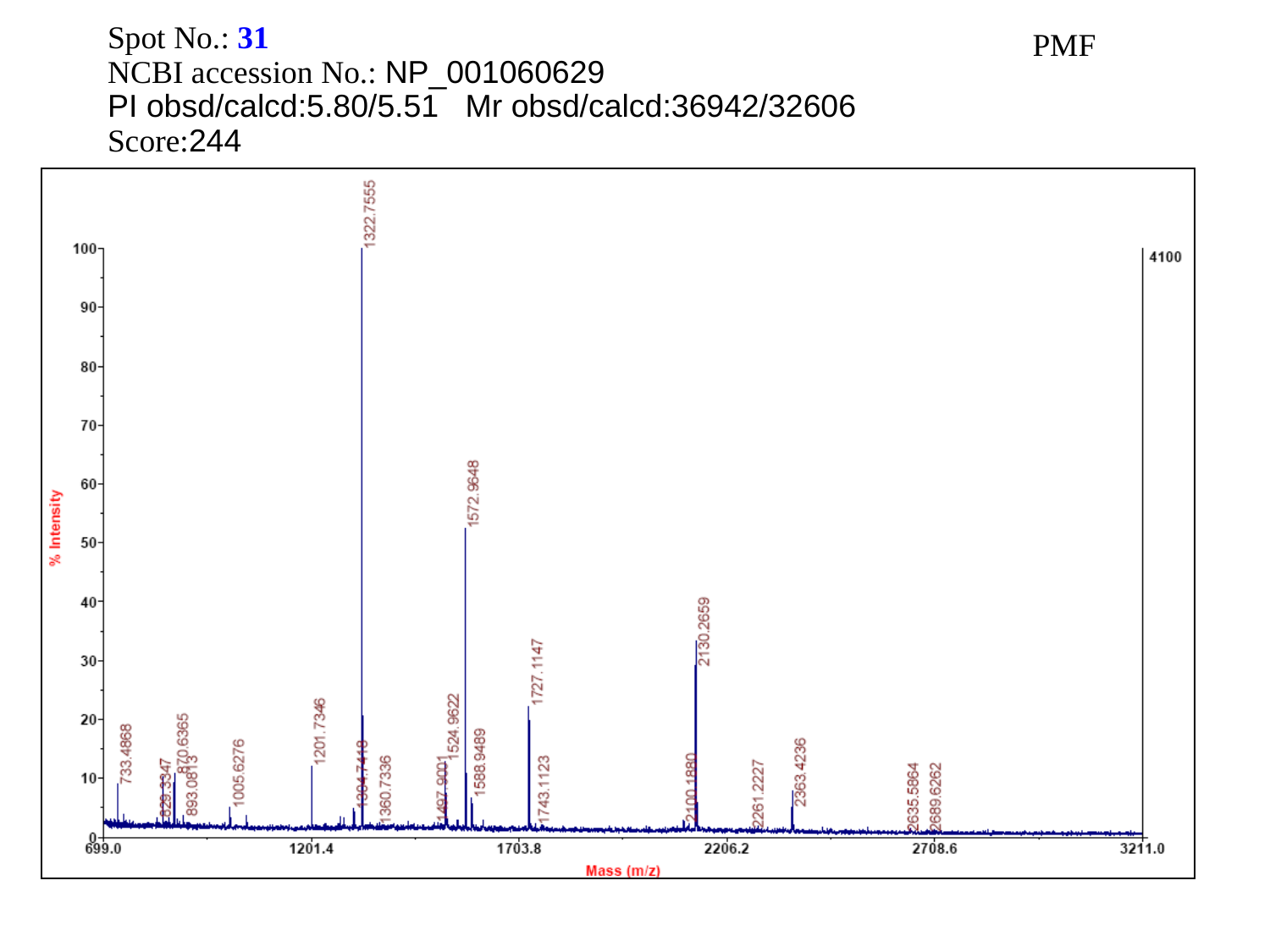

Spot No.: 31
NCBI accession No.: NP_001060629
PI obsd/calcd:5.80/5.51 Mr obsd/calcd:36942/32606
Score:244
PMF

## Slide 65
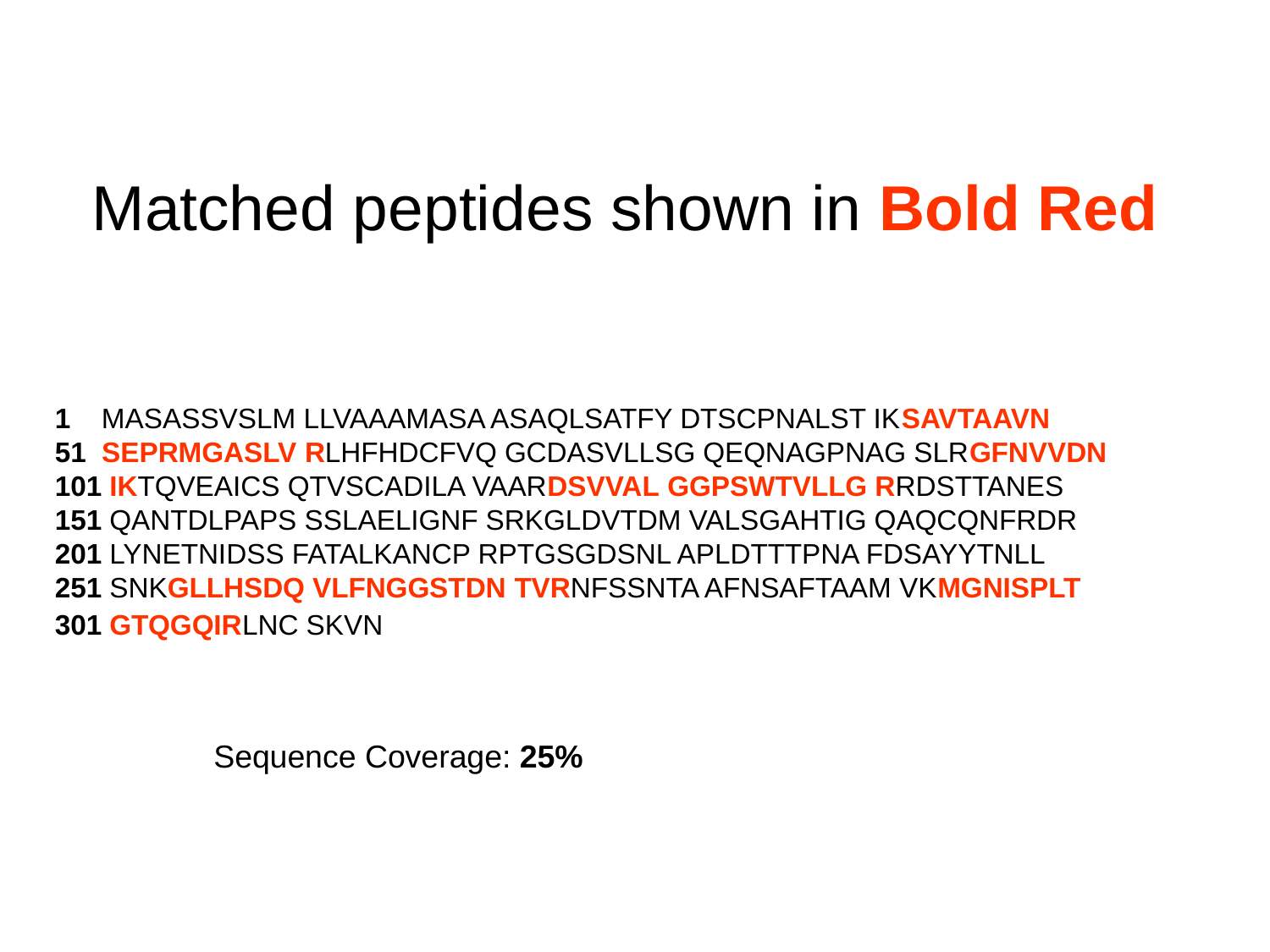

#
Matched peptides shown in Bold Red
1 MASASSVSLM LLVAAAMASA ASAQLSATFY DTSCPNALST IKSAVTAAVN
51 SEPRMGASLV RLHFHDCFVQ GCDASVLLSG QEQNAGPNAG SLRGFNVVDN
101 IKTQVEAICS QTVSCADILA VAARDSVVAL GGPSWTVLLG RRDSTTANES
151 QANTDLPAPS SSLAELIGNF SRKGLDVTDM VALSGAHTIG QAQCQNFRDR
201 LYNETNIDSS FATALKANCP RPTGSGDSNL APLDTTTPNA FDSAYYTNLL
251 SNKGLLHSDQ VLFNGGSTDN TVRNFSSNTA AFNSAFTAAM VKMGNISPLT
301 GTQGQIRLNC SKVN
Sequence Coverage: 25%

## Slide 66
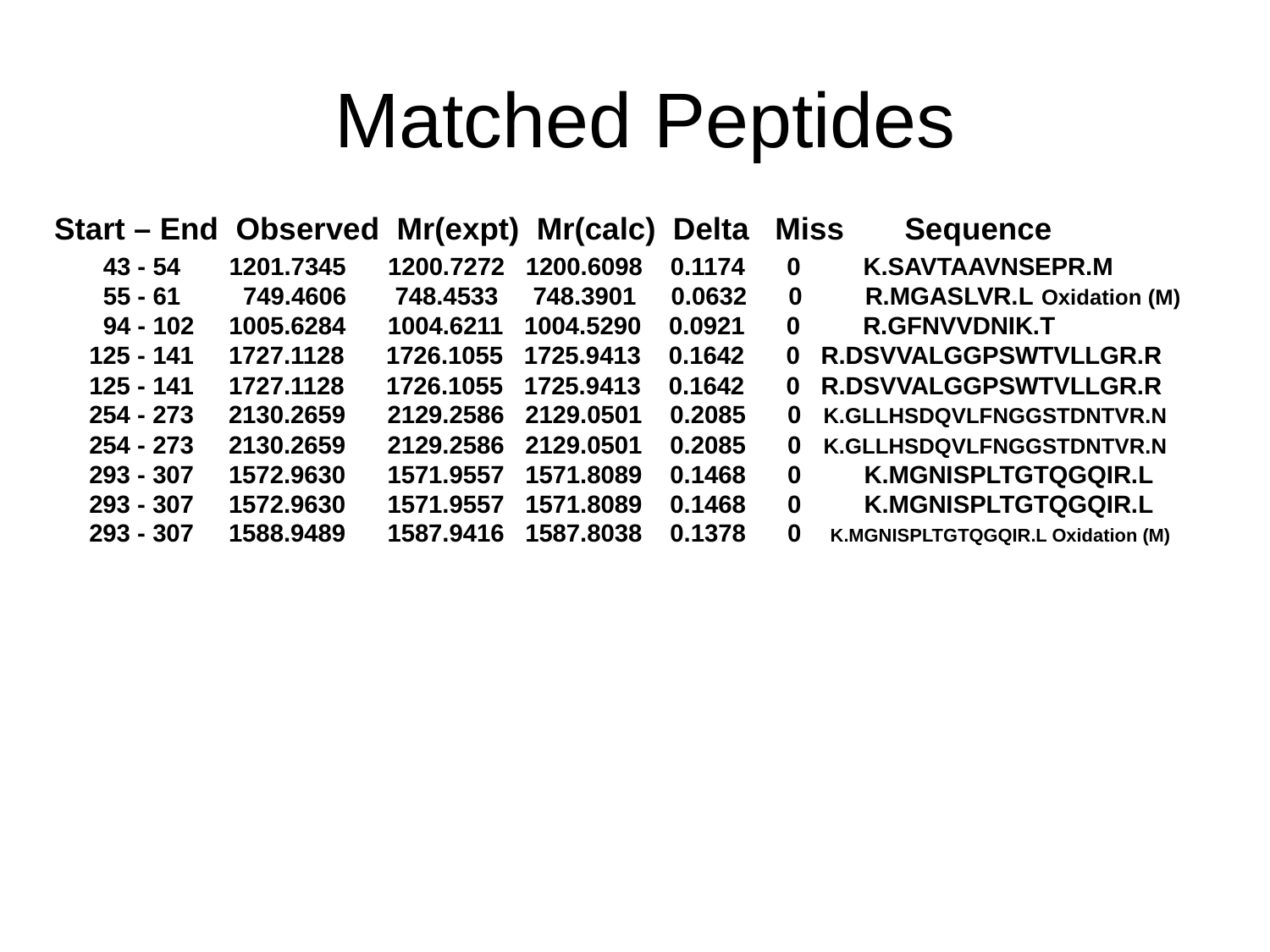

# Matched Peptides
Start – End Observed Mr(expt) Mr(calc) Delta Miss Sequence
 43 - 54 1201.7345 1200.7272 1200.6098 0.1174 0 K.SAVTAAVNSEPR.M
 55 - 61 749.4606 748.4533 748.3901 0.0632 0 R.MGASLVR.L Oxidation (M)
 94 - 102 1005.6284 1004.6211 1004.5290 0.0921 0 R.GFNVVDNIK.T
 125 - 141 1727.1128 1726.1055 1725.9413 0.1642 0 R.DSVVALGGPSWTVLLGR.R
 125 - 141 1727.1128 1726.1055 1725.9413 0.1642 0 R.DSVVALGGPSWTVLLGR.R
 254 - 273 2130.2659 2129.2586 2129.0501 0.2085 0 K.GLLHSDQVLFNGGSTDNTVR.N
 254 - 273 2130.2659 2129.2586 2129.0501 0.2085 0 K.GLLHSDQVLFNGGSTDNTVR.N
 293 - 307 1572.9630 1571.9557 1571.8089 0.1468 0 K.MGNISPLTGTQGQIR.L
 293 - 307 1572.9630 1571.9557 1571.8089 0.1468 0 K.MGNISPLTGTQGQIR.L
 293 - 307 1588.9489 1587.9416 1587.8038 0.1378 0 K.MGNISPLTGTQGQIR.L Oxidation (M)

## Slide 67
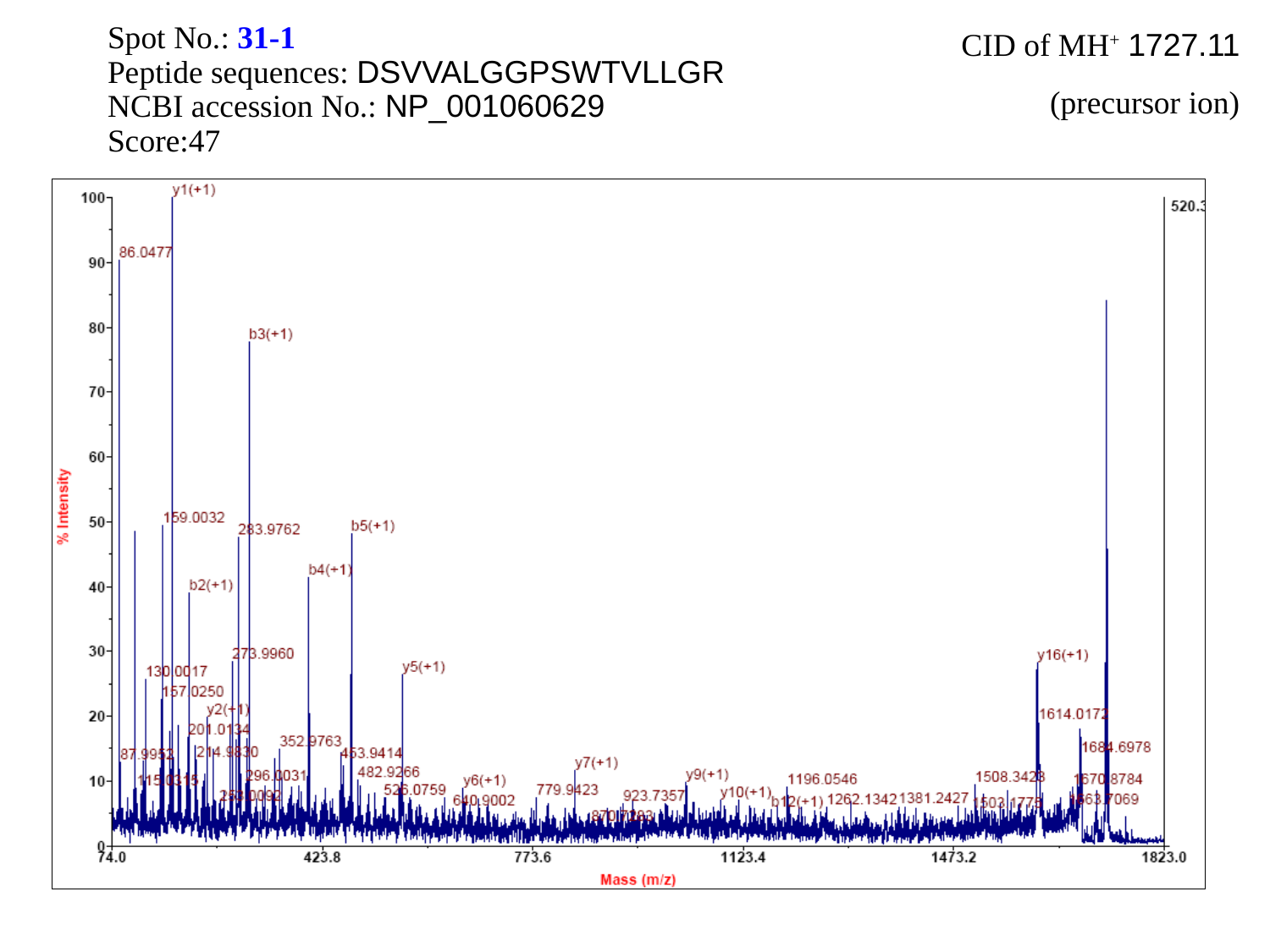

Spot No.: 31-1
Peptide sequences: DSVVALGGPSWTVLLGR
NCBI accession No.: NP_001060629
Score:47
CID of MH+ 1727.11
(precursor ion)

## Slide 68
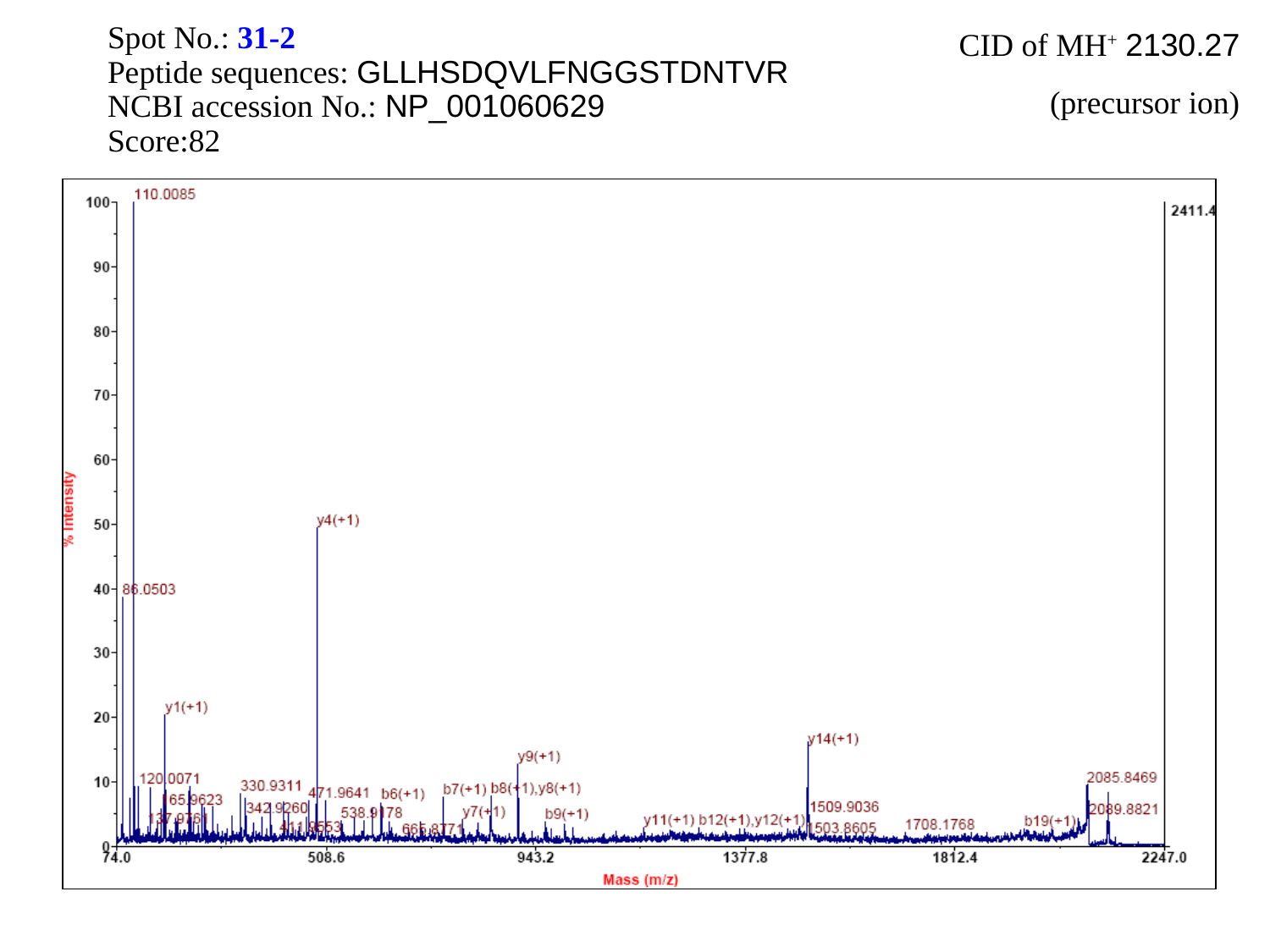

Spot No.: 31-2
Peptide sequences: GLLHSDQVLFNGGSTDNTVR
NCBI accession No.: NP_001060629
Score:82
CID of MH+ 2130.27
(precursor ion)

## Slide 69
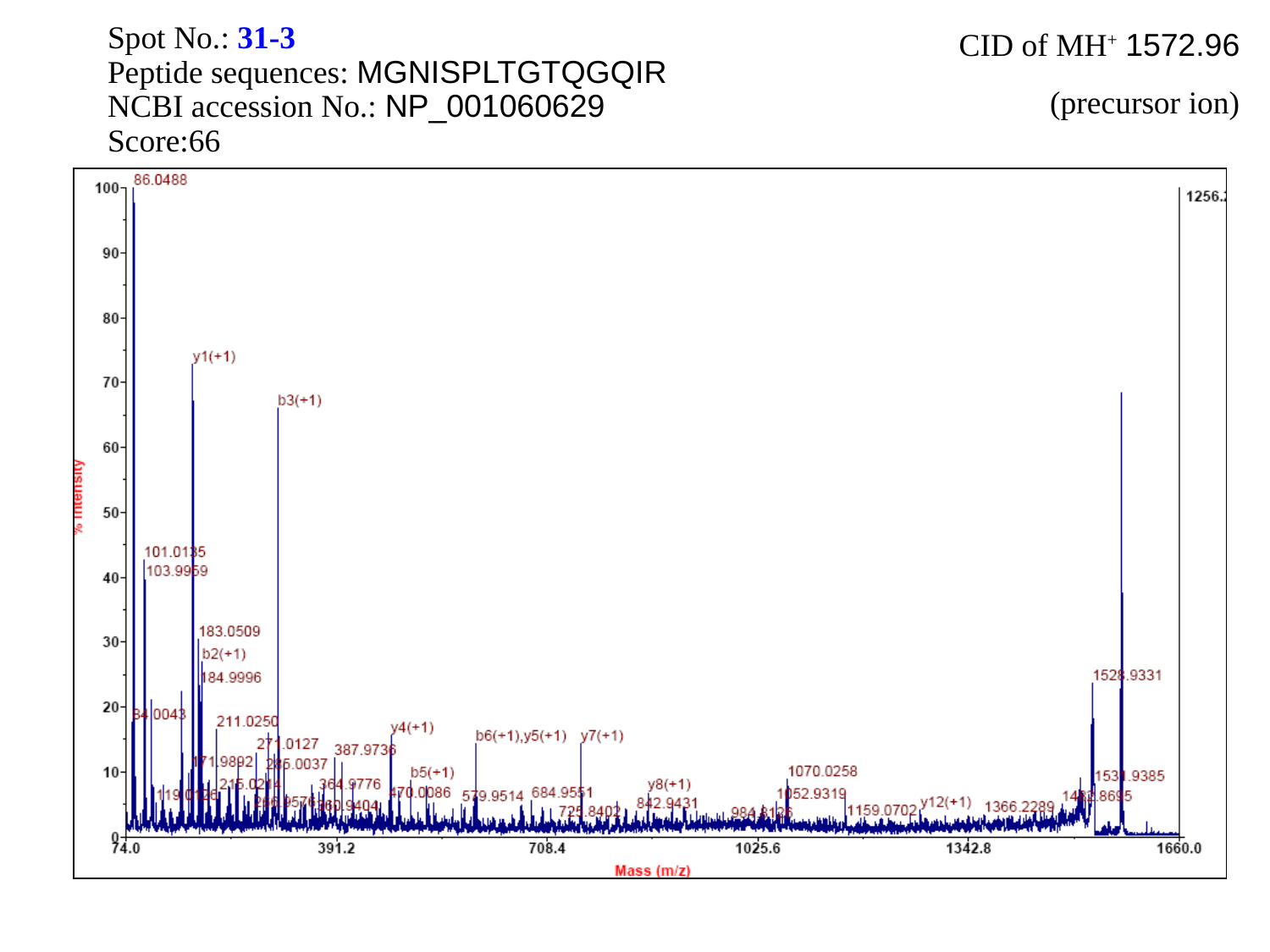

Spot No.: 31-3
Peptide sequences: MGNISPLTGTQGQIR
NCBI accession No.: NP_001060629
Score:66
CID of MH+ 1572.96
(precursor ion)

## Slide 70
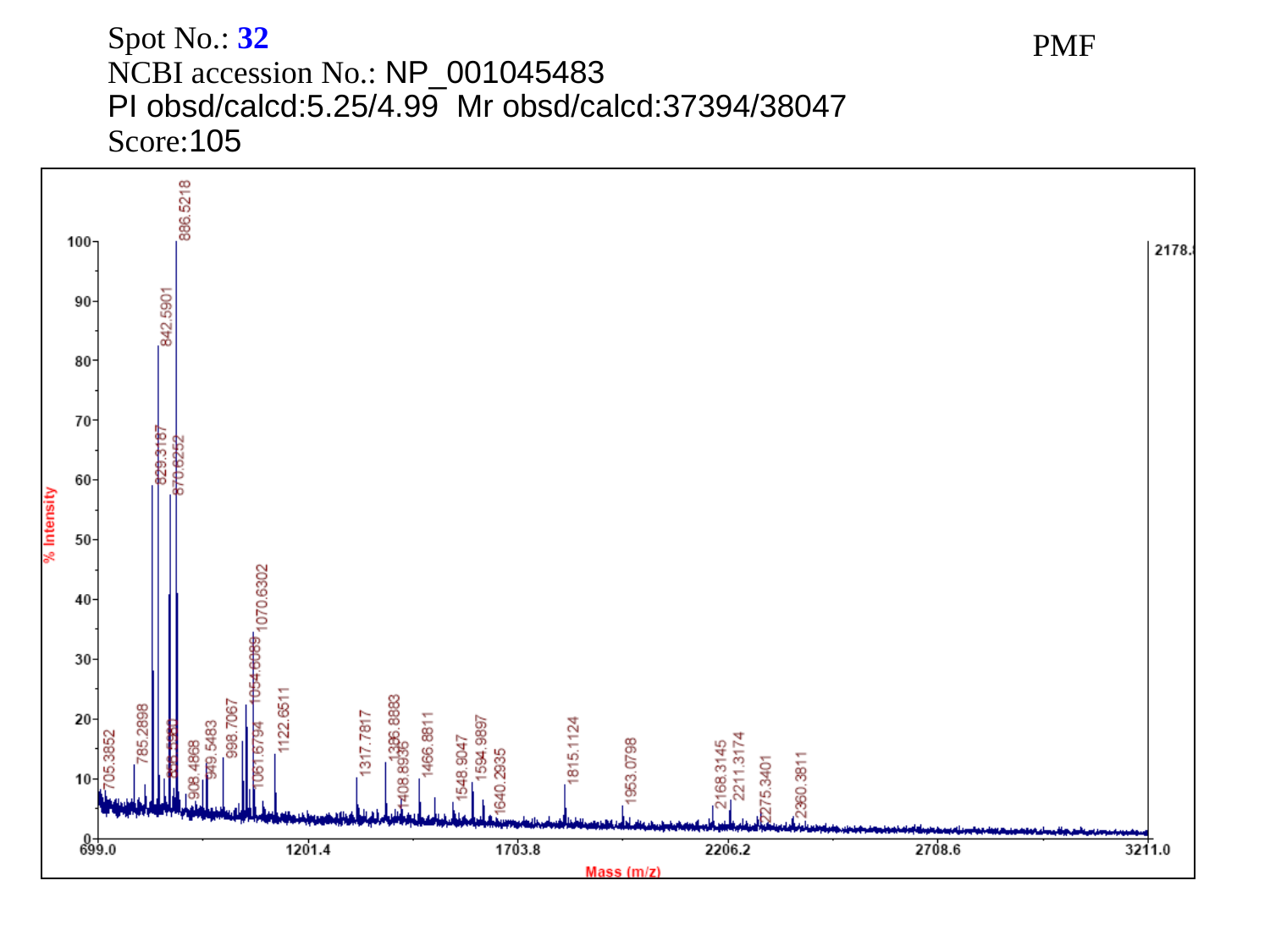

Spot No.: 32
NCBI accession No.: NP_001045483
PI obsd/calcd:5.25/4.99 Mr obsd/calcd:37394/38047
Score:105
PMF

## Slide 71
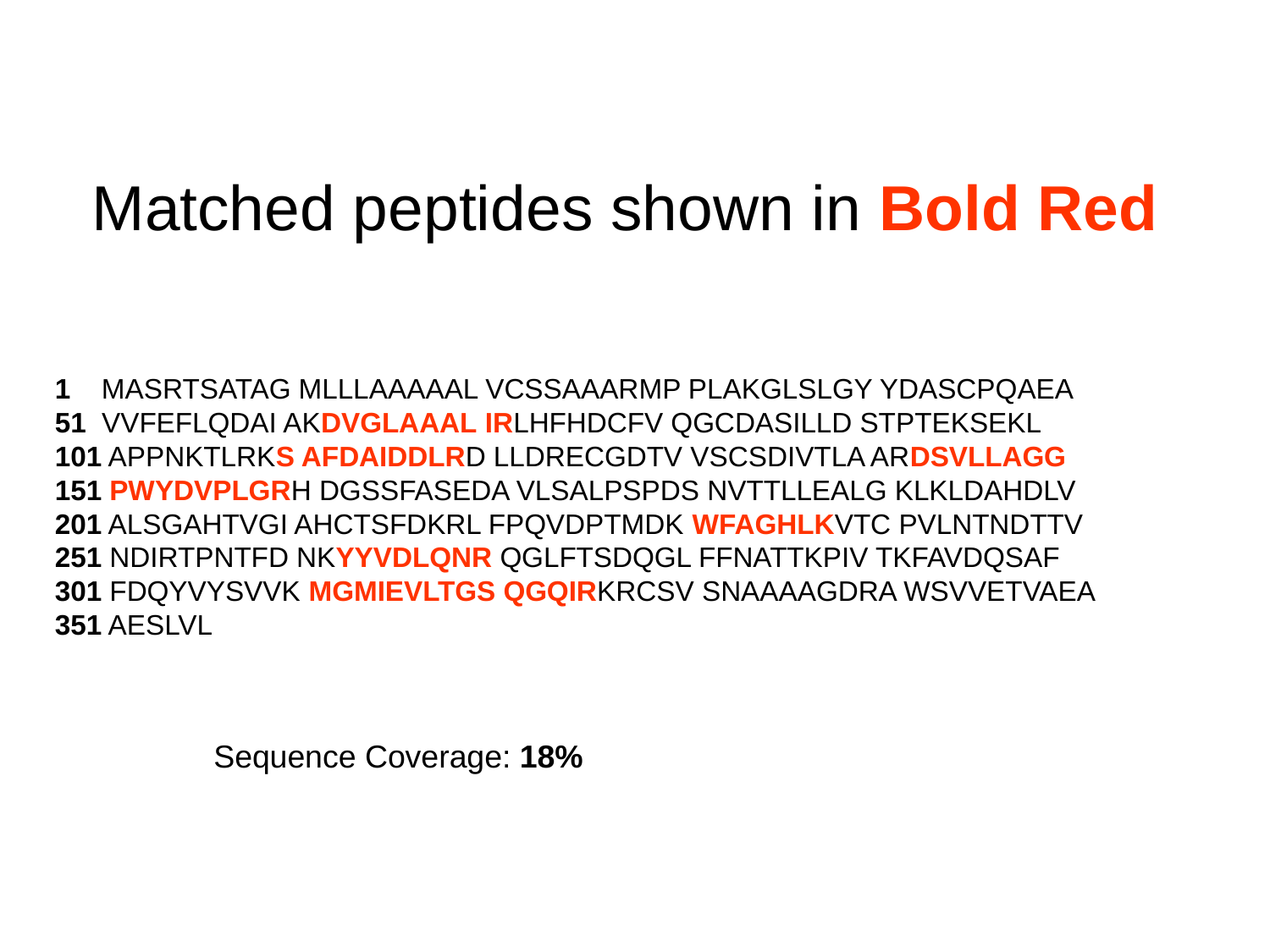

#
Matched peptides shown in Bold Red
1 MASRTSATAG MLLLAAAAAL VCSSAAARMP PLAKGLSLGY YDASCPQAEA
51 VVFEFLQDAI AKDVGLAAAL IRLHFHDCFV QGCDASILLD STPTEKSEKL
101 APPNKTLRKS AFDAIDDLRD LLDRECGDTV VSCSDIVTLA ARDSVLLAGG
151 PWYDVPLGRH DGSSFASEDA VLSALPSPDS NVTTLLEALG KLKLDAHDLV
201 ALSGAHTVGI AHCTSFDKRL FPQVDPTMDK WFAGHLKVTC PVLNTNDTTV
251 NDIRTPNTFD NKYYVDLQNR QGLFTSDQGL FFNATTKPIV TKFAVDQSAF
301 FDQYVYSVVK MGMIEVLTGS QGQIRKRCSV SNAAAAGDRA WSVVETVAEA
351 AESLVL
Sequence Coverage: 18%

## Slide 72
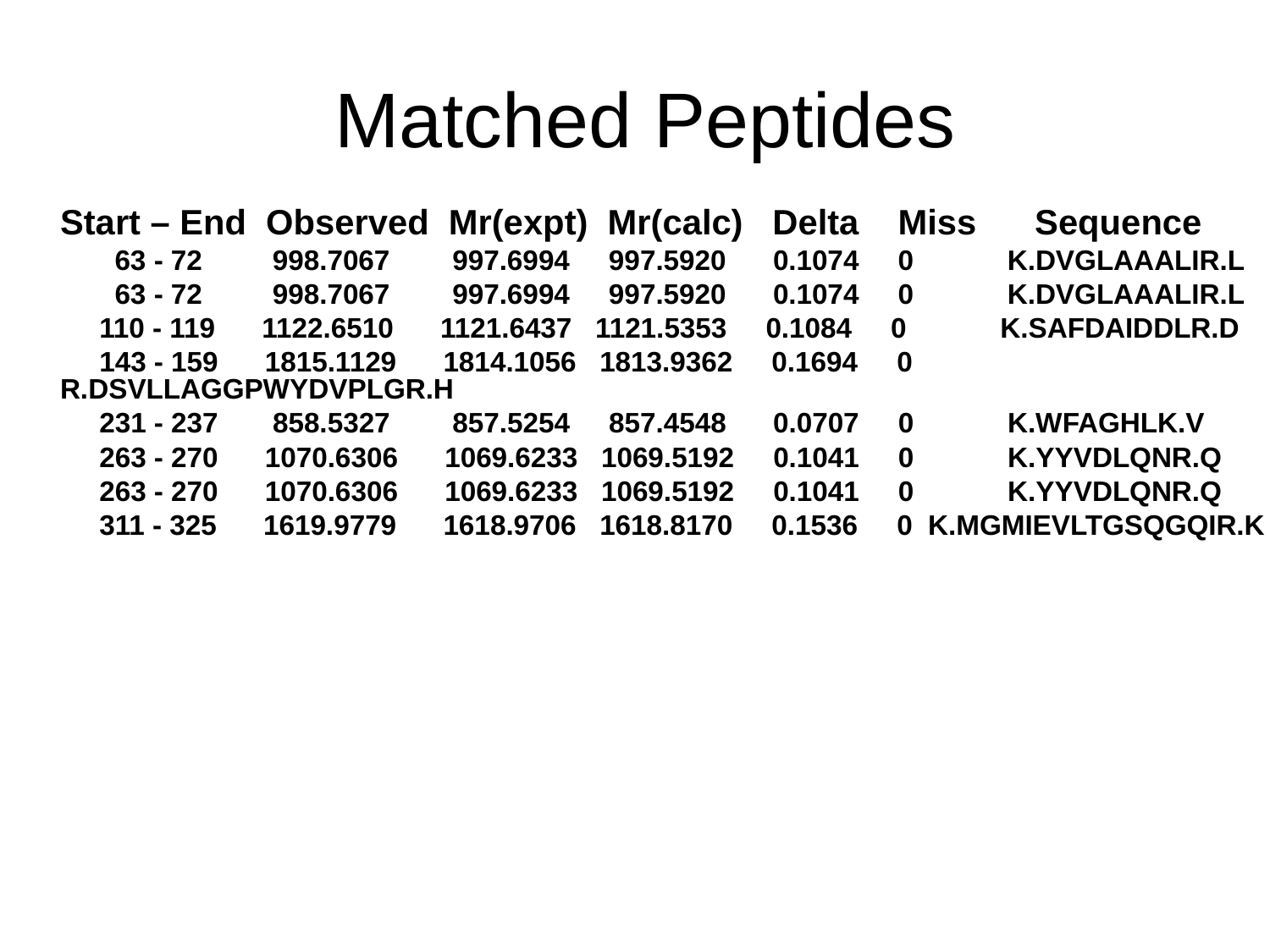

# Matched Peptides
Start – End Observed Mr(expt) Mr(calc) Delta Miss Sequence
 63 - 72 998.7067 997.6994 997.5920 0.1074 0 K.DVGLAAALIR.L
 63 - 72 998.7067 997.6994 997.5920 0.1074 0 K.DVGLAAALIR.L
 110 - 119 1122.6510 1121.6437 1121.5353 0.1084 0 K.SAFDAIDDLR.D
 143 - 159 1815.1129 1814.1056 1813.9362 0.1694 0 R.DSVLLAGGPWYDVPLGR.H
 231 - 237 858.5327 857.5254 857.4548 0.0707 0 K.WFAGHLK.V
 263 - 270 1070.6306 1069.6233 1069.5192 0.1041 0 K.YYVDLQNR.Q
 263 - 270 1070.6306 1069.6233 1069.5192 0.1041 0 K.YYVDLQNR.Q
 311 - 325 1619.9779 1618.9706 1618.8170 0.1536 0 K.MGMIEVLTGSQGQIR.K

## Slide 73
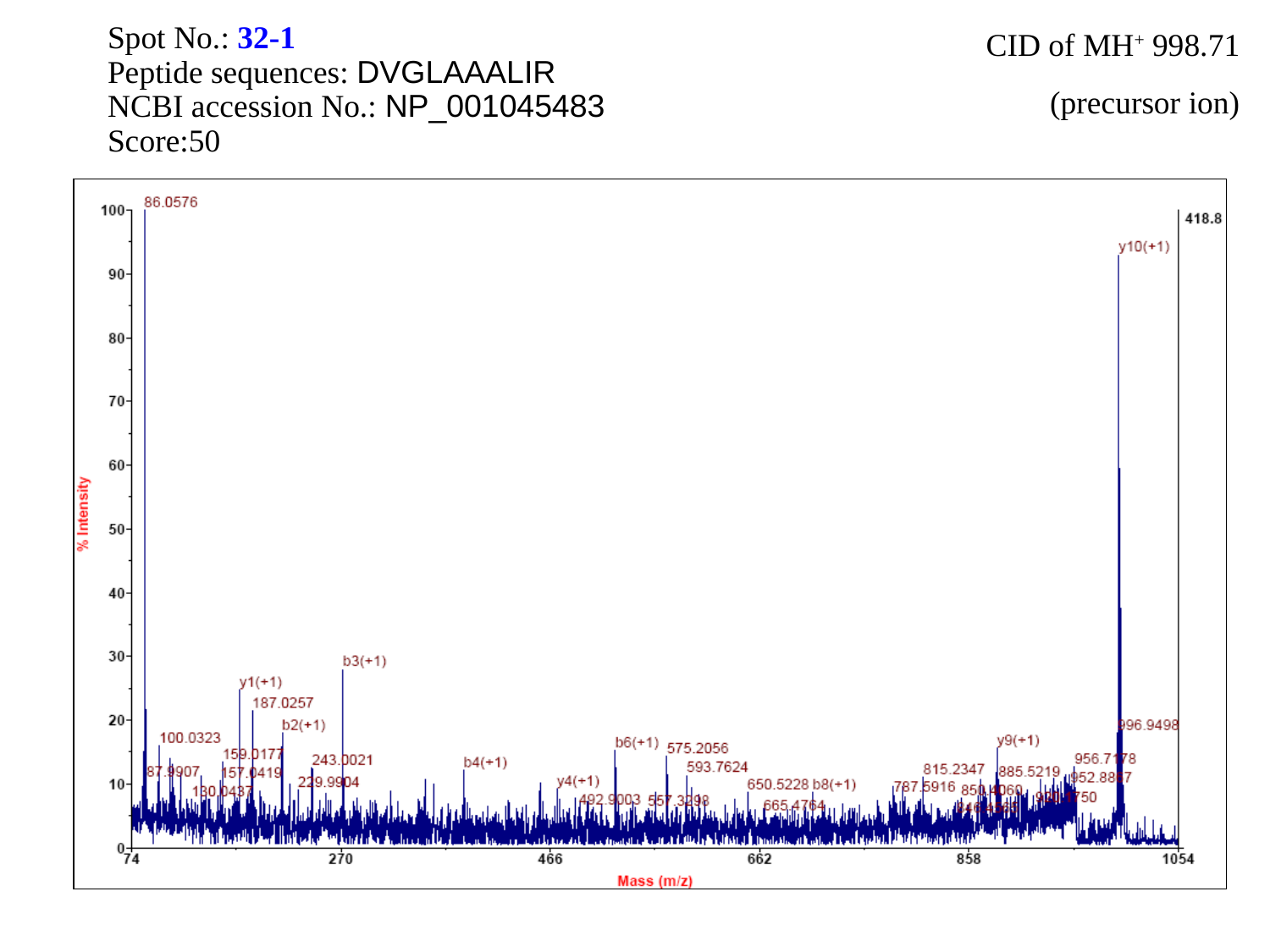

Spot No.: 32-1
Peptide sequences: DVGLAAALIR
NCBI accession No.: NP_001045483
Score:50
CID of MH+ 998.71
(precursor ion)

## Slide 74
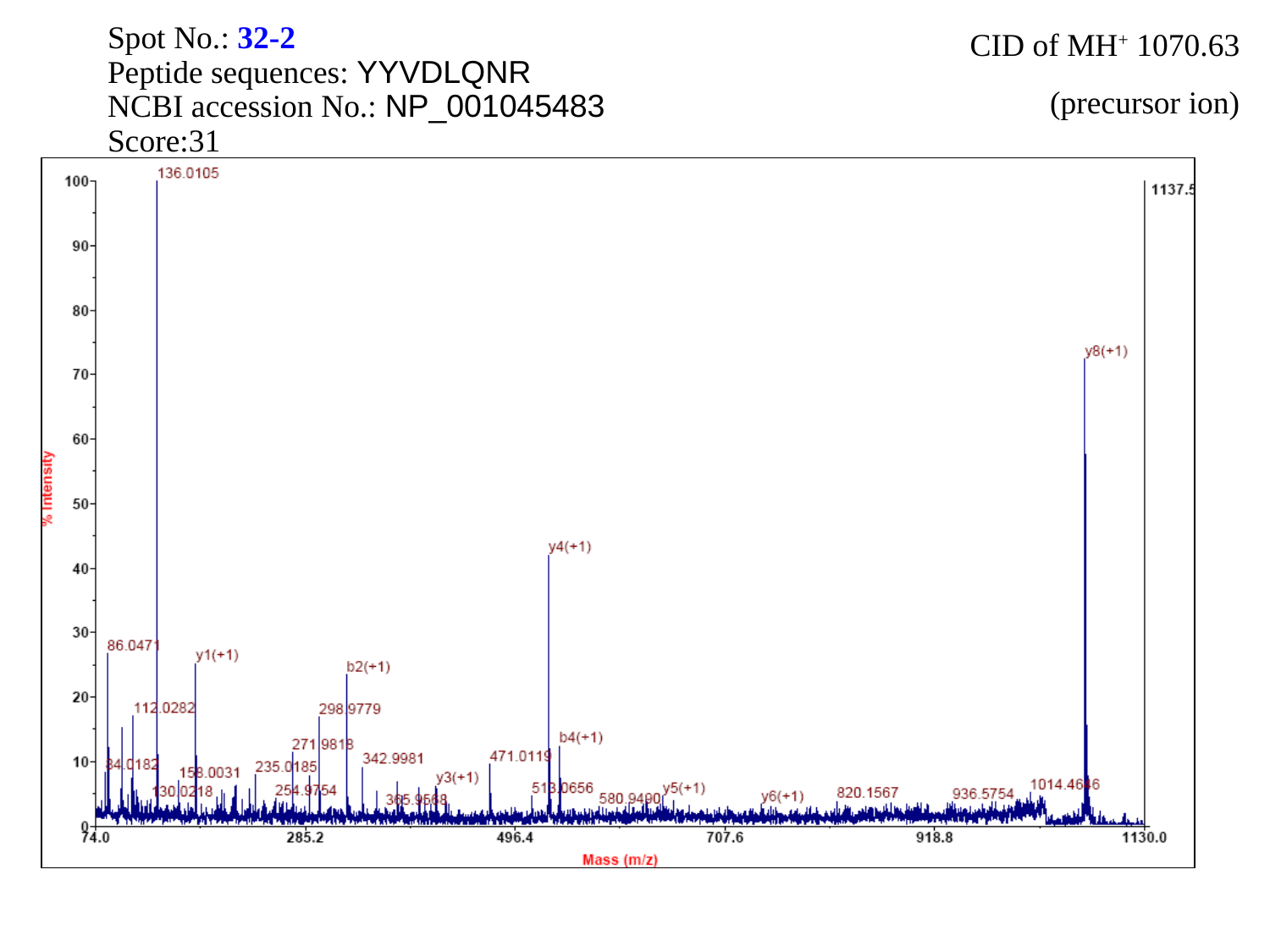

Spot No.: 32-2
Peptide sequences: YYVDLQNR
NCBI accession No.: NP_001045483
Score:31
CID of MH+ 1070.63
(precursor ion)

## Slide 75
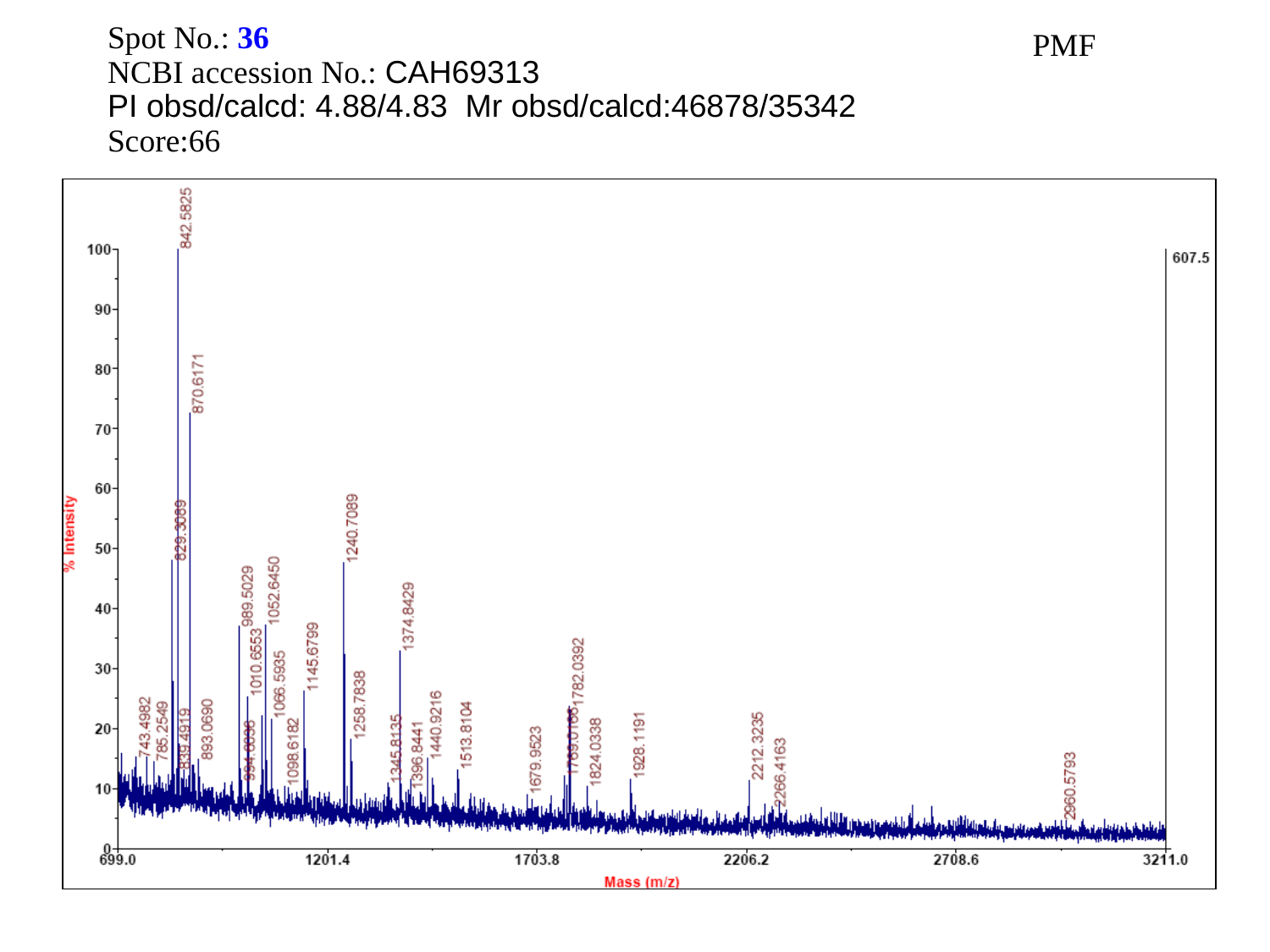

Spot No.: 36
NCBI accession No.: CAH69313
PI obsd/calcd: 4.88/4.83 Mr obsd/calcd:46878/35342
Score:66
PMF

## Slide 76
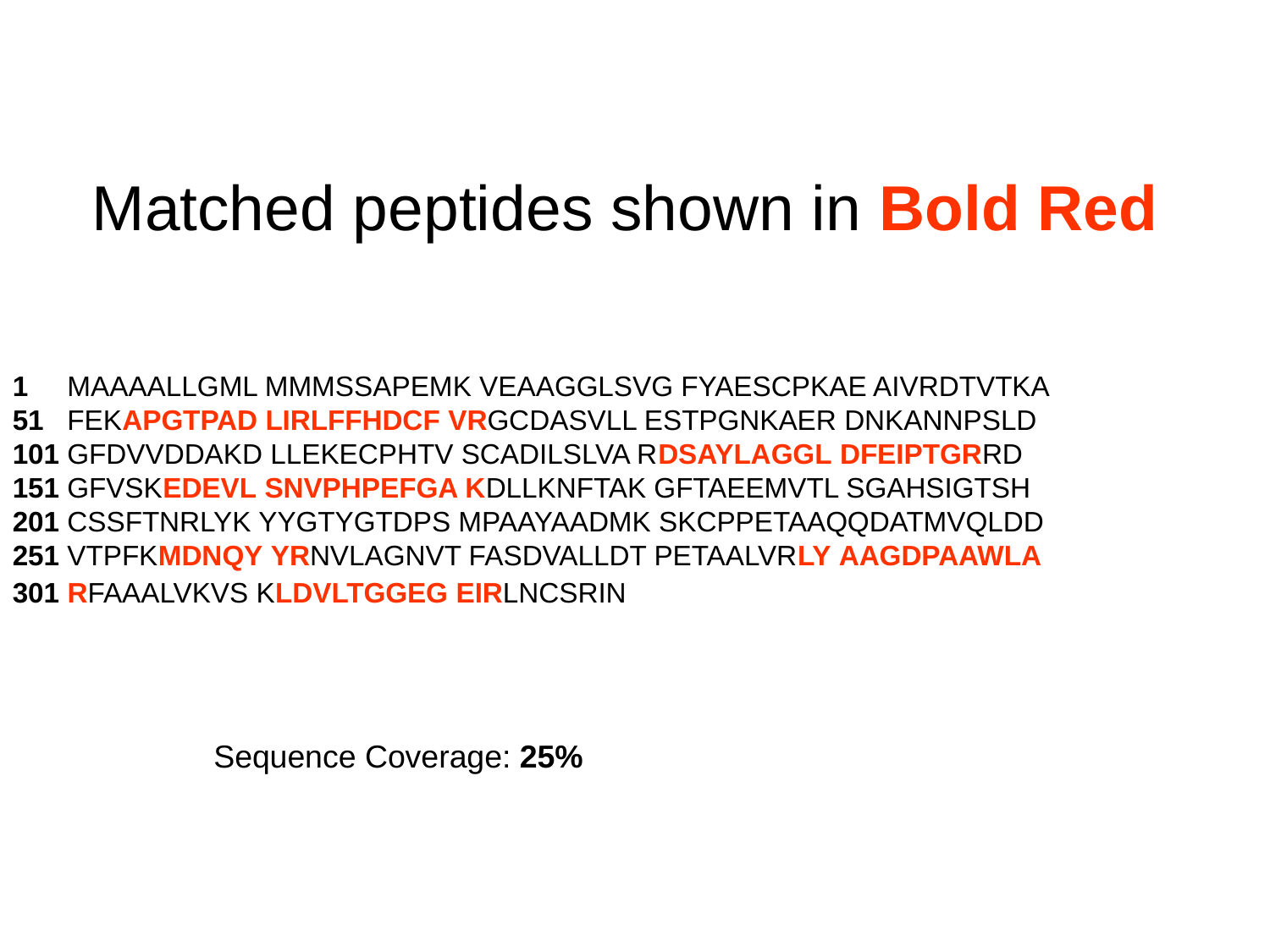

#
Matched peptides shown in Bold Red
1 MAAAALLGML MMMSSAPEMK VEAAGGLSVG FYAESCPKAE AIVRDTVTKA
51 FEKAPGTPAD LIRLFFHDCF VRGCDASVLL ESTPGNKAER DNKANNPSLD
101 GFDVVDDAKD LLEKECPHTV SCADILSLVA RDSAYLAGGL DFEIPTGRRD
151 GFVSKEDEVL SNVPHPEFGA KDLLKNFTAK GFTAEEMVTL SGAHSIGTSH
201 CSSFTNRLYK YYGTYGTDPS MPAAYAADMK SKCPPETAAQQDATMVQLDD
251 VTPFKMDNQY YRNVLAGNVT FASDVALLDT PETAALVRLY AAGDPAAWLA
301 RFAAALVKVS KLDVLTGGEG EIRLNCSRIN
Sequence Coverage: 25%

## Slide 77
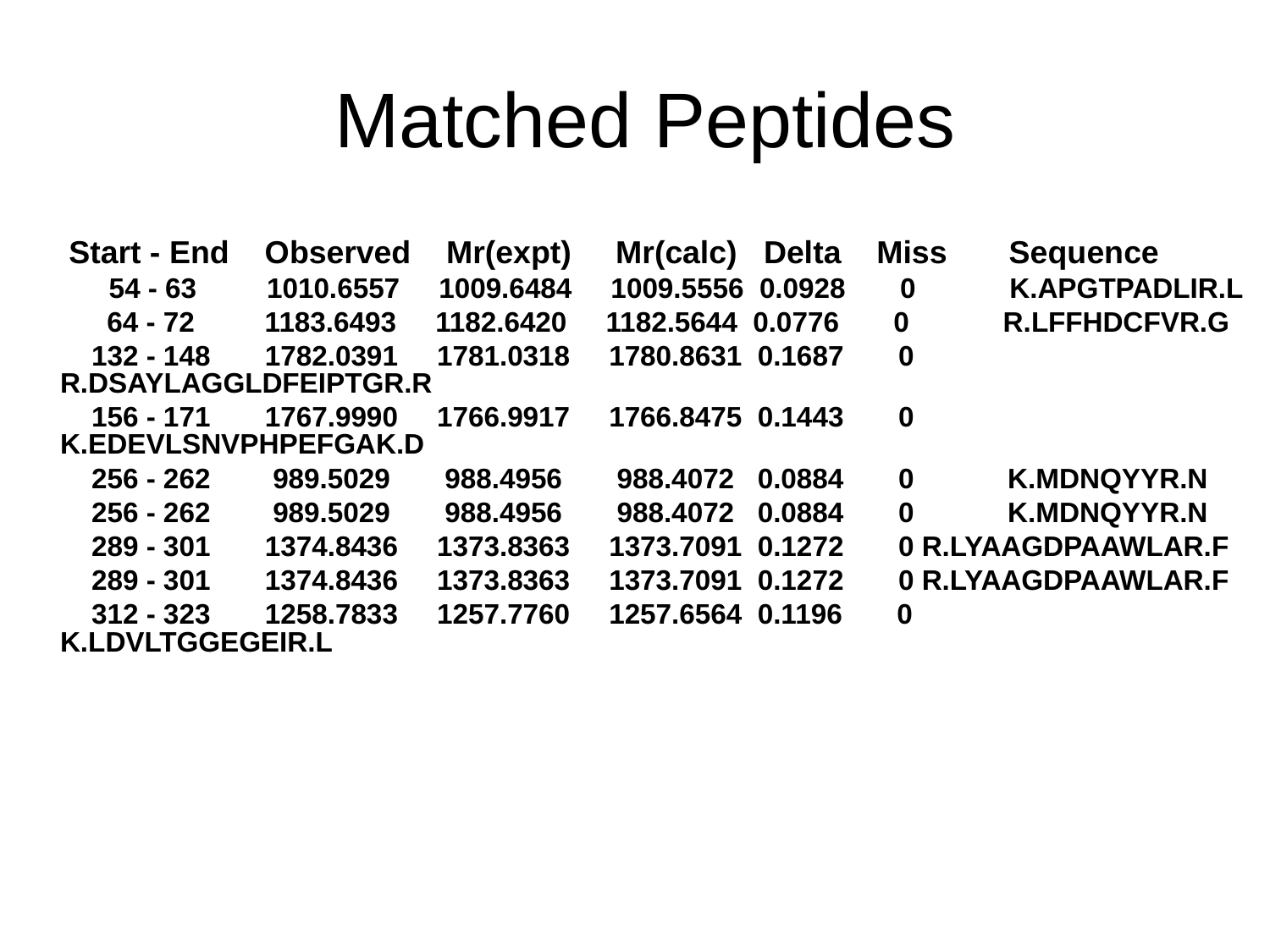

# Matched Peptides
 Start - End Observed Mr(expt) Mr(calc) Delta Miss Sequence
 54 - 63 1010.6557 1009.6484 1009.5556 0.0928 0 K.APGTPADLIR.L
 64 - 72 1183.6493 1182.6420 1182.5644 0.0776 0 R.LFFHDCFVR.G
 132 - 148 1782.0391 1781.0318 1780.8631 0.1687 0 R.DSAYLAGGLDFEIPTGR.R
 156 - 171 1767.9990 1766.9917 1766.8475 0.1443 0 K.EDEVLSNVPHPEFGAK.D
 256 - 262 989.5029 988.4956 988.4072 0.0884 0 K.MDNQYYR.N
 256 - 262 989.5029 988.4956 988.4072 0.0884 0 K.MDNQYYR.N
 289 - 301 1374.8436 1373.8363 1373.7091 0.1272 0 R.LYAAGDPAAWLAR.F
 289 - 301 1374.8436 1373.8363 1373.7091 0.1272 0 R.LYAAGDPAAWLAR.F
 312 - 323 1258.7833 1257.7760 1257.6564 0.1196 0 K.LDVLTGGEGEIR.L

## Slide 78
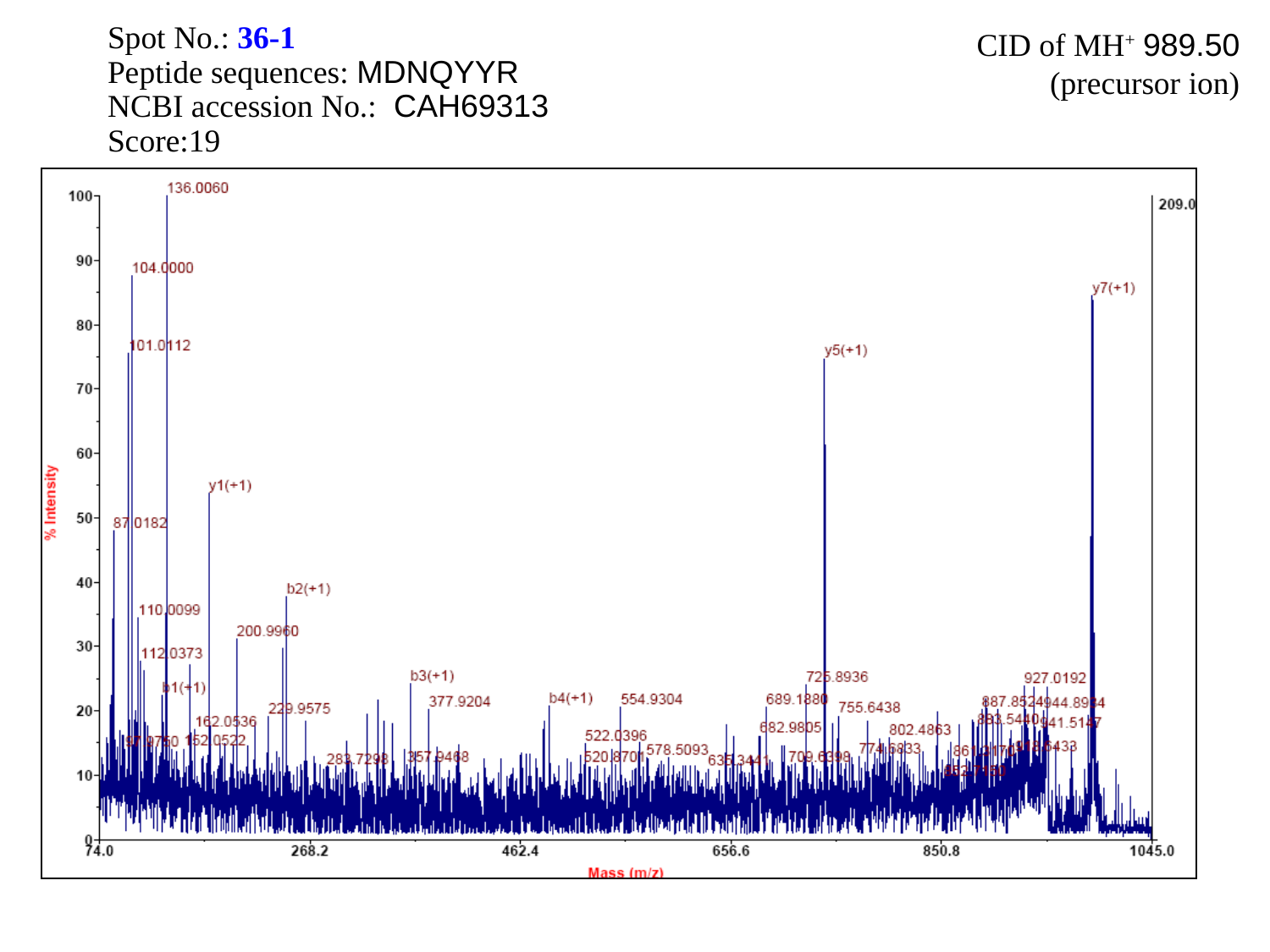

Spot No.: 36-1
Peptide sequences: MDNQYYR
NCBI accession No.: CAH69313
Score:19
CID of MH+ 989.50 (precursor ion)

## Slide 79
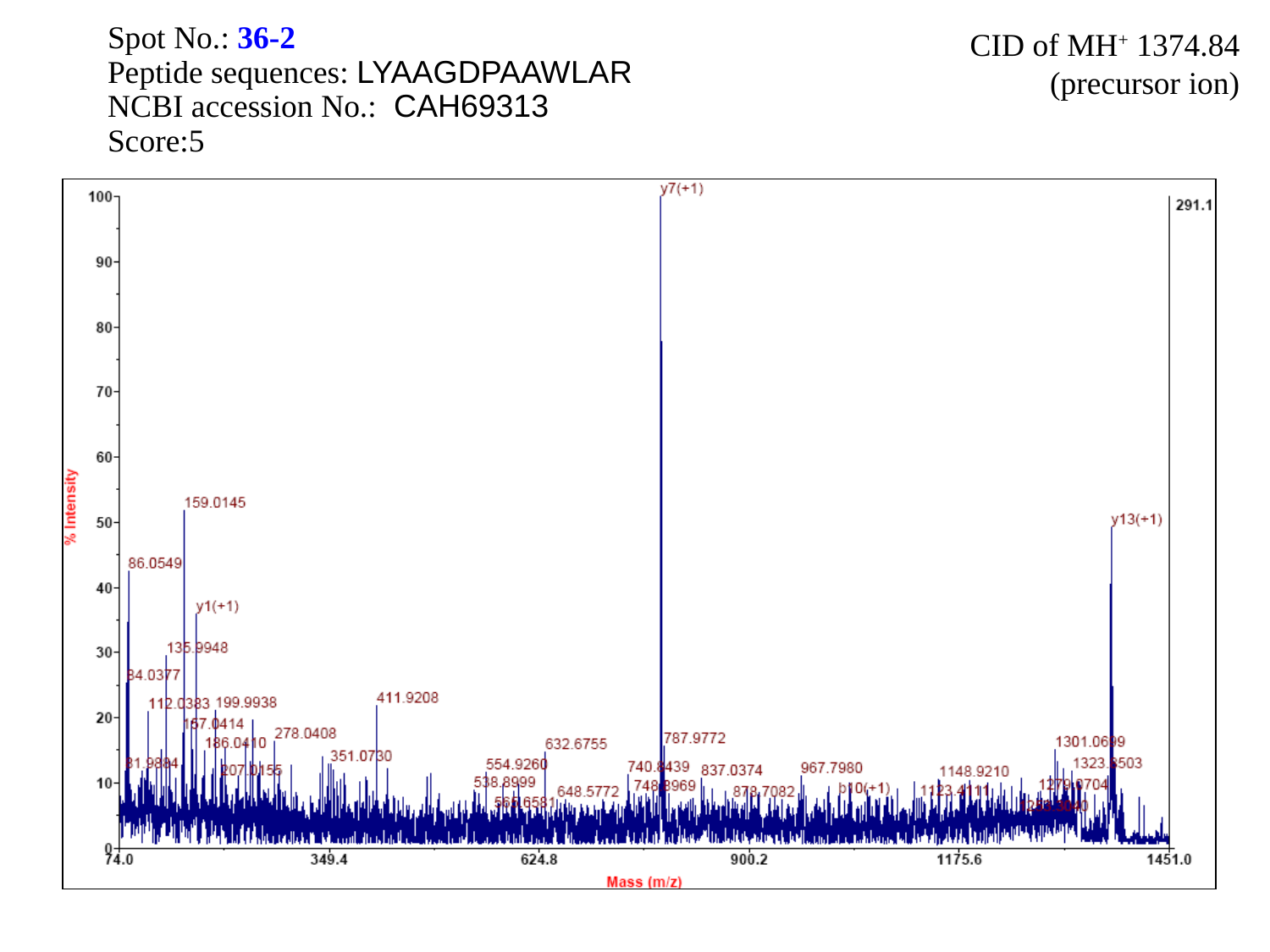

Spot No.: 36-2
Peptide sequences: LYAAGDPAAWLAR
NCBI accession No.: CAH69313
Score:5
CID of MH+ 1374.84 (precursor ion)

## Slide 80
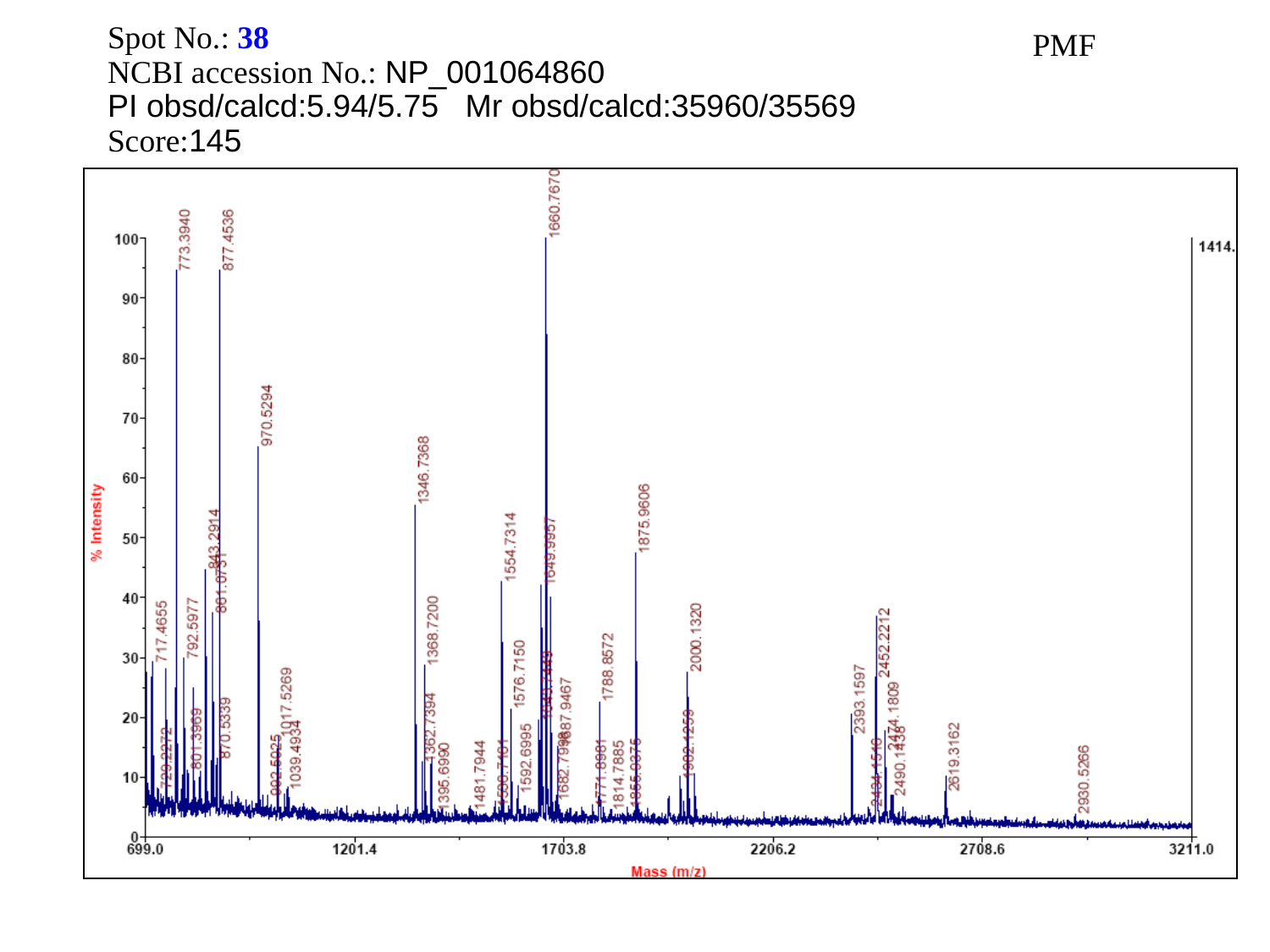

Spot No.: 38
NCBI accession No.: NP_001064860
PI obsd/calcd:5.94/5.75 Mr obsd/calcd:35960/35569
Score:145
PMF

## Slide 81
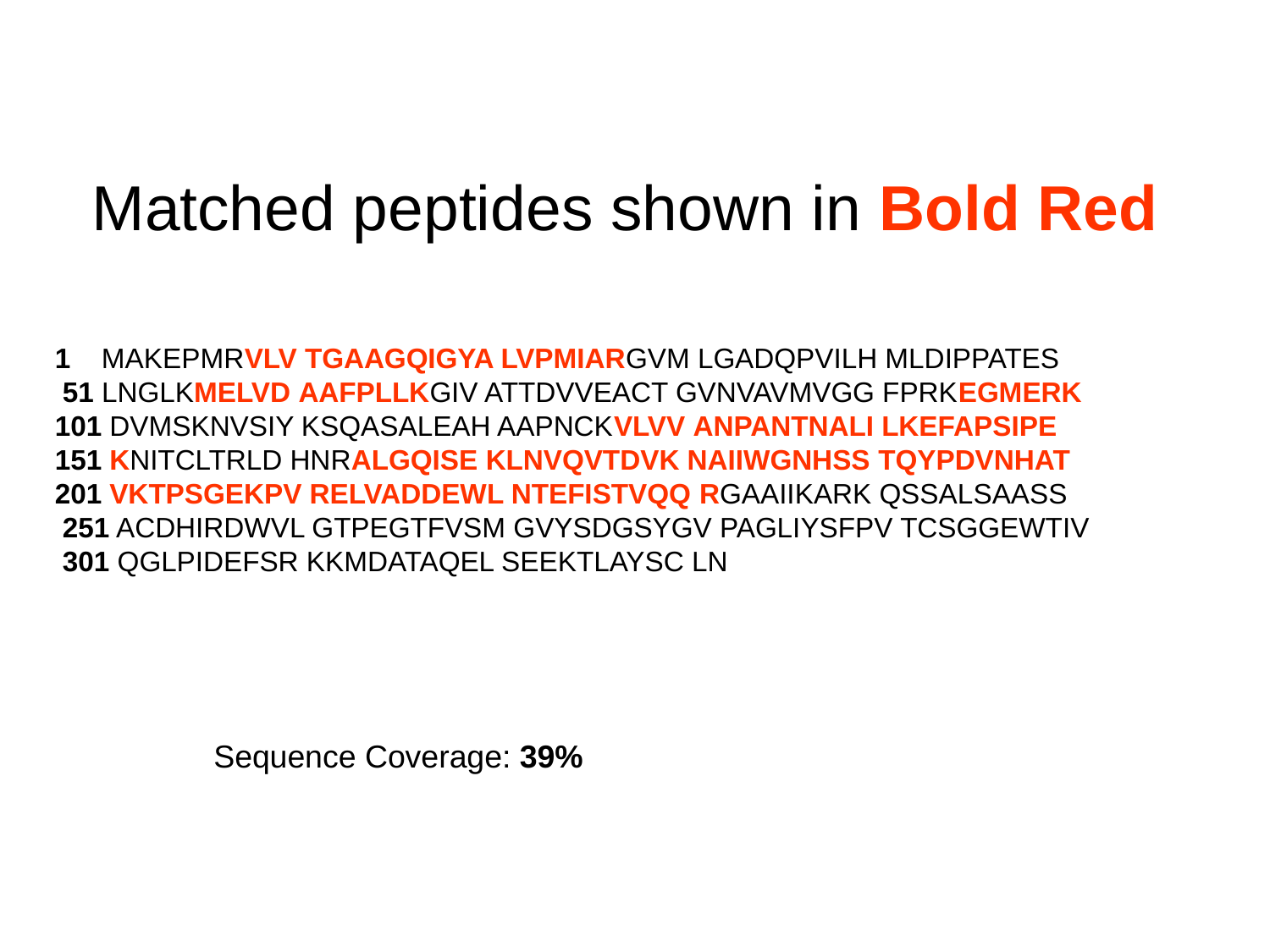

#
Matched peptides shown in Bold Red
1 MAKEPMRVLV TGAAGQIGYA LVPMIARGVM LGADQPVILH MLDIPPATES
 51 LNGLKMELVD AAFPLLKGIV ATTDVVEACT GVNVAVMVGG FPRKEGMERK
101 DVMSKNVSIY KSQASALEAH AAPNCKVLVV ANPANTNALI LKEFAPSIPE
151 KNITCLTRLD HNRALGQISE KLNVQVTDVK NAIIWGNHSS TQYPDVNHAT
201 VKTPSGEKPV RELVADDEWL NTEFISTVQQ RGAAIIKARK QSSALSAASS
 251 ACDHIRDWVL GTPEGTFVSM GVYSDGSYGV PAGLIYSFPV TCSGGEWTIV
 301 QGLPIDEFSR KKMDATAQEL SEEKTLAYSC LN
Sequence Coverage: 39%

## Slide 82
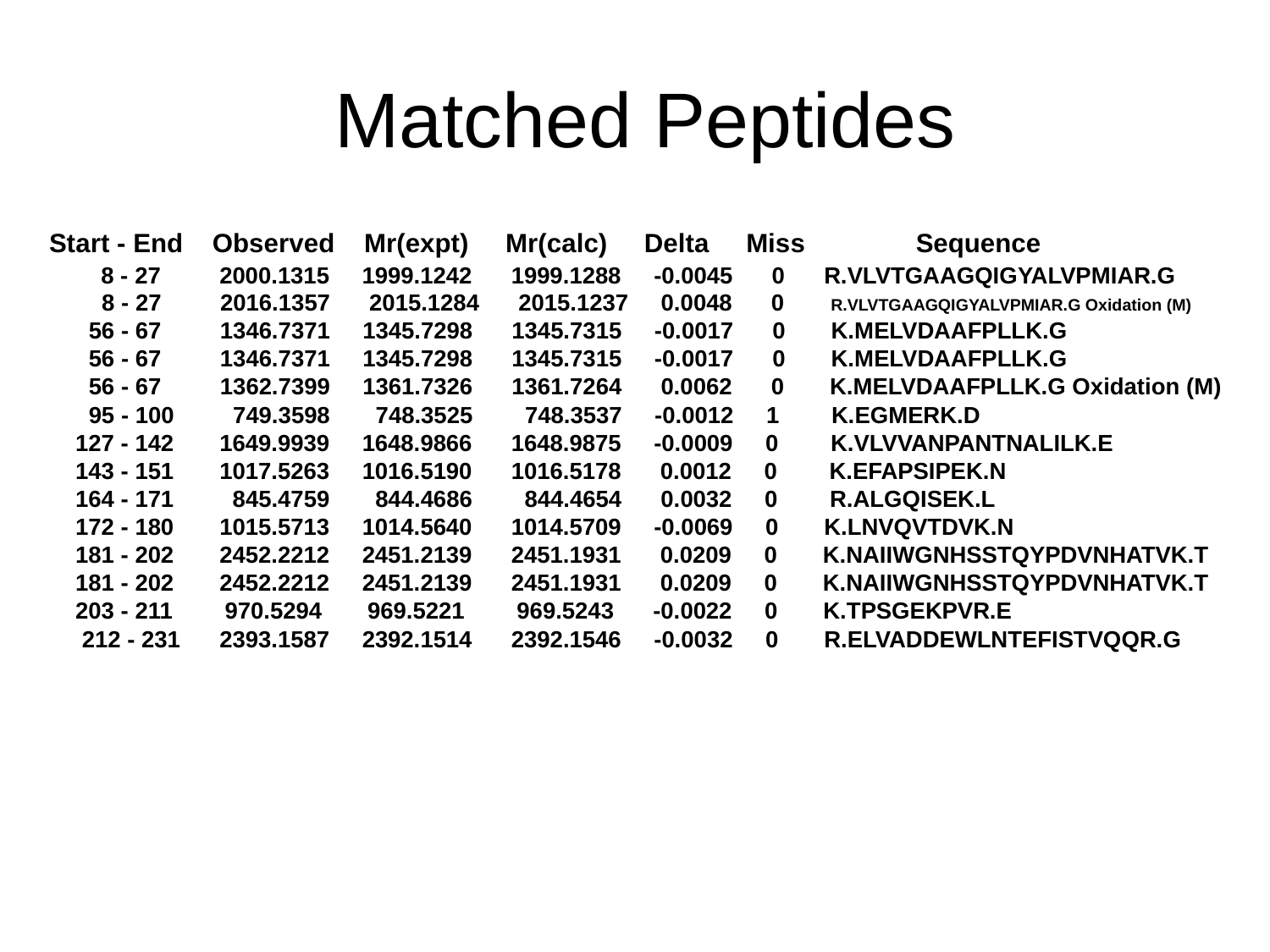

# Matched Peptides
Start - End Observed Mr(expt) Mr(calc) Delta Miss Sequence
 8 - 27 2000.1315 1999.1242 1999.1288 -0.0045 0 R.VLVTGAAGQIGYALVPMIAR.G
 8 - 27 2016.1357 2015.1284 2015.1237 0.0048 0 R.VLVTGAAGQIGYALVPMIAR.G Oxidation (M)
 56 - 67 1346.7371 1345.7298 1345.7315 -0.0017 0 K.MELVDAAFPLLK.G
 56 - 67 1346.7371 1345.7298 1345.7315 -0.0017 0 K.MELVDAAFPLLK.G
 56 - 67 1362.7399 1361.7326 1361.7264 0.0062 0 K.MELVDAAFPLLK.G Oxidation (M)
 95 - 100 749.3598 748.3525 748.3537 -0.0012 1 K.EGMERK.D
 127 - 142 1649.9939 1648.9866 1648.9875 -0.0009 0 K.VLVVANPANTNALILK.E
 143 - 151 1017.5263 1016.5190 1016.5178 0.0012 0 K.EFAPSIPEK.N
 164 - 171 845.4759 844.4686 844.4654 0.0032 0 R.ALGQISEK.L
 172 - 180 1015.5713 1014.5640 1014.5709 -0.0069 0 K.LNVQVTDVK.N
 181 - 202 2452.2212 2451.2139 2451.1931 0.0209 0 K.NAIIWGNHSSTQYPDVNHATVK.T
 181 - 202 2452.2212 2451.2139 2451.1931 0.0209 0 K.NAIIWGNHSSTQYPDVNHATVK.T
 203 - 211 970.5294 969.5221 969.5243 -0.0022 0 K.TPSGEKPVR.E
 212 - 231 2393.1587 2392.1514 2392.1546 -0.0032 0 R.ELVADDEWLNTEFISTVQQR.G

## Slide 83
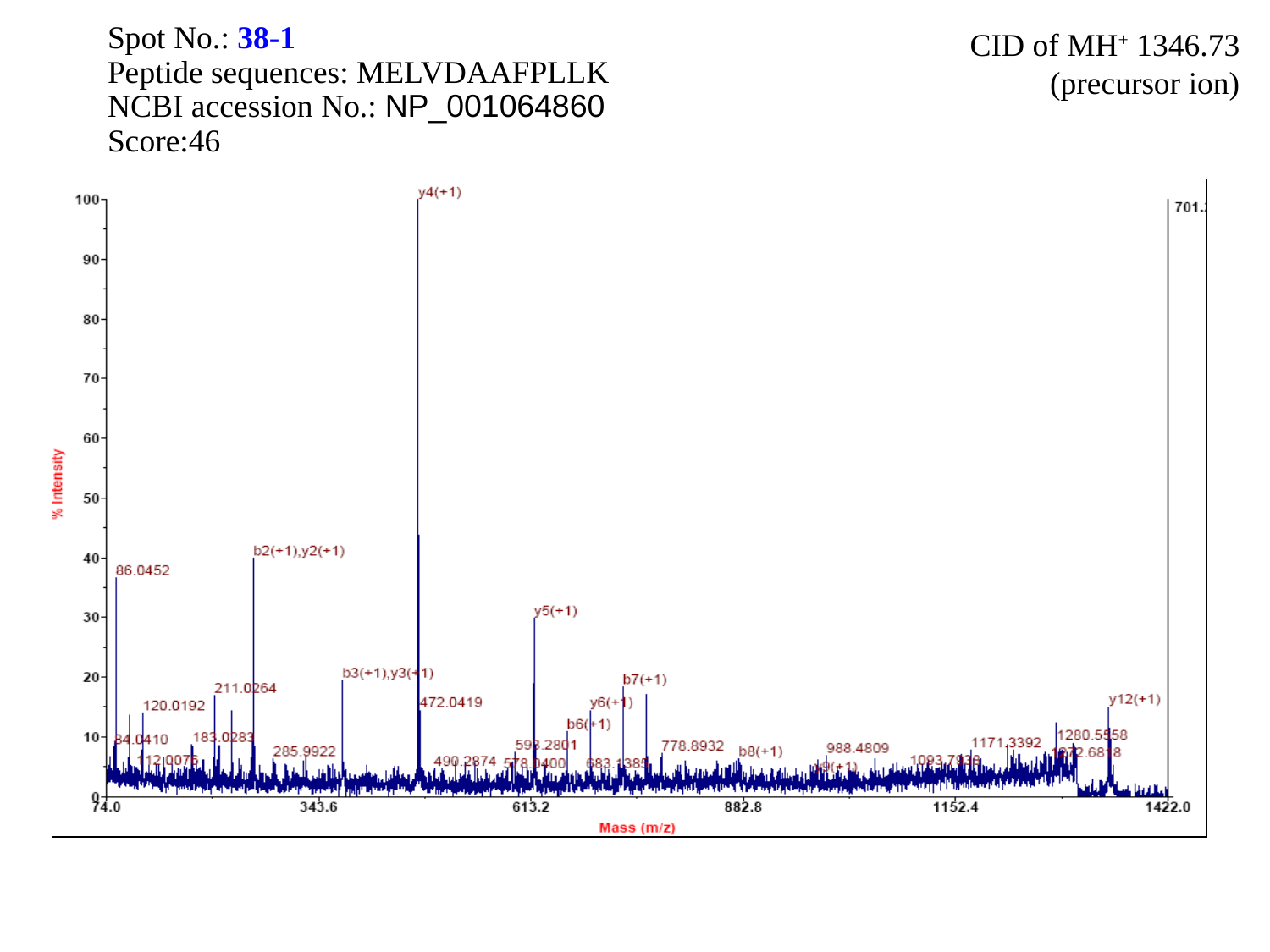

Spot No.: 38-1
Peptide sequences: MELVDAAFPLLK
NCBI accession No.: NP_001064860
Score:46
CID of MH+ 1346.73 (precursor ion)

## Slide 84
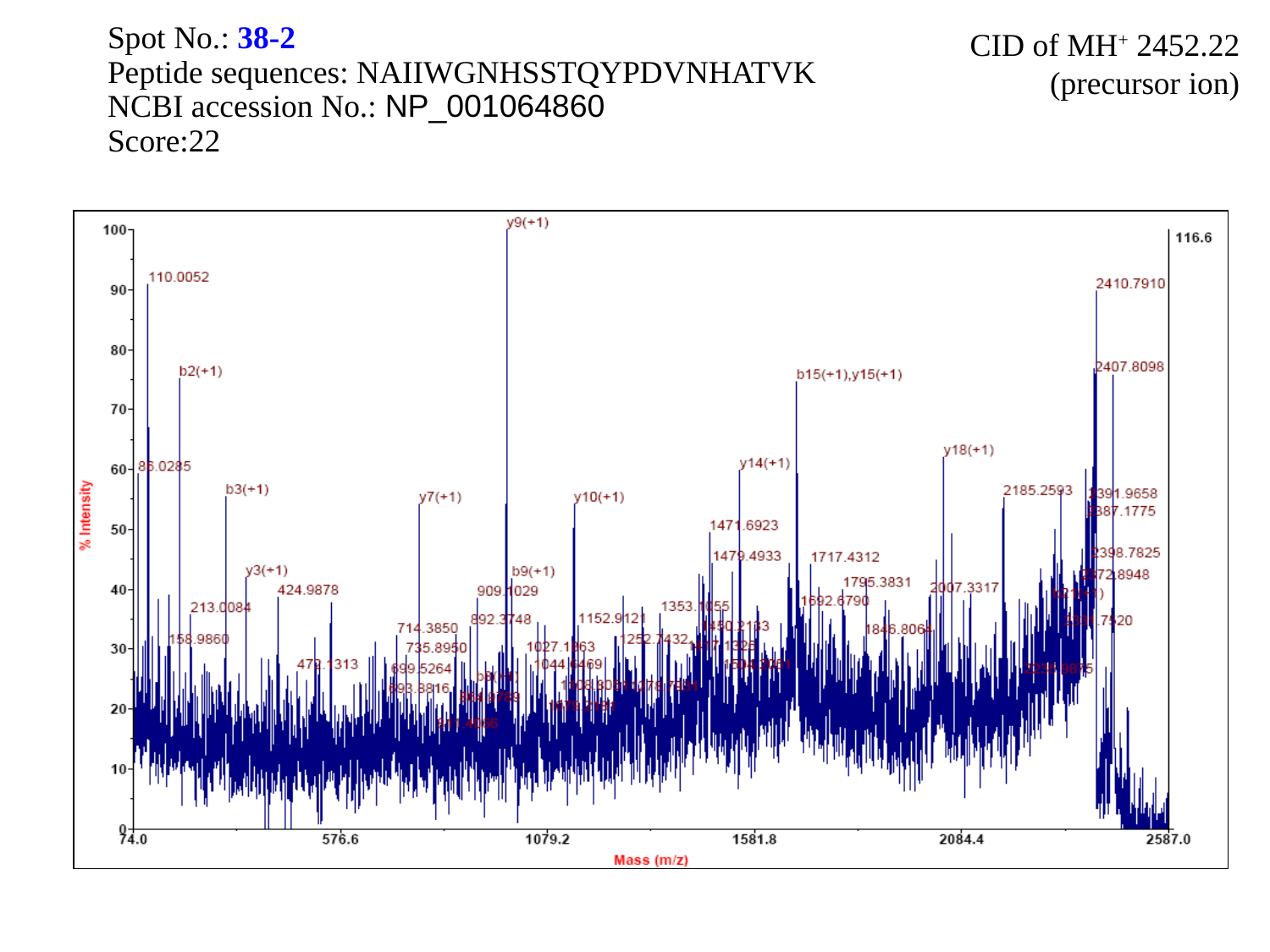

Spot No.: 38-2
Peptide sequences: NAIIWGNHSSTQYPDVNHATVK
NCBI accession No.: NP_001064860
Score:22
CID of MH+ 2452.22 (precursor ion)

## Slide 85
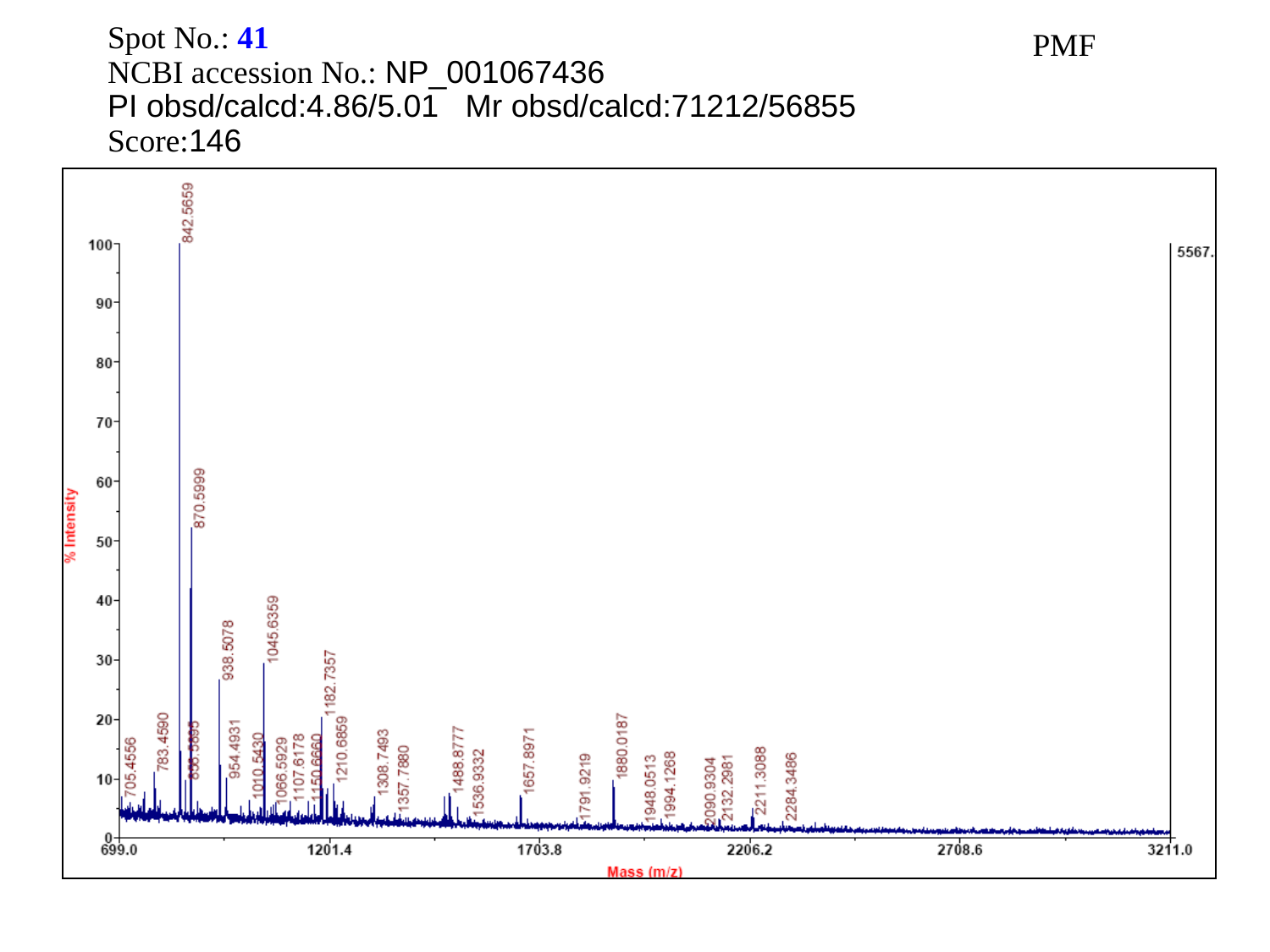

Spot No.: 41
NCBI accession No.: NP_001067436
PI obsd/calcd:4.86/5.01 Mr obsd/calcd:71212/56855
Score:146
PMF

## Slide 86
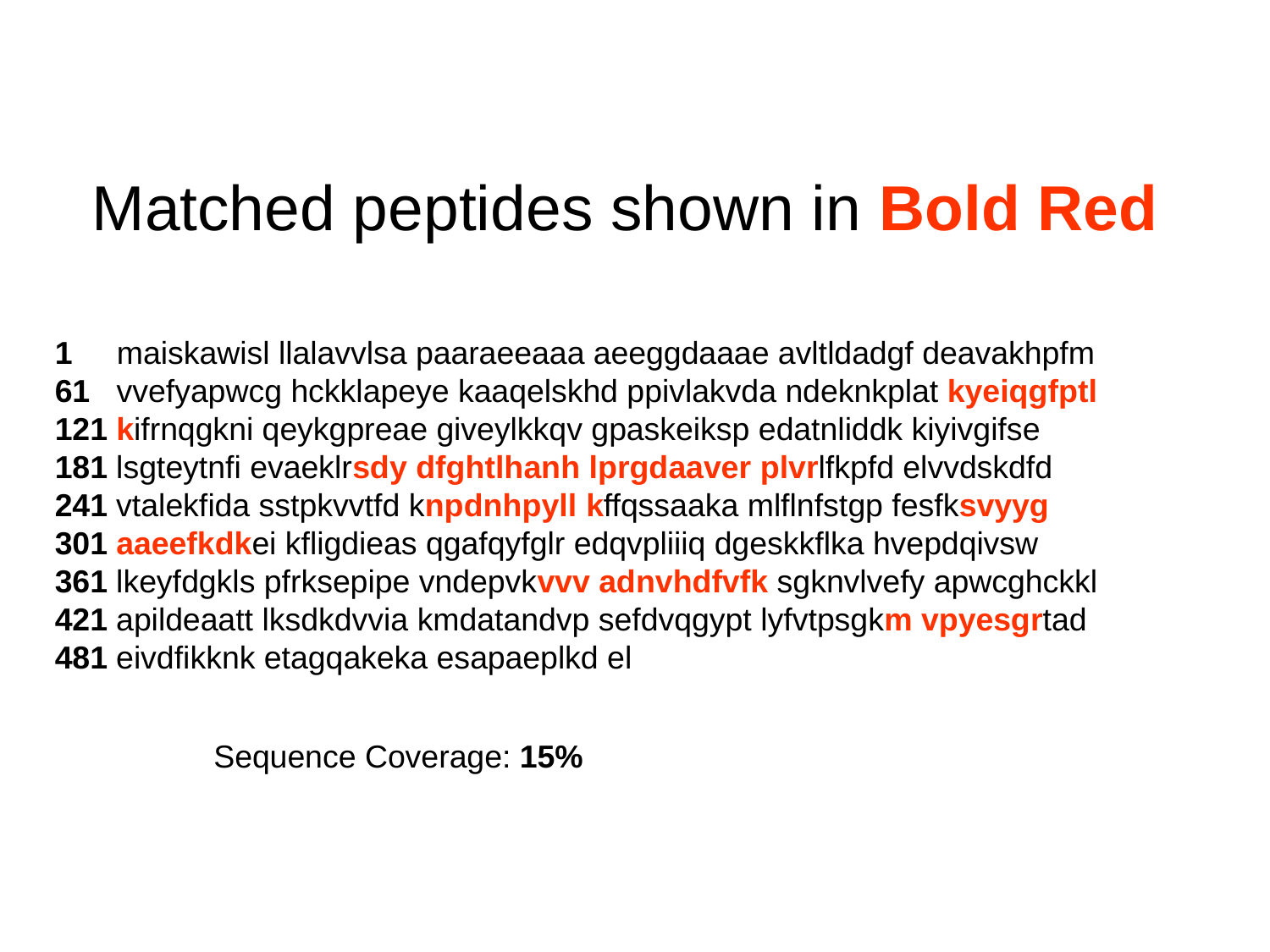

#
Matched peptides shown in Bold Red
1 maiskawisl llalavvlsa paaraeeaaa aeeggdaaae avltldadgf deavakhpfm
61 vvefyapwcg hckklapeye kaaqelskhd ppivlakvda ndeknkplat kyeiqgfptl
121 kifrnqgkni qeykgpreae giveylkkqv gpaskeiksp edatnliddk kiyivgifse
181 lsgteytnfi evaeklrsdy dfghtlhanh lprgdaaver plvrlfkpfd elvvdskdfd
241 vtalekfida sstpkvvtfd knpdnhpyll kffqssaaka mlflnfstgp fesfksvyyg
301 aaeefkdkei kfligdieas qgafqyfglr edqvpliiiq dgeskkflka hvepdqivsw
361 lkeyfdgkls pfrksepipe vndepvkvvv adnvhdfvfk sgknvlvefy apwcghckkl
421 apildeaatt lksdkdvvia kmdatandvp sefdvqgypt lyfvtpsgkm vpyesgrtad
481 eivdfikknk etagqakeka esapaeplkd el
Sequence Coverage: 15%

## Slide 87
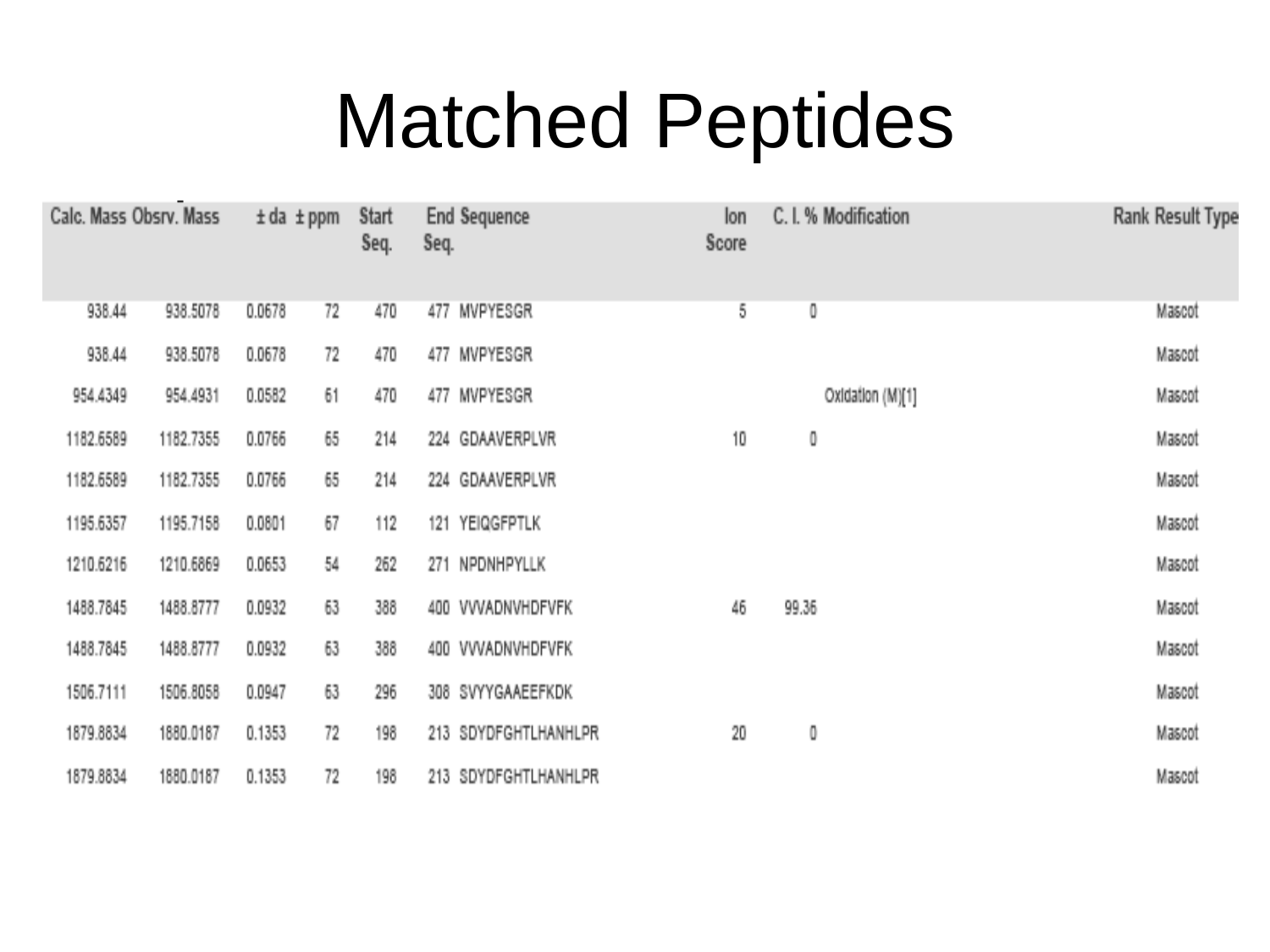

# Matched Peptides

## Slide 88
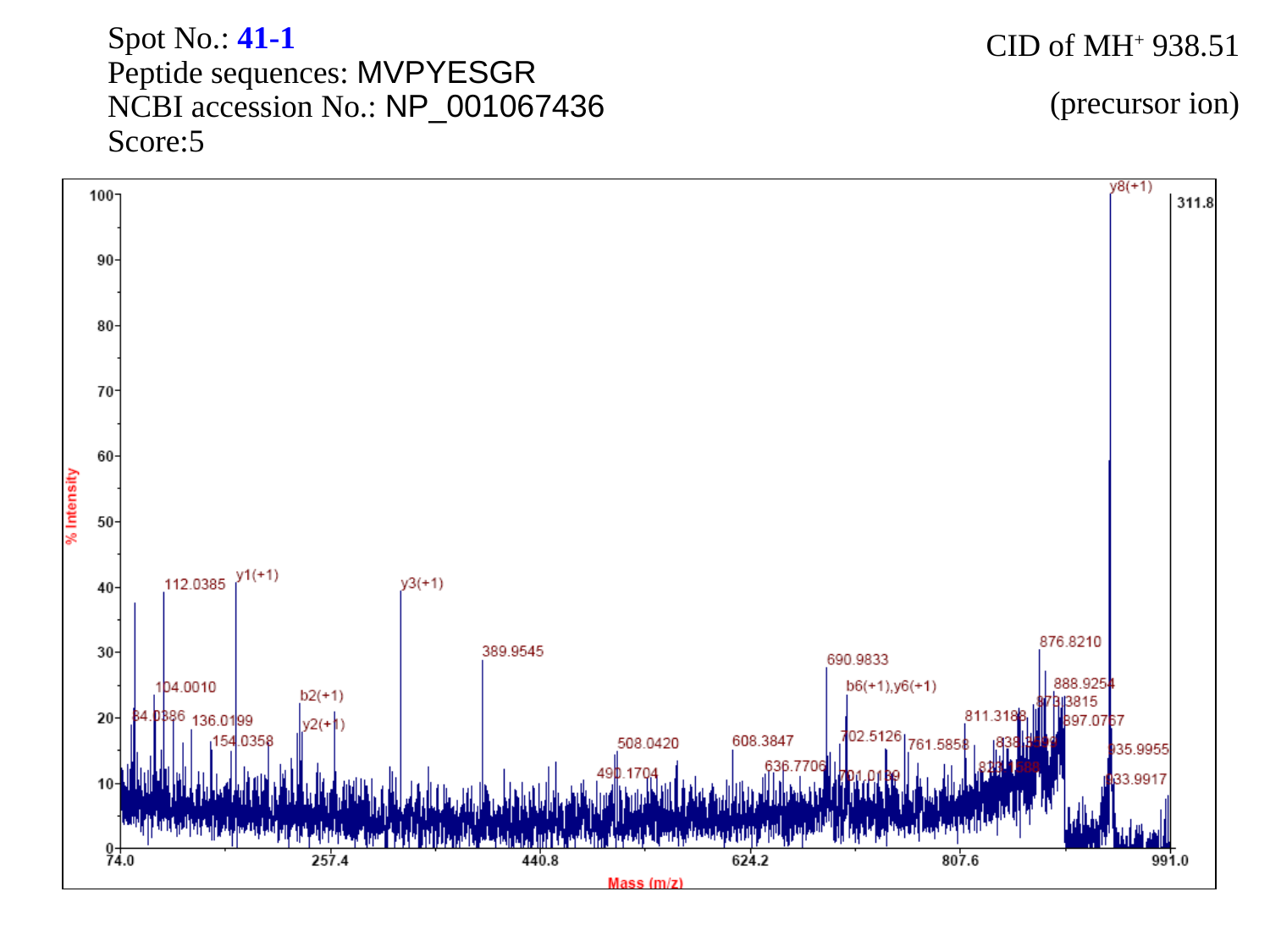

Spot No.: 41-1
Peptide sequences: MVPYESGR
NCBI accession No.: NP_001067436
Score:5
CID of MH+ 938.51
(precursor ion)

## Slide 89
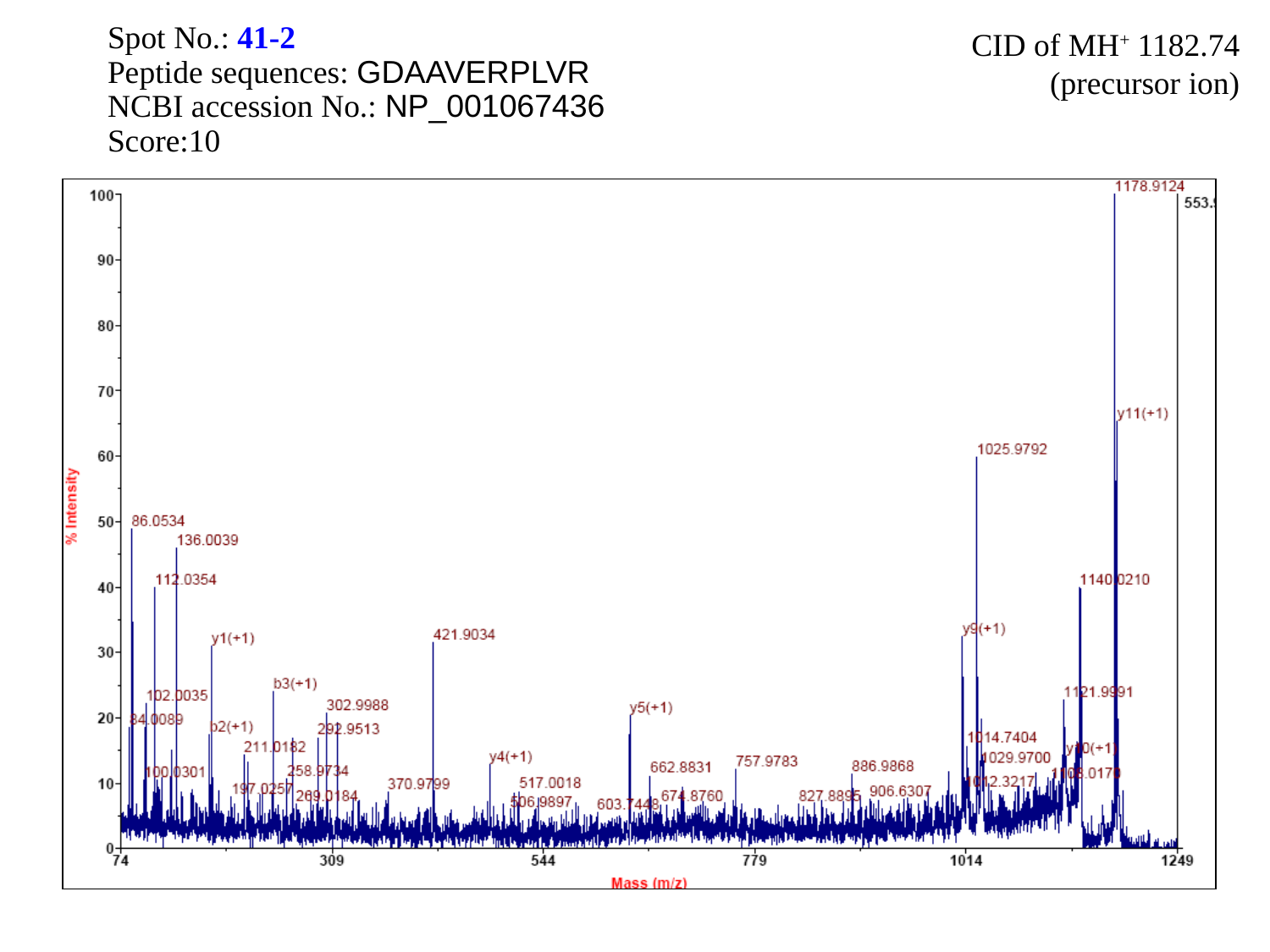

Spot No.: 41-2
Peptide sequences: GDAAVERPLVR
NCBI accession No.: NP_001067436
Score:10
CID of MH+ 1182.74 (precursor ion)

## Slide 90
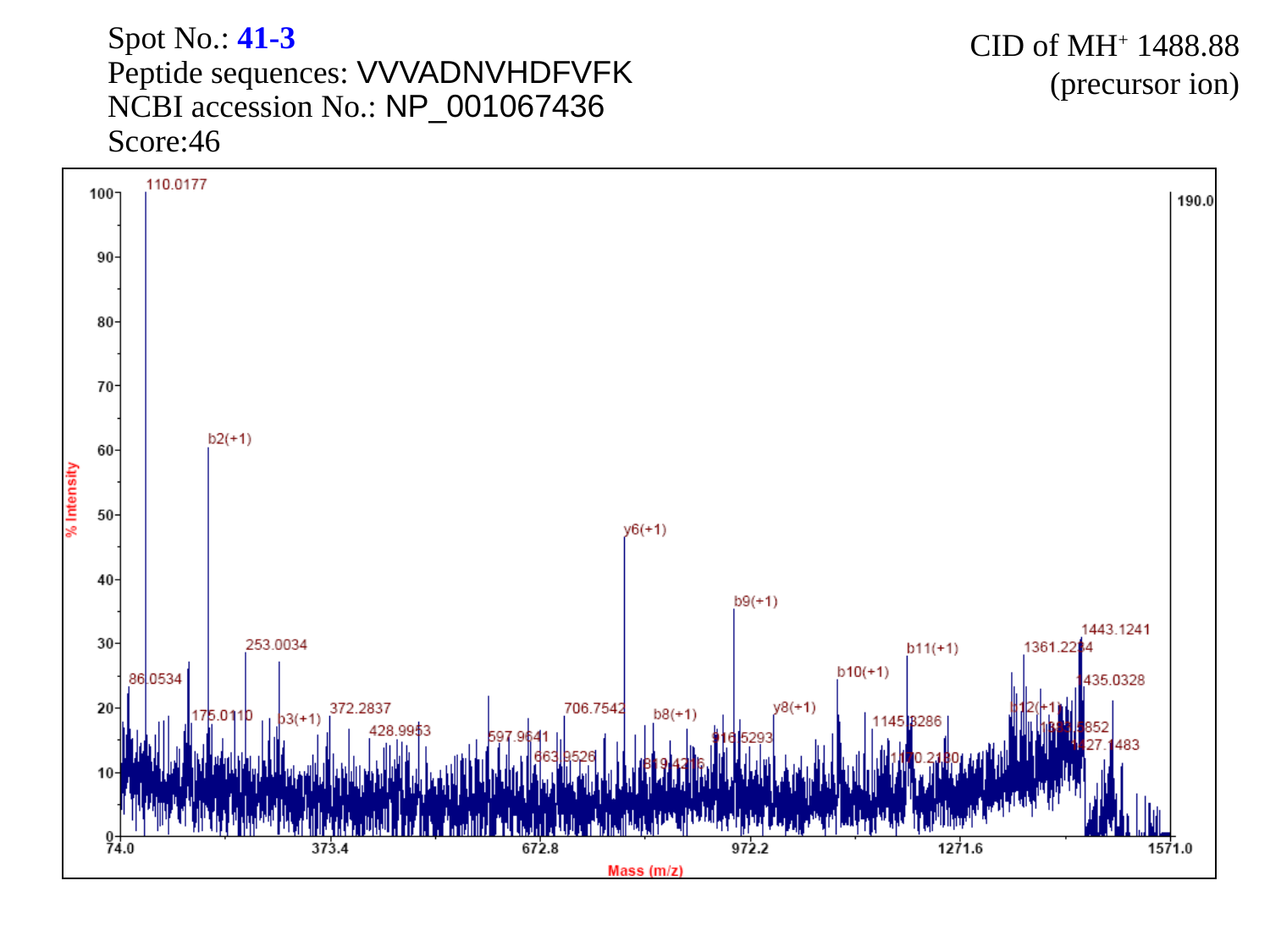

Spot No.: 41-3
Peptide sequences: VVVADNVHDFVFK
NCBI accession No.: NP_001067436
Score:46
CID of MH+ 1488.88 (precursor ion)

## Slide 91
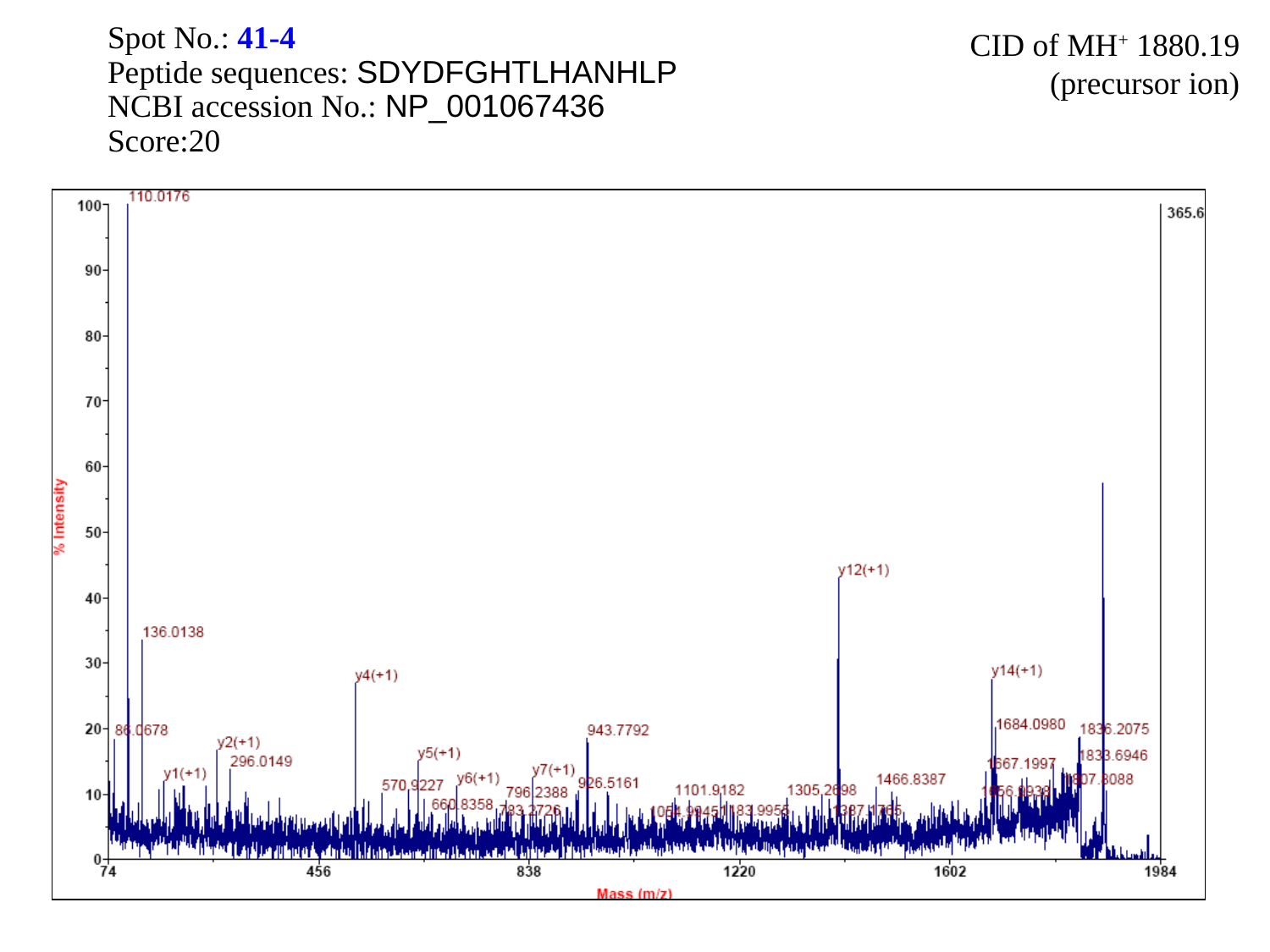

Spot No.: 41-4
Peptide sequences: SDYDFGHTLHANHLP
NCBI accession No.: NP_001067436
Score:20
CID of MH+ 1880.19 (precursor ion)

## Slide 92
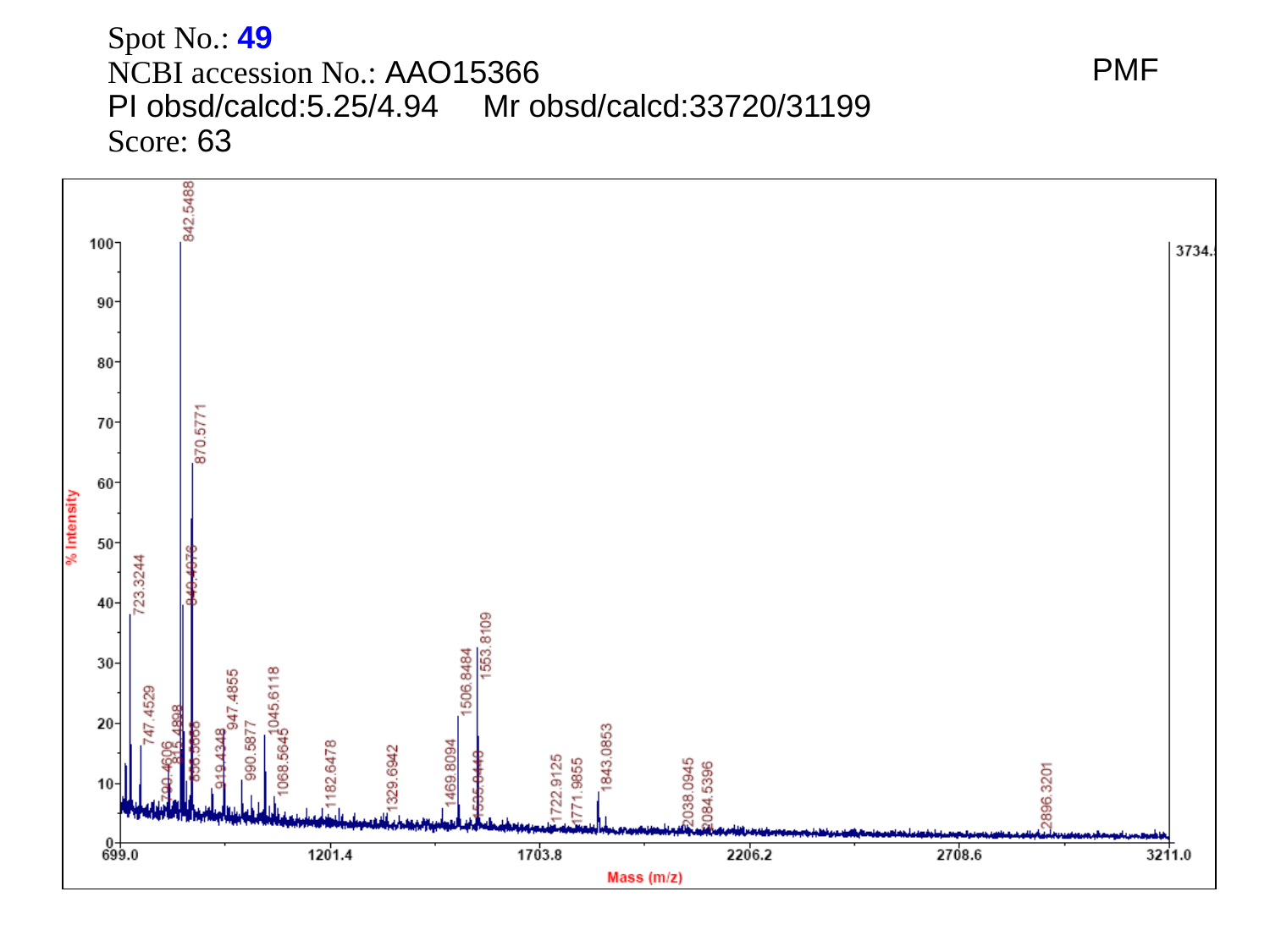

Spot No.: 49
NCBI accession No.: AAO15366
PI obsd/calcd:5.25/4.94 Mr obsd/calcd:33720/31199
Score: 63
PMF

## Slide 93
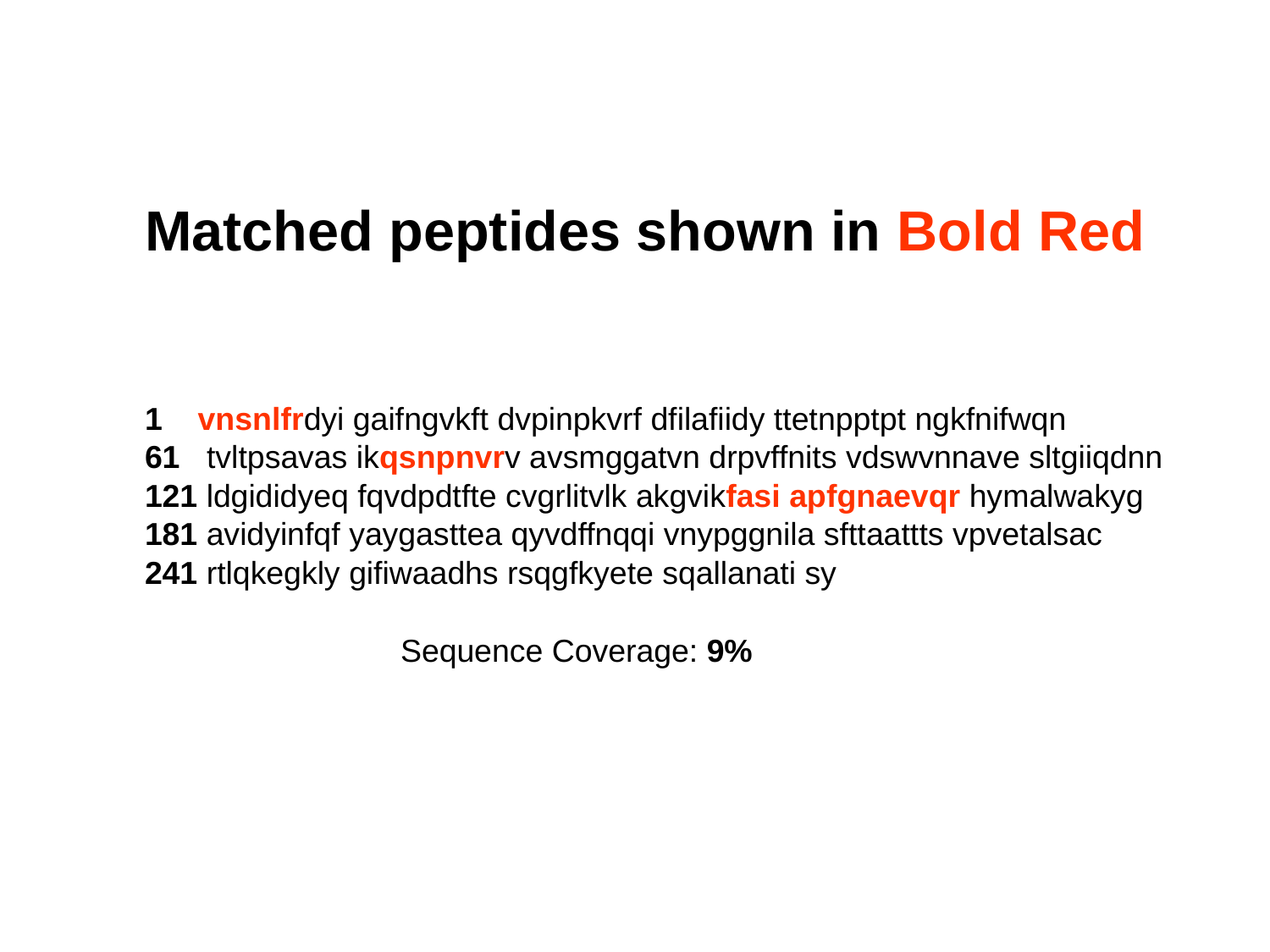

Matched peptides shown in Bold Red
1 vnsnlfrdyi gaifngvkft dvpinpkvrf dfilafiidy ttetnpptpt ngkfnifwqn
61 tvltpsavas ikqsnpnvrv avsmggatvn drpvffnits vdswvnnave sltgiiqdnn
121 ldgididyeq fqvdpdtfte cvgrlitvlk akgvikfasi apfgnaevqr hymalwakyg
181 avidyinfqf yaygasttea qyvdffnqqi vnypggnila sfttaattts vpvetalsac
241 rtlqkegkly gifiwaadhs rsqgfkyete sqallanati sy
# Sequence Coverage: 9%

## Slide 94
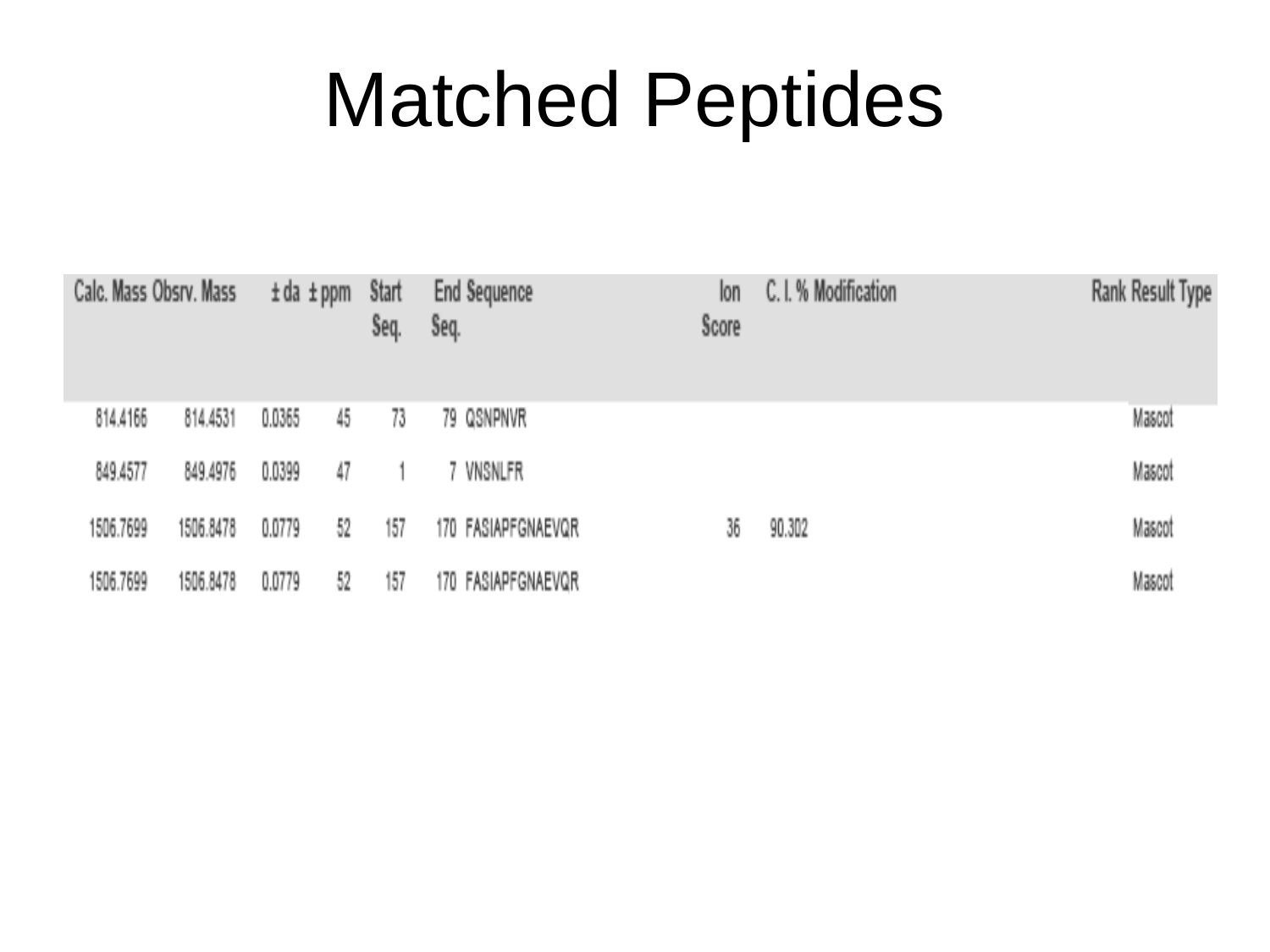

# Matched Peptides

## Slide 95
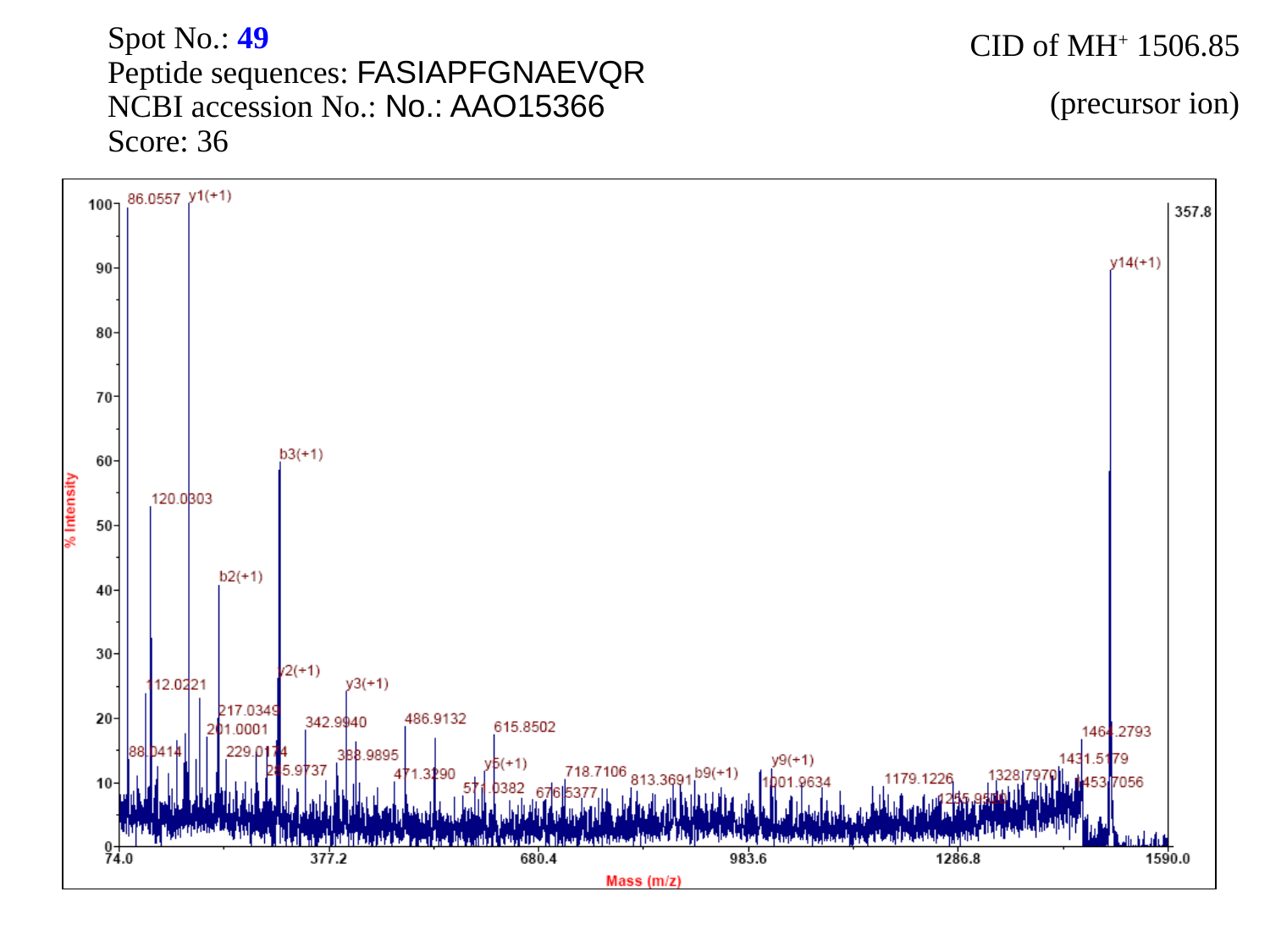

Spot No.: 49
Peptide sequences: FASIAPFGNAEVQR
NCBI accession No.: No.: AAO15366
Score: 36
CID of MH+ 1506.85
(precursor ion)

## Slide 96
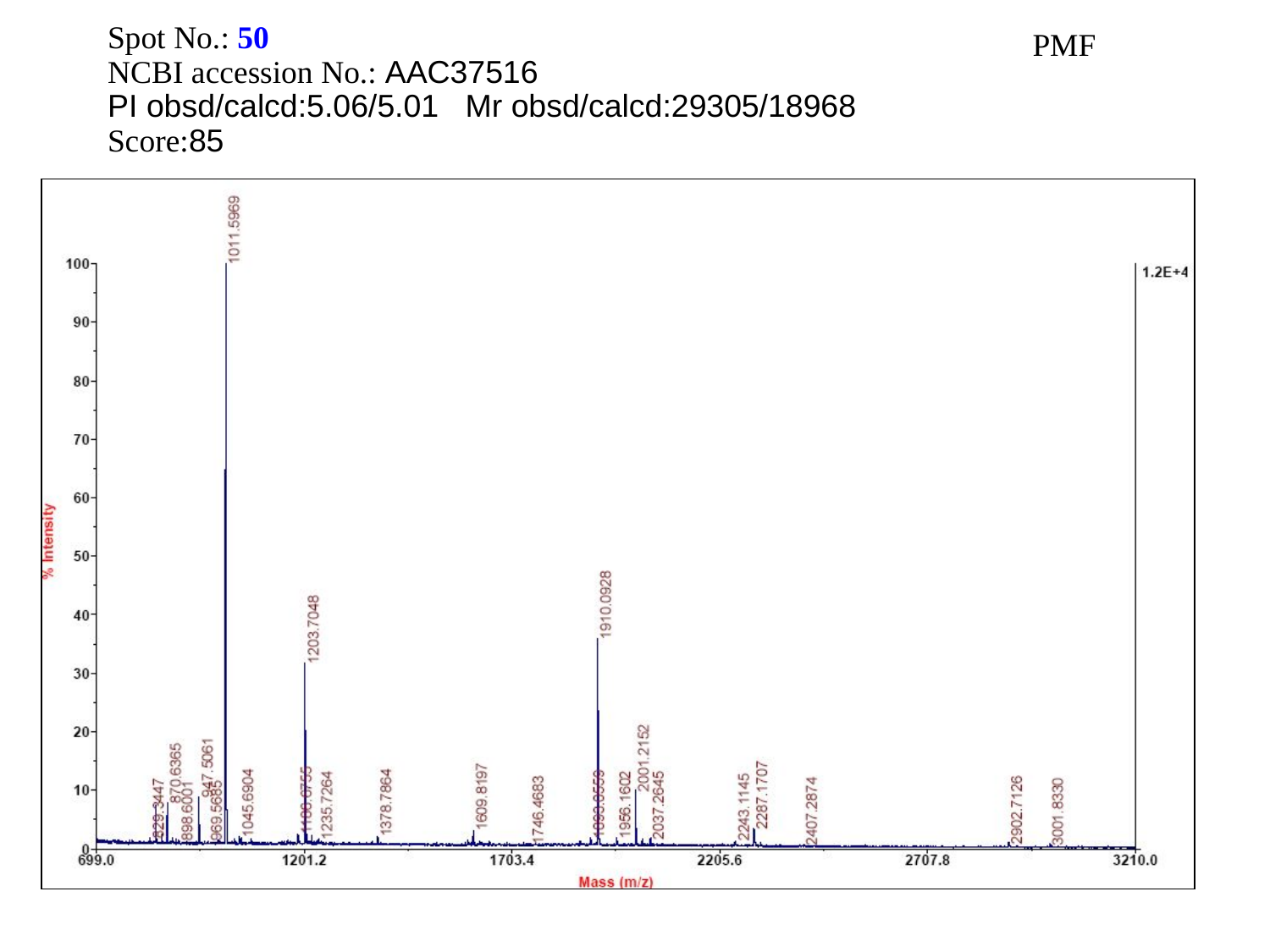

Spot No.: 50
NCBI accession No.: AAC37516
PI obsd/calcd:5.06/5.01 Mr obsd/calcd:29305/18968
Score:85
PMF

## Slide 97
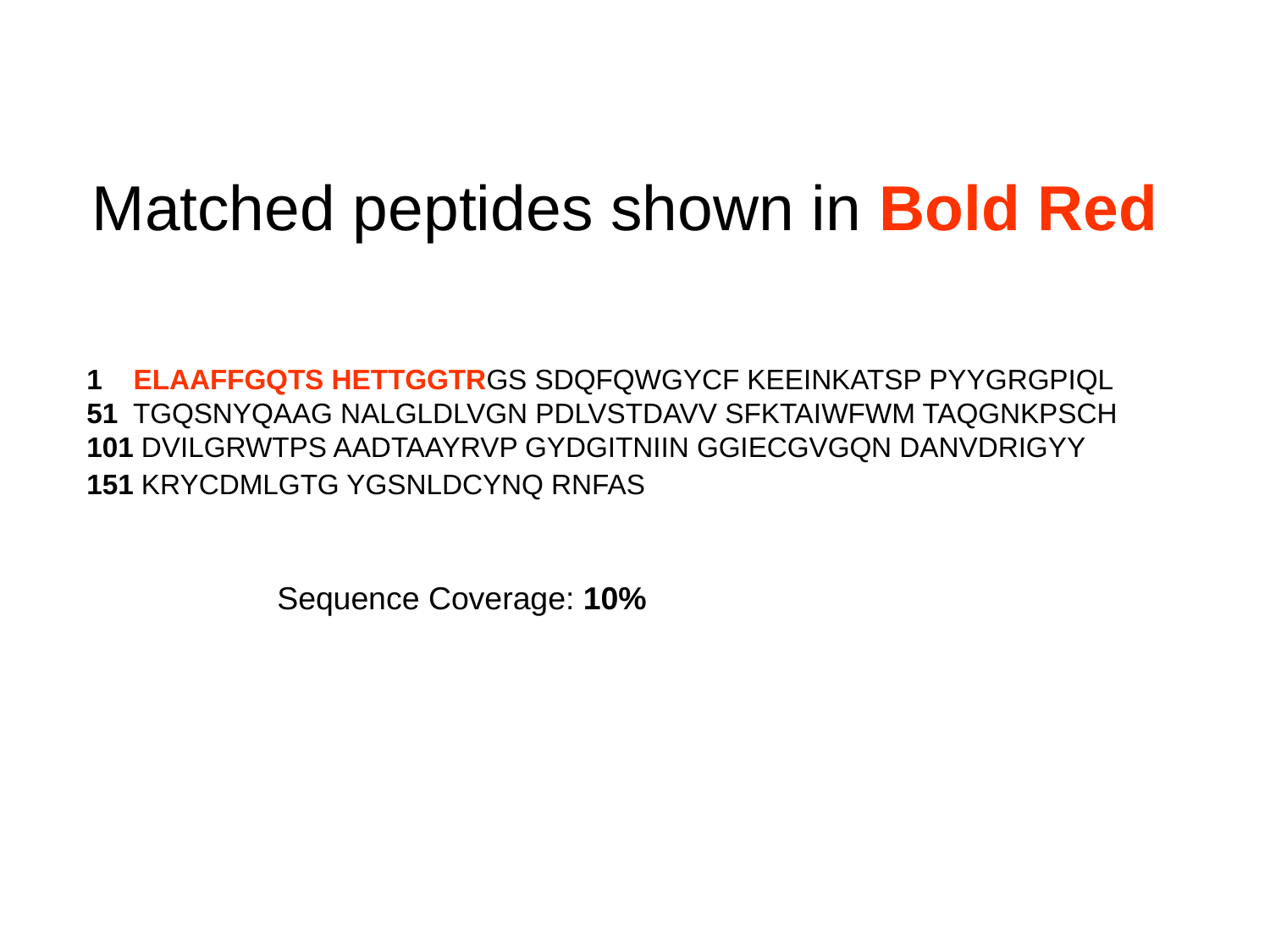

#
Matched peptides shown in Bold Red
1 ELAAFFGQTS HETTGGTRGS SDQFQWGYCF KEEINKATSP PYYGRGPIQL
51 TGQSNYQAAG NALGLDLVGN PDLVSTDAVV SFKTAIWFWM TAQGNKPSCH
101 DVILGRWTPS AADTAAYRVP GYDGITNIIN GGIECGVGQN DANVDRIGYY
151 KRYCDMLGTG YGSNLDCYNQ RNFAS
Sequence Coverage: 10%

## Slide 98
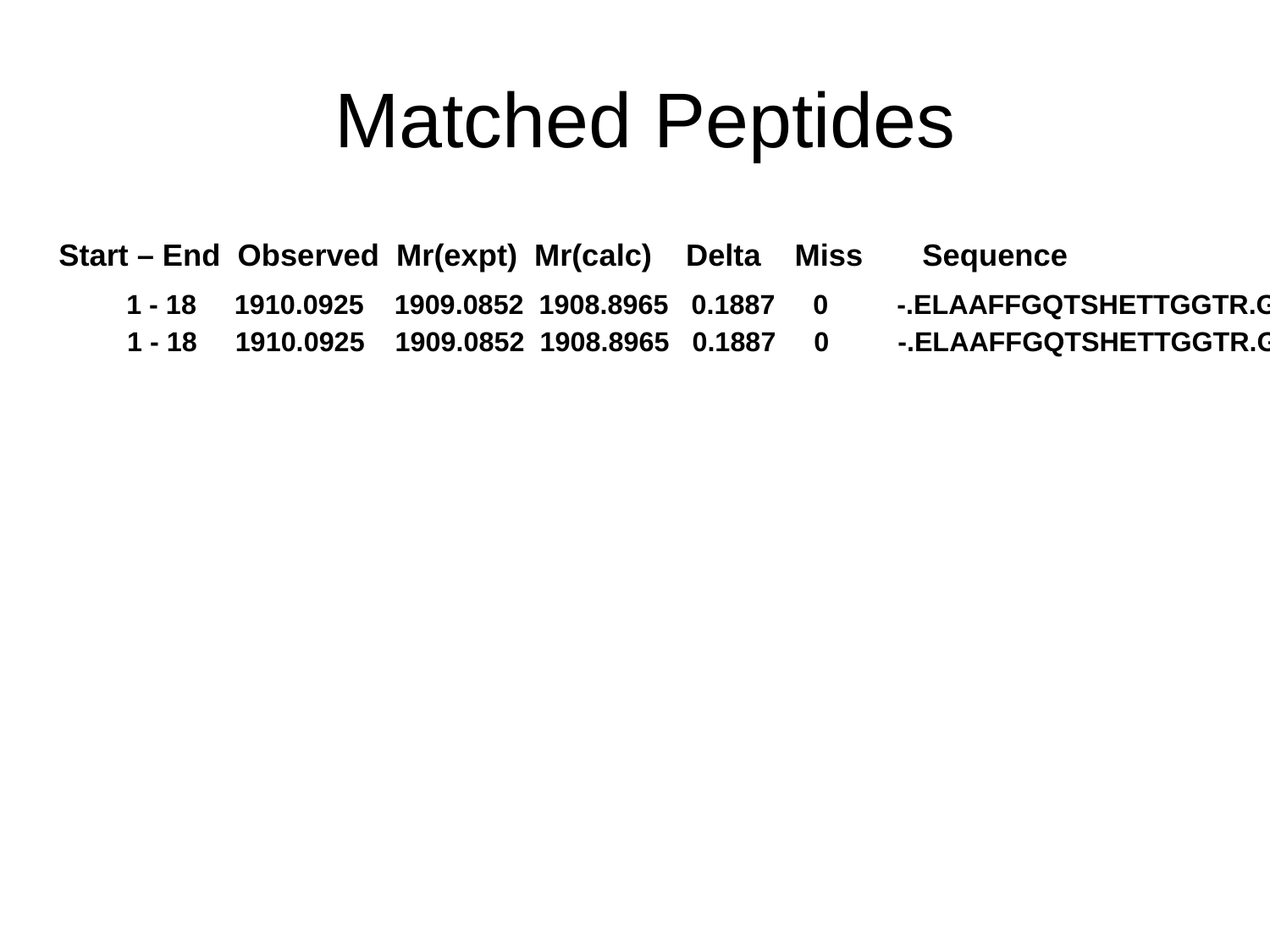

# Matched Peptides
Start – End Observed Mr(expt) Mr(calc) Delta Miss Sequence
 1 - 18 1910.0925 1909.0852 1908.8965 0.1887 0 -.ELAAFFGQTSHETTGGTR.G
 1 - 18 1910.0925 1909.0852 1908.8965 0.1887 0 -.ELAAFFGQTSHETTGGTR.G

## Slide 99
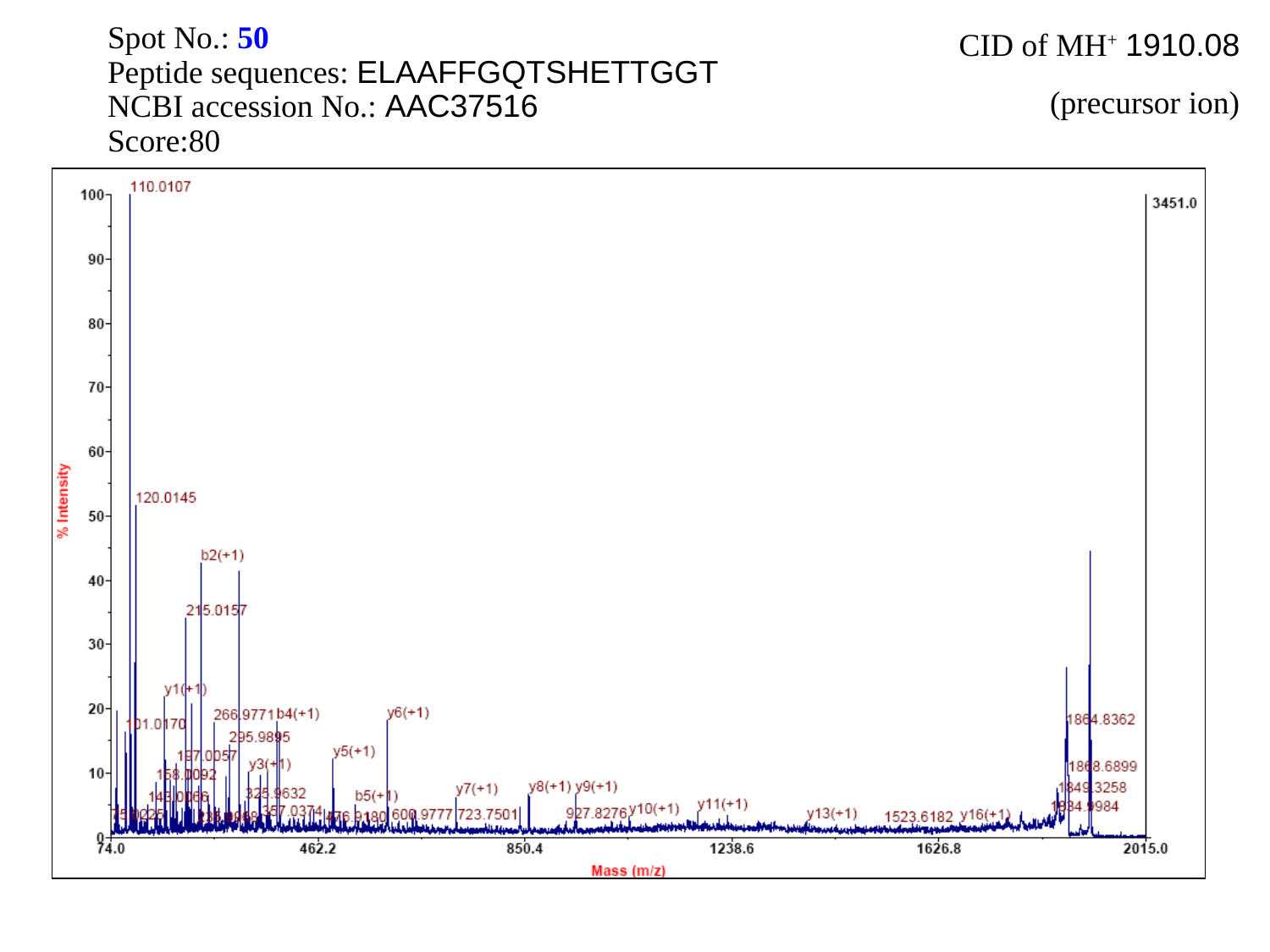

Spot No.: 50
Peptide sequences: ELAAFFGQTSHETTGGT
NCBI accession No.: AAC37516
Score:80
CID of MH+ 1910.08
(precursor ion)

## Slide 100
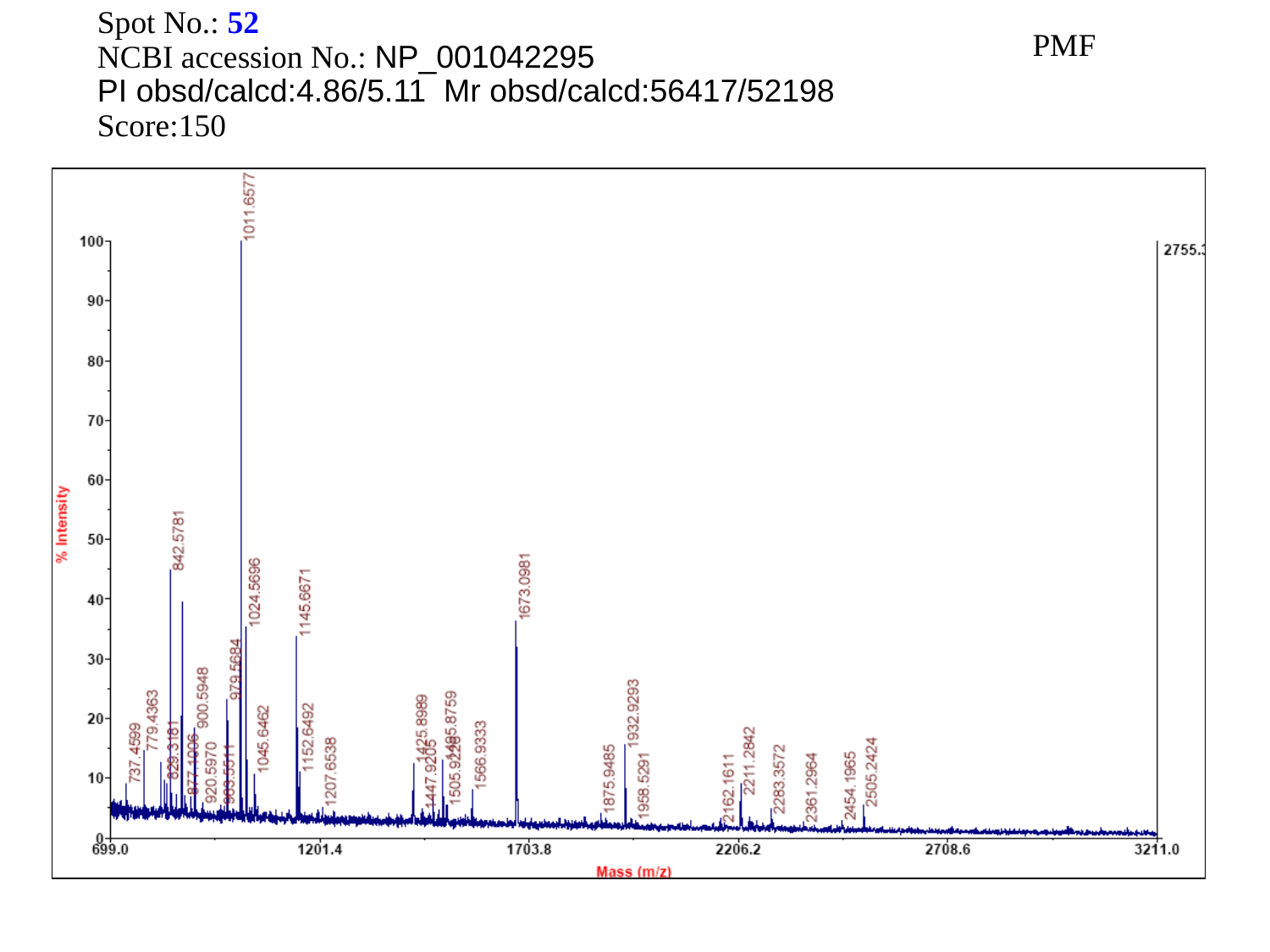

Spot No.: 52
NCBI accession No.: NP_001042295
PI obsd/calcd:4.86/5.11 Mr obsd/calcd:56417/52198
Score:150
PMF

## Slide 101
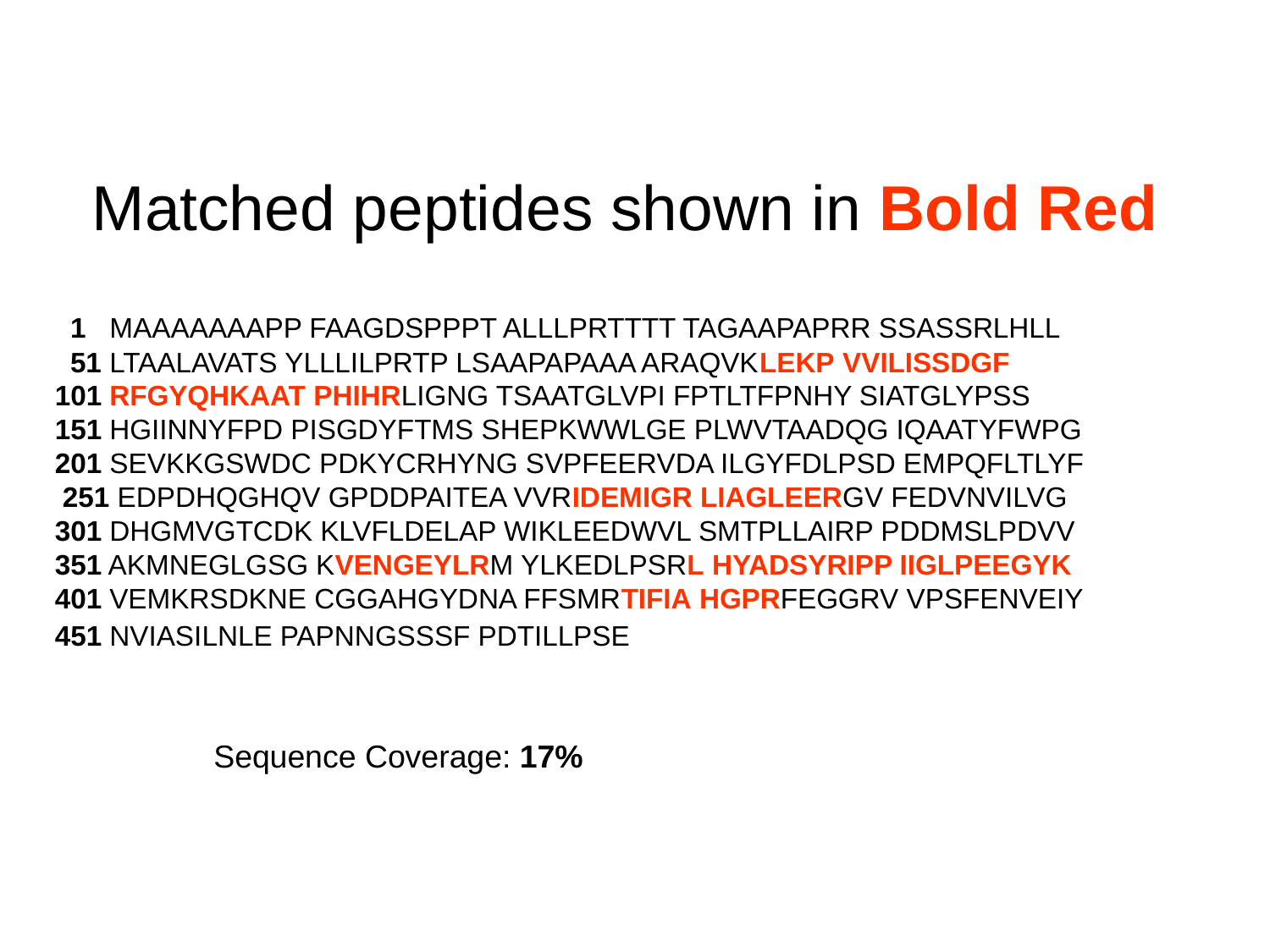

#
Matched peptides shown in Bold Red
 1 MAAAAAAAPP FAAGDSPPPT ALLLPRTTTT TAGAAPAPRR SSASSRLHLL
 51 LTAALAVATS YLLLILPRTP LSAAPAPAAA ARAQVKLEKP VVILISSDGF
101 RFGYQHKAAT PHIHRLIGNG TSAATGLVPI FPTLTFPNHY SIATGLYPSS
151 HGIINNYFPD PISGDYFTMS SHEPKWWLGE PLWVTAADQG IQAATYFWPG
201 SEVKKGSWDC PDKYCRHYNG SVPFEERVDA ILGYFDLPSD EMPQFLTLYF
 251 EDPDHQGHQV GPDDPAITEA VVRIDEMIGR LIAGLEERGV FEDVNVILVG
301 DHGMVGTCDK KLVFLDELAP WIKLEEDWVL SMTPLLAIRP PDDMSLPDVV
351 AKMNEGLGSG KVENGEYLRM YLKEDLPSRL HYADSYRIPP IIGLPEEGYK
401 VEMKRSDKNE CGGAHGYDNA FFSMRTIFIA HGPRFEGGRV VPSFENVEIY
451 NVIASILNLE PAPNNGSSSF PDTILLPSE
Sequence Coverage: 17%

## Slide 102
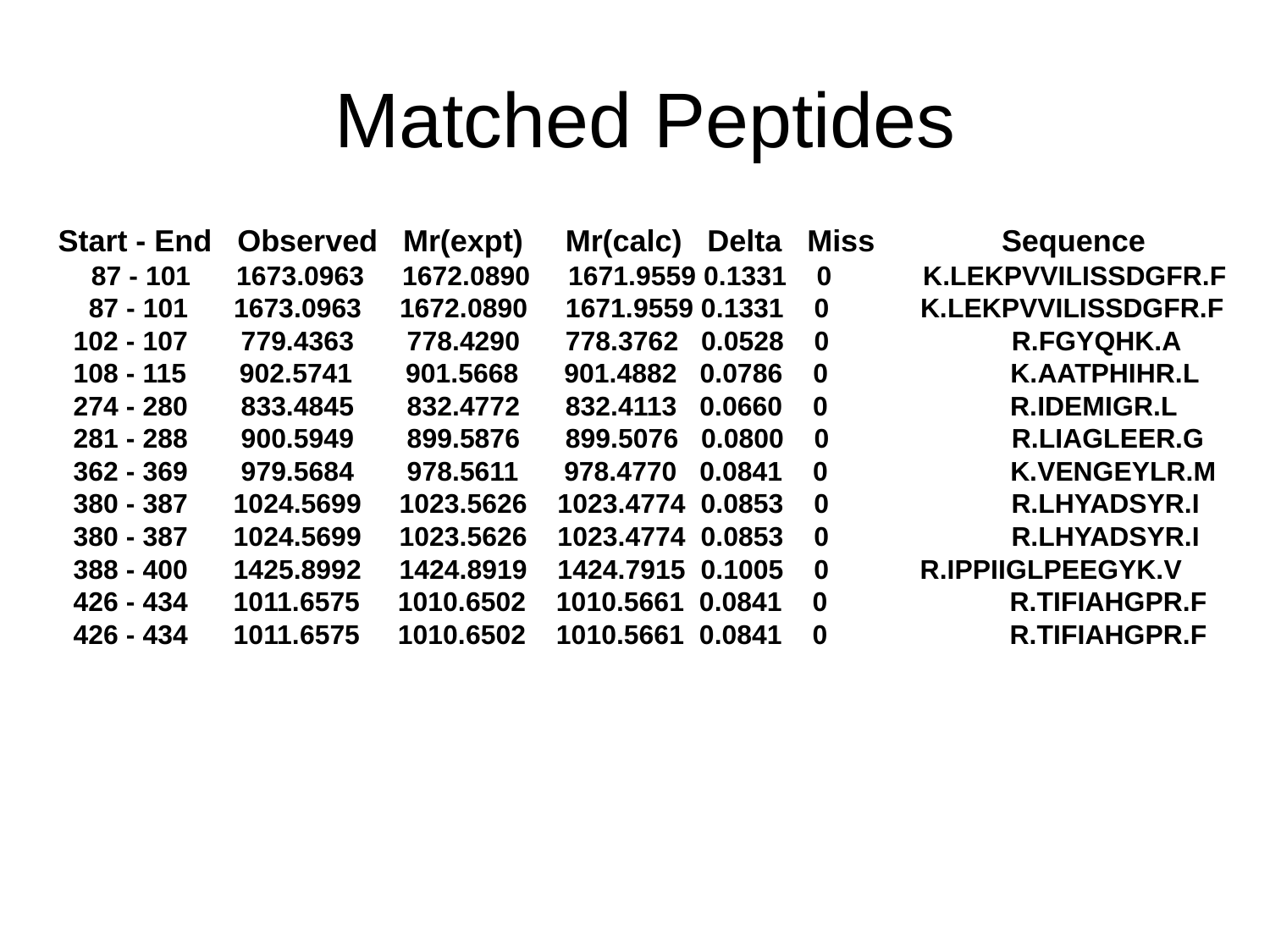

# Matched Peptides
Start - End Observed Mr(expt) Mr(calc) Delta Miss Sequence
 87 - 101 1673.0963 1672.0890 1671.9559 0.1331 0 K.LEKPVVILISSDGFR.F
 87 - 101 1673.0963 1672.0890 1671.9559 0.1331 0 K.LEKPVVILISSDGFR.F
 102 - 107 779.4363 778.4290 778.3762 0.0528 0 R.FGYQHK.A
 108 - 115 902.5741 901.5668 901.4882 0.0786 0 K.AATPHIHR.L
 274 - 280 833.4845 832.4772 832.4113 0.0660 0 R.IDEMIGR.L
 281 - 288 900.5949 899.5876 899.5076 0.0800 0 R.LIAGLEER.G
 362 - 369 979.5684 978.5611 978.4770 0.0841 0 K.VENGEYLR.M
 380 - 387 1024.5699 1023.5626 1023.4774 0.0853 0 R.LHYADSYR.I
 380 - 387 1024.5699 1023.5626 1023.4774 0.0853 0 R.LHYADSYR.I
 388 - 400 1425.8992 1424.8919 1424.7915 0.1005 0 R.IPPIIGLPEEGYK.V
 426 - 434 1011.6575 1010.6502 1010.5661 0.0841 0 R.TIFIAHGPR.F
 426 - 434 1011.6575 1010.6502 1010.5661 0.0841 0 R.TIFIAHGPR.F

## Slide 103
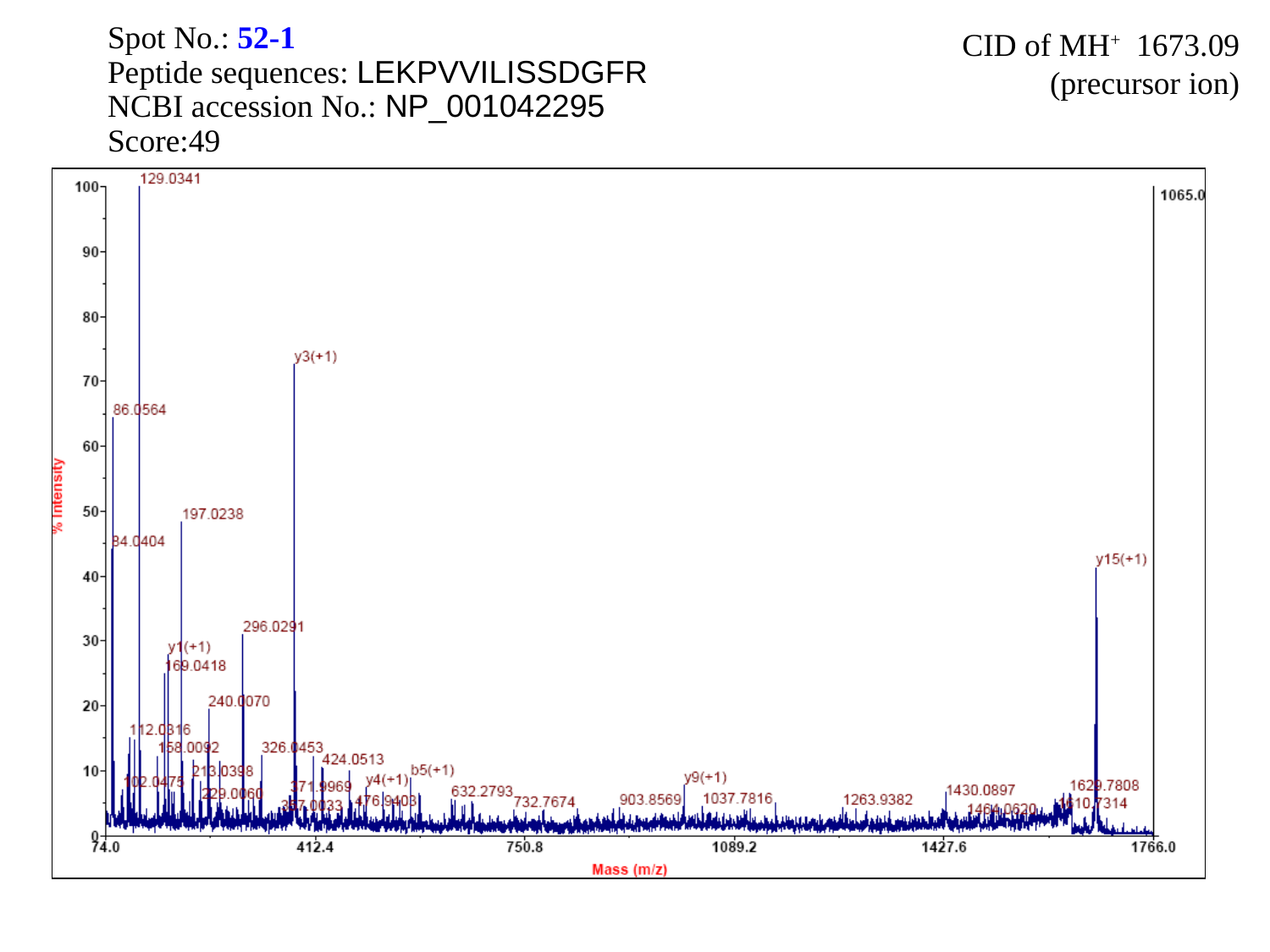

Spot No.: 52-1
Peptide sequences: LEKPVVILISSDGFR
NCBI accession No.: NP_001042295
Score:49
CID of MH+ 1673.09 (precursor ion)

## Slide 104
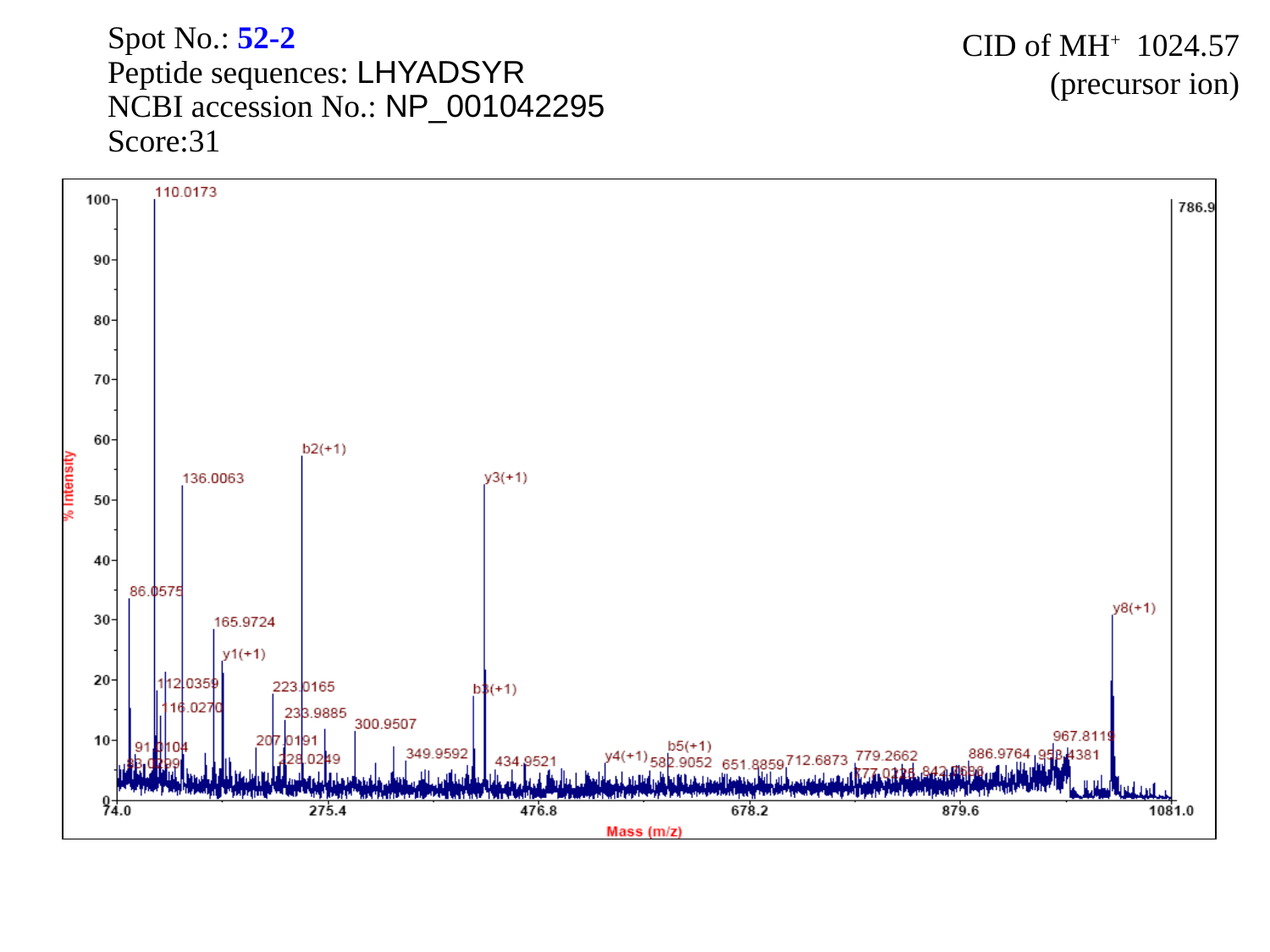

Spot No.: 52-2
Peptide sequences: LHYADSYR
NCBI accession No.: NP_001042295
Score:31
CID of MH+ 1024.57 (precursor ion)

## Slide 105
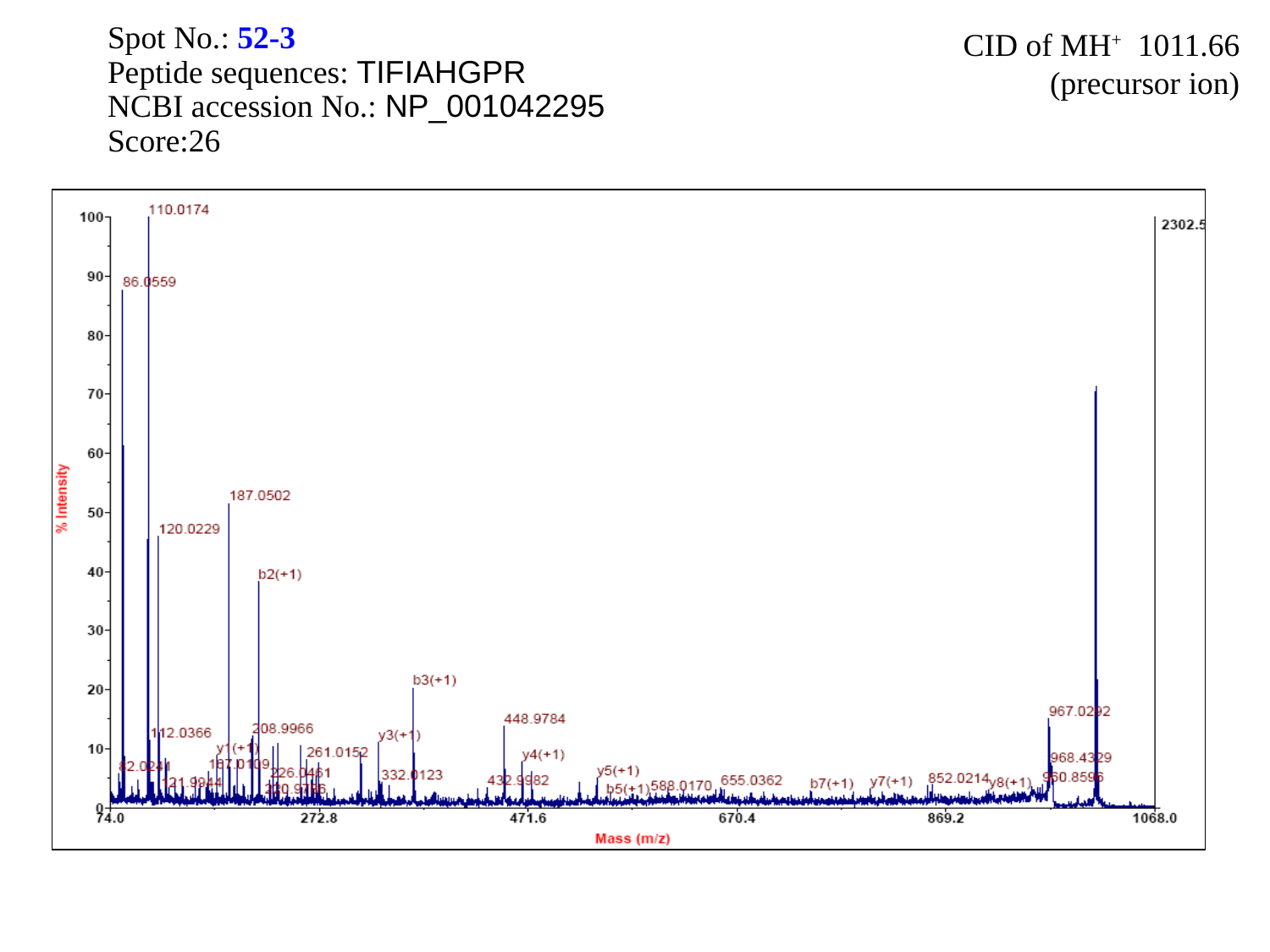

Spot No.: 52-3
Peptide sequences: TIFIAHGPR
NCBI accession No.: NP_001042295
Score:26
CID of MH+ 1011.66 (precursor ion)

## Slide 106
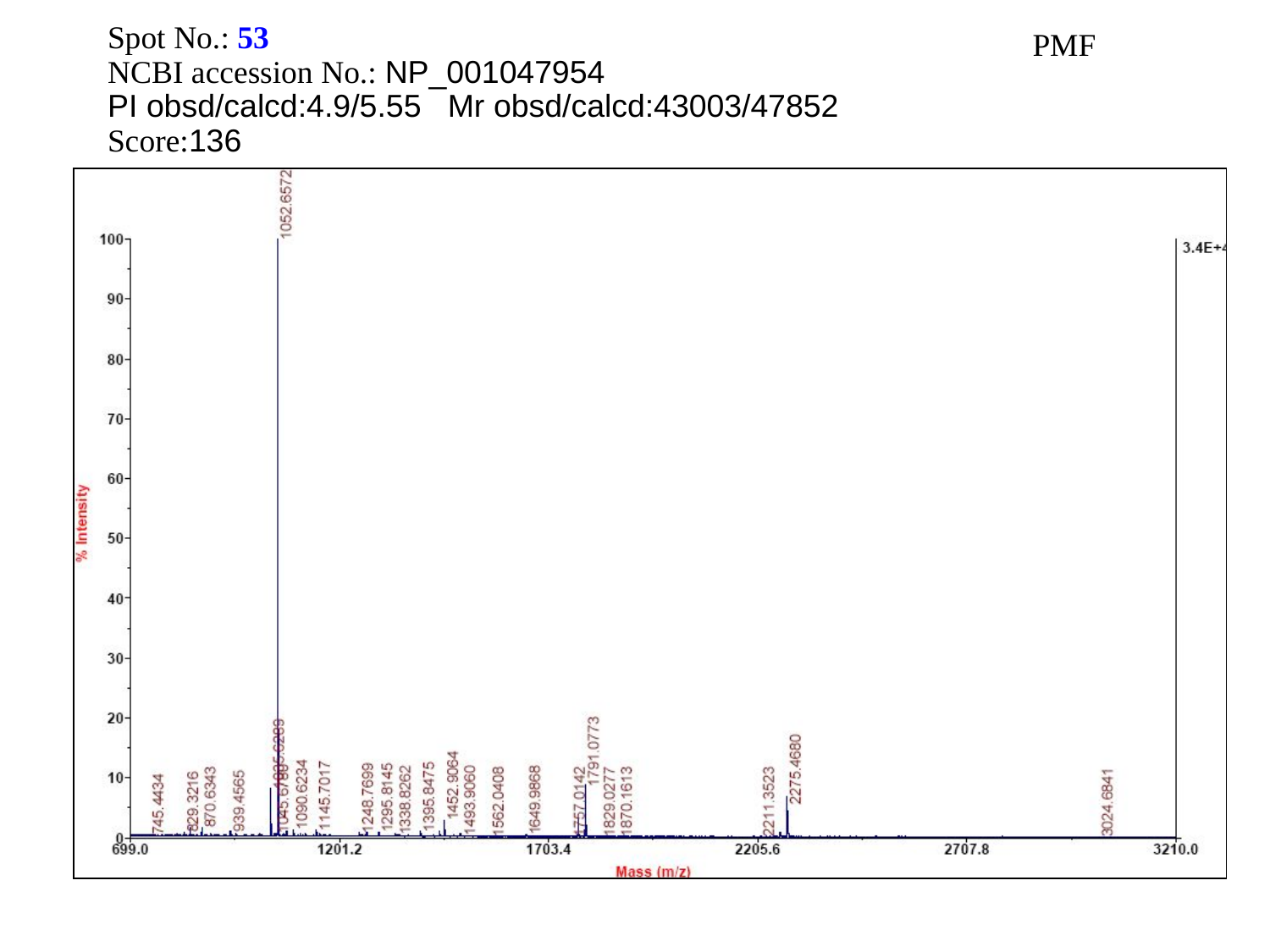

Spot No.: 53
NCBI accession No.: NP_001047954
PI obsd/calcd:4.9/5.55 Mr obsd/calcd:43003/47852
Score:136
PMF

## Slide 107
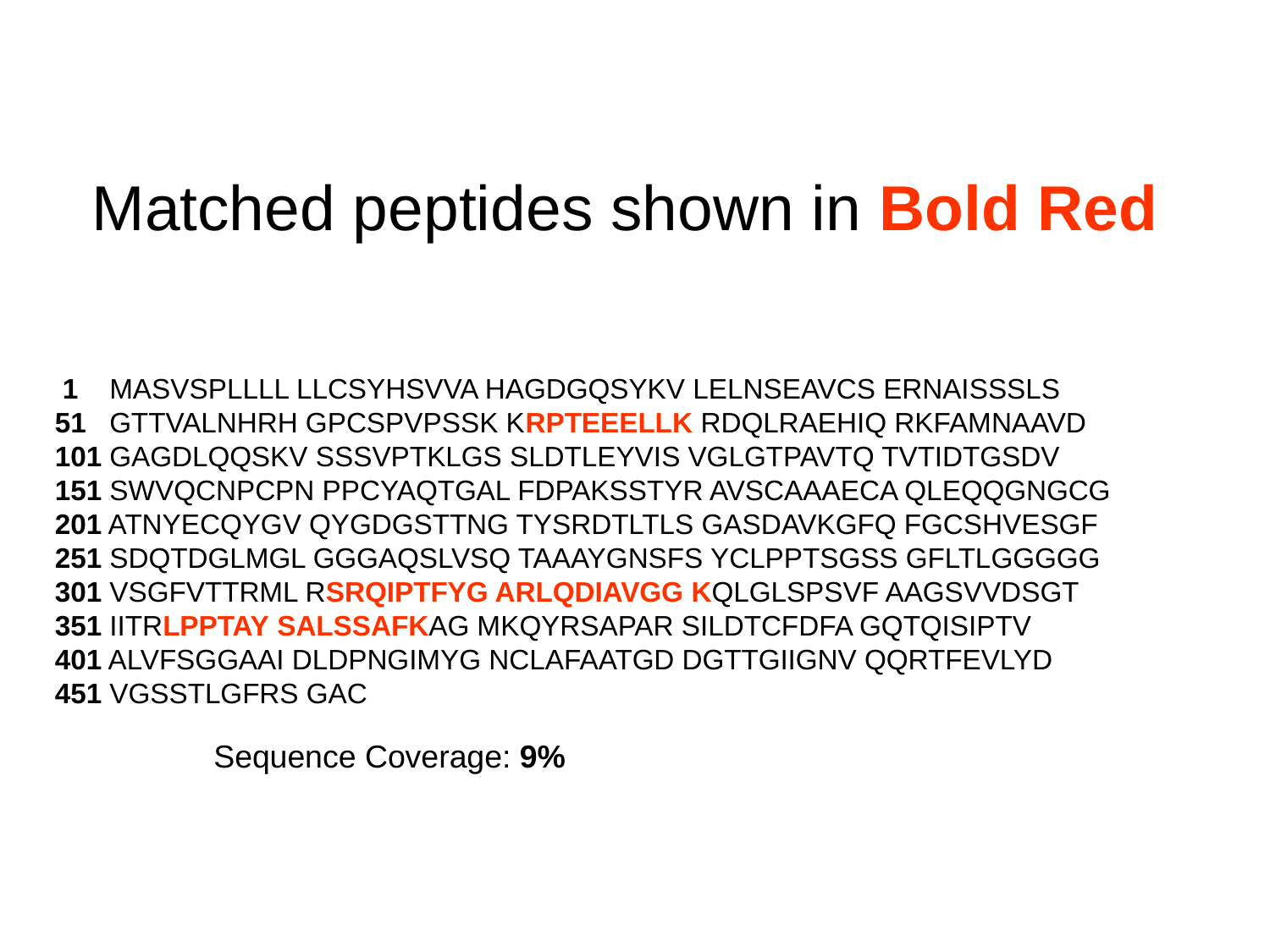

#
Matched peptides shown in Bold Red
 1 MASVSPLLLL LLCSYHSVVA HAGDGQSYKV LELNSEAVCS ERNAISSSLS
51 GTTVALNHRH GPCSPVPSSK KRPTEEELLK RDQLRAEHIQ RKFAMNAAVD
101 GAGDLQQSKV SSSVPTKLGS SLDTLEYVIS VGLGTPAVTQ TVTIDTGSDV
151 SWVQCNPCPN PPCYAQTGAL FDPAKSSTYR AVSCAAAECA QLEQQGNGCG
201 ATNYECQYGV QYGDGSTTNG TYSRDTLTLS GASDAVKGFQ FGCSHVESGF
251 SDQTDGLMGL GGGAQSLVSQ TAAAYGNSFS YCLPPTSGSS GFLTLGGGGG
301 VSGFVTTRML RSRQIPTFYG ARLQDIAVGG KQLGLSPSVF AAGSVVDSGT
351 IITRLPPTAY SALSSAFKAG MKQYRSAPAR SILDTCFDFA GQTQISIPTV
401 ALVFSGGAAI DLDPNGIMYG NCLAFAATGD DGTTGIIGNV QQRTFEVLYD
451 VGSSTLGFRS GAC
Sequence Coverage: 9%

## Slide 108
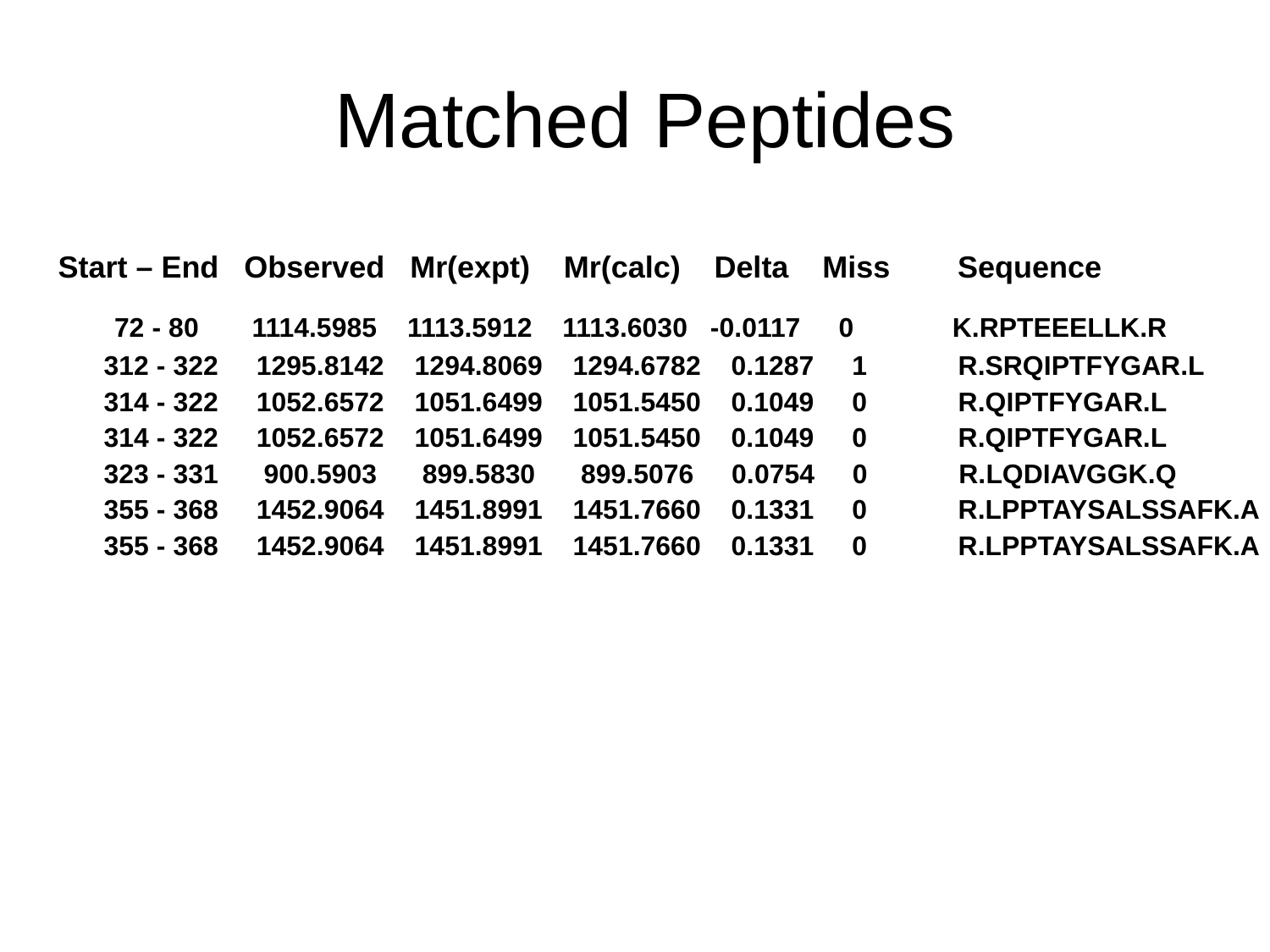

# Matched Peptides
Start – End Observed Mr(expt) Mr(calc) Delta Miss Sequence
 72 - 80 1114.5985 1113.5912 1113.6030 -0.0117 0 K.RPTEEELLK.R
 312 - 322 1295.8142 1294.8069 1294.6782 0.1287 1 R.SRQIPTFYGAR.L
 314 - 322 1052.6572 1051.6499 1051.5450 0.1049 0 R.QIPTFYGAR.L
 314 - 322 1052.6572 1051.6499 1051.5450 0.1049 0 R.QIPTFYGAR.L
 323 - 331 900.5903 899.5830 899.5076 0.0754 0 R.LQDIAVGGK.Q
 355 - 368 1452.9064 1451.8991 1451.7660 0.1331 0 R.LPPTAYSALSSAFK.A
 355 - 368 1452.9064 1451.8991 1451.7660 0.1331 0 R.LPPTAYSALSSAFK.A

## Slide 109
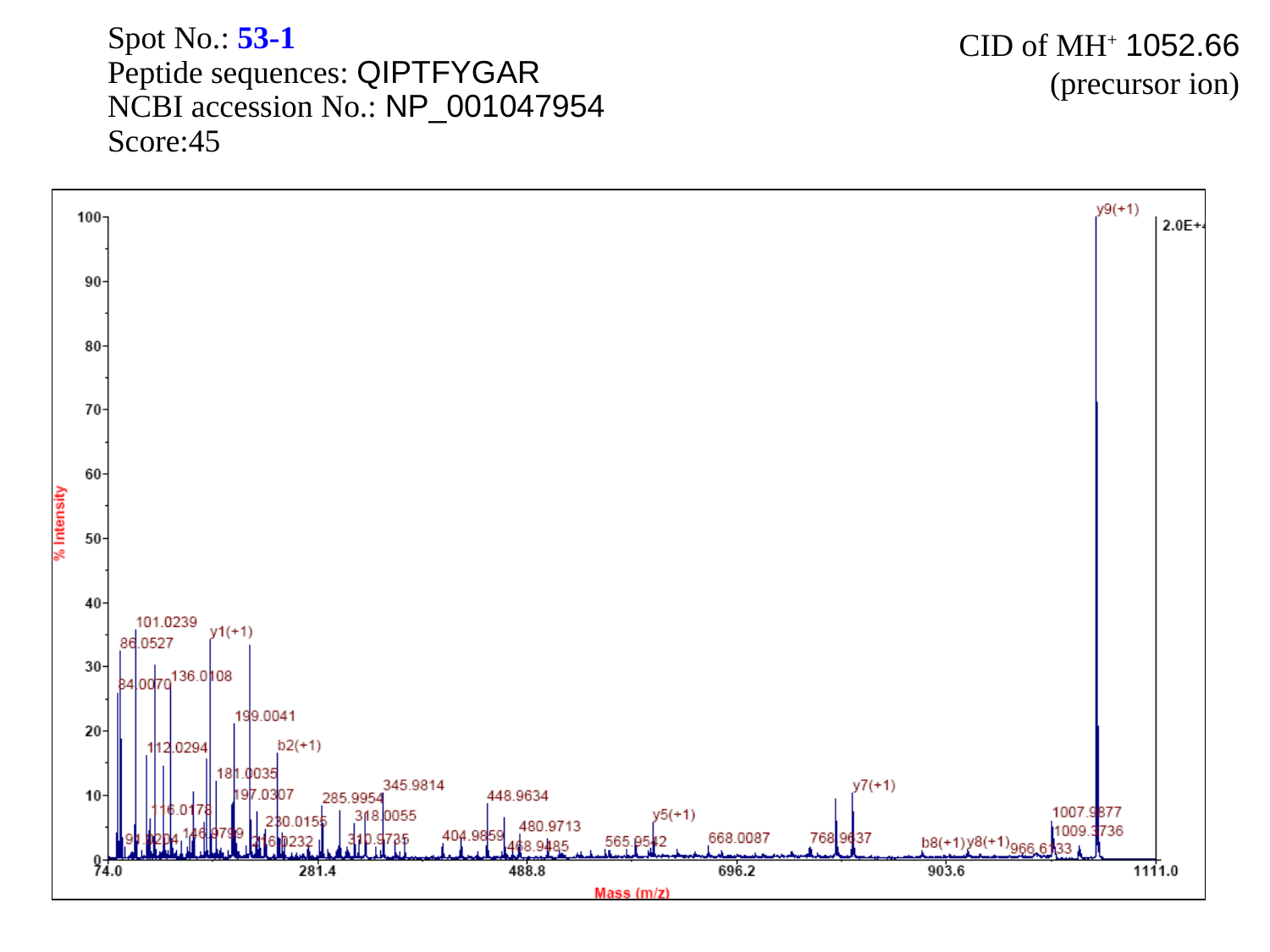

Spot No.: 53-1
Peptide sequences: QIPTFYGAR
NCBI accession No.: NP_001047954
Score:45
CID of MH+ 1052.66 (precursor ion)

## Slide 110
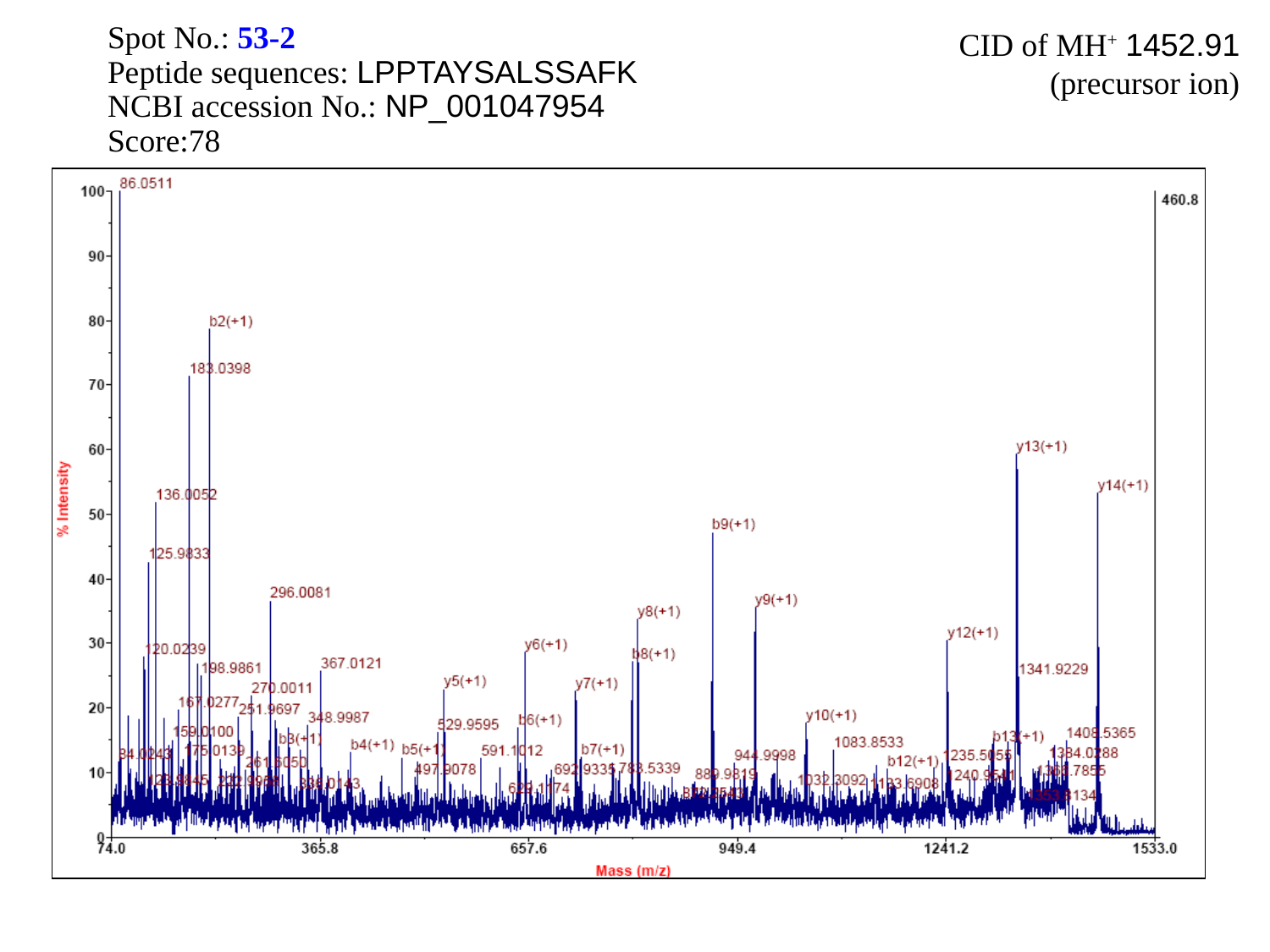

Spot No.: 53-2
Peptide sequences: LPPTAYSALSSAFK
NCBI accession No.: NP_001047954
Score:78
CID of MH+ 1452.91 (precursor ion)
